# Supplementary figures and images for: Identification of hub genes and small molecule therapeutic drugs related to breast cancer with comprehensive bioinformatics analysis
Source: PeerJ. 2020 Sep 29;8:e9946. doi: 10.7717/peerj.9946 (PMC7556247; doi:10.7717/peerj.9946)

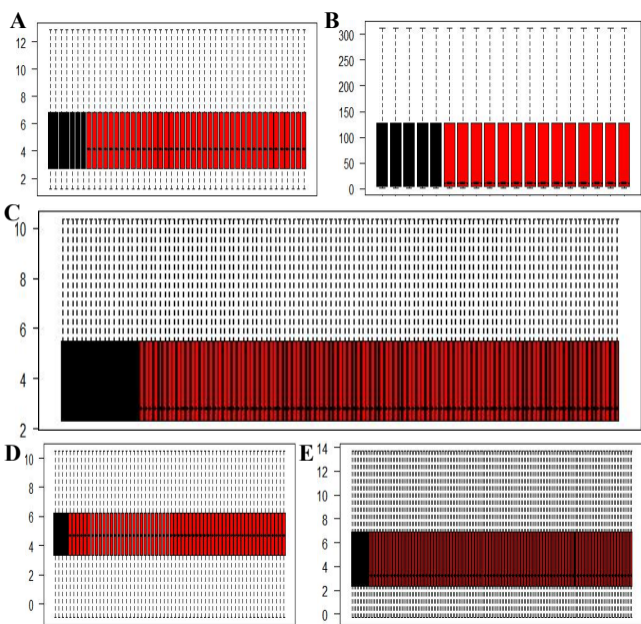

Supplement: Supplemental Information 1 — (A) Normalization of GSE3744. (B) Normalization of GSE21422. (C) Normalization of GSE42568. (D) Normalization of GSE61304 . (E) Normalization of GSE65194. Vertical axis: expression value; Horizontal axis: sample list. Black: normal; Red: tumor. [file peerj-08-9946-s001.pdf]

**A**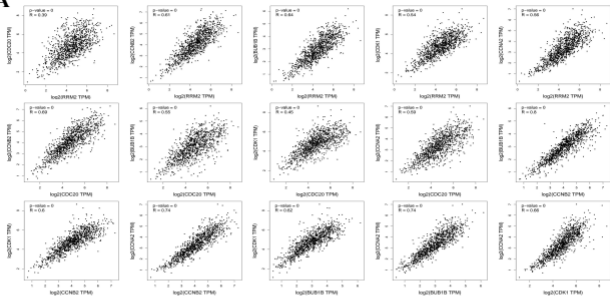**B**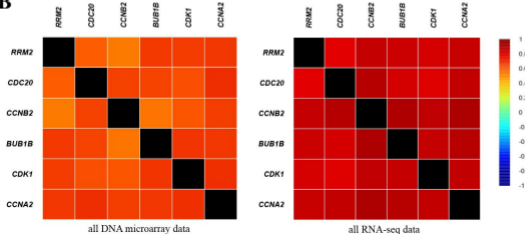

Supplement: Supplemental Information 3 — (A) Expression correlation analysis of six hub genes based on GEPIA2 database. (B) Expression correlation verification of six hub genes in all DNA microarray data and RNA-seq data related to breast cancer based on BC-GenExMiner tool. [file peerj-08-9946-s003.pdf]

A

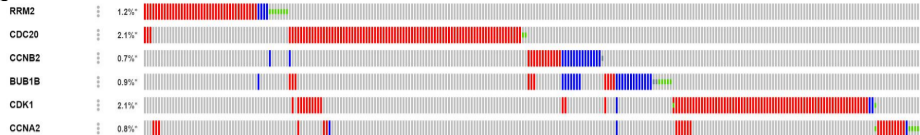

B

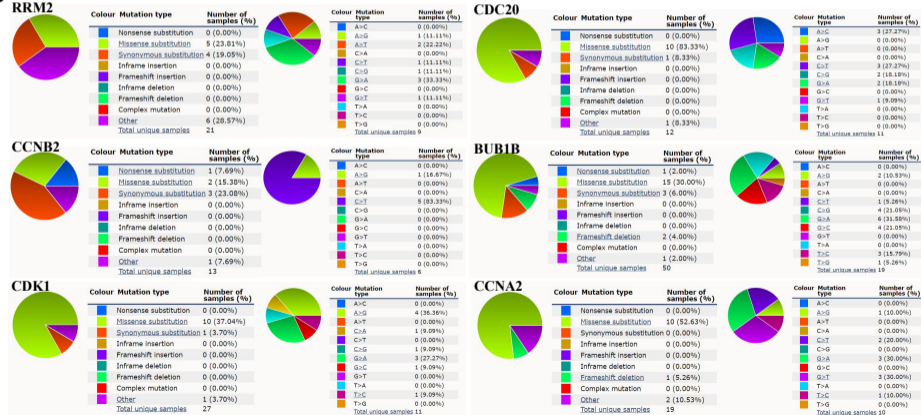

Supplement: Supplemental Information 4 [file peerj-08-9946-s004.pdf]

A

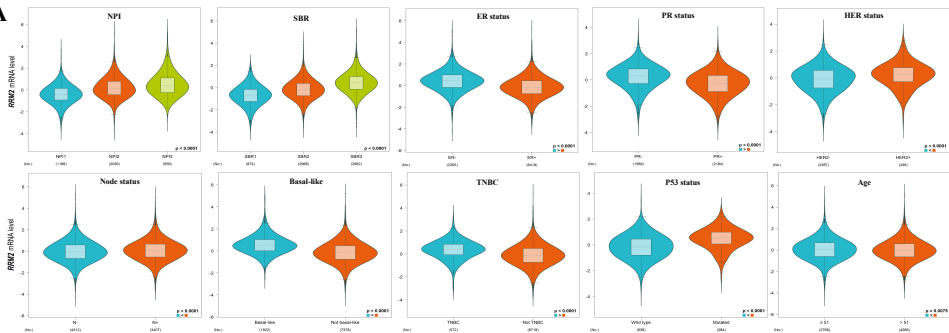

B

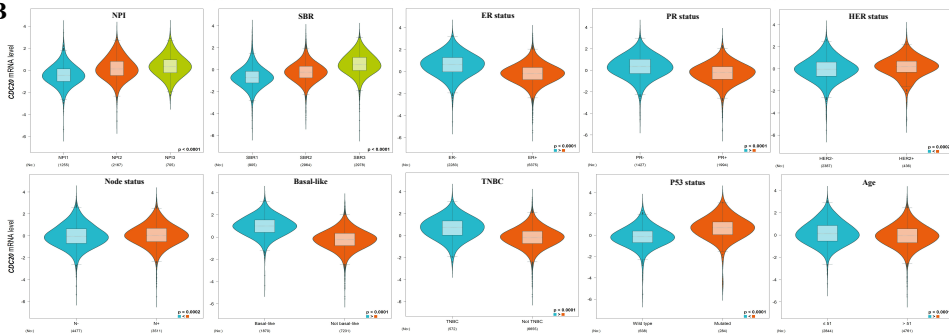

Supplement: Supplemental Information 5 [file peerj-08-9946-s005.pdf]

A

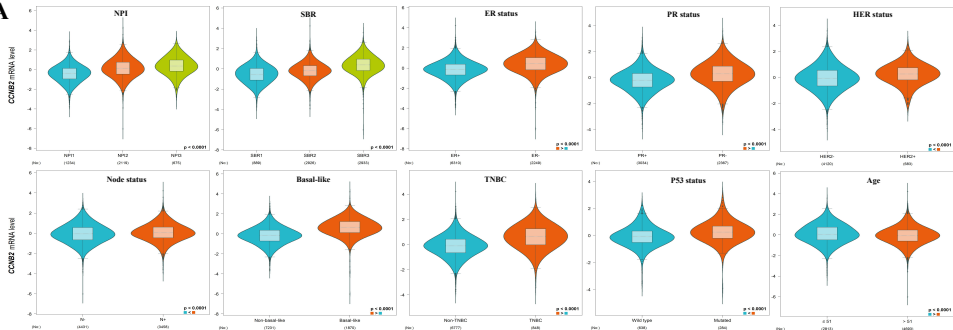

B

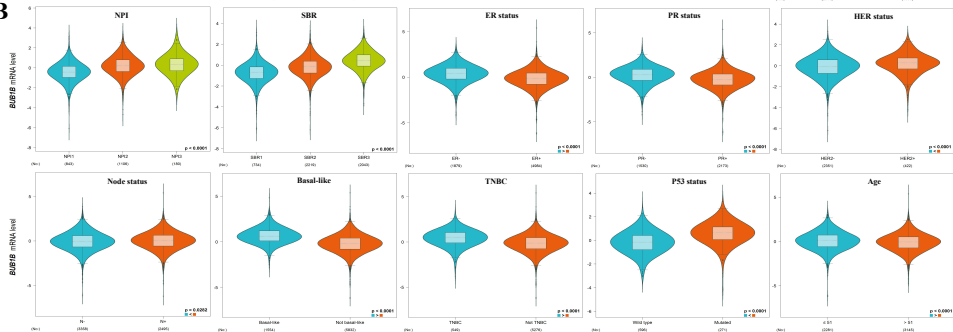

Supplement: Supplemental Information 6 [file peerj-08-9946-s006.pdf]

A

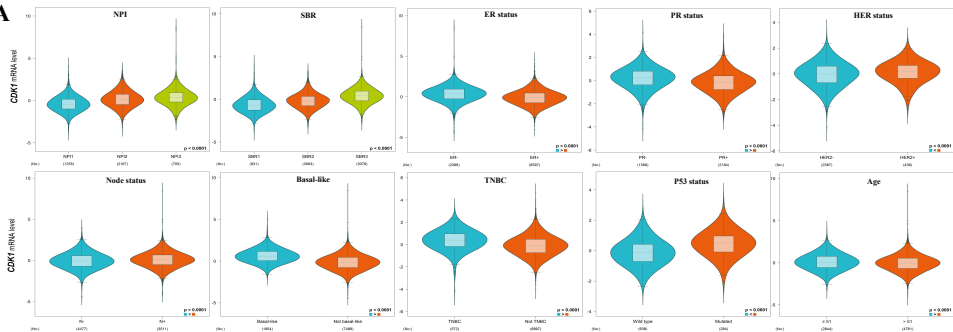

B

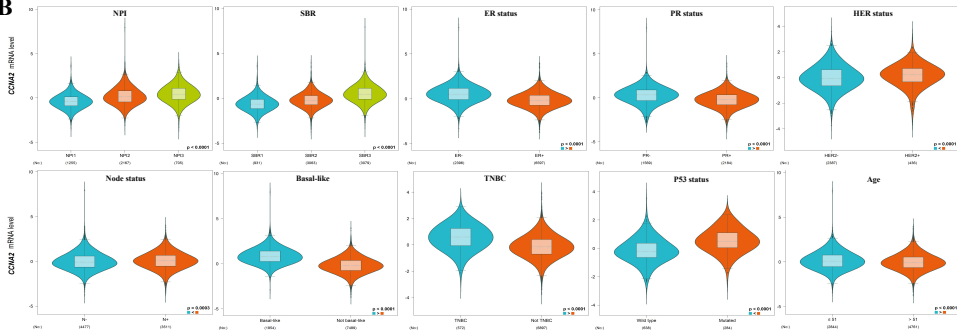

Supplement: Supplemental Information 7 [file peerj-08-9946-s007.pdf]

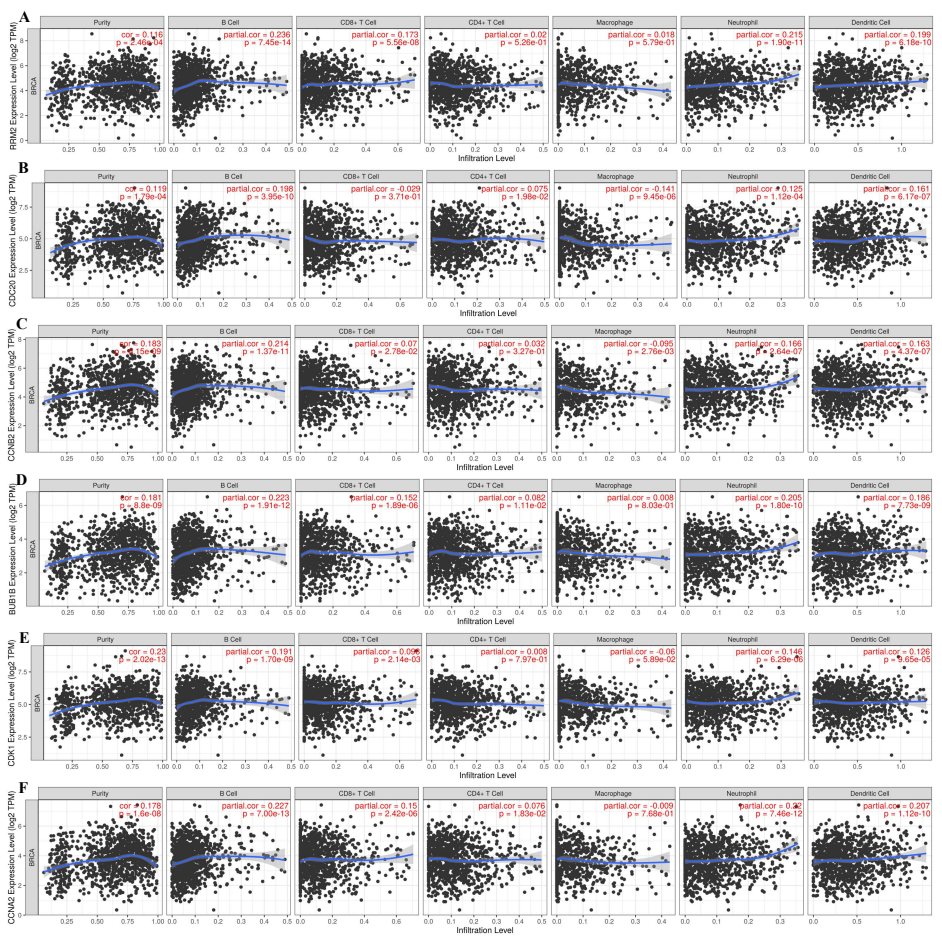

Supplement: Supplemental Information 8 — (A) RRM2. (B) CDC20. (C) CCNB2. (D) BUB1B. (E) CDK1. (F) CCNA2. [file peerj-08-9946-s008.pdf]

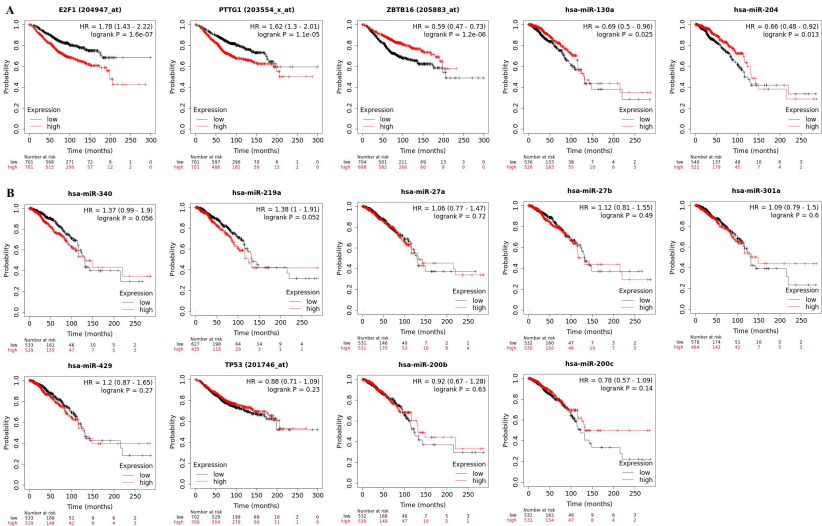

Supplement: Supplemental Information 9 — (A) Reporter regulatory factors that are significantly related to OS. (B) Some reporter regulatory factors that have no statistically significant correlation with OS. [file peerj-08-9946-s009.pdf]
