# Supplementary material for: Identification of hub genes and small molecule therapeutic drugs related to breast cancer with comprehensive bioinformatics analysis
Source: PeerJ. 2020 Sep 29;8:e9946. doi: 10.7717/peerj.9946 (PMC7556247; doi:10.7717/peerj.9946)
Supplement: Supplemental Information 10 [file peerj-08-9946-s010.docx]

| **ID** | **Log2FC** | **adj.P.Val** |
| --- | --- | --- |
| PIP | -6.16 | 1.41E-03 |
| SCARA5 | -6.07 | 1.33E-18 |
| ECRG4 | -5.82 | 1.46E-06 |
| LINC00993 | -5.79 | 1.66E-04 |
| MUCL1 | -5.72 | 2.26E-03 |
| ADH1B | -5.66 | 1.18E-06 |
| OGN | -5.4 | 2.32E-07 |
| PGR | -5.3 | 3.06E-04 |
| ABCA8 | -5.2 | 1.28E-12 |
| SCGB3A1 | -5.12 | 2.14E-07 |
| SCUBE2 | -5.05 | 2.75E-03 |
| SCGB2A2 | -5.02 | 2.72E-02 |
| OXTR | -4.97 | 1.33E-18 |
| SCGB1D2 | -4.95 | 1.25E-02 |
| PI15 | -4.94 | 1.31E-04 |
| MFAP4 | -4.93 | 6.62E-10 |
| CITED1 | -4.92 | 5.74E-08 |
| FXYD1 | -4.89 | 1.40E-20 |
| MAMDC2 | -4.82 | 7.14E-16 |
| CNN1 | -4.79 | 6.13E-11 |
| MYH11 | -4.78 | 7.19E-12 |
| PTN | -4.72 | 6.86E-09 |
| LAMA3 | -4.7 | 2.98E-05 |
| TP63 | -4.68 | 1.85E-07 |
| SAMD5 | -4.67 | 4.70E-18 |
| NTRK2 | -4.61 | 4.17E-05 |
| ACKR1 | -4.59 | 1.27E-06 |
| KRT14 | -4.56 | 3.58E-03 |
| HOXA5 | -4.56 | 4.54E-09 |
| STC2 | -4.56 | 4.51E-08 |
| WIF1 | -4.55 | 7.43E-05 |
| CARMN | -4.5 | 2.01E-08 |
| FAM189A2 | -4.39 | 7.63E-10 |
| FOSB | -4.39 | 1.23E-06 |
| ZBTB16 | -4.36 | 9.79E-15 |
| LYVE1 | -4.35 | 9.26E-13 |
| FABP4 | -4.33 | 1.92E-04 |
| SYNM | -4.32 | 6.18E-04 |
| MIR205HG | -4.29 | 3.42E-10 |
| KRT15 | -4.27 | 7.49E-04 |
| GPC3 | -4.26 | 2.82E-13 |
| CXCL14 | -4.26 | 3.00E-04 |
| LMOD1 | -4.22 | 4.59E-09 |
| SFRP1 | -4.22 | 3.02E-03 |
| ALOX15B | -4.19 | 3.19E-05 |
| FMO2 | -4.17 | 3.81E-05 |
| PLIN1 | -4.16 | 1.72E-07 |
| KIT | -4.15 | 9.15E-06 |
| IGFBP6 | -4.15 | 5.76E-11 |
| APOD | -4.15 | 1.28E-03 |
| ANKRD30A | -4.13 | 5.69E-03 |
| AK5 | -4.13 | 1.01E-13 |
| KRT5 | -4.1 | 4.74E-04 |
| CHRDL1 | -4.03 | 1.24E-07 |
| TGFBR3 | -3.98 | 9.01E-06 |
| CFD | -3.96 | 1.26E-05 |
| PDGFD | -3.96 | 4.26E-07 |
| PAMR1 | -3.96 | 3.03E-15 |
| CALML3 | -3.94 | 1.75E-06 |
| ADIPOQ | -3.91 | 7.16E-05 |
| KLHL13 | -3.91 | 2.57E-05 |
| CXCL12 | -3.89 | 1.75E-06 |
| CLDN8 | -3.88 | 2.99E-04 |
| OPRPN | -3.83 | 3.69E-06 |
| PIGR | -3.77 | 9.10E-10 |
| SEMA6D | -3.75 | 1.64E-05 |
| CLDN11 | -3.74 | 4.69E-06 |
| KLHL29 | -3.73 | 3.57E-12 |
| KCNMB1 | -3.71 | 1.41E-05 |
| COL17A1 | -3.69 | 1.40E-20 |
| HBB | -3.67 | 7.22E-05 |
| SEMA5A | -3.67 | 1.41E-08 |
| RBP4 | -3.66 | 1.73E-08 |
| TFAP2B | -3.65 | 3.74E-02 |
| FHL1 | -3.64 | 4.60E-06 |
| MATN2 | -3.63 | 6.01E-04 |
| FGF1 | -3.62 | 4.13E-12 |
| KLK5 | -3.62 | 1.23E-02 |
| SLC26A3 | -3.61 | 7.47E-10 |
| MYBPC1 | -3.56 | 2.42E-02 |
| IGF1 | -3.55 | 4.65E-06 |
| RUNX1T1 | -3.51 | 5.77E-07 |
| PDK4 | -3.49 | 2.25E-05 |
| ARHGAP36 | -3.47 | 2.13E-04 |
| SERHL2 | -3.47 | 9.83E-03 |
| ITM2A | -3.47 | 5.74E-08 |
| MME | -3.45 | 7.93E-11 |
| ADAMTS15 | -3.45 | 8.74E-03 |
| ALDH1A1 | -3.44 | 2.98E-05 |
| TRIM29 | -3.43 | 2.04E-03 |
| ITIH5 | -3.42 | 1.70E-06 |
| TMTC1 | -3.41 | 2.38E-05 |
| LIFR | -3.38 | 5.17E-06 |
| HAS3 | -3.38 | 8.65E-15 |
| NTN4 | -3.37 | 1.37E-03 |
| EGR3 | -3.36 | 6.18E-05 |
| PLAT | -3.36 | 4.07E-03 |
| COL4A6 | -3.35 | 1.46E-05 |
| ADIRF | -3.35 | 1.02E-02 |
| AQP1 | -3.34 | 2.63E-05 |
| AMIGO2 | -3.33 | 1.73E-04 |
| TSHZ2 | -3.33 | 1.41E-11 |
| FMO5 | -3.32 | 1.22E-05 |
| COL14A1 | -3.32 | 2.25E-06 |
| AKR1C1 | -3.32 | 4.44E-04 |
| GHR | -3.31 | 1.97E-04 |
| MIR100HG | -3.31 | 3.30E-05 |
| AFF3 | -3.3 | 3.67E-02 |
| CCL21 | -3.28 | 3.27E-07 |
| UGT2B28 | -3.28 | 7.68E-03 |
| TCEAL7 | -3.25 | 1.87E-11 |
| SMOC2 | -3.25 | 2.96E-04 |
| DIRAS3 | -3.25 | 1.00E-11 |
| CYP26A1 | -3.25 | 6.87E-09 |
| TMEM178A | -3.24 | 8.97E-08 |
| AKR1C2 | -3.23 | 1.68E-04 |
| PGM5 | -3.23 | 1.36E-13 |
| BOC | -3.22 | 5.37E-04 |
| APCDD1 | -3.22 | 5.23E-06 |
| SCN4B | -3.21 | 3.37E-13 |
| CX3CL1 | -3.2 | 1.10E-05 |
| ANO1 | -3.2 | 2.01E-04 |
| WLS | -3.2 | 7.16E-06 |
| SRPX | -3.19 | 1.28E-07 |
| SLC7A2 | -3.18 | 4.02E-02 |
| FOS | -3.18 | 3.56E-04 |
| RNF39 | -3.17 | 4.52E-09 |
| LAMB3 | -3.16 | 1.76E-05 |
| CD36 | -3.16 | 5.03E-04 |
| HSPB6 | -3.16 | 1.81E-06 |
| ACTG2 | -3.15 | 1.97E-02 |
| OSR1 | -3.15 | 5.90E-05 |
| MAPT | -3.14 | 2.02E-02 |
| GPX3 | -3.14 | 1.40E-05 |
| EGR2 | -3.12 | 2.28E-08 |
| PENK | -3.11 | 6.12E-09 |
| SPRY2 | -3.09 | 4.52E-09 |
| EGR1 | -3.09 | 5.98E-05 |
| TESC | -3.08 | 4.94E-08 |
| CMYA5 | -3.08 | 1.89E-05 |
| PDZK1 | -3.08 | 1.25E-02 |
| JCHAIN | -3.07 | 5.71E-03 |
| C16orf89 | -3.06 | 5.75E-05 |
| FBLN1 | -3.06 | 7.94E-06 |
| PLIN4 | -3.02 | 1.15E-08 |
| OLFM4 | -3 | 2.12E-03 |
| GPD1 | -3 | 5.22E-07 |
| CLU | -2.99 | 9.55E-04 |
| CXCL2 | -2.99 | 1.58E-04 |
| PDGFRA | -2.98 | 5.05E-07 |
| RAI2 | -2.97 | 4.74E-05 |
| ANPEP | -2.96 | 8.21E-08 |
| CRYAB | -2.96 | 2.60E-02 |
| RBMS3 | -2.95 | 7.18E-07 |
| AKR1C3 | -2.94 | 1.62E-03 |
| ABI3BP | -2.93 | 2.59E-03 |
| CX3CR1 | -2.93 | 3.55E-04 |
| PROS1 | -2.93 | 4.94E-08 |
| CCL28 | -2.92 | 2.17E-03 |
| ISM1 | -2.9 | 3.77E-05 |
| AKAP12 | -2.9 | 3.18E-05 |
| PLAC9 | -2.89 | 1.95E-09 |
| TFPI | -2.89 | 1.02E-07 |
| ID4 | -2.89 | 1.97E-02 |
| AREG | -2.88 | 2.39E-02 |
| ADAMTS5 | -2.88 | 2.38E-05 |
| DNALI1 | -2.88 | 2.98E-02 |
| NOSTRIN | -2.87 | 1.76E-03 |
| INHBB | -2.87 | 3.39E-03 |
| LHFPL6 | -2.87 | 6.97E-07 |
| FBLN5 | -2.87 | 1.34E-05 |
| MYLK | -2.87 | 2.59E-04 |
| SYNPO2 | -2.86 | 1.23E-02 |
| LYPD6 | -2.85 | 1.88E-05 |
| STEAP4 | -2.84 | 8.13E-03 |
| TNS4 | -2.84 | 4.49E-06 |
| NR4A1 | -2.83 | 1.65E-04 |
| SH3BGRL2 | -2.82 | 8.27E-04 |
| BTNL9 | -2.82 | 1.48E-09 |
| PPP1R14A | -2.81 | 1.85E-03 |
| PON3 | -2.81 | 1.97E-02 |
| PDGFA | -2.79 | 7.76E-06 |
| DPT | -2.79 | 2.01E-03 |
| ADRA2A | -2.79 | 1.77E-04 |
| CGNL1 | -2.77 | 5.37E-04 |
| SVEP1 | -2.76 | 1.94E-10 |
| TNS1 | -2.75 | 2.02E-05 |
| SPARCL1 | -2.75 | 1.46E-05 |
| SEMA3G | -2.75 | 3.25E-09 |
| CAV1 | -2.74 | 1.26E-05 |
| FRZB | -2.73 | 1.66E-02 |
| FCGBP | -2.73 | 3.06E-04 |
| EFEMP1 | -2.72 | 6.61E-04 |
| PTPRZ1 | -2.72 | 1.93E-04 |
| ENPP2 | -2.71 | 3.34E-04 |
| SLIT2 | -2.71 | 4.95E-05 |
| FMO1 | -2.7 | 1.10E-04 |
| ALDH3B2 | -2.7 | 2.23E-02 |
| RASSF6 | -2.69 | 1.60E-04 |
| PDE2A | -2.69 | 1.70E-10 |
| PTHLH | -2.69 | 6.43E-07 |
| CBX7 | -2.68 | 2.81E-07 |
| IL33 | -2.68 | 7.45E-09 |
| THSD4 | -2.67 | 4.55E-02 |
| TGFBR2 | -2.67 | 4.49E-07 |
| MAOB | -2.67 | 2.62E-02 |
| IRX1 | -2.66 | 1.30E-02 |
| EBF1 | -2.66 | 1.67E-08 |
| AOC3 | -2.65 | 5.46E-06 |
| LTBP2 | -2.64 | 1.21E-04 |
| GSTM5 | -2.64 | 1.22E-05 |
| ECM2 | -2.64 | 2.60E-05 |
| HOXA3 | -2.63 | 2.73E-04 |
| COX7A1 | -2.62 | 1.84E-04 |
| DCDC2 | -2.62 | 1.26E-04 |
| STON2 | -2.62 | 8.58E-03 |
| SOCS2 | -2.62 | 2.17E-03 |
| CFI | -2.62 | 1.99E-04 |
| NDRG2 | -2.61 | 3.00E-04 |
| PDGFRL | -2.61 | 8.06E-05 |
| IRX2 | -2.61 | 2.32E-02 |
| GSTM2 | -2.6 | 2.64E-03 |
| TCN1 | -2.59 | 3.20E-02 |
| DCN | -2.58 | 6.77E-04 |
| ANK2 | -2.58 | 1.03E-04 |
| IGSF10 | -2.58 | 1.07E-11 |
| TSPAN8 | -2.58 | 5.26E-03 |
| PCDH18 | -2.58 | 2.45E-06 |
| MEIS2 | -2.58 | 5.34E-04 |
| LRRN4CL | -2.57 | 6.22E-12 |
| BHLHE41 | -2.57 | 5.26E-04 |
| OLFML3 | -2.57 | 1.34E-04 |
| SEMA3C | -2.56 | 7.30E-03 |
| PTGS2 | -2.56 | 2.00E-03 |
| CACHD1 | -2.56 | 1.42E-06 |
| LRRC17 | -2.55 | 1.29E-02 |
| CAVIN1 | -2.55 | 6.31E-07 |
| PDLIM3 | -2.55 | 1.26E-02 |
| BBOX1 | -2.55 | 4.12E-02 |
| PTGDS | -2.55 | 5.68E-05 |
| LDB2 | -2.54 | 6.27E-07 |
| INMT | -2.54 | 1.87E-13 |
| ZNF204P | -2.53 | 2.92E-05 |
| CHL1 | -2.52 | 1.28E-08 |
| GPRASP1 | -2.52 | 3.95E-06 |
| HLF | -2.51 | 5.37E-07 |
| GSN | -2.51 | 1.11E-10 |
| GRAMD2B | -2.51 | 5.11E-08 |
| CFH | -2.5 | 4.84E-04 |
| SETBP1 | -2.5 | 3.35E-03 |
| ATF3 | -2.48 | 6.60E-04 |
| AMT | -2.48 | 2.89E-04 |
| G0S2 | -2.48 | 1.21E-03 |
| JAM2 | -2.48 | 2.28E-04 |
| PALMD | -2.47 | 1.09E-04 |
| CES1 | -2.47 | 4.93E-08 |
| CCND2 | -2.47 | 1.03E-03 |
| NES | -2.47 | 9.55E-04 |
| KLF4 | -2.46 | 7.76E-06 |
| TSPAN7 | -2.46 | 1.73E-07 |
| DLC1 | -2.46 | 8.42E-05 |
| FMOD | -2.46 | 2.43E-03 |
| FTO | -2.45 | 4.68E-09 |
| SNCG | -2.45 | 9.16E-05 |
| OLFML2A | -2.45 | 1.17E-04 |
| ZCCHC24 | -2.45 | 8.06E-05 |
| GAS1 | -2.45 | 8.30E-06 |
| PLSCR4 | -2.45 | 2.88E-07 |
| LAMB2 | -2.45 | 1.94E-03 |
| SLC7A3 | -2.44 | 2.46E-17 |
| P3H2 | -2.44 | 1.96E-06 |
| ABLIM3 | -2.44 | 3.14E-03 |
| COL4A5 | -2.44 | 2.14E-02 |
| GEM | -2.43 | 6.49E-05 |
| DKK3 | -2.43 | 6.69E-05 |
| EGFR | -2.42 | 2.92E-03 |
| CDO1 | -2.42 | 1.36E-06 |
| FGF2 | -2.4 | 5.28E-06 |
| FHOD3 | -2.4 | 6.32E-04 |
| GPIHBP1 | -2.4 | 9.17E-14 |
| EHD2 | -2.4 | 2.00E-04 |
| CREB5 | -2.39 | 1.60E-12 |
| BTBD11 | -2.39 | 8.03E-05 |
| ABCA6 | -2.39 | 1.11E-10 |
| RNASE4 | -2.38 | 4.73E-03 |
| ENTPD5 | -2.38 | 5.89E-04 |
| KCNE1 | -2.38 | 1.48E-16 |
| STEAP2 | -2.38 | 1.99E-03 |
| NR3C2 | -2.37 | 9.16E-06 |
| PPP1R12B | -2.37 | 1.07E-04 |
| NGFR | -2.37 | 2.80E-05 |
| IL17RD | -2.37 | 1.69E-03 |
| VIPR1 | -2.37 | 1.57E-03 |
| THRB | -2.36 | 1.32E-03 |
| PHYHD1 | -2.36 | 7.89E-06 |
| TAGLN | -2.36 | 1.32E-03 |
| TAC1 | -2.34 | 4.13E-07 |
| PIK3C2G | -2.34 | 2.47E-05 |
| GRP | -2.34 | 6.65E-03 |
| ITGA7 | -2.34 | 6.44E-05 |
| CPA3 | -2.33 | 2.98E-02 |
| DMD | -2.32 | 3.07E-02 |
| IFFO2 | -2.32 | 1.07E-07 |
| SLC27A6 | -2.32 | 5.59E-03 |
| PDE5A | -2.31 | 1.47E-04 |
| EGFLAM | -2.31 | 1.67E-07 |
| MEOX1 | -2.3 | 3.42E-05 |
| IQGAP2 | -2.3 | 5.37E-04 |
| PLPP3 | -2.3 | 1.06E-07 |
| ECHDC2 | -2.3 | 6.03E-08 |
| ACSM3 | -2.3 | 1.75E-05 |
| NDN | -2.29 | 1.59E-03 |
| PDLIM4 | -2.29 | 5.11E-05 |
| GLT8D2 | -2.29 | 2.63E-04 |
| FAM126A | -2.28 | 9.04E-05 |
| AZGP1 | -2.27 | 3.99E-02 |
| JAM3 | -2.26 | 2.88E-06 |
| GSTM1 | -2.26 | 2.04E-03 |
| CSRNP1 | -2.25 | 8.49E-05 |
| LAMA2 | -2.25 | 2.65E-05 |
| ITGA9 | -2.25 | 7.48E-03 |
| NAV3 | -2.25 | 4.07E-04 |
| REEP6 | -2.24 | 4.40E-03 |
| SLC28A3 | -2.24 | 2.47E-03 |
| UNC5C | -2.24 | 9.99E-07 |
| VWF | -2.23 | 2.84E-04 |
| MEIS1 | -2.23 | 2.89E-04 |
| METTL7A | -2.23 | 6.47E-04 |
| CLDN5 | -2.23 | 2.34E-11 |
| MYL9 | -2.23 | 2.39E-03 |
| ACTA2 | -2.23 | 8.55E-04 |
| GOLGA8A | -2.23 | 1.11E-03 |
| OMD | -2.22 | 9.08E-03 |
| LPCAT2 | -2.22 | 2.56E-06 |
| IL11RA | -2.22 | 8.58E-05 |
| PLCB1 | -2.22 | 1.67E-02 |
| CTSG | -2.22 | 8.56E-07 |
| CIRBP | -2.22 | 4.49E-06 |
| IGFBP4 | -2.21 | 1.54E-03 |
| GRAMD2A | -2.21 | 4.08E-03 |
| ZFP36 | -2.21 | 5.56E-05 |
| NCALD | -2.21 | 1.83E-02 |
| CNRIP1 | -2.21 | 1.61E-04 |
| NFIA | -2.2 | 3.04E-02 |
| CAVIN2 | -2.2 | 2.15E-11 |
| MTUS1 | -2.19 | 3.34E-04 |
| ANGPTL2 | -2.18 | 2.24E-04 |
| C7 | -2.18 | 7.89E-06 |
| ADGRA2 | -2.18 | 1.12E-07 |
| MSRB3 | -2.17 | 9.16E-03 |
| MRC1 | -2.17 | 1.93E-03 |
| LEP | -2.17 | 2.04E-03 |
| SOBP | -2.17 | 2.79E-03 |
| PPP1R1A | -2.17 | 1.67E-02 |
| MEOX2 | -2.16 | 6.13E-11 |
| LAYN | -2.15 | 8.03E-04 |
| CRACR2B | -2.15 | 5.91E-03 |
| CSRP1 | -2.15 | 1.58E-05 |
| ITPR1 | -2.15 | 2.83E-04 |
| EMCN | -2.15 | 4.43E-04 |
| KIF13B | -2.14 | 5.76E-03 |
| BRINP1 | -2.14 | 1.71E-05 |
| CYBRD1 | -2.14 | 2.04E-03 |
| C1orf115 | -2.12 | 8.07E-03 |
| TNS2 | -2.12 | 5.70E-06 |
| SPATA18 | -2.12 | 1.42E-03 |
| TIMP4 | -2.12 | 7.36E-04 |
| LINC01279 | -2.12 | 2.71E-02 |
| DUSP6 | -2.12 | 3.22E-03 |
| PAPLN | -2.12 | 3.38E-04 |
| PODN | -2.11 | 1.13E-04 |
| ACSM1 | -2.11 | 6.94E-05 |
| PLEKHH2 | -2.11 | 1.22E-05 |
| SYBU | -2.11 | 2.98E-02 |
| MST1 | -2.11 | 6.17E-03 |
| AKR1B10 | -2.11 | 3.89E-02 |
| ZNF662 | -2.11 | 5.26E-03 |
| ADD3 | -2.11 | 8.62E-05 |
| RCAN1 | -2.11 | 1.08E-02 |
| CDKN1C | -2.1 | 8.91E-04 |
| AHNAK | -2.09 | 8.00E-04 |
| SPRY1 | -2.09 | 2.15E-07 |
| FREM1 | -2.09 | 1.99E-10 |
| CCN1 | -2.09 | 4.17E-04 |
| GGTA1P | -2.09 | 7.06E-06 |
| RSPO3 | -2.09 | 1.10E-03 |
| C8orf88 | -2.09 | 3.12E-08 |
| EPB41L4A-AS1 | -2.08 | 3.66E-08 |
| MAST4 | -2.08 | 2.26E-02 |
| LPL | -2.08 | 4.54E-02 |
| DUSP1 | -2.08 | 3.99E-04 |
| KIRREL1 | -2.08 | 1.64E-03 |
| ID1 | -2.07 | 1.50E-02 |
| RHOU | -2.07 | 1.76E-03 |
| MOB3B | -2.07 | 8.00E-03 |
| CDC42EP5 | -2.06 | 4.12E-02 |
| FILIP1L | -2.06 | 2.38E-03 |
| CYYR1 | -2.06 | 2.65E-03 |
| PTPRG | -2.06 | 1.44E-03 |
| KCNMA1 | -2.06 | 5.41E-03 |
| CRIM1-DT | -2.05 | 1.56E-02 |
| NAV2 | -2.05 | 1.56E-03 |
| F13A1 | -2.05 | 7.59E-03 |
| RBPMS | -2.05 | 1.02E-02 |
| LOC101926959 | -2.04 | 8.13E-03 |
| ANXA1 | -2.04 | 6.97E-04 |
| ZBTB20 | -2.04 | 1.71E-03 |
| PTGIS | -2.03 | 1.55E-02 |
| SH3D19 | -2.03 | 2.54E-08 |
| ERG | -2.03 | 1.70E-05 |
| CD34 | -2.03 | 9.84E-09 |
| TXNIP | -2.03 | 5.98E-05 |
| CD200 | -2.02 | 1.79E-03 |
| EPB41L4A | -2.02 | 4.71E-02 |
| CD248 | -2.01 | 1.89E-05 |
| PER2 | -2.01 | 2.78E-04 |
| CCDC18-AS1 | -2.01 | 7.32E-04 |
| NIBAN1 | -2.01 | 1.62E-02 |
| ADAMTS1 | -2.01 | 7.10E-03 |
| SERPING1 | -2.01 | 1.81E-06 |
| FAM107A | -2 | 4.06E-02 |
| MAML2 | -2 | 1.69E-02 |
| AFAP1L2 | -2 | 2.61E-05 |
| CPE | -2 | 1.97E-02 |
| CCDC3 | -2 | 1.89E-03 |
| PRICKLE2 | -2 | 1.84E-02 |
| SHE | -1.99 | 4.18E-06 |
| OLFML1 | -1.99 | 8.92E-06 |
| FAT2 | -1.99 | 2.24E-06 |
| DLL1 | -1.99 | 6.91E-04 |
| C1orf226 | -1.99 | 4.74E-02 |
| XIST | -1.99 | 4.21E-02 |
| ADH1C | -1.98 | 1.68E-08 |
| FBXO2 | -1.98 | 2.27E-02 |
| LRIG1 | -1.97 | 3.42E-03 |
| STAC2 | -1.97 | 1.43E-05 |
| SRSF5 | -1.97 | 4.17E-05 |
| ADHFE1 | -1.96 | 2.36E-03 |
| PPL | -1.96 | 1.37E-03 |
| NDNF | -1.96 | 4.19E-06 |
| CDH5 | -1.95 | 1.03E-03 |
| ETV1 | -1.95 | 1.37E-02 |
| ARHGAP20 | -1.95 | 5.94E-07 |
| IL17B | -1.95 | 2.86E-02 |
| TPM2 | -1.95 | 1.53E-03 |
| PCNX1 | -1.95 | 3.60E-05 |
| GLI3 | -1.95 | 4.53E-02 |
| ABLIM1 | -1.94 | 1.66E-03 |
| ARID5A | -1.94 | 5.60E-06 |
| CIDEC | -1.94 | 1.24E-05 |
| ARHGAP19 | -1.93 | 1.15E-08 |
| FHL2 | -1.93 | 2.25E-02 |
| CPXM1 | -1.93 | 1.78E-04 |
| KLK6 | -1.92 | 3.57E-02 |
| TM4SF18 | -1.92 | 3.20E-02 |
| EPAS1 | -1.92 | 6.54E-03 |
| ZBTB7C | -1.92 | 1.02E-02 |
| ABCA5 | -1.92 | 1.32E-04 |
| LIMCH1 | -1.91 | 3.01E-02 |
| COL16A1 | -1.91 | 3.27E-04 |
| FZD7 | -1.91 | 2.88E-02 |
| RBMS2 | -1.91 | 2.31E-06 |
| F3 | -1.91 | 2.46E-04 |
| STXBP1 | -1.91 | 3.05E-02 |
| FRMD6 | -1.9 | 8.46E-04 |
| TRIM68 | -1.9 | 2.83E-03 |
| JADE2 | -1.9 | 6.99E-03 |
| ZNF423 | -1.9 | 2.11E-04 |
| MMRN2 | -1.89 | 1.40E-07 |
| HIC1 | -1.89 | 5.94E-07 |
| ARRDC4 | -1.88 | 3.79E-03 |
| RPS6KA5 | -1.88 | 1.61E-02 |
| RERE | -1.88 | 2.69E-03 |
| EREG | -1.88 | 2.05E-02 |
| GAS6 | -1.87 | 3.14E-07 |
| ARID5B | -1.87 | 1.78E-04 |
| RELN | -1.87 | 2.69E-07 |
| TRMT9B | -1.87 | 8.93E-09 |
| PDLIM1 | -1.87 | 1.18E-05 |
| PELI2 | -1.87 | 2.19E-04 |
| PLPP1 | -1.87 | 6.70E-05 |
| ZNF608 | -1.86 | 7.64E-03 |
| ALKAL2 | -1.86 | 4.53E-02 |
| SCN7A | -1.86 | 1.75E-05 |
| PGM5-AS1 | -1.86 | 8.17E-04 |
| FRY | -1.86 | 2.05E-02 |
| MTURN | -1.86 | 9.14E-03 |
| MGP | -1.85 | 8.47E-03 |
| IRX4 | -1.85 | 2.04E-02 |
| CCN5 | -1.85 | 3.78E-02 |
| PTGER4 | -1.85 | 2.88E-04 |
| TMEM98 | -1.85 | 2.26E-02 |
| ALDH1A3 | -1.85 | 7.61E-03 |
| EPHX2 | -1.85 | 6.90E-04 |
| HCAR3 | -1.85 | 1.85E-02 |
| MYADM | -1.84 | 4.53E-04 |
| DEFB1 | -1.84 | 4.19E-02 |
| FYB2 | -1.84 | 7.82E-03 |
| MAFF | -1.83 | 4.33E-03 |
| MIRLET7BHG | -1.83 | 3.34E-04 |
| IL20RA | -1.83 | 5.71E-04 |
| TWIST2 | -1.83 | 6.47E-04 |
| SEC14L2 | -1.83 | 5.03E-03 |
| GNG11 | -1.83 | 1.95E-04 |
| FOXO1 | -1.83 | 9.81E-07 |
| CA4 | -1.82 | 8.78E-07 |
| ANKRD35 | -1.82 | 3.70E-04 |
| ME3 | -1.82 | 1.04E-02 |
| IRS1 | -1.82 | 2.74E-02 |
| AMOTL2 | -1.82 | 2.01E-03 |
| SLIT3 | -1.81 | 1.39E-11 |
| MXRA8 | -1.81 | 4.19E-03 |
| MMP2 | -1.81 | 6.72E-03 |
| MAGI2-AS3 | -1.81 | 2.65E-05 |
| NBEA | -1.81 | 2.37E-02 |
| SASH1 | -1.81 | 5.78E-04 |
| ACADVL | -1.8 | 6.15E-06 |
| KANK2 | -1.8 | 7.30E-04 |
| BICC1 | -1.8 | 1.65E-03 |
| TSHZ1 | -1.8 | 3.15E-03 |
| RND3 | -1.8 | 9.22E-04 |
| SC5D | -1.8 | 4.60E-04 |
| PTPRE | -1.8 | 1.02E-04 |
| LGR6 | -1.8 | 2.21E-02 |
| RGS2 | -1.79 | 1.42E-02 |
| MICU3 | -1.79 | 7.78E-10 |
| SMIM10L2A | -1.79 | 8.54E-09 |
| ECI2 | -1.79 | 9.89E-03 |
| SLC25A27 | -1.79 | 1.05E-03 |
| NISCH | -1.79 | 5.94E-05 |
| CFAP70 | -1.78 | 2.48E-02 |
| UTRN | -1.78 | 1.02E-04 |
| PTPN21 | -1.78 | 1.07E-05 |
| CLMP | -1.78 | 8.11E-04 |
| ARHGEF37 | -1.78 | 7.62E-04 |
| STAT5B | -1.77 | 4.75E-05 |
| RERGL | -1.77 | 5.92E-07 |
| CAPN11 | -1.77 | 8.15E-16 |
| ARL4A | -1.77 | 1.80E-04 |
| TACC1 | -1.76 | 1.35E-03 |
| SELENOP | -1.76 | 6.60E-03 |
| FAM13C | -1.76 | 1.69E-06 |
| MAOA | -1.76 | 2.00E-03 |
| SIK3 | -1.76 | 1.09E-03 |
| GPD1L | -1.75 | 3.14E-02 |
| THBD | -1.75 | 1.87E-03 |
| A2M | -1.74 | 1.55E-02 |
| PKD2 | -1.74 | 4.35E-06 |
| TGFB1I1 | -1.74 | 9.37E-03 |
| TENM2 | -1.74 | 4.37E-03 |
| ADAMTS9-AS2 | -1.74 | 2.89E-09 |
| ALPL | -1.74 | 9.19E-06 |
| N4BP2L1 | -1.74 | 5.07E-03 |
| LINC01697 | -1.73 | 1.04E-07 |
| CLIP3 | -1.73 | 2.41E-04 |
| MCCC2 | -1.73 | 5.41E-03 |
| TRIL | -1.73 | 2.43E-02 |
| ANGPTL1 | -1.73 | 5.11E-08 |
| CIDEA | -1.72 | 2.86E-05 |
| EGF | -1.71 | 4.55E-02 |
| EFNB3 | -1.71 | 1.19E-02 |
| SSPN | -1.71 | 2.40E-03 |
| PLAGL1 | -1.71 | 4.55E-02 |
| INPP1 | -1.71 | 3.82E-04 |
| LIMA1 | -1.7 | 6.17E-03 |
| ANG | -1.7 | 1.06E-02 |
| IQCN | -1.7 | 1.41E-06 |
| ITGA10 | -1.7 | 1.02E-02 |
| ANKRD29 | -1.7 | 1.86E-07 |
| SLC27A1 | -1.7 | 3.72E-03 |
| HYMAI | -1.7 | 4.05E-06 |
| FCGRT | -1.7 | 6.41E-04 |
| NATD1 | -1.69 | 3.18E-04 |
| FZD4 | -1.69 | 3.62E-03 |
| C3 | -1.69 | 4.72E-03 |
| DIXDC1 | -1.69 | 1.60E-03 |
| GOLGA8N | -1.69 | 1.92E-03 |
| CACNA2D1 | -1.69 | 1.37E-02 |
| GASK1B | -1.69 | 4.21E-02 |
| DST | -1.69 | 1.35E-03 |
| ADRB2 | -1.69 | 1.02E-03 |
| RASIP1 | -1.68 | 9.08E-03 |
| LRP1 | -1.68 | 5.04E-06 |
| ACCS | -1.68 | 2.51E-05 |
| ZNF334 | -1.68 | 9.66E-06 |
| KLF2 | -1.68 | 4.62E-03 |
| CMTM7 | -1.68 | 2.80E-02 |
| GNAL | -1.68 | 1.56E-02 |
| AMOTL1 | -1.67 | 1.88E-02 |
| SRARP | -1.67 | 3.22E-02 |
| APLP2 | -1.67 | 2.47E-04 |
| LOC100506990 | -1.66 | 2.30E-03 |
| CAV2 | -1.66 | 8.30E-03 |
| PRKD1 | -1.66 | 2.61E-03 |
| DAB2IP | -1.66 | 1.62E-05 |
| MEG3 | -1.66 | 2.19E-02 |
| GAS7 | -1.66 | 7.42E-03 |
| NSG1 | -1.66 | 2.79E-08 |
| ABCA9 | -1.66 | 6.85E-09 |
| JUN | -1.66 | 4.62E-05 |
| TMEM255A | -1.66 | 2.84E-06 |
| DENND2B | -1.65 | 2.49E-03 |
| MAF | -1.65 | 1.67E-04 |
| MYOF | -1.65 | 4.96E-03 |
| TIMP3 | -1.65 | 4.60E-02 |
| NRN1 | -1.64 | 9.67E-03 |
| ITIH2 | -1.64 | 1.36E-02 |
| PID1 | -1.64 | 8.81E-03 |
| FERMT2 | -1.63 | 2.24E-02 |
| RNASE1 | -1.63 | 2.53E-02 |
| NR4A2 | -1.62 | 3.71E-02 |
| SLC25A37 | -1.62 | 1.01E-02 |
| USP53 | -1.62 | 6.96E-04 |
| EVA1C | -1.62 | 2.83E-02 |
| HSD17B11 | -1.62 | 2.32E-02 |
| FCER1A | -1.62 | 8.08E-03 |
| MAN2C1 | -1.62 | 1.89E-02 |
| PIK3R1 | -1.62 | 1.15E-02 |
| NEO1 | -1.62 | 1.06E-02 |
| HEPACAM2 | -1.62 | 2.74E-02 |
| TLE2 | -1.62 | 1.16E-02 |
| EGFL7 | -1.61 | 5.71E-03 |
| LPAR6 | -1.61 | 3.60E-03 |
| CYGB | -1.61 | 6.73E-05 |
| ABHD14B | -1.61 | 1.37E-03 |
| ANTXR2 | -1.61 | 2.08E-04 |
| PCCA | -1.61 | 1.46E-04 |
| NEDD9 | -1.6 | 9.23E-03 |
| PGC | -1.6 | 1.83E-02 |
| TCF7L2 | -1.6 | 2.40E-03 |
| DNMBP | -1.6 | 3.58E-02 |
| SCGB1A1 | -1.6 | 6.81E-03 |
| HOXA7 | -1.59 | 9.37E-05 |
| KCNIP2 | -1.59 | 4.14E-04 |
| AUTS2 | -1.59 | 1.58E-02 |
| ATG2B | -1.59 | 1.44E-03 |
| NAP1L2 | -1.59 | 2.80E-09 |
| FAM13A | -1.59 | 2.19E-03 |
| PNLIPRP3 | -1.59 | 7.33E-03 |
| ABCG2 | -1.58 | 4.03E-07 |
| ZNF395 | -1.58 | 6.96E-04 |
| FTX | -1.58 | 2.53E-02 |
| C1R | -1.58 | 2.56E-03 |
| GGTLC1 | -1.58 | 9.43E-03 |
| COLEC12 | -1.57 | 8.19E-03 |
| CSN1S1 | -1.57 | 4.19E-02 |
| TTC36 | -1.57 | 1.75E-02 |
| EZH1 | -1.57 | 3.80E-05 |
| ZFP36L2 | -1.57 | 5.52E-04 |
| KLHL21 | -1.57 | 2.64E-03 |
| TNMD | -1.57 | 1.41E-03 |
| GRAMD1C | -1.56 | 2.02E-02 |
| TSPAN5 | -1.56 | 3.25E-02 |
| STAB1 | -1.56 | 9.96E-04 |
| CRIM1 | -1.56 | 8.07E-03 |
| PRELP | -1.56 | 2.81E-02 |
| PLXDC1 | -1.56 | 2.42E-02 |
| CTF1 | -1.56 | 1.78E-04 |
| TMEM100 | -1.56 | 2.32E-02 |
| LPAR1 | -1.56 | 2.50E-02 |
| ZBTB4 | -1.55 | 6.63E-06 |
| ITGA3 | -1.55 | 8.30E-03 |
| MSRA | -1.55 | 5.88E-03 |
| LAMC3 | -1.54 | 1.99E-10 |
| RBM5 | -1.54 | 3.15E-04 |
| CALCOCO1 | -1.54 | 2.80E-06 |
| PRKCH | -1.54 | 2.99E-03 |
| ZSCAN18 | -1.54 | 7.78E-03 |
| TNNI2 | -1.52 | 3.45E-02 |
| IRS2 | -1.52 | 3.58E-03 |
| SESN1 | -1.52 | 1.20E-03 |
| JAG2 | -1.52 | 6.11E-04 |
| MMRN1 | -1.51 | 8.97E-08 |
| YAP1 | -1.51 | 2.59E-03 |
| LTBP3 | -1.51 | 1.26E-02 |
| IGIP | -1.51 | 4.08E-03 |
| INTU | -1.51 | 5.39E-03 |
| MRGPRF | -1.51 | 5.98E-06 |
| PCOLCE | -1.51 | 5.26E-03 |
| MAP2K6 | -1.51 | 2.76E-02 |
| PCK1 | -1.5 | 4.63E-04 |
| RARRES2 | -1.5 | 1.01E-02 |
| COL15A1 | -1.5 | 3.17E-02 |
| PLTP | -1.5 | 1.16E-03 |
| ELOVL5 | -1.5 | 6.23E-03 |
| NPY2R | -1.5 | 1.08E-03 |
| LENG8 | -1.5 | 1.50E-02 |
| SPATA6 | -1.5 | 2.88E-03 |
| GNMT | -1.5 | 1.53E-04 |
| RASL10A | -1.5 | 1.03E-02 |
| CAPN3 | -1.49 | 4.67E-03 |
| BDH2 | -1.49 | 3.71E-03 |
| PPARG | -1.49 | 6.43E-03 |
| PRIMA1 | -1.49 | 9.04E-03 |
| MYOM2 | -1.49 | 9.55E-04 |
| NID1 | -1.49 | 4.72E-02 |
| DDB2 | -1.48 | 9.02E-04 |
| FAM160B2 | -1.48 | 8.54E-03 |
| HTRA1 | -1.47 | 3.82E-02 |
| MT1X | -1.47 | 3.58E-03 |
| DPYSL2 | -1.47 | 4.93E-03 |
| GNG2 | -1.47 | 2.78E-02 |
| CEP126 | -1.47 | 4.03E-02 |
| IL6ST | -1.47 | 3.05E-02 |
| CRYBG3 | -1.46 | 4.23E-04 |
| SYNE1 | -1.46 | 1.48E-02 |
| PDGFC | -1.46 | 1.56E-02 |
| LAMC1 | -1.46 | 4.77E-03 |
| VSIR | -1.46 | 2.05E-04 |
| KIAA1217 | -1.46 | 4.75E-03 |
| TCEAL2 | -1.46 | 3.82E-02 |
| PNPLA7 | -1.46 | 5.15E-03 |
| FOLR2 | -1.46 | 1.30E-03 |
| AOX1 | -1.46 | 1.47E-05 |
| ITGB4 | -1.46 | 1.25E-02 |
| DCAKD | -1.46 | 1.54E-02 |
| STARD13 | -1.45 | 1.16E-02 |
| PDZRN3 | -1.45 | 1.06E-02 |
| ARHGAP6 | -1.45 | 3.12E-03 |
| FAM13B | -1.45 | 8.97E-03 |
| CYB5D2 | -1.45 | 7.50E-03 |
| SPON1 | -1.44 | 3.14E-02 |
| PMP22 | -1.44 | 2.73E-02 |
| PTPRT | -1.44 | 1.17E-02 |
| VPS51 | -1.44 | 3.82E-03 |
| PAN2 | -1.44 | 4.12E-02 |
| TPCN1 | -1.44 | 1.03E-02 |
| CA11 | -1.44 | 1.72E-02 |
| TLE4 | -1.43 | 2.79E-02 |
| LDHD | -1.43 | 2.22E-05 |
| CTSO | -1.43 | 3.06E-02 |
| LINC02381 | -1.43 | 1.36E-02 |
| FZD1 | -1.43 | 3.00E-02 |
| RORA | -1.43 | 1.40E-02 |
| AKR1D1 | -1.43 | 3.58E-03 |
| ARHGAP23 | -1.43 | 4.18E-03 |
| PLK2 | -1.42 | 1.55E-02 |
| TIE1 | -1.42 | 1.07E-02 |
| ROR1 | -1.42 | 7.31E-03 |
| EFEMP2 | -1.42 | 3.91E-03 |
| TTC28 | -1.42 | 5.60E-04 |
| TNN | -1.42 | 1.44E-04 |
| CARD10 | -1.42 | 3.06E-02 |
| KCNJ2 | -1.42 | 8.19E-03 |
| RAMP2 | -1.41 | 1.44E-02 |
| PTPRB | -1.41 | 9.48E-04 |
| LINC00472 | -1.41 | 2.39E-02 |
| GNG7 | -1.41 | 1.66E-03 |
| MXI1 | -1.41 | 2.58E-03 |
| WDR19 | -1.41 | 4.12E-02 |
| KLHL3 | -1.41 | 3.86E-02 |
| CTSF | -1.41 | 1.53E-02 |
| ADGRL2 | -1.41 | 2.75E-02 |
| ACSF2 | -1.41 | 4.74E-02 |
| LAMA4 | -1.4 | 3.59E-02 |
| TBRG1 | -1.4 | 6.52E-03 |
| PLEKHA4 | -1.4 | 2.55E-03 |
| GNG12 | -1.4 | 2.16E-02 |
| ATP7A | -1.4 | 8.13E-03 |
| ARHGEF40 | -1.4 | 4.30E-03 |
| ADGRF5 | -1.4 | 5.96E-03 |
| SPTBN1 | -1.4 | 9.16E-05 |
| SMAD9 | -1.4 | 3.61E-02 |
| GULP1 | -1.4 | 2.20E-02 |
| ANKMY2 | -1.39 | 5.73E-04 |
| EDNRB | -1.39 | 2.02E-03 |
| SAMD4A | -1.39 | 1.85E-02 |
| ROBO4 | -1.39 | 6.18E-04 |
| SRSF6 | -1.39 | 5.86E-03 |
| NFAT5 | -1.39 | 2.01E-04 |
| FAM43A | -1.39 | 6.78E-03 |
| TRO | -1.39 | 3.91E-02 |
| BEND5 | -1.39 | 1.06E-03 |
| SLC46A3 | -1.39 | 3.24E-02 |
| ZFHX4 | -1.39 | 6.08E-03 |
| IER2 | -1.39 | 1.60E-03 |
| HSPA12B | -1.39 | 9.54E-04 |
| MYO15B | -1.38 | 4.13E-03 |
| DOCK1 | -1.38 | 1.18E-03 |
| WDFY2 | -1.38 | 7.27E-04 |
| EMP1 | -1.38 | 3.75E-02 |
| CFAP69 | -1.38 | 6.50E-04 |
| NCOR2 | -1.38 | 4.81E-02 |
| PALM | -1.38 | 1.12E-02 |
| HSPB2 | -1.37 | 8.57E-04 |
| VPS36 | -1.37 | 1.89E-03 |
| TMX4 | -1.37 | 2.14E-02 |
| ZC3H6 | -1.37 | 4.24E-03 |
| TECPR2 | -1.36 | 8.02E-04 |
| ZNF300P1 | -1.36 | 1.04E-05 |
| TIMP2 | -1.36 | 2.65E-03 |
| TTC12 | -1.36 | 2.43E-02 |
| TPTEP1 | -1.36 | 1.41E-02 |
| DAB2 | -1.36 | 5.82E-03 |
| EPDR1 | -1.36 | 4.03E-02 |
| LRCH1 | -1.35 | 2.34E-03 |
| NUMA1 | -1.35 | 5.73E-03 |
| DAAM2 | -1.35 | 1.82E-03 |
| SOX17 | -1.35 | 1.37E-05 |
| GRK3 | -1.35 | 1.92E-02 |
| TACSTD2 | -1.35 | 4.84E-02 |
| PTPN14 | -1.35 | 1.19E-02 |
| MAP3K1 | -1.35 | 5.07E-03 |
| KIAA1671 | -1.35 | 5.71E-03 |
| ZBTB44 | -1.35 | 2.80E-03 |
| PRAG1 | -1.34 | 1.56E-02 |
| CD14 | -1.34 | 4.60E-02 |
| TAT | -1.34 | 2.91E-02 |
| LCAT | -1.34 | 1.44E-03 |
| ZNF689 | -1.34 | 2.24E-02 |
| TEF | -1.33 | 9.70E-05 |
| NR3C1 | -1.33 | 1.92E-02 |
| PYROXD2 | -1.33 | 4.12E-02 |
| DPYD | -1.33 | 2.98E-02 |
| MAPKBP1 | -1.32 | 1.51E-02 |
| RGL1 | -1.32 | 5.73E-04 |
| CCDC80 | -1.32 | 4.67E-02 |
| EOGT | -1.32 | 3.99E-03 |
| GLA | -1.32 | 1.55E-02 |
| NSA2 | -1.32 | 1.38E-05 |
| P2RY14 | -1.32 | 1.83E-02 |
| SLC4A7 | -1.32 | 2.63E-03 |
| MARCHF8 | -1.31 | 1.05E-03 |
| SGSM2 | -1.31 | 5.26E-03 |
| WDR6 | -1.31 | 1.92E-02 |
| SIRPA | -1.31 | 2.72E-02 |
| AFMID | -1.31 | 7.03E-03 |
| LPP | -1.31 | 1.88E-03 |
| KLF3-AS1 | -1.31 | 8.73E-05 |
| HPD | -1.31 | 3.84E-03 |
| PLXDC2 | -1.31 | 8.28E-03 |
| FOXP1 | -1.31 | 2.39E-02 |
| CRY2 | -1.31 | 4.95E-05 |
| CASD1 | -1.3 | 2.26E-02 |
| NEK10 | -1.3 | 1.42E-02 |
| BBS2 | -1.3 | 1.91E-03 |
| WDR86 | -1.3 | 9.30E-06 |
| LETMD1 | -1.3 | 1.89E-04 |
| THRSP | -1.29 | 2.29E-02 |
| GHDC | -1.29 | 2.50E-02 |
| LOC728392 | -1.29 | 1.91E-02 |
| ALDH6A1 | -1.29 | 3.89E-02 |
| THYN1 | -1.29 | 4.41E-04 |
| PRRC2B | -1.29 | 6.67E-03 |
| KLHL2 | -1.29 | 2.57E-03 |
| ZNF559 | -1.29 | 4.51E-02 |
| ALB | -1.29 | 7.48E-03 |
| ARRDC3 | -1.29 | 7.40E-04 |
| ZNF540 | -1.29 | 1.65E-03 |
| CCNL1 | -1.29 | 1.22E-03 |
| DYNC2H1 | -1.29 | 1.91E-03 |
| TFAP2C | -1.28 | 2.31E-02 |
| CLMN | -1.28 | 1.53E-02 |
| PLK3 | -1.28 | 3.57E-03 |
| ECE1 | -1.28 | 1.46E-02 |
| C1S | -1.28 | 4.84E-02 |
| CCDC8 | -1.28 | 1.83E-04 |
| CCNDBP1 | -1.28 | 5.47E-03 |
| TRPC1 | -1.28 | 2.20E-02 |
| PECAM1 | -1.28 | 8.91E-04 |
| CCAR2 | -1.28 | 4.88E-02 |
| GIMAP8 | -1.28 | 7.54E-03 |
| TSKU | -1.28 | 1.67E-02 |
| KCTD1 | -1.27 | 3.56E-02 |
| KCTD12 | -1.27 | 1.61E-03 |
| DDR2 | -1.27 | 2.68E-02 |
| RARB | -1.27 | 3.14E-02 |
| CAT | -1.27 | 6.99E-03 |
| SAP30L | -1.27 | 4.35E-03 |
| LYRM9 | -1.27 | 1.60E-04 |
| IL4R | -1.27 | 8.40E-04 |
| ENG | -1.26 | 2.62E-03 |
| PSMA3-AS1 | -1.26 | 5.39E-03 |
| AACS | -1.26 | 1.27E-02 |
| NAP1L5 | -1.26 | 1.97E-02 |
| FGF7 | -1.26 | 1.42E-02 |
| ACAD8 | -1.26 | 1.50E-02 |
| FAM172A | -1.26 | 2.02E-02 |
| PRNP | -1.26 | 2.27E-02 |
| ARHGEF10 | -1.26 | 4.76E-02 |
| INTS6L | -1.26 | 2.00E-02 |
| ARL15 | -1.26 | 3.89E-02 |
| LIMS2 | -1.26 | 4.13E-06 |
| KLHL31 | -1.26 | 1.53E-02 |
| ZNF655 | -1.26 | 4.75E-02 |
| ARSD | -1.25 | 3.05E-02 |
| PELI1 | -1.25 | 4.74E-02 |
| IGBP1 | -1.25 | 1.07E-03 |
| RCBTB2 | -1.25 | 6.02E-03 |
| SCPEP1 | -1.25 | 3.14E-02 |
| VILL | -1.25 | 4.76E-02 |
| ARHGEF6 | -1.24 | 1.59E-02 |
| CCNG1 | -1.24 | 8.65E-03 |
| ZNF740 | -1.24 | 1.62E-02 |
| ZNF385B | -1.24 | 3.10E-02 |
| BCKDHB | -1.24 | 2.39E-02 |
| TOM1L2 | -1.24 | 3.62E-03 |
| FLNA | -1.24 | 8.19E-03 |
| CRYZL2P | -1.24 | 4.84E-02 |
| ZNF10 | -1.23 | 1.73E-04 |
| TRIM56 | -1.23 | 8.37E-03 |
| PRRG3 | -1.23 | 4.08E-03 |
| TMEM30B | -1.23 | 2.29E-02 |
| OGT | -1.23 | 4.64E-04 |
| TSPYL2 | -1.23 | 4.97E-03 |
| XPC | -1.23 | 1.95E-04 |
| LATS2 | -1.23 | 2.25E-02 |
| PHYHIP | -1.23 | 1.19E-06 |
| DUSP16 | -1.22 | 1.30E-02 |
| GYPC | -1.22 | 7.26E-03 |
| FSTL1 | -1.22 | 1.04E-02 |
| GSDME | -1.22 | 9.37E-03 |
| CCDC178 | -1.22 | 2.55E-03 |
| SERTAD3 | -1.22 | 3.60E-02 |
| TESK2 | -1.22 | 4.74E-02 |
| GPAM | -1.22 | 1.12E-02 |
| RGMB | -1.22 | 2.26E-02 |
| GUSB | -1.22 | 1.98E-02 |
| OAF | -1.21 | 4.30E-05 |
| KMT2A | -1.21 | 1.03E-02 |
| C19orf18 | -1.21 | 1.01E-02 |
| PEAK1 | -1.21 | 1.11E-04 |
| PSMD6-AS2 | -1.21 | 4.03E-03 |
| HNMT | -1.21 | 1.10E-02 |
| CYP4F22 | -1.21 | 4.97E-02 |
| P2RY13 | -1.2 | 6.45E-03 |
| RAPGEF3 | -1.2 | 1.52E-02 |
| PXN | -1.2 | 6.78E-03 |
| BNIP3L | -1.2 | 2.30E-03 |
| MEDAG | -1.2 | 2.29E-02 |
| MYH10 | -1.2 | 2.39E-03 |
| PARVA | -1.2 | 3.81E-05 |
| CH25H | -1.2 | 8.01E-04 |
| SNRNP70 | -1.2 | 2.99E-03 |
| EP400 | -1.2 | 4.47E-02 |
| PYGB | -1.2 | 1.69E-02 |
| DNASE1L3 | -1.2 | 1.26E-04 |
| LMO2 | -1.19 | 2.11E-03 |
| TMEM220 | -1.19 | 3.37E-02 |
| PPP1R15A | -1.19 | 1.05E-03 |
| EMILIN1 | -1.19 | 3.44E-02 |
| CHD9 | -1.19 | 1.05E-03 |
| FHIT | -1.19 | 4.36E-02 |
| SIDT2 | -1.19 | 3.37E-02 |
| XDH | -1.19 | 2.53E-02 |
| TTN-AS1 | -1.18 | 2.70E-02 |
| POLR2A | -1.18 | 4.85E-02 |
| TRARG1 | -1.18 | 7.76E-06 |
| PLP1 | -1.18 | 1.02E-02 |
| PCGF5 | -1.18 | 1.25E-02 |
| BBS1 | -1.18 | 3.35E-02 |
| IMPDH2 | -1.18 | 7.23E-04 |
| SOX7 | -1.18 | 1.37E-03 |
| LINC01278 | -1.18 | 5.68E-03 |
| GALNT15 | -1.18 | 4.47E-03 |
| PJVK | -1.18 | 2.45E-02 |
| TSHZ3 | -1.18 | 2.53E-02 |
| GNB5 | -1.18 | 1.19E-02 |
| ROBO3 | -1.18 | 7.56E-05 |
| CSF1R | -1.18 | 1.85E-02 |
| ESD | -1.17 | 1.66E-02 |
| EPHA2 | -1.17 | 4.59E-03 |
| KLF9 | -1.17 | 3.50E-02 |
| CCNL2 | -1.17 | 1.56E-02 |
| CORO2B | -1.17 | 1.31E-09 |
| TPM1 | -1.17 | 1.21E-02 |
| LONRF1 | -1.17 | 1.85E-02 |
| FAM122A | -1.16 | 9.41E-03 |
| SYN2 | -1.16 | 5.35E-05 |
| MYLIP | -1.16 | 2.76E-02 |
| TBC1D4 | -1.16 | 1.08E-02 |
| HSPB7 | -1.16 | 8.16E-04 |
| SIK2 | -1.16 | 2.32E-04 |
| ARAP3 | -1.15 | 4.77E-03 |
| GALNT16 | -1.15 | 3.73E-02 |
| KDM3B | -1.15 | 3.69E-03 |
| BCL6 | -1.15 | 2.92E-02 |
| TUBGCP6 | -1.15 | 1.32E-02 |
| N4BP2L2 | -1.15 | 2.85E-02 |
| ALDH7A1 | -1.15 | 4.55E-03 |
| SAT2 | -1.15 | 7.68E-03 |
| PFDN5 | -1.15 | 6.27E-04 |
| FST | -1.15 | 5.45E-03 |
| TNKS | -1.15 | 3.85E-02 |
| GLUD1 | -1.15 | 1.23E-02 |
| HIF1AN | -1.15 | 8.57E-04 |
| CRTAP | -1.14 | 4.44E-04 |
| NPTN-IT1 | -1.14 | 3.37E-02 |
| SMARCA2 | -1.14 | 1.82E-03 |
| ZNF219 | -1.14 | 7.41E-03 |
| SAV1 | -1.14 | 1.89E-02 |
| MXRA7 | -1.14 | 5.59E-03 |
| NEDD4L | -1.14 | 4.38E-02 |
| GLIDR | -1.14 | 4.81E-02 |
| LPCAT4 | -1.14 | 2.56E-02 |
| NBR1 | -1.14 | 1.75E-03 |
| EFCAB14 | -1.14 | 8.50E-06 |
| TMEM245 | -1.14 | 1.44E-04 |
| BIN3 | -1.14 | 3.42E-05 |
| KLC1 | -1.14 | 1.78E-04 |
| SPTAN1 | -1.13 | 1.99E-03 |
| PDGFRB | -1.13 | 4.97E-02 |
| SNTB2 | -1.13 | 2.95E-04 |
| C11orf1 | -1.13 | 1.15E-02 |
| CREBRF | -1.13 | 1.91E-02 |
| NAB1 | -1.13 | 3.04E-02 |
| PDE1C | -1.13 | 1.68E-03 |
| HSD17B4 | -1.13 | 7.08E-03 |
| C14orf28 | -1.13 | 1.78E-03 |
| KLF10 | -1.12 | 1.23E-02 |
| RYR3 | -1.12 | 2.79E-02 |
| SHROOM3 | -1.12 | 1.03E-02 |
| SGSH | -1.12 | 1.67E-03 |
| ARL4C | -1.12 | 3.42E-02 |
| PDK2 | -1.12 | 3.64E-02 |
| SH3BP5-AS1 | -1.12 | 7.19E-03 |
| FAM3D | -1.12 | 7.86E-03 |
| ZSWIM8 | -1.11 | 2.26E-02 |
| PARD3B | -1.11 | 9.65E-04 |
| TRNP1 | -1.11 | 4.63E-02 |
| SDC3 | -1.11 | 1.26E-02 |
| B4GAT1 | -1.11 | 2.85E-02 |
| GPR146 | -1.11 | 1.34E-04 |
| LRRC37A2 | -1.11 | 1.66E-02 |
| LTC4S | -1.1 | 3.86E-06 |
| C20orf194 | -1.1 | 1.21E-02 |
| NAP1L3 | -1.1 | 1.20E-02 |
| TINAGL1 | -1.1 | 2.53E-03 |
| AUH | -1.1 | 2.30E-02 |
| UNC119B | -1.1 | 8.14E-03 |
| AASS | -1.1 | 6.91E-04 |
| MAFK | -1.1 | 1.61E-02 |
| DHRS1 | -1.09 | 7.41E-03 |
| SYNE2 | -1.09 | 1.25E-02 |
| TMEM91 | -1.09 | 1.94E-03 |
| NPHP3 | -1.09 | 1.64E-03 |
| EIF3G | -1.09 | 1.49E-02 |
| ACSS2 | -1.09 | 1.22E-02 |
| ABTB1 | -1.09 | 4.74E-02 |
| PTEN | -1.09 | 5.18E-04 |
| MAP3K20 | -1.08 | 2.26E-02 |
| MTFR1L | -1.08 | 4.08E-03 |
| TMEM198B | -1.08 | 5.80E-04 |
| KLHDC2 | -1.08 | 2.32E-02 |
| TLN1 | -1.08 | 4.77E-02 |
| TEAD1 | -1.08 | 1.93E-02 |
| ADCY4 | -1.08 | 9.09E-03 |
| ZFYVE21 | -1.08 | 2.62E-03 |
| LRP10 | -1.08 | 2.59E-03 |
| OSBPL9 | -1.08 | 1.05E-03 |
| ADAMTS8 | -1.08 | 7.42E-04 |
| PHYKPL | -1.07 | 6.35E-03 |
| EBF4 | -1.07 | 3.75E-02 |
| BEGAIN | -1.07 | 4.44E-04 |
| ATP6V0A1 | -1.07 | 4.40E-03 |
| ECSCR | -1.07 | 1.40E-05 |
| NXF1 | -1.07 | 2.19E-04 |
| ADD1 | -1.07 | 4.36E-03 |
| MEIS3P1 | -1.07 | 2.92E-02 |
| APOLD1 | -1.07 | 1.96E-02 |
| ARGLU1 | -1.07 | 1.06E-02 |
| PURA | -1.07 | 1.91E-03 |
| CNTN1 | -1.07 | 1.75E-02 |
| ADGRD1 | -1.07 | 5.56E-03 |
| SYT8 | -1.07 | 2.02E-02 |
| MRNIP | -1.07 | 1.02E-02 |
| PREX2 | -1.06 | 1.78E-04 |
| FAM117A | -1.06 | 4.43E-02 |
| ABL1 | -1.06 | 6.55E-03 |
| WWC1 | -1.06 | 1.58E-02 |
| AFF1 | -1.06 | 3.43E-02 |
| HMGCS2 | -1.06 | 2.62E-02 |
| ATG14 | -1.06 | 7.90E-06 |
| CTDSP2 | -1.05 | 1.14E-03 |
| TMEM273 | -1.05 | 1.01E-02 |
| SEC31B | -1.05 | 5.31E-03 |
| SF1 | -1.04 | 2.76E-03 |
| EIF3L | -1.04 | 1.41E-03 |
| CTDSP1 | -1.04 | 4.28E-03 |
| C6orf120 | -1.04 | 1.67E-03 |
| CAST | -1.04 | 1.29E-02 |
| LIX1L | -1.04 | 1.13E-02 |
| CMA1 | -1.04 | 2.79E-03 |
| RAPGEF2 | -1.04 | 1.34E-04 |
| AXIN2 | -1.04 | 1.24E-02 |
| FYCO1 | -1.04 | 1.92E-02 |
| LOC286437 | -1.03 | 1.62E-03 |
| DHRS3 | -1.03 | 3.95E-02 |
| ATF7 | -1.03 | 1.03E-02 |
| WASF2 | -1.03 | 2.22E-03 |
| RILPL2 | -1.03 | 6.65E-03 |
| CHID1 | -1.03 | 1.10E-04 |
| ZFP36L1 | -1.03 | 1.68E-02 |
| PER3 | -1.03 | 3.54E-03 |
| ZKSCAN8 | -1.03 | 1.93E-02 |
| CIC | -1.03 | 1.73E-02 |
| PLEKHA7 | -1.02 | 4.69E-03 |
| ANKRD65 | -1.02 | 3.44E-04 |
| KYAT1 | -1.02 | 4.69E-03 |
| SPEN | -1.02 | 4.95E-02 |
| SH3PXD2B | -1.02 | 3.05E-03 |
| P2RY12 | -1.02 | 1.77E-04 |
| CALD1 | -1.02 | 2.26E-02 |
| SRF | -1.02 | 2.64E-02 |
| TRABD2B | -1.02 | 1.31E-04 |
| CD93 | -1.01 | 4.98E-02 |
| FOSL2 | -1.01 | 3.82E-02 |
| VCL | -1.01 | 2.52E-02 |
| ZER1 | -1.01 | 7.78E-03 |
| RNLS | -1.01 | 3.07E-02 |
| SYNPO | -1.01 | 1.84E-03 |
| LMBRD1 | -1.01 | 1.18E-03 |
| NRP1 | -1.01 | 3.78E-02 |
| SAFB2 | -1.01 | 2.71E-02 |
| ADGRD2 | -1.01 | 1.44E-03 |
| HYI | -1 | 4.02E-02 |
| POPDC2 | -1 | 2.50E-04 |
| ZYX | -1 | 1.30E-02 |
| DNAJC9 | 1 | 2.41E-03 |
| CD3EAP | 1 | 1.44E-02 |
| SLC35A2 | 1 | 7.65E-03 |
| TMEM79 | 1 | 3.18E-02 |
| KIAA1841 | 1 | 1.75E-02 |
| ND6 | 1 | 3.14E-02 |
| VPS13B | 1.01 | 4.61E-02 |
| HSPA14 | 1.01 | 2.09E-02 |
| ZNF236 | 1.01 | 1.46E-03 |
| TADA1 | 1.01 | 3.83E-03 |
| PLEK2 | 1.01 | 3.69E-02 |
| EIF5B | 1.01 | 1.86E-03 |
| CIAO1 | 1.01 | 1.15E-03 |
| CDK8 | 1.01 | 2.84E-02 |
| CEP97 | 1.01 | 2.26E-02 |
| ZNF281 | 1.01 | 3.38E-02 |
| R3HDM1 | 1.01 | 2.86E-04 |
| ABCB10 | 1.01 | 1.11E-02 |
| CPSF3 | 1.01 | 1.38E-02 |
| YEATS4 | 1.01 | 2.97E-02 |
| MRPS17 | 1.02 | 4.70E-02 |
| ELL3 | 1.02 | 1.02E-02 |
| RAE1 | 1.02 | 1.70E-02 |
| TMEM68 | 1.02 | 4.44E-02 |
| UBXN7 | 1.02 | 1.26E-02 |
| GINS3 | 1.02 | 3.14E-02 |
| CCT3 | 1.02 | 2.99E-04 |
| ACOT13 | 1.03 | 2.41E-02 |
| MRGBP | 1.03 | 8.19E-03 |
| LINC01208 | 1.03 | 9.37E-03 |
| LINC00520 | 1.03 | 3.93E-02 |
| GNAO1 | 1.03 | 6.18E-03 |
| NOL10 | 1.03 | 2.37E-03 |
| ADA | 1.03 | 1.88E-02 |
| DR1 | 1.03 | 5.41E-03 |
| BRCA1 | 1.03 | 1.37E-02 |
| PCBD2 | 1.03 | 7.74E-03 |
| RPAP3 | 1.03 | 2.75E-03 |
| APOO | 1.04 | 1.16E-02 |
| DSN1 | 1.04 | 1.43E-04 |
| BAZ1A | 1.04 | 1.53E-02 |
| PRKDC | 1.04 | 3.58E-02 |
| UNG | 1.05 | 1.26E-02 |
| WDHD1 | 1.05 | 5.40E-03 |
| G2E3 | 1.05 | 4.49E-03 |
| CSNK2A1 | 1.05 | 8.67E-04 |
| LINC00467 | 1.05 | 3.63E-02 |
| TUBG1 | 1.05 | 9.23E-03 |
| SLC7A7 | 1.06 | 3.20E-02 |
| BFAR | 1.06 | 1.73E-04 |
| SAYSD1 | 1.06 | 7.99E-03 |
| HPRT1 | 1.06 | 2.79E-02 |
| MID1IP1 | 1.06 | 2.17E-02 |
| FAHD1 | 1.06 | 3.58E-04 |
| VRK2 | 1.06 | 1.65E-03 |
| DERL1 | 1.06 | 1.53E-03 |
| TSEN15 | 1.06 | 1.63E-02 |
| UBQLN4 | 1.06 | 2.49E-02 |
| GMPS | 1.07 | 1.63E-02 |
| RECQL4 | 1.07 | 2.56E-02 |
| HGH1 | 1.07 | 9.51E-03 |
| PKMYT1 | 1.07 | 2.21E-03 |
| PSMC3IP | 1.07 | 2.99E-03 |
| RAB22A | 1.07 | 1.41E-04 |
| TAF1B | 1.07 | 8.53E-03 |
| ARPC1B | 1.08 | 4.84E-02 |
| ACOX1 | 1.08 | 1.23E-02 |
| ELOC | 1.08 | 2.07E-03 |
| COQ2 | 1.08 | 8.98E-03 |
| URB2 | 1.08 | 2.67E-03 |
| TAF5L | 1.08 | 5.26E-04 |
| SLBP | 1.08 | 2.24E-03 |
| EP300-AS1 | 1.08 | 1.57E-02 |
| MSH6 | 1.08 | 1.35E-03 |
| ME2 | 1.08 | 4.11E-02 |
| KCTD5 | 1.09 | 3.87E-04 |
| USP38 | 1.09 | 9.23E-03 |
| RIF1 | 1.09 | 5.35E-03 |
| SFXN1 | 1.09 | 4.30E-03 |
| RAD54L | 1.09 | 3.47E-03 |
| SKA2 | 1.09 | 2.49E-02 |
| SPRTN | 1.1 | 2.62E-02 |
| TMEM132A | 1.1 | 4.86E-02 |
| CDK16 | 1.1 | 2.44E-02 |
| CABLES2 | 1.1 | 1.70E-02 |
| IMPDH1 | 1.1 | 6.87E-03 |
| PNO1 | 1.1 | 1.04E-02 |
| ADAM19 | 1.1 | 1.67E-02 |
| MIR302B | 1.1 | 9.28E-03 |
| ATP23 | 1.1 | 3.88E-02 |
| PRDX4 | 1.11 | 6.99E-03 |
| SMC2 | 1.11 | 8.03E-04 |
| C5orf34 | 1.11 | 4.81E-02 |
| POLR2K | 1.11 | 8.64E-03 |
| YIPF4 | 1.11 | 2.13E-02 |
| UBE2F | 1.11 | 4.92E-04 |
| H2AZ2 | 1.11 | 6.28E-03 |
| UTP23 | 1.12 | 4.04E-03 |
| PRIM2 | 1.12 | 1.66E-02 |
| SEC24D | 1.12 | 1.43E-02 |
| FBXL6 | 1.12 | 4.49E-02 |
| PFDN4 | 1.12 | 9.18E-03 |
| LINC01560 | 1.13 | 2.20E-02 |
| TBC1D7 | 1.13 | 3.58E-03 |
| CCT6A | 1.13 | 3.95E-03 |
| B3GALNT1 | 1.13 | 1.36E-02 |
| GEMIN6 | 1.13 | 5.60E-04 |
| PPP1R14B | 1.13 | 2.69E-02 |
| MRPL12 | 1.13 | 1.21E-02 |
| AGO3 | 1.13 | 9.69E-03 |
| GFPT1 | 1.13 | 2.98E-03 |
| EXOSC3 | 1.13 | 9.91E-03 |
| PSMD12 | 1.14 | 1.70E-02 |
| CENPH | 1.14 | 1.75E-02 |
| C1orf131 | 1.14 | 3.34E-04 |
| PUS7 | 1.14 | 1.07E-02 |
| CENPI | 1.14 | 3.65E-02 |
| TASOR2 | 1.14 | 2.88E-02 |
| ELF4 | 1.14 | 6.55E-03 |
| ADCY7 | 1.14 | 4.67E-02 |
| HES6 | 1.14 | 3.37E-02 |
| TCTEX1D2 | 1.15 | 2.71E-02 |
| RUSC1-AS1 | 1.15 | 5.45E-03 |
| POLR2D | 1.15 | 3.29E-03 |
| STRBP | 1.15 | 2.54E-04 |
| ZHX1-C8orf76 | 1.15 | 4.68E-02 |
| CXorf38 | 1.16 | 1.76E-03 |
| MEX3A | 1.16 | 4.53E-02 |
| RMDN1 | 1.17 | 6.21E-03 |
| CST4 | 1.17 | 1.79E-02 |
| MCM8 | 1.17 | 1.36E-03 |
| PARP12 | 1.17 | 2.81E-02 |
| CCDC18 | 1.17 | 3.58E-02 |
| KPNA4 | 1.17 | 2.99E-03 |
| FSD1L | 1.18 | 1.33E-02 |
| POC1A | 1.18 | 1.14E-02 |
| SLC25A32 | 1.18 | 5.13E-03 |
| RCC1 | 1.18 | 9.37E-03 |
| LSM4 | 1.18 | 7.80E-05 |
| HTATIP2 | 1.18 | 1.41E-03 |
| SKA1 | 1.18 | 1.92E-02 |
| ARL11 | 1.18 | 1.50E-02 |
| FAM210A | 1.19 | 4.45E-02 |
| MCRIP2 | 1.19 | 4.51E-02 |
| PSMD14 | 1.19 | 1.65E-07 |
| NPL | 1.19 | 5.95E-03 |
| TTC13 | 1.19 | 2.26E-03 |
| CRCP | 1.19 | 1.22E-05 |
| CHCHD3 | 1.2 | 1.35E-04 |
| STRN | 1.2 | 1.92E-03 |
| TAF2 | 1.2 | 8.15E-04 |
| MCM3AP-AS1 | 1.2 | 3.96E-02 |
| RIDA | 1.2 | 3.27E-02 |
| HAUS3 | 1.21 | 3.44E-04 |
| VANGL1 | 1.21 | 5.69E-03 |
| CD80 | 1.21 | 1.02E-02 |
| VEGFA | 1.21 | 3.24E-02 |
| AHCTF1 | 1.21 | 4.55E-04 |
| TBCE | 1.21 | 3.42E-05 |
| FOXP4 | 1.21 | 3.86E-02 |
| ZNF184 | 1.21 | 2.11E-03 |
| C3orf38 | 1.21 | 7.16E-03 |
| SNRNP25 | 1.21 | 6.81E-04 |
| MRPL14 | 1.21 | 2.07E-03 |
| GLRX2 | 1.22 | 2.92E-04 |
| AMMECR1 | 1.22 | 4.44E-02 |
| CCDC125 | 1.22 | 2.63E-02 |
| TICRR | 1.22 | 3.79E-03 |
| TCAIM | 1.22 | 1.49E-02 |
| TFAM | 1.22 | 9.14E-03 |
| PARPBP | 1.23 | 9.17E-03 |
| KIF2A | 1.23 | 1.23E-02 |
| MARCHF1 | 1.23 | 9.08E-03 |
| HTATSF1P2 | 1.23 | 7.36E-03 |
| SPAST | 1.23 | 6.77E-03 |
| BPNT1 | 1.23 | 2.35E-03 |
| CALCRL | 1.23 | 3.22E-02 |
| DNA2 | 1.23 | 5.74E-03 |
| ENTPD7 | 1.24 | 1.43E-03 |
| OTULIN | 1.24 | 1.96E-03 |
| PRELID3B | 1.24 | 5.31E-03 |
| PPIL1 | 1.24 | 4.30E-04 |
| METTL8 | 1.25 | 2.75E-04 |
| PTTG3P | 1.25 | 2.33E-03 |
| SALL4 | 1.25 | 1.26E-03 |
| WDR12 | 1.25 | 1.45E-05 |
| PGM3 | 1.25 | 1.67E-02 |
| HAVCR2 | 1.25 | 2.58E-02 |
| GARS1 | 1.26 | 3.37E-04 |
| TIPRL | 1.26 | 1.71E-03 |
| PCDH17 | 1.26 | 1.37E-02 |
| TIMM17A | 1.26 | 4.48E-05 |
| RAB23 | 1.26 | 4.12E-02 |
| BRCA2 | 1.26 | 3.40E-02 |
| DLEU1 | 1.26 | 9.60E-03 |
| CCDC58 | 1.26 | 4.84E-02 |
| POP1 | 1.26 | 1.57E-02 |
| PDSS1 | 1.26 | 1.50E-02 |
| DCBLD1 | 1.26 | 1.52E-02 |
| ESRP1 | 1.26 | 4.23E-03 |
| RBL1 | 1.27 | 8.92E-03 |
| MRPL15 | 1.27 | 2.11E-02 |
| GTPBP4 | 1.27 | 1.02E-02 |
| ZNF267 | 1.27 | 2.35E-04 |
| KNSTRN | 1.27 | 9.03E-03 |
| PARP1 | 1.28 | 3.93E-04 |
| SLA | 1.28 | 2.93E-02 |
| LINC01094 | 1.28 | 6.89E-03 |
| CACYBP | 1.28 | 2.11E-04 |
| STX6 | 1.28 | 3.39E-05 |
| DTYMK | 1.28 | 4.09E-04 |
| CCR1 | 1.29 | 2.19E-02 |
| UBE2V2 | 1.29 | 4.67E-04 |
| NUP155 | 1.29 | 1.29E-02 |
| BID | 1.3 | 1.92E-02 |
| SMC5 | 1.3 | 4.05E-03 |
| DPP3 | 1.3 | 8.31E-03 |
| RPP40 | 1.31 | 1.15E-02 |
| FAM20B | 1.31 | 1.54E-04 |
| PTP4A3 | 1.31 | 1.31E-02 |
| DEPDC1B | 1.31 | 1.48E-02 |
| GART | 1.31 | 5.37E-04 |
| GPR180 | 1.32 | 2.18E-02 |
| SYNCRIP | 1.32 | 1.66E-03 |
| SLC2A1 | 1.32 | 1.32E-02 |
| SPATS2 | 1.32 | 2.90E-04 |
| RAD21 | 1.33 | 2.59E-04 |
| TMEM97 | 1.33 | 3.41E-02 |
| BYSL | 1.33 | 1.45E-02 |
| FLAD1 | 1.33 | 4.99E-04 |
| C11orf80 | 1.33 | 4.55E-02 |
| PTPDC1 | 1.34 | 6.32E-03 |
| SMC6 | 1.34 | 5.98E-04 |
| LIN9 | 1.34 | 8.15E-04 |
| SDC1 | 1.34 | 4.49E-02 |
| DHTKD1 | 1.34 | 4.17E-03 |
| COA6 | 1.35 | 3.18E-03 |
| CNIH4 | 1.35 | 6.90E-04 |
| STAT1 | 1.35 | 1.91E-02 |
| FAM111B | 1.36 | 1.17E-02 |
| PSMA7 | 1.36 | 4.40E-06 |
| AP1S1 | 1.36 | 5.71E-04 |
| PANK1 | 1.36 | 9.23E-03 |
| METTL21A | 1.36 | 4.57E-03 |
| CKS1B | 1.36 | 1.67E-02 |
| TFRC | 1.36 | 1.41E-02 |
| DNMT3B | 1.36 | 2.92E-02 |
| RIPK2 | 1.37 | 9.40E-03 |
| CCDC186 | 1.37 | 2.66E-02 |
| CD300LF | 1.37 | 4.59E-03 |
| TMEM45A | 1.38 | 1.48E-02 |
| RFC5 | 1.38 | 7.83E-04 |
| MRPL19 | 1.38 | 5.14E-04 |
| LMNB2 | 1.38 | 1.39E-02 |
| KNTC1 | 1.38 | 1.27E-04 |
| NFKBIE | 1.38 | 1.17E-02 |
| WDYHV1 | 1.39 | 2.56E-04 |
| NAA15 | 1.39 | 2.27E-05 |
| HOMER3 | 1.39 | 1.36E-02 |
| INTS8 | 1.39 | 4.32E-04 |
| FAM49B | 1.39 | 2.73E-07 |
| DCK | 1.39 | 1.75E-03 |
| HSD17B6 | 1.39 | 7.68E-03 |
| MIS18A | 1.4 | 3.00E-03 |
| SLC12A8 | 1.4 | 2.45E-02 |
| NCBP1 | 1.4 | 1.60E-03 |
| MAL2 | 1.4 | 8.31E-03 |
| ZNF124 | 1.4 | 7.50E-03 |
| RIT1 | 1.4 | 2.26E-02 |
| DAPK1 | 1.41 | 1.33E-02 |
| H2AX | 1.41 | 8.96E-04 |
| PLAUR | 1.42 | 1.14E-02 |
| C12orf73 | 1.42 | 2.96E-04 |
| NCK1-DT | 1.42 | 7.88E-03 |
| AGPS | 1.42 | 1.11E-02 |
| ERCC6L | 1.43 | 6.54E-03 |
| AP1S2 | 1.43 | 2.38E-03 |
| KIF26B | 1.43 | 2.30E-02 |
| BAIAP2L1 | 1.43 | 1.48E-02 |
| SYNDIG1 | 1.43 | 6.59E-03 |
| ACTL6A | 1.44 | 3.71E-04 |
| RBM12B | 1.44 | 5.16E-05 |
| SLC25A19 | 1.44 | 6.51E-04 |
| SLC52A2 | 1.44 | 2.67E-03 |
| C16orf87 | 1.45 | 1.18E-03 |
| NCAPD2 | 1.45 | 1.06E-02 |
| CENPL | 1.45 | 8.17E-04 |
| RFWD3 | 1.45 | 6.25E-03 |
| PYCR1 | 1.45 | 4.35E-04 |
| MTERF3 | 1.46 | 1.42E-03 |
| TNFAIP6 | 1.46 | 4.75E-02 |
| MSR1 | 1.46 | 2.19E-03 |
| NUDT5 | 1.47 | 5.16E-03 |
| PNPT1 | 1.47 | 9.37E-05 |
| ATP6V1C1 | 1.48 | 2.32E-03 |
| TTF2 | 1.48 | 7.54E-03 |
| DDX39A | 1.48 | 3.30E-05 |
| E2F5 | 1.48 | 3.56E-02 |
| TIPIN | 1.48 | 1.50E-02 |
| NME1 | 1.48 | 5.18E-03 |
| SNX10 | 1.49 | 1.32E-02 |
| ZWILCH | 1.49 | 2.35E-04 |
| RSRC1 | 1.49 | 6.06E-04 |
| ACOT7 | 1.5 | 1.02E-02 |
| IGSF6 | 1.5 | 1.33E-02 |
| CENPA | 1.5 | 5.34E-03 |
| MTDH | 1.5 | 1.37E-04 |
| H2AZ1 | 1.5 | 3.51E-06 |
| RRP15 | 1.52 | 1.87E-05 |
| EPRS1 | 1.52 | 3.46E-04 |
| RGS1 | 1.52 | 1.12E-02 |
| FANCD2 | 1.52 | 6.99E-03 |
| INTS7 | 1.52 | 1.73E-04 |
| AGO2 | 1.53 | 8.19E-03 |
| NUDCD1 | 1.53 | 4.62E-04 |
| MTFR1 | 1.53 | 4.20E-04 |
| MCOLN2 | 1.53 | 3.75E-02 |
| FLVCR1 | 1.54 | 2.90E-05 |
| IL4I1 | 1.54 | 2.17E-02 |
| PDXK | 1.54 | 2.11E-03 |
| NET1 | 1.55 | 8.58E-03 |
| TBC1D31 | 1.55 | 1.30E-02 |
| AUNIP | 1.56 | 7.49E-03 |
| CIP2A | 1.56 | 1.74E-03 |
| XPOT | 1.56 | 2.65E-05 |
| C8orf76 | 1.56 | 5.05E-03 |
| NDUFAF6 | 1.57 | 1.57E-03 |
| FBXO6 | 1.57 | 2.79E-02 |
| KDM1B | 1.57 | 8.49E-05 |
| PCNA | 1.57 | 8.02E-05 |
| DCAF13 | 1.58 | 2.92E-05 |
| MRPL13 | 1.58 | 1.45E-03 |
| ANKRD22 | 1.58 | 5.20E-03 |
| MRS2 | 1.58 | 5.38E-04 |
| RABIF | 1.59 | 5.67E-06 |
| GJC1 | 1.59 | 4.18E-02 |
| GALNT14 | 1.59 | 4.01E-02 |
| CHST11 | 1.59 | 2.22E-02 |
| PAQR4 | 1.6 | 2.01E-04 |
| HMGB2 | 1.6 | 3.11E-03 |
| SRPK1 | 1.6 | 2.41E-04 |
| CHEK1 | 1.6 | 8.69E-03 |
| TIMELESS | 1.6 | 2.72E-06 |
| EME1 | 1.6 | 2.70E-04 |
| ABRACL | 1.62 | 6.59E-03 |
| SLC7A11 | 1.62 | 1.96E-02 |
| CXCR4 | 1.62 | 8.65E-03 |
| CLEC7A | 1.62 | 3.56E-02 |
| GEN1 | 1.62 | 5.31E-06 |
| SLAMF8 | 1.62 | 1.08E-02 |
| RFC4 | 1.63 | 8.97E-04 |
| PNP | 1.63 | 1.71E-03 |
| SBK1 | 1.63 | 6.10E-03 |
| PSPH | 1.63 | 1.52E-03 |
| CDC73 | 1.64 | 2.69E-06 |
| DNMT3A | 1.64 | 6.73E-05 |
| HILPDA | 1.65 | 9.15E-03 |
| AIM2 | 1.65 | 4.03E-02 |
| UCHL5 | 1.65 | 4.81E-04 |
| HMOX1 | 1.66 | 1.70E-02 |
| TP53RK | 1.66 | 1.48E-04 |
| TMEM65 | 1.66 | 7.37E-04 |
| ITCH | 1.66 | 1.22E-05 |
| NCAPG2 | 1.66 | 5.62E-04 |
| F12 | 1.67 | 8.19E-03 |
| COTL1 | 1.67 | 1.97E-02 |
| IFIT2 | 1.67 | 3.17E-02 |
| SAC3D1 | 1.67 | 1.33E-03 |
| OTUD6B | 1.67 | 2.89E-04 |
| E2F8 | 1.68 | 6.38E-04 |
| POLQ | 1.69 | 6.20E-04 |
| UBE2W | 1.69 | 2.98E-05 |
| SUV39H2 | 1.69 | 9.11E-03 |
| STK26 | 1.7 | 1.08E-02 |
| RMI1 | 1.7 | 4.68E-06 |
| MASTL | 1.7 | 1.05E-03 |
| OAS3 | 1.7 | 2.56E-02 |
| SPC24 | 1.71 | 2.39E-03 |
| PLOD2 | 1.71 | 4.07E-03 |
| INHBC | 1.71 | 1.33E-04 |
| SSX2IP | 1.71 | 2.02E-04 |
| PAFAH1B3 | 1.71 | 6.03E-05 |
| MCM6 | 1.71 | 1.75E-04 |
| E2F2 | 1.71 | 1.22E-04 |
| TROAP | 1.72 | 8.64E-05 |
| SAPCD2 | 1.73 | 1.91E-03 |
| FBXO5 | 1.74 | 7.62E-03 |
| TLCD1 | 1.74 | 8.39E-03 |
| C4orf46 | 1.74 | 4.57E-03 |
| ORC6 | 1.74 | 7.65E-05 |
| CAPS | 1.74 | 1.16E-02 |
| CENPM | 1.75 | 5.18E-04 |
| BORA | 1.75 | 4.44E-04 |
| CENPW | 1.75 | 2.91E-02 |
| KIFC1 | 1.75 | 3.74E-04 |
| CHML | 1.76 | 5.41E-03 |
| FPR3 | 1.76 | 4.12E-02 |
| DDIAS | 1.77 | 5.40E-03 |
| NBN | 1.78 | 2.04E-05 |
| LRP8 | 1.78 | 2.22E-03 |
| CHAC2 | 1.78 | 7.10E-03 |
| HMGA1 | 1.79 | 8.11E-04 |
| USP18 | 1.79 | 2.88E-02 |
| PLK1 | 1.8 | 1.57E-02 |
| LAGE3 | 1.8 | 4.55E-04 |
| FBXO45 | 1.8 | 1.55E-05 |
| KIF21A | 1.8 | 4.15E-04 |
| CDCA2 | 1.8 | 1.69E-02 |
| KDELR3 | 1.8 | 8.94E-03 |
| PGP | 1.81 | 2.70E-06 |
| PLA2G7 | 1.81 | 1.63E-02 |
| SPAG5 | 1.82 | 1.73E-03 |
| SPAG1 | 1.82 | 9.63E-03 |
| RAD51 | 1.82 | 1.67E-05 |
| ZNF800 | 1.84 | 1.49E-04 |
| LRR1 | 1.84 | 1.22E-03 |
| COL8A1 | 1.85 | 2.27E-02 |
| PSRC1 | 1.85 | 1.64E-03 |
| DBF4 | 1.85 | 5.41E-04 |
| DONSON | 1.86 | 8.51E-05 |
| GGH | 1.86 | 4.24E-02 |
| OLR1 | 1.87 | 1.59E-02 |
| ZBED6 | 1.87 | 9.45E-03 |
| MCUB | 1.87 | 1.68E-03 |
| TAP1 | 1.87 | 1.58E-02 |
| RNFT2 | 1.87 | 3.03E-03 |
| RPL39L | 1.88 | 3.89E-03 |
| SIKE1 | 1.89 | 1.07E-04 |
| SDS | 1.9 | 6.15E-03 |
| BCAT1 | 1.91 | 2.53E-02 |
| MTHFD2 | 1.91 | 8.61E-08 |
| MICB | 1.91 | 2.40E-02 |
| TNFSF4 | 1.91 | 4.08E-03 |
| SAP30 | 1.91 | 1.97E-02 |
| DNAH14 | 1.93 | 1.17E-03 |
| SHCBP1 | 1.93 | 2.89E-04 |
| POLE2 | 1.94 | 2.71E-04 |
| KIF18B | 1.94 | 1.86E-04 |
| TEAD4 | 1.94 | 1.34E-03 |
| CENPK | 1.95 | 1.23E-03 |
| GPSM2 | 1.95 | 1.92E-04 |
| PPAT | 1.95 | 7.87E-06 |
| SLC7A5 | 1.97 | 1.03E-02 |
| CKAP2 | 1.98 | 7.89E-06 |
| TK1 | 1.99 | 1.37E-03 |
| TDRKH | 1.99 | 4.36E-03 |
| CEMIP | 1.99 | 6.45E-03 |
| FEN1 | 1.99 | 8.80E-05 |
| TFEC | 2 | 2.63E-02 |
| MND1 | 2 | 1.41E-03 |
| SQLE | 2.01 | 1.70E-03 |
| RNASEH2A | 2.01 | 5.58E-05 |
| MCM4 | 2.01 | 2.47E-05 |
| MAGOHB | 2.02 | 9.47E-04 |
| TYMP | 2.02 | 1.17E-02 |
| SMC4 | 2.04 | 1.46E-06 |
| HMGB3P1 | 2.04 | 5.68E-05 |
| TRIM59 | 2.04 | 2.89E-04 |
| ESPL1 | 2.05 | 2.31E-08 |
| MMP13 | 2.05 | 4.20E-03 |
| E2F3 | 2.08 | 5.07E-04 |
| HPSE | 2.08 | 1.06E-04 |
| CBS | 2.08 | 4.45E-02 |
| GINS2 | 2.09 | 2.89E-04 |
| E2F7 | 2.1 | 1.17E-02 |
| TCF19 | 2.1 | 1.09E-03 |
| KIF18A | 2.1 | 7.12E-04 |
| DYRK2 | 2.11 | 1.07E-05 |
| MCM2 | 2.11 | 6.12E-05 |
| EXO1 | 2.11 | 7.64E-05 |
| PACC1 | 2.11 | 1.91E-05 |
| FCGR1B | 2.12 | 4.05E-03 |
| ELOVL6 | 2.12 | 8.45E-04 |
| CCDC167 | 2.13 | 7.00E-07 |
| C1orf112 | 2.16 | 3.45E-05 |
| IFI6 | 2.16 | 2.70E-02 |
| STMN1 | 2.17 | 4.32E-06 |
| KIF23 | 2.18 | 2.72E-06 |
| NCAPH | 2.2 | 2.19E-04 |
| SPC25 | 2.2 | 6.50E-04 |
| FN1 | 2.21 | 9.56E-04 |
| STIL | 2.22 | 1.62E-06 |
| NCAPG | 2.23 | 7.57E-07 |
| ATAD2 | 2.23 | 3.39E-05 |
| PLK4 | 2.23 | 3.41E-03 |
| TDO2 | 2.24 | 1.06E-02 |
| ECE2 | 2.24 | 2.03E-04 |
| RAD54B | 2.25 | 1.04E-04 |
| SPP1 | 2.25 | 2.59E-02 |
| TNFSF13B | 2.26 | 5.35E-03 |
| MX1 | 2.27 | 9.16E-03 |
| CTXN1 | 2.3 | 2.58E-03 |
| CCNE1 | 2.3 | 3.60E-03 |
| OASL | 2.31 | 2.26E-02 |
| CXCL8 | 2.32 | 3.46E-02 |
| CDCA7 | 2.34 | 1.52E-02 |
| CDC45 | 2.35 | 5.92E-05 |
| MFAP2 | 2.35 | 3.55E-04 |
| DSCC1 | 2.35 | 1.02E-05 |
| HELLS | 2.35 | 7.26E-05 |
| FNDC1 | 2.36 | 5.11E-04 |
| NSD2 | 2.37 | 3.15E-09 |
| MYBL1 | 2.38 | 2.67E-02 |
| CDC25C | 2.38 | 4.01E-04 |
| PLAAT1 | 2.39 | 1.69E-02 |
| CDT1 | 2.39 | 1.40E-05 |
| CDC6 | 2.41 | 2.50E-04 |
| MYBL2 | 2.41 | 2.84E-07 |
| UBE2S | 2.41 | 3.07E-06 |
| AURKB | 2.42 | 2.03E-04 |
| KPNA2 | 2.44 | 1.35E-08 |
| CENPN | 2.45 | 1.17E-05 |
| SGO2 | 2.46 | 6.79E-06 |
| CCN4 | 2.48 | 5.83E-03 |
| CKAP2L | 2.48 | 4.67E-04 |
| CTPS1 | 2.5 | 2.14E-07 |
| PITX1 | 2.51 | 9.77E-03 |
| JPT1 | 2.51 | 6.94E-05 |
| OAS2 | 2.52 | 4.03E-03 |
| ZNF367 | 2.56 | 1.35E-06 |
| OIP5 | 2.56 | 5.99E-04 |
| MKI67 | 2.58 | 8.82E-07 |
| CDCA8 | 2.59 | 2.56E-06 |
| TYMS | 2.61 | 2.65E-05 |
| EPSTI1 | 2.64 | 2.91E-03 |
| ASF1B | 2.65 | 2.25E-06 |
| CDC7 | 2.65 | 2.05E-05 |
| RMI2 | 2.67 | 6.90E-07 |
| NMU | 2.69 | 1.30E-02 |
| LAMP3 | 2.69 | 9.09E-03 |
| HMGB3 | 2.74 | 8.78E-07 |
| TACC3 | 2.76 | 7.93E-11 |
| FANCI | 2.77 | 3.35E-07 |
| BRIP1 | 2.8 | 3.19E-04 |
| CCNA2 | 2.81 | 1.86E-08 |
| PIMREG | 2.81 | 1.66E-04 |
| LRRC15 | 2.82 | 6.77E-03 |
| MTFR2 | 2.87 | 8.46E-06 |
| NDC80 | 2.88 | 7.52E-05 |
| IQGAP3 | 2.9 | 3.48E-06 |
| RACGAP1 | 2.93 | 9.99E-10 |
| LMNB1 | 2.93 | 1.83E-06 |
| IFI44L | 2.94 | 1.39E-02 |
| BIRC5 | 3.01 | 1.31E-04 |
| ZWINT | 3.01 | 9.84E-09 |
| ECT2 | 3.03 | 1.46E-08 |
| HJURP | 3.05 | 4.70E-08 |
| RSAD2 | 3.05 | 2.19E-04 |
| KIF15 | 3.09 | 2.56E-06 |
| MMP9 | 3.12 | 1.48E-04 |
| SOX11 | 3.16 | 2.80E-02 |
| GAS2L3 | 3.17 | 1.21E-06 |
| RAD51AP1 | 3.17 | 1.06E-07 |
| CBX2 | 3.17 | 2.06E-04 |
| TRIP13 | 3.18 | 1.39E-07 |
| CTHRC1 | 3.18 | 2.32E-07 |
| CMPK2 | 3.2 | 4.40E-04 |
| DIAPH3 | 3.21 | 3.27E-07 |
| EZH2 | 3.22 | 3.15E-09 |
| KNL1 | 3.23 | 4.03E-08 |
| BLM | 3.23 | 2.91E-07 |
| GINS1 | 3.23 | 1.28E-07 |
| CDCA3 | 3.24 | 1.60E-07 |
| CENPE | 3.27 | 2.35E-05 |
| INHBA | 3.28 | 8.98E-06 |
| HMMR | 3.3 | 8.06E-09 |
| CENPU | 3.36 | 7.89E-07 |
| KIF11 | 3.36 | 4.56E-07 |
| GJB2 | 3.37 | 6.73E-05 |
| ISG15 | 3.37 | 6.76E-05 |
| APOBEC3B | 3.38 | 2.13E-04 |
| CCNB1 | 3.4 | 9.13E-08 |
| ADAMDEC1 | 3.49 | 1.54E-03 |
| CDCA5 | 3.53 | 7.47E-07 |
| CXCL11 | 3.56 | 2.85E-02 |
| DTL | 3.64 | 9.43E-11 |
| DLGAP5 | 3.65 | 5.87E-07 |
| KIF14 | 3.66 | 1.26E-08 |
| BUB1 | 3.67 | 1.10E-05 |
| CKS2 | 3.67 | 3.57E-11 |
| DEPDC1 | 3.69 | 7.89E-06 |
| PTTG1 | 3.71 | 9.26E-11 |
| KIF20A | 3.82 | 1.62E-06 |
| BUB1B | 3.83 | 2.10E-10 |
| CDKN3 | 3.84 | 2.67E-07 |
| MAD2L1 | 3.9 | 1.41E-09 |
| UHRF1 | 3.92 | 1.28E-08 |
| CXCL9 | 3.95 | 9.30E-03 |
| CDK1 | 4.02 | 7.93E-11 |
| PRC1 | 4.03 | 2.82E-11 |
| KIF2C | 4.04 | 3.02E-09 |
| CCNE2 | 4.06 | 3.03E-07 |
| FOXM1 | 4.07 | 1.60E-06 |
| AURKA | 4.08 | 2.78E-12 |
| UBE2C | 4.09 | 2.24E-11 |
| MMP11 | 4.11 | 5.98E-06 |
| NUF2 | 4.16 | 1.95E-09 |
| KIF4A | 4.17 | 3.15E-09 |
| UBE2T | 4.17 | 3.67E-09 |
| PCLAF | 4.18 | 2.89E-09 |
| CENPF | 4.2 | 4.74E-09 |
| FAM83D | 4.2 | 1.35E-07 |
| CEP55 | 4.25 | 1.85E-08 |
| CXCL10 | 4.29 | 4.05E-04 |
| MMP1 | 4.31 | 8.15E-04 |
| TTK | 4.32 | 1.66E-07 |
| CDC20 | 4.39 | 1.15E-06 |
| NUSAP1 | 4.41 | 4.83E-14 |
| CCNB2 | 4.52 | 2.74E-11 |
| PBK | 4.54 | 5.00E-08 |
| ANLN | 4.59 | 6.87E-09 |
| TPX2 | 4.69 | 2.29E-09 |
| NEK2 | 4.75 | 1.28E-12 |
| MELK | 5 | 1.03E-10 |
| RRM2 | 5 | 3.07E-13 |
| ASPM | 5.15 | 6.40E-11 |
| COL10A1 | 5.15 | 7.81E-07 |
| COL11A1 | 5.34 | 7.12E-06 |
| S100P | 5.41 | 8.06E-05 |
| TOP2A | 5.77 | 2.16E-12 |
