# Supplementary material for: Identification of hub genes and small molecule therapeutic drugs related to breast cancer with comprehensive bioinformatics analysis
Source: PeerJ. 2020 Sep 29;8:e9946. doi: 10.7717/peerj.9946 (PMC7556247; doi:10.7717/peerj.9946)
Supplement: Supplemental Information 11 [file peerj-08-9946-s011.docx]

| **ID** | **Log2FC** | **adj.P.Val** |
| --- | --- | --- |
| LEP | -7.46 | 1.13E-04 |
| TIMP4 | -6.94 | 3.25E-05 |
| GPD1 | -6.87 | 5.04E-05 |
| CIDEA | -6.75 | 5.78E-06 |
| PLIN4 | -6.63 | 5.04E-05 |
| ACVR1C | -6.61 | 9.21E-06 |
| RBP4 | -6.55 | 1.11E-04 |
| PLIN1 | -6.46 | 1.97E-04 |
| TRARG1 | -6.41 | 9.56E-07 |
| PCOLCE2 | -6.38 | 9.58E-05 |
| CIDEC | -6.10 | 5.80E-06 |
| S100B | -6.09 | 5.62E-06 |
| THRSP | -6.05 | 1.67E-03 |
| HSPB7 | -5.95 | 1.50E-05 |
| PCK1 | -5.78 | 4.08E-05 |
| ZBTB16 | -5.70 | 2.12E-05 |
| GPAM | -5.63 | 2.12E-05 |
| DEFB132 | -5.60 | 2.99E-05 |
| CD300LG | -5.58 | 2.91E-05 |
| LINC01697 | -5.53 | 3.19E-12 |
| TNMD | -5.48 | 4.40E-05 |
| MRAP | -5.46 | 7.77E-05 |
| ACACB | -5.43 | 2.12E-05 |
| GPIHBP1 | -5.40 | 2.16E-07 |
| LPL | -5.38 | 7.71E-05 |
| AKR1C1 | -5.28 | 1.97E-04 |
| C14orf180 | -5.27 | 9.52E-07 |
| ADH1B | -5.25 | 1.35E-03 |
| MAOA | -5.22 | 5.09E-05 |
| LGALS12 | -5.21 | 6.37E-04 |
| G0S2 | -5.20 | 4.43E-05 |
| SCARA5 | -5.09 | 3.33E-04 |
| AKR1C2 | -5.06 | 2.12E-05 |
| LIPE | -5.05 | 3.51E-05 |
| LYVE1 | -5.01 | 1.06E-04 |
| MME | -5.00 | 9.56E-07 |
| GYG2 | -5.00 | 3.34E-05 |
| CFD | -4.99 | 9.83E-06 |
| NTRK2 | -4.98 | 9.37E-05 |
| KCNIP2 | -4.97 | 4.27E-05 |
| CALB2 | -4.95 | 7.62E-05 |
| TF | -4.94 | 8.02E-03 |
| AGTR1 | -4.93 | 8.96E-04 |
| KLB | -4.92 | 5.23E-04 |
| CA3 | -4.91 | 1.03E-04 |
| PPP1R1A | -4.90 | 1.24E-03 |
| ECRG4 | -4.86 | 2.01E-03 |
| HSD11B1-AS1 | -4.86 | 1.95E-05 |
| KLHL31 | -4.82 | 6.95E-06 |
| CES1 | -4.82 | 3.17E-04 |
| DGAT2 | -4.78 | 4.93E-04 |
| ADRB1 | -4.76 | 4.50E-04 |
| PPARG | -4.74 | 1.60E-05 |
| ITGA7 | -4.74 | 5.04E-05 |
| AOC3 | -4.73 | 9.73E-06 |
| ITIH5 | -4.70 | 1.73E-03 |
| LOC101926960 | -4.69 | 7.47E-08 |
| LRRN4CL | -4.64 | 5.40E-04 |
| PCDH9 | -4.63 | 1.39E-06 |
| TMEM100 | -4.62 | 1.97E-04 |
| CRYAB | -4.61 | 5.66E-04 |
| ADH1C | -4.60 | 1.24E-06 |
| CAVIN2 | -4.59 | 3.04E-05 |
| SLC19A3 | -4.53 | 9.73E-06 |
| ATP1A2 | -4.50 | 1.74E-04 |
| CD36 | -4.50 | 1.47E-03 |
| SLC7A10 | -4.49 | 4.40E-05 |
| ADIPOQ | -4.47 | 1.90E-02 |
| BTNL9 | -4.47 | 2.12E-05 |
| ABCA8 | -4.46 | 1.98E-03 |
| HCAR3 | -4.45 | 1.66E-04 |
| HSPB6 | -4.44 | 1.70E-04 |
| CA4 | -4.44 | 1.02E-07 |
| TMEM132C | -4.44 | 1.71E-06 |
| COL6A6 | -4.44 | 1.81E-03 |
| GHR | -4.43 | 7.61E-05 |
| FGF2 | -4.40 | 7.40E-07 |
| GPC3 | -4.39 | 9.21E-06 |
| FIGN | -4.39 | 2.16E-07 |
| CHRDL1 | -4.35 | 2.49E-03 |
| FMO2 | -4.35 | 1.54E-06 |
| FXYD1 | -4.33 | 2.52E-04 |
| FABP4 | -4.31 | 2.03E-02 |
| LVRN | -4.29 | 1.80E-04 |
| KCNB1 | -4.28 | 3.25E-05 |
| CDO1 | -4.27 | 1.27E-04 |
| SPX | -4.21 | 1.93E-03 |
| HLF | -4.20 | 5.62E-07 |
| PTGER3 | -4.20 | 2.47E-04 |
| NIPSNAP3B | -4.19 | 5.06E-06 |
| HBB | -4.18 | 3.85E-04 |
| HOXA5 | -4.14 | 4.69E-05 |
| ANGPT1 | -4.10 | 2.70E-05 |
| ACADL | -4.10 | 2.39E-04 |
| ABCA6 | -4.10 | 9.89E-05 |
| RNF150 | -4.09 | 4.39E-06 |
| ADAMTS5 | -4.08 | 6.23E-04 |
| GPR146 | -4.07 | 3.64E-06 |
| GPX3 | -4.07 | 2.12E-05 |
| SLC4A4 | -4.02 | 2.86E-04 |
| PLAAT5 | -4.01 | 2.51E-04 |
| SGCG | -3.98 | 3.20E-04 |
| FHL1 | -3.95 | 2.27E-04 |
| AKR1C3 | -3.94 | 2.13E-04 |
| TPPP3 | -3.93 | 8.02E-05 |
| ASPA | -3.93 | 2.57E-04 |
| CCN5 | -3.92 | 5.45E-04 |
| GDF10 | -3.92 | 4.14E-05 |
| MAMDC2 | -3.91 | 1.73E-03 |
| IGFBP6 | -3.90 | 2.24E-04 |
| BHMT2 | -3.90 | 1.40E-05 |
| ABLIM3 | -3.89 | 3.01E-04 |
| SEMA3G | -3.87 | 2.88E-04 |
| MYOC | -3.85 | 5.86E-03 |
| TRHDE-AS1 | -3.85 | 1.08E-05 |
| TLN2 | -3.83 | 5.90E-06 |
| ABCA9 | -3.83 | 3.34E-05 |
| SOX5 | -3.82 | 5.16E-06 |
| PLAC9 | -3.78 | 5.86E-04 |
| PPP1R14A | -3.77 | 2.12E-05 |
| FAM107A | -3.77 | 2.12E-05 |
| ACSM5 | -3.75 | 9.21E-06 |
| VIT | -3.74 | 7.40E-07 |
| NRN1 | -3.69 | 1.52E-03 |
| HSPA12A | -3.67 | 4.62E-04 |
| MARCO | -3.67 | 2.43E-03 |
| AIFM2 | -3.67 | 2.53E-04 |
| 1-Mar | -3.64 | 1.43E-04 |
| MTURN | -3.64 | 3.19E-04 |
| CYP26B1 | -3.64 | 5.77E-05 |
| EBF1 | -3.63 | 4.78E-05 |
| RBP7 | -3.62 | 5.04E-05 |
| LINC01140 | -3.61 | 1.66E-03 |
| EBF3 | -3.61 | 3.82E-05 |
| GLDN | -3.60 | 7.77E-05 |
| IGSF10 | -3.60 | 2.17E-03 |
| TSLP | -3.57 | 3.69E-07 |
| PID1 | -3.56 | 1.48E-04 |
| SLC16A7 | -3.55 | 6.54E-05 |
| KLHL29 | -3.54 | 9.88E-04 |
| CORO2B | -3.53 | 6.49E-04 |
| PALMD | -3.52 | 1.97E-04 |
| MATN2 | -3.52 | 3.82E-03 |
| CSN1S1 | -3.52 | 3.85E-04 |
| RBPMS2 | -3.51 | 7.96E-04 |
| LMO3 | -3.51 | 3.65E-02 |
| TMEM37 | -3.51 | 1.45E-04 |
| GALNT15 | -3.51 | 9.21E-06 |
| CLSTN2 | -3.51 | 3.98E-02 |
| CAV1 | -3.48 | 7.27E-05 |
| NOVA1 | -3.48 | 4.45E-04 |
| NPY1R | -3.47 | 1.99E-02 |
| NPR3 | -3.46 | 2.46E-03 |
| FGFBP2 | -3.46 | 5.01E-04 |
| TGFBR3 | -3.45 | 1.66E-03 |
| CCDC3 | -3.43 | 5.78E-04 |
| COX7A1 | -3.42 | 2.16E-05 |
| ADIRF | -3.41 | 7.54E-04 |
| CCDC85A | -3.41 | 1.06E-06 |
| ARHGAP20 | -3.40 | 3.24E-05 |
| LIFR | -3.39 | 3.44E-05 |
| CREB5 | -3.39 | 3.85E-03 |
| ECM2 | -3.39 | 4.75E-05 |
| ZNF436-AS1 | -3.37 | 2.54E-05 |
| PDE2A | -3.36 | 1.33E-05 |
| CHL1 | -3.36 | 4.56E-04 |
| PDK4 | -3.34 | 1.29E-03 |
| CKMT2 | -3.33 | 4.40E-05 |
| OGN | -3.33 | 4.12E-02 |
| MFAP4 | -3.32 | 2.18E-02 |
| ELMOD3 | -3.31 | 1.97E-04 |
| HSPB2 | -3.31 | 7.78E-07 |
| AGPAT2 | -3.30 | 6.04E-05 |
| TMTC1 | -3.30 | 1.89E-04 |
| CLMP | -3.29 | 1.69E-04 |
| DMD | -3.29 | 6.60E-05 |
| PLAAT3 | -3.29 | 2.99E-04 |
| BMP2 | -3.28 | 2.47E-05 |
| CCDC178 | -3.28 | 4.92E-05 |
| ADAMTS9-AS2 | -3.27 | 3.98E-06 |
| TNS1 | -3.27 | 3.78E-05 |
| AKAP12 | -3.26 | 4.40E-04 |
| CYP4B1 | -3.26 | 3.15E-02 |
| XG | -3.24 | 5.06E-03 |
| SIK2 | -3.23 | 1.21E-05 |
| PRKAR2B | -3.22 | 3.85E-04 |
| TBX15 | -3.22 | 1.08E-05 |
| ECHDC3 | -3.22 | 1.89E-03 |
| CAV2 | -3.22 | 7.22E-05 |
| ALDOC | -3.21 | 3.11E-04 |
| PDE8B | -3.20 | 1.46E-02 |
| CRIM1-DT | -3.20 | 6.96E-04 |
| PAMR1 | -3.19 | 8.65E-03 |
| PRIMA1 | -3.18 | 1.54E-04 |
| NAT8L | -3.18 | 2.12E-05 |
| GSN | -3.17 | 1.78E-05 |
| SCN4B | -3.17 | 4.78E-05 |
| CPED1 | -3.17 | 2.54E-05 |
| PDZD2 | -3.17 | 2.19E-03 |
| CLDN5 | -3.16 | 9.21E-06 |
| ANK2 | -3.16 | 1.02E-02 |
| CEBPA | -3.15 | 2.88E-04 |
| MRAS | -3.15 | 6.69E-04 |
| APCDD1 | -3.14 | 3.85E-04 |
| SOD3 | -3.14 | 9.58E-05 |
| NPR1 | -3.13 | 2.84E-05 |
| ACKR1 | -3.12 | 3.54E-02 |
| PGM5 | -3.12 | 6.95E-06 |
| ANGPTL4 | -3.12 | 8.65E-03 |
| LARP6 | -3.11 | 1.31E-03 |
| ADRA2A | -3.11 | 5.26E-03 |
| DEPP1 | -3.11 | 4.39E-07 |
| MYMX | -3.09 | 2.64E-04 |
| SYNM | -3.09 | 2.69E-02 |
| ACO1 | -3.07 | 3.31E-04 |
| P2RY12 | -3.07 | 6.65E-04 |
| NMB | -3.07 | 1.19E-04 |
| TSPAN7 | -3.07 | 1.08E-02 |
| PRRG3 | -3.06 | 1.98E-03 |
| PRELP | -3.06 | 8.19E-05 |
| PFKFB3 | -3.05 | 2.12E-05 |
| NATD1 | -3.05 | 1.51E-04 |
| EBF2 | -3.04 | 2.43E-03 |
| RGMA | -3.04 | 2.13E-04 |
| ADGRD1 | -3.02 | 2.32E-03 |
| ATOH8 | -3.02 | 1.93E-05 |
| C6 | -3.01 | 1.01E-02 |
| TMEM178A | -3.01 | 2.49E-03 |
| DAAM2 | -3.01 | 3.98E-04 |
| TYRO3 | -3.01 | 3.22E-04 |
| MOCS1 | -3.00 | 1.13E-04 |
| FAM149A | -3.00 | 8.52E-03 |
| DDR2 | -2.99 | 2.35E-04 |
| LMOD1 | -2.98 | 7.92E-05 |
| PPL | -2.98 | 5.45E-04 |
| RERGL | -2.97 | 7.99E-03 |
| CACNA2D1 | -2.97 | 1.88E-03 |
| TWIST2 | -2.97 | 3.65E-03 |
| ANGPTL1 | -2.94 | 1.60E-03 |
| MT1M | -2.93 | 4.70E-04 |
| ANKRD29 | -2.92 | 1.95E-04 |
| PC | -2.92 | 1.97E-04 |
| PREX2 | -2.91 | 5.16E-06 |
| PYGL | -2.90 | 9.64E-04 |
| FGF14-AS2 | -2.90 | 5.76E-04 |
| CCDC69 | -2.89 | 2.48E-04 |
| SVEP1 | -2.89 | 1.90E-04 |
| EGFEM1P | -2.88 | 9.45E-05 |
| UST | -2.88 | 8.88E-04 |
| LINC00968 | -2.88 | 3.34E-04 |
| TMEM220 | -2.87 | 8.56E-06 |
| GNAI1 | -2.87 | 1.50E-04 |
| ZFPM2 | -2.86 | 2.50E-03 |
| KANK1 | -2.86 | 2.24E-04 |
| CDKN2C | -2.85 | 4.69E-05 |
| NMT2 | -2.84 | 2.69E-03 |
| GRK3 | -2.84 | 9.70E-05 |
| RBMS3 | -2.84 | 1.07E-04 |
| RDH5 | -2.83 | 7.81E-05 |
| MPPED2 | -2.83 | 1.70E-04 |
| GNG2 | -2.82 | 1.86E-04 |
| FZD4 | -2.82 | 4.98E-04 |
| CEP126 | -2.81 | 4.97E-03 |
| KANK3 | -2.81 | 2.70E-05 |
| IRS2 | -2.81 | 6.58E-04 |
| MLXIPL | -2.81 | 2.13E-04 |
| CLIC5 | -2.80 | 5.46E-05 |
| FBLN5 | -2.80 | 2.54E-04 |
| SCRN2 | -2.80 | 1.56E-05 |
| OXTR | -2.80 | 3.51E-02 |
| LTBP4 | -2.79 | 9.82E-06 |
| EHD2 | -2.79 | 9.68E-04 |
| LAMA3 | -2.78 | 8.05E-03 |
| HOXA7 | -2.78 | 2.12E-05 |
| MICU3 | -2.78 | 5.66E-04 |
| MEOX2 | -2.78 | 2.49E-03 |
| ACSS2 | -2.76 | 1.34E-04 |
| STX11 | -2.75 | 7.89E-04 |
| BOK | -2.74 | 3.81E-03 |
| ITSN1 | -2.74 | 1.87E-04 |
| MRGPRF | -2.74 | 1.01E-02 |
| ACSL1 | -2.74 | 5.45E-04 |
| TACR1 | -2.74 | 5.65E-05 |
| MCAM | -2.74 | 5.77E-05 |
| DENND2A | -2.73 | 2.27E-05 |
| FOSB | -2.73 | 2.79E-02 |
| PLEKHH2 | -2.73 | 8.18E-04 |
| BMP6 | -2.72 | 2.61E-04 |
| TNS2 | -2.71 | 4.77E-05 |
| CDKN1C | -2.71 | 7.70E-03 |
| AHNAK2 | -2.71 | 1.61E-02 |
| HOXA10 | -2.70 | 1.63E-02 |
| APOB | -2.70 | 2.28E-03 |
| TSHZ2 | -2.70 | 5.11E-03 |
| PRG4 | -2.70 | 1.47E-03 |
| CD34 | -2.69 | 2.05E-04 |
| RUNX1T1 | -2.69 | 2.03E-02 |
| RAPGEF3 | -2.69 | 1.34E-06 |
| SRPX | -2.69 | 3.26E-03 |
| AMOTL2 | -2.68 | 7.43E-06 |
| TEF | -2.68 | 8.18E-04 |
| MOB3B | -2.67 | 3.05E-03 |
| DPT | -2.67 | 6.56E-03 |
| MEOX1 | -2.67 | 6.93E-03 |
| KLF15 | -2.66 | 2.16E-07 |
| RGN | -2.66 | 4.27E-05 |
| IL17D | -2.65 | 3.41E-03 |
| SEMA6A | -2.64 | 3.12E-03 |
| SAMD4A | -2.64 | 6.78E-04 |
| WDFY3-AS2 | -2.64 | 8.89E-07 |
| PALM | -2.62 | 8.74E-04 |
| PLP1 | -2.61 | 1.69E-03 |
| MGLL | -2.60 | 2.53E-04 |
| MMD | -2.59 | 2.61E-04 |
| TLCD2 | -2.59 | 1.16E-03 |
| WNT11 | -2.58 | 2.20E-02 |
| FAM162B | -2.58 | 2.38E-04 |
| ID4 | -2.57 | 1.68E-02 |
| DSEL | -2.57 | 1.49E-02 |
| PYGM | -2.57 | 1.05E-03 |
| RNASE4 | -2.57 | 1.39E-03 |
| DLC1 | -2.57 | 1.17E-04 |
| PTPN21 | -2.57 | 5.33E-03 |
| SYNPO2 | -2.57 | 9.94E-04 |
| CCBE1 | -2.56 | 1.33E-05 |
| RARRES2 | -2.56 | 2.62E-04 |
| GALNT16 | -2.55 | 1.80E-04 |
| MESP1 | -2.55 | 1.03E-02 |
| NEGR1 | -2.55 | 4.88E-03 |
| FOXO1 | -2.55 | 4.80E-05 |
| CAPN6 | -2.55 | 3.80E-02 |
| KCNAB1 | -2.54 | 6.89E-03 |
| CPM | -2.52 | 6.19E-03 |
| ACKR4 | -2.52 | 7.39E-06 |
| C8orf88 | -2.52 | 2.36E-04 |
| CAVIN1 | -2.52 | 1.93E-04 |
| WASF3 | -2.52 | 1.84E-03 |
| ANKRD35 | -2.51 | 9.28E-04 |
| MYH11 | -2.51 | 1.33E-02 |
| GNG11 | -2.51 | 2.34E-05 |
| GIPC2 | -2.50 | 2.12E-05 |
| IGF1 | -2.50 | 7.12E-03 |
| NDRG2 | -2.50 | 2.28E-03 |
| KL | -2.49 | 2.48E-07 |
| ADAMTSL3 | -2.49 | 1.49E-03 |
| MYCT1 | -2.49 | 3.34E-05 |
| STAT5A | -2.49 | 1.56E-04 |
| F10 | -2.49 | 3.80E-04 |
| ADHFE1 | -2.49 | 3.13E-02 |
| CFH | -2.48 | 3.18E-04 |
| GNAL | -2.48 | 9.81E-05 |
| TCF7L1 | -2.48 | 1.42E-02 |
| ABCC6 | -2.48 | 1.01E-02 |
| PELI2 | -2.47 | 8.00E-04 |
| ALDH2 | -2.47 | 7.77E-05 |
| NDN | -2.47 | 5.58E-04 |
| PDZRN3 | -2.46 | 8.02E-03 |
| ACKR3 | -2.46 | 2.34E-03 |
| ANGPTL2 | -2.46 | 1.25E-03 |
| PLAGL1 | -2.46 | 9.49E-04 |
| RETSAT | -2.45 | 1.20E-03 |
| FRMD3 | -2.45 | 6.85E-04 |
| FAM13A | -2.45 | 4.25E-04 |
| FERMT2 | -2.45 | 3.33E-04 |
| CNR1 | -2.45 | 5.49E-03 |
| MIR100HG | -2.44 | 7.68E-03 |
| SPRY2 | -2.44 | 1.25E-03 |
| PPBP | -2.44 | 2.92E-02 |
| LDB2 | -2.43 | 1.22E-03 |
| PNPLA2 | -2.43 | 5.91E-05 |
| LINC00312 | -2.43 | 7.88E-04 |
| CYYR1 | -2.43 | 1.35E-03 |
| EEPD1 | -2.43 | 2.12E-05 |
| INMT | -2.43 | 5.50E-03 |
| CPEB1 | -2.43 | 5.92E-04 |
| SOBP | -2.42 | 1.38E-02 |
| AVPR1A | -2.42 | 1.98E-03 |
| DCUN1D3 | -2.42 | 4.76E-05 |
| EZH1 | -2.42 | 1.21E-05 |
| PLSCR4 | -2.42 | 6.56E-04 |
| NR3C2 | -2.42 | 8.60E-03 |
| CETP | -2.41 | 4.74E-03 |
| GYPC | -2.41 | 4.65E-05 |
| VGLL3 | -2.41 | 7.39E-04 |
| CCM2L | -2.41 | 1.97E-04 |
| NUDT7 | -2.41 | 7.54E-04 |
| ENPP2 | -2.41 | 7.62E-04 |
| ARHGAP6 | -2.41 | 1.57E-04 |
| CDC14B | -2.40 | 3.78E-04 |
| ADRB2 | -2.40 | 8.51E-04 |
| STBD1 | -2.39 | 2.31E-04 |
| P2RY14 | -2.39 | 1.15E-03 |
| PCSK5 | -2.39 | 4.45E-02 |
| VLDLR | -2.39 | 2.40E-02 |
| MYOM1 | -2.39 | 1.86E-04 |
| LOC102723493 | -2.39 | 6.58E-04 |
| PKDCC | -2.38 | 6.48E-03 |
| MEDAG | -2.38 | 1.13E-02 |
| ROBO4 | -2.38 | 5.91E-05 |
| OMD | -2.37 | 5.78E-04 |
| IRS1 | -2.37 | 1.93E-02 |
| PTPRB | -2.37 | 1.22E-04 |
| C19orf12 | -2.37 | 8.29E-05 |
| DMRT3 | -2.37 | 1.21E-04 |
| RSPO3 | -2.37 | 3.92E-02 |
| TCEAL7 | -2.37 | 1.52E-02 |
| CARMN | -2.37 | 1.81E-02 |
| ANO3 | -2.36 | 2.92E-03 |
| LRRN3 | -2.36 | 6.36E-03 |
| EHBP1 | -2.36 | 2.24E-04 |
| SYNPO | -2.36 | 1.49E-03 |
| FREM1 | -2.35 | 4.62E-04 |
| OSR1 | -2.35 | 2.67E-03 |
| RASGRF2 | -2.34 | 1.25E-03 |
| CD248 | -2.34 | 4.62E-03 |
| JAM2 | -2.33 | 6.35E-03 |
| AFAP1L1 | -2.33 | 2.96E-03 |
| ADGRL4 | -2.33 | 7.69E-05 |
| FADS3 | -2.33 | 1.54E-04 |
| BEND7 | -2.33 | 2.24E-02 |
| CFL2 | -2.33 | 2.52E-04 |
| PDE7B | -2.33 | 2.53E-02 |
| OLFM1 | -2.32 | 3.07E-02 |
| NOSTRIN | -2.32 | 2.50E-02 |
| CAMK1 | -2.32 | 6.83E-05 |
| SLC29A4 | -2.32 | 7.14E-03 |
| ADAMTS3 | -2.32 | 8.73E-03 |
| VWF | -2.32 | 1.52E-04 |
| RECK | -2.31 | 8.36E-04 |
| MEIS2 | -2.30 | 2.46E-02 |
| CDKN2B | -2.30 | 2.37E-02 |
| BIN1 | -2.30 | 1.03E-04 |
| CD209 | -2.30 | 6.02E-03 |
| ARHGEF28 | -2.29 | 8.29E-05 |
| CEP112 | -2.29 | 1.42E-04 |
| FAM13C | -2.29 | 2.03E-02 |
| CDON | -2.29 | 6.12E-03 |
| MYRIP | -2.29 | 1.06E-04 |
| ADM | -2.29 | 2.42E-03 |
| GDPD5 | -2.29 | 1.16E-02 |
| PDGFRL | -2.29 | 2.71E-02 |
| KCNJ2 | -2.29 | 2.32E-03 |
| LILRB5 | -2.29 | 6.47E-04 |
| ANXA1 | -2.28 | 3.51E-05 |
| NFIX | -2.28 | 1.19E-02 |
| TUB | -2.28 | 4.81E-02 |
| VKORC1L1 | -2.27 | 4.21E-04 |
| CSRNP3 | -2.27 | 2.88E-04 |
| EGFLAM | -2.27 | 1.21E-04 |
| LAMA4 | -2.27 | 1.24E-03 |
| PKD1L2 | -2.27 | 9.81E-04 |
| MSX1 | -2.27 | 2.25E-03 |
| HRCT1 | -2.26 | 5.53E-05 |
| RPL23AP32 | -2.26 | 2.12E-05 |
| HYMAI | -2.26 | 2.04E-03 |
| GPER1 | -2.26 | 4.57E-03 |
| DCLK1 | -2.26 | 3.82E-02 |
| PLXNA4 | -2.26 | 3.40E-05 |
| STOX1 | -2.25 | 1.29E-04 |
| SPTBN1 | -2.25 | 6.83E-06 |
| RHOJ | -2.25 | 1.50E-02 |
| ISM1 | -2.25 | 1.19E-02 |
| ALDH3A2 | -2.25 | 5.09E-05 |
| SHE | -2.24 | 2.61E-04 |
| ERG | -2.24 | 8.77E-04 |
| PTGIS | -2.24 | 2.44E-02 |
| NCAM1 | -2.24 | 1.63E-05 |
| NNAT | -2.24 | 5.60E-04 |
| CYBRD1 | -2.23 | 4.01E-04 |
| MAGI2-AS3 | -2.23 | 1.00E-03 |
| RASA3 | -2.23 | 1.03E-04 |
| EMX2 | -2.23 | 5.70E-04 |
| PRRT3-AS1 | -2.23 | 3.32E-02 |
| TLR3 | -2.23 | 1.65E-03 |
| CRYBG3 | -2.23 | 1.55E-03 |
| LINC00702 | -2.22 | 1.21E-03 |
| ADCY4 | -2.22 | 2.88E-04 |
| AQP1 | -2.22 | 2.87E-03 |
| HSDL2 | -2.22 | 6.80E-04 |
| TM4SF18 | -2.21 | 2.39E-03 |
| OSR2 | -2.21 | 1.13E-02 |
| FAM13A-AS1 | -2.21 | 6.88E-03 |
| CXCL12 | -2.21 | 8.15E-04 |
| LGI4 | -2.21 | 7.32E-04 |
| LIMS2 | -2.20 | 9.21E-06 |
| OLFML2A | -2.20 | 2.07E-05 |
| TENM3 | -2.20 | 4.90E-03 |
| SNX1 | -2.20 | 2.84E-04 |
| C1QTNF7 | -2.19 | 1.75E-02 |
| SH3BGRL2 | -2.19 | 3.40E-03 |
| FAXDC2 | -2.19 | 4.90E-05 |
| SH3D19 | -2.19 | 4.74E-04 |
| CDC42EP2 | -2.19 | 3.61E-04 |
| PODN | -2.19 | 7.66E-03 |
| RNASE1 | -2.18 | 1.12E-04 |
| TMEM47 | -2.18 | 8.77E-04 |
| SEL1L2 | -2.18 | 3.36E-02 |
| PCDH19 | -2.17 | 2.12E-05 |
| C1QTNF2 | -2.17 | 2.67E-02 |
| STARD13 | -2.17 | 5.66E-04 |
| LOXL4 | -2.17 | 2.01E-03 |
| PLIN5 | -2.17 | 2.15E-03 |
| EPDR1 | -2.17 | 9.29E-03 |
| FEZ1 | -2.17 | 7.13E-03 |
| GIMAP8 | -2.16 | 1.69E-04 |
| FMOD | -2.16 | 4.54E-02 |
| CLIP4 | -2.16 | 1.52E-03 |
| ACSS3 | -2.16 | 1.03E-02 |
| FNDC5 | -2.15 | 4.35E-04 |
| LHX6 | -2.15 | 5.59E-04 |
| ZNF677 | -2.15 | 4.30E-02 |
| CNN1 | -2.14 | 3.65E-02 |
| THSD7A | -2.14 | 3.47E-03 |
| EMX2OS | -2.14 | 8.76E-05 |
| HOGA1 | -2.14 | 2.41E-03 |
| MMRN2 | -2.14 | 1.96E-04 |
| NPY5R | -2.14 | 3.92E-02 |
| SLC35G2 | -2.14 | 8.99E-04 |
| MAML2 | -2.14 | 1.16E-03 |
| LRP5 | -2.13 | 3.34E-03 |
| EMCN | -2.13 | 7.66E-03 |
| PTPRM | -2.13 | 3.06E-04 |
| ABCD2 | -2.13 | 6.14E-04 |
| PCDH18 | -2.13 | 4.07E-03 |
| C1QTNF1 | -2.13 | 5.86E-04 |
| CACHD1 | -2.13 | 3.47E-02 |
| PTH1R | -2.12 | 8.89E-04 |
| MECOM | -2.12 | 3.31E-04 |
| PCNX1 | -2.12 | 1.21E-04 |
| MYL9 | -2.12 | 2.23E-04 |
| RBMS2 | -2.12 | 2.98E-04 |
| PLA2G4A | -2.12 | 1.22E-03 |
| CAT | -2.12 | 2.35E-05 |
| HSD11B1 | -2.12 | 2.92E-02 |
| NT5E | -2.12 | 2.35E-03 |
| ANG | -2.12 | 2.41E-03 |
| GAS1 | -2.11 | 4.70E-03 |
| THRB | -2.11 | 1.16E-02 |
| EPB42 | -2.11 | 1.76E-03 |
| SGK2 | -2.11 | 2.76E-03 |
| ARHGEF40 | -2.11 | 4.94E-04 |
| ZC3H12C | -2.11 | 1.95E-02 |
| PTGFR | -2.11 | 3.78E-02 |
| CNRIP1 | -2.10 | 2.96E-03 |
| GSTM5 | -2.10 | 6.76E-05 |
| LRP1 | -2.10 | 1.28E-03 |
| MYO16 | -2.10 | 2.92E-03 |
| ADAMTSL4 | -2.10 | 1.83E-03 |
| IL11RA | -2.10 | 4.01E-04 |
| ITM2A | -2.10 | 1.95E-02 |
| PLPP3 | -2.09 | 4.77E-05 |
| AADAC | -2.08 | 3.11E-02 |
| HSPA12B | -2.08 | 1.64E-03 |
| ZFHX4 | -2.08 | 6.27E-03 |
| MCOLN3 | -2.07 | 1.32E-02 |
| DMTN | -2.07 | 1.97E-03 |
| FGF1 | -2.07 | 2.36E-02 |
| LHFPL6 | -2.07 | 1.97E-03 |
| IRAK3 | -2.07 | 1.22E-02 |
| PPM1L | -2.07 | 2.79E-02 |
| CBX7 | -2.06 | 1.25E-03 |
| MEST | -2.06 | 2.22E-03 |
| HOXA4 | -2.06 | 2.55E-07 |
| ARHGEF6 | -2.06 | 5.18E-05 |
| SGCB | -2.06 | 6.79E-04 |
| ADGRF5 | -2.05 | 8.98E-05 |
| GPAT3 | -2.05 | 3.49E-02 |
| ADGRA2 | -2.05 | 3.17E-03 |
| PEAR1 | -2.05 | 2.77E-04 |
| HCG11 | -2.05 | 1.53E-03 |
| SUCNR1 | -2.05 | 3.44E-02 |
| ZNF423 | -2.05 | 1.02E-02 |
| OLFML1 | -2.04 | 5.11E-03 |
| LOC100286925 | -2.04 | 1.52E-03 |
| SAMD5 | -2.04 | 3.81E-02 |
| TMEM170B | -2.04 | 9.80E-03 |
| GULP1 | -2.04 | 7.54E-04 |
| LAMA2 | -2.04 | 3.00E-02 |
| DIAPH2 | -2.04 | 1.14E-03 |
| NDNF | -2.04 | 3.74E-02 |
| MSRB3 | -2.04 | 2.28E-03 |
| CASQ2 | -2.04 | 4.95E-03 |
| PDGFD | -2.03 | 7.54E-03 |
| SHANK3 | -2.03 | 4.01E-04 |
| ALDH1A1 | -2.02 | 1.80E-03 |
| FBLN2 | -2.02 | 7.16E-03 |
| TWIST1 | -2.02 | 1.05E-02 |
| ATP8B4 | -2.02 | 9.84E-04 |
| MAP3K20 | -2.02 | 2.54E-03 |
| ANTXR2 | -2.02 | 1.93E-04 |
| TMEM35A | -2.02 | 4.41E-03 |
| EDNRB | -2.02 | 8.54E-04 |
| OSBPL1A | -2.02 | 5.81E-04 |
| CYP2U1 | -2.01 | 4.03E-05 |
| PER3 | -2.01 | 1.73E-03 |
| STXBP1 | -2.01 | 1.84E-04 |
| ACADS | -2.01 | 2.61E-04 |
| S100A4 | -2.00 | 5.44E-04 |
| ZCCHC24 | -2.00 | 4.25E-03 |
| DOCK11 | -2.00 | 2.32E-03 |
| PRICKLE2 | -2.00 | 1.34E-02 |
| TGFBR2 | -2.00 | 2.72E-04 |
| SLC25A27 | -2.00 | 2.72E-03 |
| FZD5 | -2.00 | 3.17E-03 |
| DIXDC1 | -1.99 | 3.18E-04 |
| KLHL21 | -1.99 | 2.89E-03 |
| HINT3 | -1.99 | 7.35E-04 |
| GPBAR1 | -1.99 | 1.01E-04 |
| RNF157 | -1.98 | 2.11E-02 |
| C2CD2 | -1.98 | 6.29E-04 |
| TEAD1 | -1.98 | 9.22E-05 |
| ZNF204P | -1.98 | 2.91E-03 |
| COQ8A | -1.98 | 1.09E-04 |
| PGAP4 | -1.98 | 1.06E-06 |
| GLYAT | -1.97 | 4.04E-04 |
| FSTL3 | -1.97 | 7.65E-03 |
| FILIP1 | -1.97 | 5.35E-03 |
| GPRASP1 | -1.97 | 1.09E-04 |
| KLF9 | -1.97 | 2.05E-03 |
| RGL1 | -1.97 | 1.54E-04 |
| MID1 | -1.97 | 9.31E-03 |
| LDHD | -1.96 | 6.45E-03 |
| FAM126A | -1.96 | 1.57E-03 |
| FGD5 | -1.96 | 7.23E-04 |
| FHL5 | -1.96 | 7.13E-03 |
| MYEOV | -1.96 | 3.71E-04 |
| PI16 | -1.96 | 1.47E-03 |
| RAI2 | -1.96 | 9.43E-03 |
| RASIP1 | -1.95 | 6.37E-04 |
| NLGN1 | -1.95 | 1.54E-04 |
| MAP7D3 | -1.95 | 4.64E-04 |
| CTSG | -1.94 | 2.61E-02 |
| EXOC6B | -1.94 | 4.04E-04 |
| ID1 | -1.94 | 3.03E-02 |
| TPPP | -1.94 | 1.02E-02 |
| ST6GALNAC3 | -1.94 | 9.70E-04 |
| KLF4 | -1.93 | 6.62E-03 |
| PEMT | -1.93 | 1.79E-03 |
| AASS | -1.93 | 1.11E-04 |
| PAK3 | -1.92 | 3.62E-04 |
| ALPK3 | -1.92 | 4.11E-03 |
| F8 | -1.92 | 2.98E-03 |
| EHHADH | -1.92 | 9.54E-04 |
| STAT5B | -1.92 | 8.46E-05 |
| HSPB8 | -1.92 | 3.54E-02 |
| CLEC14A | -1.92 | 5.30E-05 |
| ARHGEF15 | -1.92 | 5.76E-04 |
| CMA1 | -1.91 | 3.82E-03 |
| SLITRK4 | -1.91 | 1.04E-03 |
| AVPI1 | -1.91 | 2.54E-04 |
| ABCB5 | -1.91 | 8.48E-03 |
| GABARAPL1 | -1.90 | 7.77E-05 |
| PLPP7 | -1.90 | 5.92E-04 |
| ABLIM1 | -1.90 | 7.54E-03 |
| ME1 | -1.89 | 2.01E-02 |
| SMIM3 | -1.89 | 2.47E-03 |
| ECSCR | -1.89 | 2.47E-03 |
| CYGB | -1.89 | 3.29E-05 |
| STAB1 | -1.88 | 3.18E-04 |
| KATNAL1 | -1.88 | 8.14E-04 |
| HPGDS | -1.88 | 1.60E-03 |
| IGFBP5 | -1.88 | 7.35E-03 |
| PDK2 | -1.87 | 2.83E-03 |
| ITGB1BP1 | -1.87 | 8.18E-04 |
| DTX1 | -1.87 | 5.77E-05 |
| ITGA1 | -1.87 | 2.12E-05 |
| ACAT1 | -1.87 | 3.19E-04 |
| FBXO17 | -1.87 | 3.27E-03 |
| FGD4 | -1.86 | 1.12E-03 |
| SOX7 | -1.86 | 2.03E-03 |
| GID4 | -1.86 | 1.08E-05 |
| NR3C1 | -1.86 | 7.77E-05 |
| POU6F1 | -1.85 | 3.45E-05 |
| C2orf88 | -1.85 | 5.54E-03 |
| STARD9 | -1.85 | 7.11E-04 |
| HIF3A | -1.85 | 6.88E-07 |
| EFEMP1 | -1.84 | 7.09E-03 |
| NAV3 | -1.84 | 2.48E-02 |
| LAMB2 | -1.84 | 2.13E-02 |
| HOXD8 | -1.83 | 4.03E-02 |
| SERPING1 | -1.83 | 7.69E-05 |
| COPZ2 | -1.83 | 1.68E-02 |
| EPAS1 | -1.83 | 4.20E-04 |
| C14orf28 | -1.83 | 7.21E-03 |
| ARHGEF4 | -1.83 | 3.56E-03 |
| PMP22 | -1.82 | 1.07E-03 |
| TEK | -1.82 | 4.27E-03 |
| FAH | -1.82 | 5.39E-03 |
| GPATCH11 | -1.82 | 3.75E-04 |
| GAS7 | -1.81 | 1.42E-02 |
| DHRS3 | -1.81 | 6.04E-04 |
| NUAK1 | -1.81 | 4.51E-03 |
| SSPN | -1.81 | 4.93E-03 |
| CD99L2 | -1.80 | 4.64E-04 |
| TIE1 | -1.80 | 1.45E-03 |
| CPXM2 | -1.80 | 3.10E-03 |
| CRIM1 | -1.80 | 7.00E-03 |
| GFOD1 | -1.79 | 1.93E-03 |
| COBLL1 | -1.79 | 2.85E-03 |
| TACC1 | -1.79 | 1.86E-04 |
| CASP17P | -1.78 | 1.42E-03 |
| PROCR | -1.78 | 3.85E-03 |
| DHDDS | -1.78 | 8.42E-04 |
| F13A1 | -1.78 | 1.51E-02 |
| PTCH1 | -1.78 | 4.92E-02 |
| TMOD2 | -1.77 | 1.89E-03 |
| CDH5 | -1.77 | 4.18E-04 |
| ECHDC1 | -1.77 | 3.86E-04 |
| LRRC34 | -1.77 | 3.37E-04 |
| SNX21 | -1.77 | 3.88E-03 |
| SNCG | -1.76 | 3.13E-03 |
| ZEB2 | -1.76 | 4.25E-03 |
| MAGI1 | -1.76 | 3.75E-03 |
| TMOD1 | -1.76 | 2.88E-04 |
| GRK5 | -1.76 | 5.66E-04 |
| ICAM2 | -1.76 | 2.24E-03 |
| FOXN3 | -1.76 | 1.06E-04 |
| SCN4A | -1.75 | 3.57E-04 |
| FOLR2 | -1.75 | 6.41E-03 |
| INHBB | -1.75 | 1.39E-02 |
| DNM1 | -1.75 | 1.39E-02 |
| CHST3 | -1.75 | 6.55E-03 |
| TRHDE | -1.75 | 1.55E-03 |
| C1orf115 | -1.75 | 4.99E-02 |
| SEMA6D | -1.75 | 2.27E-02 |
| RRAS2 | -1.75 | 3.10E-02 |
| DIPK1A | -1.75 | 3.12E-03 |
| KANK2 | -1.75 | 3.36E-03 |
| SLC66A1L | -1.74 | 4.74E-04 |
| DBP | -1.74 | 4.32E-03 |
| TTC28 | -1.74 | 4.94E-04 |
| LRRC2 | -1.74 | 1.52E-04 |
| PLA2R1 | -1.74 | 4.04E-02 |
| AKT3 | -1.74 | 6.80E-03 |
| ST6GALNAC6 | -1.73 | 9.44E-04 |
| RAMP2 | -1.73 | 1.35E-03 |
| KIRREL1 | -1.73 | 8.75E-03 |
| EMP1 | -1.73 | 3.60E-03 |
| SEPTIN4 | -1.73 | 2.28E-03 |
| HADH | -1.73 | 1.06E-04 |
| CHST7 | -1.72 | 4.23E-03 |
| GIMAP1 | -1.72 | 1.54E-03 |
| RGCC | -1.72 | 5.79E-04 |
| BMX | -1.72 | 3.85E-04 |
| CRY2 | -1.72 | 2.16E-04 |
| KCNJ8 | -1.72 | 2.80E-03 |
| PLEKHG6 | -1.72 | 1.05E-02 |
| METTL7A | -1.72 | 3.04E-04 |
| DPY19L2 | -1.71 | 2.46E-02 |
| RRAS | -1.71 | 3.17E-04 |
| ADAMTS1 | -1.71 | 1.17E-03 |
| EPB41L2 | -1.71 | 2.76E-03 |
| EGFL7 | -1.71 | 4.13E-03 |
| MET | -1.71 | 2.29E-03 |
| HRH1 | -1.70 | 4.02E-02 |
| ZEB1 | -1.70 | 3.02E-03 |
| HOTAIRM1 | -1.70 | 7.04E-03 |
| CYS1 | -1.70 | 1.53E-02 |
| PARD3B | -1.70 | 5.33E-03 |
| NEXN | -1.70 | 4.01E-03 |
| NR5A2 | -1.70 | 2.22E-04 |
| PLEKHM3 | -1.70 | 3.88E-03 |
| MRC1 | -1.70 | 1.67E-02 |
| LAMC1 | -1.69 | 5.16E-03 |
| PROS1 | -1.69 | 3.09E-02 |
| TFPI | -1.69 | 4.32E-02 |
| WBP4 | -1.69 | 7.41E-05 |
| SCN7A | -1.69 | 3.59E-03 |
| PTPN14 | -1.69 | 3.44E-02 |
| DNASE1L3 | -1.69 | 1.89E-02 |
| RPH3AL | -1.69 | 2.78E-02 |
| ROBO3 | -1.69 | 2.78E-05 |
| B4GALT6 | -1.69 | 3.15E-02 |
| EIF4EBP2 | -1.68 | 2.13E-03 |
| LATS2 | -1.68 | 7.43E-04 |
| ABCA5 | -1.68 | 1.73E-03 |
| ETFB | -1.68 | 4.83E-04 |
| SCN3A | -1.68 | 9.07E-03 |
| STON1 | -1.68 | 2.87E-02 |
| EPHX1 | -1.68 | 9.91E-03 |
| PCYOX1 | -1.68 | 3.04E-05 |
| KLHDC8B | -1.67 | 1.59E-03 |
| FRMD4A | -1.67 | 2.42E-03 |
| GBE1 | -1.67 | 2.48E-03 |
| SCARB1 | -1.67 | 4.13E-03 |
| PRR5 | -1.67 | 6.15E-03 |
| GAB2 | -1.66 | 3.51E-03 |
| FXYD6 | -1.66 | 4.96E-02 |
| TXNIP | -1.66 | 2.58E-04 |
| SLIT3 | -1.66 | 2.72E-03 |
| PDE5A | -1.66 | 4.72E-02 |
| TTC7B | -1.66 | 5.50E-03 |
| AHNAK | -1.66 | 1.01E-04 |
| AOC2 | -1.66 | 6.93E-03 |
| SMYD4 | -1.65 | 4.03E-04 |
| TMEM120B | -1.65 | 1.59E-03 |
| MXRA7 | -1.65 | 3.04E-03 |
| HBD | -1.65 | 1.16E-02 |
| TRPC1 | -1.65 | 1.54E-02 |
| ADGRL2 | -1.64 | 1.30E-02 |
| EPB41L1 | -1.64 | 1.77E-03 |
| TTC23 | -1.64 | 2.22E-04 |
| EMILIN2 | -1.64 | 1.56E-02 |
| ZNF106 | -1.64 | 4.70E-03 |
| SAV1 | -1.64 | 3.10E-04 |
| MITF | -1.63 | 1.70E-03 |
| ANO6 | -1.63 | 2.43E-04 |
| FABP5 | -1.63 | 3.54E-02 |
| PDGFRA | -1.63 | 1.53E-03 |
| SERPINF1 | -1.63 | 6.88E-03 |
| NR1H3 | -1.63 | 1.20E-02 |
| GGTA1P | -1.63 | 1.56E-03 |
| TNNT3 | -1.63 | 6.70E-03 |
| GPR34 | -1.63 | 4.20E-04 |
| RNF180 | -1.63 | 8.47E-04 |
| EVC2 | -1.63 | 1.29E-04 |
| CRHBP | -1.62 | 2.10E-03 |
| VEGFB | -1.62 | 8.20E-04 |
| P3H2 | -1.62 | 1.04E-04 |
| TIMP3 | -1.62 | 1.59E-03 |
| CELF2 | -1.62 | 1.80E-03 |
| PIGZ | -1.62 | 1.10E-02 |
| JHY | -1.62 | 2.13E-02 |
| NOTCH4 | -1.62 | 3.30E-04 |
| SMOC2 | -1.62 | 3.19E-03 |
| CTNNAL1 | -1.61 | 1.24E-03 |
| DCN | -1.61 | 2.11E-03 |
| FHOD3 | -1.61 | 4.15E-02 |
| JAZF1 | -1.61 | 4.05E-04 |
| GPHN | -1.61 | 2.83E-03 |
| TKT | -1.61 | 1.04E-03 |
| ZNF471 | -1.61 | 6.52E-04 |
| SNTB2 | -1.60 | 1.42E-04 |
| EPB41L4B | -1.60 | 6.74E-03 |
| ABHD15 | -1.60 | 7.38E-03 |
| NR2F2-AS1 | -1.60 | 3.81E-02 |
| RHOXF1 | -1.60 | 1.31E-02 |
| TCF4 | -1.60 | 4.03E-03 |
| SLC9A9 | -1.60 | 9.72E-04 |
| GAS6 | -1.60 | 1.16E-02 |
| NPR2 | -1.59 | 1.97E-04 |
| KAT2B | -1.59 | 1.92E-05 |
| ARHGEF10 | -1.59 | 1.76E-03 |
| ANKDD1A | -1.59 | 7.66E-03 |
| HIC1 | -1.59 | 1.75E-02 |
| MAF | -1.58 | 8.74E-04 |
| IGIP | -1.58 | 1.55E-03 |
| PLPP1 | -1.58 | 5.58E-03 |
| GIMAP6 | -1.58 | 2.27E-03 |
| KCND2 | -1.57 | 6.96E-04 |
| SMAD6 | -1.57 | 1.59E-03 |
| LIAS | -1.57 | 9.12E-04 |
| ABCC9 | -1.57 | 1.02E-02 |
| SLC27A1 | -1.57 | 5.75E-04 |
| VSTM4 | -1.57 | 1.07E-03 |
| UGP2 | -1.57 | 1.96E-04 |
| ADIPOR2 | -1.57 | 2.07E-02 |
| SPART | -1.56 | 1.07E-03 |
| EPHX2 | -1.56 | 4.58E-03 |
| LRRC8C | -1.56 | 1.44E-03 |
| ARHGAP21 | -1.56 | 8.18E-04 |
| YAP1 | -1.56 | 2.45E-03 |
| KLHDC1 | -1.56 | 1.92E-04 |
| CAP2 | -1.55 | 2.66E-02 |
| TSPAN4 | -1.55 | 9.94E-05 |
| RASSF9 | -1.55 | 2.19E-02 |
| PLEKHF1 | -1.55 | 7.60E-05 |
| FGF7 | -1.55 | 2.91E-03 |
| DPYSL2 | -1.55 | 2.54E-03 |
| PPARA | -1.54 | 1.56E-02 |
| CEP68 | -1.54 | 6.09E-05 |
| SASH1 | -1.54 | 2.62E-04 |
| TRIM52-AS1 | -1.54 | 7.23E-04 |
| PDP2 | -1.54 | 6.91E-03 |
| ABTB1 | -1.54 | 5.31E-05 |
| RFTN1 | -1.54 | 8.22E-03 |
| ANGPTL8 | -1.54 | 2.35E-02 |
| ZHX3 | -1.53 | 9.32E-03 |
| AIF1L | -1.53 | 4.81E-02 |
| CAVIN3 | -1.53 | 2.34E-02 |
| EDN1 | -1.53 | 1.25E-02 |
| SPARCL1 | -1.53 | 2.16E-02 |
| PGM1 | -1.52 | 9.93E-03 |
| ESAM | -1.52 | 2.64E-03 |
| DOCK6 | -1.52 | 8.88E-04 |
| GPLD1 | -1.52 | 4.33E-03 |
| ALDH6A1 | -1.52 | 1.24E-03 |
| UTRN | -1.51 | 2.79E-05 |
| GKAP1 | -1.51 | 7.89E-03 |
| PYROXD2 | -1.51 | 4.41E-03 |
| PPP2R1B | -1.51 | 2.64E-02 |
| PCGF5 | -1.50 | 9.53E-05 |
| TCF7L2 | -1.50 | 2.44E-02 |
| FAT4 | -1.50 | 4.91E-02 |
| PHLDB2 | -1.50 | 3.91E-02 |
| AMOTL1 | -1.50 | 1.26E-02 |
| BCL2L2 | -1.49 | 5.68E-03 |
| PHYHD1 | -1.49 | 4.29E-03 |
| TBC1D2B | -1.49 | 1.55E-03 |
| MOB3C | -1.49 | 3.24E-05 |
| RBPMS | -1.49 | 2.64E-03 |
| TRIP10 | -1.49 | 1.44E-03 |
| DPP4 | -1.49 | 3.09E-02 |
| CLEC1A | -1.48 | 3.08E-03 |
| LOC101928000 | -1.48 | 6.20E-03 |
| FGFR1 | -1.48 | 2.89E-03 |
| DIP2C | -1.48 | 2.01E-03 |
| TNIP1 | -1.48 | 1.89E-04 |
| PDZRN3-AS1 | -1.47 | 1.64E-03 |
| DAB2 | -1.47 | 3.56E-03 |
| JADE1 | -1.47 | 1.12E-03 |
| GLIDR | -1.47 | 2.47E-02 |
| NECTIN3 | -1.47 | 2.36E-03 |
| CTSF | -1.47 | 2.11E-02 |
| MDFIC | -1.47 | 2.14E-03 |
| TSPAN18 | -1.47 | 1.82E-02 |
| KCTD12 | -1.47 | 9.81E-04 |
| TMEM273 | -1.46 | 1.34E-02 |
| TTBK2 | -1.46 | 1.30E-03 |
| CUTC | -1.46 | 6.34E-04 |
| ABCA1 | -1.46 | 5.40E-04 |
| NR1D2 | -1.46 | 1.11E-02 |
| HOXD4 | -1.46 | 1.98E-02 |
| HYAL1 | -1.46 | 2.41E-03 |
| SNX33 | -1.45 | 1.68E-04 |
| TMEM140 | -1.45 | 1.47E-03 |
| ZBTB20 | -1.45 | 3.37E-03 |
| ME3 | -1.45 | 8.51E-03 |
| TK2 | -1.44 | 1.41E-03 |
| LOC100506388 | -1.44 | 1.83E-03 |
| ST3GAL3 | -1.44 | 9.77E-04 |
| OPTN | -1.43 | 1.60E-02 |
| ALDH1A3 | -1.43 | 2.52E-02 |
| SLC29A1 | -1.43 | 8.03E-03 |
| MAP4 | -1.43 | 2.90E-04 |
| PECAM1 | -1.43 | 2.71E-02 |
| TSPAN3 | -1.43 | 1.32E-02 |
| LMO2 | -1.43 | 9.78E-04 |
| FOXN2 | -1.43 | 5.26E-03 |
| BST1 | -1.42 | 2.40E-02 |
| ARHGAP24 | -1.42 | 4.17E-03 |
| EML3 | -1.42 | 2.40E-03 |
| FAM228B | -1.42 | 1.00E-02 |
| RHOQ | -1.42 | 1.76E-03 |
| PLEKHA4 | -1.42 | 4.22E-02 |
| MYOCD | -1.42 | 1.36E-02 |
| PPP1R16B | -1.42 | 4.66E-02 |
| CARD6 | -1.42 | 6.05E-03 |
| PLCL2 | -1.42 | 2.29E-03 |
| ZBTB4 | -1.42 | 6.13E-04 |
| BCL6 | -1.41 | 4.40E-04 |
| SGCE | -1.41 | 1.30E-02 |
| MAP1LC3C | -1.41 | 1.09E-07 |
| PPP1R12B | -1.41 | 1.16E-03 |
| VSIR | -1.41 | 2.64E-04 |
| CALCOCO1 | -1.41 | 2.13E-04 |
| FSTL1 | -1.41 | 2.07E-02 |
| HDAC4 | -1.41 | 1.43E-02 |
| PIP4P2 | -1.40 | 7.91E-03 |
| L3HYPDH | -1.40 | 1.19E-02 |
| PAM | -1.40 | 8.80E-03 |
| TUBB6 | -1.40 | 1.99E-02 |
| AAMDC | -1.40 | 1.29E-03 |
| BLCAP | -1.40 | 1.13E-04 |
| RUNDC3B | -1.39 | 1.17E-02 |
| CCDC82 | -1.39 | 4.04E-04 |
| GSTM4 | -1.39 | 4.92E-02 |
| SDC2 | -1.39 | 2.14E-02 |
| TFE3 | -1.39 | 2.70E-04 |
| TMEM88 | -1.39 | 2.11E-03 |
| TUBG2 | -1.38 | 5.25E-03 |
| MTHFD1 | -1.38 | 2.12E-05 |
| CAPS2 | -1.38 | 3.23E-03 |
| RNF125 | -1.38 | 1.49E-02 |
| ZNF300P1 | -1.38 | 4.50E-04 |
| KIAA1107 | -1.38 | 3.34E-05 |
| PLTP | -1.38 | 5.68E-03 |
| ARHGAP31 | -1.38 | 6.79E-04 |
| PKD2 | -1.38 | 4.67E-03 |
| TLE1 | -1.37 | 2.76E-02 |
| ZNF395 | -1.37 | 1.56E-03 |
| PGM5-AS1 | -1.37 | 2.48E-02 |
| F3 | -1.37 | 2.88E-02 |
| TMEM245 | -1.37 | 1.21E-03 |
| MT1X | -1.37 | 1.36E-02 |
| APBB1IP | -1.36 | 2.64E-02 |
| LOC729970 | -1.36 | 6.18E-03 |
| DDAH2 | -1.36 | 5.63E-03 |
| KLF11 | -1.36 | 8.22E-03 |
| NFIB | -1.36 | 1.21E-02 |
| CSAD | -1.36 | 6.80E-04 |
| ABCA11P | -1.36 | 7.22E-03 |
| SOX17 | -1.36 | 6.87E-03 |
| LINC00526 | -1.36 | 8.74E-03 |
| KLHL3 | -1.36 | 2.39E-03 |
| LDHB | -1.35 | 4.10E-03 |
| SATB2 | -1.35 | 3.84E-02 |
| MXRA8 | -1.35 | 1.32E-02 |
| LNPEP | -1.35 | 6.13E-04 |
| ADCY6 | -1.35 | 1.52E-03 |
| DMRT2 | -1.35 | 2.46E-04 |
| WDR86 | -1.35 | 1.52E-03 |
| FAM184A | -1.35 | 5.89E-03 |
| PTEN | -1.34 | 8.43E-05 |
| FGF7P3 | -1.34 | 7.25E-04 |
| TMEM120A | -1.34 | 8.83E-03 |
| RILP | -1.34 | 2.77E-03 |
| RAB11B-AS1 | -1.34 | 8.89E-04 |
| ADAMTS9 | -1.34 | 9.50E-03 |
| PEAK1 | -1.34 | 2.95E-03 |
| EPM2A | -1.34 | 8.48E-03 |
| FCGRT | -1.34 | 1.51E-02 |
| SH3RF3 | -1.34 | 1.87E-03 |
| INPP5K | -1.34 | 3.68E-04 |
| ACAA2 | -1.34 | 1.13E-02 |
| LRFN5 | -1.34 | 1.81E-03 |
| ESYT1 | -1.33 | 8.59E-04 |
| TESC | -1.33 | 1.30E-02 |
| CADM3 | -1.33 | 7.25E-03 |
| EOGT | -1.33 | 3.35E-02 |
| VPS53 | -1.33 | 1.42E-03 |
| ATP2B4 | -1.33 | 4.99E-03 |
| GIMAP7 | -1.33 | 4.64E-02 |
| ITPK1 | -1.32 | 3.08E-03 |
| FYN | -1.32 | 7.93E-03 |
| KBTBD11 | -1.32 | 1.47E-03 |
| DYNC2H1 | -1.32 | 5.05E-03 |
| PPP1R36 | -1.32 | 1.55E-02 |
| CLYBL | -1.32 | 9.83E-03 |
| NRP1 | -1.32 | 2.36E-02 |
| SYDE1 | -1.32 | 5.94E-04 |
| AMT | -1.32 | 1.08E-03 |
| BOLA3-AS1 | -1.31 | 5.77E-05 |
| SAP30L | -1.31 | 5.68E-03 |
| SKI | -1.31 | 2.61E-04 |
| MEIS3P1 | -1.31 | 2.73E-03 |
| PDGFC | -1.31 | 4.15E-04 |
| FBXO31 | -1.31 | 8.51E-05 |
| SLC1A3 | -1.31 | 3.62E-02 |
| MPDZ | -1.31 | 1.48E-03 |
| MBNL1-AS1 | -1.31 | 2.29E-02 |
| INPP1 | -1.31 | 2.11E-02 |
| MAPK10 | -1.30 | 4.65E-02 |
| SIDT2 | -1.30 | 3.11E-04 |
| MAN1A1 | -1.30 | 2.05E-02 |
| TBX5 | -1.30 | 2.94E-02 |
| ABHD14B | -1.30 | 1.24E-02 |
| ZNF502 | -1.30 | 2.15E-02 |
| NIFK-AS1 | -1.30 | 8.29E-05 |
| HSPG2 | -1.30 | 1.14E-02 |
| JRKL | -1.30 | 1.96E-02 |
| ZNF438 | -1.29 | 1.50E-03 |
| FLT1 | -1.29 | 1.16E-02 |
| SPSB1 | -1.29 | 2.97E-02 |
| ARHGAP42 | -1.29 | 7.41E-03 |
| ETFDH | -1.29 | 5.35E-03 |
| COLCA2 | -1.29 | 1.43E-02 |
| BNIP3L | -1.29 | 7.89E-04 |
| NAB1 | -1.29 | 1.61E-02 |
| NLRP1 | -1.28 | 6.67E-04 |
| PARVA | -1.28 | 6.63E-03 |
| THSD7B | -1.28 | 5.96E-05 |
| PKN3 | -1.28 | 1.25E-02 |
| TLN1 | -1.28 | 2.11E-03 |
| HACD2 | -1.28 | 1.25E-02 |
| CLEC4GP1 | -1.28 | 3.44E-05 |
| RBPMS-AS1 | -1.27 | 1.72E-03 |
| ESD | -1.27 | 6.35E-04 |
| CSF1 | -1.27 | 3.51E-05 |
| PHLDA3 | -1.27 | 1.87E-02 |
| MTMR10 | -1.27 | 9.02E-04 |
| FAM43A | -1.27 | 2.27E-02 |
| STX7 | -1.27 | 4.20E-04 |
| TAF8 | -1.27 | 1.44E-03 |
| CREBRF | -1.26 | 2.47E-04 |
| WTIP | -1.26 | 2.42E-02 |
| CMKLR1 | -1.26 | 2.85E-02 |
| ACADVL | -1.26 | 1.21E-03 |
| RGS3 | -1.26 | 1.46E-02 |
| ADD3 | -1.26 | 2.17E-02 |
| SBF2 | -1.26 | 9.56E-04 |
| PLCE1 | -1.26 | 2.21E-02 |
| TLR4 | -1.26 | 2.46E-02 |
| LETMD1 | -1.26 | 6.90E-05 |
| NAP1L5 | -1.25 | 3.02E-02 |
| NYNRIN | -1.25 | 1.32E-02 |
| VIPR1 | -1.25 | 1.28E-02 |
| NACC2 | -1.25 | 4.57E-02 |
| GPX4 | -1.25 | 2.15E-03 |
| SMG6 | -1.25 | 6.09E-04 |
| YBX3 | -1.25 | 1.87E-02 |
| TSC22D3 | -1.25 | 1.21E-02 |
| DHRS12 | -1.25 | 4.87E-03 |
| MYCBP2 | -1.25 | 1.16E-03 |
| HCFC2 | -1.25 | 6.04E-04 |
| LIX1L | -1.25 | 6.96E-03 |
| PRCD | -1.25 | 3.34E-04 |
| EPB41L4A-AS1 | -1.24 | 1.66E-02 |
| PCCA | -1.24 | 5.46E-04 |
| TFDP2 | -1.24 | 3.76E-02 |
| LRRTM2 | -1.24 | 5.45E-04 |
| ST3GAL6 | -1.24 | 3.53E-02 |
| SESTD1 | -1.24 | 6.36E-03 |
| SNX9 | -1.24 | 9.79E-04 |
| ETS2 | -1.24 | 1.63E-02 |
| WNT2B | -1.24 | 2.94E-02 |
| RORA | -1.24 | 7.34E-03 |
| PPP3CB | -1.23 | 4.52E-04 |
| ZBED3 | -1.23 | 2.35E-03 |
| CABLES1 | -1.23 | 1.48E-03 |
| QKI | -1.22 | 1.30E-02 |
| ALAD | -1.22 | 6.16E-05 |
| CYTH3 | -1.22 | 8.08E-03 |
| MYADM | -1.22 | 2.87E-03 |
| OAF | -1.22 | 1.48E-02 |
| FAM110D | -1.22 | 9.66E-03 |
| PJA1 | -1.22 | 6.93E-04 |
| SELENBP1 | -1.22 | 4.22E-03 |
| PRRG1 | -1.22 | 4.48E-02 |
| HACL1 | -1.22 | 1.14E-02 |
| ATXN1L | -1.21 | 3.75E-04 |
| ARHGEF17 | -1.21 | 3.00E-02 |
| S1PR1 | -1.21 | 3.72E-03 |
| FLNC | -1.21 | 9.31E-03 |
| ARHGAP5 | -1.21 | 8.22E-04 |
| SPAAR | -1.21 | 1.35E-03 |
| DUSP3 | -1.21 | 4.01E-04 |
| WASF1 | -1.21 | 4.05E-02 |
| TIMP2 | -1.21 | 2.92E-02 |
| BTBD6 | -1.21 | 4.07E-04 |
| TMEM135 | -1.21 | 3.91E-02 |
| FLJ20021 | -1.21 | 9.37E-05 |
| PHYHIP | -1.20 | 5.43E-03 |
| LOC286437 | -1.20 | 1.20E-04 |
| RXRA | -1.20 | 2.47E-03 |
| ZNF541 | -1.20 | 2.02E-02 |
| LTC4S | -1.20 | 5.76E-04 |
| NEURL1B | -1.19 | 1.94E-02 |
| OGFRL1 | -1.19 | 3.00E-02 |
| SKAP2 | -1.19 | 9.57E-03 |
| CERT1 | -1.19 | 5.68E-03 |
| ARL2 | -1.19 | 9.64E-04 |
| SCAI | -1.19 | 2.33E-03 |
| PDGFRB | -1.19 | 1.52E-02 |
| PIK3R1 | -1.19 | 1.84E-02 |
| FAM172A | -1.18 | 9.28E-03 |
| DAPK2 | -1.18 | 1.01E-02 |
| SCN1B | -1.18 | 4.13E-03 |
| HIPK3 | -1.18 | 4.32E-03 |
| C1R | -1.18 | 3.16E-02 |
| CC2D2A | -1.18 | 5.39E-03 |
| DMGDH | -1.18 | 6.76E-03 |
| MAP4K3-DT | -1.18 | 1.34E-02 |
| TINAGL1 | -1.18 | 1.79E-03 |
| XPNPEP2 | -1.18 | 1.41E-02 |
| FER | -1.18 | 2.47E-03 |
| MMP28 | -1.18 | 1.11E-03 |
| TNFRSF21 | -1.18 | 4.01E-02 |
| SPATA7 | -1.18 | 4.06E-02 |
| FGFRL1 | -1.17 | 6.73E-03 |
| WASF2 | -1.17 | 2.85E-02 |
| N4BP2L1 | -1.17 | 5.84E-03 |
| TRIM23 | -1.17 | 1.43E-02 |
| STARD8 | -1.17 | 2.88E-04 |
| EFEMP2 | -1.17 | 4.04E-02 |
| CHCHD10 | -1.17 | 1.68E-03 |
| PPP1R15A | -1.17 | 4.71E-03 |
| ACBD4 | -1.16 | 4.19E-04 |
| SLC35G1 | -1.16 | 3.86E-02 |
| JCAD | -1.16 | 4.01E-02 |
| ANKRD40 | -1.16 | 3.65E-02 |
| ELOVL5 | -1.16 | 9.30E-03 |
| MYO15B | -1.16 | 2.05E-04 |
| PXDC1 | -1.16 | 2.48E-02 |
| FAM228A | -1.16 | 2.17E-04 |
| TBC1D13 | -1.16 | 1.02E-03 |
| ZFP36L2 | -1.16 | 2.82E-03 |
| MYL5 | -1.16 | 2.44E-02 |
| DNAJB4 | -1.16 | 6.02E-03 |
| DENND5B | -1.16 | 2.25E-02 |
| CDH13 | -1.16 | 5.54E-03 |
| A2M | -1.15 | 2.51E-03 |
| GTDC1 | -1.15 | 3.12E-03 |
| TMEM42 | -1.15 | 1.23E-03 |
| MAP7D1 | -1.15 | 5.43E-03 |
| 2-Mar | -1.15 | 6.63E-03 |
| COL15A1 | -1.14 | 1.77E-02 |
| SUCLA2 | -1.14 | 3.93E-04 |
| HOOK2 | -1.14 | 2.66E-03 |
| DCHS1 | -1.14 | 4.17E-02 |
| TESK1 | -1.14 | 2.09E-03 |
| CSPG4 | -1.14 | 6.40E-03 |
| STRADB | -1.14 | 4.97E-02 |
| CEBPD | -1.14 | 8.08E-03 |
| PDE3A | -1.14 | 2.41E-02 |
| MID2 | -1.14 | 1.64E-03 |
| AK3 | -1.14 | 1.63E-03 |
| STX8 | -1.14 | 1.86E-04 |
| SIK3 | -1.13 | 1.67E-02 |
| SAMD8 | -1.13 | 1.86E-02 |
| AXL | -1.13 | 5.65E-03 |
| PPM1F | -1.13 | 2.00E-02 |
| WDR36 | -1.13 | 4.00E-03 |
| LOC105379426 | -1.13 | 3.78E-05 |
| ACVRL1 | -1.13 | 1.78E-02 |
| FMNL2 | -1.13 | 2.36E-02 |
| LGALS3 | -1.13 | 6.13E-03 |
| CPQ | -1.13 | 2.24E-02 |
| SH3BP5 | -1.13 | 2.27E-04 |
| SDK1 | -1.12 | 6.41E-03 |
| NACAD | -1.12 | 2.35E-04 |
| COL4A2 | -1.12 | 6.20E-03 |
| PINK1 | -1.12 | 4.27E-04 |
| MBNL1 | -1.12 | 2.47E-03 |
| IL6ST | -1.12 | 3.44E-02 |
| NMNAT3 | -1.12 | 3.65E-02 |
| ARHGEF7 | -1.12 | 5.10E-04 |
| SIM1 | -1.12 | 2.96E-02 |
| ACE | -1.12 | 2.76E-02 |
| VAT1 | -1.12 | 2.75E-02 |
| KLF7 | -1.11 | 1.63E-02 |
| CASTOR3 | -1.11 | 5.55E-03 |
| ACAP1 | -1.11 | 1.73E-03 |
| SCN9A | -1.11 | 3.88E-04 |
| SPRY1 | -1.11 | 2.88E-02 |
| SH3KBP1 | -1.11 | 8.75E-03 |
| PCDH12 | -1.11 | 5.84E-03 |
| NPHP3 | -1.11 | 1.18E-02 |
| SIRPA | -1.11 | 1.81E-02 |
| SLC27A3 | -1.11 | 9.77E-03 |
| B4GAT1 | -1.11 | 6.19E-04 |
| TMEM204 | -1.11 | 2.74E-03 |
| TMEM198B | -1.10 | 1.17E-03 |
| MAN2C1 | -1.10 | 7.40E-04 |
| DENND2C | -1.10 | 2.47E-02 |
| VAMP3 | -1.10 | 4.73E-04 |
| DST | -1.10 | 1.47E-02 |
| LMCD1 | -1.10 | 3.19E-02 |
| APBA1 | -1.10 | 1.07E-03 |
| ZFP28 | -1.10 | 1.86E-03 |
| LGALS1 | -1.10 | 3.58E-02 |
| CAST | -1.10 | 1.94E-02 |
| TBC1D16 | -1.10 | 1.80E-02 |
| C9orf24 | -1.10 | 5.54E-03 |
| NKIRAS1 | -1.10 | 6.05E-03 |
| JDP2 | -1.09 | 9.82E-03 |
| CYB5R3 | -1.09 | 8.18E-04 |
| RASL12 | -1.09 | 5.24E-03 |
| PSIP1 | -1.09 | 1.82E-03 |
| NFATC1 | -1.09 | 1.88E-02 |
| PLCL1 | -1.09 | 2.20E-02 |
| SEPTIN10 | -1.09 | 3.42E-02 |
| CORO1C | -1.09 | 1.98E-03 |
| CREBL2 | -1.09 | 1.88E-03 |
| MGAT3 | -1.09 | 4.61E-03 |
| SYNE1 | -1.09 | 2.22E-02 |
| NFIA | -1.09 | 1.25E-02 |
| EPHA4 | -1.08 | 3.57E-02 |
| NHS | -1.08 | 3.52E-02 |
| PCAT19 | -1.08 | 4.42E-04 |
| CLCN6 | -1.08 | 1.42E-03 |
| LYRM9 | -1.08 | 1.41E-02 |
| NQO2 | -1.08 | 4.20E-02 |
| GARS-DT | -1.08 | 5.58E-04 |
| NID1 | -1.08 | 4.26E-02 |
| STX2 | -1.07 | 1.49E-02 |
| SNTA1 | -1.07 | 4.45E-03 |
| HABP4 | -1.07 | 3.76E-04 |
| SCML1 | -1.07 | 3.87E-02 |
| USP30 | -1.07 | 1.18E-03 |
| SELENOP | -1.07 | 3.49E-02 |
| HYI | -1.07 | 7.35E-04 |
| CRYZL1 | -1.07 | 6.05E-04 |
| ZNF219 | -1.07 | 5.28E-04 |
| CCR10 | -1.06 | 1.75E-02 |
| PRDX6 | -1.06 | 6.29E-04 |
| HDAC5 | -1.06 | 8.65E-03 |
| DAB2IP | -1.06 | 1.24E-02 |
| HNMT | -1.06 | 4.69E-03 |
| PTPRS | -1.06 | 2.22E-02 |
| NFU1 | -1.05 | 1.95E-02 |
| SNN | -1.05 | 8.42E-03 |
| BEND5 | -1.05 | 3.54E-02 |
| MYO9A | -1.05 | 1.63E-03 |
| C16orf86 | -1.05 | 1.14E-02 |
| A2M-AS1 | -1.05 | 4.61E-02 |
| ECHDC2 | -1.05 | 7.21E-03 |
| EVI5 | -1.05 | 3.54E-03 |
| LYSMD4 | -1.05 | 2.54E-04 |
| LRP3 | -1.04 | 2.64E-02 |
| FAM122A | -1.04 | 2.55E-03 |
| RGS5 | -1.04 | 2.98E-02 |
| C20orf194 | -1.04 | 4.30E-04 |
| MSRA | -1.04 | 3.42E-02 |
| MAN2A2 | -1.04 | 5.66E-04 |
| ADH1A | -1.04 | 1.88E-03 |
| MTUS1 | -1.04 | 1.81E-02 |
| HDAC7 | -1.04 | 2.74E-03 |
| IFFO1 | -1.04 | 4.61E-02 |
| GALT | -1.03 | 3.83E-04 |
| FYCO1 | -1.03 | 3.36E-02 |
| SCAPER | -1.03 | 1.25E-03 |
| SAT2 | -1.03 | 2.40E-03 |
| PHKA2 | -1.03 | 5.20E-03 |
| CACNA2D4 | -1.03 | 9.03E-03 |
| EIF5A2 | -1.03 | 1.01E-02 |
| C22orf39 | -1.02 | 9.49E-03 |
| PDHA1 | -1.02 | 1.49E-03 |
| NEK7 | -1.02 | 3.51E-05 |
| LOC100287387 | -1.02 | 1.10E-02 |
| VIM | -1.02 | 6.29E-03 |
| PURA | -1.02 | 4.01E-03 |
| FOXN3-AS1 | -1.02 | 1.30E-02 |
| PMM1 | -1.02 | 7.59E-03 |
| KCNE1 | -1.02 | 3.32E-02 |
| RFLNB | -1.02 | 4.52E-02 |
| VPS13D | -1.01 | 2.43E-02 |
| NAALADL1 | -1.01 | 1.36E-03 |
| DOK1 | -1.01 | 6.59E-03 |
| EIF4E3 | -1.01 | 9.59E-03 |
| BBS12 | -1.01 | 4.27E-03 |
| CLCN4 | -1.01 | 1.97E-02 |
| ASB1 | -1.00 | 1.46E-02 |
| PRSS12 | -1.00 | 3.93E-03 |
| RIMKLB | -1.00 | 1.26E-02 |
| WDR19 | -1.00 | 4.02E-03 |
| PLCD3 | -1.00 | 8.48E-03 |
| TCP11L2 | -1.00 | 2.28E-03 |
| PEX5 | -1.00 | 3.52E-03 |
| TRIM37 | 1.00 | 4.04E-02 |
| MAPK6 | 1.00 | 8.43E-04 |
| MTPN | 1.00 | 4.27E-03 |
| ERCC6L | 1.00 | 4.33E-02 |
| KPNA1 | 1.01 | 4.92E-05 |
| SCAMP3 | 1.01 | 1.16E-04 |
| ZNF793-AS1 | 1.01 | 8.15E-03 |
| EIPR1 | 1.01 | 1.61E-02 |
| SHMT2 | 1.01 | 7.14E-03 |
| MOV10 | 1.01 | 1.67E-03 |
| NXT1 | 1.01 | 4.67E-02 |
| F2R | 1.01 | 2.17E-02 |
| LAMTOR2 | 1.01 | 6.28E-03 |
| EARS2 | 1.01 | 5.35E-03 |
| BRPF3 | 1.01 | 2.81E-02 |
| RPP40 | 1.01 | 7.34E-03 |
| EIF2AK3 | 1.01 | 1.25E-02 |
| ASCC3 | 1.01 | 4.59E-03 |
| SPC24 | 1.01 | 2.64E-02 |
| DAP3 | 1.01 | 1.03E-03 |
| NUTF2 | 1.01 | 1.54E-03 |
| NUP205 | 1.01 | 2.09E-02 |
| ALG6 | 1.01 | 7.66E-03 |
| NOP2 | 1.02 | 1.81E-02 |
| UBAP2L | 1.02 | 7.66E-03 |
| SLC31A1 | 1.02 | 2.43E-02 |
| MACO1 | 1.02 | 1.17E-02 |
| BDH1 | 1.02 | 2.92E-02 |
| MAGEF1 | 1.02 | 7.24E-03 |
| PPP1R13L | 1.02 | 4.77E-02 |
| MAGOHB | 1.02 | 8.67E-03 |
| SLC33A1 | 1.02 | 5.54E-03 |
| KCTD13 | 1.03 | 2.92E-03 |
| TMEM14A | 1.03 | 3.35E-04 |
| LSM7 | 1.03 | 8.34E-04 |
| PSMD14 | 1.03 | 3.54E-03 |
| TOR3A | 1.03 | 1.59E-03 |
| TARBP1 | 1.03 | 7.25E-03 |
| HEATR3 | 1.03 | 4.38E-04 |
| PTS | 1.03 | 1.89E-02 |
| MINPP1 | 1.03 | 5.62E-04 |
| LAPTM5 | 1.03 | 4.66E-02 |
| VOPP1 | 1.03 | 4.85E-04 |
| GORAB | 1.03 | 1.51E-04 |
| SRP54 | 1.03 | 4.78E-05 |
| DPM2 | 1.03 | 1.20E-03 |
| IGSF8 | 1.03 | 3.83E-02 |
| NOP58 | 1.03 | 2.85E-03 |
| MCM9 | 1.04 | 4.26E-03 |
| NCBP1 | 1.04 | 1.83E-03 |
| LNX2 | 1.04 | 3.12E-02 |
| COA6 | 1.04 | 1.35E-03 |
| NAA40 | 1.04 | 1.52E-03 |
| KIF9 | 1.04 | 7.90E-03 |
| ASPHD2 | 1.04 | 2.66E-02 |
| STIP1 | 1.04 | 2.20E-02 |
| UTP18 | 1.04 | 2.77E-02 |
| NDE1 | 1.04 | 6.20E-03 |
| CNOT11 | 1.04 | 5.11E-04 |
| TADA1 | 1.04 | 4.28E-04 |
| PRDM2 | 1.04 | 3.48E-04 |
| RGS10 | 1.05 | 7.18E-03 |
| SNRPD1 | 1.05 | 3.43E-03 |
| PEX13 | 1.05 | 1.94E-04 |
| HMG20B | 1.05 | 6.89E-03 |
| PPDPF | 1.05 | 1.22E-03 |
| ZMYND8 | 1.05 | 1.55E-02 |
| UCHL5 | 1.05 | 4.84E-03 |
| PDIA6 | 1.05 | 3.14E-02 |
| TBCE | 1.05 | 5.51E-03 |
| STYXL1 | 1.05 | 7.55E-03 |
| RAB11A | 1.05 | 2.05E-03 |
| SPICE1 | 1.05 | 3.91E-02 |
| C1orf131 | 1.05 | 7.54E-04 |
| CHML | 1.05 | 2.28E-02 |
| PGM2 | 1.05 | 7.93E-04 |
| FNDC10 | 1.05 | 3.09E-03 |
| CDCA4 | 1.05 | 4.03E-03 |
| MRPL14 | 1.06 | 3.68E-04 |
| CCT6A | 1.06 | 4.15E-04 |
| CXorf40A | 1.06 | 7.70E-03 |
| ATIC | 1.06 | 1.00E-02 |
| B3GALT6 | 1.06 | 1.51E-03 |
| WDR55 | 1.06 | 1.26E-03 |
| UFC1 | 1.06 | 5.09E-04 |
| APH1A | 1.06 | 9.14E-04 |
| EIF2AK1 | 1.06 | 3.86E-03 |
| TRIM11 | 1.06 | 2.78E-03 |
| KIF21A | 1.06 | 3.64E-03 |
| RAP2B | 1.06 | 3.52E-03 |
| SERP1 | 1.07 | 8.87E-05 |
| AMMECR1 | 1.07 | 1.90E-02 |
| MAP9 | 1.07 | 1.95E-02 |
| HMGB2 | 1.07 | 4.41E-02 |
| RPN2 | 1.07 | 2.57E-04 |
| SMARCA4 | 1.07 | 2.52E-03 |
| MST1R | 1.07 | 4.25E-02 |
| PIGN | 1.07 | 2.85E-02 |
| MTA3 | 1.07 | 8.48E-03 |
| DAP | 1.07 | 6.67E-04 |
| SFXN2 | 1.07 | 1.14E-02 |
| ITPR3 | 1.07 | 1.16E-02 |
| MIDN | 1.08 | 2.19E-02 |
| CNIH4 | 1.08 | 1.88E-02 |
| MRPS12 | 1.08 | 8.01E-03 |
| BCL2L11 | 1.08 | 7.19E-03 |
| PSMA5 | 1.08 | 1.89E-03 |
| PMM2 | 1.08 | 6.06E-03 |
| GID8 | 1.08 | 3.26E-04 |
| ACTR3 | 1.08 | 1.11E-03 |
| SRXN1 | 1.08 | 8.30E-03 |
| CCDC85C | 1.09 | 1.85E-02 |
| PATZ1 | 1.09 | 1.82E-02 |
| ZDHHC16 | 1.09 | 5.11E-03 |
| MOSPD1 | 1.09 | 2.17E-04 |
| MASTL | 1.09 | 4.60E-02 |
| YIPF1 | 1.09 | 2.68E-02 |
| ENTPD2 | 1.09 | 4.76E-02 |
| BOLA2 | 1.09 | 1.20E-02 |
| TMEM165 | 1.09 | 1.97E-04 |
| TXN | 1.09 | 2.07E-03 |
| SPRTN | 1.09 | 2.80E-04 |
| UBE2L6 | 1.09 | 3.36E-02 |
| SLC25A13 | 1.09 | 2.29E-03 |
| ORMDL2 | 1.09 | 4.16E-03 |
| ATP6V1G1 | 1.09 | 1.99E-02 |
| TIGD1 | 1.10 | 1.69E-02 |
| CASP3 | 1.10 | 3.83E-04 |
| JADE3 | 1.10 | 7.68E-03 |
| GPATCH4 | 1.10 | 3.40E-03 |
| ERGIC1 | 1.10 | 1.09E-02 |
| FARP1 | 1.10 | 4.01E-02 |
| FDPS | 1.11 | 9.54E-03 |
| STMP1 | 1.11 | 8.84E-03 |
| RNF19A | 1.11 | 2.19E-03 |
| CASKIN1 | 1.11 | 1.17E-04 |
| GTF2IRD1 | 1.11 | 1.47E-02 |
| GNPNAT1 | 1.11 | 1.42E-03 |
| SPATS2 | 1.11 | 6.56E-03 |
| CIAO2A | 1.11 | 3.22E-04 |
| DDX39A | 1.11 | 3.97E-03 |
| MANF | 1.11 | 5.47E-03 |
| PTP4A1 | 1.11 | 4.48E-04 |
| ARRDC1 | 1.11 | 1.55E-03 |
| TIAM1 | 1.11 | 4.00E-02 |
| ZNF296 | 1.11 | 2.79E-02 |
| ILF2 | 1.11 | 2.28E-03 |
| LAT2 | 1.11 | 1.75E-02 |
| PDIK1L | 1.11 | 2.61E-02 |
| KIF22 | 1.11 | 2.40E-02 |
| HSPE1 | 1.12 | 4.95E-03 |
| ZNF326 | 1.12 | 2.76E-04 |
| PAXIP1 | 1.12 | 2.04E-03 |
| RNPEP | 1.12 | 1.60E-03 |
| SUSD6 | 1.12 | 2.41E-03 |
| ENSA | 1.12 | 2.35E-03 |
| AP1G2 | 1.12 | 1.01E-02 |
| SMIM29 | 1.13 | 1.77E-02 |
| C8orf76 | 1.13 | 3.31E-02 |
| OSTC | 1.13 | 2.05E-04 |
| RFC4 | 1.13 | 1.59E-02 |
| SFXN1 | 1.13 | 8.48E-03 |
| TRIM3 | 1.13 | 1.02E-02 |
| SMS | 1.14 | 5.73E-04 |
| FANCF | 1.14 | 6.59E-03 |
| GSKIP | 1.14 | 2.88E-04 |
| TBC1D2 | 1.14 | 5.28E-03 |
| NACC1 | 1.14 | 4.59E-04 |
| RNF213 | 1.14 | 4.13E-02 |
| NANS | 1.14 | 6.51E-03 |
| FBLIM1 | 1.14 | 3.13E-02 |
| ZNF710 | 1.15 | 2.05E-03 |
| SMCO4 | 1.15 | 3.88E-02 |
| GPD2 | 1.15 | 2.92E-02 |
| ZNF107 | 1.15 | 1.82E-02 |
| GLA | 1.15 | 3.00E-02 |
| SNRPB | 1.15 | 1.21E-02 |
| PEX7 | 1.15 | 2.49E-02 |
| VPS37C | 1.15 | 2.74E-02 |
| TMEM141 | 1.15 | 3.82E-03 |
| CXorf40B | 1.15 | 5.50E-03 |
| C1orf162 | 1.15 | 4.89E-02 |
| PLK1 | 1.15 | 3.01E-02 |
| SNRPG | 1.15 | 1.24E-04 |
| SLX4IP | 1.15 | 2.65E-03 |
| INTS8 | 1.16 | 8.04E-03 |
| MCM8 | 1.16 | 1.03E-02 |
| TRIM46 | 1.16 | 3.93E-02 |
| POLR2H | 1.16 | 2.90E-04 |
| CTSD | 1.16 | 3.57E-02 |
| GPSM2 | 1.17 | 3.26E-02 |
| CMTM4 | 1.17 | 2.98E-02 |
| TGIF1 | 1.17 | 7.93E-04 |
| NFKBIE | 1.17 | 2.82E-03 |
| HIF1A | 1.17 | 6.51E-03 |
| RIT1 | 1.17 | 1.78E-02 |
| YRDC | 1.18 | 1.57E-03 |
| RASSF5 | 1.18 | 3.53E-02 |
| PGK1 | 1.18 | 3.34E-04 |
| ZNF101 | 1.18 | 3.24E-02 |
| G6PD | 1.18 | 3.42E-02 |
| FAM91A1 | 1.18 | 5.43E-03 |
| USP18 | 1.18 | 3.28E-02 |
| RIPK2 | 1.18 | 4.27E-03 |
| GPR157 | 1.18 | 2.92E-02 |
| SEC24D | 1.19 | 8.62E-03 |
| NUDCD1 | 1.19 | 4.32E-02 |
| SLC35F5 | 1.19 | 2.28E-03 |
| LPCAT1 | 1.19 | 2.42E-02 |
| RDH11 | 1.19 | 1.06E-04 |
| TMC6 | 1.19 | 4.34E-03 |
| BEND3 | 1.19 | 1.70E-02 |
| SRPRB | 1.19 | 5.02E-04 |
| TMEM87B | 1.19 | 6.33E-03 |
| PNPT1 | 1.19 | 3.87E-03 |
| ZNF814 | 1.19 | 4.41E-02 |
| ATAD2B | 1.20 | 9.70E-03 |
| CSTF2 | 1.20 | 9.95E-03 |
| PHKA1 | 1.20 | 3.59E-02 |
| LILRB4 | 1.20 | 4.42E-02 |
| CHPF2 | 1.20 | 2.19E-03 |
| HM13 | 1.20 | 9.15E-03 |
| TDG | 1.20 | 4.81E-04 |
| PPP3R1 | 1.20 | 2.80E-02 |
| IER5 | 1.20 | 1.06E-02 |
| TMCO1 | 1.20 | 3.00E-04 |
| SEC23B | 1.21 | 7.96E-04 |
| ZNF827 | 1.21 | 5.92E-04 |
| CYP2R1 | 1.21 | 2.04E-02 |
| AAGAB | 1.22 | 1.95E-03 |
| STAT1 | 1.22 | 4.37E-02 |
| HES1 | 1.22 | 3.91E-02 |
| RFX5 | 1.22 | 7.40E-04 |
| ZNF217 | 1.22 | 6.49E-04 |
| EFNA1 | 1.22 | 4.62E-03 |
| HSPA14 | 1.22 | 2.95E-02 |
| AP1S1 | 1.22 | 4.69E-05 |
| SEMA3F | 1.22 | 2.69E-02 |
| AGPAT5 | 1.22 | 1.45E-02 |
| C12orf49 | 1.23 | 4.40E-03 |
| RAN | 1.23 | 5.92E-04 |
| TXNDC17 | 1.23 | 5.27E-05 |
| IPO4 | 1.23 | 2.80E-03 |
| LSM4 | 1.23 | 6.50E-04 |
| SNX25 | 1.23 | 1.55E-03 |
| IDI1 | 1.23 | 9.57E-03 |
| FBXO41 | 1.23 | 1.51E-02 |
| WDR12 | 1.24 | 3.31E-04 |
| PIGM | 1.24 | 1.70E-04 |
| HLA-DOA | 1.24 | 2.02E-02 |
| FAM102A | 1.24 | 1.76E-03 |
| F11R | 1.24 | 1.71E-03 |
| APOO | 1.24 | 9.23E-03 |
| GGTLC1 | 1.25 | 3.93E-02 |
| SAYSD1 | 1.25 | 4.94E-04 |
| ZNF281 | 1.25 | 4.70E-03 |
| AHCY | 1.25 | 6.73E-03 |
| COMMD5 | 1.25 | 1.91E-02 |
| DNAJC10 | 1.25 | 2.28E-03 |
| CDK5R1 | 1.25 | 3.28E-02 |
| DNAJC9 | 1.25 | 2.33E-04 |
| EME2 | 1.26 | 4.45E-02 |
| TMEM40 | 1.26 | 3.47E-02 |
| SDC4 | 1.26 | 2.81E-02 |
| CNNM4 | 1.26 | 2.10E-02 |
| DYNLT1 | 1.28 | 2.32E-03 |
| HNRNPAB | 1.28 | 4.20E-04 |
| FUT8 | 1.28 | 2.69E-02 |
| PARVG | 1.28 | 1.75E-02 |
| FYB1 | 1.28 | 4.93E-02 |
| NAXE | 1.28 | 2.83E-03 |
| SGK1 | 1.28 | 1.14E-02 |
| PSENEN | 1.28 | 1.77E-03 |
| TPM3 | 1.29 | 1.34E-04 |
| QSOX1 | 1.29 | 1.09E-02 |
| LEO1 | 1.29 | 7.81E-03 |
| PTRH2 | 1.29 | 2.46E-02 |
| PLEKHA8 | 1.29 | 3.66E-02 |
| POGK | 1.29 | 3.78E-04 |
| PIP4K2C | 1.29 | 6.07E-04 |
| UBQLN4 | 1.30 | 5.43E-03 |
| S100A11 | 1.30 | 6.79E-03 |
| DPCD | 1.30 | 5.71E-04 |
| DANCR | 1.30 | 3.93E-02 |
| TTLL12 | 1.30 | 4.06E-03 |
| KIAA1211L | 1.30 | 3.87E-03 |
| EPHA1 | 1.30 | 2.01E-02 |
| PYCR3 | 1.30 | 4.98E-02 |
| RRP15 | 1.31 | 2.19E-05 |
| SIPA1L3 | 1.31 | 3.65E-03 |
| PARP1 | 1.31 | 3.56E-04 |
| GPR68 | 1.31 | 2.97E-02 |
| ANKRD37 | 1.31 | 1.67E-02 |
| RUSC1-AS1 | 1.31 | 1.10E-02 |
| ARHGEF19 | 1.31 | 4.75E-02 |
| ELL3 | 1.32 | 3.87E-02 |
| PRDX1 | 1.32 | 6.14E-03 |
| TIMM17A | 1.32 | 2.03E-03 |
| ACSL3 | 1.32 | 7.07E-03 |
| WEE1 | 1.32 | 1.09E-02 |
| TOMM34 | 1.32 | 6.21E-05 |
| FBXO5 | 1.32 | 1.54E-02 |
| NCAPH | 1.33 | 4.49E-02 |
| DNMT1 | 1.33 | 1.49E-04 |
| DOCK5 | 1.33 | 4.81E-02 |
| SDF2L1 | 1.33 | 9.04E-03 |
| SLC44A1 | 1.33 | 1.99E-04 |
| SCCPDH | 1.33 | 3.20E-02 |
| CENPX | 1.33 | 2.17E-02 |
| TCF3 | 1.33 | 9.69E-04 |
| ODF2 | 1.33 | 2.89E-03 |
| ADIPOR1 | 1.33 | 4.62E-03 |
| ALDH18A1 | 1.33 | 4.44E-04 |
| TRIM14 | 1.34 | 2.40E-03 |
| SGPL1 | 1.34 | 4.79E-04 |
| SNRPE | 1.34 | 9.90E-04 |
| GEN1 | 1.34 | 3.08E-03 |
| PAK4 | 1.34 | 6.09E-04 |
| EEF1E1 | 1.34 | 1.89E-03 |
| KNTC1 | 1.34 | 2.13E-02 |
| DNAAF4 | 1.35 | 1.87E-02 |
| PSPH | 1.35 | 2.92E-02 |
| RAB17 | 1.35 | 3.16E-02 |
| CXCR3 | 1.35 | 3.69E-02 |
| SRPK1 | 1.35 | 2.67E-03 |
| TSTD1 | 1.35 | 1.66E-02 |
| GSR | 1.35 | 1.24E-02 |
| PARP9 | 1.35 | 1.36E-02 |
| MTHFD2L | 1.36 | 1.12E-02 |
| HDGF | 1.36 | 3.10E-04 |
| CENPM | 1.36 | 1.01E-02 |
| TCTEX1D2 | 1.36 | 1.22E-03 |
| LPAR2 | 1.36 | 1.12E-02 |
| NUDT16L1 | 1.36 | 9.07E-04 |
| RNF24 | 1.36 | 2.27E-02 |
| P4HA1 | 1.36 | 1.17E-02 |
| CDKN1A | 1.37 | 4.56E-02 |
| GSPT1 | 1.37 | 1.13E-05 |
| TNFSF4 | 1.37 | 3.07E-02 |
| CEP70 | 1.37 | 5.48E-03 |
| WDR4 | 1.37 | 2.33E-02 |
| MPZL3 | 1.37 | 3.67E-02 |
| IRX3 | 1.37 | 4.54E-02 |
| CENPL | 1.37 | 1.83E-04 |
| TMEM86A | 1.38 | 4.76E-02 |
| HMGCS1 | 1.38 | 3.23E-02 |
| PHF19 | 1.38 | 7.32E-04 |
| MAPKAPK2 | 1.38 | 6.15E-03 |
| TNIK | 1.38 | 1.11E-02 |
| LRBA | 1.38 | 1.15E-03 |
| ZNF468 | 1.38 | 2.75E-03 |
| C1orf112 | 1.38 | 1.54E-03 |
| METTL21A | 1.39 | 2.30E-03 |
| MOB1A | 1.39 | 1.89E-04 |
| PASK | 1.39 | 1.88E-02 |
| CABLES2 | 1.39 | 1.77E-02 |
| DEPDC1B | 1.39 | 1.54E-02 |
| UGCG | 1.39 | 4.42E-02 |
| LYPLA1 | 1.39 | 2.66E-02 |
| RAI14 | 1.39 | 1.19E-02 |
| TIMP1 | 1.39 | 3.18E-04 |
| SERTAD4 | 1.40 | 8.48E-03 |
| SHTN1 | 1.40 | 7.39E-04 |
| ATP6AP1 | 1.40 | 2.70E-05 |
| ZSCAN16 | 1.40 | 1.70E-04 |
| PRRG4 | 1.40 | 3.94E-02 |
| PPP1R14B | 1.41 | 7.58E-03 |
| ACP5 | 1.41 | 4.64E-02 |
| CSK | 1.41 | 4.15E-04 |
| AP1AR | 1.41 | 5.60E-03 |
| NECTIN4 | 1.42 | 1.31E-02 |
| CHST11 | 1.42 | 2.46E-02 |
| ALYREF | 1.42 | 1.25E-02 |
| FAM174B | 1.43 | 1.82E-02 |
| HPRT1 | 1.43 | 2.31E-04 |
| PARP12 | 1.43 | 5.44E-04 |
| CCDC88C | 1.43 | 2.96E-02 |
| TBC1D7 | 1.43 | 2.68E-03 |
| TP53INP1 | 1.43 | 1.39E-02 |
| SLC6A6 | 1.43 | 2.24E-04 |
| CERS2 | 1.43 | 2.00E-02 |
| POLR3K | 1.44 | 1.77E-02 |
| MSI2 | 1.44 | 1.42E-02 |
| MACROH2A1 | 1.44 | 9.01E-06 |
| DCAF13 | 1.44 | 3.51E-03 |
| H2AZ2 | 1.44 | 2.04E-03 |
| CCT3 | 1.45 | 2.71E-04 |
| RUSC1 | 1.45 | 1.25E-03 |
| KDELR2 | 1.45 | 3.94E-03 |
| RALGPS2 | 1.45 | 1.97E-02 |
| DEF6 | 1.45 | 4.15E-03 |
| COL13A1 | 1.45 | 4.33E-02 |
| CYFIP2 | 1.45 | 1.34E-02 |
| WDR34 | 1.45 | 1.19E-03 |
| ECE2 | 1.45 | 1.13E-03 |
| ZNF93 | 1.45 | 1.43E-02 |
| ACOT7 | 1.45 | 2.10E-02 |
| KDF1 | 1.46 | 1.81E-03 |
| ARFIP2 | 1.46 | 1.58E-02 |
| TMEM51 | 1.46 | 5.89E-04 |
| GPR65 | 1.46 | 3.19E-02 |
| ZNF692 | 1.46 | 2.66E-03 |
| DDIAS | 1.46 | 3.85E-02 |
| SYNGR2 | 1.46 | 1.00E-03 |
| PRXL2B | 1.46 | 2.04E-03 |
| DHRS13 | 1.46 | 2.98E-02 |
| SEPHS2 | 1.47 | 1.08E-03 |
| E2F3 | 1.47 | 6.58E-04 |
| XPR1 | 1.47 | 2.92E-02 |
| CDC42SE1 | 1.47 | 5.30E-05 |
| CRNDE | 1.47 | 8.44E-03 |
| ZNF165 | 1.48 | 4.37E-02 |
| PPIL1 | 1.48 | 3.31E-04 |
| SYTL2 | 1.48 | 2.84E-02 |
| DCTPP1 | 1.48 | 1.35E-03 |
| BZW2 | 1.48 | 1.29E-02 |
| TIGAR | 1.48 | 1.96E-03 |
| JAK3 | 1.49 | 3.99E-02 |
| STMN1 | 1.49 | 6.47E-03 |
| CANT1 | 1.49 | 1.96E-02 |
| B4GALT3 | 1.49 | 2.86E-04 |
| NAT14 | 1.49 | 3.84E-02 |
| LINC02603 | 1.50 | 3.39E-02 |
| DOCK10 | 1.50 | 3.48E-02 |
| MRPL42 | 1.50 | 2.77E-04 |
| RCC2 | 1.50 | 3.31E-04 |
| SCYL3 | 1.50 | 5.01E-04 |
| MRPL13 | 1.51 | 1.63E-02 |
| DONSON | 1.51 | 5.31E-03 |
| ZNRF2 | 1.51 | 5.04E-05 |
| NVL | 1.52 | 3.05E-03 |
| PRPS2 | 1.52 | 1.02E-03 |
| RHNO1 | 1.53 | 2.32E-04 |
| KIAA0895 | 1.53 | 1.42E-02 |
| RCC1 | 1.53 | 1.42E-03 |
| PLAAT4 | 1.53 | 3.54E-02 |
| GPR137B | 1.53 | 1.29E-04 |
| CD72 | 1.53 | 2.92E-02 |
| ADSS2 | 1.53 | 7.38E-05 |
| PUSL1 | 1.53 | 9.07E-03 |
| HACD3 | 1.53 | 6.36E-03 |
| BHLHE40 | 1.53 | 5.48E-03 |
| PTK7 | 1.54 | 5.30E-04 |
| TUFT1 | 1.54 | 6.91E-03 |
| TMEM54 | 1.54 | 1.74E-02 |
| ATP2A3 | 1.54 | 3.26E-02 |
| GSTCD | 1.54 | 6.83E-04 |
| ZDHHC13 | 1.55 | 4.57E-03 |
| SPINT1-AS1 | 1.55 | 3.52E-03 |
| CARMIL1 | 1.55 | 1.76E-03 |
| LY86 | 1.55 | 1.18E-02 |
| LIG1 | 1.56 | 1.80E-03 |
| RNFT2 | 1.56 | 1.12E-02 |
| SUCO | 1.56 | 9.18E-04 |
| H2AZ1 | 1.56 | 2.32E-04 |
| FKBP4 | 1.56 | 1.88E-02 |
| TOB1 | 1.56 | 1.54E-03 |
| HOMER1 | 1.56 | 4.56E-02 |
| BRI3BP | 1.58 | 9.54E-04 |
| TSEN54 | 1.58 | 6.82E-03 |
| BCAS4 | 1.58 | 5.51E-03 |
| HMGCR | 1.58 | 5.06E-03 |
| MCM10 | 1.58 | 3.98E-02 |
| NFYA | 1.58 | 4.28E-04 |
| RUVBL1 | 1.58 | 1.49E-03 |
| RHOF | 1.59 | 1.89E-02 |
| B3GALNT1 | 1.59 | 6.29E-04 |
| GOT2 | 1.59 | 2.82E-03 |
| LAPTM4B | 1.59 | 3.91E-02 |
| ISG20L2 | 1.59 | 8.85E-05 |
| OASL | 1.59 | 3.29E-02 |
| TIMELESS | 1.59 | 1.46E-04 |
| SNX20 | 1.59 | 3.27E-02 |
| NIPSNAP1 | 1.59 | 4.45E-04 |
| SUV39H2 | 1.60 | 2.80E-02 |
| SERPINE1 | 1.60 | 2.85E-02 |
| DHCR24 | 1.60 | 4.64E-02 |
| MAGED2 | 1.60 | 3.82E-03 |
| SPTSSA | 1.60 | 3.38E-03 |
| BPNT1 | 1.60 | 3.80E-04 |
| LMBR1 | 1.60 | 6.47E-04 |
| CDCA2 | 1.60 | 7.89E-03 |
| DENND1B | 1.60 | 8.81E-06 |
| PDSS1 | 1.60 | 1.28E-02 |
| SERPINE2 | 1.60 | 8.26E-03 |
| S1PR3 | 1.60 | 4.59E-03 |
| ATP6V0B | 1.61 | 2.89E-03 |
| PSD4 | 1.61 | 3.78E-04 |
| LRRC59 | 1.61 | 1.34E-02 |
| BICDL2 | 1.61 | 5.70E-03 |
| ZBED4 | 1.61 | 3.29E-04 |
| RNASEH2A | 1.62 | 4.27E-03 |
| RUNX1-IT1 | 1.62 | 1.43E-03 |
| CDT1 | 1.62 | 7.14E-03 |
| MCM4 | 1.62 | 1.68E-03 |
| GCAT | 1.63 | 3.76E-02 |
| CDH2 | 1.63 | 4.75E-02 |
| CACNB3 | 1.63 | 6.33E-03 |
| GCNT1 | 1.63 | 6.55E-03 |
| MTFP1 | 1.64 | 7.93E-04 |
| CYB561 | 1.64 | 6.72E-03 |
| VAMP8 | 1.64 | 1.39E-04 |
| ASCL2 | 1.64 | 2.62E-02 |
| PAICS | 1.64 | 1.59E-03 |
| ZWILCH | 1.64 | 1.76E-05 |
| CENPW | 1.65 | 8.03E-03 |
| RASL11B | 1.65 | 5.60E-03 |
| STXBP2 | 1.65 | 3.34E-05 |
| MYBL2 | 1.65 | 2.54E-02 |
| TACC3 | 1.65 | 1.44E-03 |
| MAGED1 | 1.65 | 8.72E-04 |
| SLC52A2 | 1.66 | 8.72E-04 |
| MXRA5 | 1.66 | 1.13E-02 |
| PLEK2 | 1.66 | 6.84E-03 |
| SLC35F2 | 1.66 | 3.84E-02 |
| RMI1 | 1.66 | 2.12E-05 |
| GK | 1.67 | 8.89E-03 |
| APOC1 | 1.67 | 4.23E-03 |
| AMPD3 | 1.67 | 4.56E-03 |
| RHOV | 1.67 | 1.96E-02 |
| JPT2 | 1.67 | 3.90E-05 |
| ITGAX | 1.68 | 6.89E-03 |
| SMC4 | 1.68 | 3.86E-04 |
| HJURP | 1.68 | 1.17E-02 |
| KCTD5 | 1.68 | 1.78E-05 |
| SEMA4D | 1.68 | 7.99E-03 |
| IL4I1 | 1.68 | 3.83E-02 |
| ARSD | 1.68 | 7.02E-04 |
| CEP85 | 1.69 | 1.17E-02 |
| ZNF367 | 1.69 | 2.17E-03 |
| CDK5 | 1.69 | 1.76E-03 |
| REEP6 | 1.69 | 1.42E-02 |
| ROGDI | 1.69 | 2.43E-03 |
| ADA2 | 1.70 | 3.59E-02 |
| CBX4 | 1.70 | 6.85E-03 |
| ATP1B1 | 1.70 | 4.45E-02 |
| TGM2 | 1.70 | 1.06E-02 |
| AURKB | 1.70 | 9.10E-03 |
| MMEL1 | 1.71 | 4.36E-02 |
| TP53RK | 1.71 | 1.86E-04 |
| CAPG | 1.71 | 2.78E-05 |
| KIF18B | 1.71 | 3.33E-02 |
| EP300-AS1 | 1.71 | 4.04E-02 |
| BASP1 | 1.71 | 1.54E-03 |
| FHDC1 | 1.71 | 1.63E-03 |
| LMO7 | 1.72 | 2.76E-03 |
| ZNF467 | 1.73 | 2.61E-02 |
| MCOLN2 | 1.73 | 3.48E-02 |
| FEN1 | 1.73 | 3.21E-04 |
| RABEP2 | 1.73 | 3.75E-04 |
| TTC9 | 1.73 | 1.86E-02 |
| CIP2A | 1.73 | 8.01E-03 |
| KDM5B | 1.73 | 1.29E-03 |
| HSPH1 | 1.73 | 5.46E-04 |
| CAMSAP3 | 1.74 | 4.10E-03 |
| TFRC | 1.74 | 3.38E-03 |
| RPGRIP1L | 1.74 | 1.70E-02 |
| GRHL2 | 1.75 | 3.68E-04 |
| IKZF2 | 1.75 | 1.19E-02 |
| PPM1H | 1.75 | 2.10E-02 |
| VDR | 1.75 | 1.21E-02 |
| HMGN1 | 1.75 | 5.86E-04 |
| PKM | 1.76 | 7.81E-05 |
| TMEM154 | 1.76 | 4.32E-03 |
| PIK3R2 | 1.76 | 4.76E-05 |
| HLA-DQB1 | 1.76 | 1.38E-02 |
| KIAA1522 | 1.76 | 7.05E-04 |
| MYCL | 1.77 | 2.23E-03 |
| NME1 | 1.77 | 9.29E-03 |
| PCNA | 1.77 | 2.62E-04 |
| MAP3K9 | 1.77 | 7.55E-04 |
| RPS6KA1 | 1.78 | 8.18E-04 |
| IRF7 | 1.78 | 2.48E-04 |
| DLG3 | 1.78 | 9.37E-04 |
| DUSP10 | 1.79 | 1.38E-03 |
| EME1 | 1.79 | 1.18E-02 |
| ASF1B | 1.80 | 7.72E-03 |
| SLC35A2 | 1.80 | 2.54E-05 |
| LMNB2 | 1.80 | 3.78E-05 |
| COL9A2 | 1.80 | 1.84E-02 |
| SEMA4B | 1.80 | 1.54E-03 |
| NECTIN2 | 1.81 | 3.91E-03 |
| OAS2 | 1.81 | 4.89E-02 |
| ATP8B1 | 1.81 | 1.24E-03 |
| SUSD2 | 1.81 | 3.83E-02 |
| RSPH1 | 1.82 | 1.10E-03 |
| DNMT3A | 1.82 | 9.51E-04 |
| OSBPL3 | 1.82 | 5.57E-03 |
| TSPAN13 | 1.82 | 1.52E-03 |
| MBOAT7 | 1.82 | 4.75E-05 |
| MAP7 | 1.82 | 9.53E-03 |
| CD9 | 1.82 | 1.21E-03 |
| PRR36 | 1.83 | 4.98E-02 |
| PDIA4 | 1.83 | 1.92E-04 |
| DPP3 | 1.83 | 1.92E-03 |
| FGD6 | 1.84 | 4.05E-03 |
| LPXN | 1.84 | 1.97E-04 |
| TMTC4 | 1.85 | 7.25E-04 |
| PLEKHF2 | 1.85 | 4.41E-03 |
| ISG15 | 1.85 | 4.50E-02 |
| RAD54B | 1.85 | 1.37E-02 |
| SINHCAF | 1.85 | 6.23E-03 |
| INTS7 | 1.85 | 9.68E-03 |
| RACGAP1 | 1.86 | 1.42E-03 |
| SPC25 | 1.86 | 1.53E-02 |
| ERO1A | 1.86 | 7.32E-04 |
| SMYD3 | 1.87 | 4.84E-03 |
| CKAP2 | 1.87 | 1.83E-04 |
| IL18 | 1.88 | 8.70E-04 |
| MOCOS | 1.88 | 3.77E-02 |
| UNC5B | 1.88 | 1.24E-03 |
| MREG | 1.88 | 1.85E-03 |
| FAAH2 | 1.88 | 9.57E-03 |
| LRATD2 | 1.89 | 1.09E-02 |
| C3orf80 | 1.89 | 4.08E-03 |
| ATP2A2 | 1.89 | 8.93E-07 |
| SLC20A1 | 1.90 | 7.59E-06 |
| DNMT3B | 1.90 | 7.10E-03 |
| METRN | 1.90 | 1.66E-02 |
| HMGA1 | 1.90 | 8.40E-04 |
| PRKCZ | 1.90 | 1.31E-03 |
| PNP | 1.90 | 3.19E-04 |
| CCDC167 | 1.91 | 3.64E-04 |
| IDH2 | 1.91 | 2.38E-04 |
| MPHOSPH6 | 1.92 | 4.29E-03 |
| POLE2 | 1.92 | 3.17E-04 |
| SLC2A1 | 1.92 | 1.20E-03 |
| BLNK | 1.92 | 2.32E-04 |
| STIL | 1.92 | 5.85E-04 |
| ST14 | 1.92 | 1.08E-03 |
| BORA | 1.92 | 3.84E-04 |
| RAD51 | 1.92 | 5.64E-03 |
| ARNT2 | 1.92 | 3.95E-02 |
| RBM47 | 1.92 | 1.11E-03 |
| ELMO3 | 1.93 | 1.16E-02 |
| STRBP | 1.94 | 2.02E-03 |
| H2AX | 1.94 | 8.77E-04 |
| SLC50A1 | 1.94 | 9.48E-05 |
| PGM2L1 | 1.95 | 1.89E-02 |
| MICAL2 | 1.95 | 2.75E-03 |
| EFNA3 | 1.95 | 1.03E-02 |
| GOLM1 | 1.95 | 7.91E-03 |
| HOXC13 | 1.96 | 5.25E-03 |
| TET3 | 1.96 | 2.13E-04 |
| ABHD11 | 1.96 | 2.74E-03 |
| CKAP4 | 1.96 | 5.98E-03 |
| ERMP1 | 1.96 | 3.39E-04 |
| ARHGAP32 | 1.96 | 1.02E-02 |
| FBP1 | 1.97 | 4.72E-02 |
| SHB | 1.97 | 2.67E-02 |
| CERS6 | 1.97 | 5.12E-03 |
| FN1 | 1.97 | 3.82E-03 |
| RAB11FIP1 | 1.99 | 2.00E-02 |
| LAGE3 | 1.99 | 9.40E-04 |
| STAP2 | 1.99 | 5.77E-03 |
| QPRT | 1.99 | 7.89E-03 |
| SLC2A10 | 1.99 | 5.12E-03 |
| TRAF3IP3 | 1.99 | 1.72E-02 |
| SEMA4A | 2.00 | 3.87E-03 |
| CDYL2 | 2.00 | 2.07E-02 |
| TANC2 | 2.00 | 1.36E-02 |
| ZBTB42 | 2.00 | 4.81E-03 |
| KRT80 | 2.01 | 2.23E-02 |
| GRP | 2.01 | 4.13E-02 |
| CNTNAP2 | 2.01 | 1.37E-02 |
| DBF4 | 2.02 | 1.20E-04 |
| TMEM30B | 2.02 | 4.95E-03 |
| LYZ | 2.02 | 9.38E-04 |
| IGSF3 | 2.02 | 5.78E-04 |
| ANK3 | 2.02 | 9.56E-03 |
| CCR5 | 2.03 | 1.14E-02 |
| PLPP5 | 2.03 | 3.96E-03 |
| RABIF | 2.03 | 9.18E-04 |
| TFEC | 2.04 | 4.62E-02 |
| GPATCH2 | 2.04 | 4.65E-02 |
| GGCT | 2.04 | 1.59E-03 |
| PAQR4 | 2.04 | 2.92E-03 |
| AIM2 | 2.05 | 4.03E-02 |
| OCIAD2 | 2.05 | 3.57E-04 |
| CENPN | 2.06 | 2.44E-02 |
| SAP30 | 2.06 | 3.85E-04 |
| NDUFAF6 | 2.06 | 1.50E-02 |
| SOX4 | 2.06 | 4.00E-04 |
| XBP1 | 2.06 | 8.04E-03 |
| SPA17 | 2.06 | 3.81E-03 |
| ADAM19 | 2.07 | 1.20E-03 |
| E2F5 | 2.07 | 5.11E-03 |
| HELLS | 2.07 | 4.59E-04 |
| MBOAT2 | 2.07 | 7.77E-03 |
| RAB3D | 2.08 | 2.16E-04 |
| CDCA8 | 2.08 | 6.74E-03 |
| NCAPG | 2.09 | 2.78E-03 |
| ENAH | 2.09 | 2.90E-04 |
| CHEK1 | 2.09 | 1.30E-02 |
| BGN | 2.09 | 2.64E-03 |
| KPNA2 | 2.10 | 2.28E-04 |
| BARD1 | 2.10 | 3.57E-04 |
| STX3 | 2.10 | 2.14E-04 |
| MND1 | 2.10 | 1.52E-02 |
| MAL2 | 2.10 | 8.29E-03 |
| LOC374443 | 2.10 | 2.16E-03 |
| PYCR1 | 2.11 | 6.54E-05 |
| BRIP1 | 2.11 | 6.08E-03 |
| LINC01094 | 2.11 | 6.02E-03 |
| CD37 | 2.11 | 1.21E-02 |
| MTHFD2 | 2.12 | 2.78E-05 |
| SIDT1 | 2.12 | 3.17E-02 |
| ADAM28 | 2.12 | 2.90E-03 |
| PIMREG | 2.13 | 6.92E-04 |
| SLC15A2 | 2.14 | 4.13E-02 |
| DNAH14 | 2.15 | 3.12E-03 |
| CD52 | 2.15 | 4.49E-02 |
| LRRC1 | 2.15 | 9.52E-05 |
| CAPS | 2.15 | 3.36E-02 |
| GCH1 | 2.15 | 9.07E-04 |
| CTPS1 | 2.15 | 4.58E-04 |
| MYEF2 | 2.16 | 2.62E-03 |
| PTK6 | 2.16 | 1.24E-02 |
| PLAUR | 2.16 | 1.53E-03 |
| TMED3 | 2.16 | 9.53E-05 |
| MGAT4A | 2.17 | 4.27E-03 |
| SLC1A4 | 2.17 | 5.11E-03 |
| TSTA3 | 2.17 | 3.24E-03 |
| CDCA7 | 2.17 | 2.09E-02 |
| PSRC1 | 2.17 | 2.96E-03 |
| RAB3IP | 2.17 | 9.43E-03 |
| STK26 | 2.18 | 7.34E-03 |
| PARD6B | 2.18 | 3.06E-02 |
| SLC39A11 | 2.18 | 6.13E-03 |
| CDH11 | 2.19 | 1.49E-03 |
| BICDL1 | 2.19 | 8.04E-04 |
| MYO6 | 2.19 | 4.21E-04 |
| OLR1 | 2.20 | 3.48E-02 |
| DOP1B | 2.20 | 4.20E-04 |
| PCAT6 | 2.20 | 2.54E-02 |
| RTKN | 2.21 | 1.25E-03 |
| PLPP4 | 2.21 | 4.92E-02 |
| CYTIP | 2.21 | 3.18E-02 |
| SHROOM3 | 2.21 | 4.37E-02 |
| C1orf56 | 2.22 | 3.68E-04 |
| GDPD1 | 2.23 | 2.67E-02 |
| OPN3 | 2.23 | 2.87E-03 |
| SRD5A3 | 2.23 | 4.34E-03 |
| GRHL1 | 2.23 | 3.43E-02 |
| EZR | 2.23 | 3.66E-04 |
| ABRACL | 2.23 | 9.81E-04 |
| RORC | 2.24 | 3.90E-02 |
| SIX4 | 2.24 | 8.02E-05 |
| LOC100505938 | 2.24 | 8.24E-04 |
| H2AW | 2.24 | 2.07E-02 |
| POSTN | 2.24 | 1.95E-03 |
| EXO1 | 2.25 | 6.38E-03 |
| ENC1 | 2.25 | 3.49E-03 |
| UBE2S | 2.25 | 3.57E-03 |
| MSMO1 | 2.25 | 9.49E-04 |
| ISG20 | 2.25 | 1.02E-02 |
| TMEM79 | 2.26 | 1.02E-03 |
| PLS1 | 2.26 | 4.40E-02 |
| CDC7 | 2.26 | 2.54E-03 |
| CST6 | 2.26 | 4.27E-02 |
| EPN3 | 2.26 | 3.00E-02 |
| FLVCR1 | 2.26 | 4.72E-04 |
| BCL11B | 2.27 | 1.38E-02 |
| H2BC12 | 2.27 | 6.11E-03 |
| EPSTI1 | 2.27 | 8.25E-03 |
| BLM | 2.27 | 4.16E-04 |
| MCM2 | 2.28 | 8.48E-04 |
| CLDN4 | 2.30 | 3.12E-03 |
| RGS4 | 2.30 | 3.43E-02 |
| TMEM125 | 2.30 | 4.23E-03 |
| PABPC1L | 2.30 | 1.53E-02 |
| RAP1GAP | 2.31 | 1.88E-02 |
| ESPN | 2.33 | 3.21E-03 |
| LLGL2 | 2.33 | 1.66E-04 |
| RUNX2 | 2.33 | 1.82E-02 |
| SGO2 | 2.34 | 6.53E-05 |
| ADAM12 | 2.34 | 3.03E-02 |
| TSPAN5 | 2.34 | 1.95E-02 |
| SPAG5 | 2.34 | 5.63E-03 |
| FANCD2 | 2.34 | 3.88E-04 |
| MDK | 2.35 | 1.78E-04 |
| CTHRC1 | 2.35 | 2.33E-03 |
| PKP3 | 2.36 | 2.49E-03 |
| ORC6 | 2.36 | 2.40E-03 |
| CDC45 | 2.36 | 5.26E-03 |
| EFNA4 | 2.37 | 2.60E-03 |
| RHPN2 | 2.37 | 1.71E-02 |
| CDCP1 | 2.37 | 6.62E-03 |
| KCTD15 | 2.38 | 2.44E-03 |
| EGLN3 | 2.38 | 2.32E-02 |
| KIF15 | 2.40 | 1.75E-02 |
| TESMIN | 2.40 | 1.11E-02 |
| STARD10 | 2.40 | 5.36E-03 |
| SFN | 2.41 | 1.91E-02 |
| SHCBP1 | 2.41 | 1.24E-03 |
| TOM1L1 | 2.42 | 5.05E-03 |
| H2AC6 | 2.42 | 2.58E-03 |
| TK1 | 2.45 | 5.36E-03 |
| C3orf52 | 2.46 | 1.26E-02 |
| OAZ3 | 2.46 | 8.29E-03 |
| CD300LF | 2.46 | 6.23E-03 |
| NCEH1 | 2.46 | 1.55E-03 |
| KYNU | 2.46 | 2.90E-02 |
| MKI67 | 2.48 | 6.94E-04 |
| CDS1 | 2.48 | 8.18E-04 |
| MIF | 2.48 | 1.33E-05 |
| EPS8L1 | 2.48 | 7.73E-03 |
| CENPK | 2.49 | 1.09E-03 |
| IKZF3 | 2.49 | 3.60E-02 |
| TBC1D30 | 2.52 | 9.22E-03 |
| CGN | 2.52 | 7.58E-03 |
| ILDR1 | 2.52 | 1.51E-04 |
| MARVELD3 | 2.53 | 6.98E-04 |
| OVOL2 | 2.53 | 2.32E-03 |
| SELL | 2.55 | 4.62E-02 |
| IFI6 | 2.55 | 1.52E-02 |
| BAIAP2L1 | 2.55 | 4.57E-03 |
| FANCI | 2.56 | 2.45E-04 |
| FAM83B | 2.58 | 1.02E-02 |
| HSD17B6 | 2.58 | 1.03E-03 |
| SLC38A1 | 2.59 | 2.13E-03 |
| IRF6 | 2.59 | 1.20E-02 |
| IER3 | 2.60 | 2.65E-03 |
| NETO2 | 2.60 | 1.95E-02 |
| KIF23 | 2.61 | 6.10E-04 |
| DEGS2 | 2.61 | 8.51E-04 |
| TRAF4 | 2.62 | 5.71E-04 |
| PACC1 | 2.62 | 3.34E-05 |
| KIF14 | 2.62 | 3.87E-03 |
| CELSR1 | 2.63 | 7.65E-04 |
| SLC37A1 | 2.63 | 1.21E-04 |
| JPT1 | 2.63 | 9.94E-05 |
| ATAD2 | 2.63 | 1.09E-02 |
| RMI2 | 2.63 | 1.48E-03 |
| KNL1 | 2.64 | 9.28E-04 |
| ABHD17C | 2.64 | 4.41E-04 |
| OCLN | 2.65 | 4.02E-03 |
| TMEM97 | 2.65 | 8.48E-03 |
| NFE2L3 | 2.65 | 1.88E-02 |
| SORD | 2.67 | 1.43E-03 |
| TJP3 | 2.67 | 2.62E-04 |
| OIP5 | 2.67 | 2.02E-03 |
| MIAT | 2.68 | 5.34E-03 |
| H2BC4 | 2.69 | 4.47E-02 |
| NUP210 | 2.69 | 3.88E-03 |
| LSR | 2.69 | 7.12E-04 |
| MTFR2 | 2.70 | 2.81E-03 |
| TMEM45B | 2.70 | 4.73E-02 |
| RAB15 | 2.71 | 1.97E-04 |
| PHLDA2 | 2.71 | 5.64E-03 |
| MARVELD2 | 2.71 | 2.16E-03 |
| KLHDC7B | 2.72 | 1.90E-02 |
| SLC9A3R1 | 2.72 | 6.39E-03 |
| SULF1 | 2.73 | 2.17E-03 |
| TNFRSF12A | 2.73 | 2.32E-05 |
| GALE | 2.73 | 3.84E-04 |
| MEX3D | 2.74 | 1.46E-03 |
| SYCP2 | 2.74 | 2.07E-02 |
| TREM2 | 2.74 | 2.26E-03 |
| ECT2 | 2.75 | 2.43E-04 |
| CAPN13 | 2.75 | 4.41E-02 |
| ADAM8 | 2.76 | 8.35E-04 |
| RHOH | 2.77 | 3.67E-02 |
| CDCA5 | 2.77 | 4.23E-04 |
| CDCA3 | 2.78 | 5.45E-04 |
| HID1 | 2.79 | 5.42E-03 |
| MLPH | 2.80 | 1.63E-02 |
| FGFR3 | 2.80 | 1.80E-02 |
| CCR7 | 2.80 | 1.27E-02 |
| TMC4 | 2.80 | 1.64E-02 |
| HOPX | 2.81 | 2.04E-03 |
| E2F8 | 2.82 | 2.85E-03 |
| TMEM63C | 2.83 | 1.40E-03 |
| CHMP4C | 2.85 | 3.06E-02 |
| KIF26B | 2.85 | 9.44E-03 |
| SLAMF8 | 2.86 | 3.37E-03 |
| DUSP5 | 2.87 | 2.68E-03 |
| CLDN7 | 2.87 | 1.21E-03 |
| LRRC8E | 2.87 | 4.49E-04 |
| ENPP5 | 2.88 | 7.18E-03 |
| GATA3 | 2.88 | 1.07E-02 |
| TSPAN1 | 2.88 | 4.56E-02 |
| IL1RN | 2.88 | 1.93E-02 |
| TRIM59 | 2.89 | 7.38E-06 |
| DEPDC1 | 2.90 | 2.86E-03 |
| HMGB3 | 2.90 | 1.08E-03 |
| APOBEC3B | 2.91 | 6.00E-04 |
| MANEAL | 2.92 | 6.13E-03 |
| FOXM1 | 2.92 | 2.33E-03 |
| CASZ1 | 2.93 | 1.06E-04 |
| FCGR1B | 2.95 | 4.62E-03 |
| RAB27B | 2.95 | 1.40E-03 |
| SYNE4 | 2.96 | 1.21E-03 |
| H2BS1 | 2.96 | 2.62E-03 |
| CLEC7A | 2.97 | 1.38E-04 |
| SYTL1 | 2.97 | 1.20E-03 |
| LMNB1 | 2.99 | 3.34E-05 |
| MFSD2A | 3.02 | 6.62E-03 |
| BSPRY | 3.04 | 1.99E-03 |
| FA2H | 3.04 | 1.43E-02 |
| SAMD12 | 3.05 | 1.61E-03 |
| SAPCD2 | 3.05 | 5.11E-03 |
| CLDN3 | 3.06 | 5.49E-03 |
| SELENOI | 3.07 | 2.75E-04 |
| ANKRD22 | 3.07 | 4.84E-03 |
| IRX5 | 3.07 | 1.84E-03 |
| DBNDD1 | 3.09 | 6.93E-04 |
| C3orf14 | 3.09 | 8.83E-05 |
| RAB26 | 3.10 | 7.68E-03 |
| TRIB3 | 3.10 | 3.88E-04 |
| SLC7A8 | 3.10 | 1.43E-03 |
| H2BC6 | 3.10 | 9.62E-04 |
| ELF3 | 3.10 | 4.83E-03 |
| SBK1 | 3.11 | 3.64E-04 |
| NPNT | 3.11 | 3.01E-03 |
| TTK | 3.12 | 3.64E-03 |
| CBX2 | 3.12 | 8.94E-03 |
| GALNT7 | 3.12 | 8.72E-04 |
| RNF43 | 3.12 | 1.24E-02 |
| TTC39A | 3.13 | 2.82E-03 |
| RAD51AP1 | 3.13 | 2.79E-05 |
| GALNT3 | 3.13 | 1.77E-02 |
| PRLR | 3.13 | 8.85E-05 |
| F12 | 3.14 | 4.23E-04 |
| CHI3L1 | 3.14 | 4.31E-02 |
| CRABP2 | 3.15 | 5.04E-05 |
| GPR160 | 3.16 | 3.30E-03 |
| NDC80 | 3.17 | 8.35E-04 |
| LEF1 | 3.17 | 9.29E-05 |
| BUB1 | 3.17 | 1.52E-03 |
| FAM110A | 3.17 | 2.12E-05 |
| BCL2A1 | 3.17 | 1.25E-03 |
| HOOK1 | 3.18 | 4.00E-03 |
| PLA2G7 | 3.20 | 1.71E-03 |
| SSX2IP | 3.20 | 2.95E-06 |
| CCNA2 | 3.22 | 2.52E-04 |
| H2AC8 | 3.22 | 1.85E-02 |
| IQGAP3 | 3.22 | 1.76E-03 |
| KIF20A | 3.22 | 4.62E-04 |
| SPAG1 | 3.23 | 2.54E-04 |
| LOC285097 | 3.23 | 2.68E-02 |
| MYO5B | 3.23 | 3.68E-04 |
| TYMS | 3.24 | 2.11E-04 |
| CENPE | 3.24 | 4.53E-04 |
| KRT8 | 3.26 | 1.89E-04 |
| PKIB | 3.26 | 1.01E-02 |
| SRD5A1 | 3.27 | 5.58E-04 |
| SPINT1 | 3.27 | 1.20E-03 |
| LAD1 | 3.27 | 4.97E-03 |
| FCMR | 3.30 | 3.65E-03 |
| CXCR4 | 3.31 | 9.94E-05 |
| CEP55 | 3.31 | 6.14E-04 |
| SLC16A6 | 3.32 | 3.31E-02 |
| PAFAH1B3 | 3.32 | 2.12E-05 |
| IGSF9 | 3.33 | 2.31E-04 |
| PLPP2 | 3.34 | 3.23E-03 |
| GINS1 | 3.34 | 6.65E-04 |
| CD24 | 3.34 | 1.65E-03 |
| GDF15 | 3.35 | 2.70E-02 |
| RARRES1 | 3.36 | 8.47E-03 |
| DSP | 3.37 | 1.03E-02 |
| CENPU | 3.37 | 9.14E-04 |
| TLCD1 | 3.38 | 2.20E-03 |
| PPP2R2C | 3.39 | 1.58E-02 |
| MAPK13 | 3.39 | 8.95E-05 |
| BAMBI | 3.40 | 9.66E-03 |
| H4C8 | 3.40 | 9.72E-03 |
| NUF2 | 3.40 | 8.64E-04 |
| MAD2L1 | 3.41 | 2.71E-04 |
| C9orf152 | 3.41 | 2.87E-02 |
| TMEM132A | 3.41 | 6.26E-04 |
| VAV3 | 3.48 | 7.65E-04 |
| DLGAP5 | 3.48 | 3.30E-04 |
| DIO2 | 3.49 | 9.36E-03 |
| ATP2C2 | 3.49 | 6.89E-03 |
| TACSTD2 | 3.51 | 1.31E-02 |
| CXCL8 | 3.51 | 3.36E-02 |
| EPB41L5 | 3.51 | 6.83E-04 |
| MARCKSL1 | 3.52 | 2.27E-05 |
| SEPTIN6 | 3.53 | 7.45E-05 |
| ARFGEF3 | 3.54 | 3.24E-04 |
| TRIP13 | 3.56 | 7.42E-04 |
| MB | 3.56 | 4.40E-04 |
| F2RL2 | 3.56 | 4.67E-03 |
| MYB | 3.57 | 2.11E-03 |
| AP1M2 | 3.57 | 2.98E-04 |
| TPD52 | 3.58 | 7.40E-04 |
| LAMP5 | 3.62 | 1.79E-03 |
| CDC20 | 3.62 | 1.82E-03 |
| FAM83H | 3.62 | 2.08E-04 |
| CORO2A | 3.65 | 3.44E-05 |
| ERBB2 | 3.66 | 3.15E-02 |
| MLLT11 | 3.68 | 2.49E-03 |
| RGS1 | 3.69 | 9.29E-04 |
| SERINC2 | 3.70 | 4.08E-04 |
| KIF11 | 3.70 | 2.57E-04 |
| KIF2C | 3.71 | 9.88E-04 |
| MMP1 | 3.71 | 3.90E-02 |
| HMMR | 3.74 | 8.23E-04 |
| CREB3L4 | 3.74 | 2.62E-04 |
| PROM2 | 3.74 | 1.20E-03 |
| SPINT2 | 3.75 | 4.20E-04 |
| GPRC5A | 3.77 | 3.04E-02 |
| KRT18 | 3.77 | 1.82E-03 |
| MMP9 | 3.79 | 1.25E-03 |
| SQLE | 3.79 | 7.62E-05 |
| INHBA | 3.85 | 2.18E-05 |
| CCNE2 | 3.86 | 1.66E-04 |
| GINS2 | 3.87 | 2.00E-03 |
| KIF4A | 3.87 | 2.32E-04 |
| FAM110C | 3.88 | 1.58E-04 |
| CXADR | 3.88 | 1.17E-02 |
| CKS2 | 3.89 | 5.39E-05 |
| ERP27 | 3.93 | 2.39E-03 |
| CDK1 | 3.94 | 1.65E-04 |
| ZG16B | 3.95 | 1.03E-02 |
| ZWINT | 3.95 | 1.97E-04 |
| GALNT6 | 3.97 | 9.56E-03 |
| CCN4 | 3.97 | 6.85E-05 |
| CENPF | 3.98 | 3.78E-05 |
| SLC12A8 | 3.99 | 1.63E-05 |
| MMP3 | 3.99 | 3.76E-03 |
| PRR15L | 4.00 | 9.37E-04 |
| PTTG1 | 4.03 | 6.28E-05 |
| EPPK1 | 4.04 | 4.05E-03 |
| TFAP2A | 4.04 | 4.10E-03 |
| INAVA | 4.06 | 2.26E-03 |
| LOC100130449 | 4.07 | 5.54E-03 |
| BIK | 4.08 | 6.32E-05 |
| CCNB1 | 4.08 | 3.78E-05 |
| PRR15 | 4.10 | 3.56E-03 |
| PRSS8 | 4.10 | 3.25E-04 |
| H2BC21 | 4.11 | 3.58E-04 |
| SLC7A5 | 4.13 | 1.18E-04 |
| LAMP3 | 4.15 | 1.97E-04 |
| H2BC5 | 4.16 | 2.78E-03 |
| HOTAIR | 4.18 | 1.08E-05 |
| ALDH3B2 | 4.21 | 1.47E-03 |
| TDO2 | 4.22 | 2.75E-03 |
| CYP4Z2P | 4.23 | 3.77E-03 |
| PRC1 | 4.24 | 7.40E-05 |
| C15orf48 | 4.25 | 3.31E-04 |
| PBK | 4.25 | 4.52E-04 |
| EZH2 | 4.26 | 6.13E-06 |
| CDKN3 | 4.26 | 1.03E-04 |
| H2BC9 | 4.26 | 1.97E-04 |
| UHRF1 | 4.29 | 2.12E-05 |
| CXCL11 | 4.29 | 1.59E-02 |
| MELK | 4.34 | 7.92E-05 |
| UBE2T | 4.34 | 3.79E-04 |
| BUB1B | 4.35 | 2.12E-05 |
| TOX3 | 4.36 | 4.21E-03 |
| LRRC15 | 4.36 | 2.52E-04 |
| CXCL10 | 4.37 | 9.84E-03 |
| VMP1 | 4.38 | 3.14E-04 |
| HOXC10 | 4.38 | 4.39E-06 |
| AURKA | 4.39 | 3.66E-05 |
| ERBB3 | 4.39 | 1.83E-03 |
| KIAA1324 | 4.40 | 3.38E-03 |
| ANLN | 4.40 | 1.15E-04 |
| UBE2C | 4.41 | 5.27E-05 |
| CLGN | 4.46 | 2.91E-03 |
| TPX2 | 4.51 | 4.45E-04 |
| SPDEF | 4.51 | 3.85E-04 |
| CDH1 | 4.51 | 6.54E-03 |
| CCNB2 | 4.53 | 4.69E-05 |
| FXYD3 | 4.54 | 2.45E-03 |
| TFAP2B | 4.55 | 4.34E-02 |
| DTL | 4.55 | 3.12E-04 |
| FAM83D | 4.64 | 4.63E-04 |
| GJB2 | 4.68 | 4.52E-04 |
| ESRP1 | 4.72 | 6.15E-04 |
| MUC1 | 4.73 | 3.19E-04 |
| RAB25 | 4.76 | 7.49E-04 |
| NUSAP1 | 4.79 | 2.12E-05 |
| CYP4Z1 | 4.80 | 2.81E-02 |
| BIRC5 | 4.81 | 2.01E-05 |
| KRT19 | 4.81 | 4.50E-03 |
| NEK2 | 4.89 | 8.63E-05 |
| ADAMDEC1 | 4.90 | 1.73E-03 |
| MMP11 | 5.00 | 2.50E-05 |
| TOP2A | 5.02 | 1.63E-03 |
| ASPM | 5.06 | 4.92E-05 |
| FOXA1 | 5.09 | 1.55E-03 |
| S100A14 | 5.11 | 1.64E-03 |
| TMC5 | 5.12 | 4.84E-03 |
| AGR2 | 5.20 | 2.57E-02 |
| SDC1 | 5.21 | 1.97E-04 |
| COMP | 5.28 | 5.62E-06 |
| SLC44A4 | 5.30 | 2.69E-04 |
| ST8SIA6-AS1 | 5.31 | 1.40E-03 |
| EPCAM | 5.33 | 8.15E-04 |
| SPP1 | 5.36 | 1.21E-05 |
| PCLAF | 5.37 | 8.62E-05 |
| MMP12 | 5.42 | 1.26E-02 |
| COL11A1 | 5.68 | 1.54E-03 |
| KMO | 5.83 | 8.39E-05 |
| CEACAM6 | 6.64 | 1.35E-03 |
| RRM2 | 6.79 | 4.30E-06 |
| COL10A1 | 7.17 | 1.39E-07 |
| S100P | 7.83 | 4.76E-05 |
