# Supplementary material for: Identification of hub genes and small molecule therapeutic drugs related to breast cancer with comprehensive bioinformatics analysis
Source: PeerJ. 2020 Sep 29;8:e9946. doi: 10.7717/peerj.9946 (PMC7556247; doi:10.7717/peerj.9946)
Supplement: Supplemental Information 12 [file peerj-08-9946-s012.docx]

| **ID** | **Log2FC** | **adj.P.Val** |
| --- | --- | --- |
| KRT19 | 5.52 | 2.55E-15 |
| COL11A1 | 5.29 | 2.70E-12 |
| EPCAM | 5.18 | 3.16E-21 |
| EPPK1 | 5.01 | 6.08E-16 |
| DSP | 4.97 | 3.23E-25 |
| KRT18 | 4.96 | 1.77E-18 |
| CDH1 | 4.94 | 1.78E-18 |
| ESRP1 | 4.85 | 3.87E-23 |
| TFAP2A | 4.64 | 7.77E-19 |
| ERBB3 | 4.58 | 1.86E-17 |
| TACSTD2 | 4.57 | 3.20E-14 |
| MUC1 | 4.48 | 3.71E-12 |
| TOP2A | 4.44 | 1.32E-15 |
| RRM2 | 4.33 | 1.04E-16 |
| CD24 | 4.31 | 1.88E-16 |
| AGR2 | 4.30 | 1.22E-06 |
| S100P | 4.27 | 2.48E-08 |
| SDC1 | 4.26 | 1.44E-19 |
| RAB25 | 4.16 | 4.49E-19 |
| SPINT2 | 4.14 | 2.00E-26 |
| SCNN1A | 4.10 | 2.04E-10 |
| C15orf48 | 4.07 | 3.56E-11 |
| PROM2 | 4.03 | 6.25E-16 |
| KIAA1324 | 3.98 | 3.20E-09 |
| ZG16B | 3.93 | 1.09E-10 |
| INHBA | 3.92 | 5.60E-18 |
| KRT8 | 3.91 | 8.80E-19 |
| FAM83H | 3.83 | 2.12E-22 |
| CELSR1 | 3.81 | 9.16E-14 |
| TMC4 | 3.80 | 1.65E-13 |
| PRSS8 | 3.79 | 1.79E-19 |
| FOXA1 | 3.78 | 1.39E-08 |
| PCLAF | 3.74 | 9.61E-16 |
| GATA3 | 3.74 | 5.20E-11 |
| TPD52 | 3.74 | 2.52E-19 |
| SFN | 3.71 | 2.56E-16 |
| KRT7 | 3.69 | 1.95E-07 |
| PAFAH1B3 | 3.69 | 2.93E-21 |
| BAIAP2L1 | 3.69 | 1.05E-15 |
| CDC20 | 3.68 | 4.54E-15 |
| EZH2 | 3.66 | 5.96E-19 |
| GALNT6 | 3.65 | 4.16E-12 |
| EHF | 3.65 | 7.73E-09 |
| NUSAP1 | 3.63 | 1.30E-17 |
| MYB | 3.62 | 8.93E-10 |
| SLC38A1 | 3.57 | 3.88E-17 |
| LRRC15 | 3.55 | 1.88E-12 |
| AURKA | 3.53 | 2.38E-15 |
| GPRC5A | 3.52 | 1.21E-08 |
| SYNE4 | 3.50 | 6.45E-15 |
| VAV3 | 3.50 | 1.44E-12 |
| COL10A1 | 3.49 | 1.51E-11 |
| C11orf80 | 3.48 | 1.11E-18 |
| AP1M2 | 3.47 | 5.04E-16 |
| CREB3L4 | 3.42 | 2.67E-12 |
| UHRF1 | 3.40 | 1.14E-18 |
| COMP | 3.39 | 9.58E-10 |
| MLPH | 3.37 | 5.10E-06 |
| NPNT | 3.33 | 5.70E-08 |
| MMP11 | 3.31 | 8.83E-11 |
| CEACAM6 | 3.30 | 8.16E-05 |
| IGSF3 | 3.29 | 2.98E-14 |
| IRF6 | 3.28 | 1.63E-15 |
| SERPINA3 | 3.28 | 7.68E-06 |
| S100A14 | 3.27 | 6.54E-12 |
| PRC1 | 3.26 | 1.89E-11 |
| TMC5 | 3.24 | 1.08E-05 |
| PARD6B | 3.23 | 2.03E-09 |
| SMIM22 | 3.21 | 1.10E-09 |
| BSPRY | 3.20 | 2.28E-14 |
| ANLN | 3.19 | 2.80E-11 |
| BMPR1B | 3.18 | 1.19E-03 |
| CXADR | 3.17 | 7.83E-10 |
| TOX3 | 3.17 | 2.65E-06 |
| FXYD3 | 3.16 | 2.10E-11 |
| CLDN3 | 3.16 | 3.82E-11 |
| MARVELD2 | 3.16 | 4.02E-16 |
| STARD10 | 3.15 | 1.01E-12 |
| ANKRD30A | 3.15 | 1.45E-03 |
| PRLR | 3.14 | 4.52E-10 |
| EPN3 | 3.14 | 3.63E-09 |
| MMP1 | 3.12 | 7.93E-05 |
| SQLE | 3.11 | 6.09E-13 |
| PABPC1L | 3.09 | 4.02E-11 |
| CHMP4C | 3.09 | 5.33E-12 |
| NEBL | 3.08 | 2.94E-10 |
| CA12 | 3.08 | 1.44E-05 |
| TMEM125 | 3.06 | 3.35E-13 |
| GINS1 | 3.05 | 9.89E-15 |
| ERBB2 | 3.05 | 9.56E-08 |
| IQGAP3 | 3.04 | 4.48E-12 |
| EPS8L1 | 3.04 | 1.58E-12 |
| TJP3 | 3.04 | 9.28E-12 |
| CRABP2 | 3.02 | 9.06E-09 |
| RAB11FIP3 | 3.02 | 6.97E-14 |
| SYTL1 | 3.01 | 6.86E-14 |
| TFF1 | 2.99 | 5.20E-03 |
| AGR3 | 2.97 | 3.26E-03 |
| TRPS1 | 2.96 | 1.14E-14 |
| RAB27B | 2.96 | 8.17E-08 |
| CDS1 | 2.95 | 3.99E-18 |
| SPINT1 | 2.95 | 2.61E-13 |
| CFB | 2.93 | 2.97E-06 |
| H2BC5 | 2.93 | 5.04E-09 |
| THSD4 | 2.93 | 9.02E-07 |
| FBXL16 | 2.93 | 1.48E-09 |
| EFHD1 | 2.92 | 1.32E-07 |
| MARCKSL1 | 2.91 | 9.38E-19 |
| LSR | 2.89 | 6.31E-16 |
| SHROOM3 | 2.88 | 3.68E-10 |
| CYB561 | 2.88 | 7.70E-18 |
| ALDH3B2 | 2.87 | 1.71E-05 |
| CDCA3 | 2.87 | 7.61E-11 |
| SPP1 | 2.87 | 1.08E-05 |
| TFAP2C | 2.87 | 3.48E-09 |
| CENPF | 2.86 | 2.48E-14 |
| GJB2 | 2.85 | 9.33E-08 |
| LRRC1 | 2.84 | 2.32E-19 |
| PDCD6 | 2.84 | 2.37E-15 |
| IRX3 | 2.83 | 3.71E-11 |
| F12 | 2.82 | 9.70E-11 |
| GALNT3 | 2.82 | 4.85E-06 |
| FBXO32 | 2.82 | 8.25E-09 |
| PTTG1 | 2.82 | 2.69E-11 |
| CCNE2 | 2.81 | 3.31E-12 |
| LTF | 2.81 | 2.29E-03 |
| CASZ1 | 2.80 | 1.38E-11 |
| IRX5 | 2.80 | 6.30E-09 |
| DTL | 2.79 | 4.77E-15 |
| HOOK1 | 2.78 | 1.04E-14 |
| COL4A5 | 2.77 | 3.43E-09 |
| OVOL2 | 2.77 | 4.24E-17 |
| ATP1B1 | 2.75 | 8.68E-09 |
| ZWINT | 2.73 | 3.92E-14 |
| MYO5B | 2.72 | 7.04E-16 |
| TYMS | 2.72 | 1.58E-10 |
| GRHL1 | 2.71 | 5.43E-09 |
| FGFR3 | 2.71 | 3.19E-06 |
| LLGL2 | 2.70 | 9.38E-19 |
| ANKRD10-IT1 | 2.70 | 5.51E-17 |
| RHPN2 | 2.69 | 6.87E-11 |
| OCLN | 2.69 | 8.16E-15 |
| SPDEF | 2.68 | 6.38E-07 |
| MELK | 2.67 | 2.22E-09 |
| CKS2 | 2.66 | 1.91E-12 |
| LRATD2 | 2.66 | 5.01E-14 |
| DLG3 | 2.65 | 1.34E-19 |
| KIF2C | 2.65 | 7.21E-09 |
| SOX4 | 2.64 | 2.04E-17 |
| VDR | 2.64 | 5.08E-17 |
| GINS2 | 2.64 | 4.43E-12 |
| LINC00993 | 2.64 | 4.80E-03 |
| SLITRK6 | 2.63 | 1.37E-03 |
| APOBEC3B | 2.63 | 4.11E-08 |
| LINC00537 | 2.63 | 3.60E-07 |
| UBE2C | 2.63 | 2.84E-11 |
| CXCL10 | 2.63 | 6.26E-05 |
| CDH3 | 2.62 | 3.16E-06 |
| SULF1 | 2.61 | 7.82E-09 |
| EPB41L5 | 2.61 | 1.35E-12 |
| CDK1 | 2.61 | 6.43E-11 |
| MICAL2 | 2.61 | 9.06E-14 |
| ST14 | 2.61 | 1.84E-14 |
| VTCN1 | 2.60 | 2.22E-05 |
| WDR90 | 2.60 | 1.45E-14 |
| TMEM97 | 2.60 | 1.88E-09 |
| EFNA1 | 2.60 | 4.71E-15 |
| TFAP2B | 2.59 | 2.20E-02 |
| SLC7A5 | 2.58 | 2.58E-06 |
| SORD | 2.58 | 5.87E-10 |
| ANKRD13D | 2.58 | 5.24E-12 |
| PRR15L | 2.57 | 5.12E-09 |
| ARFGEF3 | 2.55 | 4.89E-07 |
| EPS8L2 | 2.55 | 9.70E-15 |
| RUSC1-AS1 | 2.55 | 5.27E-12 |
| CD9 | 2.54 | 1.68E-07 |
| XBP1 | 2.54 | 1.66E-06 |
| PRKCZ | 2.54 | 4.91E-23 |
| LRRC8E | 2.52 | 1.36E-11 |
| MAPK13 | 2.52 | 1.35E-13 |
| IER3 | 2.52 | 8.97E-13 |
| ISG15 | 2.51 | 1.58E-07 |
| SERINC2 | 2.50 | 1.72E-13 |
| CBX4 | 2.50 | 2.65E-18 |
| BAMBI | 2.50 | 3.62E-06 |
| CEP55 | 2.49 | 4.14E-08 |
| SDC4 | 2.49 | 9.65E-15 |
| LOC100190986 | 2.48 | 1.84E-14 |
| GSTO2 | 2.48 | 8.07E-08 |
| LOC285097 | 2.48 | 4.69E-05 |
| TMED3 | 2.47 | 2.77E-16 |
| FN1 | 2.47 | 2.47E-10 |
| GRHL2 | 2.47 | 2.65E-17 |
| STAP2 | 2.47 | 8.74E-15 |
| SLC16A6 | 2.46 | 1.37E-05 |
| HOXC10 | 2.46 | 1.62E-06 |
| TRIB3 | 2.46 | 1.28E-10 |
| BUB1B | 2.45 | 6.30E-09 |
| MMP9 | 2.45 | 4.23E-04 |
| KCNK1 | 2.44 | 3.60E-06 |
| PHLDA2 | 2.44 | 4.85E-07 |
| CENPU | 2.44 | 1.44E-12 |
| KIAA1522 | 2.44 | 1.24E-14 |
| SOX9 | 2.43 | 1.94E-08 |
| TNNT1 | 2.43 | 3.33E-04 |
| FAM83D | 2.43 | 2.11E-08 |
| TRAF4 | 2.42 | 4.35E-15 |
| TPBG | 2.42 | 3.80E-11 |
| SHANK2 | 2.42 | 7.52E-08 |
| SEMA3F | 2.42 | 6.91E-10 |
| FANCI | 2.41 | 7.38E-15 |
| HMGB3 | 2.41 | 6.25E-12 |
| CCN4 | 2.41 | 3.79E-09 |
| CCNB1 | 2.40 | 2.38E-08 |
| CDCP1 | 2.40 | 8.36E-11 |
| CAPN8 | 2.40 | 5.02E-04 |
| SHISA2 | 2.40 | 5.48E-04 |
| MB | 2.39 | 3.83E-08 |
| POSTN | 2.39 | 1.26E-05 |
| MARVELD3 | 2.39 | 7.70E-13 |
| ANKRD36B | 2.39 | 7.26E-10 |
| PPP1R13L | 2.38 | 2.18E-12 |
| S100A7 | 2.38 | 2.13E-02 |
| LIMK2 | 2.38 | 1.67E-16 |
| PLPP2 | 2.38 | 7.40E-11 |
| CGN | 2.36 | 2.68E-12 |
| NUF2 | 2.35 | 7.12E-08 |
| MCM2 | 2.35 | 1.53E-13 |
| RTKN | 2.35 | 3.80E-13 |
| PKIB | 2.35 | 6.60E-05 |
| ADGRG1 | 2.34 | 6.17E-10 |
| FLVCR1 | 2.34 | 6.67E-21 |
| PAQR4 | 2.34 | 3.76E-14 |
| DNAJC12 | 2.34 | 1.80E-03 |
| KCTD15 | 2.33 | 1.47E-11 |
| CTHRC1 | 2.32 | 9.59E-08 |
| SFI1 | 2.32 | 2.18E-08 |
| COBL | 2.31 | 1.87E-07 |
| ARNT2 | 2.31 | 4.18E-06 |
| CBFA2T2 | 2.30 | 4.35E-16 |
| CACNB3 | 2.30 | 8.42E-15 |
| COL27A1 | 2.30 | 4.89E-06 |
| KRTCAP3 | 2.29 | 1.53E-08 |
| LEF1 | 2.29 | 1.15E-07 |
| PDZK1IP1 | 2.29 | 7.95E-05 |
| RIPK4 | 2.29 | 7.04E-08 |
| MDK | 2.28 | 1.26E-07 |
| TTC39A | 2.27 | 3.86E-06 |
| FANCD2 | 2.27 | 2.69E-13 |
| RAB26 | 2.27 | 1.44E-08 |
| TPX2 | 2.26 | 3.26E-08 |
| CHTF18 | 2.26 | 1.05E-12 |
| PRRG4 | 2.26 | 6.43E-11 |
| ANK3 | 2.25 | 3.57E-08 |
| AGRN | 2.25 | 1.22E-15 |
| GALNT7 | 2.24 | 8.36E-08 |
| BIK | 2.24 | 5.81E-08 |
| SAPCD2 | 2.24 | 1.04E-10 |
| FKBP4 | 2.23 | 2.75E-12 |
| NEK2 | 2.22 | 1.13E-09 |
| MYO5C | 2.22 | 9.52E-10 |
| EME2 | 2.22 | 4.24E-09 |
| KIF18B | 2.22 | 2.79E-09 |
| ELF3 | 2.22 | 6.81E-10 |
| CLDN4 | 2.22 | 1.09E-13 |
| HMMR | 2.21 | 3.44E-08 |
| SLC35A2 | 2.21 | 3.40E-17 |
| EPHB4 | 2.21 | 6.64E-12 |
| CCNB2 | 2.21 | 7.21E-09 |
| REEP1 | 2.20 | 1.75E-05 |
| SHB | 2.20 | 4.45E-13 |
| TMEM30B | 2.20 | 1.45E-08 |
| LAPTM4B | 2.20 | 3.97E-07 |
| TK1 | 2.19 | 3.61E-09 |
| FOXO6 | 2.19 | 1.25E-10 |
| SLC9A3R1 | 2.19 | 3.41E-09 |
| KIF20A | 2.19 | 1.82E-09 |
| SIX4 | 2.19 | 4.74E-11 |
| ZNF692 | 2.18 | 7.24E-15 |
| WDR34 | 2.18 | 3.65E-17 |
| CANT1 | 2.18 | 1.19E-13 |
| PATJ | 2.17 | 9.98E-10 |
| BGN | 2.17 | 6.39E-09 |
| BIRC5 | 2.17 | 1.10E-06 |
| SELENOI | 2.17 | 7.85E-11 |
| MYO6 | 2.16 | 1.37E-09 |
| LPAR2 | 2.15 | 8.44E-14 |
| NAT14 | 2.15 | 2.49E-13 |
| C19orf33 | 2.15 | 2.81E-03 |
| TFF3 | 2.15 | 1.37E-02 |
| MAGED2 | 2.15 | 2.38E-06 |
| SLC12A8 | 2.15 | 1.46E-08 |
| TRA2A | 2.14 | 6.18E-14 |
| FTX | 2.14 | 2.86E-13 |
| EZR | 2.14 | 1.51E-14 |
| AUTS2 | 2.13 | 2.55E-10 |
| SYCP2 | 2.13 | 1.83E-07 |
| FANK1 | 2.13 | 4.44E-07 |
| TMEM132A | 2.13 | 1.99E-09 |
| RPL32P3 | 2.13 | 2.32E-14 |
| IFI6 | 2.13 | 5.86E-05 |
| TTK | 2.12 | 1.59E-08 |
| MTHFD2 | 2.12 | 4.16E-16 |
| FAM110C | 2.12 | 6.83E-06 |
| TRMT13 | 2.11 | 1.78E-11 |
| GOLM1 | 2.11 | 1.54E-05 |
| CACNA1D | 2.11 | 1.19E-04 |
| RABEP2 | 2.11 | 3.76E-11 |
| BARD1 | 2.10 | 2.37E-12 |
| ERP27 | 2.10 | 1.52E-04 |
| POTEM | 2.10 | 9.41E-04 |
| ASPM | 2.09 | 2.62E-08 |
| SOX11 | 2.09 | 4.71E-03 |
| SH3BP4 | 2.09 | 5.88E-08 |
| TET3 | 2.09 | 5.46E-18 |
| SLC7A1 | 2.09 | 2.20E-11 |
| ZNF512B | 2.09 | 1.37E-13 |
| WASIR2 | 2.09 | 7.64E-06 |
| IRX2 | 2.08 | 5.03E-04 |
| MGAT4A | 2.08 | 3.07E-07 |
| ANXA9 | 2.08 | 3.23E-04 |
| SMARCA4 | 2.08 | 7.19E-20 |
| STK26 | 2.07 | 4.55E-08 |
| RHOH | 2.07 | 1.13E-04 |
| ENPP5 | 2.07 | 6.28E-04 |
| AP1G2 | 2.07 | 8.37E-15 |
| HELLS | 2.06 | 1.12E-11 |
| MSI2 | 2.06 | 4.13E-10 |
| MIF | 2.05 | 3.70E-15 |
| SSX2IP | 2.05 | 2.73E-10 |
| C1orf116 | 2.05 | 7.83E-05 |
| CALML5 | 2.04 | 4.69E-03 |
| ATP2A2 | 2.04 | 6.62E-17 |
| SLC39A6 | 2.04 | 4.57E-06 |
| KNL1 | 2.04 | 2.07E-09 |
| KRT15 | 2.03 | 1.16E-02 |
| MICALL2 | 2.03 | 3.14E-11 |
| ORC6 | 2.03 | 2.96E-08 |
| ANO1 | 2.03 | 4.19E-05 |
| STIL | 2.02 | 5.92E-12 |
| ALCAM | 2.02 | 5.20E-06 |
| KNOP1 | 2.02 | 2.42E-08 |
| C3orf14 | 2.02 | 1.37E-07 |
| F2RL2 | 2.01 | 2.10E-05 |
| TNC | 2.01 | 1.51E-04 |
| STC2 | 2.01 | 4.47E-03 |
| SLC25A29 | 2.00 | 1.56E-07 |
| LY6E | 2.00 | 9.16E-06 |
| PPM1H | 2.00 | 5.55E-07 |
| TRIP13 | 2.00 | 6.21E-06 |
| DLGAP5 | 1.99 | 4.59E-07 |
| H2BC21 | 1.99 | 3.20E-04 |
| WWC1 | 1.99 | 4.76E-11 |
| NOL4L | 1.99 | 7.37E-12 |
| GALE | 1.98 | 4.29E-10 |
| IGF1R | 1.98 | 2.13E-04 |
| RAB15 | 1.98 | 2.77E-14 |
| ZNF827 | 1.98 | 9.99E-08 |
| TTC22 | 1.98 | 2.05E-05 |
| MAP7 | 1.97 | 3.94E-08 |
| RCC2 | 1.97 | 4.48E-19 |
| PKM | 1.97 | 8.03E-15 |
| PIK3R3 | 1.97 | 2.91E-09 |
| POGK | 1.97 | 5.00E-10 |
| RAD51AP1 | 1.96 | 8.40E-08 |
| KIF26B | 1.96 | 1.35E-08 |
| CLGN | 1.96 | 8.88E-04 |
| DHRS13 | 1.96 | 2.48E-07 |
| ECT2 | 1.96 | 4.01E-11 |
| SINHCAF | 1.96 | 1.20E-08 |
| ATP1A1 | 1.96 | 6.00E-14 |
| UBE2T | 1.96 | 3.03E-07 |
| NAT1 | 1.96 | 3.26E-02 |
| ARHGAP32 | 1.95 | 9.50E-08 |
| CRACR2B | 1.95 | 4.02E-06 |
| ZBTB42 | 1.95 | 8.87E-10 |
| EFNA4 | 1.95 | 4.06E-10 |
| C3orf52 | 1.94 | 3.78E-07 |
| TTC6 | 1.93 | 3.15E-04 |
| TSPAN13 | 1.93 | 3.34E-12 |
| PRR15 | 1.93 | 2.16E-05 |
| MCM4 | 1.93 | 7.02E-13 |
| TRIM59 | 1.93 | 5.07E-10 |
| WDR54 | 1.92 | 1.74E-09 |
| SRD5A1 | 1.92 | 3.76E-04 |
| TSTA3 | 1.91 | 5.92E-12 |
| TIAM1 | 1.91 | 1.40E-05 |
| PADI2 | 1.91 | 1.22E-03 |
| PPAT | 1.90 | 3.75E-10 |
| ITPR3 | 1.90 | 5.01E-09 |
| SYNJ2 | 1.90 | 1.93E-07 |
| FARP1 | 1.89 | 1.78E-08 |
| CYP2B7P | 1.89 | 2.20E-02 |
| CYP4Z1 | 1.89 | 3.17E-02 |
| MX1 | 1.89 | 8.55E-04 |
| FAM174B | 1.89 | 2.02E-08 |
| LRP2 | 1.89 | 1.66E-02 |
| TYMP | 1.89 | 2.15E-07 |
| MXRA5 | 1.89 | 1.90E-07 |
| SLC52A2 | 1.89 | 2.52E-12 |
| AQP3 | 1.89 | 6.49E-03 |
| RORC | 1.88 | 3.28E-05 |
| RUSC1 | 1.88 | 6.05E-08 |
| ARMC9 | 1.88 | 1.92E-09 |
| SEC61A1 | 1.87 | 3.69E-12 |
| CXCL9 | 1.87 | 2.01E-02 |
| KHDC4 | 1.87 | 1.02E-15 |
| CKAP4 | 1.87 | 6.18E-17 |
| RAB3D | 1.86 | 1.27E-10 |
| SULF2 | 1.86 | 7.49E-08 |
| KIF4A | 1.86 | 6.24E-07 |
| TBC1D30 | 1.86 | 6.83E-06 |
| KDM4B | 1.86 | 2.96E-08 |
| BICDL2 | 1.86 | 1.72E-11 |
| RSPH1 | 1.86 | 2.27E-06 |
| LAMA5 | 1.86 | 1.74E-10 |
| SYTL2 | 1.86 | 1.41E-04 |
| JUP | 1.85 | 1.99E-12 |
| MYBL1 | 1.85 | 1.17E-03 |
| PRXL2B | 1.85 | 2.35E-11 |
| MBOAT2 | 1.85 | 5.70E-08 |
| GART | 1.85 | 3.02E-09 |
| IGFBP2 | 1.84 | 4.10E-04 |
| OCIAD2 | 1.84 | 6.64E-10 |
| PEG10 | 1.84 | 2.11E-02 |
| TSPAN5 | 1.83 | 1.34E-05 |
| HLA-DRB4 | 1.83 | 4.91E-02 |
| AKAP10 | 1.83 | 6.75E-10 |
| INAVA | 1.83 | 2.68E-04 |
| DEGS2 | 1.82 | 8.45E-05 |
| HES1 | 1.82 | 3.82E-06 |
| NECTIN4 | 1.82 | 4.45E-08 |
| CDC25B | 1.82 | 6.32E-07 |
| ARRDC1 | 1.82 | 5.02E-12 |
| SEMA4C | 1.82 | 4.38E-11 |
| SYMPK | 1.82 | 3.88E-10 |
| JPT2 | 1.82 | 3.32E-14 |
| FLNB | 1.82 | 7.44E-09 |
| JPT1 | 1.81 | 5.13E-10 |
| WNK4 | 1.81 | 2.12E-03 |
| PLAAT4 | 1.81 | 9.03E-05 |
| ELMO3 | 1.81 | 7.90E-08 |
| TMPRSS3 | 1.81 | 7.09E-04 |
| KLRG2 | 1.80 | 1.41E-04 |
| LOC100505938 | 1.80 | 8.47E-06 |
| CHML | 1.80 | 3.62E-08 |
| ERMP1 | 1.80 | 3.43E-13 |
| PGGHG | 1.80 | 5.98E-04 |
| PDIA4 | 1.80 | 2.97E-12 |
| FUS | 1.79 | 2.58E-14 |
| VPS37C | 1.79 | 1.03E-13 |
| ATP6AP1 | 1.79 | 2.69E-08 |
| RDH13 | 1.79 | 6.13E-14 |
| DNMT1 | 1.78 | 6.95E-17 |
| KIF11 | 1.78 | 1.13E-06 |
| TAPBP | 1.78 | 3.54E-12 |
| CELSR2 | 1.78 | 4.18E-06 |
| KPNA2 | 1.78 | 1.65E-13 |
| IRF7 | 1.77 | 5.94E-08 |
| TPM4 | 1.77 | 2.03E-08 |
| KDM5B | 1.77 | 2.97E-10 |
| RHBDF1 | 1.77 | 5.72E-08 |
| BCOR | 1.77 | 2.96E-07 |
| WFDC2 | 1.77 | 4.80E-03 |
| PARP12 | 1.77 | 2.45E-08 |
| SERTAD4 | 1.76 | 3.28E-05 |
| SEMA4B | 1.76 | 4.50E-10 |
| RASSF6 | 1.76 | 2.92E-04 |
| PYCARD | 1.76 | 9.60E-07 |
| TESMIN | 1.76 | 2.07E-06 |
| ZNF587 | 1.76 | 3.94E-11 |
| FAM110A | 1.76 | 7.31E-10 |
| VCAN | 1.76 | 9.65E-06 |
| VTI1A | 1.76 | 4.89E-08 |
| ATAD2 | 1.76 | 1.31E-07 |
| DCAF8 | 1.75 | 4.16E-12 |
| C6orf132 | 1.75 | 3.91E-10 |
| SLC44A1 | 1.75 | 1.19E-10 |
| RECQL4 | 1.75 | 1.00E-07 |
| SLC2A10 | 1.75 | 7.29E-04 |
| ISYNA1 | 1.75 | 8.22E-06 |
| PGM2L1 | 1.75 | 6.42E-08 |
| CXCR4 | 1.75 | 2.47E-06 |
| ZNF703 | 1.75 | 2.90E-04 |
| ZNF618 | 1.74 | 1.60E-08 |
| PLPP5 | 1.74 | 3.16E-06 |
| SAMD12 | 1.74 | 1.34E-05 |
| PPP1R10 | 1.74 | 3.50E-08 |
| OVOL1 | 1.73 | 6.09E-09 |
| PTGFRN | 1.73 | 2.63E-09 |
| EIF3C | 1.73 | 1.26E-08 |
| BEX2 | 1.73 | 4.24E-04 |
| BHLHE40 | 1.73 | 2.37E-08 |
| FANCF | 1.73 | 1.21E-08 |
| NIBAN2 | 1.73 | 2.18E-10 |
| CLSTN1 | 1.73 | 3.14E-13 |
| NECTIN2 | 1.73 | 3.53E-09 |
| SORL1 | 1.73 | 3.43E-05 |
| NCAPG | 1.72 | 1.94E-06 |
| ENO2 | 1.72 | 1.31E-04 |
| ZNF493 | 1.72 | 1.05E-07 |
| GRP | 1.72 | 2.35E-04 |
| AZGP1 | 1.72 | 3.17E-02 |
| CHST15 | 1.72 | 1.33E-07 |
| SUSD2 | 1.72 | 2.72E-04 |
| ABCC5 | 1.71 | 1.18E-05 |
| IQCA1 | 1.71 | 7.75E-04 |
| PERP | 1.71 | 2.84E-04 |
| MAD2L1 | 1.71 | 6.83E-06 |
| DUXAP10 | 1.71 | 3.07E-04 |
| GPR160 | 1.71 | 1.94E-03 |
| CBX2 | 1.71 | 9.43E-05 |
| CMTM4 | 1.70 | 1.56E-08 |
| HIP1R | 1.70 | 1.41E-09 |
| NDC80 | 1.70 | 5.51E-06 |
| CTPS1 | 1.70 | 9.40E-07 |
| UNC5B | 1.70 | 8.91E-08 |
| GATA3-AS1 | 1.69 | 1.69E-03 |
| CLDN7 | 1.69 | 2.17E-07 |
| PGAP3 | 1.68 | 9.90E-04 |
| STX3 | 1.68 | 8.87E-09 |
| SHTN1 | 1.68 | 1.61E-08 |
| UGCG | 1.68 | 8.94E-07 |
| RNF43 | 1.68 | 1.00E-05 |
| RAP1GAP | 1.68 | 3.62E-06 |
| IGSF9 | 1.68 | 5.64E-08 |
| HMGN1 | 1.68 | 7.48E-14 |
| TEFM | 1.68 | 3.34E-07 |
| DEF6 | 1.67 | 3.24E-08 |
| PREX1 | 1.67 | 5.23E-04 |
| ENC1 | 1.67 | 8.61E-07 |
| SLC11A2 | 1.67 | 3.81E-12 |
| PSRC1 | 1.67 | 7.04E-09 |
| LMNB2 | 1.67 | 1.64E-07 |
| CCT3 | 1.66 | 9.00E-17 |
| CEP41 | 1.66 | 1.22E-08 |
| SLC1A4 | 1.66 | 3.12E-04 |
| N4BP2L2 | 1.66 | 4.86E-12 |
| LYPD6B | 1.66 | 1.05E-04 |
| PSD4 | 1.66 | 8.12E-14 |
| SERPINA1 | 1.66 | 1.44E-02 |
| ABCG1 | 1.66 | 5.38E-06 |
| GPR27 | 1.65 | 4.42E-04 |
| SPHK1 | 1.65 | 5.01E-06 |
| FGD3 | 1.65 | 2.18E-03 |
| AJUBA | 1.65 | 5.72E-06 |
| GOT2 | 1.65 | 3.78E-09 |
| EVL | 1.65 | 2.04E-04 |
| PTP4A3 | 1.65 | 6.89E-06 |
| SRRM2 | 1.64 | 6.49E-07 |
| LAMP3 | 1.64 | 4.82E-03 |
| FAM102A | 1.64 | 4.05E-11 |
| PBK | 1.64 | 1.72E-05 |
| ZMYND8 | 1.64 | 2.05E-07 |
| H2BC12 | 1.63 | 4.10E-04 |
| DNAJC1 | 1.63 | 6.39E-07 |
| ABCC3 | 1.63 | 1.06E-03 |
| RCAN3 | 1.63 | 1.35E-07 |
| SLC39A4 | 1.63 | 2.24E-04 |
| RETREG2 | 1.63 | 3.80E-18 |
| MPZL2 | 1.63 | 3.04E-05 |
| TMEM9 | 1.62 | 3.83E-10 |
| ARFGAP1 | 1.62 | 2.17E-11 |
| PPP1R14B | 1.62 | 1.36E-10 |
| CAPS | 1.62 | 1.45E-04 |
| PPFIBP1 | 1.62 | 2.03E-04 |
| MYO19 | 1.62 | 9.54E-09 |
| LIG1 | 1.62 | 4.78E-13 |
| S100A2 | 1.61 | 1.76E-02 |
| NUP210 | 1.61 | 3.80E-08 |
| PAICS | 1.61 | 2.05E-12 |
| H2AW | 1.61 | 6.51E-04 |
| SBK1 | 1.61 | 2.58E-06 |
| MTFR2 | 1.61 | 7.16E-06 |
| KIFC2 | 1.61 | 1.78E-05 |
| TRPM4 | 1.61 | 1.45E-07 |
| RACGAP1 | 1.61 | 1.99E-09 |
| VMP1 | 1.61 | 1.34E-05 |
| TLCD1 | 1.61 | 1.08E-04 |
| PTPRK | 1.60 | 5.60E-06 |
| MFSD10 | 1.60 | 1.31E-08 |
| DSG2 | 1.60 | 2.62E-05 |
| AP1S2 | 1.60 | 3.96E-07 |
| SUSD6 | 1.59 | 7.61E-13 |
| CYP4Z2P | 1.59 | 1.45E-02 |
| MEAK7 | 1.59 | 7.18E-08 |
| LRRN1 | 1.59 | 8.51E-04 |
| KIAA0895 | 1.59 | 2.26E-07 |
| GUSBP14 | 1.59 | 9.81E-05 |
| P3H4 | 1.59 | 9.17E-06 |
| ZNF107 | 1.59 | 1.88E-06 |
| DLG5 | 1.59 | 3.36E-06 |
| HOTAIR | 1.59 | 3.98E-04 |
| ZNF587B | 1.58 | 6.93E-10 |
| FAM185A | 1.58 | 3.08E-07 |
| TNFAIP8L1 | 1.58 | 7.33E-09 |
| SLC39A11 | 1.58 | 6.02E-06 |
| RAB40C | 1.58 | 8.25E-09 |
| ADAR | 1.58 | 7.00E-19 |
| COL5A1 | 1.58 | 3.97E-04 |
| LINC01004 | 1.58 | 1.24E-08 |
| TRAPPC10 | 1.58 | 1.16E-10 |
| C9orf152 | 1.58 | 2.10E-04 |
| KDM2A | 1.58 | 2.07E-11 |
| MAL2 | 1.58 | 6.54E-04 |
| DEPDC1 | 1.57 | 2.01E-06 |
| CDC7 | 1.57 | 8.90E-06 |
| CHD6 | 1.57 | 7.27E-09 |
| ITGA2 | 1.57 | 8.67E-04 |
| WDR6 | 1.57 | 3.99E-11 |
| TRIM14 | 1.57 | 7.92E-08 |
| TMEM63A | 1.57 | 5.69E-08 |
| CDT1 | 1.57 | 7.12E-06 |
| MYEF2 | 1.57 | 1.05E-05 |
| ZWILCH | 1.57 | 4.68E-09 |
| SLC20A1 | 1.57 | 3.39E-11 |
| CCDC14 | 1.57 | 2.40E-07 |
| GCH1 | 1.57 | 5.07E-05 |
| ZNF764 | 1.56 | 1.34E-10 |
| CSTF3 | 1.56 | 1.41E-06 |
| ARSD | 1.56 | 2.22E-05 |
| MICB | 1.56 | 6.27E-04 |
| KLHDC7B | 1.56 | 8.83E-03 |
| BICDL1 | 1.56 | 1.57E-07 |
| GABPB1-AS1 | 1.56 | 1.36E-04 |
| H2AX | 1.56 | 3.09E-08 |
| SEPTIN9 | 1.56 | 4.66E-16 |
| TUFT1 | 1.56 | 4.45E-06 |
| EXOC7 | 1.55 | 2.73E-09 |
| VAMP8 | 1.55 | 8.53E-09 |
| DOK7 | 1.55 | 1.19E-02 |
| SMC4 | 1.55 | 9.26E-10 |
| ZNF92 | 1.55 | 1.12E-05 |
| PLEKHS1 | 1.55 | 1.25E-03 |
| RASSF10 | 1.55 | 9.66E-03 |
| HERC6 | 1.55 | 2.99E-03 |
| ALDH18A1 | 1.55 | 1.07E-15 |
| SRARP | 1.55 | 4.80E-03 |
| ASNS | 1.55 | 7.07E-06 |
| ITGB8 | 1.54 | 2.09E-03 |
| POLR2J4 | 1.54 | 4.64E-11 |
| RASGRP1 | 1.54 | 1.86E-03 |
| PLK2 | 1.54 | 9.52E-05 |
| TSPAN1 | 1.54 | 3.71E-03 |
| JPH1 | 1.54 | 8.52E-04 |
| LUC7L | 1.54 | 8.51E-09 |
| PUS7 | 1.54 | 4.81E-10 |
| LMO7 | 1.54 | 1.46E-05 |
| RAI1 | 1.54 | 3.92E-11 |
| FAAH2 | 1.53 | 6.58E-10 |
| HCP5 | 1.53 | 7.20E-04 |
| F11R | 1.53 | 4.25E-13 |
| FOXM1 | 1.53 | 1.63E-05 |
| CCDC18-AS1 | 1.53 | 4.43E-08 |
| RMI2 | 1.53 | 8.04E-05 |
| TNFRSF12A | 1.53 | 2.72E-05 |
| FAM83B | 1.53 | 2.74E-04 |
| SLC4A11 | 1.52 | 9.05E-03 |
| CST6 | 1.52 | 1.24E-03 |
| POU2F3 | 1.52 | 1.69E-04 |
| GRTP1 | 1.52 | 2.39E-05 |
| RUNX2 | 1.52 | 1.17E-05 |
| TOP1MT | 1.52 | 1.69E-07 |
| SCRIB | 1.52 | 1.98E-09 |
| SEPTIN8 | 1.52 | 2.97E-13 |
| CERS2 | 1.52 | 7.49E-07 |
| TAGLN2 | 1.52 | 1.14E-08 |
| SUCO | 1.52 | 7.48E-12 |
| LINC02591 | 1.52 | 3.42E-05 |
| PIK3R2 | 1.52 | 3.71E-11 |
| RGS4 | 1.52 | 2.36E-03 |
| BST2 | 1.52 | 4.37E-04 |
| RNF183 | 1.51 | 6.46E-03 |
| MFAP2 | 1.51 | 3.72E-04 |
| SYT13 | 1.51 | 3.34E-02 |
| SLC1A2 | 1.51 | 5.23E-03 |
| DDX39A | 1.51 | 2.74E-09 |
| LOC102724851 | 1.51 | 3.70E-05 |
| STC1 | 1.51 | 6.77E-03 |
| ZDHHC13 | 1.51 | 1.35E-08 |
| CDKN3 | 1.50 | 1.49E-04 |
| PLEKHB1 | 1.50 | 1.48E-03 |
| NRIP3 | 1.50 | 1.43E-02 |
| AMMECR1 | 1.50 | 6.97E-07 |
| SUSD4 | 1.50 | 3.16E-04 |
| NME3 | 1.50 | 1.12E-06 |
| TAP1 | 1.49 | 2.74E-04 |
| CENPK | 1.49 | 3.77E-07 |
| CXCL11 | 1.49 | 5.17E-03 |
| REPS2 | 1.49 | 2.18E-02 |
| CGNL1 | 1.49 | 3.14E-04 |
| SLC30A8 | 1.49 | 4.85E-02 |
| CEACAM1 | 1.49 | 4.80E-04 |
| WDR4 | 1.49 | 1.70E-06 |
| B3GALNT1 | 1.48 | 1.74E-06 |
| RSKR | 1.48 | 1.96E-08 |
| MAP3K9 | 1.48 | 2.76E-10 |
| SPAG5 | 1.48 | 4.66E-07 |
| GCAT | 1.48 | 2.52E-07 |
| TWNK | 1.48 | 1.06E-07 |
| ANKRD30B | 1.47 | 1.03E-02 |
| RBM47 | 1.47 | 1.00E-06 |
| ISG20L2 | 1.47 | 8.27E-09 |
| CTBP2 | 1.47 | 5.27E-10 |
| PATZ1 | 1.47 | 9.38E-11 |
| NR2F6 | 1.47 | 8.60E-07 |
| CAPG | 1.47 | 1.49E-04 |
| TC2N | 1.46 | 2.45E-04 |
| PLS1 | 1.46 | 2.10E-04 |
| SPATA17 | 1.46 | 8.51E-04 |
| GGCT | 1.46 | 5.87E-08 |
| GTF3C1 | 1.46 | 1.34E-10 |
| ILF3 | 1.46 | 1.76E-14 |
| RGL2 | 1.46 | 1.34E-09 |
| SPIN4 | 1.46 | 4.40E-08 |
| MANEAL | 1.46 | 6.02E-04 |
| RAB17 | 1.45 | 3.60E-06 |
| BMS1P2 | 1.45 | 2.19E-08 |
| KIAA1671 | 1.45 | 2.66E-08 |
| MAGED1 | 1.45 | 4.28E-06 |
| FZD2 | 1.45 | 7.01E-06 |
| TMEM87B | 1.45 | 1.66E-08 |
| SNRNP70 | 1.45 | 4.69E-07 |
| SLC44A4 | 1.45 | 5.33E-04 |
| PRPF38B | 1.45 | 4.58E-09 |
| POLD1 | 1.45 | 2.74E-09 |
| HMGA1 | 1.45 | 5.94E-06 |
| SPAG1 | 1.45 | 1.18E-05 |
| SFPQ | 1.45 | 3.29E-06 |
| USP54 | 1.45 | 7.88E-08 |
| LMLN | 1.44 | 3.14E-08 |
| TANC2 | 1.44 | 2.09E-04 |
| PARP9 | 1.44 | 9.95E-06 |
| SOGA1 | 1.44 | 2.26E-09 |
| MLLT11 | 1.44 | 4.96E-06 |
| FBXO41 | 1.44 | 2.95E-07 |
| PIAS3 | 1.44 | 2.30E-07 |
| LPCAT1 | 1.44 | 1.44E-06 |
| RALGPS2 | 1.44 | 5.82E-05 |
| GCNT2 | 1.44 | 6.33E-03 |
| LMNB1 | 1.44 | 5.42E-08 |
| TRIM11 | 1.43 | 2.96E-10 |
| TCF3 | 1.43 | 9.19E-07 |
| MID1 | 1.43 | 5.44E-09 |
| TUBA4A | 1.43 | 7.83E-06 |
| PLEKHA6 | 1.43 | 2.68E-04 |
| DNAJC7 | 1.43 | 6.68E-12 |
| ABRACL | 1.43 | 7.04E-05 |
| H2BC6 | 1.43 | 7.89E-05 |
| H3C6 | 1.43 | 7.00E-05 |
| CASP2 | 1.43 | 2.99E-11 |
| CEP164 | 1.43 | 3.82E-07 |
| MELTF | 1.43 | 2.35E-03 |
| INPP4B | 1.43 | 1.32E-03 |
| EPHB3 | 1.42 | 4.28E-06 |
| SPATS2 | 1.42 | 2.46E-09 |
| SHCBP1 | 1.41 | 7.67E-05 |
| ZKSCAN1 | 1.41 | 5.35E-08 |
| CCNA2 | 1.41 | 6.39E-07 |
| XPO7 | 1.41 | 6.58E-07 |
| MOV10 | 1.41 | 5.33E-06 |
| CCDC85C | 1.41 | 7.09E-13 |
| KIF22 | 1.41 | 9.80E-07 |
| DKK3 | 1.41 | 2.26E-04 |
| CTPS2 | 1.41 | 9.21E-06 |
| IPO9 | 1.41 | 8.43E-12 |
| HMG20B | 1.41 | 3.81E-09 |
| BAIAP2-DT | 1.40 | 2.09E-06 |
| TRAF5 | 1.40 | 8.00E-05 |
| KIAA1211L | 1.40 | 7.41E-04 |
| MTCL1 | 1.40 | 3.97E-04 |
| SRRT | 1.40 | 3.16E-12 |
| SEC61A2 | 1.40 | 1.99E-06 |
| GALNT10 | 1.40 | 1.47E-04 |
| GREB1 | 1.40 | 3.73E-02 |
| SUSD3 | 1.40 | 4.51E-03 |
| UBQLN4 | 1.40 | 6.33E-09 |
| IPO4 | 1.40 | 1.91E-08 |
| TENT5C | 1.40 | 8.38E-03 |
| TAF1D | 1.40 | 8.48E-08 |
| DPP3 | 1.40 | 5.85E-10 |
| NPDC1 | 1.40 | 2.82E-05 |
| TRIM25 | 1.40 | 5.87E-11 |
| PTBP3 | 1.39 | 4.48E-10 |
| PLEKHF2 | 1.39 | 2.91E-04 |
| TPM3 | 1.39 | 7.48E-14 |
| CCNL2 | 1.39 | 3.18E-08 |
| MMP3 | 1.39 | 4.30E-03 |
| MREG | 1.39 | 1.38E-05 |
| TIMP1 | 1.39 | 9.48E-05 |
| PEX11B | 1.39 | 4.19E-08 |
| CSK | 1.39 | 6.98E-08 |
| LOC101926959 | 1.39 | 9.57E-03 |
| TIGD1 | 1.39 | 2.46E-06 |
| BCL11A | 1.39 | 3.64E-02 |
| APP | 1.39 | 4.80E-06 |
| DLEU2 | 1.38 | 4.72E-05 |
| NETO2 | 1.38 | 5.58E-04 |
| IKBKB | 1.38 | 8.85E-06 |
| MORC2 | 1.38 | 1.39E-10 |
| STARD3 | 1.38 | 4.62E-04 |
| SH3RF1 | 1.38 | 2.58E-06 |
| FAM111A-DT | 1.38 | 3.77E-09 |
| H2BC4 | 1.37 | 1.39E-02 |
| NSUN6 | 1.37 | 3.80E-05 |
| CRIP2 | 1.37 | 1.78E-03 |
| GLI3 | 1.37 | 3.69E-04 |
| FEN1 | 1.37 | 2.98E-07 |
| TRIM28 | 1.37 | 3.73E-09 |
| RBM12B | 1.37 | 2.51E-10 |
| MAZ | 1.37 | 9.27E-09 |
| RAE1 | 1.37 | 6.42E-14 |
| BAIAP2 | 1.37 | 1.20E-06 |
| MBOAT7 | 1.37 | 1.32E-09 |
| EP300-AS1 | 1.37 | 8.58E-04 |
| AEBP1 | 1.37 | 4.16E-04 |
| SLC4A8 | 1.37 | 9.81E-05 |
| KIF23 | 1.37 | 2.14E-05 |
| ENAH | 1.37 | 2.93E-07 |
| CCNF | 1.37 | 7.62E-10 |
| NRARP | 1.37 | 6.33E-06 |
| MYBL2 | 1.37 | 1.28E-04 |
| E2F7 | 1.36 | 8.95E-06 |
| ERN1 | 1.36 | 5.41E-07 |
| BZW2 | 1.36 | 3.17E-11 |
| COL13A1 | 1.36 | 1.80E-04 |
| HDGF | 1.36 | 5.77E-10 |
| ADAM8 | 1.36 | 2.03E-05 |
| ELAVL2 | 1.36 | 9.84E-03 |
| NAA25 | 1.35 | 1.90E-07 |
| ZSCAN16 | 1.35 | 4.48E-10 |
| MST1R | 1.35 | 5.58E-06 |
| AHCY | 1.35 | 2.73E-07 |
| DNMT3A | 1.35 | 5.45E-10 |
| ISG20 | 1.35 | 2.15E-03 |
| NDUFAF6 | 1.35 | 2.21E-05 |
| PTTG1IP | 1.35 | 3.26E-10 |
| HSPA2 | 1.35 | 1.02E-02 |
| SYNGR2 | 1.35 | 5.27E-05 |
| SNHG17 | 1.35 | 2.05E-07 |
| DACH1 | 1.35 | 3.95E-02 |
| CAPN13 | 1.35 | 7.41E-04 |
| FOXK2 | 1.35 | 1.04E-08 |
| ANKRD36 | 1.35 | 4.62E-04 |
| VANGL2 | 1.35 | 3.31E-04 |
| CYFIP2 | 1.35 | 3.19E-03 |
| RAB3IP | 1.35 | 1.08E-07 |
| NREP | 1.35 | 1.17E-05 |
| AKAP8L | 1.34 | 1.46E-05 |
| TUBBP5 | 1.34 | 1.41E-05 |
| LINC00665 | 1.34 | 4.93E-07 |
| ITGB4 | 1.34 | 3.43E-04 |
| EME1 | 1.34 | 1.51E-04 |
| ST6GALNAC5 | 1.34 | 7.34E-03 |
| PHF12 | 1.34 | 5.25E-07 |
| ATP13A1 | 1.34 | 2.12E-07 |
| IL17RB | 1.34 | 3.15E-03 |
| UVSSA | 1.34 | 4.88E-07 |
| COL5A2 | 1.34 | 1.88E-03 |
| ROGDI | 1.33 | 5.49E-08 |
| GTF3C4 | 1.33 | 8.26E-07 |
| ZNF3 | 1.33 | 1.12E-08 |
| RUVBL2 | 1.33 | 4.52E-08 |
| RAP1GAP2 | 1.33 | 2.07E-06 |
| CFAP44 | 1.33 | 1.29E-06 |
| C5orf46 | 1.33 | 2.03E-04 |
| GLYATL2 | 1.33 | 2.81E-02 |
| CBX5 | 1.33 | 2.50E-06 |
| FAM111A | 1.33 | 6.75E-06 |
| ARF3 | 1.33 | 2.32E-07 |
| CENPM | 1.33 | 1.34E-06 |
| DHCR7 | 1.33 | 2.04E-04 |
| FKBP11 | 1.33 | 6.55E-06 |
| TTLL12 | 1.33 | 2.96E-05 |
| MFSD6 | 1.33 | 3.99E-04 |
| TLE3 | 1.33 | 5.21E-06 |
| B4GALT3 | 1.33 | 2.03E-07 |
| RBM8A | 1.32 | 5.84E-10 |
| TRIM29 | 1.32 | 4.04E-02 |
| GPAT4 | 1.32 | 1.18E-04 |
| PRPF19 | 1.32 | 7.28E-11 |
| GON4L | 1.32 | 7.53E-06 |
| RNF213 | 1.32 | 2.80E-06 |
| CHPF2 | 1.32 | 1.42E-10 |
| PDK3 | 1.32 | 1.47E-04 |
| DHCR24 | 1.32 | 1.45E-03 |
| MACROH2A1 | 1.32 | 1.72E-13 |
| KIF5C | 1.32 | 1.79E-02 |
| PHLDB3 | 1.31 | 2.61E-07 |
| CAPN1 | 1.31 | 3.44E-09 |
| MIDN | 1.31 | 6.87E-05 |
| SLAMF8 | 1.31 | 1.43E-02 |
| GGT6 | 1.31 | 2.24E-04 |
| NSUN5P1 | 1.31 | 2.72E-08 |
| KISS1R | 1.31 | 1.24E-02 |
| EMP2 | 1.31 | 7.36E-06 |
| IFT122 | 1.31 | 2.75E-03 |
| STRBP | 1.31 | 1.64E-06 |
| H2AC6 | 1.31 | 1.22E-02 |
| TMEM158 | 1.31 | 2.63E-03 |
| SMYD3 | 1.31 | 4.41E-06 |
| POLR1C | 1.31 | 6.86E-12 |
| MTA1 | 1.31 | 4.63E-08 |
| DNAH5 | 1.31 | 2.21E-03 |
| NPIPA1 | 1.31 | 6.39E-06 |
| TASOR2 | 1.31 | 2.39E-05 |
| LYPD3 | 1.31 | 8.21E-04 |
| RBM6 | 1.31 | 1.37E-04 |
| MUC20 | 1.31 | 1.80E-03 |
| ENTPD6 | 1.30 | 9.43E-10 |
| PLXNA1 | 1.30 | 6.86E-05 |
| KMT2D | 1.30 | 9.30E-08 |
| NME1 | 1.30 | 1.65E-04 |
| RNF144B | 1.30 | 2.13E-05 |
| POLR2H | 1.30 | 1.31E-14 |
| BLNK | 1.30 | 1.42E-03 |
| PLXNB2 | 1.30 | 2.72E-08 |
| MED16 | 1.30 | 3.86E-06 |
| RASEF | 1.30 | 3.29E-03 |
| HYOU1 | 1.30 | 2.72E-08 |
| H4C8 | 1.30 | 1.00E-02 |
| F2RL1 | 1.30 | 1.11E-03 |
| MMEL1 | 1.30 | 3.36E-05 |
| CADM4 | 1.30 | 2.50E-04 |
| GPSM2 | 1.30 | 6.86E-05 |
| TINCR | 1.30 | 3.24E-03 |
| SETD5 | 1.30 | 3.69E-09 |
| RALGPS1 | 1.30 | 1.28E-06 |
| ERG28 | 1.29 | 2.12E-08 |
| POLB | 1.29 | 1.32E-06 |
| BRD2 | 1.29 | 5.22E-08 |
| GRAMD1A | 1.29 | 1.04E-06 |
| ZNF785 | 1.29 | 4.56E-04 |
| SRPK1 | 1.29 | 5.76E-10 |
| TACC3 | 1.29 | 6.36E-06 |
| PIGM | 1.29 | 5.85E-06 |
| FANCL | 1.29 | 1.19E-08 |
| TFPT | 1.29 | 4.04E-05 |
| GPATCH2L | 1.29 | 3.75E-08 |
| KCTD5 | 1.29 | 2.86E-05 |
| UBE2S | 1.29 | 4.29E-04 |
| VPS37B | 1.29 | 8.40E-08 |
| TMF1 | 1.29 | 5.45E-07 |
| SEPTIN7P2 | 1.29 | 9.89E-06 |
| KCNS3 | 1.29 | 3.33E-05 |
| CTSD | 1.29 | 1.66E-03 |
| PAGR1 | 1.28 | 2.83E-07 |
| FAAH | 1.28 | 3.91E-05 |
| PFKFB4 | 1.28 | 2.81E-08 |
| LUC7L3 | 1.28 | 1.69E-04 |
| PDRG1 | 1.28 | 3.56E-08 |
| LRATD1 | 1.28 | 4.80E-04 |
| CERS4 | 1.28 | 1.33E-03 |
| DGKD | 1.28 | 7.60E-06 |
| C1orf56 | 1.28 | 6.80E-05 |
| MBD6 | 1.28 | 4.09E-06 |
| ZNF776 | 1.28 | 3.36E-09 |
| HES4 | 1.27 | 1.25E-06 |
| IDH2 | 1.27 | 6.60E-05 |
| NIPSNAP1 | 1.27 | 2.47E-10 |
| SNRPB | 1.27 | 4.39E-07 |
| NAIP | 1.27 | 2.41E-03 |
| TBCD | 1.27 | 1.53E-09 |
| FZD3 | 1.27 | 7.84E-04 |
| RASGEF1A | 1.27 | 8.29E-03 |
| FRK | 1.27 | 5.28E-04 |
| CHD7 | 1.27 | 2.67E-04 |
| PDIA3 | 1.26 | 1.37E-09 |
| H2BS1 | 1.26 | 5.70E-03 |
| RAI14 | 1.26 | 6.46E-08 |
| CRNDE | 1.26 | 1.23E-04 |
| ANTKMT | 1.26 | 1.86E-07 |
| TSC22D4 | 1.26 | 4.61E-07 |
| ZNF217 | 1.26 | 7.05E-09 |
| SUGP2 | 1.26 | 3.47E-12 |
| SEC16A | 1.26 | 5.84E-09 |
| SPICE1 | 1.26 | 1.05E-05 |
| CTTN | 1.25 | 5.33E-07 |
| PLP2 | 1.25 | 1.09E-04 |
| SLC7A11 | 1.25 | 4.30E-04 |
| VARS1 | 1.25 | 1.83E-06 |
| PLPP4 | 1.25 | 1.07E-03 |
| TDG | 1.25 | 4.95E-11 |
| ORAI2 | 1.25 | 3.15E-05 |
| ABHD11 | 1.25 | 1.50E-05 |
| CLK2 | 1.25 | 7.96E-11 |
| B3GALT5 | 1.25 | 8.38E-03 |
| NMRAL1 | 1.25 | 2.02E-10 |
| PPP1CA | 1.24 | 7.57E-05 |
| TNFRSF10A | 1.24 | 1.48E-05 |
| CAMK2N1 | 1.24 | 1.40E-02 |
| TELO2 | 1.24 | 1.02E-08 |
| COL1A1 | 1.24 | 1.04E-03 |
| MKI67 | 1.24 | 2.34E-03 |
| PLEK2 | 1.24 | 1.31E-04 |
| CNNM4 | 1.24 | 9.71E-07 |
| EBLN2 | 1.24 | 4.64E-07 |
| KRT80 | 1.24 | 3.64E-05 |
| KIAA0319L | 1.24 | 2.69E-08 |
| APH1A | 1.24 | 5.91E-07 |
| SLC12A7 | 1.24 | 1.95E-10 |
| BCAS1 | 1.24 | 8.40E-04 |
| SRCIN1 | 1.24 | 1.76E-02 |
| HDAC1 | 1.24 | 1.31E-07 |
| CENPX | 1.24 | 2.38E-07 |
| PAK4 | 1.24 | 9.62E-08 |
| TMPO | 1.24 | 1.95E-06 |
| RNF207 | 1.24 | 7.01E-06 |
| ETV6 | 1.24 | 2.90E-07 |
| ZNF239 | 1.24 | 4.68E-05 |
| ADNP | 1.24 | 7.71E-07 |
| CNKSR1 | 1.23 | 9.29E-08 |
| SHMT2 | 1.23 | 9.76E-07 |
| PDE7A | 1.23 | 1.01E-04 |
| SLC2A1 | 1.23 | 7.71E-04 |
| NAT10 | 1.23 | 5.50E-08 |
| LINC00173 | 1.23 | 7.15E-03 |
| NUMA1 | 1.23 | 2.34E-07 |
| GDAP1 | 1.23 | 3.59E-02 |
| DIO1 | 1.23 | 4.47E-02 |
| TM4SF1 | 1.23 | 1.44E-02 |
| MYH9 | 1.23 | 3.82E-09 |
| PTPA | 1.23 | 4.96E-09 |
| DDAH1 | 1.23 | 2.96E-05 |
| D2HGDH | 1.23 | 1.33E-04 |
| DGCR2 | 1.23 | 1.53E-10 |
| DMKN | 1.23 | 1.02E-03 |
| CERS6 | 1.23 | 2.75E-04 |
| RNASEH2A | 1.23 | 2.72E-05 |
| PNMA8A | 1.23 | 2.09E-02 |
| DHX33 | 1.23 | 1.08E-07 |
| MIR181A2HG | 1.22 | 3.43E-04 |
| HS6ST1 | 1.22 | 3.77E-09 |
| GDF15 | 1.22 | 9.12E-03 |
| MEGF10 | 1.22 | 1.26E-02 |
| DDX31 | 1.22 | 2.68E-07 |
| MLLT6 | 1.22 | 7.28E-04 |
| ADAM1A | 1.22 | 1.26E-05 |
| GOLGA2P10 | 1.22 | 1.29E-05 |
| CEACAM5 | 1.22 | 2.12E-02 |
| AP3D1 | 1.22 | 1.59E-05 |
| DTX3L | 1.22 | 4.96E-10 |
| PTPRF | 1.22 | 1.47E-03 |
| SMARCC1 | 1.22 | 1.65E-11 |
| PRRC2C | 1.22 | 8.67E-14 |
| NSD2 | 1.22 | 6.46E-10 |
| CDCA4 | 1.22 | 5.39E-06 |
| CNTNAP2 | 1.22 | 1.25E-02 |
| MGAT4B | 1.22 | 6.31E-07 |
| FAM234B | 1.21 | 3.32E-02 |
| DCTPP1 | 1.21 | 3.16E-08 |
| NEK5 | 1.21 | 2.85E-02 |
| RFX5 | 1.21 | 1.96E-06 |
| S100A16 | 1.21 | 5.33E-04 |
| FBN2 | 1.21 | 6.65E-03 |
| RPS6KB2 | 1.21 | 1.51E-07 |
| HPRT1 | 1.21 | 1.19E-05 |
| SPATA13 | 1.21 | 6.29E-06 |
| E2F3 | 1.21 | 5.36E-06 |
| MDM4 | 1.21 | 9.56E-09 |
| CORO2A | 1.21 | 2.05E-05 |
| MAPKAPK2 | 1.21 | 1.04E-08 |
| CHTOP | 1.21 | 5.74E-10 |
| PGAP2 | 1.20 | 7.05E-06 |
| LINC01000 | 1.20 | 3.46E-04 |
| PACSIN2 | 1.20 | 1.33E-09 |
| TPD52L1 | 1.20 | 1.87E-02 |
| PARP14 | 1.20 | 8.82E-05 |
| PBX1 | 1.20 | 3.00E-04 |
| MMP13 | 1.20 | 4.82E-03 |
| DIAPH1 | 1.20 | 6.58E-10 |
| SLC37A1 | 1.20 | 4.71E-07 |
| FJX1 | 1.20 | 6.25E-04 |
| SLC26A11 | 1.20 | 3.57E-07 |
| ERBB4 | 1.20 | 1.01E-02 |
| LINC01138 | 1.20 | 3.41E-04 |
| IRF2BP2 | 1.20 | 1.82E-13 |
| PAWR | 1.20 | 4.28E-04 |
| SPAG4 | 1.20 | 2.20E-04 |
| MEN1 | 1.20 | 2.49E-05 |
| USP34 | 1.20 | 5.21E-05 |
| PAXBP1 | 1.20 | 1.88E-04 |
| TNK2 | 1.20 | 2.34E-05 |
| ZNF700 | 1.19 | 9.47E-07 |
| MICALL1 | 1.19 | 1.40E-03 |
| MED1 | 1.19 | 2.37E-03 |
| INPP5J | 1.19 | 1.05E-03 |
| PLAU | 1.19 | 1.14E-02 |
| KRAS | 1.19 | 8.86E-09 |
| GRAMD1C | 1.19 | 7.07E-05 |
| ZNF738 | 1.19 | 6.37E-04 |
| LAGE3 | 1.19 | 1.46E-04 |
| MEX3A | 1.19 | 4.35E-04 |
| PIP4K2C | 1.19 | 3.36E-11 |
| AGPAT5 | 1.19 | 3.72E-06 |
| AFG3L1P | 1.19 | 5.66E-06 |
| PPP4C | 1.19 | 1.58E-06 |
| CELSR3 | 1.19 | 2.33E-03 |
| DENND1B | 1.19 | 3.17E-04 |
| RPS6KA1 | 1.19 | 7.59E-05 |
| SLC26A6 | 1.19 | 1.05E-09 |
| ATAD2B | 1.19 | 9.66E-06 |
| PUS1 | 1.19 | 1.04E-07 |
| TRIM36 | 1.18 | 1.24E-02 |
| NADSYN1 | 1.18 | 1.28E-09 |
| NLN | 1.18 | 8.53E-04 |
| EXOSC4 | 1.18 | 6.84E-05 |
| LINC02076 | 1.18 | 9.17E-06 |
| ABCC10 | 1.18 | 8.20E-06 |
| TP53INP1 | 1.18 | 7.28E-04 |
| FBXO45 | 1.18 | 2.13E-08 |
| ZNF544 | 1.18 | 5.38E-10 |
| ZNF500 | 1.18 | 1.53E-07 |
| TMEM134 | 1.18 | 3.22E-08 |
| ANKRD10 | 1.18 | 5.30E-04 |
| DCBLD1 | 1.18 | 1.75E-04 |
| ATP6V0B | 1.18 | 7.53E-04 |
| ZNF629 | 1.18 | 1.75E-08 |
| LOXL1-AS1 | 1.18 | 2.93E-04 |
| DKFZP586I1420 | 1.17 | 4.36E-05 |
| TPRN | 1.17 | 6.02E-07 |
| NBPF4 | 1.17 | 2.20E-02 |
| CBS | 1.17 | 2.67E-03 |
| SLC6A6 | 1.17 | 7.36E-06 |
| DCAF13 | 1.17 | 5.27E-05 |
| ENSA | 1.17 | 7.48E-09 |
| P4HTM | 1.17 | 1.87E-03 |
| LCOR | 1.17 | 1.54E-07 |
| MIF-AS1 | 1.17 | 1.71E-07 |
| TRIM47 | 1.17 | 3.55E-03 |
| RAMP1 | 1.17 | 7.40E-03 |
| KIF3A | 1.17 | 1.22E-07 |
| TCEAL3 | 1.17 | 8.19E-04 |
| CARD10 | 1.17 | 8.48E-03 |
| STYK1 | 1.17 | 8.95E-03 |
| TCF20 | 1.16 | 1.92E-09 |
| MIAT | 1.16 | 6.43E-04 |
| NFKBIE | 1.16 | 2.43E-05 |
| DTX3 | 1.16 | 2.38E-07 |
| TGIF1 | 1.16 | 2.78E-06 |
| MICAL1 | 1.16 | 5.70E-04 |
| INTS7 | 1.16 | 1.65E-06 |
| CDK5RAP3 | 1.16 | 1.36E-07 |
| PMS2P5 | 1.16 | 2.83E-07 |
| DDX17 | 1.16 | 3.05E-10 |
| PPP2R2C | 1.16 | 5.11E-03 |
| SHROOM2 | 1.16 | 4.36E-06 |
| KNTC1 | 1.16 | 2.64E-05 |
| NUDT16L1 | 1.16 | 9.36E-09 |
| XPO5 | 1.16 | 1.26E-06 |
| HLA-C | 1.16 | 9.47E-06 |
| CLMN | 1.15 | 3.77E-04 |
| TMEM25 | 1.15 | 1.04E-03 |
| ZNF121 | 1.15 | 4.72E-09 |
| RASSF7 | 1.15 | 8.39E-06 |
| PIGU | 1.15 | 6.46E-05 |
| POGZ | 1.15 | 1.94E-08 |
| CD47 | 1.15 | 5.89E-07 |
| VAV2 | 1.15 | 1.18E-08 |
| EGLN3 | 1.15 | 4.48E-03 |
| NSUN7 | 1.15 | 2.42E-04 |
| PCNX2 | 1.15 | 4.10E-04 |
| UBAP2L | 1.15 | 2.33E-06 |
| ASCL2 | 1.15 | 8.82E-03 |
| ARFIP2 | 1.15 | 4.38E-05 |
| DNAJC9 | 1.14 | 1.02E-06 |
| DANCR | 1.14 | 6.78E-03 |
| BUB3 | 1.14 | 9.36E-10 |
| GMDS | 1.14 | 6.44E-04 |
| CMPK2 | 1.14 | 1.52E-02 |
| ZNF623 | 1.14 | 3.79E-07 |
| METRN | 1.14 | 8.16E-04 |
| ST6GALNAC2 | 1.14 | 5.36E-03 |
| SS18L1 | 1.14 | 8.13E-06 |
| SIDT1 | 1.14 | 2.27E-02 |
| HSD17B7 | 1.14 | 1.46E-05 |
| LINC01355 | 1.14 | 2.43E-05 |
| IRF9 | 1.14 | 4.94E-04 |
| FAM241B | 1.14 | 3.15E-06 |
| KMT2B | 1.14 | 2.14E-08 |
| TES | 1.14 | 1.12E-05 |
| NOD2 | 1.14 | 1.17E-04 |
| NPAS2 | 1.14 | 4.07E-04 |
| LRG1 | 1.14 | 2.87E-02 |
| CD2 | 1.14 | 3.69E-02 |
| FRMD8 | 1.14 | 1.08E-07 |
| CORO1B | 1.14 | 1.04E-03 |
| EIF2AK1 | 1.14 | 9.15E-08 |
| NPTN-IT1 | 1.14 | 9.70E-06 |
| PYCR3 | 1.14 | 2.30E-04 |
| SGPL1 | 1.14 | 8.38E-07 |
| CDH11 | 1.13 | 1.05E-02 |
| ERGIC1 | 1.13 | 2.93E-04 |
| ABI2 | 1.13 | 3.23E-06 |
| SOCS7 | 1.13 | 3.60E-05 |
| ATG16L1 | 1.13 | 4.97E-07 |
| TCIRG1 | 1.13 | 8.38E-04 |
| PCSK6 | 1.13 | 3.46E-03 |
| PAK6 | 1.13 | 3.61E-04 |
| GOLGA2P5 | 1.13 | 5.18E-05 |
| VWA1 | 1.13 | 1.51E-05 |
| LCLAT1 | 1.13 | 4.18E-08 |
| CSNK1D | 1.13 | 1.99E-11 |
| KCTD1 | 1.13 | 3.49E-04 |
| FUBP1 | 1.13 | 2.78E-06 |
| OBSL1 | 1.13 | 1.42E-03 |
| HK1 | 1.13 | 2.74E-12 |
| TOMM34 | 1.13 | 4.31E-08 |
| NECAB3 | 1.13 | 4.98E-03 |
| C12orf49 | 1.13 | 8.61E-07 |
| FNBP4 | 1.12 | 9.15E-04 |
| PPP1R35 | 1.12 | 1.37E-06 |
| STMN1 | 1.12 | 1.07E-05 |
| TMEM141 | 1.12 | 2.34E-06 |
| PYCR1 | 1.12 | 8.37E-06 |
| MDC1 | 1.12 | 7.88E-07 |
| PRRT3 | 1.12 | 9.90E-04 |
| SPNS1 | 1.12 | 5.32E-06 |
| CDCA8 | 1.12 | 1.24E-04 |
| NVL | 1.12 | 4.77E-04 |
| KIFC1 | 1.12 | 2.32E-05 |
| WEE1 | 1.12 | 2.96E-06 |
| NANS | 1.12 | 4.26E-08 |
| CCAR1 | 1.12 | 1.68E-05 |
| NLRC5 | 1.12 | 4.94E-03 |
| LINC01410 | 1.11 | 2.29E-02 |
| NAV2 | 1.11 | 7.32E-03 |
| CCDC88C | 1.11 | 2.02E-05 |
| CMYA5 | 1.11 | 1.15E-03 |
| ZNF736 | 1.11 | 7.35E-06 |
| ZNF467 | 1.11 | 1.23E-04 |
| SGSM2 | 1.11 | 3.86E-05 |
| THBS2 | 1.11 | 1.02E-02 |
| PTPRJ | 1.11 | 2.34E-04 |
| RNF187 | 1.11 | 2.26E-09 |
| TIA1 | 1.11 | 2.87E-05 |
| RCC1 | 1.11 | 1.96E-05 |
| VAMP1 | 1.11 | 2.44E-04 |
| EHMT2 | 1.11 | 3.21E-06 |
| MYO10 | 1.11 | 1.89E-03 |
| TSPOAP1 | 1.11 | 6.44E-05 |
| COMMD5 | 1.11 | 1.00E-03 |
| RHNO1 | 1.11 | 1.43E-05 |
| ZNF137P | 1.11 | 4.40E-05 |
| DGKH | 1.11 | 6.86E-05 |
| NEURL1B | 1.11 | 6.26E-04 |
| PRPF3 | 1.11 | 4.22E-06 |
| LAD1 | 1.11 | 9.55E-04 |
| AFDN | 1.11 | 3.87E-04 |
| CRACR2A | 1.10 | 1.57E-02 |
| NUP62 | 1.10 | 1.79E-09 |
| SIPA1L3 | 1.10 | 7.57E-08 |
| HLA-B | 1.10 | 1.02E-03 |
| SIPA1L2 | 1.10 | 6.51E-04 |
| TSTD1 | 1.10 | 9.95E-05 |
| IL4I1 | 1.10 | 1.90E-02 |
| DAGLB | 1.10 | 1.37E-07 |
| RAB11FIP4 | 1.10 | 2.66E-06 |
| DNMT3B | 1.10 | 2.99E-05 |
| RNPS1 | 1.10 | 4.64E-11 |
| ING5 | 1.10 | 4.38E-04 |
| CHKA | 1.10 | 1.29E-05 |
| FZD1 | 1.10 | 1.41E-03 |
| LMTK2 | 1.10 | 5.64E-07 |
| PRKD2 | 1.10 | 2.72E-08 |
| OAS3 | 1.10 | 4.15E-03 |
| C6orf141 | 1.10 | 1.73E-02 |
| MZF1 | 1.10 | 6.51E-04 |
| CHST11 | 1.10 | 1.45E-03 |
| FNBP1L | 1.10 | 1.05E-03 |
| VGLL4 | 1.10 | 5.33E-10 |
| ARF1 | 1.09 | 4.08E-09 |
| SSR2 | 1.09 | 3.25E-10 |
| USP21 | 1.09 | 1.89E-03 |
| PRKCI | 1.09 | 3.89E-07 |
| GDPD1 | 1.09 | 1.07E-02 |
| CEP85 | 1.09 | 5.13E-05 |
| NOP2 | 1.09 | 2.96E-05 |
| SCUBE3 | 1.09 | 2.16E-02 |
| LRBA | 1.09 | 1.54E-04 |
| NIPAL3 | 1.09 | 5.06E-05 |
| CHSY1 | 1.09 | 1.24E-08 |
| XYLT2 | 1.08 | 4.28E-06 |
| COL8A2 | 1.08 | 2.08E-03 |
| FOXP1-IT1 | 1.08 | 5.46E-05 |
| EIF2B5 | 1.08 | 1.22E-11 |
| HLA-A | 1.08 | 1.51E-04 |
| TGIF2 | 1.08 | 8.93E-06 |
| CNOT11 | 1.08 | 5.11E-09 |
| SEPHS2 | 1.08 | 1.07E-05 |
| GBAP1 | 1.08 | 1.60E-06 |
| PSMG3 | 1.08 | 2.38E-07 |
| TNRC18 | 1.08 | 1.13E-06 |
| MIR570HG | 1.08 | 1.80E-05 |
| RANGAP1 | 1.08 | 1.59E-08 |
| ZNF431 | 1.08 | 8.47E-05 |
| TMEM41B | 1.08 | 1.58E-05 |
| PRAME | 1.08 | 3.95E-02 |
| GAK | 1.08 | 5.97E-08 |
| EIF1AD | 1.08 | 1.32E-08 |
| ZNF518A | 1.08 | 1.52E-07 |
| PTOV1 | 1.08 | 2.07E-06 |
| ST6GAL2 | 1.08 | 4.73E-04 |
| FIGNL1 | 1.08 | 3.67E-05 |
| JARID2 | 1.07 | 1.20E-06 |
| ZNF496 | 1.07 | 9.11E-06 |
| RMI1 | 1.07 | 3.76E-07 |
| TRIT1 | 1.07 | 8.73E-08 |
| FRAS1 | 1.07 | 2.31E-02 |
| LRIG2 | 1.07 | 5.53E-06 |
| ZNF562 | 1.07 | 2.47E-11 |
| TSPAN17 | 1.07 | 3.24E-09 |
| RNF24 | 1.07 | 7.04E-05 |
| PTBP1 | 1.07 | 2.57E-18 |
| SETDB1 | 1.07 | 2.46E-06 |
| ELL3 | 1.07 | 1.96E-03 |
| RSAD2 | 1.07 | 3.92E-02 |
| MCM5 | 1.07 | 1.76E-03 |
| MAT2A | 1.07 | 1.09E-13 |
| PTDSS1 | 1.07 | 5.79E-04 |
| ZBTB41 | 1.07 | 4.51E-05 |
| EBPL | 1.07 | 1.90E-04 |
| SIRT7 | 1.07 | 5.50E-07 |
| VPS13B | 1.07 | 1.18E-04 |
| PRSS16 | 1.07 | 2.10E-03 |
| FYB2 | 1.07 | 3.41E-02 |
| RND1 | 1.07 | 4.38E-03 |
| PPT1 | 1.07 | 3.66E-06 |
| SETD4 | 1.07 | 2.50E-06 |
| GEN1 | 1.07 | 4.06E-05 |
| CLEC7A | 1.06 | 2.39E-03 |
| MBOAT1 | 1.06 | 8.16E-03 |
| CDC42SE1 | 1.06 | 5.26E-07 |
| STK35 | 1.06 | 6.93E-10 |
| CFL1 | 1.06 | 2.65E-13 |
| RHOT2 | 1.06 | 7.97E-10 |
| SETD1B | 1.06 | 8.16E-12 |
| SLC25A15 | 1.06 | 1.65E-05 |
| GPR157 | 1.06 | 5.23E-05 |
| ZNF165 | 1.06 | 1.65E-03 |
| CAD | 1.06 | 3.77E-07 |
| GAA | 1.06 | 8.56E-05 |
| ECE2 | 1.06 | 7.38E-04 |
| ATP2A3 | 1.06 | 1.58E-02 |
| SDR16C5 | 1.06 | 4.34E-02 |
| SMKR1 | 1.06 | 6.13E-04 |
| ZNF367 | 1.06 | 5.81E-06 |
| TMEM121 | 1.06 | 7.62E-05 |
| PPP1R9A | 1.06 | 3.24E-03 |
| PCNA | 1.06 | 9.77E-07 |
| FRMD6-AS1 | 1.06 | 3.10E-04 |
| BANF1 | 1.06 | 5.59E-08 |
| YTHDF1 | 1.05 | 9.91E-13 |
| LCMT1 | 1.05 | 4.83E-08 |
| ZMYND19 | 1.05 | 8.47E-07 |
| USP42 | 1.05 | 1.91E-04 |
| ASB13 | 1.05 | 7.09E-04 |
| ARFGEF1 | 1.05 | 8.93E-10 |
| ZXDC | 1.05 | 1.55E-07 |
| SLC44A5 | 1.05 | 4.37E-02 |
| RRBP1 | 1.05 | 2.80E-04 |
| CYP2R1 | 1.05 | 3.08E-05 |
| COL16A1 | 1.05 | 2.18E-03 |
| SH2D3A | 1.05 | 1.79E-05 |
| EP400 | 1.05 | 1.84E-02 |
| ANKRD50 | 1.05 | 6.55E-04 |
| C2CD3 | 1.05 | 6.22E-08 |
| LTBP3 | 1.05 | 9.05E-04 |
| CABLES2 | 1.05 | 1.29E-05 |
| GABBR1 | 1.05 | 2.34E-04 |
| PAQR6 | 1.05 | 5.46E-04 |
| ZNF26 | 1.05 | 2.62E-04 |
| TBX3 | 1.04 | 5.26E-03 |
| H2BC9 | 1.04 | 1.98E-03 |
| JMJD4 | 1.04 | 1.81E-05 |
| NAPEPLD | 1.04 | 2.88E-05 |
| NBEAL2 | 1.04 | 8.28E-06 |
| NAA40 | 1.04 | 4.61E-07 |
| PRR14 | 1.04 | 1.71E-05 |
| SMAGP | 1.04 | 6.07E-03 |
| U2AF2 | 1.04 | 8.73E-10 |
| HNRNPAB | 1.04 | 6.10E-07 |
| ZNF124 | 1.04 | 6.68E-07 |
| MCF2L | 1.04 | 1.87E-03 |
| ACTN4 | 1.04 | 1.48E-06 |
| ABHD2 | 1.04 | 2.41E-03 |
| JAK3 | 1.04 | 2.98E-02 |
| CCR5 | 1.04 | 1.20E-02 |
| HACD3 | 1.04 | 5.35E-04 |
| QSOX1 | 1.04 | 7.20E-04 |
| LOC100129917 | 1.04 | 1.19E-04 |
| AGAP1 | 1.04 | 1.19E-04 |
| WWC3 | 1.04 | 9.67E-06 |
| VANGL1 | 1.04 | 2.86E-07 |
| SAC3D1 | 1.03 | 3.75E-07 |
| NOP58 | 1.03 | 2.82E-07 |
| PCBP1-AS1 | 1.03 | 1.46E-05 |
| ZNF682 | 1.03 | 2.59E-04 |
| PFKP | 1.03 | 2.22E-02 |
| E2F8 | 1.03 | 6.54E-04 |
| CHD4 | 1.03 | 5.23E-13 |
| DCAF7 | 1.03 | 3.73E-07 |
| CREBZF | 1.03 | 1.47E-04 |
| PIMREG | 1.03 | 5.41E-04 |
| ZNF606 | 1.03 | 6.84E-04 |
| UBXN10-AS1 | 1.03 | 1.04E-02 |
| TEX9 | 1.03 | 6.17E-04 |
| CCDC152 | 1.03 | 7.07E-04 |
| CTSH | 1.03 | 2.20E-04 |
| ADSS2 | 1.03 | 7.71E-05 |
| HJURP | 1.03 | 9.66E-06 |
| CACNA2D2 | 1.03 | 6.70E-03 |
| PDPK1 | 1.02 | 1.38E-08 |
| SART3 | 1.02 | 5.55E-07 |
| SLC35F2 | 1.02 | 8.30E-04 |
| CSE1L | 1.02 | 2.22E-07 |
| NCOA3 | 1.02 | 3.28E-05 |
| KIAA1217 | 1.02 | 1.12E-04 |
| DSC2 | 1.02 | 4.91E-02 |
| TRERF1 | 1.02 | 6.75E-03 |
| CXorf40B | 1.02 | 9.73E-04 |
| NKTR | 1.02 | 1.53E-03 |
| CDCA7 | 1.02 | 3.53E-02 |
| B3GAT3 | 1.02 | 1.40E-05 |
| RHBDF2 | 1.02 | 6.48E-04 |
| BICD1 | 1.02 | 2.92E-04 |
| FBLIM1 | 1.02 | 1.24E-04 |
| DGKQ | 1.02 | 3.86E-05 |
| FGFR4 | 1.02 | 1.89E-02 |
| RALY | 1.02 | 2.77E-09 |
| PRRT2 | 1.02 | 9.15E-04 |
| NAPB | 1.02 | 1.27E-04 |
| MCM7 | 1.02 | 1.89E-06 |
| TRAFD1 | 1.02 | 9.16E-06 |
| GSR | 1.02 | 1.01E-03 |
| B3GALNT2 | 1.02 | 3.09E-04 |
| HLA-DQB2 | 1.02 | 1.50E-02 |
| HSPB1 | 1.01 | 4.06E-02 |
| TFRC | 1.01 | 1.29E-04 |
| EIF2AK3 | 1.01 | 3.19E-07 |
| RUFY3 | 1.01 | 2.39E-05 |
| TBC1D8 | 1.01 | 8.01E-04 |
| CCDC167 | 1.01 | 5.04E-05 |
| MTFP1 | 1.01 | 4.50E-05 |
| DCUN1D2 | 1.01 | 4.86E-05 |
| TAP2 | 1.01 | 4.03E-02 |
| DNAAF5 | 1.01 | 2.38E-06 |
| SLC35D1 | 1.01 | 2.77E-05 |
| CLUHP3 | 1.01 | 6.12E-07 |
| ZNF469 | 1.01 | 1.62E-03 |
| FHDC1 | 1.01 | 5.57E-05 |
| HTRA4 | 1.01 | 1.14E-02 |
| MAP3K21 | 1.01 | 4.50E-03 |
| SMS | 1.01 | 4.35E-05 |
| TAF4 | 1.01 | 2.95E-07 |
| HLA-F | 1.01 | 2.80E-03 |
| HOMER1 | 1.01 | 4.88E-03 |
| SEMA4D | 1.01 | 1.33E-04 |
| RGS16 | 1.01 | 1.28E-02 |
| PTCD3 | 1.01 | 4.41E-06 |
| ANKZF1 | 1.01 | 6.02E-06 |
| ODF2 | 1.01 | 1.39E-05 |
| CENPW | 1.01 | 7.20E-03 |
| TARBP1 | 1.01 | 8.42E-06 |
| FANCG | 1.00 | 6.73E-06 |
| CNPY3 | 1.00 | 1.68E-07 |
| RNPEP | 1.00 | 2.41E-04 |
| DDX56 | 1.00 | 3.07E-06 |
| PHF19 | 1.00 | 4.23E-03 |
| C9orf64 | 1.00 | 8.94E-04 |
| IRAK1 | 1.00 | 2.49E-05 |
| ACTR3 | 1.00 | 4.24E-07 |
| MGME1 | 1.00 | 2.27E-06 |
| UBE2Z | 1.00 | 3.42E-05 |
| TP53I11 | 1.00 | 5.07E-05 |
| FTL | -1.00 | 4.20E-08 |
| PRMT9 | -1.00 | 6.05E-11 |
| C11orf71 | -1.00 | 4.71E-05 |
| CCL4 | -1.00 | 2.52E-03 |
| OSGIN2 | -1.00 | 6.77E-04 |
| ZDHHC4 | -1.00 | 2.50E-03 |
| MEIS1 | -1.00 | 8.71E-05 |
| ABRAXAS1 | -1.00 | 5.83E-11 |
| EIF1B | -1.01 | 6.61E-05 |
| TTC37 | -1.01 | 4.55E-07 |
| TMEM245 | -1.01 | 1.02E-04 |
| POPDC3 | -1.01 | 5.55E-07 |
| SLU7 | -1.01 | 8.59E-06 |
| COL14A1 | -1.01 | 3.86E-02 |
| SMYD4 | -1.01 | 3.16E-09 |
| IL1R2 | -1.01 | 9.17E-03 |
| RAB32 | -1.01 | 7.86E-04 |
| RFLNB | -1.01 | 2.59E-04 |
| MXRA8 | -1.01 | 1.23E-02 |
| TBC1D15 | -1.01 | 1.13E-07 |
| TSC22D3 | -1.01 | 1.71E-03 |
| ORMDL3 | -1.01 | 1.06E-02 |
| LETMD1 | -1.01 | 1.76E-05 |
| SYDE1 | -1.02 | 1.22E-05 |
| ACER3 | -1.02 | 5.22E-04 |
| SLAIN2 | -1.02 | 1.04E-08 |
| NDNF | -1.02 | 6.77E-04 |
| AMZ2P1 | -1.02 | 9.77E-11 |
| JAK1 | -1.02 | 2.38E-05 |
| MCEE | -1.02 | 5.89E-07 |
| FER | -1.02 | 6.71E-11 |
| TFE3 | -1.02 | 6.22E-08 |
| COLEC11 | -1.02 | 3.50E-05 |
| FNDC3B | -1.02 | 4.52E-05 |
| TIMP3 | -1.02 | 2.48E-02 |
| VPS29 | -1.02 | 1.89E-05 |
| MT1HL1 | -1.02 | 3.92E-04 |
| NDUFAF3 | -1.02 | 1.81E-05 |
| PLCL1 | -1.02 | 1.05E-02 |
| LINC02381 | -1.02 | 4.83E-04 |
| KBTBD11 | -1.02 | 3.26E-08 |
| RBM18 | -1.02 | 2.67E-08 |
| CHURC1 | -1.03 | 1.60E-08 |
| GALT | -1.03 | 4.90E-07 |
| SESN3 | -1.03 | 1.82E-02 |
| MBIP | -1.03 | 4.06E-06 |
| BACE1 | -1.03 | 7.07E-06 |
| HBP1 | -1.03 | 2.86E-07 |
| LINC-PINT | -1.03 | 8.75E-04 |
| SNTB1 | -1.03 | 1.09E-05 |
| AIF1 | -1.03 | 1.52E-03 |
| AKAP11 | -1.03 | 1.11E-03 |
| TGDS | -1.03 | 8.79E-06 |
| IL10RA | -1.03 | 2.16E-02 |
| COPS4 | -1.03 | 5.58E-05 |
| GSTP1 | -1.03 | 4.47E-02 |
| RNF141 | -1.03 | 9.05E-04 |
| C1S | -1.03 | 1.19E-02 |
| FUOM | -1.03 | 1.07E-06 |
| HYMAI | -1.03 | 2.22E-06 |
| ATP6V0E2-AS1 | -1.03 | 9.28E-11 |
| GIMAP4 | -1.03 | 3.96E-05 |
| A2M-AS1 | -1.03 | 3.23E-14 |
| LNPEP | -1.04 | 8.47E-05 |
| IRF8 | -1.04 | 1.68E-02 |
| CRYZL1 | -1.04 | 1.72E-10 |
| PXMP2 | -1.04 | 1.37E-05 |
| SEPTIN4 | -1.04 | 5.31E-08 |
| ARHGEF10 | -1.04 | 4.74E-04 |
| EPS15 | -1.04 | 9.32E-08 |
| FGFR1 | -1.04 | 5.00E-04 |
| TMEM120A | -1.04 | 1.65E-05 |
| UVRAG | -1.04 | 3.27E-06 |
| ALKAL2 | -1.04 | 1.26E-02 |
| COQ3 | -1.04 | 4.67E-04 |
| EGR2 | -1.04 | 1.44E-03 |
| CCSER2 | -1.04 | 9.06E-06 |
| FFAR4 | -1.04 | 9.80E-17 |
| NKAPL | -1.04 | 8.43E-14 |
| ZFHX4-AS1 | -1.04 | 1.46E-08 |
| BHLHE22 | -1.05 | 1.67E-05 |
| RRAGC | -1.05 | 2.14E-08 |
| ELL2 | -1.05 | 4.48E-04 |
| RMDN1 | -1.05 | 3.20E-05 |
| PEAR1 | -1.05 | 2.79E-09 |
| CAVIN3 | -1.05 | 2.84E-04 |
| GCLM | -1.05 | 1.16E-04 |
| ESD | -1.05 | 2.94E-07 |
| LOC100506388 | -1.05 | 2.44E-12 |
| SPAG16 | -1.05 | 4.78E-03 |
| GSTM2 | -1.05 | 1.16E-02 |
| EMP3 | -1.05 | 2.04E-03 |
| S100A1 | -1.05 | 1.15E-07 |
| BANK1 | -1.05 | 6.79E-07 |
| PRG4 | -1.05 | 5.03E-08 |
| PPM1L | -1.05 | 4.24E-09 |
| BPHL | -1.05 | 1.10E-06 |
| PTH1R | -1.05 | 3.53E-15 |
| TTC28 | -1.06 | 8.22E-04 |
| POLR2E | -1.06 | 1.08E-06 |
| NUDCD2 | -1.06 | 9.87E-06 |
| TPST2 | -1.06 | 1.83E-03 |
| TNFSF8 | -1.06 | 5.30E-04 |
| TOMM22 | -1.06 | 3.06E-04 |
| WTIP | -1.06 | 1.65E-06 |
| BBS7 | -1.06 | 5.23E-07 |
| SMIM4 | -1.06 | 1.45E-07 |
| RNF11 | -1.06 | 1.44E-06 |
| PTPN11 | -1.06 | 1.38E-12 |
| CCL18 | -1.06 | 2.74E-02 |
| CRADD | -1.06 | 1.70E-08 |
| DBP | -1.06 | 8.07E-03 |
| ATE1 | -1.06 | 4.19E-06 |
| CNTFR-AS1 | -1.06 | 1.54E-16 |
| POP5 | -1.06 | 1.56E-06 |
| LONRF1 | -1.06 | 1.18E-04 |
| UQCRFS1 | -1.07 | 9.41E-04 |
| COQ10A | -1.07 | 4.93E-05 |
| C1QC | -1.07 | 5.00E-03 |
| NHLRC3 | -1.07 | 6.24E-07 |
| PGRMC2 | -1.07 | 2.70E-05 |
| POLK | -1.07 | 1.23E-10 |
| SGPP1 | -1.07 | 8.06E-04 |
| C1QTNF2 | -1.07 | 7.61E-10 |
| NDUFS1 | -1.07 | 8.53E-09 |
| RTN3 | -1.07 | 8.83E-05 |
| POLE4 | -1.07 | 5.59E-05 |
| GPHN | -1.07 | 1.01E-03 |
| COMMD6 | -1.07 | 1.87E-05 |
| SNTG2-AS1 | -1.07 | 1.77E-03 |
| CLU | -1.07 | 2.86E-02 |
| TMEM255A | -1.07 | 5.75E-07 |
| KIAA1324L | -1.07 | 1.45E-03 |
| PTEN | -1.07 | 2.32E-04 |
| CPXM2 | -1.07 | 1.54E-05 |
| ARHGAP5 | -1.07 | 5.00E-04 |
| CXCL8 | -1.07 | 4.26E-02 |
| SUCLG2 | -1.08 | 4.27E-06 |
| PRXL2C | -1.08 | 2.25E-07 |
| SGCD | -1.08 | 1.27E-04 |
| AK6 | -1.08 | 6.19E-07 |
| MANEA | -1.08 | 1.66E-04 |
| JRKL | -1.08 | 1.89E-03 |
| CHEK1 | -1.08 | 2.01E-06 |
| ZNF271P | -1.08 | 3.89E-05 |
| LIMCH1 | -1.08 | 1.71E-02 |
| NDUFV3 | -1.08 | 3.97E-04 |
| LLPH | -1.08 | 1.79E-05 |
| ZHX1 | -1.08 | 4.69E-09 |
| ITGA9 | -1.08 | 7.86E-03 |
| RNLS | -1.08 | 6.45E-07 |
| SLC2A3 | -1.08 | 8.27E-03 |
| TPST1 | -1.08 | 1.56E-06 |
| CPPED1 | -1.09 | 1.90E-04 |
| DHRS11 | -1.09 | 9.56E-03 |
| HGF | -1.09 | 2.12E-04 |
| RGS7BP | -1.09 | 1.42E-10 |
| FNIP2 | -1.09 | 1.39E-05 |
| KLF3 | -1.09 | 7.01E-04 |
| ITPK1 | -1.09 | 3.54E-06 |
| RUNDC3B | -1.09 | 1.76E-06 |
| MPDZ | -1.09 | 3.77E-06 |
| PER3 | -1.09 | 1.62E-03 |
| HK2 | -1.09 | 9.17E-04 |
| CDC26 | -1.09 | 1.51E-05 |
| SLC30A4 | -1.09 | 1.11E-04 |
| PI16 | -1.09 | 1.24E-09 |
| BHLHE41 | -1.09 | 1.42E-02 |
| AUH | -1.09 | 4.84E-05 |
| PMM1 | -1.09 | 1.10E-07 |
| ADAMTS3 | -1.09 | 8.15E-05 |
| GYS2 | -1.09 | 8.96E-09 |
| HINT2 | -1.10 | 3.73E-07 |
| OGFRL1 | -1.10 | 1.92E-03 |
| MAN1C1 | -1.10 | 2.71E-04 |
| NAALADL1 | -1.10 | 5.94E-12 |
| SMIM10 | -1.10 | 3.20E-11 |
| UBXN8 | -1.10 | 1.88E-07 |
| MYO1C | -1.10 | 3.78E-08 |
| GNPDA2 | -1.10 | 3.23E-07 |
| SNRNP27 | -1.10 | 5.47E-11 |
| ERCC8 | -1.10 | 1.03E-10 |
| PRDX3 | -1.10 | 6.54E-04 |
| FAM13A-AS1 | -1.10 | 1.08E-06 |
| MLYCD | -1.10 | 4.64E-09 |
| GCSH | -1.10 | 1.12E-06 |
| TMEM120B | -1.10 | 1.29E-06 |
| PIM1 | -1.10 | 3.78E-05 |
| LOC100506476 | -1.10 | 5.94E-17 |
| SH2B3 | -1.10 | 9.98E-05 |
| CC2D2A | -1.10 | 3.42E-10 |
| PHGDH | -1.10 | 8.77E-03 |
| DDHD2 | -1.10 | 4.49E-04 |
| HBD | -1.10 | 1.30E-08 |
| POGLUT3 | -1.10 | 1.22E-05 |
| PHKA2 | -1.11 | 5.53E-06 |
| CCDC28A | -1.11 | 1.34E-05 |
| KIAA1107 | -1.11 | 1.08E-07 |
| NHLRC2 | -1.11 | 3.49E-05 |
| MED11 | -1.11 | 1.55E-08 |
| EOGT | -1.11 | 3.86E-05 |
| ACO2 | -1.11 | 1.65E-04 |
| CRIM1 | -1.11 | 2.34E-03 |
| DCUN1D4 | -1.11 | 1.57E-06 |
| AZI2 | -1.11 | 9.77E-11 |
| EIF4EBP2 | -1.11 | 1.80E-08 |
| SLC25A1 | -1.11 | 4.23E-03 |
| TUBB2A | -1.11 | 1.14E-03 |
| SLC26A7 | -1.11 | 6.33E-03 |
| PTPRS | -1.11 | 2.56E-04 |
| COPG2 | -1.11 | 1.67E-06 |
| KLHDC1 | -1.11 | 3.96E-05 |
| TMED5 | -1.12 | 1.63E-05 |
| EFEMP2 | -1.12 | 5.76E-05 |
| FITM2 | -1.12 | 1.08E-03 |
| HIBCH | -1.12 | 3.06E-07 |
| BCAR3 | -1.12 | 3.88E-04 |
| MBD2 | -1.12 | 9.64E-10 |
| CEP63 | -1.12 | 4.90E-07 |
| CD200 | -1.12 | 6.77E-05 |
| SGTB | -1.12 | 2.27E-05 |
| RAB9A | -1.12 | 5.88E-07 |
| RPH3AL | -1.12 | 9.48E-06 |
| NCKAP1L | -1.12 | 4.37E-03 |
| CHMP2B | -1.12 | 8.11E-06 |
| PPM1M | -1.12 | 7.68E-06 |
| TM4SF18 | -1.12 | 1.77E-03 |
| LOXL4 | -1.13 | 5.35E-04 |
| LINC00312 | -1.13 | 2.25E-07 |
| CYB5D2 | -1.13 | 1.99E-06 |
| GNG12 | -1.13 | 5.05E-03 |
| CD14 | -1.13 | 2.44E-02 |
| C1QTNF7 | -1.13 | 8.96E-19 |
| SLC25A46 | -1.13 | 7.22E-05 |
| CYB5R3 | -1.13 | 1.57E-08 |
| ADI1 | -1.13 | 4.08E-04 |
| FBXO25 | -1.13 | 1.33E-04 |
| CS | -1.13 | 8.97E-09 |
| TSPAN7 | -1.13 | 6.18E-06 |
| FAM171A1 | -1.13 | 1.19E-02 |
| SLC66A3 | -1.13 | 7.31E-03 |
| NAB1 | -1.13 | 5.65E-05 |
| IRAK2 | -1.13 | 5.43E-04 |
| GATA6 | -1.13 | 7.81E-03 |
| METTL5 | -1.13 | 1.11E-11 |
| ELAC1 | -1.13 | 1.77E-06 |
| EML1 | -1.13 | 2.06E-08 |
| NDRG2 | -1.13 | 7.14E-03 |
| BCL2 | -1.14 | 3.01E-02 |
| CIB2 | -1.14 | 1.74E-05 |
| MRPL46 | -1.14 | 7.62E-07 |
| PRIMA1 | -1.14 | 6.61E-06 |
| USP30 | -1.14 | 5.23E-09 |
| GPRASP1 | -1.14 | 6.74E-07 |
| MBNL1 | -1.14 | 1.16E-10 |
| DUSP14 | -1.14 | 3.27E-04 |
| CEP68 | -1.14 | 9.78E-07 |
| BTBD6 | -1.15 | 1.72E-08 |
| LOC105379426 | -1.15 | 9.67E-04 |
| GATB | -1.15 | 3.43E-07 |
| DOCK6 | -1.15 | 9.86E-07 |
| LOC101929398 | -1.15 | 3.31E-16 |
| PKIG | -1.15 | 2.73E-05 |
| LOC285095 | -1.15 | 1.59E-08 |
| AASS | -1.15 | 3.23E-10 |
| BTD | -1.15 | 2.18E-06 |
| CYB5R4 | -1.15 | 1.06E-07 |
| PLEKHA8P1 | -1.15 | 1.79E-06 |
| NID2 | -1.15 | 5.31E-03 |
| PTCD2 | -1.15 | 1.94E-11 |
| ABHD14B | -1.15 | 9.45E-07 |
| HOTS | -1.16 | 4.29E-03 |
| NFIA | -1.16 | 7.22E-03 |
| LRMP | -1.16 | 2.05E-02 |
| ZBED3 | -1.16 | 1.60E-07 |
| ANKRD46 | -1.16 | 9.74E-05 |
| C9orf24 | -1.16 | 1.89E-15 |
| COPS2 | -1.16 | 1.62E-09 |
| COL5A3 | -1.16 | 7.34E-05 |
| OSBPL11 | -1.16 | 4.75E-05 |
| IDH1 | -1.16 | 7.31E-06 |
| MYL5 | -1.16 | 2.93E-06 |
| C1QTNF1 | -1.16 | 2.65E-19 |
| MTFR1L | -1.16 | 7.93E-12 |
| MID2 | -1.16 | 3.89E-13 |
| C1QB | -1.16 | 4.19E-02 |
| PTGFR | -1.17 | 5.36E-11 |
| SETD9 | -1.17 | 1.19E-03 |
| HCFC2 | -1.17 | 1.25E-09 |
| ST3GAL6 | -1.17 | 4.66E-03 |
| ID1 | -1.17 | 2.55E-03 |
| LOC100289361 | -1.17 | 4.13E-10 |
| NABP1 | -1.17 | 6.56E-04 |
| MAP1B | -1.17 | 3.68E-03 |
| CSRNP1 | -1.17 | 8.83E-07 |
| MAP1LC3B | -1.17 | 1.92E-07 |
| EZH1 | -1.17 | 3.97E-09 |
| PINK1 | -1.17 | 4.05E-08 |
| UFSP2 | -1.18 | 1.27E-08 |
| TSPAN4 | -1.18 | 5.32E-06 |
| LPP-AS2 | -1.18 | 1.20E-16 |
| DEFB1 | -1.18 | 2.09E-02 |
| TEF | -1.18 | 5.67E-05 |
| NCF2 | -1.18 | 4.93E-03 |
| FAM43A | -1.18 | 5.74E-07 |
| FAM49A | -1.18 | 1.31E-09 |
| LPIN1 | -1.19 | 7.52E-04 |
| NRP2 | -1.19 | 6.92E-04 |
| AQP1 | -1.19 | 6.33E-03 |
| AKT3 | -1.19 | 1.61E-04 |
| IL17D | -1.19 | 2.14E-11 |
| SDCBP | -1.19 | 1.52E-06 |
| HLA-F-AS1 | -1.19 | 3.05E-09 |
| LGALS3 | -1.19 | 1.59E-05 |
| NDUFAF1 | -1.19 | 1.31E-07 |
| DGAT1 | -1.20 | 5.58E-04 |
| GLUD2 | -1.20 | 1.92E-08 |
| CARD16 | -1.20 | 1.39E-03 |
| SCML1 | -1.20 | 4.13E-03 |
| C6orf120 | -1.20 | 2.28E-06 |
| DHRS7B | -1.20 | 5.51E-06 |
| CILP | -1.20 | 4.08E-02 |
| ARRDC3 | -1.20 | 5.34E-05 |
| C4orf3 | -1.20 | 7.77E-07 |
| RELN | -1.20 | 3.15E-04 |
| MAP3K8 | -1.20 | 4.96E-04 |
| CD69 | -1.21 | 9.23E-03 |
| SIRT1 | -1.21 | 5.27E-06 |
| DIP2C | -1.21 | 1.62E-05 |
| BCL2L13 | -1.21 | 7.95E-08 |
| MAP2K1 | -1.21 | 8.26E-06 |
| STOX1 | -1.21 | 8.30E-07 |
| KLHL13 | -1.21 | 1.65E-02 |
| EGR3 | -1.21 | 2.74E-03 |
| PDZD8 | -1.21 | 6.60E-07 |
| CST3 | -1.21 | 4.03E-02 |
| PDE8A | -1.21 | 3.61E-06 |
| SMIM19 | -1.21 | 7.93E-06 |
| IRAK3 | -1.21 | 2.23E-05 |
| DPYSL2 | -1.21 | 6.38E-05 |
| SCO1 | -1.21 | 2.62E-04 |
| PTH2R | -1.21 | 1.16E-04 |
| EXOC6B | -1.21 | 1.20E-09 |
| ZNF226 | -1.21 | 3.92E-05 |
| CCL13 | -1.22 | 2.02E-07 |
| NINJ2-AS1 | -1.22 | 1.66E-04 |
| BORCS7 | -1.22 | 2.15E-05 |
| LRFN5 | -1.22 | 6.13E-13 |
| RBM7 | -1.22 | 4.76E-07 |
| NR4A1 | -1.22 | 5.04E-04 |
| TRAM2-AS1 | -1.22 | 2.71E-08 |
| PEAK1 | -1.22 | 3.36E-05 |
| ZNF521 | -1.22 | 2.26E-03 |
| CPNE2 | -1.22 | 6.35E-04 |
| C15orf40 | -1.22 | 1.68E-05 |
| PIGP | -1.22 | 1.47E-06 |
| SVBP | -1.22 | 2.58E-08 |
| GPN3 | -1.22 | 2.50E-12 |
| TKT | -1.22 | 4.27E-06 |
| SUCLG1 | -1.22 | 2.14E-22 |
| C1GALT1C1L | -1.23 | 1.72E-09 |
| NCF4 | -1.23 | 4.48E-04 |
| SMAD6 | -1.23 | 5.06E-05 |
| OLFML3 | -1.23 | 9.49E-03 |
| LINC00310 | -1.23 | 2.04E-16 |
| CSRP2 | -1.23 | 3.52E-04 |
| SPARCL1 | -1.23 | 1.79E-02 |
| GEM | -1.23 | 7.45E-03 |
| MAFF | -1.23 | 2.56E-03 |
| RASSF3 | -1.23 | 3.01E-08 |
| LOC101927752 | -1.23 | 1.09E-04 |
| FCER1A | -1.23 | 2.62E-06 |
| ME3 | -1.23 | 3.16E-05 |
| TMEM243 | -1.23 | 8.80E-05 |
| RPP30 | -1.23 | 1.38E-08 |
| SNRK | -1.23 | 4.83E-08 |
| TEAD1 | -1.23 | 5.64E-05 |
| ST3GAL3 | -1.23 | 7.50E-09 |
| CPEB1 | -1.23 | 4.66E-11 |
| KCTD9 | -1.23 | 6.51E-07 |
| BNIP3 | -1.23 | 7.20E-04 |
| C1orf115 | -1.23 | 2.60E-03 |
| CCN3 | -1.24 | 1.86E-04 |
| ROBO1 | -1.24 | 7.31E-05 |
| CEBPD | -1.24 | 9.57E-04 |
| LHCGR | -1.24 | 6.98E-12 |
| FMNL2 | -1.24 | 9.78E-04 |
| CRK | -1.24 | 1.33E-11 |
| VAMP3 | -1.24 | 1.23E-11 |
| ST6GALNAC6 | -1.24 | 1.71E-21 |
| KCND3 | -1.24 | 2.83E-02 |
| TST | -1.24 | 3.36E-04 |
| CSGALNACT1 | -1.24 | 3.82E-04 |
| BCAT1 | -1.24 | 8.63E-03 |
| SEMA6D | -1.24 | 1.03E-02 |
| RMND1 | -1.24 | 6.02E-04 |
| 2-Mar | -1.24 | 1.92E-07 |
| ANXA6 | -1.25 | 7.09E-07 |
| GIMAP7 | -1.25 | 3.30E-03 |
| CHST7 | -1.25 | 2.94E-12 |
| AKR1B1 | -1.25 | 5.85E-05 |
| CGRRF1 | -1.25 | 4.36E-08 |
| MYRIP | -1.25 | 1.30E-04 |
| SRR | -1.25 | 3.35E-08 |
| NIPSNAP3A | -1.25 | 6.20E-06 |
| PEX11A | -1.25 | 8.07E-06 |
| CASP4 | -1.25 | 2.43E-04 |
| TPPP | -1.25 | 5.35E-04 |
| IMMP2L | -1.25 | 9.03E-09 |
| SNCG | -1.25 | 1.14E-11 |
| SEMA3A | -1.25 | 8.94E-16 |
| MSANTD4 | -1.25 | 2.37E-06 |
| ADH1A | -1.26 | 8.38E-18 |
| STX8 | -1.26 | 7.25E-10 |
| SFTA1P | -1.26 | 1.46E-13 |
| SNN | -1.26 | 9.73E-08 |
| PLP1 | -1.26 | 7.52E-06 |
| GAS7 | -1.26 | 5.55E-04 |
| EVI5 | -1.26 | 1.81E-11 |
| SC5D | -1.26 | 3.20E-03 |
| HACD2 | -1.26 | 9.03E-09 |
| CHP1 | -1.26 | 1.72E-12 |
| DAPK2 | -1.26 | 1.79E-09 |
| THBD | -1.26 | 8.27E-04 |
| MPP6 | -1.26 | 7.59E-08 |
| STAMBPL1 | -1.26 | 3.61E-04 |
| EMC3 | -1.26 | 7.99E-05 |
| AVEN | -1.26 | 2.08E-08 |
| C16orf54 | -1.26 | 1.58E-02 |
| TENM1 | -1.26 | 2.71E-09 |
| ATPAF1 | -1.26 | 6.38E-06 |
| CPE | -1.26 | 3.15E-03 |
| PROCR | -1.27 | 2.10E-11 |
| JADE1 | -1.27 | 3.91E-07 |
| PLEKHH2 | -1.27 | 3.72E-04 |
| MARCHF3 | -1.27 | 1.91E-04 |
| F10 | -1.27 | 2.13E-15 |
| GINM1 | -1.27 | 5.29E-12 |
| PYROXD2 | -1.27 | 7.89E-05 |
| NNT-AS1 | -1.27 | 4.20E-09 |
| CCNG1 | -1.27 | 5.52E-07 |
| TNN | -1.27 | 7.15E-04 |
| ANAPC10 | -1.27 | 3.61E-10 |
| PNRC1 | -1.27 | 4.63E-08 |
| NRBF2 | -1.27 | 6.58E-07 |
| ZNF91 | -1.27 | 8.50E-03 |
| C5AR1 | -1.28 | 1.93E-05 |
| GLRX3 | -1.28 | 1.17E-05 |
| RGS17 | -1.28 | 3.01E-14 |
| STAB1 | -1.28 | 1.58E-05 |
| GTF2E2 | -1.28 | 1.40E-06 |
| ECH1 | -1.28 | 1.49E-08 |
| ERG | -1.28 | 3.22E-07 |
| RAB8B | -1.28 | 1.68E-05 |
| ADH5 | -1.28 | 2.65E-17 |
| CCDC71L | -1.28 | 7.37E-05 |
| SEC23A | -1.29 | 4.93E-07 |
| ERAP1 | -1.29 | 1.61E-06 |
| NQO1 | -1.29 | 4.88E-03 |
| LAMC1 | -1.29 | 1.30E-04 |
| FASN | -1.29 | 1.57E-03 |
| STEAP2 | -1.29 | 1.35E-03 |
| RERGL | -1.29 | 6.59E-06 |
| FEZ2 | -1.29 | 6.08E-08 |
| AXL | -1.29 | 7.91E-04 |
| FBN1 | -1.29 | 1.51E-03 |
| CRTAP | -1.29 | 6.31E-07 |
| NDUFAF4 | -1.29 | 6.49E-06 |
| SEMA3C | -1.29 | 1.62E-02 |
| HTR2B | -1.29 | 1.61E-08 |
| MS4A6A | -1.29 | 1.36E-03 |
| PXDC1 | -1.29 | 2.00E-06 |
| BCL2L2 | -1.29 | 1.29E-06 |
| ZNF667-AS1 | -1.29 | 5.11E-03 |
| DLAT | -1.29 | 2.59E-11 |
| ITGAE | -1.29 | 6.14E-12 |
| NQO2 | -1.29 | 1.52E-04 |
| OR51E1 | -1.30 | 1.87E-15 |
| DTD2 | -1.30 | 1.18E-07 |
| MT1X | -1.30 | 6.80E-05 |
| TIPARP | -1.30 | 2.05E-05 |
| USP25 | -1.30 | 2.23E-08 |
| DMAC2L | -1.30 | 9.55E-06 |
| C4orf19 | -1.30 | 4.78E-04 |
| YAP1 | -1.30 | 1.01E-04 |
| RNF145 | -1.30 | 1.86E-03 |
| PLOD2 | -1.30 | 8.19E-04 |
| CAST | -1.30 | 1.56E-06 |
| RASSF4 | -1.30 | 3.21E-03 |
| ATL3 | -1.30 | 1.48E-08 |
| LATS2 | -1.30 | 5.39E-07 |
| MCTP1 | -1.30 | 2.83E-05 |
| CREBL2 | -1.31 | 2.04E-09 |
| WASHC3 | -1.31 | 1.11E-08 |
| KLF9 | -1.31 | 1.96E-05 |
| KCNJ5 | -1.31 | 7.33E-07 |
| MPPED2 | -1.31 | 4.44E-03 |
| TBX5 | -1.31 | 1.45E-07 |
| CYS1 | -1.31 | 2.18E-08 |
| USP53 | -1.31 | 3.15E-06 |
| MMGT1 | -1.31 | 1.68E-06 |
| CD99L2 | -1.31 | 1.60E-06 |
| CR1 | -1.31 | 6.84E-07 |
| ECHS1 | -1.32 | 7.15E-11 |
| PTPN4 | -1.32 | 1.70E-08 |
| LGMN | -1.32 | 2.93E-04 |
| LMO2 | -1.32 | 3.25E-05 |
| SNX3 | -1.32 | 8.59E-14 |
| SNX2 | -1.32 | 2.23E-14 |
| TENM4 | -1.32 | 5.54E-09 |
| ETFRF1 | -1.32 | 4.99E-09 |
| FZD4-DT | -1.33 | 5.59E-18 |
| AGTPBP1 | -1.33 | 1.12E-08 |
| ACSL5 | -1.33 | 2.90E-04 |
| EEA1 | -1.33 | 1.44E-07 |
| MOB3C | -1.34 | 1.17E-09 |
| EPHA3 | -1.34 | 1.40E-09 |
| UBL3 | -1.34 | 3.07E-07 |
| RIMKLB | -1.34 | 1.09E-08 |
| SLC16A2 | -1.34 | 1.82E-07 |
| ZYG11B | -1.34 | 6.38E-08 |
| LACC1 | -1.34 | 5.46E-06 |
| ST6GALNAC3 | -1.34 | 3.02E-17 |
| FOXN3-AS1 | -1.34 | 1.67E-12 |
| MAP3K5 | -1.35 | 2.61E-06 |
| BOC | -1.35 | 2.90E-03 |
| FDX1 | -1.35 | 1.21E-05 |
| DMTN | -1.35 | 8.68E-17 |
| MACROD2 | -1.35 | 2.41E-04 |
| SERPING1 | -1.35 | 7.84E-04 |
| GFPT2 | -1.35 | 1.10E-08 |
| NKIRAS1 | -1.35 | 7.79E-10 |
| FAM172A | -1.35 | 1.71E-06 |
| TMEM126B | -1.36 | 4.50E-05 |
| CORO1C | -1.36 | 1.61E-08 |
| RGS3 | -1.36 | 5.97E-10 |
| MTLN | -1.36 | 5.49E-08 |
| CPA3 | -1.36 | 5.48E-04 |
| LIN7A | -1.36 | 1.70E-03 |
| VSTM4 | -1.36 | 1.06E-16 |
| SH3BP5 | -1.36 | 4.89E-08 |
| CD58 | -1.36 | 8.60E-06 |
| NR2F1-AS1 | -1.36 | 1.35E-08 |
| RAI2 | -1.36 | 8.59E-03 |
| CAPZA2 | -1.36 | 5.82E-08 |
| PHACTR2 | -1.36 | 1.15E-07 |
| NMRK1 | -1.36 | 1.75E-06 |
| LOC100286925 | -1.36 | 4.21E-10 |
| CTH | -1.36 | 5.45E-07 |
| SLC31A2 | -1.36 | 6.92E-07 |
| MGST3 | -1.36 | 5.05E-06 |
| IGIP | -1.37 | 3.34E-06 |
| PHLDA3 | -1.37 | 1.46E-18 |
| ADAMTSL3 | -1.37 | 9.88E-17 |
| CLYBL | -1.37 | 2.85E-09 |
| ECI2 | -1.37 | 9.78E-04 |
| MRPS36 | -1.37 | 8.25E-08 |
| PRADC1 | -1.38 | 8.95E-07 |
| PCYOX1 | -1.38 | 1.28E-05 |
| UBE2E3 | -1.38 | 1.10E-03 |
| RAB7B | -1.38 | 2.02E-06 |
| SDHAF3 | -1.38 | 1.29E-05 |
| ALG9 | -1.38 | 1.47E-11 |
| TSKU | -1.38 | 1.29E-06 |
| NINJ2 | -1.38 | 1.86E-05 |
| ARHGEF28 | -1.38 | 6.59E-06 |
| FILNC1 | -1.38 | 1.35E-15 |
| MMP28 | -1.38 | 1.11E-24 |
| OPTN | -1.38 | 1.54E-07 |
| ISCU | -1.39 | 6.55E-15 |
| NLGN1 | -1.39 | 1.70E-19 |
| SLC9A6 | -1.39 | 8.61E-06 |
| CTSG | -1.39 | 4.88E-10 |
| PRRG1 | -1.39 | 1.90E-06 |
| IRS1 | -1.39 | 1.85E-03 |
| SBF2 | -1.39 | 9.25E-07 |
| ISM1 | -1.39 | 1.62E-03 |
| DNAJC15 | -1.39 | 6.02E-06 |
| PLEKHG6 | -1.39 | 3.14E-09 |
| OAF | -1.39 | 7.37E-10 |
| HPGD | -1.40 | 7.18E-05 |
| SESTD1 | -1.40 | 1.54E-05 |
| BCKDHB | -1.40 | 4.16E-08 |
| HEATR5A | -1.40 | 2.08E-05 |
| GIMAP6 | -1.40 | 3.29E-06 |
| EPB41L1 | -1.40 | 2.08E-05 |
| LSM6 | -1.40 | 9.91E-13 |
| SNED1 | -1.40 | 2.32E-06 |
| CCBE1 | -1.40 | 3.40E-11 |
| PRRX1 | -1.40 | 5.28E-04 |
| HOXB7 | -1.40 | 2.10E-03 |
| SOD3 | -1.40 | 5.70E-12 |
| PLS3 | -1.40 | 6.66E-05 |
| SYNC | -1.40 | 7.10E-05 |
| ARRDC4 | -1.40 | 1.25E-05 |
| CZIB | -1.40 | 4.21E-16 |
| LYRM7 | -1.40 | 5.83E-08 |
| CLDN11 | -1.41 | 6.29E-03 |
| TSPAN8 | -1.41 | 1.71E-02 |
| RILP | -1.41 | 1.61E-13 |
| MECR | -1.41 | 2.22E-09 |
| GIMAP1 | -1.41 | 8.42E-04 |
| TMEM218 | -1.41 | 6.22E-07 |
| FNDC4 | -1.41 | 3.66E-13 |
| RABL3 | -1.41 | 7.05E-09 |
| MRPL2 | -1.41 | 4.01E-08 |
| NUDT6 | -1.41 | 5.68E-08 |
| STX12 | -1.42 | 1.23E-08 |
| COX14 | -1.42 | 2.36E-12 |
| CSF1R | -1.42 | 5.21E-05 |
| GPX1 | -1.42 | 5.79E-07 |
| ARHGAP31 | -1.42 | 1.06E-13 |
| RHOU | -1.42 | 1.91E-03 |
| PXK | -1.42 | 1.26E-09 |
| CH25H | -1.42 | 2.02E-06 |
| FLJ20021 | -1.43 | 2.41E-07 |
| LPAR6 | -1.43 | 1.87E-07 |
| CCN1 | -1.43 | 1.14E-04 |
| POLR2L | -1.43 | 1.32E-07 |
| ARHGAP42 | -1.43 | 1.97E-13 |
| ESYT1 | -1.43 | 2.96E-10 |
| PPARGC1A | -1.43 | 2.34E-05 |
| GAB2 | -1.43 | 2.80E-09 |
| HDDC2 | -1.43 | 3.25E-07 |
| MARK1 | -1.44 | 7.33E-11 |
| RAB11B-AS1 | -1.44 | 2.81E-11 |
| HDAC4-AS1 | -1.44 | 6.32E-16 |
| CYB5A | -1.44 | 2.04E-04 |
| UBA2 | -1.44 | 6.29E-16 |
| SMAD9 | -1.44 | 2.00E-07 |
| EMX2OS | -1.44 | 6.89E-16 |
| RAB29 | -1.44 | 2.82E-05 |
| MPP1 | -1.44 | 4.64E-08 |
| HOXD8 | -1.44 | 2.26E-05 |
| AGPS | -1.44 | 1.24E-04 |
| FAM126A | -1.44 | 1.01E-05 |
| HABP4 | -1.44 | 3.45E-16 |
| GID4 | -1.45 | 1.97E-11 |
| CDC42EP2 | -1.45 | 4.54E-20 |
| DHRS9 | -1.45 | 1.46E-05 |
| CPXM1 | -1.45 | 2.97E-04 |
| CD34 | -1.45 | 5.10E-17 |
| ARL2 | -1.45 | 2.25E-10 |
| CEP126 | -1.45 | 1.64E-04 |
| GIPC2 | -1.46 | 6.09E-17 |
| CFAP300 | -1.46 | 8.20E-20 |
| CYGB | -1.46 | 1.99E-10 |
| SATB1 | -1.46 | 1.19E-04 |
| SLC41A2 | -1.46 | 4.85E-06 |
| MTIF3 | -1.46 | 3.64E-13 |
| PELI2 | -1.46 | 1.39E-10 |
| PPP1R36 | -1.46 | 1.74E-06 |
| PTGR1 | -1.46 | 1.18E-05 |
| CMA1 | -1.46 | 8.73E-20 |
| PPP1R15A | -1.46 | 8.43E-09 |
| RNF130 | -1.46 | 7.46E-06 |
| KLHL29 | -1.46 | 1.62E-05 |
| ZNF438 | -1.46 | 1.65E-11 |
| UTRN | -1.47 | 3.34E-09 |
| SOCS2 | -1.47 | 5.35E-04 |
| IDNK | -1.47 | 1.62E-09 |
| SNTA1 | -1.47 | 1.19E-09 |
| ALPK3 | -1.47 | 6.95E-14 |
| HIBADH | -1.47 | 5.16E-05 |
| TNFRSF21 | -1.47 | 1.10E-03 |
| TLCD4 | -1.47 | 1.36E-07 |
| IL11RA | -1.47 | 5.18E-12 |
| FBXL5 | -1.47 | 6.24E-08 |
| MAP4K3-DT | -1.48 | 9.56E-09 |
| KLHDC8B | -1.48 | 4.02E-12 |
| RHOJ | -1.48 | 1.17E-05 |
| RDX | -1.48 | 1.88E-08 |
| PCGF5 | -1.48 | 1.00E-07 |
| CCDC141 | -1.48 | 5.81E-18 |
| LRMDA | -1.48 | 2.68E-07 |
| INPP5K | -1.48 | 4.47E-10 |
| DHRS12 | -1.48 | 2.41E-16 |
| PLPP7 | -1.48 | 1.49E-19 |
| ASPH | -1.48 | 1.37E-04 |
| FGF13 | -1.48 | 9.07E-04 |
| PCNX1 | -1.49 | 2.28E-05 |
| TMEM126A | -1.49 | 1.19E-05 |
| ASS1 | -1.49 | 7.38E-03 |
| PPP3CB | -1.49 | 1.92E-13 |
| CASP1 | -1.49 | 8.75E-04 |
| HEBP2 | -1.49 | 6.49E-07 |
| FMO1 | -1.49 | 2.73E-04 |
| CRYL1 | -1.49 | 8.91E-08 |
| MAML2 | -1.49 | 2.20E-04 |
| ABCA1 | -1.49 | 1.28E-12 |
| PIK3CA | -1.50 | 1.03E-09 |
| ROBO4 | -1.50 | 2.63E-08 |
| CDON | -1.50 | 1.42E-08 |
| NUBPL | -1.50 | 2.69E-13 |
| CLEC10A | -1.50 | 3.17E-06 |
| SLC25A51 | -1.50 | 2.96E-18 |
| FAM228A | -1.50 | 4.77E-15 |
| RTN1 | -1.50 | 1.36E-02 |
| ARHGAP28 | -1.50 | 1.04E-09 |
| DUSP6 | -1.50 | 8.63E-05 |
| COL15A1 | -1.50 | 5.38E-04 |
| PEPD | -1.50 | 3.97E-09 |
| STXBP1 | -1.50 | 6.61E-06 |
| ZFHX4 | -1.50 | 1.35E-15 |
| DECR1 | -1.51 | 3.13E-11 |
| LILRB5 | -1.51 | 1.16E-18 |
| ADK | -1.51 | 7.98E-08 |
| ANXA5 | -1.51 | 9.03E-09 |
| MBNL2 | -1.51 | 8.60E-07 |
| PPARA | -1.51 | 9.78E-12 |
| ZSWIM7 | -1.51 | 1.99E-04 |
| ABLIM3 | -1.51 | 9.83E-07 |
| ELOVL5 | -1.51 | 3.17E-04 |
| UST | -1.51 | 1.22E-08 |
| GRK5 | -1.51 | 3.77E-07 |
| CPVL | -1.51 | 3.09E-04 |
| C22orf39 | -1.51 | 9.63E-20 |
| CPS1 | -1.52 | 9.51E-12 |
| CLIC2 | -1.52 | 1.58E-11 |
| TNIP1 | -1.52 | 1.13E-11 |
| AMOTL2 | -1.52 | 1.66E-06 |
| WDFY3-AS2 | -1.52 | 9.88E-20 |
| HIKESHI | -1.52 | 3.73E-08 |
| SDHB | -1.52 | 3.45E-06 |
| DPH3 | -1.52 | 2.29E-06 |
| RCAN2 | -1.52 | 7.82E-08 |
| GPX4 | -1.53 | 5.36E-10 |
| EPS8 | -1.53 | 1.45E-05 |
| AK3 | -1.53 | 4.72E-10 |
| ADAMTS18 | -1.53 | 1.06E-08 |
| PKD2 | -1.53 | 1.89E-07 |
| FBLN1 | -1.53 | 1.04E-03 |
| MCCC1 | -1.53 | 4.57E-12 |
| RDH10 | -1.53 | 3.38E-03 |
| PGAP4 | -1.54 | 2.82E-21 |
| CDC37L1 | -1.54 | 9.98E-10 |
| NDUFS4 | -1.54 | 3.38E-12 |
| CYBRD1 | -1.54 | 1.92E-03 |
| DMRT2 | -1.54 | 2.89E-18 |
| SPON1 | -1.54 | 1.67E-03 |
| WDR86 | -1.54 | 6.23E-10 |
| NPR2 | -1.54 | 1.99E-21 |
| GCLC | -1.54 | 3.00E-09 |
| MSRA | -1.54 | 5.45E-09 |
| RCBTB2 | -1.54 | 6.90E-11 |
| MAP7D1 | -1.54 | 2.53E-13 |
| LPAR1 | -1.55 | 6.58E-09 |
| NR4A3 | -1.55 | 1.42E-07 |
| UBE2E2 | -1.55 | 1.42E-07 |
| CLDN5 | -1.55 | 2.67E-09 |
| ACOT13 | -1.55 | 8.20E-08 |
| SNX10 | -1.55 | 1.27E-05 |
| C1QA | -1.55 | 2.92E-03 |
| ALAD | -1.55 | 1.92E-09 |
| HACD1 | -1.55 | 2.53E-04 |
| STARD13 | -1.55 | 2.17E-06 |
| KLF11 | -1.56 | 4.04E-07 |
| GIMAP8 | -1.56 | 2.24E-05 |
| ROCK2 | -1.56 | 1.72E-07 |
| HOGA1 | -1.56 | 5.53E-18 |
| MOB3B | -1.56 | 1.37E-07 |
| ARID5B | -1.56 | 2.09E-07 |
| ZNF25 | -1.56 | 1.51E-07 |
| VWF | -1.56 | 4.03E-04 |
| DAB2 | -1.56 | 5.24E-06 |
| EIF2B3 | -1.56 | 1.55E-09 |
| MLXIPL | -1.56 | 1.37E-21 |
| CERT1 | -1.57 | 2.10E-13 |
| PGD | -1.57 | 3.43E-06 |
| ZNF677 | -1.57 | 2.91E-04 |
| ALDH5A1 | -1.57 | 4.62E-07 |
| SHE | -1.57 | 1.82E-08 |
| GDF10 | -1.57 | 1.13E-18 |
| TMLHE | -1.57 | 2.83E-08 |
| KATNAL1 | -1.57 | 3.92E-11 |
| FAM107A | -1.58 | 4.03E-07 |
| GAS1 | -1.58 | 3.63E-04 |
| DRAM2 | -1.58 | 1.90E-08 |
| GALNT12 | -1.58 | 8.60E-06 |
| CDH5 | -1.58 | 7.74E-09 |
| RND3 | -1.58 | 6.46E-05 |
| CRY2 | -1.58 | 7.39E-18 |
| FTCDNL1 | -1.58 | 4.85E-16 |
| SPATA9 | -1.59 | 2.35E-15 |
| JAM2 | -1.59 | 1.34E-05 |
| PRKD1 | -1.59 | 6.05E-11 |
| PLIN5 | -1.59 | 3.93E-05 |
| THYN1 | -1.59 | 9.72E-08 |
| BMPER | -1.59 | 6.43E-15 |
| STAT5B | -1.60 | 3.80E-13 |
| TMEM64 | -1.60 | 3.38E-04 |
| MTMR10 | -1.60 | 1.50E-11 |
| MTHFD1 | -1.60 | 2.61E-14 |
| NFU1 | -1.60 | 1.03E-13 |
| ARHGAP21 | -1.60 | 2.17E-11 |
| BST1 | -1.60 | 1.02E-13 |
| DCUN1D3 | -1.60 | 4.08E-13 |
| ADGRA2 | -1.60 | 9.11E-11 |
| FYN | -1.61 | 4.68E-08 |
| LMBRD1 | -1.61 | 4.79E-06 |
| THSD7B | -1.61 | 2.93E-15 |
| PITPNA-AS1 | -1.61 | 2.95E-08 |
| ABCA5 | -1.61 | 1.20E-08 |
| VEGFC | -1.61 | 3.32E-08 |
| MXRA7 | -1.61 | 4.04E-08 |
| SYNM | -1.61 | 8.72E-03 |
| CITED2 | -1.61 | 1.15E-06 |
| ALDH3A2 | -1.61 | 5.26E-06 |
| GLRB | -1.61 | 8.69E-04 |
| TMEM44-AS1 | -1.62 | 8.66E-11 |
| C11orf74 | -1.62 | 1.01E-12 |
| PPP2R5A | -1.62 | 1.56E-09 |
| OSBPL1A | -1.62 | 2.22E-06 |
| PTX3 | -1.62 | 1.66E-03 |
| LYRM1 | -1.62 | 4.11E-13 |
| DTX1 | -1.62 | 3.79E-15 |
| NT5E | -1.62 | 1.03E-07 |
| RNF125 | -1.62 | 9.81E-11 |
| FAM76A | -1.62 | 4.95E-10 |
| QDPR | -1.63 | 1.01E-03 |
| ACADS | -1.63 | 1.67E-19 |
| HACL1 | -1.63 | 1.69E-09 |
| TMEM273 | -1.63 | 4.05E-07 |
| LY96 | -1.63 | 3.92E-06 |
| RMDN2 | -1.63 | 8.14E-15 |
| RORB | -1.63 | 3.53E-09 |
| CKB | -1.63 | 1.73E-06 |
| NID1 | -1.63 | 1.26E-04 |
| SH3BGRL2 | -1.64 | 9.54E-09 |
| MPC1 | -1.64 | 9.78E-17 |
| GALNT16 | -1.64 | 1.36E-02 |
| CCM2L | -1.64 | 7.58E-10 |
| C2orf76 | -1.64 | 1.02E-08 |
| FAM200B | -1.64 | 8.39E-14 |
| PDE4DIP | -1.64 | 1.40E-06 |
| FIGN | -1.64 | 1.65E-04 |
| SETD7 | -1.64 | 3.90E-10 |
| TEK | -1.64 | 3.82E-11 |
| PRDX6 | -1.64 | 3.53E-13 |
| HSPB6 | -1.64 | 2.18E-24 |
| PRKD3 | -1.64 | 9.24E-07 |
| LGALS1 | -1.65 | 4.40E-07 |
| HOXA10 | -1.65 | 3.88E-12 |
| COLEC12 | -1.65 | 2.07E-04 |
| CRLS1 | -1.65 | 1.81E-10 |
| ADAMTS9 | -1.65 | 5.63E-06 |
| NPR1 | -1.65 | 1.31E-15 |
| CYYR1 | -1.65 | 1.62E-05 |
| EEPD1 | -1.66 | 6.59E-16 |
| GSPT2 | -1.66 | 6.24E-04 |
| MITF | -1.66 | 1.23E-09 |
| FAM228B | -1.66 | 2.40E-12 |
| ADD3 | -1.66 | 4.37E-06 |
| SRPX2 | -1.66 | 3.34E-05 |
| ICAM2 | -1.66 | 2.30E-06 |
| MKX | -1.66 | 3.33E-04 |
| C11orf96 | -1.66 | 1.07E-05 |
| ANKRD40 | -1.66 | 6.45E-11 |
| NAP1L5 | -1.66 | 4.63E-06 |
| GPR183 | -1.67 | 1.87E-05 |
| CCND2 | -1.67 | 5.27E-06 |
| FAT4 | -1.67 | 5.82E-13 |
| TK2 | -1.67 | 4.58E-14 |
| NAT8L | -1.67 | 8.18E-17 |
| KANK2 | -1.67 | 1.49E-07 |
| FBXO17 | -1.67 | 4.22E-07 |
| KL | -1.67 | 1.94E-19 |
| SOD2 | -1.67 | 1.59E-05 |
| LOC100507557 | -1.67 | 8.39E-12 |
| TNS2 | -1.67 | 5.98E-11 |
| PDE1A | -1.67 | 5.92E-12 |
| S1PR1 | -1.67 | 1.34E-19 |
| LIX1L | -1.68 | 5.97E-10 |
| FSTL1 | -1.68 | 4.71E-05 |
| DNAJB4 | -1.68 | 7.76E-09 |
| CLCN4 | -1.68 | 7.76E-08 |
| APIP | -1.68 | 4.12E-09 |
| MARCO | -1.68 | 5.54E-04 |
| DPYD | -1.68 | 6.90E-06 |
| EMCN | -1.69 | 1.49E-08 |
| GKAP1 | -1.70 | 1.25E-07 |
| NLRP3 | -1.70 | 3.86E-12 |
| DDIT4L | -1.70 | 1.14E-09 |
| DENND5B | -1.71 | 1.93E-08 |
| OSTM1 | -1.71 | 1.16E-12 |
| TMEM256 | -1.71 | 3.22E-07 |
| PDK2 | -1.71 | 3.67E-13 |
| NNT | -1.71 | 1.65E-07 |
| CALM1 | -1.71 | 9.51E-17 |
| SOCS3 | -1.71 | 2.21E-05 |
| DPP4 | -1.71 | 5.89E-11 |
| SEPTIN11 | -1.71 | 1.60E-10 |
| ZNF423 | -1.71 | 6.47E-07 |
| S100A4 | -1.72 | 2.78E-06 |
| SLC25A18 | -1.73 | 4.07E-09 |
| EPB42 | -1.73 | 1.76E-24 |
| FLI1 | -1.73 | 2.06E-05 |
| STX7 | -1.73 | 6.22E-08 |
| PODN | -1.73 | 2.28E-08 |
| PLD1 | -1.73 | 2.53E-09 |
| RGL1 | -1.74 | 6.98E-09 |
| FOXN2 | -1.74 | 6.43E-10 |
| RGS22 | -1.74 | 5.21E-05 |
| IDH3A | -1.74 | 1.80E-09 |
| SNX1 | -1.74 | 7.04E-09 |
| MAOB | -1.74 | 3.69E-03 |
| MYEOV | -1.74 | 2.58E-06 |
| CHCHD10 | -1.75 | 5.45E-10 |
| CNTN3 | -1.75 | 6.26E-07 |
| PARVA | -1.75 | 2.52E-08 |
| SPRY1 | -1.75 | 5.75E-06 |
| PHYH | -1.75 | 6.41E-08 |
| LACTB2 | -1.75 | 5.53E-08 |
| SASH1 | -1.75 | 1.36E-04 |
| DIS3L | -1.76 | 1.15E-14 |
| IL6 | -1.76 | 8.31E-10 |
| ALDH6A1 | -1.76 | 1.09E-05 |
| NTM | -1.76 | 1.81E-05 |
| PAMR1 | -1.76 | 2.33E-10 |
| SNX21 | -1.76 | 1.34E-06 |
| TLR3 | -1.76 | 2.47E-10 |
| POLR3GL | -1.76 | 1.84E-12 |
| PRICKLE2 | -1.77 | 1.77E-07 |
| EMX2 | -1.77 | 3.09E-16 |
| ANKRD29 | -1.77 | 1.54E-07 |
| CORO2B | -1.77 | 1.14E-21 |
| SPART | -1.77 | 2.16E-08 |
| ECSCR | -1.77 | 9.72E-08 |
| LIPE-AS1 | -1.77 | 9.69E-23 |
| HAS2 | -1.78 | 4.16E-09 |
| ADGRL2 | -1.78 | 1.93E-08 |
| B4GALT6 | -1.78 | 4.82E-13 |
| KANSL1-AS1 | -1.78 | 2.88E-05 |
| RGS2 | -1.78 | 2.56E-05 |
| NNAT | -1.78 | 7.19E-16 |
| PJA1 | -1.78 | 5.20E-11 |
| HOXA5 | -1.78 | 1.72E-07 |
| PLAGL1 | -1.78 | 5.66E-07 |
| LINC00888 | -1.78 | 1.57E-07 |
| EPHX1 | -1.78 | 4.51E-07 |
| C7 | -1.78 | 5.14E-05 |
| ABCD2 | -1.79 | 1.23E-20 |
| EGR1 | -1.79 | 4.47E-05 |
| MYCT1 | -1.79 | 1.02E-19 |
| SGCB | -1.80 | 4.69E-11 |
| GULP1 | -1.80 | 6.00E-06 |
| TSPAN3 | -1.80 | 1.63E-09 |
| PDE2A | -1.80 | 2.40E-20 |
| CAVIN1 | -1.80 | 2.57E-08 |
| MARCHF2 | -1.80 | 5.73E-14 |
| CCNDBP1 | -1.80 | 1.29E-06 |
| CYSTM1 | -1.81 | 9.54E-13 |
| SDHD | -1.81 | 3.26E-13 |
| ACYP2 | -1.81 | 4.36E-11 |
| SERINC1 | -1.81 | 3.12E-09 |
| FGF1 | -1.81 | 4.46E-09 |
| CARD6 | -1.81 | 6.28E-13 |
| PBLD | -1.81 | 1.08E-09 |
| FREM1 | -1.82 | 5.03E-20 |
| LOC729970 | -1.82 | 4.94E-16 |
| SPATA7 | -1.82 | 2.28E-10 |
| SKAP2 | -1.83 | 8.08E-07 |
| ADGRF5 | -1.83 | 2.14E-08 |
| PTPRG | -1.83 | 2.83E-07 |
| FCGR2B | -1.83 | 1.72E-06 |
| ID4 | -1.83 | 4.26E-03 |
| C8orf88 | -1.83 | 4.48E-10 |
| TRIM52-AS1 | -1.84 | 1.98E-17 |
| ADGRD1 | -1.84 | 1.24E-22 |
| ARHGEF4 | -1.84 | 2.14E-08 |
| ZCCHC24 | -1.84 | 3.73E-08 |
| SLC25A20 | -1.84 | 1.22E-14 |
| INMT | -1.84 | 7.96E-15 |
| OSR2 | -1.84 | 4.65E-06 |
| DCTN6 | -1.84 | 1.79E-13 |
| ACKR1 | -1.84 | 5.27E-04 |
| ADSS1 | -1.85 | 3.12E-05 |
| EPB41L4B | -1.85 | 6.14E-06 |
| RUNX1T1 | -1.85 | 2.52E-06 |
| DENND2A | -1.85 | 5.57E-24 |
| ABHD15 | -1.85 | 9.29E-10 |
| FADS3 | -1.85 | 2.35E-11 |
| KLF2 | -1.85 | 3.37E-09 |
| FGL2 | -1.85 | 1.80E-06 |
| GTDC1 | -1.86 | 2.82E-18 |
| MYH1 | -1.86 | 4.24E-07 |
| FILIP1 | -1.86 | 1.27E-15 |
| ANG | -1.86 | 5.94E-06 |
| ABHD6 | -1.86 | 1.95E-12 |
| CCDC82 | -1.86 | 2.03E-12 |
| TSHZ2 | -1.87 | 5.38E-08 |
| STRADB | -1.87 | 1.68E-17 |
| NR3C1 | -1.87 | 1.64E-11 |
| CCNH | -1.87 | 4.56E-16 |
| PDP2 | -1.87 | 1.55E-14 |
| SNTB2 | -1.87 | 3.02E-10 |
| PROS1 | -1.88 | 5.78E-06 |
| EMILIN2 | -1.88 | 1.48E-06 |
| DSE | -1.88 | 2.10E-06 |
| HIGD1B | -1.88 | 1.82E-15 |
| SGK2 | -1.88 | 1.33E-24 |
| ARHGAP6 | -1.88 | 2.93E-09 |
| FZD5 | -1.89 | 1.57E-07 |
| PYGM | -1.89 | 1.19E-16 |
| CCDC50 | -1.89 | 2.11E-13 |
| FGF7P3 | -1.89 | 8.84E-17 |
| AK4 | -1.89 | 9.12E-05 |
| MMRN2 | -1.89 | 7.05E-09 |
| PLPP3 | -1.89 | 1.17E-06 |
| SELENOP | -1.89 | 5.07E-05 |
| RDH5 | -1.89 | 2.55E-16 |
| L3HYPDH | -1.90 | 3.75E-19 |
| FAXDC2 | -1.90 | 5.78E-13 |
| COQ8A | -1.90 | 1.03E-11 |
| EPAS1 | -1.90 | 1.27E-10 |
| SYN2 | -1.90 | 1.55E-19 |
| PENK | -1.90 | 1.33E-10 |
| CREB5 | -1.90 | 1.92E-19 |
| MEIS2 | -1.91 | 2.57E-07 |
| LRP1 | -1.91 | 7.30E-07 |
| DLD | -1.91 | 2.24E-10 |
| SLC25A33 | -1.91 | 2.18E-09 |
| WLS | -1.91 | 2.08E-05 |
| PTPRM | -1.91 | 1.69E-09 |
| DHDDS | -1.91 | 1.17E-15 |
| CTIF | -1.91 | 1.39E-09 |
| PTGDS | -1.91 | 6.44E-06 |
| ADGRL4 | -1.92 | 6.08E-10 |
| TMEM47 | -1.92 | 1.48E-05 |
| ARHGEF40 | -1.92 | 5.73E-09 |
| TFPI | -1.92 | 1.45E-04 |
| CACHD1 | -1.92 | 8.82E-07 |
| JHY | -1.92 | 1.25E-21 |
| HOXA7 | -1.92 | 3.26E-11 |
| CYP2U1 | -1.93 | 3.06E-13 |
| SCN4B | -1.93 | 3.80E-13 |
| F3 | -1.93 | 6.03E-06 |
| SOX7 | -1.93 | 6.50E-10 |
| EPM2A | -1.93 | 1.61E-15 |
| CD163 | -1.93 | 5.29E-04 |
| FAM13C | -1.93 | 1.96E-09 |
| CYP26B1 | -1.93 | 3.45E-10 |
| MS4A4A | -1.94 | 2.36E-12 |
| NEK7 | -1.94 | 2.99E-15 |
| AKAP7 | -1.94 | 1.14E-11 |
| PDGFRA | -1.94 | 1.51E-05 |
| PLEKHM3 | -1.94 | 2.93E-15 |
| VAMP5 | -1.94 | 1.30E-06 |
| MBNL1-AS1 | -1.94 | 1.34E-10 |
| MSC-AS1 | -1.95 | 1.64E-12 |
| PPP2R3C | -1.95 | 4.57E-19 |
| THRB | -1.95 | 1.62E-10 |
| ZC3H12C | -1.95 | 7.26E-10 |
| LRRC34 | -1.95 | 3.07E-15 |
| ANO6 | -1.96 | 1.95E-15 |
| PLN | -1.96 | 1.90E-07 |
| TENM3 | -1.96 | 1.47E-12 |
| AVPR1A | -1.96 | 1.47E-15 |
| EPB41L3 | -1.96 | 1.13E-06 |
| NPY5R | -1.96 | 2.22E-15 |
| GMFG | -1.96 | 3.01E-07 |
| C2CD2 | -1.96 | 3.78E-08 |
| NPY1R | -1.96 | 7.03E-03 |
| ACADM | -1.96 | 1.95E-15 |
| SH3GLB1 | -1.96 | 6.62E-13 |
| EHBP1 | -1.96 | 1.10E-11 |
| CALCRL | -1.97 | 5.24E-16 |
| ARHGEF6 | -1.97 | 1.70E-08 |
| MDH1 | -1.97 | 2.14E-15 |
| BNIP3L | -1.97 | 1.55E-12 |
| TMOD1 | -1.97 | 7.25E-20 |
| BICC1 | -1.97 | 1.73E-09 |
| PKD1L2 | -1.97 | 3.84E-16 |
| UCHL1 | -1.97 | 1.40E-05 |
| MIR100HG | -1.97 | 5.49E-07 |
| TM7SF2 | -1.97 | 5.01E-06 |
| MPEG1 | -1.98 | 3.11E-07 |
| SPX | -1.98 | 7.08E-20 |
| RASGRF2 | -1.98 | 2.50E-16 |
| MAF | -1.98 | 6.51E-09 |
| PTPRB | -1.98 | 8.69E-09 |
| MBNL3 | -1.98 | 6.11E-07 |
| TUBB6 | -1.98 | 7.65E-09 |
| CASP17P | -1.98 | 3.92E-12 |
| PEX19 | -1.99 | 3.27E-10 |
| GPER1 | -1.99 | 1.82E-08 |
| MAP7D3 | -1.99 | 2.22E-19 |
| MAP1LC3C | -1.99 | 2.88E-23 |
| SCD | -1.99 | 4.59E-05 |
| C6 | -1.99 | 3.61E-18 |
| TXNIP | -2.00 | 1.38E-07 |
| FBLN2 | -2.00 | 3.96E-08 |
| SIM1 | -2.00 | 6.92E-18 |
| PDE3A | -2.00 | 3.17E-18 |
| SAMD8 | -2.00 | 4.08E-14 |
| TACC1 | -2.00 | 9.67E-10 |
| FAM92A | -2.00 | 1.14E-15 |
| LINC00667 | -2.01 | 1.45E-12 |
| MAN1A1 | -2.01 | 1.26E-06 |
| CCL8 | -2.01 | 6.59E-05 |
| TNFAIP8 | -2.01 | 1.13E-07 |
| GABARAPL1 | -2.01 | 5.48E-15 |
| FEZ1 | -2.01 | 1.37E-12 |
| FKBP5 | -2.01 | 6.49E-07 |
| CLEC2B | -2.01 | 1.57E-09 |
| ABHD5 | -2.01 | 2.37E-17 |
| PIR | -2.01 | 1.27E-07 |
| KLHL5 | -2.02 | 1.08E-09 |
| LIFR | -2.02 | 2.23E-07 |
| PEMT | -2.02 | 5.84E-10 |
| C19orf12 | -2.02 | 1.30E-18 |
| HLF | -2.02 | 3.72E-16 |
| CCL2 | -2.02 | 5.83E-08 |
| APOLD1 | -2.02 | 5.16E-10 |
| PDZRN3 | -2.02 | 4.50E-07 |
| PLIN2 | -2.02 | 1.83E-08 |
| TFPI2 | -2.03 | 2.45E-03 |
| NAV3 | -2.03 | 5.19E-05 |
| ETFB | -2.03 | 1.49E-10 |
| CTTNBP2 | -2.03 | 1.98E-15 |
| LDHD | -2.03 | 4.94E-19 |
| SGCE | -2.03 | 2.30E-05 |
| PCCA | -2.04 | 1.37E-09 |
| CDC14B | -2.04 | 5.82E-11 |
| SLIT3 | -2.04 | 1.14E-11 |
| MYL9 | -2.04 | 8.70E-08 |
| EMP1 | -2.04 | 5.05E-06 |
| HP | -2.05 | 1.41E-07 |
| BCHE | -2.05 | 1.06E-06 |
| RASA3 | -2.05 | 2.44E-16 |
| KANK3 | -2.05 | 4.87E-14 |
| SH3KBP1 | -2.05 | 2.25E-12 |
| ETFA | -2.05 | 1.39E-14 |
| TLR4 | -2.05 | 3.17E-16 |
| FMO3 | -2.07 | 4.38E-12 |
| RNF157 | -2.07 | 7.31E-16 |
| PTGER4 | -2.07 | 1.81E-06 |
| HADH | -2.07 | 1.03E-14 |
| KLF15 | -2.07 | 2.76E-18 |
| ATP8B4 | -2.08 | 9.24E-17 |
| DAAM2 | -2.08 | 1.15E-12 |
| NRP1 | -2.08 | 9.32E-07 |
| TCEAL7 | -2.08 | 2.22E-15 |
| STS | -2.08 | 6.98E-08 |
| SDHAF4 | -2.08 | 3.13E-14 |
| CYP4B1 | -2.08 | 5.17E-03 |
| PCSK5 | -2.08 | 7.17E-08 |
| ATP23 | -2.09 | 3.99E-14 |
| NEXN | -2.09 | 2.16E-08 |
| ACSL4 | -2.09 | 4.75E-09 |
| CLSTN2 | -2.10 | 6.84E-03 |
| NR3C2 | -2.10 | 1.17E-09 |
| VTI1B | -2.10 | 8.07E-21 |
| ETFDH | -2.10 | 3.20E-14 |
| PHLDB2 | -2.10 | 4.14E-08 |
| SMIM20 | -2.11 | 1.53E-08 |
| PARD3B | -2.11 | 2.55E-24 |
| RRAS2 | -2.11 | 1.32E-07 |
| ATF3 | -2.11 | 8.00E-05 |
| RRAS | -2.12 | 8.39E-12 |
| TTC7B | -2.12 | 9.30E-20 |
| BNIP2 | -2.12 | 1.13E-10 |
| RGN | -2.12 | 5.67E-17 |
| NOVA1 | -2.13 | 9.31E-06 |
| CXCL14 | -2.13 | 1.02E-02 |
| MEOX1 | -2.13 | 5.51E-08 |
| ADAMTS1 | -2.13 | 3.18E-05 |
| THBS4 | -2.13 | 3.10E-04 |
| PRXL2A | -2.13 | 4.68E-10 |
| FOXN3 | -2.14 | 1.16E-13 |
| STAT5A | -2.14 | 2.64E-16 |
| ACAT1 | -2.14 | 1.16E-13 |
| ANKRD33B | -2.14 | 5.82E-14 |
| GSTM5 | -2.14 | 1.01E-09 |
| PTPN21 | -2.15 | 9.51E-12 |
| CD248 | -2.16 | 6.75E-10 |
| MOCS1 | -2.16 | 3.10E-22 |
| FAH | -2.16 | 2.37E-08 |
| RIDA | -2.16 | 1.26E-08 |
| F8 | -2.17 | 1.27E-15 |
| STON1 | -2.17 | 3.74E-10 |
| SPRY2 | -2.17 | 9.84E-07 |
| PALM | -2.17 | 2.06E-26 |
| HSD17B11 | -2.18 | 2.07E-09 |
| HOXD-AS2 | -2.18 | 1.60E-14 |
| HRCT1 | -2.18 | 1.99E-09 |
| PLPP1 | -2.18 | 3.15E-11 |
| ENPEP | -2.18 | 1.26E-15 |
| OMD | -2.19 | 3.06E-12 |
| LMOD1 | -2.19 | 6.25E-12 |
| ATOH8 | -2.19 | 3.76E-27 |
| CSTA | -2.21 | 7.04E-05 |
| SOBP | -2.21 | 2.72E-08 |
| CCDC178 | -2.21 | 3.12E-33 |
| TPPP3 | -2.21 | 3.04E-11 |
| ACSS2 | -2.22 | 8.65E-18 |
| SYNPO | -2.22 | 1.98E-13 |
| EPB41L2 | -2.22 | 1.39E-12 |
| PTGS2 | -2.23 | 1.68E-08 |
| ABCA9 | -2.23 | 6.88E-17 |
| ZNF106 | -2.23 | 1.18E-12 |
| TMEM140 | -2.23 | 1.67E-13 |
| ITGA1 | -2.23 | 1.73E-14 |
| NR1H3 | -2.23 | 3.16E-09 |
| JAZF1 | -2.23 | 5.63E-18 |
| CRYBG3 | -2.23 | 1.15E-12 |
| BOK | -2.23 | 6.38E-07 |
| PLTP | -2.23 | 6.30E-07 |
| TLN2 | -2.23 | 1.20E-21 |
| GALNT15 | -2.23 | 2.74E-15 |
| CCDC80 | -2.24 | 1.04E-05 |
| PGM5 | -2.24 | 1.32E-20 |
| LAMA2 | -2.24 | 6.64E-12 |
| ITSN1 | -2.24 | 1.00E-09 |
| HSPB8 | -2.25 | 4.73E-05 |
| PRRT3-AS1 | -2.25 | 2.58E-12 |
| SLIT2 | -2.25 | 1.45E-05 |
| ARHGAP24 | -2.25 | 1.16E-20 |
| LAIR1 | -2.25 | 6.71E-10 |
| METTL7A | -2.25 | 4.88E-10 |
| RHOQ | -2.26 | 5.94E-17 |
| SPTBN1 | -2.26 | 5.82E-14 |
| SDC2 | -2.26 | 1.46E-06 |
| FILIP1L | -2.26 | 3.52E-10 |
| NECTIN3 | -2.26 | 9.07E-30 |
| IL33 | -2.27 | 3.33E-11 |
| KCNE3 | -2.27 | 6.95E-14 |
| SCRN2 | -2.27 | 2.21E-14 |
| CUTC | -2.27 | 1.97E-18 |
| VIM | -2.27 | 1.03E-11 |
| EIF4E3 | -2.28 | 9.92E-11 |
| GBE1 | -2.29 | 1.45E-18 |
| LINC02716 | -2.29 | 4.53E-29 |
| RFTN1 | -2.29 | 3.07E-10 |
| PLCL2 | -2.30 | 3.82E-15 |
| MAP3K20 | -2.30 | 2.70E-14 |
| ZFP36 | -2.30 | 6.55E-13 |
| ANO3 | -2.30 | 5.78E-13 |
| KAT2B | -2.30 | 3.20E-11 |
| MSRB3 | -2.31 | 2.73E-07 |
| HINT3 | -2.31 | 5.53E-11 |
| NFIL3 | -2.31 | 2.86E-09 |
| AADAC | -2.31 | 2.07E-19 |
| DCN | -2.32 | 7.52E-06 |
| APCDD1 | -2.32 | 5.22E-10 |
| SLC66A1L | -2.32 | 1.23E-30 |
| KCNJ8 | -2.32 | 7.18E-10 |
| EGFR | -2.33 | 1.25E-04 |
| DIXDC1 | -2.33 | 5.15E-19 |
| RETSAT | -2.33 | 1.27E-13 |
| PFKFB3 | -2.33 | 7.80E-13 |
| SSPN | -2.34 | 1.98E-13 |
| RHOXF1 | -2.34 | 1.41E-23 |
| HPGDS | -2.34 | 2.26E-16 |
| FOXO1 | -2.34 | 1.47E-15 |
| APBB1IP | -2.35 | 5.67E-11 |
| RBMS3 | -2.35 | 1.82E-06 |
| GSN | -2.35 | 3.38E-10 |
| TGFBR3 | -2.35 | 2.65E-05 |
| IGSF10 | -2.36 | 3.04E-32 |
| KANK1 | -2.36 | 1.32E-08 |
| GYPC | -2.37 | 1.34E-19 |
| ZDHHC2 | -2.37 | 3.94E-08 |
| TMEM135 | -2.37 | 1.06E-14 |
| BIN1 | -2.37 | 8.61E-14 |
| LDHB | -2.37 | 8.51E-08 |
| CAMK1 | -2.37 | 1.65E-18 |
| MGST1 | -2.38 | 7.93E-06 |
| TWIST1 | -2.38 | 1.23E-07 |
| EHHADH | -2.38 | 8.76E-19 |
| CAT | -2.39 | 3.71E-19 |
| NIPSNAP3B | -2.39 | 1.10E-26 |
| AIFM2 | -2.39 | 2.80E-22 |
| ACKR3 | -2.40 | 1.20E-10 |
| SFRP1 | -2.40 | 5.11E-03 |
| PDGFRL | -2.40 | 2.22E-07 |
| MRGPRF | -2.41 | 2.50E-12 |
| ECRG4 | -2.41 | 1.71E-06 |
| NATD1 | -2.42 | 6.07E-15 |
| MESP1 | -2.42 | 3.19E-09 |
| FMOD | -2.42 | 3.49E-07 |
| MAPK10 | -2.43 | 1.48E-14 |
| VEGFB | -2.43 | 1.03E-16 |
| CXCL2 | -2.43 | 1.91E-08 |
| FGF2 | -2.43 | 1.11E-18 |
| PC | -2.43 | 2.21E-14 |
| VIT | -2.44 | 2.26E-16 |
| OLFML1 | -2.44 | 1.95E-15 |
| RRAGD | -2.44 | 2.30E-11 |
| EHD2 | -2.44 | 7.25E-13 |
| SIK2 | -2.44 | 1.28E-14 |
| ELMOD3 | -2.44 | 2.40E-27 |
| GPAT3 | -2.45 | 3.63E-08 |
| MSX1 | -2.45 | 4.61E-12 |
| MYOC | -2.45 | 4.27E-18 |
| GPATCH11 | -2.46 | 2.38E-26 |
| ZEB1 | -2.47 | 1.16E-11 |
| VSIG4 | -2.48 | 5.90E-08 |
| ABI3BP | -2.48 | 3.90E-06 |
| KCNAB1 | -2.48 | 3.47E-16 |
| TSLP | -2.48 | 1.47E-24 |
| CELF2 | -2.48 | 1.72E-10 |
| RHOBTB3 | -2.48 | 7.67E-07 |
| FOXP2 | -2.49 | 5.27E-22 |
| SEL1L2 | -2.49 | 1.61E-15 |
| SUCLA2 | -2.50 | 7.67E-18 |
| HSDL2 | -2.50 | 7.78E-16 |
| GLDN | -2.50 | 1.21E-08 |
| GDPD5 | -2.50 | 2.55E-11 |
| APOL6 | -2.51 | 3.21E-15 |
| DIPK1A | -2.51 | 1.00E-13 |
| TGFBR2 | -2.51 | 8.76E-09 |
| LAMA4 | -2.51 | 9.56E-11 |
| HNMT | -2.51 | 1.88E-15 |
| SLC9A9 | -2.51 | 2.13E-24 |
| LHFPL6 | -2.52 | 6.37E-10 |
| ZFPM2 | -2.52 | 4.35E-18 |
| DNASE1L3 | -2.52 | 2.79E-16 |
| LDB2 | -2.52 | 6.61E-15 |
| ITGB1BP1 | -2.52 | 3.17E-22 |
| PGM1 | -2.52 | 9.15E-12 |
| PDGFD | -2.53 | 3.97E-07 |
| DUSP1 | -2.53 | 4.08E-09 |
| CA4 | -2.53 | 2.03E-26 |
| SLC1A3 | -2.53 | 1.08E-09 |
| CRIM1-DT | -2.54 | 3.98E-08 |
| DHRS3 | -2.55 | 3.92E-12 |
| ANGPTL4 | -2.55 | 1.26E-10 |
| SMIM3 | -2.56 | 1.49E-19 |
| CTNNAL1 | -2.56 | 2.75E-16 |
| ADHFE1 | -2.56 | 5.90E-21 |
| OXCT1 | -2.58 | 2.94E-12 |
| PIP4P2 | -2.58 | 4.03E-18 |
| RASD1 | -2.58 | 1.89E-11 |
| TNS1 | -2.59 | 1.33E-09 |
| MYOM1 | -2.59 | 4.04E-27 |
| COPZ2 | -2.59 | 3.40E-15 |
| CEP112 | -2.60 | 6.96E-27 |
| ACKR4 | -2.60 | 1.66E-18 |
| MDFIC | -2.60 | 1.97E-14 |
| ANKRD35 | -2.60 | 1.03E-16 |
| FAM162B | -2.61 | 2.09E-22 |
| ANGPTL8 | -2.62 | 5.53E-23 |
| ITM2A | -2.62 | 1.92E-06 |
| LRP1B | -2.63 | 1.40E-11 |
| DMGDH | -2.64 | 5.38E-31 |
| ABCC6 | -2.64 | 5.95E-10 |
| MCAM | -2.64 | 4.08E-13 |
| ANTXR2 | -2.65 | 1.67E-12 |
| ANGPTL2 | -2.65 | 4.98E-16 |
| GPC6 | -2.66 | 3.80E-09 |
| C8orf34 | -2.67 | 3.01E-28 |
| EGFL6 | -2.67 | 5.17E-07 |
| SLC7A10 | -2.68 | 3.10E-30 |
| RNF180 | -2.68 | 9.45E-20 |
| TLCD2 | -2.69 | 2.02E-13 |
| TRHDE | -2.69 | 3.98E-29 |
| DSEL | -2.70 | 4.86E-12 |
| TMTC1 | -2.71 | 1.45E-07 |
| DIAPH2 | -2.72 | 3.86E-17 |
| AGPAT2 | -2.72 | 4.96E-26 |
| WNT11 | -2.72 | 2.65E-17 |
| SLC29A4 | -2.73 | 1.02E-24 |
| AGTR1 | -2.74 | 6.58E-04 |
| COBLL1 | -2.75 | 9.52E-17 |
| SERPINF1 | -2.75 | 1.93E-11 |
| MEST | -2.76 | 3.89E-08 |
| SAMD4A | -2.76 | 2.39E-16 |
| APOB | -2.77 | 1.72E-28 |
| GPIHBP1 | -2.77 | 2.84E-29 |
| CKMT2 | -2.77 | 5.26E-23 |
| GNAL | -2.77 | 3.83E-18 |
| ZEB2 | -2.77 | 1.49E-16 |
| CD209 | -2.78 | 9.46E-26 |
| PCDH18 | -2.79 | 4.71E-11 |
| RNASE4 | -2.80 | 1.12E-11 |
| MAGI2-AS3 | -2.81 | 1.08E-23 |
| NUDT7 | -2.81 | 1.43E-19 |
| IRS2 | -2.81 | 3.11E-09 |
| FBLN5 | -2.81 | 2.49E-16 |
| TYRO3 | -2.81 | 1.63E-22 |
| PLSCR4 | -2.81 | 8.55E-14 |
| AFAP1L1 | -2.82 | 4.90E-18 |
| ADRB2 | -2.82 | 2.43E-17 |
| FGF14-AS2 | -2.82 | 1.68E-25 |
| EGFLAM | -2.83 | 2.99E-28 |
| TBX15 | -2.84 | 9.43E-29 |
| CLIP4 | -2.84 | 1.23E-10 |
| ACAA2 | -2.84 | 2.45E-16 |
| GGTA1P | -2.85 | 1.00E-15 |
| CA3 | -2.85 | 2.15E-10 |
| STEAP1 | -2.85 | 1.04E-09 |
| MYMX | -2.87 | 3.35E-24 |
| KANK4 | -2.87 | 1.10E-07 |
| UGP2 | -2.87 | 2.94E-18 |
| PTGIS | -2.87 | 9.09E-11 |
| EFEMP1 | -2.88 | 3.32E-06 |
| ECHDC1 | -2.88 | 1.59E-14 |
| MICU3 | -2.89 | 4.24E-20 |
| MGLL | -2.90 | 4.11E-12 |
| NDN | -2.91 | 5.00E-14 |
| ECHDC3 | -2.91 | 1.75E-12 |
| GRK3 | -2.91 | 2.73E-14 |
| SLC35G2 | -2.92 | 9.80E-23 |
| ACSS3 | -2.92 | 8.34E-10 |
| GNG11 | -2.92 | 1.73E-14 |
| RGCC | -2.93 | 8.84E-15 |
| CDKN2B | -2.94 | 1.46E-10 |
| PREX2 | -2.94 | 5.48E-18 |
| PRELP | -2.94 | 5.56E-12 |
| PLA2G2A | -2.95 | 5.01E-22 |
| TRHDE-AS1 | -2.95 | 3.93E-23 |
| RNASE1 | -2.96 | 1.29E-13 |
| ADAMTS5 | -2.96 | 3.05E-12 |
| PDE8B | -2.96 | 1.52E-12 |
| FZD4 | -2.97 | 4.60E-18 |
| PKDCC | -2.97 | 9.73E-20 |
| AVPI1 | -2.97 | 3.08E-21 |
| DLC1 | -2.97 | 1.13E-18 |
| NEGR1 | -2.98 | 3.27E-14 |
| LINC00968 | -2.98 | 1.94E-24 |
| ADM | -2.99 | 1.87E-10 |
| FOLR2 | -2.99 | 7.72E-16 |
| RBPMS-AS1 | -3.00 | 6.84E-27 |
| LOC102723493 | -3.00 | 1.82E-30 |
| RBPMS2 | -3.01 | 2.32E-25 |
| ABCA6 | -3.01 | 4.05E-26 |
| FABP5 | -3.02 | 2.44E-11 |
| EPDR1 | -3.03 | 3.51E-15 |
| MFAP4 | -3.03 | 4.56E-13 |
| TMEM220 | -3.03 | 5.13E-19 |
| GLYAT | -3.04 | 9.45E-31 |
| CDKN1C | -3.05 | 6.59E-10 |
| APOD | -3.06 | 2.16E-04 |
| FAM13A | -3.07 | 5.41E-14 |
| ALDH2 | -3.07 | 7.01E-13 |
| ACO1 | -3.07 | 1.24E-22 |
| FERMT2 | -3.08 | 4.84E-16 |
| P2RY12 | -3.08 | 2.05E-15 |
| CFH | -3.08 | 1.65E-10 |
| BMP2 | -3.09 | 1.84E-15 |
| CSN1S1 | -3.10 | 4.98E-18 |
| CFL2 | -3.10 | 1.91E-17 |
| VLDLR | -3.10 | 7.70E-10 |
| CEBPA | -3.12 | 3.97E-18 |
| HSD11B1 | -3.12 | 9.44E-16 |
| MAMDC2 | -3.13 | 4.11E-13 |
| EBF2 | -3.14 | 6.81E-25 |
| MATN2 | -3.15 | 1.64E-07 |
| CNRIP1 | -3.15 | 2.03E-19 |
| XG | -3.15 | 2.73E-18 |
| SOX5 | -3.15 | 1.41E-18 |
| MT1M | -3.16 | 2.05E-24 |
| BTNL9 | -3.16 | 5.67E-10 |
| MFAP5 | -3.16 | 1.01E-09 |
| FGFBP2 | -3.17 | 1.29E-19 |
| EDNRB | -3.17 | 2.34E-16 |
| CHL1 | -3.18 | 1.44E-13 |
| ANXA1 | -3.18 | 2.22E-10 |
| CXCL12 | -3.18 | 5.09E-09 |
| PLAAT3 | -3.20 | 1.35E-09 |
| SH3D19 | -3.20 | 5.73E-16 |
| RARRES2 | -3.20 | 9.22E-14 |
| VGLL3 | -3.20 | 3.89E-15 |
| PTGER3 | -3.22 | 3.25E-11 |
| CASQ2 | -3.22 | 2.01E-26 |
| ALDH1L1 | -3.23 | 1.28E-30 |
| KCNB1 | -3.23 | 7.19E-16 |
| MRAS | -3.23 | 6.65E-14 |
| KLF4 | -3.24 | 5.55E-13 |
| DMD | -3.24 | 7.30E-09 |
| LMO3 | -3.24 | 3.56E-11 |
| P2RY14 | -3.24 | 2.52E-14 |
| PID1 | -3.24 | 2.88E-19 |
| ANGPT1 | -3.24 | 2.60E-23 |
| RSPO3 | -3.25 | 6.14E-21 |
| PLAAT5 | -3.26 | 5.66E-19 |
| PLA2G4A | -3.27 | 2.12E-14 |
| NRN1 | -3.28 | 1.30E-20 |
| RBP7 | -3.29 | 4.76E-10 |
| PYGL | -3.29 | 3.49E-15 |
| CACNA2D1 | -3.30 | 6.89E-16 |
| VKORC1L1 | -3.31 | 4.48E-18 |
| FAM149A | -3.32 | 1.77E-20 |
| GPC3 | -3.32 | 5.62E-18 |
| ZNF436-AS1 | -3.32 | 3.60E-21 |
| ANK2 | -3.32 | 1.29E-22 |
| F13A1 | -3.35 | 1.20E-14 |
| DEPP1 | -3.35 | 1.32E-11 |
| HSPA12A | -3.35 | 1.35E-25 |
| MEOX2 | -3.37 | 5.05E-13 |
| LINC01279 | -3.38 | 2.11E-09 |
| AOX1 | -3.38 | 2.00E-24 |
| FMO2 | -3.39 | 5.51E-12 |
| SRPX | -3.39 | 3.22E-11 |
| ZBTB16 | -3.40 | 5.28E-13 |
| LARP6 | -3.40 | 7.13E-11 |
| SEMA3G | -3.41 | 1.80E-11 |
| FOS | -3.41 | 1.07E-08 |
| ADRA2A | -3.42 | 3.83E-12 |
| NMT2 | -3.44 | 7.19E-20 |
| COL6A6 | -3.45 | 3.51E-18 |
| EBF3 | -3.45 | 2.87E-26 |
| ENPP2 | -3.48 | 1.97E-08 |
| CAV1 | -3.48 | 6.89E-12 |
| TMEM170B | -3.48 | 4.42E-21 |
| SVEP1 | -3.49 | 8.60E-22 |
| HSPB2 | -3.49 | 6.65E-29 |
| WASF3 | -3.49 | 1.73E-21 |
| ACSL1 | -3.50 | 3.16E-15 |
| ACADL | -3.53 | 8.69E-33 |
| HSPB7 | -3.53 | 4.34E-33 |
| STX11 | -3.53 | 3.93E-37 |
| DDR2 | -3.53 | 3.85E-24 |
| NMB | -3.54 | 2.26E-24 |
| ACSM5 | -3.56 | 2.58E-34 |
| ADIRF | -3.56 | 3.92E-11 |
| PLXNA4 | -3.57 | 6.56E-24 |
| PFKFB1 | -3.57 | 7.19E-31 |
| PDE11A | -3.57 | 2.59E-29 |
| GPR146 | -3.58 | 2.26E-21 |
| MRC1 | -3.59 | 2.30E-14 |
| NTRK2 | -3.59 | 8.04E-08 |
| SLC4A4 | -3.60 | 4.18E-12 |
| RNF150 | -3.61 | 3.83E-20 |
| ME1 | -3.61 | 2.17E-12 |
| TMEM37 | -3.62 | 2.59E-19 |
| GNAI1 | -3.63 | 2.37E-15 |
| IGFBP6 | -3.63 | 2.28E-18 |
| TWIST2 | -3.64 | 5.44E-23 |
| ANGPTL1 | -3.64 | 6.30E-19 |
| FXYD1 | -3.64 | 8.58E-25 |
| PCDH9 | -3.65 | 1.16E-24 |
| CALB2 | -3.67 | 1.23E-15 |
| MTURN | -3.70 | 2.66E-17 |
| CDKN2C | -3.72 | 3.04E-21 |
| LRRN3 | -3.73 | 4.50E-21 |
| PPP1R14A | -3.73 | 8.03E-14 |
| 1-Mar | -3.74 | 2.26E-21 |
| TMEM132C | -3.77 | 3.93E-37 |
| FOSB | -3.78 | 1.46E-09 |
| ALDOC | -3.79 | 4.27E-17 |
| PRKAR2B | -3.81 | 1.77E-13 |
| BHMT2 | -3.82 | 5.51E-27 |
| HCAR3 | -3.86 | 9.88E-20 |
| GNG2 | -3.86 | 3.22E-20 |
| CIDEA | -3.86 | 3.10E-36 |
| COX7A1 | -3.87 | 2.40E-16 |
| PDZD2 | -3.90 | 1.72E-14 |
| PPP2R1B | -3.92 | 1.20E-20 |
| CCN5 | -3.94 | 2.45E-18 |
| CCDC69 | -3.95 | 5.36E-20 |
| CPM | -3.97 | 4.10E-25 |
| CLMP | -3.98 | 1.30E-22 |
| LVRN | -4.02 | 2.04E-34 |
| TNMD | -4.03 | 3.06E-27 |
| OGN | -4.03 | 1.15E-12 |
| GPR34 | -4.04 | 8.44E-24 |
| LINC01697 | -4.04 | 3.93E-37 |
| LRRN4CL | -4.05 | 3.07E-21 |
| NPR3 | -4.08 | 3.07E-24 |
| CAV2 | -4.08 | 6.80E-15 |
| ITGA7 | -4.08 | 7.76E-19 |
| MMD | -4.09 | 1.33E-24 |
| ALDH1A1 | -4.10 | 1.69E-21 |
| ACACB | -4.10 | 2.56E-13 |
| AKAP12 | -4.10 | 2.14E-22 |
| CPED1 | -4.12 | 1.10E-20 |
| DPT | -4.15 | 1.49E-16 |
| CCDC3 | -4.20 | 1.44E-21 |
| MME | -4.21 | 2.22E-28 |
| AOC3 | -4.26 | 5.72E-15 |
| KCNIP2 | -4.28 | 3.19E-34 |
| AKR1C3 | -4.28 | 2.04E-12 |
| DOCK11 | -4.30 | 4.32E-20 |
| LYVE1 | -4.31 | 3.10E-36 |
| SLC16A7 | -4.33 | 1.89E-18 |
| PLAC9 | -4.39 | 1.26E-24 |
| ATP1A2 | -4.44 | 1.44E-21 |
| ADRB1 | -4.45 | 2.59E-20 |
| ADH1C | -4.47 | 3.04E-34 |
| LIPE | -4.50 | 2.69E-28 |
| SGCG | -4.50 | 6.72E-27 |
| EBF1 | -4.50 | 1.56E-18 |
| TF | -4.51 | 1.05E-13 |
| ECM2 | -4.52 | 4.11E-16 |
| ASPA | -4.53 | 2.53E-33 |
| CFD | -4.57 | 2.96E-11 |
| LGALS12 | -4.58 | 4.62E-21 |
| ITIH5 | -4.58 | 7.69E-17 |
| FHL1 | -4.58 | 8.42E-15 |
| PALMD | -4.58 | 9.15E-18 |
| IGF1 | -4.58 | 1.51E-14 |
| KLHL31 | -4.59 | 8.98E-28 |
| PPARG | -4.60 | 2.66E-25 |
| LINC01140 | -4.61 | 2.88E-26 |
| PDK4 | -4.62 | 6.68E-13 |
| CRYAB | -4.62 | 4.84E-16 |
| GHR | -4.64 | 1.90E-15 |
| PPP1R1A | -4.67 | 8.75E-19 |
| TMEM100 | -4.70 | 6.74E-21 |
| ACVR1C | -4.71 | 1.45E-32 |
| MRAP | -4.80 | 4.68E-29 |
| GPX3 | -4.84 | 1.85E-13 |
| LOC101926960 | -4.84 | 1.00E-26 |
| CES1 | -4.84 | 1.13E-18 |
| DGAT2 | -4.85 | 2.60E-17 |
| GYG2 | -4.85 | 1.67E-15 |
| CAVIN2 | -4.92 | 5.92E-28 |
| MAOA | -4.96 | 3.62E-17 |
| ABCA8 | -4.96 | 5.16E-14 |
| SLC19A3 | -4.98 | 1.88E-30 |
| TRARG1 | -5.01 | 8.79E-28 |
| DEFB132 | -5.14 | 8.79E-28 |
| C14orf180 | -5.14 | 6.50E-28 |
| S100B | -5.16 | 9.50E-18 |
| KLB | -5.21 | 1.20E-29 |
| HSD11B1-AS1 | -5.33 | 1.67E-29 |
| GPAM | -5.33 | 2.54E-18 |
| CHRDL1 | -5.36 | 8.29E-14 |
| SCARA5 | -5.37 | 3.75E-29 |
| ADIPOQ | -5.45 | 2.74E-12 |
| G0S2 | -5.48 | 1.65E-15 |
| CD36 | -5.53 | 3.99E-12 |
| THRSP | -5.58 | 5.82E-13 |
| PCOLCE2 | -5.62 | 1.21E-16 |
| CDO1 | -5.67 | 1.32E-22 |
| AKR1C1 | -5.73 | 9.37E-15 |
| PLIN4 | -5.76 | 2.96E-18 |
| HBB | -5.87 | 5.03E-20 |
| LPL | -5.91 | 3.25E-15 |
| AKR1C2 | -5.91 | 4.38E-15 |
| GPD1 | -5.92 | 2.47E-23 |
| PCK1 | -5.98 | 2.69E-31 |
| TIMP4 | -6.12 | 6.98E-24 |
| CIDEC | -6.13 | 7.98E-22 |
| PLIN1 | -6.18 | 2.32E-17 |
| FABP4 | -6.34 | 6.18E-12 |
| ADH1B | -6.43 | 5.16E-14 |
| RBP4 | -6.94 | 2.94E-22 |
| LEP | -7.34 | 2.54E-20 |
