# Supplementary material for: Identification of hub genes and small molecule therapeutic drugs related to breast cancer with comprehensive bioinformatics analysis
Source: PeerJ. 2020 Sep 29;8:e9946. doi: 10.7717/peerj.9946 (PMC7556247; doi:10.7717/peerj.9946)
Supplement: Supplemental Information 13 [file peerj-08-9946-s013.docx]

| **ID** | **Log2FC** | **adj.P.Val** |
| --- | --- | --- |
| WIF1 | -4.12 | 5.45E-04 |
| DLK1 | -3.98 | 1.05E-03 |
| CAPN6 | -3.94 | 2.07E-03 |
| RFX6 | -3.61 | 5.74E-04 |
| OXTR | -3.56 | 1.26E-07 |
| SCGB3A1 | -3.54 | 1.88E-02 |
| PIGR | -3.50 | 1.69E-03 |
| SLC26A3 | -3.42 | 1.19E-02 |
| CLCA4 | -3.42 | 1.86E-04 |
| MYBPC1 | -3.36 | 1.08E-03 |
| LEP | -3.30 | 1.46E-02 |
| EDN3 | -3.29 | 5.83E-04 |
| TAC1 | -3.23 | 5.36E-03 |
| TNMD | -3.16 | 4.39E-03 |
| ECRG4 | -3.13 | 4.42E-04 |
| ADH1B | -3.13 | 2.06E-02 |
| SCARA5 | -3.11 | 8.08E-04 |
| ARHGAP36 | -3.08 | 1.62E-02 |
| CD300LG | -3.07 | 8.51E-05 |
| TMEM132C | -2.94 | 6.75E-04 |
| COL6A6 | -2.90 | 5.81E-05 |
| CITED1 | -2.87 | 6.85E-03 |
| PPBP | -2.84 | 6.07E-03 |
| CAVIN2 | -2.82 | 7.03E-05 |
| COL17A1 | -2.82 | 2.30E-04 |
| PLIN1 | -2.81 | 1.38E-02 |
| LINC01198 | -2.78 | 8.03E-03 |
| PAMR1 | -2.76 | 9.51E-05 |
| OPRPN | -2.74 | 5.08E-04 |
| LOC101927870 | -2.71 | 5.54E-03 |
| TIMP4 | -2.70 | 1.75E-02 |
| KRT14 | -2.70 | 3.65E-02 |
| ATP1A2 | -2.66 | 2.67E-03 |
| SAMD5 | -2.63 | 3.26E-04 |
| NTRK2 | -2.60 | 1.74E-04 |
| RELN | -2.59 | 2.03E-06 |
| CHRDL1 | -2.58 | 1.84E-03 |
| APOD | -2.57 | 6.34E-03 |
| HMGCLL1 | -2.57 | 1.83E-02 |
| ADH1C | -2.57 | 8.66E-03 |
| BTNL9 | -2.57 | 6.38E-05 |
| CA4 | -2.55 | 1.16E-04 |
| MME | -2.55 | 6.69E-04 |
| DCX | -2.54 | 1.26E-02 |
| ALKAL2 | -2.52 | 1.10E-02 |
| SLC6A14 | -2.52 | 4.48E-02 |
| CLDN8 | -2.52 | 1.32E-02 |
| ADAMTS8 | -2.52 | 1.07E-03 |
| ABCA8 | -2.51 | 6.68E-03 |
| SFRP1 | -2.51 | 9.71E-03 |
| KRT15 | -2.50 | 7.71E-03 |
| IL17B | -2.50 | 2.19E-04 |
| SPINK5 | -2.49 | 1.38E-02 |
| ADAMTS9-AS2 | -2.49 | 3.06E-04 |
| CHST9 | -2.46 | 1.51E-02 |
| TP63 | -2.46 | 9.60E-04 |
| HAS3 | -2.46 | 7.28E-05 |
| SYNM | -2.45 | 2.60E-04 |
| DAPL1 | -2.45 | 4.63E-02 |
| LOC100505851 | -2.44 | 2.61E-04 |
| PTPRZ1 | -2.43 | 1.81E-02 |
| OSR1 | -2.42 | 6.00E-03 |
| PI15 | -2.42 | 1.28E-03 |
| KCNE1 | -2.40 | 5.24E-03 |
| SPHKAP | -2.39 | 6.69E-04 |
| IRX1 | -2.38 | 5.61E-03 |
| RYR3 | -2.38 | 4.77E-04 |
| ROPN1 | -2.38 | 4.74E-02 |
| IGSF10 | -2.37 | 1.08E-03 |
| GPIHBP1 | -2.37 | 1.71E-03 |
| SLC27A6 | -2.37 | 2.33E-02 |
| MAMDC2 | -2.37 | 5.19E-05 |
| C1QTNF4 | -2.34 | 1.14E-04 |
| MUC7 | -2.34 | 3.04E-02 |
| PIK3C2G | -2.32 | 1.14E-03 |
| KLHL13 | -2.29 | 2.19E-04 |
| SCN7A | -2.29 | 4.36E-03 |
| CAPZA3 | -2.27 | 2.39E-03 |
| LMO7DN | -2.27 | 4.18E-04 |
| MYH11 | -2.26 | 7.66E-04 |
| S100B | -2.26 | 2.93E-02 |
| IGFBP1 | -2.24 | 1.84E-02 |
| SLC13A2 | -2.23 | 3.26E-02 |
| RERGL | -2.23 | 2.39E-02 |
| CSRNP3 | -2.23 | 1.60E-04 |
| ANGPTL1 | -2.22 | 1.30E-03 |
| OGN | -2.22 | 1.12E-02 |
| KCNJ16 | -2.21 | 5.98E-03 |
| LRRC3B | -2.21 | 9.05E-04 |
| LVRN | -2.21 | 2.77E-02 |
| ADRB1 | -2.20 | 3.72E-02 |
| GDF10 | -2.20 | 1.37E-02 |
| GPD1 | -2.20 | 2.16E-02 |
| PLIN4 | -2.19 | 2.66E-02 |
| ACTG2 | -2.19 | 1.72E-03 |
| PCOLCE2 | -2.19 | 2.93E-02 |
| FOSB | -2.19 | 7.15E-03 |
| SNORD114-3 | -2.17 | 2.69E-03 |
| SCN4B | -2.17 | 6.69E-04 |
| CARMN | -2.17 | 3.48E-05 |
| KIT | -2.16 | 3.10E-03 |
| LIFR | -2.16 | 5.92E-05 |
| SEL1L2 | -2.16 | 8.47E-03 |
| CCL28 | -2.15 | 6.00E-03 |
| CSN3 | -2.15 | 1.35E-02 |
| FGF2 | -2.13 | 2.19E-03 |
| CFD | -2.11 | 8.25E-04 |
| CXCL2 | -2.11 | 5.24E-03 |
| BRINP1 | -2.11 | 1.06E-02 |
| RSPO1 | -2.11 | 1.13E-03 |
| LYVE1 | -2.10 | 9.87E-03 |
| PRO1804 | -2.10 | 1.46E-02 |
| ISM1 | -2.10 | 3.87E-05 |
| LRRN4CL | -2.09 | 2.06E-05 |
| CIDEA | -2.08 | 8.63E-03 |
| SCN2A | -2.08 | 5.09E-03 |
| TMEM178A | -2.08 | 9.90E-05 |
| DMD | -2.07 | 3.87E-05 |
| ABCB5 | -2.07 | 4.57E-02 |
| SPRY2 | -2.06 | 2.03E-06 |
| TGFBR3 | -2.06 | 3.97E-03 |
| FAM3B | -2.06 | 4.60E-02 |
| DSC1 | -2.05 | 2.23E-02 |
| MEOX2 | -2.05 | 4.42E-03 |
| PTHLH | -2.04 | 3.93E-03 |
| GPC3 | -2.03 | 1.31E-03 |
| HOXA5 | -2.03 | 4.77E-04 |
| MAOA | -2.01 | 9.05E-04 |
| SRPX | -2.01 | 3.08E-05 |
| AK5 | -2.00 | 3.99E-03 |
| EGR3 | -2.00 | 1.06E-03 |
| ACACB | -1.99 | 3.38E-04 |
| SVEP1 | -1.99 | 6.20E-03 |
| PAK5 | -1.99 | 1.28E-05 |
| MYOM1 | -1.99 | 4.53E-03 |
| CNN1 | -1.98 | 3.48E-05 |
| FMO2 | -1.98 | 2.36E-03 |
| PTN | -1.98 | 5.45E-03 |
| C16orf89 | -1.97 | 3.06E-02 |
| HSD11B1-AS1 | -1.97 | 1.23E-02 |
| ID4 | -1.97 | 5.15E-03 |
| ZBTB16 | -1.95 | 5.46E-03 |
| IL33 | -1.95 | 6.82E-03 |
| RBP4 | -1.95 | 3.47E-02 |
| ADAMTS5 | -1.93 | 4.92E-05 |
| COL4A6 | -1.93 | 8.58E-03 |
| SEMA3G | -1.92 | 1.13E-03 |
| CLDN11 | -1.91 | 1.14E-03 |
| NTS | -1.91 | 2.09E-02 |
| TSHZ2 | -1.90 | 1.06E-05 |
| ENPP6 | -1.90 | 6.75E-03 |
| SLC19A3 | -1.90 | 1.35E-02 |
| PDK4 | -1.90 | 2.48E-03 |
| LPL | -1.89 | 3.10E-03 |
| CYP7A1 | -1.88 | 3.33E-02 |
| EPHB1 | -1.87 | 3.16E-04 |
| SMIM10L2A | -1.87 | 5.98E-03 |
| PLAC9 | -1.87 | 6.77E-03 |
| APCDD1L | -1.87 | 2.40E-02 |
| AOC3 | -1.86 | 1.06E-03 |
| MATN2 | -1.86 | 1.06E-03 |
| TRARG1 | -1.85 | 1.74E-02 |
| GPM6B | -1.84 | 4.70E-04 |
| RNASE7 | -1.84 | 1.24E-02 |
| TCEAL7 | -1.83 | 1.99E-05 |
| LINC01883 | -1.81 | 3.77E-03 |
| FHL1 | -1.81 | 4.02E-03 |
| BCHE | -1.81 | 4.84E-02 |
| PTGS2 | -1.80 | 3.06E-02 |
| KCNH8 | -1.80 | 2.45E-02 |
| PRSS12 | -1.80 | 3.53E-02 |
| GIPC2 | -1.80 | 1.96E-03 |
| EGR2 | -1.80 | 9.91E-05 |
| NRXN1 | -1.79 | 3.58E-02 |
| SLC25A27 | -1.79 | 5.33E-04 |
| DDX19B | -1.79 | 2.01E-02 |
| SLC7A3 | -1.79 | 2.36E-03 |
| BMPER | -1.78 | 4.00E-02 |
| TSLP | -1.78 | 1.99E-02 |
| EBF1 | -1.78 | 3.20E-05 |
| GALNT16 | -1.78 | 2.46E-02 |
| NPR1 | -1.78 | 3.08E-03 |
| HOXA2 | -1.78 | 1.12E-02 |
| CHL1 | -1.77 | 1.84E-03 |
| EDNRB | -1.76 | 2.03E-06 |
| ITIH5 | -1.76 | 3.98E-04 |
| ABCA6 | -1.76 | 5.92E-05 |
| NAALAD2 | -1.75 | 3.00E-02 |
| C2orf88 | -1.75 | 5.04E-05 |
| TRMT9B | -1.75 | 1.84E-03 |
| PLAGL1 | -1.75 | 1.51E-04 |
| PLEKHH2 | -1.75 | 5.31E-03 |
| MFSD4A-AS1 | -1.74 | 4.54E-02 |
| OR10D3 | -1.74 | 1.99E-05 |
| TMEM213 | -1.74 | 4.57E-02 |
| FBXL22 | -1.74 | 4.61E-02 |
| TENM1 | -1.74 | 1.21E-03 |
| TENM2 | -1.74 | 1.18E-02 |
| ANGPTL7 | -1.73 | 4.32E-03 |
| IL20RA | -1.73 | 2.89E-02 |
| SSTR1 | -1.73 | 3.52E-03 |
| PDGFD | -1.73 | 9.09E-05 |
| PLP1 | -1.72 | 1.19E-02 |
| LINC01697 | -1.72 | 1.04E-03 |
| CXCL6 | -1.72 | 2.02E-02 |
| FAM3D | -1.72 | 3.82E-02 |
| GAL3ST1 | -1.72 | 2.84E-03 |
| PHYHIP | -1.71 | 5.04E-04 |
| BOC | -1.71 | 1.69E-04 |
| NR3C2 | -1.70 | 2.28E-04 |
| ACVR1C | -1.70 | 4.00E-03 |
| FAM189A2 | -1.70 | 9.65E-04 |
| CD36 | -1.70 | 3.06E-02 |
| RHOJ | -1.69 | 1.60E-06 |
| MIR205HG | -1.69 | 3.80E-03 |
| GYG2 | -1.69 | 4.39E-03 |
| KLHL29 | -1.68 | 3.07E-04 |
| BMX | -1.68 | 2.72E-02 |
| HLF | -1.68 | 5.33E-03 |
| HPSE2 | -1.68 | 5.96E-04 |
| LAMA3 | -1.67 | 6.76E-03 |
| DPY19L2 | -1.67 | 9.70E-03 |
| MT1M | -1.66 | 3.36E-02 |
| PENK | -1.65 | 4.46E-03 |
| CREB5 | -1.65 | 1.76E-05 |
| PDE1C | -1.65 | 5.15E-03 |
| LDB2 | -1.65 | 7.34E-07 |
| AMIGO2 | -1.65 | 5.78E-04 |
| EGFR | -1.65 | 3.01E-03 |
| SCN2B | -1.65 | 1.15E-02 |
| ACADL | -1.65 | 1.36E-02 |
| FREM1 | -1.64 | 1.16E-04 |
| CCDC178 | -1.64 | 2.79E-02 |
| MRAP | -1.64 | 8.85E-03 |
| TMED6 | -1.63 | 4.49E-02 |
| PTCHD1 | -1.63 | 4.83E-02 |
| WDR86 | -1.63 | 1.98E-02 |
| HOXA3 | -1.62 | 7.95E-04 |
| RUNDC3B | -1.62 | 1.25E-05 |
| LAMC3 | -1.62 | 5.25E-03 |
| TM4SF18 | -1.62 | 5.00E-05 |
| CDO1 | -1.62 | 1.71E-03 |
| RBP7 | -1.62 | 2.56E-03 |
| HAND2-AS1 | -1.62 | 4.78E-02 |
| MICU3 | -1.61 | 2.48E-03 |
| PLPPR1 | -1.61 | 3.58E-02 |
| NDRG2 | -1.61 | 9.74E-04 |
| RBMS3 | -1.61 | 3.71E-04 |
| STX19 | -1.61 | 4.16E-02 |
| ITM2A | -1.60 | 2.28E-03 |
| MMRN1 | -1.60 | 3.59E-02 |
| EGF | -1.60 | 4.63E-02 |
| ADAMTS18 | -1.60 | 3.88E-03 |
| DIO3OS | -1.60 | 2.95E-02 |
| TAT | -1.59 | 3.58E-02 |
| LOC105379426 | -1.59 | 8.68E-03 |
| FGF7 | -1.59 | 1.95E-04 |
| LAMC2 | -1.59 | 1.56E-02 |
| CACHD1 | -1.59 | 1.59E-04 |
| CAV1 | -1.58 | 2.89E-04 |
| LYPLAL1-AS1 | -1.58 | 1.65E-02 |
| CORO2B | -1.58 | 2.85E-02 |
| FIGN | -1.58 | 3.60E-02 |
| ENPP2 | -1.58 | 8.73E-04 |
| SYNPO2 | -1.58 | 3.94E-04 |
| CXCL12 | -1.58 | 3.22E-04 |
| LAMA1 | -1.57 | 4.29E-02 |
| FAT2 | -1.57 | 3.58E-02 |
| TFPI | -1.57 | 3.11E-05 |
| SEMA3D | -1.57 | 3.45E-02 |
| ASTN1 | -1.56 | 2.66E-02 |
| ABCA9 | -1.56 | 2.39E-03 |
| DENND2A | -1.56 | 3.14E-03 |
| GHR | -1.56 | 1.61E-02 |
| RNF128 | -1.56 | 6.15E-03 |
| KL | -1.56 | 1.88E-04 |
| EBF3 | -1.55 | 3.66E-04 |
| HS3ST4 | -1.55 | 5.38E-04 |
| PROS1 | -1.55 | 6.47E-05 |
| CCDC85A | -1.55 | 4.83E-02 |
| CCDC8 | -1.54 | 7.91E-05 |
| IGFBP6 | -1.54 | 1.10E-04 |
| SOX7 | -1.54 | 1.17E-03 |
| PLA2R1 | -1.54 | 8.88E-05 |
| ASPA | -1.54 | 8.00E-03 |
| CDKN1C | -1.54 | 7.91E-05 |
| KIAA0408 | -1.53 | 4.30E-02 |
| TMEM100 | -1.53 | 2.00E-02 |
| FGF1 | -1.53 | 1.10E-04 |
| G0S2 | -1.53 | 7.97E-03 |
| LOC100505774 | -1.53 | 7.31E-03 |
| KCNA1 | -1.53 | 1.31E-03 |
| LINC00968 | -1.53 | 2.71E-02 |
| IGF1 | -1.52 | 1.31E-02 |
| SLC35F3 | -1.52 | 4.59E-02 |
| LAMB3 | -1.52 | 1.46E-02 |
| TCEAL2 | -1.52 | 2.45E-02 |
| ANK2 | -1.52 | 2.24E-03 |
| DPT | -1.51 | 6.03E-03 |
| SEMA5A | -1.51 | 3.11E-05 |
| IL17RD | -1.51 | 1.77E-03 |
| SCN3B | -1.51 | 3.32E-02 |
| SPATA18 | -1.50 | 1.85E-02 |
| LOXL4 | -1.50 | 3.87E-05 |
| PREX2 | -1.50 | 1.14E-03 |
| MTURN | -1.50 | 2.00E-02 |
| RASL10A | -1.50 | 5.11E-03 |
| EGR1 | -1.49 | 2.06E-03 |
| NTF3 | -1.49 | 3.40E-02 |
| PPP1R14A | -1.49 | 1.12E-03 |
| CLDND2 | -1.48 | 3.60E-02 |
| PLSCR4 | -1.48 | 3.66E-04 |
| LOC389895 | -1.48 | 2.53E-03 |
| LRFN5 | -1.48 | 4.38E-03 |
| LRRTM2 | -1.48 | 3.20E-03 |
| LOC283788 | -1.48 | 3.37E-02 |
| CIDEC | -1.47 | 2.10E-02 |
| RUNX1T1 | -1.47 | 8.19E-03 |
| MYOM2 | -1.46 | 3.14E-02 |
| NR2F2-AS1 | -1.45 | 2.88E-02 |
| ZNF837 | -1.45 | 3.64E-02 |
| PALMD | -1.45 | 3.07E-03 |
| NFIX | -1.45 | 2.77E-02 |
| FZD7 | -1.45 | 3.58E-04 |
| FAM13C | -1.45 | 1.28E-04 |
| GAS2 | -1.45 | 2.07E-02 |
| LINC02712 | -1.45 | 3.82E-02 |
| TNN | -1.44 | 9.45E-03 |
| RASSF6 | -1.44 | 1.81E-02 |
| PGM5 | -1.44 | 7.48E-03 |
| IRS2 | -1.44 | 4.64E-03 |
| SH3BGRL2 | -1.44 | 1.04E-03 |
| CASQ2 | -1.44 | 4.23E-02 |
| PID1 | -1.43 | 1.08E-03 |
| LOC440570 | -1.43 | 1.00E-02 |
| ZNF204P | -1.43 | 6.73E-03 |
| INMT | -1.42 | 7.97E-03 |
| GSTM5 | -1.42 | 1.04E-03 |
| CX3CL1 | -1.42 | 1.32E-04 |
| MEOX1 | -1.42 | 2.65E-03 |
| GPAM | -1.42 | 1.34E-03 |
| FAM13A | -1.41 | 2.25E-04 |
| ARHGAP20 | -1.41 | 5.97E-05 |
| PLEKHA4 | -1.41 | 4.51E-02 |
| CNTNAP3P2 | -1.40 | 1.60E-02 |
| TMEM88 | -1.40 | 5.46E-03 |
| SEMA6D | -1.40 | 6.34E-03 |
| MEG3 | -1.39 | 2.98E-02 |
| LOC100505874 | -1.39 | 2.38E-02 |
| FGF7P3 | -1.39 | 9.09E-05 |
| AKR1C3 | -1.39 | 4.44E-02 |
| HYMAI | -1.39 | 7.16E-03 |
| AASS | -1.39 | 1.16E-04 |
| TMTC1 | -1.38 | 6.82E-03 |
| MYLK | -1.38 | 1.04E-03 |
| MAFF | -1.38 | 1.08E-03 |
| APCDD1 | -1.38 | 8.84E-04 |
| KANK3 | -1.38 | 1.17E-04 |
| LINC00842 | -1.37 | 3.89E-02 |
| ANKRD29 | -1.37 | 4.17E-03 |
| LMOD1 | -1.37 | 1.07E-03 |
| PRDM11 | -1.36 | 1.80E-03 |
| NAP1L2 | -1.36 | 4.03E-02 |
| ST8SIA2 | -1.36 | 4.91E-02 |
| NECTIN3-AS1 | -1.36 | 2.72E-02 |
| RASSF9 | -1.35 | 9.72E-03 |
| CKMT2 | -1.35 | 9.15E-03 |
| FOXP2 | -1.35 | 9.60E-03 |
| PCDH9 | -1.35 | 2.06E-02 |
| LHFPL6 | -1.35 | 2.19E-04 |
| STOX2 | -1.35 | 3.08E-02 |
| TPT1-AS1 | -1.35 | 5.28E-04 |
| ALDH1A1 | -1.34 | 9.87E-03 |
| DEFB124 | -1.34 | 1.22E-02 |
| GPRASP1 | -1.34 | 9.09E-05 |
| AKAP12 | -1.33 | 5.13E-03 |
| SCN4A | -1.33 | 2.84E-02 |
| MYH3 | -1.33 | 4.86E-02 |
| SEMA6A | -1.33 | 2.00E-02 |
| NFASC | -1.33 | 3.26E-02 |
| AFP | -1.33 | 9.25E-03 |
| PLPP3 | -1.32 | 5.80E-06 |
| PPARG | -1.32 | 2.54E-02 |
| L3MBTL4 | -1.32 | 3.66E-02 |
| AMPD1 | -1.32 | 1.35E-02 |
| PAK3 | -1.32 | 1.03E-04 |
| SOBP | -1.32 | 8.51E-05 |
| MIR100HG | -1.32 | 1.90E-03 |
| TPTEP1 | -1.32 | 1.33E-02 |
| RNF186 | -1.32 | 2.15E-02 |
| GULP1 | -1.32 | 4.00E-03 |
| THRB | -1.31 | 6.07E-03 |
| LIPC | -1.31 | 1.20E-02 |
| TDRD6 | -1.31 | 5.39E-03 |
| WDFY3-AS2 | -1.31 | 1.03E-03 |
| HOXA7 | -1.31 | 1.46E-02 |
| MAML2 | -1.31 | 1.71E-04 |
| DLC1 | -1.30 | 1.35E-03 |
| CPED1 | -1.30 | 2.35E-03 |
| CAV2 | -1.30 | 3.10E-03 |
| TRIM9 | -1.30 | 4.98E-03 |
| TRPC6 | -1.30 | 2.45E-02 |
| PCDH18 | -1.30 | 3.81E-04 |
| FAM110D | -1.29 | 1.14E-02 |
| ECM2 | -1.29 | 7.50E-03 |
| FAM126A | -1.29 | 3.66E-04 |
| NSG1 | -1.29 | 1.23E-02 |
| KLF3-AS1 | -1.29 | 5.14E-03 |
| ACKR1 | -1.29 | 3.19E-02 |
| SLAIN1 | -1.29 | 4.98E-02 |
| MEIS2 | -1.28 | 1.17E-03 |
| DUSP6 | -1.28 | 5.24E-03 |
| CLDN5 | -1.28 | 2.48E-03 |
| PKDREJ | -1.28 | 3.20E-02 |
| CYYR1 | -1.28 | 1.59E-04 |
| PDZD2 | -1.28 | 7.57E-03 |
| PER2 | -1.27 | 3.22E-04 |
| SLC16A7 | -1.27 | 5.24E-03 |
| GAS7 | -1.27 | 4.42E-03 |
| LAMA2 | -1.27 | 3.88E-02 |
| PDGFRA | -1.26 | 1.25E-03 |
| ARMCX4 | -1.26 | 2.25E-02 |
| LOC101928519 | -1.26 | 4.50E-02 |
| RCAN1 | -1.26 | 1.04E-02 |
| WLS | -1.26 | 3.53E-02 |
| IRS1 | -1.26 | 2.20E-02 |
| P3H2 | -1.25 | 3.26E-02 |
| BEND5 | -1.25 | 4.56E-03 |
| ABI3BP | -1.25 | 4.18E-02 |
| USP44 | -1.25 | 3.96E-02 |
| AQP1 | -1.25 | 4.72E-03 |
| GGTA1P | -1.25 | 7.31E-03 |
| KLF4 | -1.25 | 1.12E-02 |
| TESC | -1.24 | 2.23E-04 |
| LINC00893 | -1.24 | 1.44E-02 |
| SH3RF2 | -1.24 | 3.09E-02 |
| GNAI1 | -1.24 | 7.86E-03 |
| ADAMTS1 | -1.24 | 1.03E-02 |
| ROR1 | -1.23 | 8.20E-03 |
| OLFML2A | -1.23 | 3.02E-03 |
| ZNF300P1 | -1.23 | 1.17E-02 |
| CACNA1G | -1.23 | 7.92E-03 |
| DCLK1 | -1.22 | 1.82E-02 |
| ANO1 | -1.22 | 4.54E-02 |
| RSPO3 | -1.22 | 5.91E-03 |
| MOB3B | -1.21 | 3.56E-03 |
| NFIB | -1.21 | 2.00E-02 |
| ABCA5 | -1.21 | 1.04E-03 |
| DPYS | -1.21 | 4.20E-02 |
| TNS1 | -1.21 | 9.74E-04 |
| ADAM33 | -1.20 | 9.70E-04 |
| SYN2 | -1.20 | 1.47E-03 |
| TSPAN7 | -1.20 | 2.89E-03 |
| NAV2 | -1.20 | 1.91E-02 |
| HOMER2 | -1.20 | 1.57E-02 |
| ADRB2 | -1.20 | 3.50E-02 |
| PHLDA1 | -1.20 | 2.74E-03 |
| NPY6R | -1.19 | 4.26E-02 |
| SMAD9 | -1.19 | 2.82E-03 |
| GPX3 | -1.19 | 4.67E-02 |
| TNS2 | -1.19 | 5.21E-03 |
| PLXNA4 | -1.18 | 1.76E-02 |
| COX7A1 | -1.18 | 4.32E-03 |
| FXYD1 | -1.18 | 2.11E-03 |
| KCND1 | -1.17 | 2.78E-02 |
| SPRY1 | -1.17 | 1.43E-03 |
| TTC28 | -1.17 | 3.66E-04 |
| OGFRL1 | -1.17 | 1.43E-04 |
| ZBTB20 | -1.17 | 3.49E-03 |
| CRIM1 | -1.17 | 1.07E-02 |
| CLIP4 | -1.17 | 2.36E-02 |
| NR4A1 | -1.17 | 2.13E-02 |
| KIF7 | -1.17 | 4.01E-02 |
| PDGFA | -1.16 | 1.99E-05 |
| GREM2 | -1.16 | 9.25E-03 |
| TCF7L2 | -1.16 | 2.23E-04 |
| EBF2 | -1.16 | 7.29E-03 |
| RAPGEF3 | -1.15 | 4.77E-02 |
| NAT8L | -1.15 | 2.46E-02 |
| ACSM3 | -1.15 | 3.44E-02 |
| SOCS2 | -1.15 | 1.25E-02 |
| FMO1 | -1.15 | 1.04E-02 |
| ZDBF2 | -1.14 | 3.44E-02 |
| HEY2 | -1.14 | 3.49E-02 |
| USHBP1 | -1.14 | 2.48E-03 |
| CFH | -1.14 | 4.32E-03 |
| IFFO2 | -1.14 | 7.16E-03 |
| F10 | -1.13 | 3.78E-02 |
| BARX1-DT | -1.13 | 3.62E-02 |
| CPE | -1.13 | 1.50E-02 |
| KCNB1 | -1.13 | 3.29E-02 |
| SMOC2 | -1.13 | 3.30E-02 |
| SP3P | -1.13 | 3.36E-02 |
| KCNMB1 | -1.13 | 4.54E-03 |
| MAP2 | -1.12 | 4.47E-02 |
| KLB | -1.12 | 5.65E-03 |
| ADCYAP1R1 | -1.12 | 8.07E-06 |
| GLI1 | -1.12 | 4.47E-02 |
| NIPSNAP3B | -1.12 | 1.25E-02 |
| JAM2 | -1.12 | 1.20E-04 |
| RNF180 | -1.11 | 2.79E-02 |
| PTPN14 | -1.11 | 1.33E-03 |
| GRAMD2B | -1.11 | 3.11E-05 |
| GOLGA8A | -1.11 | 4.84E-02 |
| ANGPT1 | -1.11 | 3.83E-02 |
| ITIH2 | -1.11 | 4.49E-02 |
| LOC100287387 | -1.10 | 1.86E-02 |
| MRGPRF | -1.10 | 1.19E-02 |
| GABPB1-AS1 | -1.10 | 2.70E-02 |
| NRN1 | -1.10 | 1.24E-02 |
| ZNF521 | -1.10 | 2.66E-02 |
| TTN-AS1 | -1.10 | 6.15E-03 |
| GNAL | -1.09 | 6.03E-03 |
| ETV5 | -1.09 | 4.25E-03 |
| LPCAT2 | -1.09 | 7.42E-03 |
| ZNF423 | -1.09 | 2.08E-02 |
| YPEL4 | -1.09 | 4.95E-02 |
| FAT4 | -1.09 | 5.17E-04 |
| LINC01140 | -1.09 | 4.88E-02 |
| MAGI2-AS3 | -1.09 | 1.04E-03 |
| RBMS3-AS3 | -1.09 | 4.30E-02 |
| MYCT1 | -1.08 | 2.71E-03 |
| ZCCHC12 | -1.08 | 2.70E-02 |
| EFEMP1 | -1.08 | 2.35E-02 |
| NEDD9 | -1.08 | 2.64E-03 |
| CTSG | -1.08 | 2.52E-03 |
| MMRN2 | -1.08 | 3.06E-04 |
| SETBP1 | -1.08 | 4.88E-02 |
| TRPC1 | -1.08 | 5.58E-03 |
| GSN | -1.08 | 2.21E-04 |
| FCER1A | -1.08 | 4.25E-02 |
| PRDM5 | -1.08 | 8.50E-04 |
| EVA1C | -1.07 | 3.06E-02 |
| ARHGAP6 | -1.07 | 5.17E-03 |
| GRK3 | -1.07 | 1.06E-03 |
| GNG11 | -1.07 | 2.23E-04 |
| KLHL31 | -1.07 | 1.60E-02 |
| CFAP69 | -1.07 | 3.26E-02 |
| TMOD1 | -1.07 | 1.31E-02 |
| PTPRB | -1.07 | 2.89E-03 |
| BHLHE41 | -1.07 | 6.00E-03 |
| EMCN | -1.06 | 8.00E-04 |
| ITPR1 | -1.06 | 2.11E-02 |
| RBBP8 | -1.06 | 5.87E-03 |
| ROBO4 | -1.06 | 1.87E-04 |
| VWF | -1.06 | 2.21E-03 |
| LRIG3 | -1.06 | 1.44E-02 |
| TWIST2 | -1.06 | 1.35E-02 |
| CLMP | -1.06 | 7.83E-03 |
| SOCS3 | -1.06 | 2.66E-02 |
| ST7-AS1 | -1.05 | 1.06E-02 |
| ACSBG2 | -1.05 | 4.91E-02 |
| ETV1 | -1.05 | 6.07E-03 |
| PELI2 | -1.05 | 1.20E-02 |
| GPR146 | -1.05 | 7.36E-03 |
| FAM20A | -1.05 | 2.70E-02 |
| LRIG2-DT | -1.05 | 2.05E-02 |
| PCNX1 | -1.04 | 2.98E-05 |
| ANKRD35 | -1.04 | 2.20E-02 |
| SASH1 | -1.04 | 6.34E-03 |
| GRAMD1C | -1.04 | 2.85E-03 |
| SH3D19 | -1.04 | 4.21E-03 |
| PDE5A | -1.03 | 3.06E-02 |
| DCHS1 | -1.03 | 4.78E-03 |
| N4BP2L1 | -1.03 | 7.57E-04 |
| LY75 | -1.03 | 1.70E-02 |
| ARRDC3 | -1.03 | 1.46E-04 |
| KLF9 | -1.03 | 1.07E-03 |
| PNMA8B | -1.03 | 9.57E-03 |
| CRYBG3 | -1.02 | 9.93E-03 |
| DST | -1.02 | 2.84E-03 |
| PIK3R1 | -1.02 | 3.38E-03 |
| RECK | -1.02 | 6.43E-03 |
| SHANK3 | -1.02 | 3.29E-02 |
| NDN | -1.02 | 5.41E-03 |
| ZC3H12C | -1.02 | 2.45E-02 |
| CAB39L | -1.02 | 4.75E-03 |
| AMOTL2 | -1.02 | 1.96E-03 |
| INPP1 | -1.01 | 1.80E-04 |
| PTCH1 | -1.01 | 4.16E-02 |
| HOTAIRM1 | -1.01 | 5.42E-03 |
| SOX17 | -1.01 | 8.97E-03 |
| TGFBR2 | -1.01 | 8.08E-04 |
| CGNL1 | -1.01 | 2.74E-02 |
| TRPM3 | -1.01 | 4.53E-02 |
| SGCE | -1.01 | 3.35E-02 |
| DPYSL2 | -1.01 | 4.18E-04 |
| MSRB3 | -1.01 | 2.74E-02 |
| NECTIN3 | -1.00 | 1.01E-02 |
| FTO | -1.00 | 7.60E-05 |
| PPP1R12B | -1.00 | 1.70E-04 |
| ZFP36 | -1.00 | 2.57E-02 |
| EPB41L4A-AS1 | -1.00 | 5.82E-04 |
| ARHGEF28 | -1.00 | 9.35E-03 |
| MAF | -1.00 | 1.08E-03 |
| SULT1B1 | 1.00 | 3.58E-02 |
| ATP6V0B | 1.00 | 5.81E-05 |
| C1QL1 | 1.00 | 1.60E-04 |
| PPP4C | 1.01 | 1.99E-04 |
| SKA3 | 1.01 | 5.74E-03 |
| CD86 | 1.01 | 6.95E-03 |
| ADRM1 | 1.01 | 3.77E-05 |
| MRPS34 | 1.01 | 4.30E-04 |
| PARP9 | 1.01 | 6.46E-03 |
| MICAL2 | 1.01 | 5.39E-03 |
| NDOR1 | 1.01 | 1.93E-02 |
| PCDH17 | 1.01 | 1.34E-03 |
| DDA1 | 1.02 | 3.87E-05 |
| SLC25A39 | 1.02 | 1.20E-04 |
| RHEBL1 | 1.02 | 4.10E-02 |
| NUP210 | 1.02 | 1.03E-02 |
| C2 | 1.02 | 8.22E-03 |
| IRF7 | 1.02 | 2.26E-03 |
| XXYLT1 | 1.02 | 3.51E-03 |
| IFI35 | 1.02 | 1.19E-02 |
| GINS2 | 1.02 | 3.25E-02 |
| CENPX | 1.02 | 6.07E-03 |
| HAVCR2 | 1.03 | 1.09E-04 |
| AP1M2 | 1.03 | 7.01E-03 |
| H2BS1 | 1.03 | 1.66E-02 |
| MIAT | 1.03 | 3.26E-02 |
| PSRC1 | 1.03 | 1.78E-03 |
| SHCBP1 | 1.03 | 3.77E-02 |
| DDIAS | 1.03 | 4.17E-02 |
| CACNG4 | 1.03 | 3.42E-02 |
| GLRX2 | 1.03 | 7.39E-04 |
| EIF5A | 1.04 | 6.48E-03 |
| LINC00266-1 | 1.04 | 9.54E-03 |
| GCHFR | 1.04 | 2.13E-02 |
| ITGA11 | 1.04 | 1.31E-02 |
| H2BC9 | 1.04 | 1.32E-02 |
| PKM | 1.04 | 8.56E-04 |
| EZH2 | 1.05 | 1.23E-02 |
| PLK4 | 1.05 | 9.52E-03 |
| TCEANC2 | 1.05 | 4.45E-02 |
| TPRN | 1.05 | 4.86E-03 |
| ESPL1 | 1.05 | 7.47E-03 |
| CLEC4E | 1.05 | 3.74E-02 |
| MAP11 | 1.05 | 3.75E-03 |
| FPR1 | 1.05 | 4.27E-03 |
| CAPG | 1.05 | 3.51E-04 |
| TEAD4 | 1.06 | 1.12E-02 |
| ZBTB7B | 1.06 | 3.06E-02 |
| SIX4 | 1.06 | 1.39E-02 |
| PLAUR | 1.06 | 6.05E-04 |
| CD80 | 1.07 | 5.54E-03 |
| KIF18A | 1.07 | 2.22E-02 |
| PGP | 1.07 | 6.99E-04 |
| KRT8 | 1.07 | 2.04E-02 |
| IL4I1 | 1.07 | 4.28E-02 |
| GREM1 | 1.07 | 3.80E-02 |
| CENPK | 1.07 | 7.94E-03 |
| BRIP1 | 1.07 | 2.24E-02 |
| CAVIN4 | 1.07 | 2.72E-02 |
| PACC1 | 1.07 | 3.96E-04 |
| LIME1 | 1.08 | 6.68E-03 |
| STMN1 | 1.08 | 1.23E-02 |
| SCG2 | 1.08 | 4.09E-02 |
| CTSD | 1.08 | 2.52E-03 |
| LOC100506585 | 1.08 | 2.50E-02 |
| CCNF | 1.09 | 3.68E-03 |
| LINC02806 | 1.09 | 9.57E-03 |
| SH2D5 | 1.09 | 3.95E-02 |
| COMMD5 | 1.09 | 3.38E-03 |
| P2RY6 | 1.10 | 4.94E-02 |
| COL5A1 | 1.10 | 3.02E-02 |
| STIL | 1.10 | 3.34E-03 |
| E2F2 | 1.10 | 2.77E-02 |
| PFN1 | 1.10 | 2.03E-06 |
| YIF1A | 1.10 | 2.53E-04 |
| LAGE3 | 1.10 | 3.64E-04 |
| B3GAT3 | 1.10 | 1.14E-04 |
| LEF1 | 1.11 | 6.00E-03 |
| RAB26 | 1.11 | 2.63E-02 |
| FANCI | 1.11 | 2.36E-03 |
| PARVG | 1.11 | 6.05E-04 |
| SLC2A6 | 1.11 | 1.06E-02 |
| ANKRD2 | 1.12 | 3.97E-02 |
| AUNIP | 1.12 | 4.11E-03 |
| METRN | 1.12 | 1.82E-02 |
| IDH2 | 1.12 | 2.33E-03 |
| NUBP2 | 1.12 | 6.38E-03 |
| TAP1 | 1.12 | 4.14E-02 |
| LINC00563 | 1.13 | 2.81E-02 |
| MCM10 | 1.13 | 4.70E-02 |
| DRAP1 | 1.13 | 2.80E-05 |
| ASF1B | 1.13 | 1.43E-03 |
| ECT2 | 1.13 | 2.40E-03 |
| SH3BGRL3 | 1.13 | 4.75E-06 |
| GPR25 | 1.13 | 2.24E-02 |
| TRIM59 | 1.14 | 1.90E-03 |
| LINC00523 | 1.14 | 6.30E-04 |
| PLA2G4E-AS1 | 1.14 | 4.68E-02 |
| NLRC4 | 1.14 | 2.53E-03 |
| LINC02683 | 1.14 | 3.36E-02 |
| FCGR1B | 1.14 | 1.73E-02 |
| PSTPIP1 | 1.14 | 1.91E-03 |
| RNASEH2A | 1.15 | 4.09E-03 |
| POC1A | 1.15 | 4.55E-03 |
| CEACAM4 | 1.15 | 5.28E-04 |
| PKMYT1 | 1.15 | 1.06E-03 |
| C1QTNF12 | 1.15 | 4.57E-02 |
| PPP1CA | 1.15 | 6.96E-05 |
| MKI67 | 1.16 | 5.62E-04 |
| F5 | 1.16 | 6.07E-03 |
| TYMS | 1.16 | 1.15E-02 |
| LINC01094 | 1.16 | 1.42E-03 |
| TRIP13 | 1.16 | 2.57E-02 |
| RUNX1-IT1 | 1.16 | 2.02E-02 |
| SLC38A5 | 1.16 | 4.59E-02 |
| RACGAP1 | 1.16 | 1.14E-03 |
| CEP85 | 1.17 | 1.78E-03 |
| CDKN2D | 1.17 | 1.77E-03 |
| B4GALNT2 | 1.17 | 1.37E-02 |
| TDRKH | 1.17 | 1.19E-02 |
| BGN | 1.17 | 1.19E-02 |
| RAD51 | 1.18 | 5.25E-03 |
| RNFT2 | 1.18 | 8.19E-03 |
| CENPU | 1.18 | 4.98E-03 |
| YIF1B | 1.19 | 3.98E-04 |
| ZNF649-AS1 | 1.19 | 9.79E-03 |
| LIMK1 | 1.20 | 2.68E-04 |
| SLC50A1 | 1.20 | 2.45E-04 |
| IL21R | 1.20 | 8.93E-03 |
| COPE | 1.20 | 5.72E-05 |
| PARPBP | 1.20 | 2.25E-03 |
| C5orf58 | 1.20 | 1.93E-02 |
| FAM104A | 1.20 | 5.19E-05 |
| TLR6 | 1.20 | 2.85E-02 |
| SUGT1P1 | 1.21 | 4.91E-02 |
| LINC01521 | 1.21 | 4.45E-02 |
| ST8SIA5 | 1.21 | 9.52E-04 |
| ATG9B | 1.22 | 4.85E-03 |
| TCF19 | 1.22 | 4.80E-03 |
| GPR35 | 1.22 | 5.93E-03 |
| SLC6A19 | 1.22 | 3.09E-02 |
| ATAD2 | 1.22 | 1.67E-02 |
| MAD2L1 | 1.23 | 1.52E-02 |
| LMNB1 | 1.23 | 1.01E-02 |
| H4C4 | 1.23 | 8.03E-03 |
| ECE2 | 1.23 | 4.14E-03 |
| FLAD1 | 1.23 | 3.87E-05 |
| ZNF367 | 1.23 | 4.43E-03 |
| TSTA3 | 1.24 | 1.67E-03 |
| TACC3 | 1.24 | 2.53E-04 |
| APOC1 | 1.24 | 1.09E-02 |
| COL8A1 | 1.25 | 1.85E-02 |
| PIMREG | 1.25 | 1.98E-03 |
| CD300LF | 1.25 | 1.04E-03 |
| CENPL | 1.25 | 1.35E-03 |
| SKA1 | 1.25 | 2.84E-02 |
| ZNF252P-AS1 | 1.26 | 1.31E-03 |
| AQP9 | 1.26 | 3.39E-02 |
| MYLK-AS1 | 1.26 | 2.00E-02 |
| HAGLROS | 1.27 | 2.92E-02 |
| OAS3 | 1.27 | 1.48E-02 |
| PCOTH | 1.27 | 2.53E-02 |
| FN1 | 1.28 | 4.78E-06 |
| CDCA5 | 1.28 | 4.70E-04 |
| DISP3 | 1.28 | 9.60E-04 |
| LOC101929122 | 1.29 | 3.62E-02 |
| LAMP5 | 1.29 | 2.50E-02 |
| SDS | 1.29 | 2.72E-05 |
| ACTA1 | 1.29 | 4.21E-02 |
| CCDC167 | 1.29 | 2.23E-04 |
| OAS2 | 1.29 | 1.55E-02 |
| UPK2 | 1.30 | 1.01E-02 |
| ORC6 | 1.30 | 2.97E-03 |
| IL1RN | 1.30 | 1.43E-02 |
| CCDC124 | 1.31 | 2.25E-04 |
| CRIP2 | 1.31 | 2.37E-02 |
| CDK5 | 1.31 | 4.75E-06 |
| PAFAH1B3 | 1.31 | 5.18E-04 |
| ZDHHC12 | 1.32 | 1.18E-04 |
| KIF18B | 1.33 | 5.91E-04 |
| ATXN3L | 1.33 | 3.36E-02 |
| CXCR3 | 1.33 | 8.22E-03 |
| B3GNT6 | 1.33 | 1.18E-02 |
| UPP1 | 1.33 | 1.68E-03 |
| RBM34 | 1.34 | 7.26E-03 |
| CD37 | 1.34 | 8.93E-03 |
| ESPNL | 1.35 | 1.12E-03 |
| LOC101928716 | 1.35 | 1.48E-02 |
| SNORA21 | 1.36 | 2.75E-03 |
| RAD51AP1 | 1.36 | 2.58E-02 |
| SQLE | 1.38 | 1.15E-02 |
| SPAG5 | 1.38 | 6.69E-04 |
| AURKB | 1.38 | 5.54E-03 |
| TRPM2 | 1.39 | 5.19E-05 |
| TK1 | 1.39 | 2.53E-04 |
| GPR26 | 1.39 | 2.22E-02 |
| LOC284379 | 1.39 | 4.49E-02 |
| WDR62 | 1.40 | 4.61E-04 |
| LILRB1 | 1.40 | 3.48E-03 |
| CDKN2A | 1.40 | 2.81E-02 |
| SOHLH1 | 1.40 | 4.14E-02 |
| OAS1 | 1.40 | 2.52E-02 |
| NDC80 | 1.40 | 1.26E-02 |
| CDT1 | 1.40 | 2.97E-02 |
| KIF24 | 1.40 | 2.73E-02 |
| TTK | 1.40 | 1.33E-02 |
| SPO11 | 1.41 | 4.65E-02 |
| CRIP1 | 1.41 | 4.29E-02 |
| LOC101927769 | 1.41 | 2.97E-03 |
| STX17-AS1 | 1.41 | 9.52E-03 |
| FPR3 | 1.41 | 1.66E-03 |
| SRPK3 | 1.41 | 4.87E-02 |
| CD70 | 1.42 | 8.58E-03 |
| TLCD1 | 1.42 | 1.01E-02 |
| RMI2 | 1.42 | 6.49E-04 |
| SLAMF8 | 1.43 | 7.16E-03 |
| PGAM5 | 1.43 | 3.49E-02 |
| CCNA2 | 1.44 | 3.14E-03 |
| SPC24 | 1.45 | 5.82E-04 |
| LINC01943 | 1.46 | 2.16E-02 |
| CDH2 | 1.46 | 1.22E-02 |
| MND1 | 1.47 | 1.12E-02 |
| S100A11 | 1.47 | 1.06E-05 |
| IQGAP3 | 1.47 | 1.04E-03 |
| ZNF460 | 1.47 | 2.74E-03 |
| UBE2S | 1.47 | 1.05E-03 |
| GBP5 | 1.47 | 3.25E-02 |
| CCDC187 | 1.48 | 2.84E-03 |
| SGO1 | 1.48 | 1.79E-03 |
| ADM2 | 1.48 | 3.49E-03 |
| H1-6 | 1.48 | 1.01E-02 |
| LIMD2 | 1.49 | 6.15E-03 |
| GINS1 | 1.49 | 4.83E-03 |
| LOC101927539 | 1.49 | 2.25E-03 |
| CENPA | 1.49 | 1.47E-03 |
| KIF26B | 1.49 | 2.35E-03 |
| NUTM1 | 1.49 | 4.63E-03 |
| HMGB3 | 1.49 | 1.66E-03 |
| HMGB3P1 | 1.50 | 1.03E-03 |
| CDC6 | 1.50 | 9.20E-03 |
| SLC35D3 | 1.51 | 4.83E-02 |
| SLC24A2 | 1.51 | 1.06E-02 |
| CD5 | 1.51 | 2.28E-02 |
| KIF23 | 1.52 | 1.22E-03 |
| CAPS | 1.52 | 2.20E-02 |
| KMO | 1.52 | 4.24E-02 |
| TROAP | 1.52 | 5.19E-05 |
| KIF11 | 1.55 | 1.78E-03 |
| HPSE | 1.55 | 1.11E-04 |
| TIGIT | 1.55 | 1.85E-02 |
| BUB1B | 1.56 | 7.25E-04 |
| E2F1 | 1.56 | 2.96E-03 |
| CCNB1 | 1.56 | 1.67E-03 |
| GINS4 | 1.56 | 1.31E-02 |
| NOX4 | 1.58 | 1.95E-04 |
| MTFR2 | 1.59 | 1.90E-03 |
| DCAF4L1 | 1.60 | 1.92E-02 |
| JPT1 | 1.60 | 4.57E-04 |
| CDC20 | 1.60 | 6.78E-03 |
| GPRC5A | 1.60 | 4.38E-02 |
| TNFSF4 | 1.60 | 2.37E-05 |
| LINC02544 | 1.61 | 3.55E-03 |
| CCR7 | 1.61 | 5.15E-03 |
| IL2RG | 1.62 | 8.51E-04 |
| CLNK | 1.63 | 1.10E-02 |
| MFAP2 | 1.63 | 9.09E-05 |
| CENPI | 1.64 | 5.71E-04 |
| CKS2 | 1.64 | 1.35E-04 |
| PLA1A | 1.64 | 3.68E-03 |
| KNL1 | 1.65 | 1.20E-04 |
| PPEF1 | 1.65 | 1.03E-04 |
| EPSTI1 | 1.66 | 8.65E-04 |
| WNT7B | 1.66 | 1.86E-02 |
| HES2 | 1.67 | 4.59E-02 |
| CTHRC1 | 1.67 | 2.81E-06 |
| PRC1 | 1.67 | 5.77E-04 |
| ORC1 | 1.67 | 1.50E-02 |
| C3orf67 | 1.67 | 2.53E-02 |
| DTL | 1.67 | 2.83E-04 |
| RSAD2 | 1.69 | 1.01E-02 |
| OASL | 1.70 | 1.07E-02 |
| HAPLN1 | 1.70 | 1.22E-03 |
| ERCC6L | 1.70 | 3.00E-03 |
| KIF15 | 1.71 | 1.09E-03 |
| BUB1 | 1.71 | 5.09E-04 |
| TRAF2 | 1.72 | 5.09E-04 |
| RAD54L | 1.72 | 1.78E-02 |
| SULF1 | 1.72 | 2.80E-05 |
| C6orf99 | 1.73 | 2.61E-02 |
| H2BC11 | 1.74 | 2.29E-02 |
| RDM1 | 1.74 | 5.69E-05 |
| E2F7 | 1.74 | 3.05E-03 |
| ZWINT | 1.74 | 1.36E-04 |
| KCNJ9 | 1.75 | 1.01E-06 |
| C3orf80 | 1.76 | 1.35E-03 |
| CST2 | 1.77 | 9.11E-03 |
| LINC02245 | 1.77 | 4.00E-03 |
| H2BC3 | 1.77 | 1.11E-02 |
| GPR84 | 1.78 | 6.69E-04 |
| OIP5 | 1.78 | 9.81E-04 |
| CDCA3 | 1.78 | 1.58E-03 |
| CENPE | 1.78 | 1.14E-03 |
| CENPM | 1.79 | 2.97E-03 |
| OR2B2 | 1.79 | 2.75E-03 |
| NCAPH | 1.79 | 4.37E-04 |
| VSX1 | 1.79 | 6.20E-03 |
| DEPDC1B | 1.79 | 2.08E-03 |
| IFI6 | 1.80 | 3.38E-03 |
| UNC5B-AS1 | 1.81 | 1.09E-02 |
| DNASE2B | 1.83 | 8.38E-03 |
| NCAPG | 1.83 | 8.26E-04 |
| KIF14 | 1.84 | 9.05E-04 |
| CCNE2 | 1.84 | 3.22E-04 |
| KIF4A | 1.84 | 1.51E-04 |
| CHAC1 | 1.85 | 3.30E-03 |
| KIF20A | 1.86 | 1.24E-04 |
| GJA3 | 1.87 | 8.51E-04 |
| PTTG3P | 1.87 | 3.11E-05 |
| CDC25C | 1.88 | 4.38E-04 |
| RTKN2 | 1.89 | 3.43E-03 |
| UHRF1 | 1.89 | 2.28E-04 |
| TDO2 | 1.89 | 1.25E-02 |
| CXCL9 | 1.91 | 3.06E-02 |
| PYCR3 | 1.93 | 3.05E-02 |
| ISG15 | 1.93 | 4.45E-03 |
| SPP1 | 1.93 | 8.15E-04 |
| TREM1 | 1.93 | 1.31E-03 |
| CDKN3 | 1.94 | 1.95E-04 |
| CENPF | 1.94 | 1.08E-04 |
| NUSAP1 | 1.94 | 2.21E-05 |
| CKAP2L | 1.95 | 1.88E-04 |
| HES6 | 1.98 | 3.83E-02 |
| MELK | 1.99 | 3.38E-04 |
| PTTG1 | 1.99 | 8.88E-05 |
| PCLAF | 2.00 | 4.75E-06 |
| HMMR | 2.02 | 3.19E-04 |
| RRM2 | 2.02 | 8.88E-05 |
| FAM83D | 2.04 | 2.11E-03 |
| OLR1 | 2.05 | 1.60E-06 |
| DLGAP5 | 2.06 | 4.81E-04 |
| SPC25 | 2.07 | 9.74E-05 |
| CCNB2 | 2.08 | 1.00E-04 |
| DEPDC1 | 2.09 | 8.46E-04 |
| MMP9 | 2.09 | 1.17E-04 |
| LRRC15 | 2.12 | 9.74E-04 |
| PSCA | 2.13 | 3.82E-02 |
| E2F8 | 2.13 | 3.24E-04 |
| PBK | 2.13 | 7.85E-04 |
| FANCB | 2.13 | 2.03E-03 |
| UBE2C | 2.14 | 6.47E-05 |
| CCN4 | 2.15 | 1.99E-05 |
| UBE2T | 2.15 | 5.19E-05 |
| HJURP | 2.15 | 7.00E-05 |
| HSD17B6 | 2.16 | 1.60E-06 |
| H2AC17 | 2.20 | 8.94E-05 |
| FNDC1 | 2.20 | 1.09E-04 |
| TOP2A | 2.23 | 1.69E-05 |
| EXO1 | 2.26 | 4.73E-04 |
| P4HA3 | 2.26 | 8.09E-05 |
| PITX1 | 2.28 | 2.60E-03 |
| SYNDIG1 | 2.28 | 9.09E-05 |
| NUF2 | 2.28 | 5.56E-05 |
| CEP55 | 2.29 | 9.74E-05 |
| ANLN | 2.29 | 4.89E-04 |
| KIFC1 | 2.34 | 1.14E-03 |
| KIF2C | 2.36 | 2.65E-05 |
| ADAMDEC1 | 2.36 | 5.03E-03 |
| CDK1 | 2.38 | 9.79E-05 |
| AURKA | 2.40 | 6.32E-06 |
| SYNGR3 | 2.44 | 6.69E-04 |
| LHX2 | 2.44 | 2.06E-03 |
| PLPP4 | 2.46 | 3.57E-05 |
| FOXM1 | 2.46 | 1.14E-03 |
| CDCA8 | 2.48 | 1.96E-06 |
| CXCL10 | 2.49 | 8.52E-04 |
| INHBA | 2.51 | 1.08E-06 |
| CDC45 | 2.52 | 1.25E-03 |
| CLEC5A | 2.53 | 6.48E-07 |
| TPX2 | 2.61 | 1.28E-05 |
| ASPM | 2.62 | 9.74E-05 |
| BIRC5 | 2.64 | 1.46E-04 |
| CEMIP | 2.72 | 1.51E-04 |
| UTS2 | 2.81 | 2.75E-03 |
| NEK2 | 2.86 | 6.10E-07 |
| S100P | 2.94 | 3.01E-02 |
| GJB2 | 2.99 | 1.12E-04 |
| MMP13 | 2.99 | 5.22E-03 |
| COMP | 3.09 | 6.03E-06 |
| MMP1 | 3.17 | 1.27E-03 |
| MMP11 | 3.19 | 1.26E-07 |
| CXCL11 | 3.22 | 7.51E-04 |
| EPYC | 3.46 | 1.96E-03 |
| IBSP | 3.68 | 1.60E-04 |
| COL11A1 | 4.03 | 3.58E-06 |
| COL10A1 | 4.55 | 2.18E-12 |
