# Supplementary material for: Identification of hub genes and small molecule therapeutic drugs related to breast cancer with comprehensive bioinformatics analysis
Source: PeerJ. 2020 Sep 29;8:e9946. doi: 10.7717/peerj.9946 (PMC7556247; doi:10.7717/peerj.9946)
Supplement: Supplemental Information 14 [file peerj-08-9946-s014.docx]

| **ID** | **Log2FC** | **adj.P.Val** |
| --- | --- | --- |
| ADIPOQ | -8.69603 | 5.68E-24 |
| FABP4 | -8.12614 | 2.54E-13 |
| SCARA5 | -7.43297 | 3.11E-68 |
| RBP4 | -7.35596 | 7.48E-36 |
| LEP | -7.19628 | 3.29E-44 |
| ECRG4 | -7.14665 | 7.62E-22 |
| PLIN1 | -6.94277 | 1.99E-27 |
| CHRDL1 | -6.77048 | 3.84E-20 |
| PLIN4 | -6.55229 | 1.98E-37 |
| CFD | -6.30629 | 2.77E-23 |
| SCGB3A1 | -6.29593 | 2.37E-13 |
| OXTR | -6.24678 | 2.09E-20 |
| LPL | -6.20574 | 1.25E-15 |
| MYBPC1 | -5.88728 | 6.88E-12 |
| KRT14 | -5.83588 | 1.06E-06 |
| CIDEC | -5.75285 | 2.28E-40 |
| CD36 | -5.7258 | 6.38E-14 |
| SFRP1 | -5.6697 | 7.46E-06 |
| IRX1 | -5.65886 | 1.03E-10 |
| TIMP4 | -5.60688 | 1.76E-32 |
| KLHL13 | -5.60625 | 1.52E-16 |
| MUCL1 | -5.602 | 0.000326 |
| ZNF595 | -5.53688 | 2.19E-15 |
| PIGR | -5.535 | 3.47E-19 |
| BTNL9 | -5.52298 | 8.75E-25 |
| HOXA5 | -5.49777 | 9.97E-19 |
| WIF1 | -5.45144 | 9.77E-13 |
| APOD | -5.41977 | 1.21E-06 |
| PIP | -5.34642 | 0.00033 |
| RBP7 | -5.34121 | 8.98E-12 |
| MYH11 | -5.33293 | 6.52E-19 |
| SCGB1D2 | -5.33008 | 7.12E-05 |
| IGF1 | -5.30869 | 5.99E-10 |
| PLAC9 | -5.29943 | 1.88E-36 |
| ACACB | -5.29203 | 1.03E-35 |
| AOC3 | -5.26384 | 6.10E-21 |
| TGFBR3 | -5.25061 | 1.57E-10 |
| G0S2 | -5.24494 | 7.45E-16 |
| SEMA3G | -5.23866 | 4.12E-32 |
| SCN4B | -5.22247 | 9.69E-58 |
| ACKR1 | -5.2152 | 2.46E-13 |
| PTN | -5.19409 | 8.83E-12 |
| S100B | -5.17232 | 1.75E-14 |
| PPP1R14A | -5.17196 | 4.85E-26 |
| PDK4 | -5.16112 | 3.13E-13 |
| SCGB2A2 | -5.12373 | 0.001818 |
| FMO2 | -5.09259 | 9.69E-12 |
| FAM189A2 | -5.0908 | 1.30E-22 |
| CAVIN2 | -5.08534 | 3.85E-25 |
| MAOA | -5.08176 | 3.39E-16 |
| ZBTB16 | -5.06972 | 3.46E-21 |
| RELN | -5.06314 | 8.03E-41 |
| TRARG1 | -5.04754 | 2.49E-55 |
| OGN | -5.04612 | 1.66E-08 |
| GYG2 | -5.02262 | 3.03E-22 |
| MATN2 | -4.94328 | 6.87E-13 |
| EBF1 | -4.92752 | 1.71E-16 |
| FXYD1 | -4.87348 | 1.93E-50 |
| LRRN4CL | -4.85138 | 2.19E-35 |
| CLDN11 | -4.85004 | 1.04E-14 |
| LMOD1 | -4.84895 | 1.06E-22 |
| SYNM | -4.82872 | 3.54E-09 |
| SCD5 | -4.79184 | 1.05E-52 |
| PPP1R1A | -4.7832 | 7.45E-14 |
| GHR | -4.76881 | 9.70E-12 |
| AKR1C1 | -4.76655 | 4.95E-11 |
| KRT15 | -4.76237 | 5.71E-08 |
| HBB | -4.7588 | 2.04E-09 |
| GPIHBP1 | -4.75519 | 3.46E-68 |
| PAMR1 | -4.71156 | 4.30E-30 |
| DPT | -4.62083 | 7.43E-20 |
| FHL1 | -4.60186 | 1.12E-11 |
| INMT | -4.54346 | 2.69E-40 |
| TMEFF2 | -4.53836 | 2.97E-30 |
| CES1 | -4.49826 | 1.71E-12 |
| CLDN8 | -4.47554 | 4.60E-09 |
| EDNRB | -4.46057 | 1.74E-23 |
| TP63 | -4.45452 | 1.19E-12 |
| PI15 | -4.45168 | 1.22E-06 |
| CNN1 | -4.41123 | 2.99E-14 |
| TNS1 | -4.40706 | 2.07E-12 |
| GABRP | -4.40645 | 0.00216 |
| RBMS3 | -4.39106 | 1.34E-11 |
| ANK2 | -4.39094 | 2.14E-11 |
| ELF5 | -4.37127 | 9.23E-05 |
| ITGA7 | -4.36598 | 3.59E-37 |
| CRYAB | -4.3574 | 1.73E-07 |
| MIR205HG | -4.35313 | 2.11E-16 |
| JAM2 | -4.35006 | 6.71E-15 |
| CDKN1C | -4.32533 | 4.37E-15 |
| GPAM | -4.32182 | 6.48E-26 |
| IGFBP6 | -4.31134 | 5.19E-18 |
| ADAMTS5 | -4.30652 | 6.03E-15 |
| LOC101926960 | -4.30585 | 7.98E-37 |
| ADAMTS9-AS2 | -4.29881 | 7.60E-39 |
| COX7A1 | -4.29825 | 5.61E-15 |
| FCGBP | -4.27823 | 5.89E-09 |
| PCOLCE2 | -4.26228 | 1.27E-08 |
| KRT5 | -4.2366 | 8.83E-05 |
| KIT | -4.22633 | 1.45E-08 |
| CCDC69 | -4.20079 | 2.43E-20 |
| TMEM100 | -4.19407 | 7.12E-18 |
| GSTM5 | -4.17406 | 1.50E-40 |
| SOBP | -4.15697 | 3.06E-15 |
| CCL28 | -4.14155 | 2.02E-09 |
| RSPO3 | -4.12475 | 1.50E-10 |
| RUNX1T1 | -4.11324 | 4.25E-11 |
| PDGFD | -4.10045 | 7.70E-09 |
| SRPX | -4.08018 | 4.88E-10 |
| MME | -4.07762 | 4.26E-16 |
| CARMN | -4.05373 | 1.97E-12 |
| FAM107A | -4.00989 | 9.38E-17 |
| CX3CL1 | -3.98545 | 7.12E-11 |
| NDRG2 | -3.97387 | 1.01E-12 |
| RNF150 | -3.95044 | 9.04E-16 |
| ENPP2 | -3.93158 | 2.86E-08 |
| IL17RD | -3.92529 | 4.18E-07 |
| CRIM1-DT | -3.92069 | 1.72E-17 |
| AKR1C3 | -3.91128 | 2.18E-07 |
| TSHZ2 | -3.88741 | 6.48E-18 |
| AQP1 | -3.88708 | 4.63E-08 |
| CDO1 | -3.88401 | 2.55E-13 |
| VWF | -3.86968 | 1.04E-11 |
| ITIH5 | -3.86258 | 6.78E-45 |
| LDB2 | -3.85273 | 2.37E-14 |
| DMD | -3.84667 | 1.60E-08 |
| SOD3 | -3.84554 | 2.41E-24 |
| PEAR1 | -3.84057 | 9.27E-49 |
| ADIRF | -3.8283 | 7.74E-06 |
| ID4 | -3.81541 | 2.33E-05 |
| MEOX2 | -3.79065 | 2.17E-15 |
| ANGPTL4 | -3.78601 | 4.89E-15 |
| GNAL | -3.78291 | 7.21E-23 |
| ANKRD35 | -3.78063 | 4.29E-13 |
| MAMDC2 | -3.75804 | 3.22E-13 |
| C16orf89 | -3.75639 | 1.92E-13 |
| CLDN5 | -3.75494 | 5.54E-44 |
| PGM5 | -3.74905 | 1.39E-33 |
| FGF1 | -3.74309 | 5.38E-17 |
| LINC00993 | -3.73913 | 0.008813 |
| ABLIM3 | -3.73856 | 8.60E-13 |
| GPR146 | -3.72733 | 1.77E-42 |
| ITM2A | -3.70309 | 1.03E-09 |
| PDE2A | -3.68657 | 1.00E-44 |
| LAMB3 | -3.67866 | 1.61E-09 |
| ADH1B | -3.67586 | 2.66E-37 |
| CPED1 | -3.67051 | 1.14E-13 |
| HSD11B1-AS1 | -3.66011 | 3.40E-33 |
| CAPN3 | -3.65812 | 5.94E-17 |
| GPC3 | -3.64241 | 2.51E-14 |
| ROBO4 | -3.63933 | 4.62E-27 |
| CACHD1 | -3.63829 | 6.57E-12 |
| LIFR | -3.63096 | 1.02E-10 |
| ITGA9 | -3.62632 | 1.58E-07 |
| LTF | -3.62378 | 0.007438 |
| LIMS2 | -3.61986 | 5.10E-32 |
| HSPB2 | -3.61616 | 7.65E-36 |
| NDN | -3.61595 | 2.72E-11 |
| NES | -3.60838 | 1.33E-08 |
| ADH1C | -3.60392 | 1.05E-41 |
| LIPE | -3.5949 | 8.96E-40 |
| PLA2R1 | -3.59049 | 1.78E-17 |
| PCK1 | -3.58905 | 2.37E-25 |
| MEOX1 | -3.58338 | 1.75E-17 |
| ADRA2A | -3.58258 | 5.45E-07 |
| SEMA6D | -3.57796 | 2.05E-11 |
| TFAP2B | -3.56701 | 0.015327 |
| LAMA3 | -3.55199 | 3.80E-06 |
| BOC | -3.54815 | 2.66E-09 |
| AKR1C2 | -3.54135 | 8.96E-07 |
| APCDD1 | -3.54008 | 8.98E-10 |
| ANPEP | -3.5056 | 8.52E-07 |
| FRG1JP | -3.4734 | 1.73E-28 |
| NCALD | -3.46905 | 1.43E-09 |
| LRP2 | -3.46829 | 6.22E-05 |
| CCDC144CP | -3.4617 | 1.65E-46 |
| GAS7 | -3.46066 | 1.27E-08 |
| AKAP12 | -3.46024 | 1.39E-08 |
| TM4SF18 | -3.44753 | 1.57E-11 |
| HOXA3 | -3.43222 | 5.94E-10 |
| C14orf180 | -3.42541 | 1.71E-27 |
| DNAH3 | -3.39855 | 1.60E-59 |
| CXCL14 | -3.39264 | 0.000904 |
| SCN3A | -3.39055 | 2.05E-23 |
| SLC25A27 | -3.38541 | 3.46E-20 |
| CIDEA | -3.38352 | 1.50E-17 |
| CCL21 | -3.37236 | 4.07E-15 |
| COL14A1 | -3.3664 | 2.00E-05 |
| GALNT16 | -3.36559 | 1.40E-22 |
| HSPA12B | -3.35259 | 5.83E-17 |
| PLEKHH2 | -3.34972 | 5.27E-10 |
| CCDC3 | -3.32014 | 1.13E-16 |
| CXCL12 | -3.31368 | 8.32E-06 |
| SLIT3 | -3.31121 | 5.16E-38 |
| ACTG2 | -3.31028 | 0.001449 |
| RASL10A | -3.29964 | 1.82E-18 |
| KANK3 | -3.29616 | 6.51E-75 |
| STAT5A | -3.29515 | 7.36E-19 |
| LGR6 | -3.2829 | 1.50E-12 |
| SCRN2 | -3.27277 | 1.47E-19 |
| TCTN2 | -3.26975 | 2.08E-44 |
| AK5 | -3.26852 | 6.39E-15 |
| ADCY4 | -3.26158 | 1.37E-26 |
| CCDC8 | -3.26058 | 6.31E-22 |
| RPS11 | -3.25224 | 8.22E-40 |
| C1orf115 | -3.24798 | 2.50E-07 |
| ECM2 | -3.24785 | 1.52E-08 |
| CAV1 | -3.24779 | 9.44E-10 |
| AMT | -3.24523 | 1.24E-25 |
| TMEM132C | -3.23829 | 9.56E-47 |
| PECAM1 | -3.23041 | 4.35E-07 |
| ABI3BP | -3.22419 | 6.79E-05 |
| GNG2 | -3.21414 | 3.51E-09 |
| XDH | -3.20592 | 2.86E-05 |
| GRK3 | -3.20423 | 3.17E-16 |
| PDGFA | -3.19966 | 6.33E-15 |
| MEFV | -3.1941 | 3.37E-43 |
| BCL2 | -3.1884 | 4.08E-05 |
| CYYR1 | -3.18838 | 4.73E-15 |
| RASIP1 | -3.18411 | 1.11E-17 |
| FMOD | -3.17917 | 1.32E-05 |
| CRISPLD1 | -3.17399 | 0.000415 |
| GNG11 | -3.16933 | 1.09E-12 |
| ANKRD29 | -3.15459 | 1.29E-24 |
| PCDH18 | -3.12858 | 3.80E-06 |
| GPM6B | -3.12614 | 5.00E-06 |
| MIR100HG | -3.12538 | 4.87E-08 |
| TMTC1 | -3.12226 | 1.16E-06 |
| SVEP1 | -3.12028 | 2.61E-18 |
| ZNF528 | -3.11956 | 1.91E-25 |
| RECK | -3.11713 | 2.25E-55 |
| OPHN1 | -3.11605 | 3.30E-44 |
| ADAMTS9 | -3.11587 | 4.32E-12 |
| MIA | -3.10994 | 6.85E-05 |
| PTGDS | -3.10934 | 4.13E-10 |
| SYNPO2 | -3.10607 | 5.70E-06 |
| HOXA7 | -3.10329 | 1.00E-17 |
| STEAP4 | -3.10077 | 0.001434 |
| SLC26A3 | -3.08966 | 1.61E-08 |
| NOSTRIN | -3.08804 | 1.69E-05 |
| ANKRD20A5P | -3.0811 | 1.26E-22 |
| GGTA1P | -3.07778 | 1.90E-08 |
| PALMD | -3.07384 | 7.60E-09 |
| SORBS2 | -3.06917 | 0.000116 |
| PGM5-AS1 | -3.06807 | 1.45E-12 |
| RGMA | -3.0679 | 1.18E-07 |
| MLXIPL | -3.06493 | 4.11E-29 |
| LAMB2 | -3.06149 | 6.52E-10 |
| ACVR1C | -3.06051 | 1.92E-43 |
| CFH | -3.05771 | 3.53E-08 |
| PROS1 | -3.0574 | 2.14E-11 |
| LGALS12 | -3.05534 | 5.09E-18 |
| RERGL | -3.05034 | 9.94E-15 |
| PROM1 | -3.04621 | 0.020496 |
| HLF | -3.04419 | 4.45E-15 |
| CNRIP1 | -3.04374 | 1.89E-10 |
| FZD4 | -3.04192 | 4.65E-17 |
| HEY1 | -3.03861 | 3.99E-11 |
| TRIM29 | -3.03197 | 0.001631 |
| EGF | -3.02577 | 4.50E-08 |
| PRICKLE2 | -3.02114 | 5.75E-08 |
| ANGPTL1 | -3.02103 | 1.12E-31 |
| CLSTN2 | -3.01255 | 0.004902 |
| PLAAT3 | -3.00772 | 2.90E-06 |
| TNMD | -3.00141 | 5.01E-15 |
| EMCN | -2.99974 | 7.64E-08 |
| AIF1L | -2.99476 | 0.000149 |
| PEG3 | -2.98933 | 0.005345 |
| CXCL2 | -2.98667 | 4.66E-06 |
| TNS2 | -2.98497 | 5.07E-10 |
| SH3BGRL2 | -2.98381 | 1.51E-10 |
| DCHS1 | -2.97984 | 6.59E-09 |
| EGFLAM | -2.97955 | 5.76E-12 |
| LTBP4 | -2.97945 | 1.35E-30 |
| ROBO3 | -2.97488 | 2.63E-36 |
| LOC105379426 | -2.9643 | 5.50E-31 |
| FAXDC2 | -2.95462 | 5.54E-32 |
| FGF7P3 | -2.9386 | 9.68E-52 |
| GSTM2 | -2.93221 | 1.29E-09 |
| PGM5P2 | -2.93171 | 2.54E-33 |
| CDC42EP5 | -2.92095 | 6.33E-08 |
| ACVRL1 | -2.92041 | 3.35E-20 |
| COLCA1 | -2.91634 | 3.58E-42 |
| ZNF662 | -2.91242 | 3.63E-13 |
| CWF19L2 | -2.90255 | 2.61E-40 |
| POU6F1 | -2.89745 | 3.20E-45 |
| NAV3 | -2.89524 | 2.70E-06 |
| NR3C2 | -2.89445 | 1.14E-14 |
| LOC286437 | -2.89319 | 1.27E-21 |
| EVA1C | -2.8864 | 6.06E-08 |
| MYOM2 | -2.88609 | 3.35E-20 |
| CITED1 | -2.88186 | 3.52E-10 |
| MST1 | -2.88058 | 2.00E-10 |
| SPARCL1 | -2.86981 | 0.001155 |
| MAOB | -2.86785 | 0.002227 |
| JAM3 | -2.86487 | 1.99E-09 |
| EHD2 | -2.86089 | 1.14E-16 |
| PDE9A | -2.85863 | 8.74E-08 |
| ARID5A | -2.85639 | 2.88E-30 |
| SHE | -2.84743 | 2.52E-18 |
| PKDCC | -2.84716 | 2.19E-14 |
| CBX7 | -2.84548 | 8.18E-17 |
| RTN1 | -2.83396 | 1.60E-05 |
| WDR86 | -2.83055 | 2.34E-39 |
| PRIMA1 | -2.82962 | 7.99E-11 |
| MOB3B | -2.82706 | 3.93E-07 |
| C8orf88 | -2.82581 | 2.98E-12 |
| SIK2 | -2.82064 | 2.14E-27 |
| CD248 | -2.81831 | 6.93E-14 |
| PLSCR4 | -2.81102 | 5.96E-08 |
| PCDH19 | -2.81079 | 1.13E-13 |
| RARRES2 | -2.80447 | 7.49E-06 |
| AGTR1 | -2.79966 | 0.000236 |
| MYO15B | -2.78667 | 9.05E-09 |
| MCAM | -2.78571 | 1.25E-10 |
| TENM2 | -2.78324 | 8.62E-09 |
| PTPRB | -2.78272 | 8.01E-08 |
| EGFL7 | -2.77953 | 4.47E-15 |
| GNAI1 | -2.76874 | 1.71E-07 |
| PGR | -2.76708 | 0.01785 |
| SNX21 | -2.76546 | 1.66E-13 |
| LHX6 | -2.76409 | 1.42E-17 |
| MEIS2 | -2.7629 | 4.28E-07 |
| SLC27A1 | -2.76081 | 8.52E-18 |
| ZNF667 | -2.7601 | 3.07E-08 |
| PPM1F | -2.75715 | 9.72E-24 |
| CA4 | -2.75371 | 1.09E-43 |
| CCM2L | -2.75329 | 3.19E-14 |
| EFEMP1 | -2.74851 | 6.41E-05 |
| BBOX1 | -2.74692 | 0.001702 |
| LINC00312 | -2.74631 | 4.42E-07 |
| VIPR1 | -2.74576 | 7.90E-11 |
| CPLX1 | -2.74366 | 5.31E-07 |
| ARHGEF28 | -2.73994 | 2.36E-19 |
| MFGE8 | -2.73754 | 3.97E-09 |
| MMRN2 | -2.72918 | 6.38E-13 |
| FAM13A-AS1 | -2.72421 | 1.20E-13 |
| PCNX1 | -2.72034 | 7.95E-22 |
| AR | -2.72006 | 0.029001 |
| PPARG | -2.71434 | 1.33E-07 |
| MTURN | -2.70848 | 4.14E-10 |
| LINC01279 | -2.70822 | 0.001711 |
| PTGER3 | -2.70656 | 2.68E-05 |
| TMEM273 | -2.70154 | 1.49E-10 |
| PGGHG | -2.70148 | 0.000213 |
| WLS | -2.69979 | 3.14E-05 |
| MAP2 | -2.69687 | 0.000185 |
| PCSK5 | -2.69496 | 1.24E-07 |
| FAM3B | -2.68883 | 0.004391 |
| CSRNP3 | -2.68814 | 1.66E-12 |
| ZNF471 | -2.68665 | 8.28E-18 |
| GFRA1 | -2.67554 | 0.006963 |
| MRAS | -2.67528 | 1.42E-09 |
| SLC28A3 | -2.67528 | 0.000559 |
| IRS1 | -2.67375 | 1.64E-07 |
| ADAMTS15 | -2.67029 | 0.00129 |
| ME3 | -2.66292 | 1.12E-08 |
| GAS6 | -2.66253 | 1.08E-08 |
| EZH1 | -2.66176 | 3.11E-27 |
| COBL | -2.65975 | 1.71E-05 |
| PTGIS | -2.65722 | 1.22E-05 |
| ACCS | -2.65613 | 2.24E-19 |
| TFPI | -2.6537 | 6.00E-06 |
| CX3CR1 | -2.65007 | 0.000916 |
| MYLK | -2.64829 | 4.69E-06 |
| SLC13A2 | -2.64815 | 8.02E-19 |
| SLC16A7 | -2.64729 | 6.25E-08 |
| LINC02504 | -2.63888 | 5.67E-33 |
| GSN | -2.63499 | 6.28E-19 |
| CASP17P | -2.63235 | 1.70E-16 |
| CAVIN1 | -2.62933 | 4.24E-14 |
| CFAP70 | -2.62292 | 2.38E-05 |
| NAT8L | -2.62266 | 4.33E-27 |
| ADGRL4 | -2.61661 | 4.85E-07 |
| FXYD6 | -2.61152 | 2.30E-07 |
| CTSF | -2.60994 | 7.22E-13 |
| GIMAP1 | -2.60469 | 3.00E-09 |
| FAM13A | -2.60433 | 6.43E-14 |
| MEG3 | -2.60039 | 3.84E-06 |
| SYNPO | -2.59815 | 1.57E-13 |
| CGNL1 | -2.59668 | 2.97E-05 |
| TCF7L2 | -2.58961 | 3.31E-18 |
| AMIGO2 | -2.58382 | 9.99E-05 |
| FAM149A | -2.583 | 1.62E-11 |
| CD34 | -2.58054 | 5.69E-17 |
| NOTCH4 | -2.57661 | 4.04E-25 |
| SPX | -2.57371 | 6.92E-10 |
| OLFML2A | -2.57352 | 5.91E-08 |
| METTL7A | -2.57217 | 1.83E-08 |
| PYROXD2 | -2.56786 | 2.85E-10 |
| ALDH1L1 | -2.56736 | 6.90E-57 |
| TPM2 | -2.56687 | 2.03E-07 |
| IGSF10 | -2.56189 | 1.10E-16 |
| OLFM4 | -2.55505 | 0.000789 |
| THSD4 | -2.55327 | 0.003721 |
| ARHGEF37 | -2.54882 | 6.04E-06 |
| GPX3 | -2.54447 | 0.000899 |
| FAHD2CP | -2.53279 | 2.03E-23 |
| CPE | -2.52712 | 2.87E-05 |
| NATD1 | -2.52528 | 1.94E-13 |
| RASSF6 | -2.52 | 3.46E-05 |
| SETBP1 | -2.51894 | 3.20E-05 |
| TLE2 | -2.51783 | 1.32E-06 |
| LINC00266-1 | -2.51632 | 4.24E-26 |
| RHOU | -2.51551 | 1.45E-07 |
| GRAMD1C | -2.51524 | 8.13E-08 |
| SLPI | -2.51216 | 0.019369 |
| MAML2 | -2.51132 | 2.20E-08 |
| NAV2 | -2.5085 | 5.56E-06 |
| STAC2 | -2.50728 | 4.41E-09 |
| AZGP1 | -2.50496 | 0.025328 |
| GSTM1 | -2.50011 | 1.41E-11 |
| JCHAIN | -2.4996 | 0.019381 |
| LOXL4 | -2.49616 | 0.000161 |
| IQCN | -2.49401 | 1.12E-20 |
| PDGFRA | -2.49173 | 0.000425 |
| EFEMP2 | -2.48992 | 1.62E-07 |
| DDR2 | -2.48855 | 2.29E-08 |
| LOC100505874 | -2.485 | 1.39E-06 |
| ARAP3 | -2.484 | 4.12E-13 |
| P3H2 | -2.48337 | 0.000116 |
| PPL | -2.47949 | 7.14E-07 |
| GPAT3 | -2.47276 | 4.97E-07 |
| DCLK1 | -2.47091 | 2.70E-05 |
| THSD7A | -2.46759 | 1.37E-11 |
| ISM1 | -2.46275 | 3.29E-05 |
| CTBP1-DT | -2.46271 | 1.89E-19 |
| LAMA4 | -2.46009 | 2.58E-06 |
| DEPP1 | -2.45931 | 1.58E-06 |
| CCDC80 | -2.45631 | 0.000396 |
| DKK3 | -2.45396 | 4.73E-05 |
| IGFBP4 | -2.45217 | 9.90E-06 |
| KLF15 | -2.45137 | 1.62E-27 |
| ABCA5 | -2.44918 | 3.58E-10 |
| LINC00663 | -2.44837 | 1.22E-14 |
| SLC6A14 | -2.44618 | 0.011929 |
| RPL23AP32 | -2.44596 | 2.02E-19 |
| FAM228B | -2.444 | 1.13E-11 |
| ZNF462 | -2.44304 | 2.47E-05 |
| FRG1BP | -2.4361 | 2.64E-27 |
| TMEM241 | -2.43087 | 5.52E-31 |
| SMOC2 | -2.42874 | 0.001661 |
| ARHGAP6 | -2.42665 | 6.27E-09 |
| TRMT9B | -2.4211 | 9.22E-19 |
| FOXC1 | -2.41968 | 0.025801 |
| GULP1 | -2.41802 | 6.48E-05 |
| ADAM33 | -2.41602 | 8.22E-24 |
| LARP6 | -2.41513 | 1.62E-08 |
| APLNR | -2.41355 | 7.01E-10 |
| STC2 | -2.40694 | 0.004627 |
| GLDN | -2.40665 | 9.42E-10 |
| TNS4 | -2.40658 | 1.09E-07 |
| A2M | -2.40478 | 0.008126 |
| TNN | -2.40471 | 4.67E-08 |
| NPR1 | -2.40301 | 5.22E-45 |
| ZNF204P | -2.40196 | 2.94E-14 |
| FAM162B | -2.39978 | 3.64E-18 |
| FRZB | -2.39956 | 0.00016 |
| ACSS2 | -2.39933 | 7.00E-15 |
| ADGRF5 | -2.399 | 4.80E-06 |
| ARL4A | -2.39243 | 7.40E-13 |
| CLIP4 | -2.3907 | 7.67E-06 |
| LHFPL6 | -2.38871 | 1.00E-06 |
| SIDT2 | -2.38306 | 6.52E-13 |
| VSIR | -2.3824 | 1.18E-16 |
| ANTXR2 | -2.38184 | 4.67E-08 |
| VPS51 | -2.37712 | 1.71E-27 |
| GPRASP1 | -2.37591 | 6.78E-13 |
| MRGPRF | -2.37486 | 8.60E-12 |
| NAP1L5 | -2.37273 | 1.86E-06 |
| LOC102724814 | -2.37259 | 3.20E-16 |
| MAP1A | -2.36553 | 1.01E-09 |
| CSTA | -2.36522 | 0.005492 |
| FBLN1 | -2.36445 | 9.63E-06 |
| SPTBN1 | -2.35972 | 8.20E-25 |
| SHC2 | -2.35647 | 1.32E-07 |
| B3GALT5 | -2.35529 | 6.74E-07 |
| ZNF423 | -2.3539 | 1.81E-10 |
| ABCA9 | -2.3538 | 2.66E-26 |
| LINC00173 | -2.3536 | 0.000543 |
| JADE1 | -2.34573 | 7.46E-21 |
| 1-Mar | -2.34333 | 1.18E-08 |
| KIF26A | -2.34236 | 7.59E-13 |
| RGL1 | -2.3423 | 3.99E-12 |
| SMAD9 | -2.33957 | 4.07E-06 |
| NISCH | -2.33883 | 3.62E-19 |
| OPRPN | -2.33676 | 3.71E-09 |
| FGFR1 | -2.33521 | 1.40E-07 |
| DSEL | -2.33276 | 1.56E-07 |
| LINC01697 | -2.33127 | 2.39E-31 |
| NRN1 | -2.32932 | 0.000186 |
| STXBP1 | -2.32728 | 3.21E-06 |
| DPY19L2P2 | -2.32703 | 3.65E-16 |
| CPXM1 | -2.32499 | 3.62E-05 |
| CIRBP | -2.32488 | 5.45E-12 |
| IL11RA | -2.32465 | 2.60E-23 |
| GIMAP8 | -2.3231 | 5.34E-08 |
| FGD5 | -2.32303 | 1.55E-08 |
| NFIX | -2.3143 | 5.85E-09 |
| AMOTL1 | -2.30649 | 1.64E-09 |
| TMEM47 | -2.30627 | 0.00037 |
| TCF7L1 | -2.306 | 0.002238 |
| ALDOC | -2.30215 | 3.74E-05 |
| ZBTB4 | -2.3021 | 4.17E-21 |
| DGAT2 | -2.3017 | 0.000702 |
| KCNN4 | -2.30079 | 0.00173 |
| SHANK3 | -2.2995 | 5.36E-09 |
| GLYAT | -2.29941 | 2.37E-25 |
| ARHGEF40 | -2.29878 | 7.61E-10 |
| PALM | -2.29682 | 1.50E-17 |
| BEND5 | -2.29533 | 8.86E-15 |
| PLLP | -2.29518 | 7.91E-07 |
| TAC1 | -2.28825 | 8.10E-14 |
| SNHG27 | -2.27934 | 6.62E-22 |
| TWIST1 | -2.27699 | 1.47E-05 |
| PRELP | -2.27597 | 3.59E-07 |
| TBX15 | -2.27205 | 2.03E-23 |
| COL7A1 | -2.2713 | 1.97E-11 |
| PDLIM4 | -2.27106 | 1.71E-12 |
| ND6 | -2.26523 | 6.13E-16 |
| MMP16 | -2.26379 | 2.60E-05 |
| MID1 | -2.25984 | 0.000535 |
| HYI | -2.25939 | 6.75E-19 |
| KLHL21 | -2.25873 | 1.60E-13 |
| CEP68 | -2.25696 | 5.78E-16 |
| ACADS | -2.25661 | 7.28E-34 |
| RASGRF2 | -2.25633 | 5.58E-11 |
| ECHDC2 | -2.25615 | 6.08E-12 |
| MFAP4 | -2.25608 | 0.000221 |
| IRX2 | -2.25428 | 0.006661 |
| MYL9 | -2.25272 | 0.000333 |
| SLC7A2 | -2.25199 | 0.019364 |
| ACADSB | -2.25169 | 0.000706 |
| CALML3 | -2.25134 | 1.34E-06 |
| RCAN2 | -2.24801 | 1.54E-07 |
| FCRL2 | -2.24436 | 4.33E-27 |
| KANK4 | -2.24348 | 0.008473 |
| B4GALT6 | -2.24328 | 1.47E-11 |
| SLC7A10 | -2.24214 | 3.80E-20 |
| PLEKHH1 | -2.23797 | 1.07E-05 |
| CRACR2B | -2.23311 | 0.000304 |
| ALDH1A1 | -2.23184 | 0.000544 |
| CRY2 | -2.23164 | 1.18E-17 |
| SELENOP | -2.2267 | 0.003377 |
| ZNF395 | -2.21891 | 6.13E-10 |
| ANGPT1 | -2.21884 | 0.000168 |
| RGS5 | -2.21214 | 0.000373 |
| ARHGEF6 | -2.21164 | 1.03E-05 |
| IQCH-AS1 | -2.2039 | 7.87E-11 |
| TMEM198B | -2.20294 | 2.54E-22 |
| COL21A1 | -2.20012 | 3.75E-11 |
| ZCCHC24 | -2.19876 | 5.02E-05 |
| ODF3B | -2.19665 | 7.59E-06 |
| FBXO31 | -2.19237 | 6.84E-15 |
| AIFM2 | -2.18926 | 7.61E-14 |
| DAAM2 | -2.18348 | 7.64E-10 |
| SOCS2 | -2.1821 | 0.000676 |
| COQ8A | -2.17624 | 2.15E-12 |
| SLC19A3 | -2.17613 | 3.85E-25 |
| NFATC1 | -2.17113 | 2.00E-05 |
| LYRM9 | -2.17046 | 1.53E-11 |
| TTC28 | -2.16846 | 4.67E-10 |
| ECSCR | -2.16811 | 7.92E-28 |
| SOX5 | -2.16731 | 3.18E-16 |
| MELTF | -2.16502 | 0.000368 |
| PLCB1 | -2.16011 | 0.001751 |
| TWIST2 | -2.15778 | 6.52E-06 |
| EPB41L4A-AS1 | -2.15663 | 1.51E-10 |
| PLPP1 | -2.15625 | 2.14E-12 |
| LPAR1 | -2.15325 | 0.000241 |
| ZNF747 | -2.14896 | 1.02E-26 |
| CFAP410 | -2.14692 | 7.01E-41 |
| ATP1A2 | -2.14383 | 1.67E-19 |
| GSTT1 | -2.14178 | 0.01371 |
| DLC1 | -2.14111 | 8.37E-05 |
| MAGI2 | -2.1406 | 2.08E-06 |
| ADGRL3 | -2.14021 | 3.27E-09 |
| HOXD8 | -2.13997 | 3.48E-06 |
| TRHDE-AS1 | -2.13939 | 1.72E-13 |
| PDGFRL | -2.13702 | 0.001427 |
| LAMC2 | -2.136 | 0.000573 |
| MT1M | -2.13466 | 2.60E-10 |
| C1orf116 | -2.13162 | 0.004349 |
| DEFB124 | -2.12973 | 3.14E-14 |
| FLJ20021 | -2.12859 | 8.13E-18 |
| CPS1 | -2.12794 | 9.49E-15 |
| B4GAT1 | -2.12663 | 1.21E-19 |
| ALDH2 | -2.12365 | 0.00039 |
| FAT3 | -2.12361 | 6.37E-08 |
| GYPC | -2.12142 | 6.75E-12 |
| CDH5 | -2.12042 | 9.92E-11 |
| TTC38 | -2.11956 | 4.71E-21 |
| MGLL | -2.11955 | 0.000322 |
| MTMR9LP | -2.11951 | 8.04E-36 |
| LRRC17 | -2.1188 | 0.025251 |
| TEF | -2.11831 | 2.21E-14 |
| SF3A2 | -2.11782 | 1.18E-15 |
| INSR | -2.11698 | 1.41E-07 |
| MAGI1 | -2.11659 | 8.57E-06 |
| MAP4 | -2.11558 | 3.56E-18 |
| BCAM | -2.11545 | 4.83E-06 |
| CDKN2C | -2.11521 | 3.70E-06 |
| PC | -2.11318 | 3.40E-08 |
| TXNIP | -2.1115 | 0.001193 |
| ABLIM1 | -2.11075 | 3.12E-08 |
| ADHFE1 | -2.10713 | 2.79E-09 |
| MRC1 | -2.1043 | 0.002456 |
| RGS2 | -2.10072 | 0.000552 |
| ISLR | -2.09973 | 0.002909 |
| TMEM178A | -2.09735 | 3.59E-06 |
| KLHL31 | -2.09578 | 1.34E-12 |
| ZEB2 | -2.09495 | 4.89E-07 |
| SYCE1L | -2.09366 | 1.60E-16 |
| SPRY2 | -2.09316 | 4.40E-05 |
| CARD10 | -2.09278 | 0.000108 |
| THRB | -2.09192 | 6.60E-06 |
| BEX2 | -2.089 | 0.003246 |
| LOC728392 | -2.08862 | 1.26E-06 |
| CA3 | -2.0881 | 1.02E-21 |
| ARRB1 | -2.08562 | 4.09E-13 |
| RUNDC3B | -2.08486 | 6.54E-18 |
| DICER1-AS1 | -2.08323 | 2.36E-17 |
| RAB11B-AS1 | -2.08319 | 3.22E-19 |
| GOLGA8N | -2.08293 | 3.79E-13 |
| RAI2 | -2.0816 | 0.000412 |
| TK2 | -2.08158 | 4.06E-16 |
| ABTB1 | -2.08138 | 1.13E-22 |
| MESP1 | -2.07656 | 4.31E-06 |
| PLAAT5 | -2.0749 | 5.27E-06 |
| TLE4 | -2.07229 | 1.45E-07 |
| ESAM | -2.07041 | 3.30E-14 |
| TPPP3 | -2.06978 | 1.97E-05 |
| TGFB1I1 | -2.06951 | 0.000252 |
| PHF1 | -2.06892 | 1.15E-25 |
| SNX1 | -2.06866 | 9.88E-11 |
| ABHD14B | -2.06531 | 7.75E-13 |
| HRCT1 | -2.06526 | 2.11E-07 |
| RPL27A | -2.06367 | 6.21E-28 |
| RSPO1 | -2.06172 | 4.69E-21 |
| CYBRD1 | -2.06148 | 5.57E-05 |
| FOXO1 | -2.06041 | 2.60E-11 |
| TCEAL7 | -2.05948 | 3.54E-08 |
| FMO1 | -2.05764 | 0.000906 |
| CMYA5 | -2.05706 | 7.84E-05 |
| ACSS3 | -2.05518 | 0.000448 |
| DHDDS | -2.05429 | 8.66E-16 |
| CPAMD8 | -2.05174 | 3.85E-05 |
| TMEM101 | -2.0514 | 4.31E-07 |
| IRS2 | -2.05041 | 0.000151 |
| FBXL17 | -2.0472 | 3.03E-15 |
| CAV2 | -2.04641 | 5.60E-05 |
| WDFY2 | -2.04562 | 5.21E-15 |
| GIMAP7 | -2.04481 | 3.25E-05 |
| DTWD1 | -2.04365 | 1.91E-07 |
| CACNA2D1 | -2.04234 | 7.60E-07 |
| SDK2 | -2.04027 | 9.61E-06 |
| COL16A1 | -2.03684 | 1.96E-05 |
| MSX1 | -2.03582 | 2.28E-10 |
| IGIP | -2.03439 | 6.29E-10 |
| MYCT1 | -2.03213 | 3.33E-25 |
| DPYSL2 | -2.03202 | 1.18E-09 |
| PFKFB3 | -2.03107 | 4.55E-07 |
| MSRB3 | -2.03014 | 0.001207 |
| MEST | -2.02912 | 0.01403 |
| CLU | -2.0287 | 0.000887 |
| TRO | -2.02498 | 2.35E-05 |
| ANGPTL2 | -2.02443 | 0.002722 |
| AKT3 | -2.02201 | 3.65E-07 |
| SPATA18 | -2.02131 | 4.68E-05 |
| LINC00893 | -2.02071 | 6.79E-17 |
| HOXD9 | -2.02061 | 7.05E-34 |
| SNHG19 | -2.01997 | 1.48E-08 |
| BIN1 | -2.0199 | 7.62E-10 |
| APH1B | -2.01938 | 6.70E-07 |
| KANK1 | -2.01765 | 2.31E-05 |
| SEMA6A | -2.01542 | 5.32E-05 |
| PPM1L | -2.01497 | 5.74E-07 |
| ARL15 | -2.01365 | 9.17E-06 |
| PLAT | -2.01317 | 0.016733 |
| PDZRN3 | -2.01277 | 7.81E-05 |
| TINAGL1 | -2.01245 | 2.83E-13 |
| CTDSP1 | -2.0119 | 3.06E-17 |
| AHNAK | -2.01168 | 2.50E-08 |
| TLN2 | -2.01069 | 1.29E-16 |
| TAT | -2.00969 | 7.00E-21 |
| NAB1 | -2.00967 | 2.37E-08 |
| SERPING1 | -2.00949 | 0.009074 |
| LOC100506990 | -2.00764 | 2.25E-06 |
| PAQR7 | -2.00498 | 8.96E-16 |
| TRIM68 | -1.99978 | 2.53E-05 |
| N4BP2L1 | -1.99895 | 1.60E-05 |
| RASD1 | -1.99544 | 0.004487 |
| STAT5B | -1.99543 | 4.43E-12 |
| LENG8 | -1.99495 | 7.26E-09 |
| POU2AF1 | -1.99477 | 0.021275 |
| MYOM1 | -1.99383 | 2.15E-23 |
| ZNF358 | -1.99301 | 2.33E-21 |
| KIZ | -1.98953 | 7.43E-18 |
| TBC1D4 | -1.98891 | 9.91E-12 |
| SALL2 | -1.98776 | 9.05E-07 |
| GLIDR | -1.98633 | 6.88E-17 |
| MMD | -1.98285 | 1.49E-10 |
| STOX2 | -1.98268 | 2.34E-08 |
| PPP1R16B | -1.98114 | 5.45E-12 |
| MFNG | -1.98106 | 3.08E-12 |
| MAPKBP1 | -1.98101 | 2.91E-18 |
| GBP2 | -1.97989 | 9.59E-06 |
| ZBTB46 | -1.97977 | 1.92E-12 |
| CRYBG3 | -1.9792 | 6.88E-08 |
| ADRB1 | -1.97896 | 0.000414 |
| MECOM | -1.97877 | 1.54E-06 |
| ZBTB20 | -1.97545 | 2.93E-05 |
| EBF3 | -1.97321 | 5.95E-08 |
| TIMP3 | -1.96993 | 0.008187 |
| PNPLA7 | -1.9696 | 1.28E-24 |
| WFDC2 | -1.96933 | 0.011234 |
| RALGAPA2 | -1.96841 | 2.32E-05 |
| CFAP69 | -1.96838 | 1.10E-05 |
| IL17B | -1.96696 | 1.12E-08 |
| COPZ2 | -1.96407 | 0.000264 |
| GUCY1A1 | -1.96032 | 0.00021 |
| HYMAI | -1.95951 | 1.15E-09 |
| COL27A1 | -1.9592 | 0.000729 |
| MRVI1 | -1.95883 | 1.34E-05 |
| ZDHHC2 | -1.95863 | 0.000479 |
| LINC02035 | -1.95813 | 9.39E-14 |
| FAM219B | -1.95542 | 1.89E-16 |
| PLCL2 | -1.95424 | 1.55E-05 |
| GPD1 | -1.95338 | 8.96E-40 |
| JAG2 | -1.95267 | 1.25E-12 |
| FKSG49 | -1.95048 | 2.39E-20 |
| DST | -1.94978 | 6.72E-10 |
| DEFB132 | -1.9491 | 4.18E-16 |
| FCGRT | -1.9468 | 1.11E-05 |
| CYGB | -1.94375 | 4.22E-14 |
| SOD2 | -1.94268 | 1.31E-07 |
| RASEF | -1.94088 | 9.23E-09 |
| LOC101927166 | -1.94008 | 1.78E-11 |
| WDTC1 | -1.93996 | 8.95E-26 |
| LVRN | -1.93851 | 9.55E-21 |
| LIMCH1 | -1.93684 | 0.001614 |
| ETFB | -1.93663 | 3.59E-09 |
| KANK2 | -1.93513 | 8.69E-06 |
| PPP1R12B | -1.93115 | 3.20E-08 |
| MZF1 | -1.92991 | 1.88E-11 |
| BOK | -1.92962 | 2.40E-07 |
| MAP3K20 | -1.92907 | 1.35E-11 |
| ICAM2 | -1.92774 | 1.37E-05 |
| ANKDD1A | -1.92657 | 1.27E-13 |
| C3orf18 | -1.9258 | 1.88E-11 |
| TRPC1 | -1.92544 | 8.12E-09 |
| ZNF436-AS1 | -1.92515 | 2.51E-17 |
| FAT2 | -1.92432 | 4.03E-06 |
| ADAMTS1 | -1.92386 | 0.002826 |
| BDKRB2 | -1.92033 | 4.63E-09 |
| MEGF8 | -1.91903 | 3.88E-14 |
| NFIB | -1.91874 | 8.96E-06 |
| SMARCD3 | -1.91795 | 3.66E-08 |
| C3 | -1.91794 | 0.001586 |
| MUC3A | -1.91763 | 8.96E-40 |
| PTCH1 | -1.91734 | 0.000421 |
| GPLD1 | -1.91681 | 1.47E-07 |
| D2HGDH | -1.91662 | 1.64E-10 |
| NFATC4 | -1.91606 | 1.21E-09 |
| NTN4 | -1.91557 | 0.028767 |
| ARHGAP19 | -1.91415 | 1.16E-14 |
| PTPN21 | -1.91305 | 1.42E-05 |
| DENND2B | -1.91189 | 9.54E-10 |
| NUAK1 | -1.91055 | 0.000102 |
| CCDC178 | -1.9087 | 1.86E-27 |
| TMEM91 | -1.90805 | 5.57E-16 |
| GRK5 | -1.9072 | 1.16E-06 |
| FERMT2 | -1.90479 | 1.67E-05 |
| GDPD5 | -1.90476 | 2.33E-15 |
| JCAD | -1.90428 | 6.70E-06 |
| POMT1 | -1.90419 | 7.54E-12 |
| GIMAP6 | -1.90352 | 0.000245 |
| FOLR2 | -1.89985 | 2.05E-08 |
| ELMOD3 | -1.89955 | 5.62E-16 |
| CEP85L | -1.89759 | 1.13E-08 |
| NRG2 | -1.89658 | 5.54E-33 |
| PLEKHA4 | -1.89598 | 2.33E-15 |
| MEDAG | -1.89586 | 3.10E-08 |
| BHLHE41 | -1.89561 | 0.000488 |
| CTSG | -1.89544 | 1.02E-08 |
| CELF2 | -1.8938 | 0.001032 |
| MSRA | -1.8931 | 1.06E-09 |
| LAMA2 | -1.89093 | 0.000264 |
| LOC283788 | -1.88896 | 5.81E-06 |
| WASF2 | -1.88896 | 2.02E-20 |
| TSPYL2 | -1.88837 | 9.16E-18 |
| RPS6KA2 | -1.88805 | 1.04E-08 |
| TLCD2 | -1.88784 | 7.81E-07 |
| PINK1 | -1.88775 | 4.27E-16 |
| SATB1 | -1.88503 | 0.000333 |
| ITPR1 | -1.88364 | 5.82E-05 |
| DEFB1 | -1.88114 | 0.022415 |
| FZD7 | -1.87888 | 2.77E-05 |
| RETREG3 | -1.87837 | 6.46E-13 |
| BLCAP | -1.87708 | 5.96E-16 |
| ZC3H6 | -1.87514 | 9.03E-10 |
| INHBB | -1.87356 | 0.003227 |
| CDK10 | -1.87105 | 2.30E-14 |
| PHLPP1 | -1.87025 | 8.55E-05 |
| SORBS3 | -1.86941 | 6.68E-21 |
| TSPAN7 | -1.86801 | 9.88E-08 |
| PTGES | -1.86592 | 0.002558 |
| RIMKLB | -1.86585 | 1.60E-08 |
| HDAC5 | -1.86404 | 6.13E-14 |
| OLFML1 | -1.85951 | 0.000237 |
| PNPLA2 | -1.85935 | 1.48E-27 |
| EGFR | -1.85867 | 0.018071 |
| LY75 | -1.85827 | 0.00382 |
| PCBP4 | -1.8581 | 8.15E-12 |
| ALKAL2 | -1.85681 | 0.000225 |
| FAM126A | -1.85486 | 3.41E-06 |
| ABCA3 | -1.854 | 4.10E-05 |
| SERPINI1 | -1.85095 | 0.000126 |
| FAM20C | -1.84994 | 7.00E-07 |
| TUBG2 | -1.84941 | 3.72E-06 |
| PLXNA4 | -1.8481 | 3.12E-17 |
| HOTAIRM1 | -1.84798 | 1.30E-07 |
| EPAS1 | -1.84696 | 1.02E-09 |
| KLHL3 | -1.84545 | 7.00E-07 |
| CYP1A2 | -1.84527 | 7.29E-37 |
| PBXIP1 | -1.84428 | 4.14E-11 |
| SESN3 | -1.84368 | 4.52E-05 |
| WNK2 | -1.84226 | 7.70E-08 |
| RETREG1 | -1.84158 | 0.00189 |
| VAMP2 | -1.84152 | 3.23E-12 |
| DIPK1A | -1.83958 | 0.000487 |
| TPCN1 | -1.83924 | 4.61E-08 |
| FGFR2 | -1.83727 | 0.025302 |
| SNRNP70 | -1.83434 | 7.49E-16 |
| ASB1 | -1.83394 | 9.26E-17 |
| FRA10AC1 | -1.83285 | 2.25E-18 |
| PHYHD1 | -1.83078 | 0.003164 |
| KCNJ2 | -1.83063 | 2.06E-05 |
| TCF4 | -1.82844 | 0.003243 |
| ABCA6 | -1.82758 | 2.25E-08 |
| DHRS3 | -1.82492 | 1.02E-06 |
| BACE1 | -1.82417 | 1.21E-09 |
| LINC01315 | -1.82146 | 0.000193 |
| NPFF | -1.82146 | 7.11E-29 |
| RBPMS2 | -1.8209 | 8.72E-08 |
| MXRA8 | -1.818 | 0.002215 |
| TUBB2B | -1.81793 | 0.003077 |
| PLA2G4C | -1.81762 | 2.24E-05 |
| AVPR1A | -1.81594 | 3.22E-07 |
| NIBAN1 | -1.81517 | 0.000369 |
| LTBP2 | -1.81516 | 2.40E-05 |
| TBX3 | -1.81429 | 0.000107 |
| GNB5 | -1.81375 | 8.48E-09 |
| CPXM2 | -1.81266 | 7.85E-06 |
| LHCGR | -1.81095 | 4.13E-20 |
| CLEC14A | -1.80981 | 7.84E-13 |
| DIPK1B | -1.80869 | 1.83E-07 |
| KCNAB1 | -1.80807 | 1.31E-05 |
| HCFC2 | -1.80718 | 4.08E-17 |
| IGF1R | -1.80495 | 0.012799 |
| PADI2 | -1.80414 | 0.01554 |
| AHI1 | -1.80065 | 1.82E-07 |
| CLIP3 | -1.80036 | 3.22E-07 |
| GAS1RR | -1.80029 | 2.47E-24 |
| TACC2 | -1.79836 | 3.69E-08 |
| FAM13C | -1.7977 | 1.02E-06 |
| KLK7 | -1.79767 | 0.016511 |
| TMEM67 | -1.79729 | 1.93E-08 |
| CCBE1 | -1.79668 | 1.99E-09 |
| KIF13B | -1.79637 | 6.11E-05 |
| MED16 | -1.79483 | 7.94E-09 |
| LINC01140 | -1.79388 | 6.49E-06 |
| PRRG3 | -1.79248 | 1.01E-18 |
| ADH4 | -1.79224 | 3.40E-33 |
| HOXA4 | -1.79075 | 3.25E-15 |
| CYP4F12 | -1.78939 | 1.76E-15 |
| ACADVL | -1.78702 | 3.44E-12 |
| TAGLN | -1.78591 | 0.017481 |
| MXRA7 | -1.78456 | 4.38E-06 |
| TNFRSF1B | -1.78245 | 6.37E-06 |
| FOXO4 | -1.78144 | 7.51E-32 |
| LOC102724851 | -1.78085 | 0.000102 |
| CHKB | -1.77729 | 4.17E-18 |
| NMB | -1.77683 | 5.41E-05 |
| EMILIN2 | -1.77645 | 0.000195 |
| RGCC | -1.77597 | 0.000648 |
| PRINS | -1.77348 | 2.90E-34 |
| PELI2 | -1.77093 | 4.14E-09 |
| STK11 | -1.77084 | 1.10E-12 |
| PDE5A | -1.77062 | 0.000605 |
| MPPED2 | -1.76964 | 4.79E-07 |
| SGSM2 | -1.76949 | 1.02E-11 |
| PPARA | -1.7694 | 0.000851 |
| MAF | -1.7683 | 0.000201 |
| INPP1 | -1.76806 | 1.27E-06 |
| CLMP | -1.76595 | 0.003511 |
| CDCA7L | -1.76509 | 0.001275 |
| CKB | -1.76464 | 4.39E-05 |
| BCL6 | -1.76231 | 3.16E-08 |
| UBXN1 | -1.76198 | 4.42E-21 |
| HSPG2 | -1.76148 | 3.09E-06 |
| ECHDC3 | -1.76091 | 0.000152 |
| ACTA2 | -1.7595 | 0.016765 |
| TMEM140 | -1.75942 | 7.71E-07 |
| MBD3 | -1.75854 | 1.44E-12 |
| LOC100996740 | -1.7576 | 0.001171 |
| ZNF236 | -1.75663 | 6.61E-25 |
| PPFIBP1 | -1.75399 | 0.000268 |
| FBLN7 | -1.75027 | 1.63E-06 |
| ZNF677 | -1.74973 | 4.12E-06 |
| PAPLN | -1.74965 | 0.000701 |
| IL27RA | -1.74956 | 0.009333 |
| HGF | -1.74933 | 1.11E-09 |
| ZEB1 | -1.74669 | 0.010426 |
| MYL5 | -1.74648 | 7.82E-06 |
| FOXP1 | -1.74619 | 4.30E-06 |
| INAFM1 | -1.74579 | 5.87E-11 |
| PELI3 | -1.74539 | 1.57E-16 |
| TMEM161B-AS1 | -1.74483 | 2.44E-21 |
| GIPC2 | -1.74469 | 6.90E-23 |
| C18orf54 | -1.74425 | 8.86E-07 |
| ALDH3A2 | -1.74404 | 9.67E-07 |
| SLC22A17 | -1.74251 | 6.25E-08 |
| ESYT1 | -1.7417 | 1.08E-15 |
| LAYN | -1.73964 | 3.09E-05 |
| SH3BP5 | -1.73876 | 1.93E-09 |
| ERO1B | -1.73812 | 0.00019 |
| CYP2U1 | -1.73559 | 6.69E-11 |
| DNAJB2 | -1.73465 | 6.88E-08 |
| ZNF300P1 | -1.73442 | 6.88E-12 |
| SLC7A6OS | -1.73361 | 3.64E-13 |
| AAMDC | -1.73306 | 3.55E-10 |
| EPB41L2 | -1.73255 | 2.85E-06 |
| NR1H3 | -1.72943 | 4.15E-06 |
| HIVEP3 | -1.72816 | 0.000216 |
| R3HCC1 | -1.72809 | 3.01E-15 |
| SLC25A29 | -1.72593 | 1.08E-07 |
| DOCK6 | -1.72588 | 3.38E-11 |
| RPARP-AS1 | -1.7249 | 2.38E-09 |
| SEMA5A | -1.72298 | 3.72E-07 |
| CEBPA | -1.72268 | 5.22E-05 |
| WBP1L | -1.72039 | 4.52E-09 |
| NEGR1-IT1 | -1.72024 | 1.62E-18 |
| IL33 | -1.71971 | 0.000313 |
| ANKRD20A11P | -1.719 | 5.86E-07 |
| CCDC9B | -1.71898 | 0.00089 |
| ADGRD2 | -1.71887 | 3.94E-35 |
| REX1BD | -1.71756 | 4.29E-17 |
| ANO1 | -1.71682 | 0.011381 |
| PSMD6-AS2 | -1.71596 | 2.50E-14 |
| CSRP1 | -1.71486 | 6.85E-10 |
| POM121L9P | -1.71301 | 1.83E-09 |
| SAT2 | -1.71251 | 3.65E-14 |
| SMAD3 | -1.71222 | 3.74E-06 |
| ANAPC4 | -1.71151 | 3.36E-18 |
| RNF170 | -1.71149 | 1.70E-07 |
| RILP | -1.71057 | 1.61E-22 |
| MAFF | -1.71028 | 1.74E-05 |
| LRRN3 | -1.70758 | 3.86E-12 |
| KCNMA1 | -1.70756 | 0.01001 |
| DSPP | -1.70716 | 6.92E-29 |
| DMTN | -1.70666 | 2.72E-10 |
| FRY | -1.70519 | 0.004094 |
| RPUSD4 | -1.70507 | 8.17E-17 |
| COL5A3 | -1.70455 | 0.000378 |
| NAALADL1 | -1.70381 | 6.53E-36 |
| SSPN | -1.70381 | 1.42E-06 |
| C2CD2 | -1.70193 | 4.06E-08 |
| CRACR2A | -1.69904 | 0.006735 |
| THRSP | -1.69663 | 1.74E-08 |
| ZBED3 | -1.69655 | 2.02E-08 |
| FAM171A1 | -1.69325 | 0.014631 |
| ZNF503 | -1.69197 | 0.000148 |
| CHL1 | -1.69139 | 3.35E-05 |
| ZMAT1 | -1.68859 | 0.004533 |
| MEIS3P1 | -1.68645 | 4.24E-06 |
| ACAD10 | -1.68625 | 1.19E-19 |
| NFIC | -1.68408 | 2.57E-14 |
| MINK1 | -1.68404 | 2.42E-19 |
| LOC646214 | -1.6834 | 8.43E-09 |
| MAP3K3 | -1.68295 | 1.26E-10 |
| ZNF703 | -1.68271 | 0.015866 |
| FBXO2 | -1.681 | 0.000393 |
| PCDH12 | -1.68082 | 1.34E-15 |
| BDH2 | -1.67994 | 3.23E-09 |
| TPTE2P2 | -1.67655 | 4.56E-20 |
| KCNJ8 | -1.67582 | 2.32E-05 |
| RERG | -1.67491 | 0.018239 |
| BCL6B | -1.67373 | 6.58E-17 |
| DNAH7 | -1.67229 | 1.44E-15 |
| HIP1R | -1.67182 | 9.58E-08 |
| TACC1 | -1.6716 | 4.08E-07 |
| IQSEC1 | -1.67081 | 7.02E-10 |
| KCTD12 | -1.67054 | 0.000328 |
| HEPH | -1.67043 | 0.011981 |
| ENG | -1.66991 | 2.33E-07 |
| MYRIP | -1.66886 | 8.76E-05 |
| TBX5 | -1.6677 | 7.59E-07 |
| EPHB3 | -1.6673 | 6.76E-05 |
| MEIS1 | -1.66672 | 0.000366 |
| GPRC5B | -1.66478 | 0.00701 |
| UTRN | -1.66439 | 1.20E-08 |
| C14orf132 | -1.66378 | 0.015324 |
| RORC | -1.66179 | 0.001902 |
| FAM172A | -1.65921 | 8.06E-07 |
| ZNF580 | -1.65906 | 1.01E-14 |
| TSPOAP1 | -1.6578 | 1.36E-05 |
| WFS1 | -1.65735 | 0.000384 |
| ASMTL | -1.65562 | 6.46E-09 |
| WTIP | -1.65173 | 6.33E-10 |
| SLC66A2 | -1.65159 | 4.26E-14 |
| SERPINA5 | -1.65087 | 0.038816 |
| SERPINB9 | -1.6495 | 0.005799 |
| VIT | -1.64929 | 0.000152 |
| CYTH3 | -1.64791 | 5.14E-09 |
| ENPP7 | -1.64733 | 4.61E-26 |
| LRRC37A4P | -1.64719 | 1.75E-16 |
| FZD10-AS1 | -1.64708 | 1.21E-29 |
| LOC729732 | -1.64577 | 0.000991 |
| PARD3B | -1.64156 | 2.81E-05 |
| THYN1 | -1.64147 | 2.06E-11 |
| CBX6 | -1.64083 | 2.06E-11 |
| MAP1B | -1.63949 | 0.000542 |
| DYNC2H1 | -1.63897 | 0.000105 |
| VWA5A | -1.63862 | 0.004264 |
| LINC01798 | -1.63809 | 7.06E-34 |
| LOC100507557 | -1.63765 | 1.45E-12 |
| SYF2 | -1.63703 | 5.58E-22 |
| PLPP3 | -1.63674 | 6.45E-05 |
| RAMP2 | -1.63622 | 1.07E-11 |
| ZNF25 | -1.63403 | 1.99E-10 |
| ADM | -1.63262 | 0.029268 |
| KAT2A | -1.63252 | 4.08E-11 |
| HSPA12A | -1.63205 | 2.85E-05 |
| PAIP2B | -1.63156 | 5.64E-06 |
| FAHD2A | -1.63108 | 1.08E-10 |
| PLEKHA6 | -1.63071 | 0.001792 |
| IDNK | -1.63027 | 0.000104 |
| NEURL3 | -1.62955 | 1.63E-06 |
| TP73-AS1 | -1.62904 | 8.05E-06 |
| ZNF44 | -1.62659 | 0.000707 |
| GLI3 | -1.62633 | 0.009273 |
| GNG7 | -1.62548 | 1.60E-11 |
| TRIM8 | -1.62466 | 1.42E-10 |
| LMO2 | -1.62454 | 1.22E-05 |
| NXPH3 | -1.6239 | 6.79E-17 |
| GAB2 | -1.62281 | 2.00E-06 |
| BEX5 | -1.62171 | 0.006376 |
| PMP22 | -1.62042 | 0.008215 |
| PTPRG | -1.62033 | 5.61E-05 |
| KLHDC1 | -1.61922 | 0.000155 |
| GOLGA8A | -1.61863 | 0.000458 |
| CARD6 | -1.61697 | 5.09E-06 |
| TGFBR2 | -1.61608 | 9.12E-07 |
| DNMBP | -1.61564 | 0.000128 |
| MYO9A | -1.61546 | 7.53E-09 |
| TNRC6B | -1.61529 | 1.33E-17 |
| SPAG16 | -1.61514 | 0.001152 |
| GGT6 | -1.61465 | 0.000217 |
| PRR5 | -1.61444 | 4.40E-06 |
| DAPK2 | -1.61441 | 2.14E-13 |
| LOC101927769 | -1.61336 | 3.34E-18 |
| TRNP1 | -1.61291 | 7.34E-06 |
| ACSL1 | -1.61169 | 6.29E-05 |
| PI16 | -1.60953 | 7.03E-26 |
| PRKAR2B | -1.60949 | 0.012166 |
| LOC100289230 | -1.60897 | 1.74E-08 |
| GRASP | -1.60838 | 4.16E-21 |
| FADS3 | -1.60749 | 1.52E-12 |
| CAMK1 | -1.60558 | 1.22E-06 |
| NLGN2 | -1.60533 | 7.55E-14 |
| S100A1 | -1.60494 | 0.007679 |
| ENKD1 | -1.60384 | 3.54E-08 |
| KAZN | -1.60317 | 5.12E-05 |
| SOX7 | -1.60299 | 0.000448 |
| WDR6 | -1.60073 | 1.02E-11 |
| PDE1C | -1.59818 | 2.48E-05 |
| ARSD | -1.59796 | 0.000225 |
| NOD1 | -1.59764 | 4.44E-12 |
| NIPAL3 | -1.59553 | 2.01E-07 |
| VAT1 | -1.59497 | 2.44E-12 |
| PTEN | -1.59443 | 3.16E-05 |
| RIN1 | -1.5925 | 8.32E-16 |
| P2RY14 | -1.59229 | 4.54E-05 |
| APBB1IP | -1.59172 | 0.000121 |
| ZNF205 | -1.59149 | 3.80E-21 |
| LTB4R | -1.59067 | 1.81E-07 |
| LPAR6 | -1.59056 | 0.002387 |
| RUSC2 | -1.58956 | 2.55E-13 |
| HPS1 | -1.58834 | 3.71E-23 |
| LMBR1L | -1.58808 | 3.92E-16 |
| WDR13 | -1.58594 | 4.06E-12 |
| SH3D19 | -1.58375 | 4.20E-06 |
| MCOLN3 | -1.58367 | 0.001845 |
| GALK2 | -1.5828 | 5.29E-05 |
| IRF1-AS1 | -1.58204 | 2.27E-05 |
| TMX4 | -1.58159 | 3.81E-07 |
| TAF1C | -1.58151 | 1.18E-11 |
| LINC01410 | -1.58013 | 0.002843 |
| OAF | -1.57944 | 7.36E-12 |
| CHID1 | -1.579 | 1.11E-07 |
| GFOD1 | -1.57895 | 0.000229 |
| RCBTB2 | -1.57798 | 1.25E-06 |
| TSPAN4 | -1.57507 | 3.44E-10 |
| PDZD2 | -1.57501 | 0.008895 |
| CDK5RAP2 | -1.57385 | 1.13E-06 |
| IDUA | -1.57184 | 0.002961 |
| MRC2 | -1.57165 | 3.69E-05 |
| APOL3 | -1.57148 | 0.000313 |
| DOK1 | -1.57141 | 1.38E-11 |
| FTO | -1.57085 | 2.54E-13 |
| TMOD2 | -1.57002 | 1.87E-06 |
| MYBBP1A | -1.56977 | 2.15E-15 |
| ZNF667-AS1 | -1.56819 | 0.048411 |
| COL6A6 | -1.56742 | 2.39E-32 |
| PODN | -1.56727 | 0.000642 |
| PLEKHG4 | -1.56619 | 2.95E-05 |
| ECE1 | -1.56477 | 9.82E-09 |
| F13A1 | -1.56394 | 0.041718 |
| SPPL2B | -1.56372 | 7.20E-18 |
| FLNC | -1.56305 | 8.66E-09 |
| PAF1 | -1.5629 | 6.38E-13 |
| NMT2 | -1.5625 | 0.00151 |
| LINC00899 | -1.56243 | 0.000148 |
| WDR27 | -1.56101 | 4.06E-16 |
| GABBR1 | -1.56066 | 0.013303 |
| TNFRSF14 | -1.55888 | 4.28E-07 |
| IMPDH2 | -1.5587 | 8.06E-10 |
| PCED1A | -1.55833 | 5.85E-09 |
| ADRB2 | -1.55784 | 0.004878 |
| ZBTB3 | -1.55723 | 1.35E-18 |
| EOGT | -1.55688 | 5.74E-07 |
| WWP2 | -1.55678 | 5.70E-08 |
| CEP126 | -1.55571 | 2.37E-20 |
| FAM43A | -1.55515 | 0.00041 |
| SNCG | -1.55469 | 4.56E-10 |
| ITSN1 | -1.55344 | 1.51E-11 |
| SEPTIN4 | -1.55213 | 3.64E-07 |
| PLXDC1 | -1.55202 | 0.009217 |
| CES2 | -1.55198 | 5.96E-10 |
| PTPRM | -1.55184 | 0.00212 |
| TNKS | -1.55154 | 3.40E-06 |
| ZBTB40 | -1.54862 | 3.73E-15 |
| IGFBP5 | -1.54855 | 0.044889 |
| RNPC3 | -1.5477 | 8.29E-08 |
| ARL10 | -1.54598 | 1.42E-05 |
| ATG2A | -1.54595 | 2.89E-17 |
| HOOK2 | -1.54458 | 1.40E-10 |
| RBM5 | -1.54419 | 3.48E-12 |
| STAB1 | -1.54365 | 0.000616 |
| PTPRZ1 | -1.54333 | 0.0122 |
| OGFRL1 | -1.54282 | 0.000116 |
| SNCAIP | -1.54254 | 0.001737 |
| GASK1B | -1.54213 | 0.023165 |
| ACO1 | -1.54198 | 3.49E-06 |
| PLCD3 | -1.54148 | 4.50E-12 |
| MICU3 | -1.54004 | 4.09E-07 |
| KLC1 | -1.53967 | 1.03E-12 |
| KIAA1217 | -1.53797 | 5.65E-08 |
| SUN2 | -1.53773 | 1.77E-15 |
| OLFML3 | -1.53763 | 0.024867 |
| CLUH | -1.53555 | 2.50E-12 |
| KLHDC4 | -1.53501 | 1.15E-06 |
| SLC30A3 | -1.53458 | 4.66E-14 |
| FLYWCH1 | -1.53396 | 1.43E-14 |
| ZNF711 | -1.53306 | 0.019804 |
| CMTM7 | -1.53201 | 0.001623 |
| SLC12A1 | -1.532 | 1.56E-24 |
| CYP4V2 | -1.53129 | 0.000295 |
| HOXD10 | -1.53008 | 1.02E-06 |
| HOXA10 | -1.52973 | 3.17E-09 |
| SELENBP1 | -1.5293 | 0.018173 |
| USHBP1 | -1.52894 | 2.28E-39 |
| PELP1 | -1.52882 | 1.16E-13 |
| FGF2 | -1.52772 | 0.004089 |
| ZNF521 | -1.52737 | 0.022796 |
| CLDN19 | -1.52698 | 6.90E-32 |
| PEX11A | -1.52572 | 0.001421 |
| S100A4 | -1.52442 | 0.000838 |
| TMEM170B | -1.52265 | 9.37E-07 |
| PLEKHM1 | -1.52191 | 1.22E-18 |
| PDK2 | -1.52155 | 5.11E-08 |
| MARVELD1 | -1.52142 | 5.11E-07 |
| CKMT2-AS1 | -1.52102 | 1.39E-08 |
| MMP28 | -1.52001 | 1.92E-15 |
| SIM1 | -1.51971 | 0.010454 |
| HSDL2 | -1.51966 | 3.49E-08 |
| SLCO1A2 | -1.51877 | 4.90E-14 |
| NAV1 | -1.51817 | 0.000501 |
| MOCS1 | -1.51774 | 6.02E-16 |
| CCDC169 | -1.51764 | 1.13E-25 |
| CAMTA2 | -1.51758 | 3.31E-09 |
| EPB41L1 | -1.51666 | 0.001548 |
| PDPN | -1.51585 | 4.65E-05 |
| SOSTDC1 | -1.51527 | 0.025273 |
| DTNA | -1.51485 | 0.029735 |
| ZNF133 | -1.51481 | 3.05E-11 |
| RETSAT | -1.51407 | 1.95E-08 |
| NUMA1 | -1.51387 | 1.58E-09 |
| MYCBP2 | -1.51381 | 6.07E-09 |
| ITPK1 | -1.51318 | 1.59E-08 |
| FZD3 | -1.51219 | 0.000295 |
| MITF | -1.50791 | 2.50E-05 |
| IFFO2 | -1.50775 | 0.00012 |
| OSER1-DT | -1.50745 | 1.64E-09 |
| DUSP22 | -1.50736 | 1.56E-09 |
| HTRA1 | -1.50484 | 0.048592 |
| THNSL2 | -1.5042 | 0.000367 |
| AGAP3 | -1.50361 | 1.38E-15 |
| SGCG | -1.50295 | 4.19E-06 |
| RBPMS | -1.50191 | 0.001027 |
| CITED4 | -1.49823 | 0.006195 |
| NLRX1 | -1.49682 | 5.69E-12 |
| TPT1-AS1 | -1.49555 | 2.71E-07 |
| LOC101927811 | -1.49547 | 1.08E-08 |
| DDB2 | -1.4948 | 3.91E-07 |
| GALT | -1.49449 | 3.65E-08 |
| LFNG | -1.49351 | 0.009887 |
| NLN | -1.49316 | 2.76E-08 |
| LOC100505938 | -1.48994 | 3.33E-05 |
| PTGER4 | -1.48926 | 0.000166 |
| CALM3 | -1.48878 | 2.83E-17 |
| XIST | -1.48796 | 0.049947 |
| SMG6 | -1.48744 | 6.92E-15 |
| GALM | -1.48735 | 1.47E-08 |
| MAST4 | -1.48716 | 0.003031 |
| RCAN1 | -1.48622 | 0.000698 |
| NPR2 | -1.48602 | 5.70E-18 |
| MARCHF8 | -1.48322 | 6.35E-05 |
| KLHDC8B | -1.48318 | 1.54E-09 |
| RNPEPL1 | -1.48247 | 1.02E-22 |
| TRA2A | -1.48245 | 6.59E-06 |
| PIK3IP1 | -1.48218 | 1.05E-05 |
| PLAGL1 | -1.48181 | 0.021501 |
| SYT8 | -1.47732 | 3.16E-05 |
| GTPBP3 | -1.47681 | 2.29E-21 |
| CHDH | -1.47609 | 0.000482 |
| ZNF493 | -1.47556 | 0.001973 |
| PARM1 | -1.47538 | 0.011835 |
| HIC1 | -1.47516 | 2.50E-06 |
| PDE7B | -1.47504 | 2.64E-23 |
| HEIH | -1.47346 | 1.56E-10 |
| GALNT15 | -1.47275 | 4.23E-16 |
| LRRC37A2 | -1.47219 | 1.51E-07 |
| TMEM44-AS1 | -1.47198 | 2.50E-07 |
| GEMIN4 | -1.47166 | 1.18E-05 |
| MAPK11 | -1.46919 | 9.80E-15 |
| OSBPL1A | -1.46911 | 2.07E-07 |
| SLC29A4 | -1.46896 | 2.19E-08 |
| CUL9 | -1.46889 | 1.74E-11 |
| SLC6A16 | -1.46808 | 0.02947 |
| TFDP2 | -1.46721 | 1.32E-06 |
| AGPAT2 | -1.46613 | 3.71E-07 |
| PCGF5 | -1.46543 | 4.15E-07 |
| THRA | -1.46486 | 4.68E-05 |
| RXRA | -1.46485 | 4.43E-05 |
| CD3EAP | -1.46451 | 1.90E-09 |
| GLI2 | -1.46308 | 2.19E-13 |
| ANKRD53 | -1.46266 | 8.56E-33 |
| KIAA1755 | -1.46262 | 0.000149 |
| ARHGEF4 | -1.4599 | 0.00049 |
| RAB6B | -1.45918 | 6.81E-13 |
| USP48 | -1.4578 | 9.78E-16 |
| RAMP3 | -1.45765 | 6.69E-05 |
| C1orf226 | -1.45742 | 0.001877 |
| ZC3H7B | -1.45719 | 6.67E-08 |
| MTMR10 | -1.45654 | 2.15E-09 |
| RUNX3 | -1.45645 | 0.014745 |
| ZFYVE21 | -1.45627 | 6.65E-10 |
| MCTP2 | -1.45626 | 6.50E-33 |
| NAA80 | -1.45526 | 6.59E-14 |
| OSR1 | -1.45472 | 0.002128 |
| PLCD1 | -1.45388 | 1.01E-10 |
| RERE | -1.4538 | 3.16E-08 |
| NEO1 | -1.45334 | 0.000605 |
| LOC91548 | -1.45317 | 6.35E-26 |
| CNTFR-AS1 | -1.45107 | 1.69E-35 |
| STON1 | -1.45079 | 0.008281 |
| HOXB-AS1 | -1.44936 | 0.003358 |
| MIRLET7BHG | -1.44839 | 3.82E-06 |
| GMFG | -1.44793 | 0.004535 |
| PDGFRB | -1.44742 | 0.002509 |
| CC2D1A | -1.44523 | 6.45E-11 |
| RTN2 | -1.44431 | 6.78E-06 |
| JRK | -1.4438 | 1.70E-09 |
| UROD | -1.44222 | 6.90E-14 |
| ERVH-6 | -1.44182 | 1.70E-10 |
| EGR2 | -1.44169 | 0.004515 |
| GIGYF1 | -1.44157 | 6.77E-13 |
| ZBTB47 | -1.44127 | 2.42E-24 |
| TAPT1-AS1 | -1.44126 | 2.92E-05 |
| MTMR9 | -1.44081 | 7.53E-14 |
| UBE4B | -1.44079 | 4.93E-10 |
| HDAC7 | -1.43991 | 3.41E-11 |
| PDK3 | -1.43986 | 0.000142 |
| BAIAP2-DT | -1.43983 | 1.39E-05 |
| TFE3 | -1.43975 | 5.70E-18 |
| EDF1 | -1.43955 | 3.23E-15 |
| PRPF6 | -1.43952 | 1.08E-10 |
| ATXN7L1 | -1.43753 | 8.46E-07 |
| UNC119B | -1.43746 | 1.78E-12 |
| HSD17B1 | -1.43731 | 1.12E-09 |
| DMAP1 | -1.43638 | 3.42E-10 |
| NPR3 | -1.43629 | 0.044714 |
| ACSS1 | -1.43486 | 0.000539 |
| HADH | -1.43434 | 7.31E-10 |
| BCORL1 | -1.43348 | 1.39E-34 |
| FOXN3 | -1.4332 | 6.62E-10 |
| DUSP6 | -1.4329 | 0.008032 |
| CCNL2 | -1.43276 | 2.18E-07 |
| TEFM | -1.43193 | 7.45E-05 |
| ORAI3 | -1.43106 | 2.76E-05 |
| WWOX | -1.431 | 0.003217 |
| NOXA1 | -1.43024 | 7.96E-14 |
| NOS1AP | -1.42975 | 1.15E-09 |
| TBC1D13 | -1.42973 | 5.36E-21 |
| MOB3C | -1.42938 | 5.28E-11 |
| EXOC3 | -1.42928 | 6.77E-12 |
| WDR89 | -1.42819 | 7.03E-26 |
| ERCC5 | -1.42779 | 3.02E-10 |
| SYDE1 | -1.42698 | 7.06E-29 |
| DELE1 | -1.42671 | 7.20E-14 |
| ZNF573 | -1.42646 | 2.75E-05 |
| SIK3 | -1.42619 | 3.21E-06 |
| ENTPD1 | -1.42608 | 0.01284 |
| MTERF2 | -1.42599 | 6.24E-06 |
| TMEM204 | -1.42469 | 0.008129 |
| TOM1L2 | -1.42366 | 6.26E-06 |
| CAVIN3 | -1.42319 | 5.02E-05 |
| ALDH1B1 | -1.42305 | 8.54E-12 |
| MIR99AHG | -1.42205 | 0.002512 |
| VPS36 | -1.42164 | 5.36E-08 |
| AVPI1 | -1.41901 | 5.96E-05 |
| FKBP1B | -1.41845 | 0.005643 |
| PRDM15 | -1.41807 | 3.44E-10 |
| COA8 | -1.41728 | 5.14E-12 |
| ARHGAP31 | -1.41694 | 5.13E-07 |
| RIPK4 | -1.41692 | 0.000744 |
| GABPB1-IT1 | -1.41645 | 1.14E-15 |
| SLC25A23 | -1.41631 | 1.98E-06 |
| ZFP14 | -1.41598 | 3.86E-34 |
| PTDSS2 | -1.41505 | 2.44E-18 |
| PPM1M | -1.41427 | 3.82E-06 |
| DDX54 | -1.41311 | 3.28E-14 |
| CROT | -1.41217 | 0.000548 |
| SPTLC3 | -1.41204 | 1.17E-25 |
| CSN1S1 | -1.4106 | 0.02674 |
| REEP5 | -1.41005 | 0.000124 |
| RGMB | -1.40997 | 0.003261 |
| DAB2IP | -1.40995 | 5.56E-10 |
| DNM1 | -1.40809 | 4.58E-05 |
| INTS1 | -1.40691 | 1.15E-08 |
| FYN | -1.40649 | 2.78E-05 |
| PLEKHM3 | -1.40646 | 5.19E-05 |
| OPTN | -1.40604 | 0.000167 |
| ANG | -1.40596 | 0.00038 |
| KLB | -1.40466 | 8.45E-13 |
| CYB5D1 | -1.40452 | 5.67E-06 |
| IVD | -1.40416 | 0.000304 |
| NYNRIN | -1.40402 | 0.000152 |
| EPN1 | -1.40375 | 1.04E-17 |
| FREM1 | -1.40333 | 3.98E-14 |
| TCF7 | -1.40284 | 2.27E-11 |
| PHC1 | -1.40263 | 2.97E-05 |
| ADGRA2 | -1.40242 | 0.001255 |
| FAM117A | -1.40176 | 7.90E-05 |
| CEP120 | -1.40154 | 5.42E-12 |
| RELL1 | -1.40081 | 1.43E-06 |
| FLJ42627 | -1.40036 | 3.69E-11 |
| ZNF502 | -1.39928 | 1.29E-08 |
| ARHGAP24 | -1.39838 | 9.18E-06 |
| CCND2 | -1.39783 | 0.02021 |
| RBM10 | -1.39772 | 4.21E-16 |
| TMCO4 | -1.39689 | 3.19E-05 |
| PKP4 | -1.39659 | 5.22E-08 |
| NUDT7 | -1.39558 | 0.008747 |
| GATD1 | -1.39537 | 1.96E-10 |
| TDRP | -1.39444 | 0.000339 |
| ZSCAN18 | -1.39309 | 0.009215 |
| MRTFB | -1.39069 | 3.60E-07 |
| MCF2L | -1.39067 | 0.022344 |
| SUGP1 | -1.38997 | 3.27E-27 |
| YPEL3 | -1.38978 | 6.15E-07 |
| SKI | -1.38962 | 9.12E-11 |
| NFATC2 | -1.38883 | 0.002094 |
| XRN1 | -1.38829 | 5.69E-12 |
| GPD1L | -1.38649 | 0.016671 |
| CAT | -1.3864 | 4.29E-09 |
| APCDD1L | -1.38617 | 0.000277 |
| STEAP2 | -1.38612 | 0.025776 |
| SLC25A16 | -1.3859 | 4.01E-09 |
| GPER1 | -1.38561 | 6.25E-05 |
| ACADL | -1.38511 | 3.96E-05 |
| TNFRSF10D | -1.38495 | 0.000989 |
| PXN | -1.38429 | 2.12E-06 |
| TXN2 | -1.38354 | 8.35E-14 |
| STAT2 | -1.38193 | 3.48E-08 |
| ZNF579 | -1.38177 | 3.15E-13 |
| ZDHHC14 | -1.38172 | 6.53E-06 |
| ANKRD20A12P | -1.37971 | 6.25E-27 |
| NPIPB15 | -1.37879 | 0.000566 |
| FCF1 | -1.3783 | 8.04E-13 |
| DOK4 | -1.37704 | 6.88E-07 |
| ZNF554 | -1.37602 | 2.55E-22 |
| SH3KBP1 | -1.37522 | 7.94E-05 |
| DIXDC1 | -1.37466 | 0.001125 |
| STX8 | -1.37446 | 6.42E-12 |
| CEBPA-DT | -1.37424 | 6.73E-25 |
| PCCA | -1.37391 | 1.42E-05 |
| ZSWIM9 | -1.37277 | 4.88E-18 |
| ATP6V0A1 | -1.3721 | 4.08E-12 |
| TRIOBP | -1.37171 | 3.51E-15 |
| PDE4A | -1.37158 | 9.58E-05 |
| TMEM72 | -1.3714 | 1.06E-22 |
| DDAH2 | -1.36882 | 3.43E-06 |
| TCP11L2 | -1.36826 | 3.82E-21 |
| GCN1 | -1.36699 | 5.35E-12 |
| SH3BP5-AS1 | -1.36688 | 2.17E-13 |
| KLF8 | -1.36683 | 0.001886 |
| RILPL2 | -1.36536 | 2.19E-09 |
| TYRO3 | -1.36473 | 6.62E-06 |
| DTX1 | -1.36419 | 3.77E-28 |
| AVPR2 | -1.364 | 1.29E-30 |
| ANKRD6 | -1.36395 | 0.000681 |
| PRNP | -1.3639 | 2.79E-05 |
| TAPT1 | -1.36382 | 0.000423 |
| RFX2 | -1.36378 | 7.54E-13 |
| GTF2IP12 | -1.36356 | 2.03E-15 |
| MINDY1 | -1.36351 | 1.25E-07 |
| ARHGEF5 | -1.36311 | 3.54E-07 |
| FIGN | -1.36235 | 0.008508 |
| RALGDS | -1.36205 | 6.84E-09 |
| FBLN2 | -1.36146 | 0.016035 |
| LIX1L | -1.36146 | 0.000177 |
| CRTAP | -1.36072 | 1.84E-05 |
| PIP4P2 | -1.36067 | 1.22E-05 |
| CTNNAL1 | -1.36045 | 0.014818 |
| FGF14-AS2 | -1.36038 | 1.43E-09 |
| EBF4 | -1.36021 | 8.31E-08 |
| BMPER | -1.36018 | 1.64E-37 |
| TIMM10B | -1.36008 | 2.07E-06 |
| GATC | -1.35947 | 2.65E-14 |
| ETV1 | -1.35937 | 0.010091 |
| C2orf81 | -1.35852 | 3.87E-16 |
| USP28 | -1.35796 | 7.65E-13 |
| KBTBD8 | -1.35682 | 1.61E-09 |
| KRBOX4 | -1.35586 | 6.05E-09 |
| ZNF862 | -1.35495 | 9.07E-05 |
| ITGA9-AS1 | -1.35482 | 1.15E-14 |
| PGAP4 | -1.35436 | 5.52E-08 |
| STMN3 | -1.35214 | 0.002012 |
| GJA4 | -1.35212 | 4.83E-13 |
| LPIN1 | -1.35173 | 0.005519 |
| NRIP2 | -1.35171 | 5.96E-37 |
| MAN2C1 | -1.35134 | 9.02E-08 |
| LOC100130452 | -1.34852 | 1.58E-24 |
| ZNF790-AS1 | -1.34817 | 4.37E-06 |
| L3MBTL4 | -1.34676 | 0.001424 |
| DTX3 | -1.34636 | 3.53E-05 |
| CBR4 | -1.34535 | 0.000816 |
| WDR19 | -1.34519 | 0.000926 |
| PTHLH | -1.34377 | 0.000495 |
| FAM3D | -1.34325 | 2.18E-06 |
| ERMAP | -1.34316 | 4.61E-09 |
| RAB3IL1 | -1.34297 | 6.23E-19 |
| KIF1C | -1.34261 | 1.24E-12 |
| EPHB1 | -1.34207 | 0.005604 |
| WDR81 | -1.34175 | 6.37E-15 |
| CIB2 | -1.34146 | 0.001309 |
| ECHDC1 | -1.34142 | 0.003817 |
| PER2 | -1.34131 | 0.000255 |
| FAM161B | -1.34128 | 9.18E-08 |
| ACKR4 | -1.3402 | 0.000103 |
| TECPR2 | -1.33927 | 2.61E-09 |
| SPRY1 | -1.33866 | 0.008841 |
| TFEB | -1.33811 | 1.71E-06 |
| EWSAT1 | -1.33774 | 8.09E-08 |
| STAT6 | -1.33745 | 5.62E-09 |
| ARMC5 | -1.33739 | 1.30E-18 |
| KLK3 | -1.33727 | 8.69E-15 |
| ZGRF1 | -1.3372 | 4.14E-15 |
| LAMA1 | -1.33598 | 1.06E-05 |
| ITGB4 | -1.33588 | 0.007763 |
| ARHGAP20 | -1.33481 | 8.19E-09 |
| SLC25A28 | -1.33453 | 1.96E-18 |
| ICA1L | -1.33415 | 1.00E-06 |
| FLI1 | -1.33354 | 0.00362 |
| SLC24A1 | -1.33287 | 6.94E-06 |
| MMAA | -1.33225 | 0.000278 |
| ZNF444 | -1.33181 | 1.12E-11 |
| LTBP3 | -1.3305 | 2.48E-05 |
| AKR7A2 | -1.33022 | 6.59E-07 |
| METAP1D | -1.33 | 8.90E-08 |
| C4orf3 | -1.32982 | 1.98E-09 |
| LDHB | -1.32974 | 0.018449 |
| FGL2 | -1.32962 | 0.046363 |
| ITGA6 | -1.32938 | 0.002385 |
| NLRP1 | -1.32925 | 7.49E-07 |
| PRRG1 | -1.32901 | 0.001502 |
| PHLDB2 | -1.32839 | 0.003026 |
| ADD1 | -1.32825 | 7.89E-11 |
| SAFB2 | -1.32749 | 1.38E-20 |
| ITPR2 | -1.32718 | 0.000681 |
| SGSH | -1.3271 | 8.71E-07 |
| LOC100505549 | -1.327 | 1.78E-05 |
| SNTB2 | -1.32697 | 1.21E-09 |
| FOXP2 | -1.32603 | 1.91E-22 |
| ATP8A1 | -1.32585 | 0.004418 |
| SART1 | -1.32568 | 1.96E-10 |
| PLA2G6 | -1.3244 | 6.07E-11 |
| C19orf12 | -1.32431 | 1.26E-06 |
| BHMT2 | -1.32334 | 0.005843 |
| KIF1B | -1.323 | 7.73E-07 |
| MEGF10 | -1.32208 | 0.023876 |
| DUBR | -1.32154 | 0.000811 |
| NCAPH2 | -1.32152 | 4.76E-14 |
| ZNF219 | -1.32139 | 3.70E-27 |
| SNORD114-3 | -1.3213 | 9.27E-07 |
| AMPD1 | -1.3213 | 1.82E-16 |
| PEMT | -1.32016 | 8.86E-06 |
| SLC35E1 | -1.32008 | 7.13E-07 |
| ETS2 | -1.31895 | 0.000164 |
| MB21D2 | -1.31891 | 0.000441 |
| ADAMTS10 | -1.31845 | 7.41E-14 |
| DIP2C | -1.31806 | 0.000409 |
| SLC48A1 | -1.31768 | 1.36E-05 |
| ZNF490 | -1.31763 | 6.53E-22 |
| BRAP | -1.3172 | 2.74E-07 |
| TTC12 | -1.31684 | 0.004647 |
| RP9P | -1.31649 | 3.16E-11 |
| PIK3C2B | -1.31623 | 3.86E-06 |
| FBXL20 | -1.31577 | 0.011682 |
| NEIL2 | -1.31564 | 1.58E-08 |
| SSC5D | -1.31535 | 9.74E-13 |
| FUT8-AS1 | -1.31502 | 1.49E-27 |
| MPV17L | -1.31482 | 0.007483 |
| NPDC1 | -1.31466 | 0.000979 |
| MUC20 | -1.31435 | 0.041762 |
| LRP3 | -1.31431 | 2.43E-08 |
| GLT8D2 | -1.31335 | 0.046914 |
| CYTH4 | -1.31137 | 0.000299 |
| ST6GALNAC6 | -1.30901 | 3.00E-16 |
| ALK | -1.30888 | 1.56E-10 |
| CC2D2A | -1.30793 | 1.15E-05 |
| SENP7 | -1.3078 | 9.74E-12 |
| COL17A1 | -1.30661 | 4.59E-05 |
| HHLA3 | -1.30623 | 4.11E-11 |
| TRAK1 | -1.30614 | 1.08E-06 |
| TMEM42 | -1.30504 | 2.06E-07 |
| GID4 | -1.30485 | 8.21E-08 |
| PRTFDC1 | -1.30353 | 0.047528 |
| RNF125 | -1.30302 | 0.000833 |
| HSD17B8 | -1.30295 | 0.00068 |
| IL34 | -1.30274 | 3.48E-11 |
| KCNT2 | -1.30121 | 6.15E-05 |
| NUP214 | -1.30063 | 3.50E-12 |
| LINC02381 | -1.30029 | 0.000666 |
| TMEM256 | -1.29888 | 1.22E-06 |
| NICN1 | -1.29887 | 3.29E-08 |
| TNFRSF21 | -1.29883 | 0.005153 |
| DPH5 | -1.29654 | 5.76E-10 |
| SPSB3 | -1.2964 | 7.26E-10 |
| FAH | -1.29636 | 0.000813 |
| ABCB1 | -1.29615 | 3.83E-39 |
| IFFO1 | -1.2958 | 6.96E-06 |
| KLF3-AS1 | -1.29444 | 0.000123 |
| TAL1 | -1.29179 | 3.78E-09 |
| FAM49A | -1.29116 | 0.000105 |
| PODXL | -1.29058 | 0.000133 |
| RAPGEF3 | -1.28994 | 1.87E-23 |
| POLL | -1.2884 | 3.58E-14 |
| GOLGA2P10 | -1.2883 | 2.86E-07 |
| TSC1 | -1.28777 | 2.59E-09 |
| FAM160B2 | -1.28768 | 2.04E-09 |
| KMT2A | -1.28704 | 1.71E-12 |
| MEF2D | -1.28621 | 2.51E-10 |
| CST3 | -1.28585 | 0.017855 |
| IFI27L2 | -1.28501 | 3.33E-08 |
| FBXW4 | -1.28489 | 1.86E-10 |
| ZNF337 | -1.28485 | 8.26E-11 |
| NEURL4 | -1.28475 | 2.07E-12 |
| LUZP1 | -1.28449 | 8.82E-14 |
| DDX59 | -1.28385 | 2.89E-10 |
| SELENOO | -1.28374 | 1.77E-12 |
| SNTB1 | -1.28339 | 3.90E-05 |
| NT5C3B | -1.28317 | 4.00E-06 |
| CNTN4 | -1.2831 | 0.005877 |
| FAN1 | -1.28263 | 2.71E-08 |
| IGBP1 | -1.28246 | 1.62E-07 |
| DTNB | -1.2824 | 1.60E-12 |
| HLX | -1.28235 | 1.08E-05 |
| SLC27A3 | -1.2803 | 0.002578 |
| C8orf58 | -1.27971 | 0.000322 |
| SRGAP2C | -1.27968 | 1.96E-06 |
| ASIC1 | -1.27881 | 2.02E-26 |
| EMID1 | -1.27853 | 6.41E-07 |
| FAM120C | -1.27807 | 0.00126 |
| IFI27L1 | -1.27701 | 1.44E-10 |
| EPC1 | -1.27689 | 1.33E-11 |
| NTRK3 | -1.27622 | 0.000567 |
| TUT1 | -1.2761 | 3.86E-22 |
| FBXW7 | -1.27599 | 1.60E-10 |
| TPPP | -1.27572 | 0.000896 |
| SLC4A7 | -1.27475 | 3.18E-05 |
| JADE2 | -1.2746 | 0.008841 |
| TADA2B | -1.27435 | 6.86E-08 |
| TBC1D2B | -1.27399 | 1.55E-05 |
| MGMT | -1.27367 | 0.00064 |
| SYMPK | -1.27233 | 0.000611 |
| SPHKAP | -1.27227 | 0.004512 |
| ZFYVE1 | -1.27172 | 2.62E-08 |
| POLR2A | -1.27038 | 1.67E-11 |
| LYVE1 | -1.2698 | 1.91E-07 |
| ZC3H12C | -1.26945 | 0.03064 |
| STOM | -1.26925 | 0.000288 |
| ATP5F1D | -1.26907 | 1.43E-09 |
| VAMP3 | -1.26837 | 2.20E-14 |
| VGLL3 | -1.26831 | 0.001248 |
| VPS11 | -1.26816 | 1.03E-11 |
| LOC400499 | -1.26814 | 7.75E-14 |
| RBSN | -1.26792 | 2.70E-10 |
| SYNE1 | -1.26742 | 0.005364 |
| POLK | -1.2646 | 5.58E-11 |
| TPTEP1 | -1.26413 | 1.84E-06 |
| LRIG2 | -1.26288 | 1.25E-07 |
| BEND6 | -1.25965 | 1.97E-30 |
| SNX22 | -1.25953 | 3.89E-07 |
| CBR3 | -1.25891 | 0.001419 |
| ZFYVE27 | -1.2589 | 3.51E-09 |
| STK40 | -1.25824 | 2.29E-12 |
| YPEL4 | -1.25821 | 8.08E-24 |
| RXRB | -1.2573 | 2.90E-12 |
| SORBS1 | -1.25719 | 2.40E-10 |
| SMARCC2 | -1.25658 | 4.14E-13 |
| POLN | -1.25566 | 1.39E-06 |
| C1R | -1.25475 | 0.01398 |
| TPRG1L | -1.2543 | 1.39E-09 |
| NFRKB | -1.25346 | 4.58E-11 |
| P2RY12 | -1.25346 | 4.37E-09 |
| C11orf95 | -1.2532 | 2.45E-05 |
| LINC00957 | -1.25317 | 1.82E-13 |
| SAMD4A | -1.25309 | 0.000487 |
| SLC25A26 | -1.25266 | 9.84E-12 |
| ESD | -1.25168 | 8.40E-10 |
| KCTD2 | -1.25143 | 1.00E-06 |
| TSC22D4 | -1.25142 | 4.03E-05 |
| GPAT4 | -1.2514 | 1.76E-05 |
| KMT2D | -1.25104 | 9.74E-10 |
| KATNAL1 | -1.25096 | 8.58E-05 |
| ST3GAL3 | -1.24998 | 3.43E-09 |
| SMU1 | -1.24969 | 2.21E-08 |
| BTBD6 | -1.24817 | 7.80E-08 |
| ARHGAP26 | -1.24815 | 0.000991 |
| SERP2 | -1.24757 | 3.18E-30 |
| CCDC163 | -1.24733 | 4.20E-29 |
| TMEM71 | -1.24725 | 0.009742 |
| DIPK2A | -1.24693 | 0.000705 |
| MYL6B | -1.24629 | 1.01E-07 |
| AASS | -1.24612 | 5.29E-15 |
| INF2 | -1.24609 | 7.65E-13 |
| USE1 | -1.24563 | 3.38E-09 |
| GPSM1 | -1.24556 | 5.52E-06 |
| TSPAN31 | -1.2455 | 0.000771 |
| OBSL1 | -1.24506 | 0.000585 |
| RBMS2 | -1.24422 | 0.002574 |
| MYLIP | -1.24402 | 0.001183 |
| PCYOX1 | -1.24373 | 1.74E-07 |
| PRR34-AS1 | -1.24296 | 9.53E-06 |
| HIPK3 | -1.24248 | 2.22E-09 |
| SETD3 | -1.24195 | 1.92E-15 |
| UCK1 | -1.24164 | 2.07E-08 |
| EIF3J-DT | -1.2413 | 1.17E-07 |
| LINC00997 | -1.24101 | 8.11E-10 |
| NUDCD3 | -1.24101 | 5.58E-09 |
| ZNF581 | -1.2409 | 9.73E-09 |
| CCDC152 | -1.24031 | 2.24E-06 |
| TTYH2 | -1.24011 | 0.000471 |
| GRB10 | -1.23889 | 0.002069 |
| ZNF266 | -1.23824 | 1.46E-07 |
| PDPR | -1.23811 | 4.30E-06 |
| LSP1 | -1.23811 | 0.001499 |
| TBC1D17 | -1.23788 | 1.69E-07 |
| SYTL4 | -1.23779 | 1.00E-06 |
| FER | -1.23637 | 7.37E-07 |
| GPR153 | -1.23561 | 4.09E-08 |
| FMC1 | -1.23351 | 3.48E-05 |
| GBE1 | -1.23317 | 8.80E-05 |
| ERMARD | -1.23291 | 9.66E-06 |
| PEBP1 | -1.23238 | 9.18E-07 |
| MPST | -1.2307 | 7.60E-07 |
| CEP164 | -1.23034 | 2.30E-10 |
| PARP10 | -1.23003 | 0.000297 |
| CREB5 | -1.22956 | 3.61E-05 |
| MAP1LC3A | -1.22907 | 0.000448 |
| PAOX | -1.2281 | 3.23E-05 |
| PGAP1 | -1.22785 | 0.000162 |
| EXOC4 | -1.22767 | 9.37E-09 |
| RASL12 | -1.22734 | 0.00067 |
| ACAP1 | -1.22623 | 5.72E-27 |
| TNIP1 | -1.2262 | 1.28E-08 |
| ABCB5 | -1.22552 | 4.14E-15 |
| SCAP | -1.22435 | 3.13E-11 |
| ENGASE | -1.22431 | 1.62E-15 |
| ZNF563 | -1.22338 | 3.36E-09 |
| ARHGAP4 | -1.22308 | 5.75E-08 |
| ATP6AP1L | -1.22305 | 0.00062 |
| TBC1D25 | -1.22178 | 8.56E-19 |
| CARMIL3 | -1.22111 | 2.36E-16 |
| SMIM11A | -1.22031 | 4.83E-06 |
| CASP9 | -1.22003 | 5.76E-11 |
| ECSIT | -1.21968 | 8.03E-07 |
| XPA | -1.21885 | 4.32E-09 |
| PML | -1.21752 | 0.000116 |
| ZFHX3 | -1.21741 | 0.000972 |
| TCTN1 | -1.21696 | 3.77E-14 |
| LOC100289333 | -1.21648 | 4.37E-08 |
| RAB28 | -1.21598 | 1.26E-10 |
| EDA2R | -1.21557 | 4.50E-27 |
| COQ8B | -1.21556 | 9.05E-17 |
| TRIP10 | -1.21554 | 7.58E-05 |
| MAML3 | -1.21537 | 0.003402 |
| CABLES1 | -1.21525 | 0.000167 |
| COBLL1 | -1.21433 | 0.003002 |
| ZNF324 | -1.21433 | 3.71E-07 |
| ZNF791 | -1.21411 | 5.16E-08 |
| LYRM1 | -1.21398 | 2.58E-08 |
| ZER1 | -1.21252 | 1.71E-11 |
| RAX2 | -1.21183 | 2.10E-22 |
| VPS13D | -1.21165 | 1.71E-09 |
| WWC1 | -1.21138 | 0.001154 |
| JAML | -1.21136 | 0.000549 |
| SNED1 | -1.21013 | 0.009376 |
| DGLUCY | -1.2097 | 0.002672 |
| MAP3K11 | -1.20949 | 4.76E-10 |
| ZBTB22 | -1.20922 | 3.42E-11 |
| FRMD4A | -1.20922 | 0.001164 |
| IGH | -1.20868 | 6.98E-14 |
| PKIG | -1.20857 | 4.32E-05 |
| SH2D3A | -1.20841 | 3.63E-22 |
| PLEKHA8P1 | -1.20834 | 8.60E-10 |
| CNTN1 | -1.20701 | 0.037627 |
| EPM2A | -1.20694 | 4.75E-07 |
| S100A13 | -1.20655 | 4.82E-05 |
| LZTS1 | -1.20622 | 5.39E-07 |
| FAM135A | -1.20609 | 0.000268 |
| BICRAL | -1.20602 | 4.64E-12 |
| CHRNB2 | -1.20568 | 6.21E-20 |
| ZNF654 | -1.20476 | 6.36E-10 |
| SMIM3 | -1.20374 | 7.96E-05 |
| CLBA1 | -1.2025 | 4.66E-06 |
| CYP4F22 | -1.20221 | 0.006386 |
| MAPK3 | -1.20168 | 1.00E-13 |
| CRIPT | -1.20158 | 0.000121 |
| FRS2 | -1.20102 | 0.007991 |
| IL4R | -1.20047 | 0.000116 |
| TNFRSF25 | -1.19993 | 0.001864 |
| ANKZF1 | -1.19985 | 1.62E-11 |
| TRMT61A | -1.19945 | 5.54E-21 |
| MIGA1 | -1.19917 | 5.32E-07 |
| SCAI | -1.19907 | 8.09E-05 |
| LUC7L | -1.19883 | 1.00E-06 |
| ANXA1 | -1.19804 | 0.013936 |
| TMEM163 | -1.19792 | 0.000246 |
| HDGFL2 | -1.19786 | 8.89E-10 |
| ARRDC4 | -1.19769 | 0.020028 |
| PAN2 | -1.19757 | 0.000906 |
| CNPPD1 | -1.19755 | 1.04E-13 |
| TP53 | -1.19736 | 0.012419 |
| WDPCP | -1.19706 | 1.53E-12 |
| NCKIPSD | -1.19644 | 1.89E-08 |
| FST | -1.19473 | 0.049056 |
| VPS4A | -1.19454 | 4.13E-09 |
| PPP3CB | -1.19416 | 6.28E-09 |
| NSUN5P1 | -1.19366 | 5.40E-09 |
| MT1E | -1.19313 | 0.005879 |
| EIF3L | -1.19248 | 5.93E-12 |
| ZSCAN26 | -1.19242 | 1.42E-06 |
| KALRN | -1.1922 | 0.002551 |
| PARP6 | -1.19031 | 4.22E-08 |
| CLIC5 | -1.18991 | 7.36E-12 |
| ZNF346 | -1.18798 | 2.02E-06 |
| KLHL22 | -1.18769 | 0.000135 |
| SPOCK3 | -1.18759 | 2.12E-19 |
| PTPN14 | -1.18654 | 0.000755 |
| SERPINB6 | -1.18567 | 0.000435 |
| SLC9A9 | -1.18554 | 9.03E-08 |
| SLC25A11 | -1.18442 | 0.000218 |
| EHD1 | -1.18442 | 7.50E-11 |
| LOC647070 | -1.18434 | 7.35E-15 |
| TBC1D16 | -1.18415 | 0.00077 |
| HIVEP2 | -1.18358 | 2.85E-08 |
| LINC02064 | -1.18286 | 1.78E-23 |
| GAS1 | -1.18265 | 0.030883 |
| ZNF268 | -1.18252 | 7.73E-05 |
| PRKAG2-AS1 | -1.18175 | 0.000684 |
| SLC25A45 | -1.18152 | 6.52E-06 |
| PLCG1 | -1.1812 | 5.11E-09 |
| SIL1 | -1.18066 | 1.05E-06 |
| THEM4 | -1.18006 | 0.011244 |
| LRRC75B | -1.17836 | 2.65E-05 |
| C20orf194 | -1.17809 | 6.18E-07 |
| TIMP2 | -1.17798 | 0.00225 |
| HNF4A | -1.17793 | 2.84E-16 |
| LOC101927814 | -1.17789 | 5.42E-14 |
| SAP30L | -1.17749 | 7.90E-05 |
| ARHGEF10L | -1.17718 | 1.25E-05 |
| GGT7 | -1.17676 | 7.16E-09 |
| CYP17A1 | -1.17648 | 1.33E-25 |
| MAP2K5 | -1.17617 | 2.45E-06 |
| KIAA1656 | -1.17551 | 1.97E-19 |
| NBEA | -1.1749 | 0.006773 |
| ADD3 | -1.17429 | 0.002428 |
| COQ4 | -1.1742 | 9.87E-06 |
| SCARB1 | -1.17414 | 0.002149 |
| APOL6 | -1.174 | 0.003011 |
| BICC1 | -1.174 | 0.036482 |
| NPRL3 | -1.1734 | 8.10E-10 |
| COL25A1 | -1.1734 | 1.54E-09 |
| NUDT11 | -1.17333 | 0.001715 |
| PLEKHF1 | -1.1729 | 0.004704 |
| VPS13C | -1.17201 | 0.004587 |
| CAND2 | -1.17196 | 0.011091 |
| BNIP3L | -1.17186 | 0.000174 |
| XAB2 | -1.17161 | 4.74E-18 |
| RPH3AL | -1.17132 | 0.000226 |
| SKAP2 | -1.17093 | 0.000202 |
| RAB24 | -1.17069 | 1.47E-05 |
| PIGV | -1.17054 | 7.04E-08 |
| CDKL3 | -1.16998 | 7.27E-06 |
| MAP3K6 | -1.16967 | 0.001642 |
| CD320 | -1.16917 | 0.012954 |
| KIAA1671 | -1.16776 | 8.03E-05 |
| ZNF775 | -1.16766 | 2.45E-09 |
| FLJ37453 | -1.16694 | 0.001023 |
| ADARB1 | -1.16652 | 0.000586 |
| LAMTOR4 | -1.16531 | 1.81E-09 |
| LAMA5-AS1 | -1.16531 | 5.80E-16 |
| ZNF117 | -1.1653 | 0.02204 |
| CSPG4 | -1.16464 | 0.000119 |
| SNU13 | -1.16391 | 4.49E-10 |
| TLCD5 | -1.16353 | 1.04E-05 |
| PLA2G12A | -1.16315 | 3.22E-05 |
| NEDD9 | -1.16296 | 0.005326 |
| IFT88 | -1.16258 | 0.000482 |
| GLIPR2 | -1.16231 | 0.00897 |
| FOXP1-IT1 | -1.16224 | 0.000563 |
| DTX4 | -1.162 | 0.004673 |
| TRIM56 | -1.16177 | 2.66E-09 |
| CYP46A1 | -1.16166 | 9.72E-13 |
| HEXD | -1.16147 | 0.000915 |
| PRKG1 | -1.16144 | 0.009974 |
| TFAP4 | -1.16113 | 3.11E-17 |
| NEK3 | -1.16109 | 0.000432 |
| MZT2B | -1.16098 | 1.61E-06 |
| MYLK3 | -1.16051 | 1.26E-16 |
| IRF2BP1 | -1.16044 | 8.35E-21 |
| LZTR1 | -1.16029 | 2.79E-08 |
| ZBTB44 | -1.15999 | 1.46E-06 |
| LINC01138 | -1.15877 | 0.013693 |
| RDH5 | -1.15801 | 2.14E-29 |
| CYHR1 | -1.15773 | 0.000631 |
| PTCRA | -1.15771 | 3.26E-20 |
| VRTN | -1.15751 | 2.74E-15 |
| DSTNP2 | -1.15703 | 3.37E-11 |
| ITSN2 | -1.15679 | 2.06E-15 |
| ZNF287 | -1.1565 | 2.09E-17 |
| PIP4K2B | -1.15635 | 0.007183 |
| ZNF839 | -1.15551 | 4.48E-07 |
| EPS8L2 | -1.15547 | 0.004838 |
| HTR4 | -1.15496 | 8.98E-12 |
| OFD1 | -1.15479 | 2.83E-07 |
| DOCK11 | -1.15446 | 0.006253 |
| ZNF564 | -1.15444 | 8.52E-08 |
| OSBPL5 | -1.15437 | 3.33E-13 |
| LGR4 | -1.15437 | 0.016573 |
| KLF2 | -1.15362 | 0.004202 |
| HIBCH | -1.15362 | 3.68E-06 |
| F3 | -1.15304 | 0.036134 |
| LALBA | -1.15279 | 0.045787 |
| ALS2CL | -1.15275 | 2.33E-21 |
| CABP4 | -1.15251 | 1.91E-22 |
| ATP13A5 | -1.15222 | 0.012267 |
| TMEM80 | -1.15147 | 2.63E-09 |
| PECR | -1.1511 | 1.88E-11 |
| RPL36 | -1.15073 | 5.22E-09 |
| LETMD1 | -1.1506 | 6.04E-08 |
| PIK3CD | -1.14987 | 0.005511 |
| LINC01527 | -1.14983 | 2.45E-27 |
| LINC00888 | -1.1496 | 0.000662 |
| WDR11 | -1.1491 | 0.008839 |
| ZNF575 | -1.14908 | 3.89E-12 |
| UACA | -1.14858 | 7.80E-11 |
| COMMD6 | -1.14845 | 1.37E-09 |
| LRP1 | -1.14746 | 3.25E-06 |
| ARHGAP25 | -1.14742 | 0.005432 |
| LRATD1 | -1.1469 | 0.02671 |
| PRAG1 | -1.14675 | 0.005456 |
| LOC654780 | -1.14589 | 2.61E-14 |
| TMEM220 | -1.14504 | 1.76E-06 |
| RASA3 | -1.1437 | 0.000108 |
| MBD6 | -1.14331 | 0.000195 |
| APOL2 | -1.14272 | 3.41E-07 |
| GNA11 | -1.14266 | 3.54E-12 |
| FUT7 | -1.14242 | 2.45E-18 |
| LONRF1 | -1.14168 | 0.000178 |
| JRKL | -1.14132 | 0.001645 |
| LINC00482 | -1.14108 | 1.34E-27 |
| GRAPL | -1.14042 | 1.07E-08 |
| MADD | -1.13995 | 1.04E-10 |
| CXXC1 | -1.13835 | 3.46E-08 |
| UBAP1L | -1.13828 | 7.85E-22 |
| ANKRD40 | -1.13749 | 2.94E-06 |
| ARHGAP10 | -1.13609 | 1.03E-08 |
| DPP7 | -1.13548 | 1.88E-05 |
| HLA-F-AS1 | -1.1348 | 1.48E-06 |
| ZNF629 | -1.13453 | 2.39E-08 |
| S1PR1 | -1.13429 | 1.87E-06 |
| ATF7IP | -1.13161 | 3.86E-06 |
| ABHD5 | -1.13102 | 8.00E-08 |
| ZNF14 | -1.13078 | 0.001521 |
| XYLT1 | -1.13075 | 0.019762 |
| RAD51B | -1.1307 | 0.000163 |
| RPS6KA5 | -1.13058 | 0.004093 |
| SETMAR | -1.1302 | 0.000217 |
| TP53I13 | -1.12972 | 0.000278 |
| GAK | -1.12962 | 6.15E-06 |
| KLHL24 | -1.12813 | 2.30E-09 |
| APP | -1.12796 | 3.66E-06 |
| SESN1 | -1.12788 | 0.001207 |
| BBS10 | -1.12759 | 4.36E-11 |
| TIAM2 | -1.12686 | 0.002806 |
| YPEL2 | -1.12671 | 0.039508 |
| HIF3A | -1.12619 | 1.21E-14 |
| NFAM1 | -1.12587 | 4.05E-22 |
| C14orf28 | -1.12508 | 5.98E-05 |
| MYMX | -1.12478 | 5.45E-12 |
| IGDCC3 | -1.12472 | 2.21E-05 |
| IDS | -1.12444 | 1.22E-05 |
| CYP27A1 | -1.12424 | 0.000261 |
| CHST2 | -1.12389 | 0.023816 |
| MPRIP | -1.12242 | 9.96E-06 |
| NLGN1 | -1.12241 | 1.06E-06 |
| AS3MT | -1.1219 | 0.00184 |
| TDRD3 | -1.12152 | 2.16E-07 |
| CYP2A13 | -1.12147 | 9.47E-19 |
| SIX5 | -1.12096 | 1.10E-06 |
| ACD | -1.12071 | 2.64E-06 |
| ZNF141 | -1.11971 | 5.67E-06 |
| ZNF793 | -1.11969 | 2.95E-10 |
| PRB1 | -1.11944 | 1.21E-15 |
| ARHGEF10 | -1.11902 | 0.001897 |
| ATP9B | -1.119 | 0.000304 |
| ATP5ME | -1.11887 | 4.05E-08 |
| LINC00184 | -1.11778 | 8.99E-14 |
| PLEKHA2 | -1.1171 | 1.83E-05 |
| LDLRAP1 | -1.1158 | 1.67E-08 |
| NNMT | -1.11577 | 0.036691 |
| AVIL | -1.11549 | 1.18E-07 |
| MTUS1 | -1.11546 | 0.033615 |
| ACVR2B | -1.11519 | 1.13E-06 |
| FAM160A2 | -1.11518 | 1.27E-06 |
| TSR3 | -1.11445 | 5.84E-09 |
| WASF3 | -1.11437 | 0.029715 |
| LRIG1 | -1.114 | 0.027705 |
| CAMLG | -1.11363 | 7.74E-09 |
| ABCD4 | -1.11314 | 4.70E-08 |
| SGSM3 | -1.11208 | 7.12E-08 |
| QSOX1 | -1.11188 | 0.000135 |
| DLGAP4 | -1.11183 | 2.49E-05 |
| FAT4 | -1.11182 | 0.002332 |
| PIWIL4 | -1.11136 | 0.00163 |
| LINC00968 | -1.11135 | 0.00012 |
| CD209 | -1.11041 | 0.000247 |
| GPALPP1 | -1.11001 | 1.15E-05 |
| NIPBL | -1.10865 | 5.50E-10 |
| SNCA | -1.108 | 2.71E-18 |
| LINC02693 | -1.10736 | 3.28E-15 |
| ZNF548 | -1.10713 | 1.41E-07 |
| NDUFV1 | -1.10704 | 1.44E-08 |
| MPHOSPH8 | -1.10678 | 3.33E-08 |
| RFTN1 | -1.10661 | 0.043685 |
| PEAK1 | -1.10534 | 5.36E-06 |
| ACVR2B-AS1 | -1.10497 | 3.15E-15 |
| PLEKHO2 | -1.10464 | 1.84E-06 |
| TSC22D1 | -1.10444 | 2.51E-05 |
| ANGPTL8 | -1.10424 | 5.86E-11 |
| THOC5 | -1.1038 | 2.14E-05 |
| SDC3 | -1.10378 | 0.000966 |
| C5orf63 | -1.10351 | 8.78E-06 |
| EPC2 | -1.10346 | 1.44E-12 |
| KANSL1 | -1.10304 | 1.47E-11 |
| SEMA3F | -1.10271 | 0.000713 |
| SLC12A2 | -1.10262 | 0.03834 |
| DCTN2 | -1.10231 | 2.40E-12 |
| FGFR1OP | -1.10169 | 2.25E-06 |
| NOP53 | -1.10167 | 2.53E-16 |
| DNAJC4 | -1.10156 | 4.91E-09 |
| KCNIP2-AS1 | -1.10145 | 1.15E-24 |
| MR1 | -1.10136 | 0.000721 |
| SMAD5 | -1.10131 | 8.60E-08 |
| GGA2 | -1.1011 | 4.79E-06 |
| GSTZ1 | -1.10066 | 0.000562 |
| FLVCR2 | -1.10037 | 4.83E-17 |
| MMAB | -1.0996 | 0.000252 |
| CSAD | -1.09936 | 0.020148 |
| ALDH7A1 | -1.09907 | 7.16E-05 |
| CACNA1G | -1.09885 | 1.88E-13 |
| CDK5RAP3 | -1.09854 | 3.73E-05 |
| CHD8 | -1.09826 | 1.17E-07 |
| NANOG | -1.09801 | 8.83E-08 |
| TCEA2 | -1.098 | 0.014067 |
| MRNIP | -1.09502 | 7.06E-16 |
| NECTIN3-AS1 | -1.09495 | 1.60E-15 |
| PRX | -1.09443 | 4.42E-14 |
| PLEKHG3 | -1.09415 | 1.98E-06 |
| PEX11G | -1.0939 | 3.96E-11 |
| DGCR2 | -1.09389 | 1.31E-09 |
| ATP9A | -1.09369 | 5.92E-05 |
| NOSIP | -1.09317 | 2.31E-08 |
| KIAA0232 | -1.09301 | 0.000347 |
| PLTP | -1.09263 | 0.04209 |
| ETV5 | -1.09259 | 0.001572 |
| IKZF5 | -1.0919 | 6.99E-06 |
| SLC38A10 | -1.09183 | 2.35E-06 |
| SPATA2L | -1.09177 | 1.17E-06 |
| EIF3G | -1.09062 | 3.33E-09 |
| ARRDC2 | -1.08906 | 3.46E-07 |
| PRDM11 | -1.08797 | 3.89E-10 |
| FAM160B1 | -1.08694 | 2.75E-09 |
| INKA1 | -1.08627 | 2.01E-16 |
| GNL1 | -1.08559 | 5.47E-17 |
| VIPR2 | -1.08548 | 2.14E-18 |
| TDH | -1.08484 | 7.26E-14 |
| SIRPA | -1.08478 | 0.006307 |
| SPAG7 | -1.08434 | 8.01E-12 |
| HNMT | -1.0831 | 0.023856 |
| NOP9 | -1.083 | 3.27E-09 |
| ACVR2A | -1.08273 | 2.09E-06 |
| ESS2 | -1.08258 | 7.32E-22 |
| PHRF1 | -1.08258 | 2.97E-11 |
| SF3A3 | -1.08235 | 1.01E-06 |
| C12orf66 | -1.0822 | 0.003995 |
| EXOSC10 | -1.08202 | 4.03E-09 |
| DVL2 | -1.08138 | 2.90E-06 |
| SLC16A4 | -1.08127 | 0.004575 |
| PARP11 | -1.08119 | 0.000998 |
| ITFG2 | -1.08001 | 4.65E-12 |
| CASTOR2 | -1.07997 | 1.70E-07 |
| INPP5K | -1.07939 | 1.24E-06 |
| CALHM2 | -1.07915 | 0.000373 |
| PHLDA3 | -1.07815 | 0.000362 |
| HSPB6 | -1.07776 | 6.75E-06 |
| MON1B | -1.07763 | 5.77E-08 |
| C1QTNF1 | -1.07725 | 0.000256 |
| PICK1 | -1.07712 | 5.04E-20 |
| EML3 | -1.07665 | 6.51E-09 |
| WFDC9 | -1.07646 | 1.12E-32 |
| SP4 | -1.07636 | 1.28E-05 |
| RIN3 | -1.07596 | 6.81E-07 |
| FAM210B | -1.07533 | 0.000203 |
| AKAP7 | -1.07525 | 0.001372 |
| EPHX2 | -1.07476 | 0.017402 |
| TMUB2 | -1.07459 | 3.91E-07 |
| BLOC1S1 | -1.07404 | 2.09E-10 |
| POLH | -1.07394 | 2.94E-11 |
| MAP3K4 | -1.07385 | 5.22E-07 |
| FUZ | -1.07342 | 6.85E-10 |
| PLXND1 | -1.07336 | 0.004099 |
| TBC1D24 | -1.07303 | 0.003152 |
| YBX3 | -1.07294 | 0.001734 |
| TBRG1 | -1.07276 | 0.001797 |
| CYB5R3 | -1.07271 | 6.57E-12 |
| MKNK2 | -1.07265 | 2.52E-06 |
| NBL1 | -1.07252 | 0.007653 |
| PDZD4 | -1.07228 | 1.32E-09 |
| TFAP2C | -1.07216 | 0.00321 |
| MUC5AC | -1.07167 | 1.83E-13 |
| LINC00597 | -1.07156 | 0.001656 |
| LINC01089 | -1.07153 | 5.89E-30 |
| NPHP3 | -1.07139 | 6.84E-05 |
| FAM71A | -1.07096 | 2.60E-10 |
| CROCCP2 | -1.07029 | 0.000183 |
| PRR29 | -1.07025 | 1.16E-09 |
| CTNNBIP1 | -1.07007 | 3.52E-05 |
| SSBP2 | -1.06982 | 0.004392 |
| ERCC6L2 | -1.06919 | 2.44E-08 |
| FLNB | -1.06819 | 0.003234 |
| STX7 | -1.06815 | 1.97E-07 |
| BHLHE22 | -1.06694 | 0.000843 |
| NEK8 | -1.0669 | 1.11E-05 |
| OGA | -1.06673 | 3.70E-06 |
| NOB1 | -1.06624 | 0.000183 |
| CPEB2 | -1.06535 | 0.022583 |
| FMNL3 | -1.06532 | 0.005013 |
| FARS2 | -1.06511 | 2.04E-09 |
| CDC42BPB | -1.06502 | 8.19E-05 |
| MAN1A1 | -1.06478 | 0.030041 |
| ARHGEF1 | -1.06468 | 3.26E-11 |
| CCDC85B | -1.06461 | 3.26E-06 |
| KLHDC10 | -1.06348 | 6.38E-08 |
| ARMCX6 | -1.06338 | 0.000559 |
| TMOD1 | -1.06298 | 0.004635 |
| MAPK8IP3 | -1.06291 | 1.80E-06 |
| TMEM245 | -1.06244 | 1.19E-08 |
| DGKE | -1.06211 | 0.011682 |
| IGFBP3 | -1.06185 | 0.041507 |
| MIIP | -1.06145 | 5.32E-11 |
| RNASEH2C | -1.06104 | 8.53E-05 |
| FTX | -1.05973 | 0.005962 |
| C6orf47 | -1.05967 | 5.94E-12 |
| CFLAR | -1.05943 | 0.008343 |
| FAM193B | -1.05937 | 1.71E-06 |
| GDPD3 | -1.05915 | 0.019639 |
| ALDH9A1 | -1.05809 | 1.32E-06 |
| HAPLN3 | -1.05766 | 0.011844 |
| GOSR2 | -1.057 | 1.13E-08 |
| TNK2 | -1.05667 | 0.000106 |
| JMY | -1.05588 | 1.14E-05 |
| GPBAR1 | -1.05549 | 4.14E-15 |
| KIAA1324L | -1.05545 | 0.016189 |
| ARHGAP23 | -1.05479 | 0.003128 |
| AMOTL2 | -1.05462 | 0.004045 |
| ZCWPW1 | -1.05454 | 1.20E-10 |
| SPATA7 | -1.05444 | 0.001808 |
| PGM1 | -1.05391 | 0.001708 |
| HSD17B11 | -1.05377 | 0.008854 |
| PLD2 | -1.05359 | 2.57E-11 |
| MDGA1 | -1.05326 | 1.39E-17 |
| NCR2 | -1.05309 | 5.19E-22 |
| CDON | -1.05285 | 0.036609 |
| PPP5D1 | -1.05202 | 5.85E-23 |
| ATG14 | -1.05199 | 8.42E-07 |
| BAZ2B | -1.05194 | 6.37E-06 |
| LRRC56 | -1.05142 | 9.65E-08 |
| MGC70870 | -1.0512 | 9.42E-10 |
| RASL10B | -1.0507 | 5.85E-14 |
| MAPRE3 | -1.05054 | 3.06E-16 |
| PHF7 | -1.05011 | 9.39E-14 |
| AQP2 | -1.0498 | 4.20E-24 |
| TENT5A | -1.04972 | 0.000554 |
| ZNF826P | -1.04906 | 2.25E-07 |
| LAMB1 | -1.04839 | 0.002408 |
| DIS3L | -1.04817 | 1.77E-06 |
| TMEM184A | -1.048 | 1.67E-11 |
| TUBGCP2 | -1.04698 | 7.06E-10 |
| TM4SF1 | -1.04651 | 0.049956 |
| NSMCE1 | -1.04649 | 8.81E-07 |
| LANCL2 | -1.04524 | 0.000706 |
| SFXN3 | -1.04512 | 0.000302 |
| FGFBP2 | -1.04476 | 0.002389 |
| NUDT18 | -1.04458 | 2.47E-07 |
| KCTD17 | -1.04429 | 4.42E-11 |
| LINC02239 | -1.04423 | 3.74E-16 |
| TBX18 | -1.04367 | 2.45E-26 |
| RRAS2 | -1.04337 | 0.027855 |
| ZNF106 | -1.04335 | 4.90E-08 |
| ZNF615 | -1.0433 | 0.000337 |
| PDGFC | -1.04252 | 0.028689 |
| PBLD | -1.04209 | 0.004533 |
| ZNF226 | -1.04204 | 0.001908 |
| EPB41 | -1.04195 | 0.000761 |
| CSMD2-AS1 | -1.04191 | 3.78E-20 |
| CIC | -1.04181 | 8.94E-12 |
| LRCH1 | -1.04139 | 0.00197 |
| PRRX2 | -1.04138 | 1.44E-05 |
| PTOV1-AS2 | -1.04117 | 4.00E-11 |
| ZNF767P | -1.04067 | 7.86E-05 |
| FAM200B | -1.04062 | 1.14E-08 |
| ZNF362 | -1.03988 | 3.34E-06 |
| ZNF559 | -1.03986 | 0.002675 |
| UFSP2 | -1.03976 | 1.77E-09 |
| NUB1 | -1.03976 | 2.13E-06 |
| BRWD3 | -1.03913 | 2.82E-14 |
| ANKRD18A | -1.0391 | 2.39E-16 |
| TECPR1 | -1.03877 | 0.002511 |
| PINX1 | -1.03848 | 5.55E-07 |
| CCDC93 | -1.03846 | 7.79E-07 |
| FBXW12 | -1.03842 | 2.95E-05 |
| TESPA1 | -1.03807 | 7.12E-24 |
| FUBP1 | -1.03778 | 6.18E-06 |
| PNKD | -1.03778 | 1.62E-07 |
| OGG1 | -1.03752 | 4.22E-06 |
| NRG1 | -1.03736 | 8.19E-12 |
| NUDT12 | -1.03594 | 0.014815 |
| LINC01809 | -1.03545 | 6.43E-20 |
| LOC155060 | -1.03357 | 0.006816 |
| GAS8 | -1.03307 | 9.14E-09 |
| NINL | -1.03288 | 0.027131 |
| GGT5 | -1.0325 | 0.001258 |
| VSTM4 | -1.03249 | 1.15E-15 |
| GUSBP1 | -1.03238 | 2.01E-16 |
| MEF2C | -1.03225 | 0.019721 |
| ZBTB48 | -1.03164 | 3.68E-06 |
| SYCP1 | -1.03158 | 2.51E-06 |
| SOCS1 | -1.03045 | 3.53E-23 |
| USP19 | -1.02971 | 1.21E-15 |
| CHD2 | -1.02969 | 0.000102 |
| ASPH | -1.02964 | 0.002538 |
| GGA1 | -1.02921 | 2.78E-08 |
| RAB33B | -1.0292 | 2.54E-07 |
| LZTFL1 | -1.02894 | 0.006304 |
| LPP | -1.02854 | 0.000105 |
| GATD3A | -1.02831 | 1.65E-08 |
| KRBA1 | -1.02822 | 6.42E-10 |
| CGAS | -1.02772 | 5.63E-22 |
| KATNB1 | -1.02695 | 0.008304 |
| AARS2 | -1.02665 | 2.80E-06 |
| CTDSPL | -1.02607 | 9.78E-05 |
| TUSC8 | -1.02592 | 5.59E-11 |
| GNL3 | -1.02549 | 1.33E-05 |
| TEX35 | -1.02527 | 1.24E-18 |
| INTS3 | -1.02514 | 2.46E-05 |
| TRAPPC12 | -1.02507 | 9.65E-10 |
| PCM1 | -1.02485 | 6.25E-05 |
| SYNGR1 | -1.02467 | 0.048098 |
| VSIG2 | -1.02454 | 3.34E-09 |
| C16orf46 | -1.02428 | 0.002558 |
| SELENOM | -1.0236 | 0.01909 |
| S1PR2 | -1.02308 | 1.07E-09 |
| CEP112 | -1.02198 | 5.93E-05 |
| LLGL1 | -1.02181 | 1.97E-07 |
| APOLD1 | -1.02181 | 0.00247 |
| KDR | -1.02079 | 0.001003 |
| PHAX | -1.01991 | 1.44E-08 |
| GKAP1 | -1.01979 | 0.004803 |
| DNAH1 | -1.01908 | 1.17E-27 |
| DHFR2 | -1.01899 | 4.89E-09 |
| CTDNEP1 | -1.01895 | 1.91E-12 |
| PHETA1 | -1.01879 | 5.59E-14 |
| MXI1 | -1.01835 | 0.000927 |
| SPAG8 | -1.01815 | 6.39E-19 |
| PTPN18 | -1.0179 | 3.98E-09 |
| SLC22A3 | -1.0169 | 6.74E-06 |
| CCDC84 | -1.01652 | 2.92E-07 |
| LOC389765 | -1.01602 | 0.001842 |
| CDC42EP2 | -1.01574 | 9.28E-11 |
| SREK1IP1 | -1.01545 | 6.53E-09 |
| PACSIN2 | -1.01524 | 4.06E-06 |
| TMEM117 | -1.01423 | 0.001653 |
| IL22RA2 | -1.0142 | 9.92E-05 |
| FGD4 | -1.01404 | 0.000706 |
| TBXA2R | -1.01366 | 1.64E-09 |
| EPHX1 | -1.01331 | 0.0079 |
| C22orf39 | -1.01303 | 0.000483 |
| IGSF9B | -1.01258 | 6.59E-09 |
| FGD2 | -1.01246 | 0.000183 |
| RIC1 | -1.01143 | 2.63E-05 |
| CREBL2 | -1.01092 | 8.96E-06 |
| SAMHD1 | -1.01051 | 0.001992 |
| AHDC1 | -1.01025 | 5.44E-08 |
| MROH1 | -1.0098 | 0.000464 |
| TMEM259 | -1.00973 | 1.11E-08 |
| SASH1 | -1.0097 | 0.006632 |
| MORN1 | -1.00956 | 1.52E-15 |
| CLECL1 | -1.00936 | 6.63E-14 |
| SLCO3A1 | -1.0092 | 0.003913 |
| C19orf18 | -1.00914 | 0.009582 |
| ZMIZ2 | -1.00903 | 1.60E-05 |
| COL4A2 | -1.00859 | 0.041013 |
| GIT1 | -1.00828 | 5.88E-08 |
| JAG1 | -1.00813 | 0.00296 |
| HSD11B1 | -1.00789 | 0.019909 |
| WDR60 | -1.00767 | 8.69E-05 |
| TMEM129 | -1.00703 | 1.28E-07 |
| ZBTB7A | -1.00694 | 9.73E-08 |
| TOP1MT | -1.00666 | 0.016548 |
| FIS1 | -1.00565 | 2.64E-08 |
| ZNF592 | -1.00542 | 3.42E-12 |
| STARD13 | -1.0053 | 0.024386 |
| KCNK3 | -1.0041 | 5.09E-09 |
| LIPE-AS1 | -1.00291 | 7.53E-22 |
| SUPT6H | -1.00248 | 0.01115 |
| PSMF1 | -1.00224 | 2.74E-06 |
| ARFGAP2 | -1.00201 | 4.48E-07 |
| TTF1 | -1.00176 | 1.21E-05 |
| IP6K1 | -1.00144 | 7.61E-12 |
| MAP1LC3C | -1.00132 | 2.49E-09 |
| SRSF6 | -1.00118 | 1.50E-05 |
| ADCK5 | 1.000087 | 0.001177 |
| HNRNPU | 1.000274 | 9.50E-20 |
| NME7 | 1.001194 | 0.004232 |
| BET1 | 1.001348 | 5.13E-06 |
| ANGPT2 | 1.001695 | 0.019658 |
| SAPCD2 | 1.002751 | 0.018798 |
| TOR3A | 1.002895 | 7.21E-06 |
| UBE2L6 | 1.003041 | 0.013165 |
| ATP10D | 1.003726 | 0.014543 |
| STARD3NL | 1.003963 | 2.45E-06 |
| SYS1 | 1.004412 | 0.000137 |
| PAPSS1 | 1.004459 | 0.000659 |
| SSBP1 | 1.004463 | 1.84E-07 |
| UBE2F | 1.004471 | 1.33E-08 |
| ZMAT3 | 1.004935 | 0.000965 |
| CTSO | 1.005464 | 0.021846 |
| RAB14 | 1.006622 | 1.07E-07 |
| NIPSNAP3A | 1.006843 | 0.000219 |
| VARS1 | 1.006981 | 0.005779 |
| RNF41 | 1.007207 | 7.06E-06 |
| GNA13 | 1.008145 | 2.19E-06 |
| UBE2I | 1.008737 | 3.47E-10 |
| VPS29 | 1.009125 | 3.38E-11 |
| CLN3 | 1.00982 | 4.16E-05 |
| MRPL17 | 1.010322 | 2.41E-07 |
| GRPEL1 | 1.010387 | 5.37E-05 |
| KIFC1 | 1.011192 | 0.013258 |
| ALG2 | 1.011744 | 1.58E-08 |
| IKBIP | 1.012064 | 0.003515 |
| TMEM54 | 1.012788 | 0.002176 |
| CCDC167 | 1.013311 | 6.43E-05 |
| ARMC8 | 1.013761 | 3.48E-07 |
| SNX10 | 1.013831 | 0.027264 |
| PTCD3 | 1.014836 | 6.32E-10 |
| PFN2 | 1.015255 | 0.017412 |
| SRP19 | 1.015305 | 2.46E-12 |
| HENMT1 | 1.015649 | 0.020783 |
| MED20 | 1.015692 | 5.22E-05 |
| BCCIP | 1.016095 | 7.77E-06 |
| ORMDL1 | 1.017093 | 7.23E-09 |
| ZNF8 | 1.01857 | 0.000132 |
| BROX | 1.021099 | 1.47E-08 |
| GPATCH2 | 1.022639 | 5.31E-05 |
| B3GNT2 | 1.023709 | 4.36E-07 |
| UBE2N | 1.023962 | 7.99E-12 |
| PSMA5 | 1.024668 | 6.24E-08 |
| ZNF273 | 1.024696 | 1.46E-05 |
| FNTB | 1.02477 | 0.004167 |
| ABHD2 | 1.025047 | 0.007558 |
| PPP2R5E | 1.025182 | 5.00E-05 |
| AGPAT5 | 1.025604 | 0.004519 |
| TRAPPC1 | 1.027065 | 2.12E-06 |
| TASOR | 1.027987 | 4.50E-08 |
| THAP9-AS1 | 1.030661 | 3.04E-05 |
| SCFD1 | 1.030905 | 7.15E-08 |
| ELF1 | 1.031324 | 2.52E-05 |
| METAP2 | 1.033325 | 3.50E-08 |
| MAPRE1 | 1.033454 | 1.61E-11 |
| PIP4P1 | 1.033599 | 2.12E-05 |
| LRATD2 | 1.03373 | 0.011272 |
| NANP | 1.034087 | 0.000842 |
| GOT1 | 1.03431 | 4.35E-05 |
| CIB1 | 1.035238 | 0.000106 |
| RIOK1 | 1.035294 | 0.005322 |
| SCOC | 1.035307 | 3.15E-08 |
| IVNS1ABP | 1.036276 | 1.36E-06 |
| TAGLN2 | 1.036343 | 8.81E-05 |
| KIAA1549 | 1.036857 | 0.001577 |
| DPH3 | 1.037975 | 0.00016 |
| VANGL1 | 1.039445 | 1.33E-07 |
| ZKSCAN5 | 1.040195 | 0.000346 |
| PPP1CA | 1.040762 | 1.07E-09 |
| YEATS4 | 1.04146 | 0.000577 |
| TMEM33 | 1.04249 | 5.59E-08 |
| GET1 | 1.043234 | 5.74E-06 |
| SELENOS | 1.043455 | 2.84E-07 |
| COG1 | 1.043956 | 0.000554 |
| NF1 | 1.044102 | 6.69E-05 |
| ALG3 | 1.044265 | 3.39E-05 |
| GTPBP4 | 1.044402 | 0.000154 |
| DOCK10 | 1.044993 | 0.010683 |
| POLR3F | 1.046393 | 8.55E-09 |
| ICMT | 1.046611 | 5.92E-06 |
| ATG4A | 1.046686 | 3.09E-05 |
| KAT7 | 1.047644 | 0.005878 |
| PDCD6 | 1.048142 | 1.40E-07 |
| PLGRKT | 1.048433 | 0.000986 |
| TCEAL8 | 1.048715 | 0.004223 |
| TRIB2 | 1.048986 | 0.048713 |
| SLC25A36 | 1.049045 | 1.06E-05 |
| ZSCAN32 | 1.049137 | 6.75E-05 |
| ALDH1L2 | 1.049434 | 0.043599 |
| PDZD11 | 1.049919 | 1.64E-08 |
| SLC49A4 | 1.049979 | 2.03E-05 |
| GGPS1 | 1.05109 | 6.23E-07 |
| AMZ2 | 1.051168 | 3.86E-05 |
| LNPEP | 1.054006 | 0.000656 |
| CHSY3 | 1.054183 | 0.016947 |
| TMEM87B | 1.055659 | 0.001692 |
| PTRH2 | 1.055979 | 0.003741 |
| SLC5A3 | 1.056391 | 0.000144 |
| B4GALT4 | 1.057822 | 4.00E-06 |
| MAGI3 | 1.058507 | 0.022607 |
| RNF8 | 1.05916 | 0.000554 |
| EME1 | 1.059729 | 0.004909 |
| CLTC | 1.061313 | 2.13E-05 |
| TXNDC16 | 1.061316 | 0.002773 |
| UBE2D3 | 1.061501 | 1.16E-12 |
| P2RY2 | 1.062548 | 0.019373 |
| HOOK1 | 1.063004 | 0.015833 |
| AHCTF1 | 1.063261 | 3.07E-07 |
| TUBB | 1.063622 | 6.22E-08 |
| LAMTOR3 | 1.064326 | 1.47E-08 |
| DHX8 | 1.06478 | 3.06E-06 |
| RELCH | 1.066491 | 7.82E-06 |
| MCUB | 1.067034 | 0.000199 |
| SYDE2 | 1.067649 | 0.005049 |
| NHSL1 | 1.067794 | 0.036974 |
| GNB2 | 1.067924 | 2.57E-13 |
| B4GALT3 | 1.068287 | 3.50E-06 |
| NIFK | 1.0693 | 2.79E-06 |
| SDHB | 1.069571 | 6.90E-07 |
| NOP16 | 1.069593 | 0.000728 |
| PRRG4 | 1.069746 | 0.014172 |
| MRGBP | 1.071114 | 8.55E-05 |
| DDX20 | 1.071416 | 3.81E-06 |
| BICDL1 | 1.072349 | 0.009313 |
| PCID2 | 1.072362 | 1.25E-06 |
| SETD9 | 1.072409 | 0.010445 |
| LDAH | 1.072762 | 0.005652 |
| MYO1D | 1.072943 | 0.021053 |
| SLC39A10 | 1.074921 | 1.80E-05 |
| NAGA | 1.075101 | 1.85E-06 |
| SDF2L1 | 1.075518 | 0.000342 |
| INSIG2 | 1.075743 | 7.28E-05 |
| GBP1 | 1.076925 | 0.044026 |
| AMMECR1 | 1.077483 | 0.001668 |
| MCOLN2 | 1.077885 | 0.025198 |
| SPINT2 | 1.077891 | 0.034487 |
| SPG21 | 1.078095 | 7.90E-13 |
| IARS1 | 1.078378 | 2.99E-06 |
| PRKCB | 1.079801 | 0.029053 |
| AGK | 1.082538 | 1.15E-06 |
| ERH | 1.083065 | 1.74E-13 |
| KMT2B | 1.083765 | 7.05E-07 |
| MEAF6 | 1.08401 | 2.71E-07 |
| PPP2R1A | 1.084051 | 2.88E-12 |
| DNAJC19 | 1.08422 | 3.94E-06 |
| RAB1A | 1.084504 | 3.40E-18 |
| SLC3A2 | 1.084643 | 0.000671 |
| GLRX2 | 1.084943 | 4.31E-05 |
| CD46 | 1.086093 | 2.34E-06 |
| FCMR | 1.086577 | 0.049473 |
| GAPDH | 1.086664 | 1.23E-08 |
| CDH2 | 1.087385 | 0.025429 |
| ZFAND3 | 1.088319 | 2.01E-07 |
| DNAJC10 | 1.088385 | 5.87E-08 |
| RHEB | 1.088484 | 1.81E-08 |
| VASP | 1.089776 | 2.50E-07 |
| DDOST | 1.090064 | 2.92E-09 |
| NAMPT | 1.090859 | 0.002536 |
| CPNE3 | 1.090925 | 1.12E-05 |
| COPE | 1.091505 | 3.11E-09 |
| PPP3R1 | 1.092576 | 0.000444 |
| NUFIP2 | 1.092778 | 0.000175 |
| ITPRID2 | 1.092904 | 0.002364 |
| PKM | 1.093001 | 2.06E-06 |
| MRPS6 | 1.093433 | 1.81E-06 |
| RRS1 | 1.093944 | 0.000102 |
| BRAF | 1.094035 | 0.002412 |
| LINC00467 | 1.094224 | 0.009178 |
| DAD1 | 1.095335 | 2.11E-11 |
| GNB4 | 1.096198 | 0.00641 |
| UBE2A | 1.096339 | 8.27E-09 |
| CHUK | 1.096631 | 1.36E-07 |
| TTC13 | 1.096731 | 2.32E-06 |
| FNDC3B | 1.096974 | 0.000115 |
| NDUFS1 | 1.097874 | 3.39E-08 |
| OCRL | 1.098372 | 1.29E-06 |
| TDP1 | 1.098593 | 1.83E-05 |
| KRT8 | 1.098741 | 0.028403 |
| AAGAB | 1.099578 | 1.34E-06 |
| FICD | 1.100773 | 0.000237 |
| ARFGEF2 | 1.10184 | 1.15E-07 |
| DPY19L4 | 1.102001 | 1.55E-05 |
| STX1A | 1.103142 | 0.000707 |
| USP46 | 1.103204 | 3.00E-06 |
| MFSD11 | 1.103531 | 2.00E-05 |
| N4BP1 | 1.103682 | 1.12E-07 |
| CFAP44 | 1.103848 | 0.0164 |
| MGAT2 | 1.104591 | 3.40E-08 |
| SLC39A11 | 1.105181 | 0.011912 |
| RHBDL2 | 1.105636 | 0.017084 |
| RAD21 | 1.10644 | 8.92E-07 |
| SGMS2 | 1.106833 | 0.005879 |
| ERCC6L | 1.107782 | 0.000512 |
| MSL1 | 1.107997 | 0.029167 |
| HEXIM1 | 1.108532 | 5.93E-05 |
| ASAP1 | 1.108698 | 3.21E-06 |
| MSL2 | 1.109116 | 1.45E-08 |
| TLR8 | 1.109259 | 0.0322 |
| NUP160 | 1.1097 | 4.90E-07 |
| RNF13 | 1.110174 | 6.41E-09 |
| ZNF681 | 1.11063 | 1.29E-05 |
| TOB1 | 1.110722 | 0.003043 |
| SPATA5L1 | 1.111536 | 6.46E-08 |
| EAF1 | 1.111723 | 2.90E-09 |
| RAB11A | 1.113905 | 4.11E-11 |
| TERF2 | 1.11452 | 6.07E-06 |
| HS3ST1 | 1.114814 | 0.012717 |
| RPUSD3 | 1.115318 | 0.005244 |
| RAP1GDS1 | 1.11556 | 1.07E-06 |
| ENY2 | 1.116191 | 5.03E-07 |
| GALNT14 | 1.117043 | 0.031604 |
| GMPS | 1.117582 | 0.000585 |
| RPS6KA1 | 1.118097 | 0.003517 |
| CENPA | 1.118566 | 0.014713 |
| WDYHV1 | 1.11867 | 4.98E-05 |
| MLF2 | 1.118886 | 8.37E-06 |
| CMAS | 1.119104 | 0.000405 |
| LCLAT1 | 1.123424 | 3.24E-07 |
| HAUS1 | 1.124192 | 3.48E-05 |
| DUSP10 | 1.124533 | 0.019269 |
| HSPA13 | 1.125502 | 9.96E-07 |
| TLCD3A | 1.125515 | 0.015598 |
| H2AC17 | 1.125716 | 0.008402 |
| MAP3K7 | 1.126333 | 2.53E-10 |
| RAD18 | 1.126992 | 0.000684 |
| HPN | 1.127701 | 0.037204 |
| BTF3L4 | 1.12882 | 7.90E-08 |
| TAF1B | 1.129045 | 6.30E-05 |
| ZNF639 | 1.129187 | 0.000368 |
| UBFD1 | 1.129993 | 2.95E-08 |
| ERMP1 | 1.130408 | 0.004056 |
| SLC52A2 | 1.13102 | 0.000134 |
| PLPBP | 1.131095 | 1.97E-05 |
| MRPS18A | 1.131395 | 6.42E-07 |
| TRAPPC8 | 1.1318 | 5.55E-10 |
| NUDCD2 | 1.132311 | 3.54E-07 |
| MTFMT | 1.134037 | 5.36E-05 |
| KNOP1 | 1.134261 | 8.93E-07 |
| GLIPR1 | 1.134311 | 0.022657 |
| SH3BGRL3 | 1.134558 | 5.89E-09 |
| CAD | 1.136303 | 5.97E-06 |
| LRP12 | 1.136445 | 0.031631 |
| EXOC2 | 1.136979 | 0.00096 |
| STK16 | 1.137859 | 5.21E-06 |
| NNT | 1.138126 | 0.00052 |
| EFNA4 | 1.139165 | 0.000759 |
| CSTF2 | 1.139322 | 2.76E-06 |
| CEP192 | 1.139796 | 1.21E-05 |
| MPLKIP | 1.140953 | 7.58E-09 |
| SUPT4H1 | 1.141006 | 1.24E-06 |
| SPRTN | 1.141168 | 0.000109 |
| CYB561 | 1.141787 | 0.004496 |
| ITK | 1.142202 | 0.03623 |
| PTK6 | 1.142321 | 0.028758 |
| RRN3 | 1.142942 | 5.71E-11 |
| PSMC2 | 1.143046 | 6.11E-14 |
| CCNJ | 1.143113 | 0.000611 |
| EMC9 | 1.144091 | 0.000391 |
| GPBP1L1 | 1.144536 | 2.67E-13 |
| C2 | 1.145317 | 0.030352 |
| IPO9 | 1.14589 | 2.24E-10 |
| PCBD1 | 1.146084 | 0.000106 |
| ETNK2 | 1.146305 | 0.027757 |
| SPRY4 | 1.146442 | 0.021038 |
| PPM1G | 1.146765 | 1.95E-08 |
| MATR3 | 1.147063 | 0.04199 |
| LSM8 | 1.14719 | 1.17E-08 |
| INAFM2 | 1.148577 | 0.002046 |
| ARHGAP30 | 1.148747 | 0.019624 |
| PDIA3 | 1.148801 | 3.49E-08 |
| ARHGEF26 | 1.148978 | 0.04823 |
| DAPK1 | 1.14945 | 0.030857 |
| CUX1 | 1.149483 | 9.65E-07 |
| PRPF4 | 1.150875 | 3.18E-09 |
| OLR1 | 1.151015 | 0.008939 |
| PCTP | 1.151265 | 0.003197 |
| CLEC7A | 1.151927 | 0.032246 |
| GNB1 | 1.152584 | 3.47E-11 |
| FAM49B | 1.153219 | 1.97E-08 |
| SMIM15 | 1.15424 | 3.01E-09 |
| ARPC5 | 1.154438 | 1.37E-11 |
| TRIM14 | 1.156291 | 0.000845 |
| ZMPSTE24 | 1.156578 | 8.51E-12 |
| GCA | 1.156599 | 0.00368 |
| ERGIC1 | 1.158207 | 0.004051 |
| TMEM19 | 1.160199 | 5.21E-06 |
| HSPA9 | 1.160308 | 8.85E-09 |
| SCAF11 | 1.161302 | 1.60E-11 |
| IFIH1 | 1.162098 | 0.026843 |
| NUDT5 | 1.162755 | 6.41E-05 |
| FANCA | 1.163053 | 0.001692 |
| FYB1 | 1.163314 | 0.01437 |
| BCAP29 | 1.164247 | 1.85E-05 |
| YKT6 | 1.164598 | 9.14E-06 |
| HLA-DOB | 1.165128 | 0.047475 |
| SMIM29 | 1.165872 | 1.52E-07 |
| AKAP8L | 1.165956 | 8.85E-06 |
| PRPS1 | 1.166426 | 7.52E-05 |
| CELSR3 | 1.168527 | 0.022585 |
| C12orf49 | 1.16886 | 4.55E-08 |
| EFHD2 | 1.170055 | 7.75E-05 |
| NRBP1 | 1.170093 | 9.41E-14 |
| HBS1L | 1.170868 | 0.000165 |
| RASAL2 | 1.173915 | 1.08E-05 |
| UCHL3 | 1.173929 | 2.15E-05 |
| BASP1 | 1.175125 | 0.031851 |
| TMEM40 | 1.176551 | 0.026256 |
| NARS2 | 1.176551 | 6.87E-07 |
| PSMA2 | 1.177778 | 2.76E-10 |
| MAD2L1BP | 1.178856 | 3.28E-08 |
| KLHL23 | 1.179214 | 0.002177 |
| GTF2B | 1.179422 | 2.69E-09 |
| ARSG | 1.179915 | 0.022718 |
| MOXD1 | 1.180334 | 0.034591 |
| PUDP | 1.181584 | 0.000185 |
| MLLT11 | 1.181794 | 0.012651 |
| IMPA1 | 1.182258 | 2.90E-07 |
| TMEM50A | 1.18275 | 2.36E-13 |
| ACOX1 | 1.183005 | 3.64E-05 |
| SLC30A6 | 1.183943 | 9.13E-10 |
| LEMD2 | 1.184342 | 8.54E-12 |
| RAD54L | 1.184383 | 0.000737 |
| UBE3C | 1.184848 | 3.53E-08 |
| FAM120A | 1.185639 | 2.47E-14 |
| ALG8 | 1.186413 | 1.53E-05 |
| C16orf87 | 1.187027 | 0.001658 |
| KIN | 1.187343 | 5.54E-06 |
| RCOR1 | 1.18758 | 2.42E-08 |
| SPATS2L | 1.188817 | 7.74E-06 |
| PWWP3A | 1.189114 | 0.003555 |
| CD37 | 1.189179 | 0.00437 |
| ADCY7 | 1.189249 | 0.001953 |
| CDK2AP2 | 1.191859 | 7.09E-07 |
| IQCB1 | 1.191982 | 1.56E-06 |
| DNAJC15 | 1.19216 | 0.013207 |
| CDK12 | 1.192435 | 0.020861 |
| LDHA | 1.193211 | 2.05E-09 |
| FASTKD3 | 1.193313 | 6.07E-07 |
| TUFT1 | 1.193671 | 0.000403 |
| ATP6V1D | 1.196141 | 9.72E-10 |
| ND2 | 1.196349 | 4.60E-15 |
| HIRA | 1.196356 | 1.27E-05 |
| GEMIN6 | 1.197055 | 2.72E-07 |
| LINC01094 | 1.197242 | 0.001828 |
| POLM | 1.198128 | 0.000116 |
| FRYL | 1.199348 | 7.17E-09 |
| PPM1H | 1.199366 | 0.029482 |
| ASCL2 | 1.199378 | 0.016189 |
| COPS5 | 1.199473 | 1.61E-08 |
| ACOT13 | 1.200809 | 0.000119 |
| RNF19A | 1.201254 | 1.68E-05 |
| GALNT18 | 1.201334 | 0.00382 |
| MAPRE2 | 1.20322 | 0.001238 |
| CLP1 | 1.203392 | 4.86E-09 |
| MRPL34 | 1.203527 | 8.81E-08 |
| CSTB | 1.204385 | 5.39E-06 |
| EIF2AK3 | 1.20442 | 3.91E-08 |
| GTF2E2 | 1.205048 | 6.72E-05 |
| SMC1A | 1.205534 | 4.50E-09 |
| CASP3 | 1.206492 | 1.72E-12 |
| CFI | 1.206623 | 0.044589 |
| PXMP4 | 1.207812 | 0.017303 |
| COPG1 | 1.208004 | 7.86E-10 |
| NAPSB | 1.208275 | 0.024581 |
| ARFGAP3 | 1.208559 | 2.23E-07 |
| RGS19 | 1.208588 | 1.43E-10 |
| NUP210 | 1.208857 | 0.008973 |
| PCSK1N | 1.208998 | 0.010339 |
| PIGM | 1.209351 | 5.67E-07 |
| ELOC | 1.209777 | 6.02E-08 |
| TNRC6A | 1.209827 | 3.47E-09 |
| TBK1 | 1.210059 | 7.23E-11 |
| KMT5A | 1.210858 | 1.37E-07 |
| NAPG | 1.211718 | 3.77E-07 |
| PRDM10 | 1.212196 | 1.35E-07 |
| CPPED1 | 1.213643 | 0.001448 |
| HNRNPA2B1 | 1.213667 | 9.78E-23 |
| CDT1 | 1.214388 | 0.026455 |
| CMC2 | 1.21445 | 0.000111 |
| ORC2 | 1.216654 | 8.56E-07 |
| GPX8 | 1.216685 | 0.006627 |
| EIF3C | 1.216878 | 0.00117 |
| SLC25A15 | 1.21725 | 5.20E-05 |
| C3orf38 | 1.217593 | 6.46E-11 |
| BLOC1S3 | 1.217653 | 1.26E-05 |
| TNFSF4 | 1.218472 | 0.000991 |
| GOT2 | 1.219372 | 1.51E-07 |
| NUDT1 | 1.219502 | 0.000447 |
| SCO2 | 1.220087 | 3.44E-05 |
| SF3B6 | 1.22027 | 1.95E-10 |
| ANP32A | 1.220429 | 2.98E-05 |
| RSU1 | 1.220569 | 6.74E-06 |
| RABGGTB | 1.221853 | 1.31E-08 |
| FANCD2 | 1.224226 | 0.004796 |
| PRKRA | 1.224271 | 1.17E-11 |
| SRPK1 | 1.225289 | 1.65E-05 |
| HMGB3P1 | 1.226658 | 0.002122 |
| SRGAP2 | 1.226978 | 0.000438 |
| YRDC | 1.227521 | 6.25E-05 |
| EPRS1 | 1.230519 | 1.01E-06 |
| CDK4 | 1.230766 | 5.28E-06 |
| SLC2A6 | 1.232437 | 0.004294 |
| MRPL19 | 1.232672 | 5.42E-09 |
| CNNM4 | 1.233137 | 2.25E-05 |
| HNRNPLL | 1.23438 | 2.72E-07 |
| TPI1 | 1.235264 | 4.72E-06 |
| RAC3 | 1.235467 | 0.000371 |
| DNAJB6 | 1.235789 | 0.000271 |
| SLC2A13 | 1.235978 | 0.007048 |
| CHCHD1 | 1.236417 | 7.91E-10 |
| GADD45B | 1.238004 | 0.000222 |
| ZDHHC13 | 1.238832 | 2.98E-05 |
| PAIP1 | 1.239082 | 1.48E-10 |
| EMC4 | 1.23949 | 8.79E-10 |
| UBXN4 | 1.241401 | 9.57E-12 |
| FAR1 | 1.243632 | 8.63E-05 |
| KDM4A | 1.24518 | 5.59E-06 |
| JAK3 | 1.245402 | 0.025439 |
| FAM214A | 1.247009 | 0.02402 |
| MSMO1 | 1.24708 | 0.000292 |
| RNPEP | 1.247827 | 8.99E-10 |
| ABCG1 | 1.24823 | 0.011256 |
| PRKAG1 | 1.248748 | 3.61E-09 |
| BIVM | 1.249447 | 0.000312 |
| SPA17 | 1.249582 | 0.003692 |
| ANKRD28 | 1.251576 | 1.02E-08 |
| GSKIP | 1.251994 | 1.58E-08 |
| SYNGR2 | 1.252105 | 0.000208 |
| ZNF567 | 1.253311 | 3.39E-06 |
| NR1D2 | 1.253875 | 0.000216 |
| NT5C3A | 1.254793 | 5.54E-07 |
| OVOL1 | 1.255601 | 0.002286 |
| PRMT5 | 1.255845 | 3.52E-08 |
| BTBD19 | 1.255874 | 0.00267 |
| MACROH2A1 | 1.255931 | 8.55E-18 |
| BEND3 | 1.256278 | 0.000475 |
| EVI2A | 1.256699 | 0.020529 |
| MNDA | 1.257457 | 0.030076 |
| RPL39L | 1.259023 | 0.00211 |
| PSMB3 | 1.260667 | 0.000474 |
| MAP4K2 | 1.260802 | 5.70E-06 |
| QTRT2 | 1.26164 | 3.24E-07 |
| ABL2 | 1.262881 | 2.54E-07 |
| RAP2C | 1.26337 | 1.15E-05 |
| SLC12A8 | 1.264367 | 0.00035 |
| PHLPP2 | 1.264805 | 4.29E-06 |
| ARHGEF9 | 1.265361 | 0.004646 |
| CCT5 | 1.266512 | 9.48E-07 |
| RUVBL1 | 1.266602 | 2.71E-06 |
| CDC23 | 1.267498 | 3.29E-10 |
| RAP2B | 1.268116 | 1.79E-08 |
| MAP2K1 | 1.268234 | 7.38E-08 |
| CDCP1 | 1.268338 | 0.018312 |
| CAMK2G | 1.268712 | 1.58E-05 |
| CYBB | 1.268981 | 0.035735 |
| MFSD3 | 1.270851 | 0.000526 |
| PRDM2 | 1.270922 | 9.02E-09 |
| LOC730101 | 1.27147 | 0.020783 |
| AMZ2P1 | 1.271641 | 4.98E-06 |
| TXN | 1.272847 | 1.12E-09 |
| CDCA4 | 1.273127 | 1.32E-05 |
| METTL6 | 1.273325 | 7.48E-05 |
| ARPC2 | 1.274884 | 1.18E-17 |
| SLC35F5 | 1.276233 | 1.89E-08 |
| DIP2B | 1.276935 | 8.14E-09 |
| KLHL12 | 1.277321 | 2.10E-09 |
| HIVEP1 | 1.277786 | 3.62E-09 |
| JPT2 | 1.278133 | 1.44E-10 |
| CCT6A | 1.278239 | 2.37E-08 |
| IPO7 | 1.278759 | 2.03E-12 |
| LACTB | 1.279276 | 2.35E-07 |
| LRRC42 | 1.279888 | 3.60E-06 |
| GGCT | 1.280138 | 4.80E-06 |
| TIFA | 1.280409 | 0.00088 |
| GIMAP2 | 1.281213 | 0.015531 |
| SCRN1 | 1.283821 | 0.005441 |
| PCBP1 | 1.284987 | 3.69E-14 |
| TOR1B | 1.285539 | 6.15E-10 |
| ELP5 | 1.285603 | 0.012523 |
| RINT1 | 1.285953 | 1.05E-09 |
| RTCA | 1.286955 | 1.56E-08 |
| CCNO | 1.287969 | 0.027869 |
| MGAT4A | 1.289299 | 0.015834 |
| DLD | 1.289752 | 1.47E-08 |
| EPCAM | 1.290346 | 0.00871 |
| ARF4 | 1.291066 | 5.58E-15 |
| TMOD3 | 1.29164 | 1.55E-07 |
| TTLL4 | 1.2921 | 0.038106 |
| GALNT3 | 1.292459 | 0.015234 |
| TP53BP2 | 1.293217 | 0.000103 |
| VDAC1 | 1.293762 | 5.78E-13 |
| HSPA5 | 1.293798 | 2.09E-12 |
| OTULIN | 1.293902 | 0.000569 |
| PDCD10 | 1.294655 | 4.98E-12 |
| GOLT1B | 1.295064 | 1.29E-08 |
| DNMT3B | 1.295127 | 0.001257 |
| ECM1 | 1.295999 | 0.045678 |
| ZNF750 | 1.296323 | 0.04066 |
| PRKDC | 1.296505 | 3.60E-06 |
| ARFGEF3 | 1.297287 | 0.030126 |
| ZNF304 | 1.298132 | 9.67E-06 |
| RSRC1 | 1.299987 | 0.000115 |
| PDCD2L | 1.300631 | 0.001041 |
| GPX7 | 1.301294 | 0.022157 |
| NIF3L1 | 1.301924 | 5.27E-10 |
| IRF7 | 1.301984 | 0.009015 |
| CBX4 | 1.302363 | 1.20E-07 |
| PPP1R14B | 1.302428 | 5.19E-08 |
| TMPO | 1.302649 | 1.13E-06 |
| RASSF2 | 1.302802 | 0.019038 |
| C11orf80 | 1.303579 | 0.013725 |
| SNORD89 | 1.305018 | 7.92E-09 |
| BLOC1S2 | 1.305038 | 2.84E-12 |
| CAAP1 | 1.305188 | 3.69E-06 |
| MYDGF | 1.305757 | 8.78E-12 |
| SRC | 1.307423 | 5.68E-05 |
| VEZF1 | 1.309235 | 6.54E-07 |
| FCGR2A | 1.310724 | 0.019909 |
| KDM5B | 1.310862 | 2.07E-08 |
| CDC123 | 1.311386 | 3.53E-07 |
| RCBTB1 | 1.311442 | 7.17E-06 |
| SBK1 | 1.311505 | 3.99E-05 |
| CAPNS1 | 1.311591 | 7.74E-13 |
| PANK3 | 1.312202 | 4.22E-08 |
| PRMT9 | 1.313051 | 3.68E-06 |
| CTXN1 | 1.313053 | 5.29E-05 |
| NUS1 | 1.313934 | 6.80E-07 |
| TMEM123 | 1.314346 | 5.52E-06 |
| ESPL1 | 1.315207 | 0.002008 |
| BPNT1 | 1.316993 | 2.52E-05 |
| MYO5B | 1.317399 | 0.014556 |
| PSMD5 | 1.31742 | 1.68E-08 |
| BACH1 | 1.317669 | 8.05E-08 |
| CDK16 | 1.318299 | 6.66E-06 |
| TMEM260 | 1.3187 | 3.08E-07 |
| C3orf14 | 1.320356 | 0.006021 |
| SNRNP40 | 1.32067 | 2.17E-05 |
| FAM126B | 1.320849 | 1.72E-09 |
| TMED4 | 1.321394 | 5.32E-11 |
| PSMB8 | 1.322798 | 0.01127 |
| HMGB2 | 1.324008 | 7.69E-05 |
| CNOT6 | 1.324066 | 5.12E-10 |
| TUBD1 | 1.324188 | 0.001685 |
| TMEM181 | 1.325157 | 4.66E-06 |
| HCK | 1.325175 | 0.010858 |
| MYO6 | 1.32535 | 0.000208 |
| WDR77 | 1.325381 | 2.74E-06 |
| KDM6A | 1.325816 | 1.96E-07 |
| INTS12 | 1.326376 | 5.28E-09 |
| MFSD14B | 1.326631 | 1.92E-11 |
| CHAMP1 | 1.327541 | 6.36E-07 |
| DDX60 | 1.328499 | 0.005881 |
| EMB | 1.329842 | 0.022768 |
| NIPA1 | 1.331417 | 2.80E-06 |
| MCCC2 | 1.332153 | 0.012065 |
| PILRA | 1.332907 | 0.000703 |
| SLC17A5 | 1.332908 | 1.26E-06 |
| ZCCHC14 | 1.332996 | 1.47E-08 |
| TBCE | 1.333586 | 9.26E-09 |
| CYC1 | 1.333808 | 2.24E-05 |
| FAM91A1 | 1.333998 | 5.12E-10 |
| DNAJC21 | 1.334587 | 1.68E-08 |
| PPRC1 | 1.335288 | 9.34E-10 |
| SKA2 | 1.337066 | 0.000481 |
| RUSC1 | 1.337805 | 2.04E-06 |
| MFSD14A | 1.338413 | 8.30E-12 |
| GABPB1 | 1.33843 | 1.09E-11 |
| PIGW | 1.338952 | 0.000257 |
| ATF6 | 1.339107 | 2.69E-18 |
| PTS | 1.339543 | 1.02E-07 |
| RAB8A | 1.339878 | 6.96E-16 |
| CSPP1 | 1.340436 | 2.61E-06 |
| FOXK2 | 1.340626 | 6.23E-05 |
| TRPM7 | 1.340636 | 2.24E-10 |
| MIF | 1.340917 | 4.76E-12 |
| FAM220A | 1.341604 | 3.86E-12 |
| GBP5 | 1.341606 | 0.040883 |
| FUNDC1 | 1.341759 | 5.71E-06 |
| ARL13B | 1.343954 | 1.08E-06 |
| C8orf76 | 1.344013 | 3.23E-06 |
| RNF138 | 1.344158 | 5.22E-08 |
| G6PD | 1.346681 | 0.00425 |
| CHORDC1 | 1.347632 | 4.97E-08 |
| JADE3 | 1.348682 | 4.74E-05 |
| DNAJA2 | 1.349598 | 2.75E-08 |
| CYBA | 1.350808 | 0.004202 |
| MCM3 | 1.350825 | 1.97E-06 |
| LAP3 | 1.351578 | 1.34E-06 |
| TRIM16 | 1.353675 | 0.014408 |
| GSPT1 | 1.354531 | 3.21E-16 |
| NBPF20 | 1.355189 | 0.000289 |
| TSEN2 | 1.355313 | 1.63E-06 |
| SAR1B | 1.356608 | 6.46E-09 |
| ZBTB8A | 1.356946 | 6.09E-09 |
| PSMD3 | 1.358093 | 0.022794 |
| CCSAP | 1.359031 | 1.68E-06 |
| CISD2 | 1.359566 | 8.07E-09 |
| RFC2 | 1.362684 | 8.34E-07 |
| GDPD1 | 1.365493 | 0.011473 |
| TBC1D5 | 1.367484 | 7.60E-09 |
| EPB41L5 | 1.367896 | 0.00154 |
| CD69 | 1.368126 | 0.003696 |
| POGLUT2 | 1.368191 | 1.49E-05 |
| SNRNP25 | 1.368581 | 3.47E-10 |
| DAGLB | 1.368588 | 4.06E-10 |
| MEN1 | 1.368804 | 4.23E-10 |
| PIK3R4 | 1.369252 | 2.98E-13 |
| SEC22B | 1.369515 | 4.52E-10 |
| NT5DC1 | 1.369964 | 7.36E-06 |
| SNX14 | 1.370108 | 1.56E-08 |
| SLC66A3 | 1.370343 | 2.08E-05 |
| RND1 | 1.371725 | 0.006562 |
| TMEM126B | 1.371885 | 7.60E-08 |
| PEX11B | 1.372813 | 1.09E-09 |
| RTN4IP1 | 1.373331 | 0.005687 |
| MRPS17 | 1.375213 | 2.07E-05 |
| CRNKL1 | 1.375351 | 1.94E-06 |
| SYAP1 | 1.375877 | 4.35E-09 |
| MX1 | 1.376708 | 0.037364 |
| DPM1 | 1.376803 | 1.16E-12 |
| MB | 1.377044 | 0.028631 |
| C1QTNF6 | 1.377773 | 7.83E-05 |
| PRDX1 | 1.379289 | 6.35E-13 |
| PPIL1 | 1.380214 | 1.53E-07 |
| EIF2S2 | 1.380944 | 3.52E-10 |
| OLFML2B | 1.381137 | 0.030918 |
| SH3GLB1 | 1.381723 | 2.78E-11 |
| RFX5 | 1.382067 | 1.09E-06 |
| ZNF200 | 1.383188 | 2.48E-09 |
| DDX55 | 1.384014 | 3.81E-09 |
| GTF2H4 | 1.384846 | 3.55E-07 |
| EDEM3 | 1.385762 | 3.52E-11 |
| RNASE6 | 1.385859 | 0.025192 |
| CYP20A1 | 1.38793 | 1.07E-08 |
| PAPOLG | 1.388215 | 3.86E-10 |
| PLCH1 | 1.388532 | 0.008463 |
| FAM117B | 1.389012 | 0.000193 |
| PIGO | 1.389319 | 3.19E-07 |
| POLR2J4 | 1.389656 | 4.13E-08 |
| TMEM267 | 1.389749 | 1.13E-05 |
| PREX1 | 1.389955 | 0.012401 |
| TANC2 | 1.391304 | 0.009639 |
| CTSC | 1.391402 | 0.000875 |
| ATP1B1 | 1.393076 | 0.001261 |
| DNAJC16 | 1.393119 | 4.16E-07 |
| RIOK2 | 1.393611 | 1.30E-11 |
| MTERF3 | 1.394041 | 3.84E-07 |
| ACAD8 | 1.394547 | 3.69E-05 |
| NUBP1 | 1.394552 | 1.01E-12 |
| TTLL7 | 1.395135 | 0.003599 |
| RAP2A | 1.396233 | 1.75E-07 |
| TEX30 | 1.396751 | 0.000403 |
| ARL6IP1 | 1.39693 | 3.20E-14 |
| GINS3 | 1.396985 | 0.000736 |
| DSN1 | 1.398611 | 4.18E-07 |
| LOXL1 | 1.398899 | 0.018057 |
| NPL | 1.399321 | 0.000137 |
| OSBPL6 | 1.399624 | 0.028736 |
| HS2ST1 | 1.39994 | 1.30E-07 |
| SCCPDH | 1.400351 | 0.003116 |
| TRRAP | 1.400355 | 5.09E-08 |
| MPC2 | 1.401485 | 3.08E-07 |
| PPM1D | 1.401764 | 0.000212 |
| CD48 | 1.401794 | 0.049029 |
| THEM6 | 1.405441 | 0.00448 |
| NCAPD3 | 1.40565 | 1.91E-08 |
| LYPLA1 | 1.408896 | 2.71E-08 |
| ATP7B | 1.411498 | 0.046467 |
| FTSJ1 | 1.412063 | 2.85E-14 |
| PPP1R11 | 1.412086 | 1.30E-14 |
| GEN1 | 1.412483 | 4.75E-07 |
| PSMD14 | 1.41436 | 6.42E-12 |
| STK17A | 1.414612 | 4.26E-06 |
| LLGL2 | 1.414847 | 0.001106 |
| TSC22D2 | 1.416466 | 4.36E-13 |
| LSM12 | 1.416693 | 2.71E-09 |
| GDAP2 | 1.417273 | 2.67E-07 |
| SIX2 | 1.41891 | 0.009354 |
| NUP205 | 1.41999 | 1.55E-06 |
| SPAG9 | 1.421572 | 1.78E-06 |
| ABHD3 | 1.421977 | 3.60E-08 |
| CTSV | 1.422085 | 0.006438 |
| PPIL3 | 1.422866 | 4.33E-06 |
| CERS6 | 1.423811 | 0.001426 |
| PLXNB1 | 1.423852 | 0.000202 |
| KIF3C | 1.42392 | 0.000904 |
| TYMP | 1.42453 | 0.002533 |
| DNAJA1 | 1.425403 | 7.28E-13 |
| CSE1L | 1.426751 | 4.12E-11 |
| EFR3A | 1.427872 | 4.02E-12 |
| TBC1D30 | 1.428513 | 0.015891 |
| C3orf80 | 1.428714 | 3.15E-05 |
| DAPK1-IT1 | 1.429029 | 0.000964 |
| JAK2 | 1.429359 | 0.003234 |
| PHB | 1.430001 | 1.94E-05 |
| SH3TC1 | 1.430102 | 0.000139 |
| LOC728613 | 1.430132 | 0.021556 |
| LINC01000 | 1.430518 | 3.15E-06 |
| FAM149B1 | 1.430894 | 3.12E-05 |
| COA6 | 1.432972 | 2.75E-09 |
| CYCS | 1.433211 | 2.44E-11 |
| SLC19A1 | 1.433589 | 9.96E-05 |
| COMMD5 | 1.433922 | 1.19E-07 |
| C2orf42 | 1.43415 | 6.58E-07 |
| UBAP2L | 1.435446 | 2.88E-12 |
| SH3GLB2 | 1.4375 | 0.00013 |
| ANKRD10-IT1 | 1.437826 | 0.001269 |
| OAZ3 | 1.437832 | 0.002385 |
| SPATS2 | 1.438091 | 3.77E-11 |
| SRXN1 | 1.438316 | 2.23E-05 |
| TMEM65 | 1.439835 | 1.37E-05 |
| HILPDA | 1.440242 | 0.001316 |
| CDC25C | 1.440493 | 0.000586 |
| GIGYF2 | 1.441341 | 4.11E-10 |
| ALAS1 | 1.441419 | 2.23E-08 |
| NINJ2 | 1.441797 | 0.001106 |
| CBFB | 1.442315 | 5.06E-10 |
| GRTP1 | 1.443005 | 0.000186 |
| CYTIP | 1.445432 | 0.01424 |
| CEP76 | 1.445701 | 6.56E-07 |
| ACTR3 | 1.445878 | 1.72E-17 |
| PIAS3 | 1.446449 | 4.15E-05 |
| CDK6 | 1.446936 | 0.012301 |
| DMXL2 | 1.447456 | 1.32E-07 |
| SLC38A6 | 1.447947 | 5.48E-07 |
| XPOT | 1.448224 | 5.48E-11 |
| TRAPPC3 | 1.448735 | 1.25E-11 |
| C5AR1 | 1.449193 | 0.000499 |
| HSPH1 | 1.45415 | 3.51E-09 |
| IL32 | 1.456043 | 0.017277 |
| RNF19B | 1.456141 | 2.10E-07 |
| INIP | 1.457181 | 4.98E-13 |
| RUNX2 | 1.459094 | 0.003378 |
| TFB2M | 1.459196 | 1.10E-10 |
| GPD2 | 1.459746 | 1.96E-08 |
| ARG2 | 1.459783 | 0.0117 |
| SLC30A5 | 1.459971 | 6.77E-12 |
| AKT1 | 1.461446 | 1.62E-05 |
| HSPA6 | 1.462938 | 0.007048 |
| GCLC | 1.463075 | 2.03E-08 |
| WASHC5 | 1.464363 | 1.90E-09 |
| ID2 | 1.465391 | 9.25E-05 |
| PLD3 | 1.465568 | 1.05E-08 |
| ZKSCAN8 | 1.465599 | 4.60E-05 |
| FDX1 | 1.466841 | 3.09E-06 |
| TMTC4 | 1.467617 | 0.002073 |
| PRMT2 | 1.468872 | 2.32E-08 |
| CDKAL1 | 1.469667 | 0.005952 |
| JUNB | 1.470175 | 7.06E-06 |
| LOC202181 | 1.471862 | 8.91E-05 |
| MARK2 | 1.471909 | 1.09E-11 |
| CNOT11 | 1.47256 | 4.84E-12 |
| CAMSAP3 | 1.477091 | 6.79E-06 |
| EMC2 | 1.477183 | 1.46E-11 |
| CD38 | 1.477734 | 0.037231 |
| IL2RG | 1.479464 | 0.037297 |
| CHST11 | 1.48008 | 0.000543 |
| PRSS23 | 1.48038 | 0.002082 |
| PGAP6 | 1.481193 | 4.54E-07 |
| ENO2 | 1.482174 | 0.026256 |
| BLMH | 1.482223 | 0.000103 |
| MRPS23 | 1.482789 | 2.53E-05 |
| TMEM248 | 1.482904 | 3.38E-18 |
| FLVCR1 | 1.483517 | 9.12E-08 |
| MICAL2 | 1.483658 | 8.10E-05 |
| ZNF514 | 1.4845 | 0.000219 |
| TCTN3 | 1.484516 | 7.68E-10 |
| SLC31A1 | 1.484907 | 2.85E-07 |
| LMNB2 | 1.485141 | 8.80E-06 |
| RAB18 | 1.487793 | 7.28E-13 |
| USP31 | 1.491106 | 1.12E-06 |
| STMP1 | 1.491575 | 1.51E-11 |
| POLQ | 1.493156 | 0.000658 |
| PRIMPOL | 1.493893 | 0.000147 |
| PPP6R3 | 1.494153 | 1.03E-14 |
| ORC5 | 1.495667 | 6.44E-10 |
| SPAG4 | 1.496386 | 0.006855 |
| TBC1D8B | 1.497335 | 1.69E-05 |
| AUNIP | 1.499343 | 0.002728 |
| GINS4 | 1.501186 | 0.005292 |
| DENND1B | 1.501733 | 6.02E-06 |
| NECAP1 | 1.503337 | 1.64E-08 |
| WTAP | 1.50399 | 7.37E-11 |
| EIF2AK2 | 1.50459 | 1.41E-08 |
| SLC2A5 | 1.505349 | 0.00218 |
| NME6 | 1.505444 | 2.02E-11 |
| SF3B4 | 1.506154 | 8.60E-10 |
| RNFT1 | 1.506349 | 0.00012 |
| FPGT | 1.508476 | 3.53E-12 |
| UBE2J1 | 1.509284 | 4.39E-11 |
| NAA20 | 1.509454 | 4.33E-13 |
| JPH1 | 1.510911 | 0.009689 |
| BCAT1 | 1.511368 | 0.008844 |
| STK26 | 1.512418 | 0.012504 |
| KRAS | 1.513062 | 4.76E-13 |
| RAI14 | 1.513171 | 1.43E-06 |
| HCCS | 1.513369 | 9.88E-06 |
| MRPS28 | 1.513588 | 6.16E-09 |
| GRSF1 | 1.51366 | 2.37E-13 |
| AGPS | 1.51383 | 0.000443 |
| AGO3 | 1.514546 | 7.66E-08 |
| PDIA6 | 1.514657 | 1.17E-10 |
| OCLN | 1.515038 | 0.000196 |
| CENPI | 1.516855 | 0.000148 |
| GORAB | 1.516972 | 9.67E-12 |
| COPB2 | 1.517362 | 7.57E-17 |
| CHCHD7 | 1.517393 | 5.70E-06 |
| PHKA1 | 1.518813 | 1.99E-06 |
| IPPK | 1.519388 | 2.32E-10 |
| TASOR2 | 1.519638 | 1.32E-07 |
| SRP54 | 1.52246 | 5.93E-15 |
| CHD1 | 1.522673 | 2.37E-13 |
| POLR2D | 1.522928 | 1.51E-08 |
| TTF2 | 1.527652 | 2.39E-07 |
| RNF34 | 1.527653 | 6.24E-12 |
| MTA3 | 1.528434 | 3.71E-10 |
| WSB2 | 1.532125 | 1.55E-12 |
| HMOX2 | 1.533241 | 6.90E-10 |
| GZMA | 1.534374 | 0.035449 |
| WNT5A | 1.536049 | 0.012707 |
| ST6GAL2 | 1.53671 | 0.009273 |
| CENPL | 1.536753 | 7.19E-05 |
| CCL8 | 1.537983 | 0.044696 |
| ODR4 | 1.538477 | 1.03E-16 |
| SLC1A4 | 1.539563 | 0.004098 |
| DNMT3A | 1.539851 | 2.91E-05 |
| SRD5A1 | 1.540362 | 0.030532 |
| TRIM32 | 1.540606 | 9.93E-09 |
| CCDC47 | 1.542046 | 5.09E-08 |
| ZNF43 | 1.542941 | 1.40E-05 |
| PDHX | 1.543154 | 3.21E-10 |
| PDIA4 | 1.543413 | 1.18E-13 |
| MSR1 | 1.543758 | 0.001422 |
| QRSL1 | 1.543868 | 3.60E-05 |
| SLC35F2 | 1.544665 | 0.000658 |
| ATP6V1B2 | 1.545009 | 9.28E-11 |
| SLC35A2 | 1.545328 | 1.14E-10 |
| PTRH1 | 1.546285 | 1.32E-06 |
| NEDD4 | 1.547326 | 0.000429 |
| DGKA | 1.550915 | 2.25E-05 |
| PYCR3 | 1.552796 | 7.24E-05 |
| ULBP2 | 1.552896 | 0.039451 |
| ZYG11A | 1.553011 | 0.003359 |
| TIMM17B | 1.555794 | 9.42E-09 |
| ALG6 | 1.5564 | 2.59E-06 |
| NVL | 1.556728 | 1.08E-08 |
| BARD1 | 1.557366 | 7.59E-08 |
| C1orf53 | 1.557452 | 0.007845 |
| EPYC | 1.559074 | 0.032522 |
| WNK1 | 1.559754 | 6.84E-11 |
| TPGS2 | 1.559813 | 1.56E-08 |
| ANKRD39 | 1.56091 | 4.38E-05 |
| IGSF9 | 1.562783 | 0.002472 |
| TMEM165 | 1.56312 | 4.03E-16 |
| ANKIB1 | 1.563371 | 1.71E-14 |
| ZNF451 | 1.563517 | 2.43E-11 |
| RAB26 | 1.563537 | 0.015329 |
| SPRED2 | 1.563558 | 0.007294 |
| LOXL2 | 1.565101 | 0.001364 |
| MCM6 | 1.565263 | 1.70E-06 |
| SYNCRIP | 1.565278 | 3.54E-09 |
| AIM2 | 1.565881 | 0.013629 |
| PTPRC | 1.566314 | 0.02914 |
| STC1 | 1.567835 | 0.039312 |
| CIAO2A | 1.568755 | 2.29E-17 |
| ADAMTS2 | 1.568859 | 0.034055 |
| PATL1 | 1.570256 | 1.14E-09 |
| AQR | 1.571255 | 8.88E-13 |
| ANXA9 | 1.571951 | 0.034948 |
| GSK3B | 1.572987 | 7.48E-18 |
| MED1 | 1.573865 | 0.000982 |
| UBE2L3 | 1.573931 | 4.39E-17 |
| MRPL35 | 1.575204 | 4.51E-13 |
| PSMA3 | 1.577142 | 7.64E-13 |
| TADA1 | 1.577172 | 4.75E-10 |
| EHMT1 | 1.578188 | 9.69E-09 |
| DDX58 | 1.578379 | 0.004184 |
| ARIH2 | 1.579552 | 1.22E-12 |
| TMEM14A | 1.579669 | 1.69E-10 |
| SERPINE1 | 1.580064 | 0.002791 |
| DCPS | 1.580245 | 9.97E-07 |
| TXNRD1 | 1.580714 | 5.48E-07 |
| MITD1 | 1.580808 | 4.53E-14 |
| BAMBI | 1.582006 | 0.022456 |
| CUL1 | 1.582645 | 1.06E-14 |
| GNPDA2 | 1.584948 | 4.91E-06 |
| PAICS | 1.586849 | 4.74E-10 |
| RAD23B | 1.586933 | 3.12E-20 |
| MORN2 | 1.587568 | 3.12E-08 |
| CEMIP2 | 1.588069 | 5.22E-09 |
| MOCOS | 1.588084 | 0.011788 |
| TMEM97 | 1.591715 | 0.002852 |
| NCOA3 | 1.591719 | 6.04E-08 |
| CAPRIN1 | 1.59208 | 2.33E-16 |
| MS4A6A | 1.592179 | 0.027555 |
| TFEC | 1.59535 | 0.005514 |
| RNFT2 | 1.595943 | 0.000827 |
| HAT1 | 1.596022 | 4.58E-17 |
| OXSR1 | 1.597156 | 5.33E-12 |
| IRF9 | 1.597226 | 3.10E-05 |
| TRMT11 | 1.598103 | 2.36E-06 |
| EIPR1 | 1.598426 | 1.67E-08 |
| USP14 | 1.598582 | 6.53E-16 |
| WDR12 | 1.601023 | 4.61E-08 |
| TXLNA | 1.601604 | 2.93E-11 |
| PIGH | 1.601673 | 1.43E-08 |
| NFKB1 | 1.602532 | 1.56E-10 |
| GMPR2 | 1.602918 | 5.55E-08 |
| MINPP1 | 1.604385 | 1.27E-06 |
| LYRM2 | 1.605355 | 2.62E-11 |
| MAD2L2 | 1.607033 | 1.20E-07 |
| CASD1 | 1.607869 | 0.000329 |
| UBE2V2 | 1.608242 | 1.99E-11 |
| GEMIN7 | 1.608534 | 5.68E-10 |
| XRCC4 | 1.60924 | 2.51E-09 |
| PSME3 | 1.609519 | 5.35E-08 |
| UHRF1BP1 | 1.611389 | 8.23E-13 |
| OSTF1 | 1.613053 | 1.25E-10 |
| MST1R | 1.613091 | 0.000271 |
| FECH | 1.614337 | 2.04E-06 |
| HSPE1 | 1.616925 | 5.04E-13 |
| TM9SF2 | 1.61716 | 5.08E-14 |
| CHEK2 | 1.61809 | 0.000105 |
| RANBP1 | 1.618156 | 9.06E-09 |
| IFIT3 | 1.618794 | 0.008589 |
| PLS1 | 1.619865 | 0.010118 |
| MTCH2 | 1.620371 | 4.70E-13 |
| RRP15 | 1.620505 | 1.07E-08 |
| TMEM200A | 1.621359 | 0.003326 |
| VPS13A | 1.622824 | 2.33E-09 |
| SMG1P5 | 1.624339 | 0.000509 |
| MRPS7 | 1.62476 | 1.53E-08 |
| XPO7 | 1.624773 | 2.40E-09 |
| ZNF195 | 1.625302 | 2.52E-08 |
| RPAP3 | 1.625652 | 1.06E-13 |
| GANAB | 1.625861 | 2.03E-12 |
| NCAPG2 | 1.626734 | 5.64E-06 |
| COTL1 | 1.626873 | 0.000164 |
| ARPC1A | 1.627776 | 1.46E-11 |
| STAU2 | 1.629208 | 7.33E-07 |
| MBOAT2 | 1.630244 | 4.41E-06 |
| MARCKSL1 | 1.631295 | 4.01E-08 |
| COCH | 1.632245 | 5.74E-11 |
| STMN1 | 1.632662 | 0.000118 |
| SRP9 | 1.632782 | 5.46E-26 |
| RPP38 | 1.634399 | 5.79E-09 |
| LILRB4 | 1.636483 | 0.000377 |
| CASP8 | 1.636499 | 5.58E-10 |
| PLEKHA8 | 1.636777 | 1.13E-08 |
| STAM2 | 1.636792 | 1.04E-14 |
| SLC50A1 | 1.639673 | 1.02E-12 |
| USP18 | 1.640682 | 0.000641 |
| COPA | 1.641003 | 5.33E-19 |
| APOO | 1.641144 | 9.38E-08 |
| CCDC117 | 1.643123 | 5.93E-09 |
| SLF2 | 1.643541 | 5.00E-11 |
| TMEM158 | 1.643639 | 0.025535 |
| UBE2Q1 | 1.643652 | 2.61E-15 |
| TMCO1 | 1.645308 | 4.11E-18 |
| CCDC125 | 1.645688 | 0.00153 |
| WDR26 | 1.645988 | 1.22E-17 |
| ATG5 | 1.647581 | 1.34E-06 |
| MRRF | 1.648938 | 2.81E-09 |
| TMEM209 | 1.649526 | 8.77E-11 |
| SPTLC2 | 1.649985 | 0.000577 |
| GTPBP2 | 1.650548 | 8.03E-09 |
| GTF3C3 | 1.653059 | 1.06E-10 |
| FCGR2C | 1.654021 | 0.000137 |
| ZNF746 | 1.654804 | 3.19E-13 |
| S100A11 | 1.655854 | 1.79E-11 |
| FYTTD1 | 1.656331 | 6.33E-15 |
| NAA15 | 1.656796 | 1.21E-10 |
| LSM14B | 1.656911 | 3.75E-09 |
| FEN1 | 1.657573 | 1.10E-05 |
| ARNTL2 | 1.65765 | 0.017432 |
| HSPA14 | 1.658772 | 3.33E-08 |
| DPY30 | 1.658821 | 2.39E-16 |
| MCM9 | 1.662959 | 8.21E-10 |
| RPP40 | 1.66309 | 0.000131 |
| PARP14 | 1.663318 | 2.65E-05 |
| TRIB3 | 1.663627 | 0.001773 |
| CDCA2 | 1.664268 | 0.003182 |
| CTSZ | 1.665248 | 1.86E-06 |
| EIF4G3 | 1.666709 | 3.23E-06 |
| SOX9 | 1.66923 | 0.005151 |
| PAFAH1B3 | 1.670087 | 1.18E-10 |
| CYB5B | 1.671048 | 7.89E-08 |
| PPP2CA | 1.671965 | 2.41E-18 |
| SMG8 | 1.673057 | 1.75E-06 |
| TSPAN1 | 1.67337 | 0.03 |
| ST6GALNAC5 | 1.673427 | 0.02754 |
| PLPP5 | 1.673587 | 2.07E-06 |
| ERC1 | 1.673917 | 0.000228 |
| OSGIN2 | 1.676469 | 4.77E-05 |
| TOPBP1 | 1.67648 | 1.94E-12 |
| SLC44A2 | 1.67662 | 7.12E-07 |
| SLC20A1 | 1.676879 | 1.77E-15 |
| PLAA | 1.677055 | 5.39E-07 |
| SEC24D | 1.677104 | 2.49E-07 |
| EIF2AK1 | 1.677953 | 1.04E-21 |
| TET3 | 1.678188 | 7.86E-11 |
| DNAJC14 | 1.678472 | 7.19E-10 |
| IGK | 1.681213 | 0.010453 |
| NME1 | 1.68369 | 1.68E-06 |
| H4C8 | 1.684129 | 0.008408 |
| BHLHE40 | 1.685785 | 5.16E-06 |
| CEP85 | 1.685878 | 8.50E-09 |
| TCFL5 | 1.686591 | 3.52E-08 |
| RAN | 1.687161 | 3.25E-15 |
| PPP4R3A | 1.687468 | 3.00E-16 |
| ARNT2 | 1.687771 | 0.009485 |
| CDC73 | 1.689306 | 2.55E-14 |
| DENND11 | 1.690405 | 5.96E-09 |
| APTR | 1.690989 | 1.09E-07 |
| SPATA5 | 1.69121 | 1.42E-06 |
| PLK4 | 1.691348 | 0.000457 |
| ITGB2 | 1.692134 | 0.006355 |
| CUEDC1 | 1.696464 | 0.000945 |
| LCP2 | 1.696583 | 0.002884 |
| DACT1 | 1.697668 | 0.008291 |
| MLLT10 | 1.698555 | 1.36E-09 |
| PARP1 | 1.699056 | 7.11E-15 |
| TMEM132A | 1.699165 | 2.38E-05 |
| SKIL | 1.700185 | 3.69E-11 |
| MS4A4A | 1.701869 | 0.016358 |
| FCER1G | 1.703236 | 0.016609 |
| SKA3 | 1.703839 | 3.73E-05 |
| TRAM2 | 1.703863 | 2.39E-08 |
| H2AZ1 | 1.70446 | 8.13E-14 |
| EXOSC3 | 1.704663 | 2.41E-10 |
| ZBTB21 | 1.70502 | 1.54E-09 |
| IMPAD1 | 1.705945 | 1.26E-08 |
| EGLN3 | 1.707611 | 0.014199 |
| PLP2 | 1.708481 | 8.59E-10 |
| DNMT1 | 1.708741 | 1.05E-11 |
| CRIP2 | 1.708947 | 0.003021 |
| COG2 | 1.710697 | 5.66E-13 |
| DBNDD1 | 1.713707 | 4.06E-05 |
| SLC35D1 | 1.715302 | 7.74E-09 |
| RFC5 | 1.715511 | 7.65E-13 |
| DNPH1 | 1.716547 | 1.10E-08 |
| ZDHHC4 | 1.717169 | 2.06E-05 |
| TMTC3 | 1.723929 | 1.22E-09 |
| FZD6 | 1.725042 | 0.001672 |
| MAD1L1 | 1.725585 | 5.77E-08 |
| TPM3 | 1.725935 | 5.39E-26 |
| RIPK2 | 1.728254 | 1.99E-06 |
| RBBP5 | 1.728287 | 3.73E-13 |
| NID2 | 1.731442 | 0.0109 |
| BPGM | 1.732107 | 5.02E-09 |
| CDKN2A | 1.732464 | 0.013367 |
| AIFM1 | 1.733142 | 1.02E-09 |
| ISCA2 | 1.733986 | 2.53E-11 |
| TAF1A | 1.735308 | 1.01E-08 |
| KCTD3 | 1.735442 | 2.00E-08 |
| GTF2I | 1.735457 | 1.62E-08 |
| TMEM251 | 1.740923 | 1.46E-11 |
| ADNP | 1.741924 | 4.46E-21 |
| BAX | 1.744919 | 5.11E-10 |
| STIL | 1.745581 | 1.55E-05 |
| TUBG1 | 1.745789 | 1.21E-09 |
| SOCS3 | 1.748944 | 5.35E-05 |
| RUNX1-IT1 | 1.749101 | 1.30E-05 |
| H2AC8 | 1.749432 | 0.009215 |
| SLC35A3 | 1.749638 | 1.47E-08 |
| RAB3IP | 1.752516 | 2.45E-05 |
| ABT1 | 1.753603 | 4.27E-12 |
| EFNA5 | 1.753902 | 0.014868 |
| ENAH | 1.754172 | 4.69E-14 |
| LSR | 1.755123 | 2.36E-06 |
| MRS2 | 1.755345 | 3.69E-15 |
| AGFG1 | 1.75558 | 2.54E-11 |
| RITA1 | 1.755756 | 3.33E-12 |
| STAT1 | 1.756932 | 3.22E-06 |
| STIP1 | 1.757105 | 3.99E-17 |
| MAGOHB | 1.757941 | 6.01E-06 |
| KDM1B | 1.760469 | 3.30E-09 |
| RHNO1 | 1.76391 | 3.78E-07 |
| INTS6 | 1.764254 | 1.69E-10 |
| RPP14 | 1.765081 | 5.30E-13 |
| CHROMR | 1.765104 | 6.75E-06 |
| PRMT1 | 1.766354 | 9.95E-12 |
| ADIPOR1 | 1.767428 | 9.95E-18 |
| NFKB2 | 1.768465 | 1.74E-09 |
| SPPL2A | 1.768971 | 2.31E-12 |
| SELL | 1.769638 | 0.005907 |
| SDS | 1.770795 | 7.68E-07 |
| BRPF3 | 1.771559 | 2.33E-08 |
| PPP1R15B | 1.772283 | 4.69E-21 |
| YIF1B | 1.774321 | 2.68E-09 |
| URI1 | 1.774694 | 2.66E-11 |
| POLR1B | 1.775713 | 2.63E-10 |
| DNAH14 | 1.776374 | 0.000232 |
| STT3A | 1.777706 | 1.68E-11 |
| FGD6 | 1.777711 | 7.34E-06 |
| ARL2BP | 1.777733 | 4.06E-12 |
| PGK1 | 1.779074 | 1.66E-14 |
| EFCAB11 | 1.779815 | 3.00E-05 |
| TENT2 | 1.780342 | 5.61E-07 |
| NDFIP2 | 1.782613 | 1.39E-08 |
| SETX | 1.783883 | 3.01E-10 |
| MED10 | 1.786115 | 7.70E-08 |
| ESRP1 | 1.786391 | 0.001828 |
| HES6 | 1.789382 | 3.70E-05 |
| RBM12 | 1.791681 | 3.26E-29 |
| GMCL1 | 1.79384 | 1.19E-12 |
| RPAP2 | 1.79441 | 1.76E-12 |
| GSE1 | 1.794534 | 1.32E-06 |
| CELSR1 | 1.795289 | 0.004514 |
| CHFR | 1.797774 | 6.32E-10 |
| PLSCR1 | 1.798147 | 1.97E-05 |
| DRG2 | 1.799906 | 3.87E-11 |
| IFI44 | 1.800148 | 0.002712 |
| ZBTB38 | 1.801852 | 6.35E-11 |
| ATP6V1G1 | 1.802202 | 1.05E-14 |
| TACO1 | 1.803487 | 3.68E-06 |
| SNAPC3 | 1.803669 | 7.42E-08 |
| COQ10B | 1.804037 | 1.72E-14 |
| ARV1 | 1.804584 | 2.16E-14 |
| PACRGL | 1.805063 | 2.82E-07 |
| CABLES2 | 1.80689 | 1.56E-07 |
| ATRX | 1.80689 | 2.47E-13 |
| DPCD | 1.809439 | 1.72E-10 |
| COL5A2 | 1.810255 | 0.015964 |
| SNRPB | 1.810422 | 1.91E-13 |
| SRFBP1 | 1.810732 | 2.91E-13 |
| ZBTB1 | 1.811999 | 4.06E-12 |
| NMU | 1.812392 | 0.005128 |
| YDJC | 1.813683 | 1.94E-12 |
| GPR137B | 1.814279 | 2.68E-05 |
| VPS26C | 1.814731 | 3.54E-14 |
| NRIP1 | 1.815202 | 6.02E-06 |
| ATP2C2 | 1.816849 | 0.00551 |
| PLAU | 1.816949 | 0.000162 |
| TAF4 | 1.817076 | 1.75E-15 |
| FBXO22 | 1.817191 | 2.54E-12 |
| ACTL6A | 1.81727 | 1.03E-13 |
| SAMD12 | 1.817566 | 0.000733 |
| PSMD10 | 1.819426 | 3.98E-15 |
| FMNL2 | 1.819813 | 1.80E-06 |
| H2BC4 | 1.819864 | 0.004862 |
| PARD6B | 1.821865 | 0.000449 |
| TOMM22 | 1.822103 | 4.30E-10 |
| FOXD1 | 1.823219 | 0.018283 |
| FPR1 | 1.825231 | 0.000233 |
| UNK | 1.826436 | 1.23E-07 |
| MOSPD1 | 1.827169 | 1.54E-07 |
| ENOPH1 | 1.827771 | 1.05E-16 |
| FARP2 | 1.828551 | 2.56E-15 |
| SEC61A2 | 1.828574 | 4.37E-13 |
| PIK3R3 | 1.829005 | 2.04E-05 |
| ZNF706 | 1.829614 | 1.07E-13 |
| CT83 | 1.831149 | 0.046785 |
| CD58 | 1.83121 | 9.14E-11 |
| DEPDC1 | 1.831912 | 0.003432 |
| DNAJC9 | 1.83235 | 4.43E-12 |
| RTP4 | 1.833431 | 0.023729 |
| LPXN | 1.833769 | 0.000316 |
| ZNF217 | 1.835067 | 2.46E-12 |
| TFRC | 1.835558 | 4.90E-08 |
| DAP | 1.838509 | 6.50E-11 |
| P4HA2 | 1.839007 | 3.30E-07 |
| GALE | 1.840373 | 1.38E-06 |
| IRX5 | 1.840979 | 0.000128 |
| UCHL1 | 1.841681 | 0.038613 |
| GNL3L | 1.842714 | 3.30E-07 |
| MAPK9 | 1.843221 | 1.62E-10 |
| SOX4 | 1.845125 | 1.75E-09 |
| LOC100130449 | 1.845736 | 0.027622 |
| CIART | 1.849374 | 0.001177 |
| PITRM1 | 1.851046 | 1.25E-10 |
| PAQR4 | 1.851351 | 3.76E-07 |
| H2AX | 1.851562 | 2.15E-08 |
| TSTA3 | 1.852158 | 4.93E-09 |
| EEF1E1 | 1.855364 | 1.01E-09 |
| CA5BP1 | 1.855546 | 9.98E-10 |
| RAB2A | 1.856821 | 1.47E-15 |
| STK38L | 1.857366 | 4.73E-07 |
| OPN3 | 1.859062 | 6.50E-05 |
| RUSC1-AS1 | 1.859173 | 1.06E-06 |
| PRPF18 | 1.859899 | 1.73E-14 |
| FBXO45 | 1.860386 | 5.28E-11 |
| HLA-DQB1 | 1.864535 | 0.013227 |
| PUS7 | 1.867264 | 6.91E-09 |
| TGDS | 1.867336 | 3.82E-11 |
| ABCB10 | 1.867456 | 1.98E-11 |
| ACP1 | 1.867647 | 2.11E-16 |
| RBBP4 | 1.868189 | 6.30E-16 |
| MUC5B | 1.868428 | 0.029033 |
| ATP6V0B | 1.86854 | 1.02E-15 |
| CHMP1B | 1.869432 | 6.21E-12 |
| TFDP1 | 1.86993 | 1.93E-09 |
| H1-2 | 1.870927 | 0.001515 |
| RAB11FIP1 | 1.871188 | 0.000131 |
| PRIM2 | 1.874723 | 0.000114 |
| THEMIS2 | 1.882594 | 0.000139 |
| CD274 | 1.883311 | 0.008764 |
| DPP3 | 1.884655 | 2.51E-12 |
| PIP5K1A | 1.884775 | 1.02E-14 |
| SMIM13 | 1.890052 | 4.55E-09 |
| CBS | 1.890461 | 0.01804 |
| HNRNPAB | 1.891197 | 8.85E-20 |
| CERS2 | 1.893337 | 1.98E-12 |
| DEPDC1B | 1.896215 | 2.20E-05 |
| RFC4 | 1.896372 | 6.00E-08 |
| GFPT2 | 1.89651 | 0.001182 |
| FLAD1 | 1.897075 | 2.66E-10 |
| RMDN1 | 1.899329 | 1.04E-11 |
| RMC1 | 1.900834 | 5.29E-15 |
| CASZ1 | 1.902061 | 0.000191 |
| C5orf46 | 1.902532 | 0.011426 |
| NCAPH | 1.904452 | 0.000249 |
| PBX3 | 1.905473 | 1.50E-06 |
| TGM2 | 1.908039 | 2.05E-05 |
| CMPK2 | 1.90817 | 0.0019 |
| TMEM45A | 1.909935 | 0.00174 |
| PLA2G7 | 1.910285 | 0.001827 |
| C12orf75 | 1.911406 | 4.18E-05 |
| MTX2 | 1.911875 | 2.16E-16 |
| CCNE1 | 1.912883 | 0.001801 |
| P4HA1 | 1.917034 | 3.28E-07 |
| F12 | 1.920874 | 0.000142 |
| CDK2 | 1.92296 | 7.56E-11 |
| VPS37A | 1.92397 | 2.05E-09 |
| CREBZF | 1.926314 | 5.59E-14 |
| PLAGL2 | 1.928047 | 9.70E-11 |
| TM9SF1 | 1.928081 | 7.32E-14 |
| MASTL | 1.929635 | 4.66E-06 |
| DARS2 | 1.934055 | 1.58E-05 |
| HUS1 | 1.938754 | 2.32E-09 |
| MRPL15 | 1.940523 | 7.61E-10 |
| PPAT | 1.941328 | 0.000164 |
| ACOT7 | 1.942354 | 3.91E-06 |
| RAD51 | 1.943506 | 3.45E-05 |
| RSBN1 | 1.944609 | 8.40E-13 |
| SAMD1 | 1.945822 | 2.29E-13 |
| ADSS2 | 1.946063 | 4.45E-18 |
| TPD52 | 1.947851 | 2.81E-08 |
| MMP13 | 1.95472 | 0.009639 |
| ABCC4 | 1.95519 | 0.000189 |
| FIGNL1 | 1.956954 | 1.45E-08 |
| TXNDC9 | 1.956986 | 3.47E-19 |
| SMAD4 | 1.957433 | 1.00E-10 |
| IMPA2 | 1.958066 | 0.004219 |
| MIS18A | 1.959087 | 6.33E-09 |
| SEC23IP | 1.959609 | 2.82E-17 |
| NFE2L3 | 1.961495 | 0.00079 |
| MANEAL | 1.961532 | 0.001728 |
| ZNF586 | 1.965028 | 2.76E-10 |
| ISG20L2 | 1.966831 | 7.43E-18 |
| S100A14 | 1.967923 | 0.008687 |
| TDRKH | 1.97155 | 5.77E-05 |
| BAZ1A | 1.975367 | 4.06E-18 |
| ZNF165 | 1.977204 | 0.000141 |
| FAXC | 1.977547 | 0.000756 |
| HLTF | 1.978174 | 8.96E-14 |
| PGM2L1 | 1.978832 | 4.88E-05 |
| ISG15 | 1.980123 | 0.000439 |
| RB1CC1 | 1.983114 | 3.80E-14 |
| C12orf73 | 1.984846 | 1.10E-10 |
| CAPN15 | 1.986345 | 1.67E-10 |
| PLXNA3 | 1.987671 | 1.54E-08 |
| PCCB | 1.989091 | 1.52E-11 |
| PTPN12 | 1.990608 | 5.72E-19 |
| CXCL17 | 1.995865 | 0.030754 |
| RMND1 | 1.996066 | 1.60E-05 |
| ANAPC10 | 1.9971 | 3.55E-13 |
| METTL8 | 1.999188 | 9.41E-14 |
| TIMM50 | 2.000042 | 1.05E-11 |
| COL8A1 | 2.000062 | 0.01954 |
| MAL2 | 2.00237 | 0.000225 |
| IGF2R | 2.002923 | 8.15E-14 |
| MRPL3 | 2.003858 | 1.47E-20 |
| APPL2 | 2.005079 | 2.47E-06 |
| SORD | 2.005512 | 6.77E-06 |
| PLK1 | 2.01076 | 0.001109 |
| GREM1 | 2.018152 | 0.021846 |
| TLR1 | 2.01844 | 0.000729 |
| TINCR | 2.018458 | 0.005736 |
| EN1 | 2.02008 | 0.025997 |
| DDIT4 | 2.022601 | 1.07E-05 |
| PGM3 | 2.023034 | 2.36E-12 |
| ACTR2 | 2.025026 | 7.19E-28 |
| IL1RN | 2.027227 | 0.001273 |
| C4orf46 | 2.027748 | 7.04E-09 |
| MDK | 2.028149 | 5.29E-06 |
| KCNK1 | 2.029314 | 0.002204 |
| OSBPL3 | 2.031056 | 2.01E-05 |
| SLC20A2 | 2.031327 | 3.63E-09 |
| CXorf40B | 2.03162 | 5.60E-11 |
| TPRKB | 2.036688 | 2.21E-16 |
| MEX3D | 2.039715 | 4.05E-06 |
| GZMB | 2.040159 | 0.012637 |
| NCK1-DT | 2.041217 | 4.64E-07 |
| DCAF13 | 2.042497 | 7.91E-12 |
| AK2 | 2.042831 | 2.30E-13 |
| SMAD7 | 2.044649 | 1.98E-09 |
| MCM8 | 2.045623 | 3.10E-10 |
| SCP2 | 2.046567 | 1.34E-17 |
| ANXA4 | 2.050156 | 5.58E-18 |
| CEP70 | 2.051177 | 7.84E-07 |
| GOLM1 | 2.05377 | 2.98E-07 |
| IL4I1 | 2.055805 | 0.000141 |
| CA2 | 2.056099 | 0.009871 |
| SPDL1 | 2.057709 | 2.00E-06 |
| UBXN6 | 2.06104 | 1.50E-08 |
| LSM2 | 2.061055 | 2.72E-12 |
| IDH2 | 2.063775 | 5.86E-09 |
| CDK7 | 2.064799 | 8.52E-13 |
| PASK | 2.065856 | 1.62E-10 |
| UBR7 | 2.066027 | 1.08E-11 |
| ILF2 | 2.071381 | 3.62E-12 |
| EXPH5 | 2.072212 | 2.22E-05 |
| SPC25 | 2.07676 | 0.000212 |
| RET | 2.077049 | 0.031255 |
| PPP4C | 2.077419 | 4.33E-27 |
| TGIF1 | 2.07801 | 1.24E-18 |
| AP1AR | 2.080586 | 2.40E-12 |
| PRPS2 | 2.080728 | 1.73E-08 |
| PEX13 | 2.080933 | 4.68E-20 |
| PNPT1 | 2.082336 | 2.26E-14 |
| KNL1 | 2.082495 | 1.07E-05 |
| PSMC3 | 2.085225 | 1.98E-18 |
| PSMD11 | 2.08795 | 1.62E-11 |
| REEP1 | 2.088221 | 0.011704 |
| CSRNP2 | 2.090892 | 1.64E-12 |
| IQCG | 2.094226 | 0.006609 |
| SDAD1 | 2.096275 | 1.95E-15 |
| PROSER1 | 2.096714 | 2.25E-12 |
| NUP155 | 2.097304 | 1.38E-08 |
| GAL | 2.097581 | 0.009169 |
| XRN2 | 2.098512 | 1.68E-20 |
| VPS72 | 2.098703 | 3.73E-12 |
| VRK2 | 2.100008 | 8.49E-13 |
| CAND1 | 2.101634 | 1.76E-16 |
| SMS | 2.103411 | 2.69E-15 |
| ELF4 | 2.104842 | 2.76E-09 |
| CST6 | 2.105199 | 0.00409 |
| MYBL2 | 2.105266 | 1.99E-05 |
| BCL2L12 | 2.105536 | 7.87E-11 |
| ASPSCR1 | 2.107852 | 1.03E-11 |
| CDH11 | 2.111484 | 0.006137 |
| ERI1 | 2.111541 | 2.72E-08 |
| MARCHF5 | 2.112029 | 7.02E-15 |
| IDH3A | 2.114244 | 2.36E-11 |
| ZCCHC2 | 2.116698 | 1.56E-05 |
| SEPTIN6 | 2.118788 | 1.05E-08 |
| TSEN15 | 2.126254 | 2.45E-14 |
| SLC7A11 | 2.132476 | 0.000476 |
| USP10 | 2.13591 | 9.93E-14 |
| CLIC3 | 2.136634 | 0.005494 |
| POLE2 | 2.137679 | 2.69E-05 |
| MIEN1 | 2.139838 | 0.002393 |
| MCMBP | 2.139881 | 1.01E-18 |
| PKIB | 2.142016 | 0.005964 |
| THBS1 | 2.142233 | 3.53E-05 |
| DIAPH3 | 2.147091 | 6.93E-05 |
| MRPL38 | 2.149719 | 7.63E-13 |
| STAM | 2.151028 | 4.09E-18 |
| CENPN | 2.155803 | 0.000346 |
| HSD17B6 | 2.160593 | 1.32E-07 |
| CDC42 | 2.16381 | 1.62E-22 |
| PSMG1 | 2.166699 | 1.16E-07 |
| PREP | 2.175243 | 5.31E-15 |
| MYBL1 | 2.175249 | 0.012241 |
| STX6 | 2.175546 | 7.80E-18 |
| CDCA8 | 2.178278 | 0.000252 |
| EXO1 | 2.180379 | 5.91E-05 |
| GDF15 | 2.18066 | 0.008726 |
| KPNA2 | 2.181151 | 2.65E-12 |
| ZNF367 | 2.1829 | 4.18E-07 |
| PRAME | 2.183252 | 0.023494 |
| IDO1 | 2.184318 | 0.02966 |
| UGCG | 2.184998 | 3.39E-09 |
| MND1 | 2.187095 | 0.000168 |
| SLAMF8 | 2.189901 | 0.001494 |
| SNX2 | 2.190749 | 1.70E-14 |
| CENPM | 2.191625 | 8.68E-07 |
| DESI2 | 2.194198 | 2.38E-15 |
| PAXIP1 | 2.19573 | 7.47E-17 |
| SAMD9L | 2.199146 | 5.33E-05 |
| HOPX | 2.203284 | 0.000916 |
| MFSD14C | 2.204896 | 3.21E-10 |
| LAMP3 | 2.206594 | 0.00974 |
| RBM12B | 2.20703 | 9.54E-16 |
| NEK6 | 2.20814 | 2.28E-13 |
| ATP6V0A2 | 2.211649 | 3.71E-10 |
| SMYD3 | 2.212049 | 2.22E-08 |
| CD9 | 2.213602 | 8.69E-13 |
| METTL21A | 2.215792 | 3.79E-09 |
| LSM10 | 2.217247 | 3.28E-16 |
| MED8 | 2.217883 | 6.60E-16 |
| DNA2 | 2.218812 | 6.33E-08 |
| PKMYT1 | 2.220751 | 6.50E-09 |
| SMAP1 | 2.221046 | 2.70E-19 |
| BORA | 2.22216 | 1.81E-08 |
| ASCC3 | 2.222363 | 1.29E-13 |
| RSAD2 | 2.222638 | 0.00128 |
| AP1M2 | 2.222926 | 3.51E-05 |
| ARMC10 | 2.225505 | 1.82E-18 |
| HMGB3 | 2.233482 | 4.42E-07 |
| ME2 | 2.23676 | 9.34E-11 |
| SLBP | 2.236926 | 3.11E-16 |
| TTYH3 | 2.244091 | 3.57E-13 |
| FBXO7 | 2.246918 | 8.57E-19 |
| UNC93B1 | 2.247135 | 5.80E-09 |
| GSTCD | 2.249537 | 5.88E-11 |
| NUP153 | 2.254994 | 2.70E-17 |
| CEP89 | 2.256437 | 3.98E-14 |
| CHML | 2.260516 | 5.74E-07 |
| UBE2W | 2.261189 | 6.67E-16 |
| MICB | 2.261748 | 2.11E-05 |
| SAMSN1 | 2.264856 | 0.000129 |
| FKBP14 | 2.264908 | 4.01E-13 |
| PLEKHF2 | 2.270447 | 4.24E-08 |
| ADAMDEC1 | 2.27104 | 0.014301 |
| SLC39A4 | 2.272895 | 1.53E-05 |
| ECE2 | 2.274398 | 3.09E-05 |
| MIS18BP1 | 2.27991 | 5.07E-14 |
| PRR15 | 2.282731 | 0.006641 |
| BRI3BP | 2.284202 | 3.95E-16 |
| ELAVL1 | 2.284207 | 1.04E-32 |
| COL12A1 | 2.28625 | 0.000255 |
| CPSF2 | 2.291715 | 1.38E-14 |
| AP3M1 | 2.293754 | 1.84E-15 |
| KNSTRN | 2.295687 | 1.71E-07 |
| RNF14 | 2.295704 | 2.41E-13 |
| TIPIN | 2.297969 | 1.30E-05 |
| FAM210A | 2.30014 | 1.09E-11 |
| MTFR2 | 2.30167 | 0.00078 |
| C1orf131 | 2.30182 | 2.29E-20 |
| MAPKAPK5 | 2.303162 | 8.15E-22 |
| ADAM19 | 2.304707 | 1.02E-07 |
| CCN2 | 2.306427 | 9.53E-05 |
| PEX3 | 2.306742 | 1.47E-11 |
| PPP4R2 | 2.308236 | 4.27E-23 |
| ANKH | 2.309713 | 1.61E-09 |
| CNIH4 | 2.310393 | 3.08E-18 |
| UBR5 | 2.310426 | 3.45E-16 |
| PTPN3 | 2.311586 | 1.40E-12 |
| GLRX3 | 2.314027 | 5.47E-14 |
| FASTKD5 | 2.315346 | 8.96E-16 |
| ASF1B | 2.3171 | 2.47E-08 |
| TMEM216 | 2.319365 | 2.26E-14 |
| RGS4 | 2.32293 | 0.001521 |
| RARRES1 | 2.323105 | 0.02489 |
| ZMYM6 | 2.326108 | 9.98E-13 |
| TMEM167A | 2.328692 | 1.48E-23 |
| SERPINH1 | 2.330903 | 2.04E-12 |
| C1orf112 | 2.333232 | 2.86E-07 |
| BAIAP2L1 | 2.333325 | 8.70E-09 |
| LOC374443 | 2.3338 | 3.49E-08 |
| TOR1A | 2.336645 | 8.96E-24 |
| MED17 | 2.338427 | 2.90E-19 |
| MCM2 | 2.341648 | 8.24E-08 |
| ATP2A2 | 2.341986 | 2.74E-25 |
| KDELR2 | 2.343492 | 2.69E-19 |
| CEMIP | 2.345265 | 0.000626 |
| NCF2 | 2.345328 | 7.41E-06 |
| DUS4L | 2.346205 | 2.20E-12 |
| DCBLD1 | 2.347374 | 9.97E-09 |
| NUTF2 | 2.347416 | 5.38E-16 |
| C1orf56 | 2.350598 | 6.63E-11 |
| CXCL8 | 2.355331 | 0.006864 |
| INAVA | 2.358242 | 0.002819 |
| C4orf33 | 2.359389 | 1.38E-07 |
| HORMAD1 | 2.359629 | 0.03697 |
| LAGE3 | 2.360164 | 3.14E-11 |
| PRCC | 2.360166 | 8.20E-22 |
| STARD4 | 2.361271 | 2.24E-07 |
| LCP1 | 2.361845 | 6.89E-05 |
| PRIM1 | 2.362451 | 1.08E-06 |
| LEO1 | 2.363544 | 1.77E-11 |
| NECTIN4 | 2.365825 | 4.48E-06 |
| CLDN7 | 2.370688 | 4.07E-06 |
| LURAP1L | 2.37731 | 0.000398 |
| PRRC1 | 2.379925 | 4.02E-20 |
| FRK | 2.381215 | 0.00012 |
| NCAPG | 2.382837 | 3.88E-06 |
| TROAP | 2.383945 | 4.13E-10 |
| CST1 | 2.389304 | 0.00722 |
| RFC3 | 2.398839 | 3.16E-08 |
| FBXL6 | 2.401037 | 7.57E-08 |
| CD86 | 2.402108 | 3.13E-05 |
| PRPF38A | 2.407883 | 1.75E-13 |
| GGH | 2.411154 | 2.01E-05 |
| MED6 | 2.415447 | 1.11E-16 |
| BLM | 2.416771 | 1.60E-05 |
| CORO2A | 2.417287 | 1.37E-07 |
| FN1 | 2.418253 | 2.95E-05 |
| PRDM1 | 2.418361 | 2.99E-06 |
| OSMR | 2.418363 | 2.41E-05 |
| SAYSD1 | 2.421587 | 1.03E-12 |
| DCUN1D1 | 2.424177 | 1.54E-20 |
| SS18 | 2.426725 | 3.35E-25 |
| DDX39A | 2.427255 | 4.85E-17 |
| CEP152 | 2.427396 | 6.15E-08 |
| FOS | 2.427816 | 0.00221 |
| KIFBP | 2.42829 | 2.54E-16 |
| HACD3 | 2.431613 | 2.20E-16 |
| PPP1CB | 2.431995 | 1.06E-13 |
| FGFR1OP2 | 2.435715 | 9.81E-16 |
| SAP30 | 2.437579 | 2.27E-10 |
| H2AZ2 | 2.43782 | 9.47E-23 |
| TDO2 | 2.438674 | 0.001031 |
| NEU1 | 2.440262 | 8.39E-15 |
| NEMP1 | 2.442162 | 4.07E-17 |
| KIF18B | 2.443888 | 2.01E-06 |
| KRIT1 | 2.445667 | 1.20E-17 |
| GAS2L3 | 2.447061 | 3.26E-09 |
| MPV17 | 2.448015 | 9.95E-18 |
| GPSM2 | 2.44807 | 1.21E-09 |
| E2F8 | 2.450583 | 1.96E-05 |
| MMP12 | 2.454378 | 0.015429 |
| ITCH | 2.455632 | 8.89E-15 |
| SCO1 | 2.459307 | 5.26E-15 |
| TAF12 | 2.461716 | 2.70E-22 |
| RDH10 | 2.465539 | 0.001448 |
| WRAP73 | 2.469168 | 5.59E-18 |
| FAR2 | 2.475877 | 0.000707 |
| CNOT3 | 2.476612 | 7.02E-19 |
| POLR2K | 2.479661 | 2.44E-21 |
| CUL2 | 2.481788 | 1.48E-23 |
| C15orf48 | 2.48301 | 0.000251 |
| RMI2 | 2.484839 | 5.83E-07 |
| PGP | 2.485679 | 1.94E-15 |
| SDR16C5 | 2.48995 | 0.012325 |
| DDIAS | 2.490448 | 1.93E-05 |
| MIA3 | 2.492018 | 3.05E-16 |
| PCNA | 2.494345 | 8.56E-16 |
| RPS6KC1 | 2.497573 | 5.72E-19 |
| HMGCR | 2.498538 | 2.06E-14 |
| MCM4 | 2.499818 | 5.74E-12 |
| COPZ1 | 2.50098 | 8.74E-22 |
| TIMELESS | 2.506276 | 1.30E-13 |
| MRPL13 | 2.510241 | 3.63E-17 |
| GLUD2 | 2.510806 | 1.47E-10 |
| PEX2 | 2.5129 | 2.27E-18 |
| MTHFD2 | 2.516803 | 4.44E-17 |
| SMC6 | 2.519996 | 4.56E-21 |
| PDSS1 | 2.520475 | 2.74E-08 |
| AGAP1 | 2.52212 | 5.11E-12 |
| CTDSPL2 | 2.522461 | 1.99E-14 |
| PITX1 | 2.52491 | 1.74E-05 |
| BRIP1 | 2.526254 | 7.61E-05 |
| GON7 | 2.529009 | 1.43E-16 |
| P2RX4 | 2.529616 | 7.73E-13 |
| SGO2 | 2.535319 | 7.67E-08 |
| ARFRP1 | 2.54101 | 1.85E-21 |
| ARHGAP32 | 2.541601 | 9.52E-06 |
| TCF19 | 2.542909 | 6.92E-07 |
| PLEKHB2 | 2.546374 | 4.20E-24 |
| DLAT | 2.546598 | 6.56E-16 |
| PMS2P2 | 2.556248 | 5.92E-20 |
| H2BC12 | 2.557182 | 4.49E-10 |
| THUMPD3-AS1 | 2.558653 | 2.40E-07 |
| NAPA | 2.559157 | 3.11E-21 |
| RNF115 | 2.559275 | 4.79E-21 |
| DYRK2 | 2.564676 | 2.38E-15 |
| GFPT1 | 2.572163 | 2.51E-18 |
| SLC39A7 | 2.573334 | 2.39E-16 |
| ZUP1 | 2.574128 | 6.25E-14 |
| DBF4 | 2.575325 | 4.22E-10 |
| TBCC | 2.57886 | 5.36E-25 |
| ARL1 | 2.58013 | 1.68E-23 |
| TMEM70 | 2.580148 | 1.60E-21 |
| COMMD10 | 2.581543 | 4.82E-12 |
| NEK2 | 2.582121 | 1.55E-05 |
| SDC1 | 2.583066 | 3.08E-11 |
| LACTB2 | 2.584408 | 4.24E-11 |
| ABCF2 | 2.584939 | 6.64E-19 |
| BSG | 2.585607 | 1.12E-15 |
| SSX2IP | 2.589583 | 6.61E-11 |
| OSBPL11 | 2.589988 | 9.33E-23 |
| DHCR7 | 2.591156 | 1.66E-07 |
| PRMT3 | 2.594376 | 1.11E-14 |
| TMCO3 | 2.596945 | 9.84E-15 |
| ZNF814 | 2.598178 | 4.06E-09 |
| LMNB1 | 2.600279 | 8.11E-09 |
| AP3B1 | 2.600371 | 5.70E-25 |
| RNASEH2A | 2.602801 | 5.21E-10 |
| HMOX1 | 2.603001 | 7.09E-08 |
| TUBGCP4 | 2.606079 | 5.20E-16 |
| ZDHHC9 | 2.606793 | 2.02E-20 |
| PPP5C | 2.606885 | 4.82E-25 |
| NSUN4 | 2.610711 | 8.55E-18 |
| ZWILCH | 2.611112 | 9.55E-21 |
| FBXO5 | 2.612849 | 1.70E-10 |
| ICAM1 | 2.612919 | 1.18E-06 |
| MSH2 | 2.613278 | 1.61E-12 |
| PMAIP1 | 2.623484 | 5.29E-05 |
| ANKRD16 | 2.624397 | 4.76E-10 |
| ZNF124 | 2.625927 | 3.75E-11 |
| TBC1D7 | 2.626765 | 3.88E-14 |
| ORMDL2 | 2.63152 | 6.66E-16 |
| PSMD12 | 2.631766 | 8.67E-16 |
| SLC2A10 | 2.63467 | 0.000344 |
| PRKCI | 2.63768 | 6.08E-23 |
| OIP5 | 2.639398 | 3.09E-05 |
| SCYL2 | 2.63997 | 8.99E-25 |
| FARSA | 2.640565 | 4.16E-17 |
| TMEM185B | 2.641312 | 3.98E-15 |
| CXorf40A | 2.641788 | 1.88E-14 |
| TGFB1 | 2.642397 | 2.40E-12 |
| DIO2 | 2.645705 | 0.0012 |
| UTP25 | 2.646084 | 3.06E-15 |
| STX3 | 2.646623 | 1.32E-17 |
| DOP1B | 2.648178 | 4.72E-10 |
| PRMT6 | 2.648742 | 1.41E-12 |
| KLHDC7B | 2.654935 | 0.013315 |
| EPSTI1 | 2.658927 | 0.000105 |
| FAM20B | 2.664058 | 4.80E-24 |
| CDK5 | 2.668454 | 1.92E-14 |
| FBXO21 | 2.67485 | 2.45E-26 |
| CHEK1 | 2.67523 | 8.63E-06 |
| FOXA1 | 2.676613 | 0.032296 |
| OAZ2 | 2.678268 | 1.36E-23 |
| IGHD | 2.681857 | 0.003135 |
| LLPH | 2.685349 | 5.35E-18 |
| FGFR3 | 2.688079 | 0.000319 |
| H2BC5 | 2.689896 | 5.49E-10 |
| NCEH1 | 2.693796 | 6.90E-08 |
| NRBF2 | 2.69607 | 2.52E-18 |
| CREM | 2.696947 | 1.46E-18 |
| PNO1 | 2.706975 | 3.91E-20 |
| PIMREG | 2.707438 | 2.43E-05 |
| PTP4A3 | 2.712891 | 2.97E-09 |
| IFI6 | 2.714246 | 3.62E-06 |
| ABI1 | 2.714751 | 2.87E-24 |
| SLC16A1 | 2.718761 | 0.000306 |
| CCL4 | 2.726335 | 7.01E-05 |
| PLPP2 | 2.731864 | 2.46E-11 |
| CCL2 | 2.737234 | 1.85E-05 |
| IQGAP3 | 2.738585 | 8.01E-08 |
| TBC1D31 | 2.755917 | 1.82E-09 |
| RACGAP1 | 2.760763 | 4.01E-12 |
| SGPL1 | 2.763038 | 1.05E-26 |
| HYLS1 | 2.764594 | 5.95E-11 |
| INTS7 | 2.76558 | 1.79E-20 |
| HOTAIR | 2.766396 | 0.000295 |
| FAM118B | 2.771537 | 5.11E-16 |
| NAT1 | 2.775273 | 0.004862 |
| HPRT1 | 2.775906 | 8.12E-15 |
| GRB7 | 2.777146 | 0.002968 |
| UCHL5 | 2.780363 | 3.58E-14 |
| LDLR | 2.800053 | 1.29E-08 |
| PHLDA2 | 2.805228 | 8.29E-08 |
| ODF2 | 2.816967 | 3.92E-16 |
| HNRNPC | 2.829686 | 2.92E-30 |
| GINS2 | 2.838 | 3.44E-08 |
| PRELID3B | 2.838894 | 4.16E-21 |
| GXYLT2 | 2.842133 | 1.20E-05 |
| OAS3 | 2.844824 | 1.02E-08 |
| SELENOT | 2.848408 | 3.62E-25 |
| TIAM1 | 2.85002 | 2.43E-09 |
| PRKCD | 2.853489 | 6.00E-14 |
| OSTC | 2.856118 | 1.82E-25 |
| ABRACL | 2.860847 | 4.14E-15 |
| RBM6 | 2.864103 | 1.19E-05 |
| CENPK | 2.866079 | 3.21E-10 |
| RGS1 | 2.867965 | 0.000545 |
| ST8SIA6-AS1 | 2.870344 | 0.003041 |
| KCTD15 | 2.872017 | 3.48E-08 |
| KIF15 | 2.87255 | 3.51E-06 |
| ZNF627 | 2.87443 | 1.26E-22 |
| MMP3 | 2.876358 | 0.00121 |
| MPDU1 | 2.878783 | 7.78E-22 |
| GET3 | 2.882569 | 4.99E-21 |
| TP53RK | 2.883151 | 2.02E-17 |
| DHTKD1 | 2.883184 | 2.37E-11 |
| SPATC1L | 2.887384 | 2.02E-08 |
| HNRNPF | 2.88778 | 2.14E-27 |
| GINS1 | 2.887975 | 1.64E-08 |
| SLC35B1 | 2.891528 | 2.39E-13 |
| KNTC1 | 2.892953 | 5.30E-17 |
| RMI1 | 2.898601 | 1.54E-20 |
| DESI1 | 2.904596 | 1.03E-10 |
| KYNU | 2.912841 | 0.000601 |
| SLC25A43 | 2.914039 | 3.72E-15 |
| MAPK13 | 2.914339 | 2.04E-10 |
| HLA-DQA1 | 2.91746 | 0.013082 |
| COA1 | 2.924028 | 1.21E-20 |
| FBXO6 | 2.924502 | 1.53E-08 |
| APH1A | 2.925839 | 7.72E-23 |
| SHCBP1 | 2.926365 | 7.77E-08 |
| CDC7 | 2.939595 | 2.89E-08 |
| PARP9 | 2.948102 | 2.02E-13 |
| CDCA3 | 2.948867 | 1.82E-06 |
| RIDA | 2.95548 | 2.14E-12 |
| USP21 | 2.956207 | 7.51E-23 |
| SPTSSA | 2.968568 | 8.10E-14 |
| MRPS12 | 2.971084 | 6.45E-19 |
| AREL1 | 2.971171 | 8.80E-26 |
| PGD | 2.987827 | 1.66E-14 |
| ENC1 | 2.988227 | 3.23E-12 |
| NUDCD1 | 2.997318 | 8.68E-17 |
| ABHD17C | 2.997987 | 1.56E-10 |
| LMBR1 | 2.999657 | 7.42E-13 |
| TLCD1 | 3.018267 | 6.90E-07 |
| LRIF1 | 3.018599 | 6.02E-23 |
| CEACAM6 | 3.03535 | 0.019412 |
| BCL2A1 | 3.035515 | 0.000214 |
| PIK3CA | 3.036857 | 5.39E-19 |
| PSRC1 | 3.038213 | 3.83E-08 |
| RABIF | 3.038694 | 1.33E-22 |
| UBE2G1 | 3.041937 | 1.78E-28 |
| CDC45 | 3.04358 | 8.14E-08 |
| OAS2 | 3.043774 | 3.07E-05 |
| PLPP4 | 3.048189 | 2.49E-05 |
| ZBED4 | 3.048389 | 4.54E-14 |
| NCBP1 | 3.067821 | 2.39E-20 |
| LRRC59 | 3.073853 | 9.99E-17 |
| MRPL44 | 3.074957 | 3.53E-23 |
| SMC4 | 3.07822 | 1.10E-20 |
| TPM4 | 3.081261 | 5.36E-21 |
| POLE3 | 3.099211 | 6.14E-24 |
| CHAC2 | 3.10401 | 8.29E-08 |
| SIAH2 | 3.109649 | 3.77E-16 |
| CDC6 | 3.115325 | 2.43E-05 |
| H2BC6 | 3.117856 | 1.69E-12 |
| RAD51AP1 | 3.121984 | 4.61E-09 |
| ASF1A | 3.123908 | 8.68E-14 |
| CDCA7 | 3.125439 | 0.000802 |
| EPPK1 | 3.127254 | 1.85E-06 |
| KMO | 3.129158 | 0.000405 |
| DVL3 | 3.130241 | 2.30E-26 |
| KIF26B | 3.130955 | 1.47E-07 |
| CYB5R4 | 3.131953 | 5.45E-19 |
| TACC3 | 3.134347 | 4.82E-12 |
| CASP8AP2 | 3.138257 | 6.17E-21 |
| ZNF207 | 3.141315 | 2.32E-30 |
| DNAJC3 | 3.143743 | 6.78E-21 |
| SECISBP2 | 3.14391 | 3.38E-23 |
| S100A9 | 3.150555 | 0.007648 |
| BAG2 | 3.152288 | 7.17E-06 |
| RAD54B | 3.154217 | 1.78E-12 |
| SLC25A39 | 3.165198 | 4.54E-24 |
| NDUFAF6 | 3.165644 | 1.05E-19 |
| SLC44A4 | 3.166678 | 0.001112 |
| AKIRIN1 | 3.167025 | 1.00E-25 |
| LRRC15 | 3.187873 | 0.000285 |
| MMP9 | 3.201083 | 0.000135 |
| CENPW | 3.205906 | 2.17E-06 |
| ERO1A | 3.209592 | 6.34E-11 |
| SLC25A32 | 3.210668 | 3.50E-19 |
| COG5 | 3.212057 | 5.17E-27 |
| SULF1 | 3.212973 | 6.32E-05 |
| VAMP7 | 3.214217 | 1.38E-21 |
| FAP | 3.216916 | 8.11E-07 |
| ABHD12 | 3.236461 | 9.61E-28 |
| SUV39H2 | 3.252007 | 3.26E-10 |
| SELENOI | 3.263489 | 2.71E-16 |
| CKAP5 | 3.272626 | 5.86E-30 |
| DONSON | 3.279717 | 9.82E-16 |
| HJURP | 3.299487 | 1.39E-07 |
| NCAPD2 | 3.30246 | 1.89E-10 |
| DUSP5 | 3.311804 | 1.83E-07 |
| CDCA5 | 3.312454 | 2.09E-10 |
| CKAP2 | 3.315127 | 1.20E-15 |
| JPT1 | 3.328973 | 2.10E-16 |
| KDELR3 | 3.336986 | 7.21E-15 |
| PPP1R2 | 3.34756 | 5.93E-29 |
| ALOX5AP | 3.349775 | 1.06E-07 |
| KIF2A | 3.359396 | 3.78E-27 |
| CLDND1 | 3.360591 | 9.62E-20 |
| FOXM1 | 3.363247 | 7.38E-06 |
| AKT1S1 | 3.369799 | 1.85E-31 |
| E2F5 | 3.371563 | 5.27E-12 |
| SOX11 | 3.378448 | 0.000775 |
| GCH1 | 3.385067 | 7.20E-14 |
| DCAF10 | 3.38584 | 2.10E-20 |
| PTTG1 | 3.400184 | 2.83E-10 |
| LRR1 | 3.401349 | 3.28E-13 |
| ATP6V1C1 | 3.401694 | 7.12E-24 |
| AURKB | 3.402746 | 1.65E-08 |
| INHBA | 3.416803 | 1.83E-08 |
| SRD5A3 | 3.419398 | 1.04E-11 |
| FAM76A | 3.419773 | 1.40E-24 |
| CENPE | 3.422086 | 2.95E-07 |
| ATAD2 | 3.440782 | 1.11E-14 |
| ANKRD22 | 3.445015 | 5.01E-08 |
| PDAP1 | 3.475713 | 1.55E-22 |
| ZNF652 | 3.478675 | 2.09E-06 |
| CTHRC1 | 3.494969 | 3.63E-06 |
| RIT1 | 3.495663 | 3.58E-28 |
| KIF14 | 3.496992 | 7.62E-08 |
| H2BC21 | 3.513813 | 1.15E-09 |
| YWHAH | 3.514364 | 2.26E-33 |
| GPR160 | 3.51988 | 4.18E-05 |
| TMEM41B | 3.526825 | 9.10E-27 |
| PTER | 3.537268 | 1.45E-17 |
| POGK | 3.552326 | 3.31E-26 |
| FCGR1B | 3.564958 | 1.65E-08 |
| CXCL11 | 3.569309 | 0.000262 |
| NDC80 | 3.575117 | 2.30E-08 |
| SMC2 | 3.576764 | 3.68E-12 |
| CTSS | 3.604186 | 1.28E-09 |
| CCNE2 | 3.640328 | 1.78E-10 |
| IFI44L | 3.662093 | 9.21E-06 |
| CBX2 | 3.662475 | 2.30E-06 |
| MFAP2 | 3.665702 | 3.44E-11 |
| FPR3 | 3.666679 | 5.22E-08 |
| KCTD5 | 3.69545 | 8.07E-28 |
| SPP1 | 3.696661 | 0.000221 |
| LARP4B | 3.712304 | 3.83E-34 |
| TIGAR | 3.724036 | 1.13E-18 |
| MKI67 | 3.724954 | 6.29E-10 |
| CARMIL1 | 3.738247 | 4.58E-20 |
| H2BC9 | 3.747159 | 4.14E-15 |
| CCNA2 | 3.760579 | 3.00E-11 |
| ERP27 | 3.781728 | 4.28E-05 |
| UBE2H | 3.798928 | 6.90E-29 |
| LOC100190986 | 3.808542 | 2.23E-11 |
| SPAG1 | 3.810091 | 1.91E-17 |
| BUB1 | 3.810555 | 1.66E-08 |
| TYMS | 3.814519 | 7.90E-13 |
| S100A7 | 3.83973 | 0.014444 |
| KIF20A | 3.841066 | 9.74E-10 |
| ASCC1 | 3.847066 | 2.98E-21 |
| FOLR1 | 3.895013 | 6.48E-26 |
| AGO2 | 3.911237 | 7.00E-23 |
| DDA1 | 3.914721 | 3.10E-31 |
| FANCI | 3.929926 | 3.23E-15 |
| POLR3K | 3.945501 | 2.25E-34 |
| FNDC1 | 3.950291 | 5.07E-09 |
| KIF23 | 3.980505 | 7.94E-12 |
| CTPS1 | 3.98632 | 3.77E-15 |
| TRIM59 | 4.028521 | 1.49E-23 |
| DLGAP5 | 4.04951 | 3.12E-08 |
| TNFRSF12A | 4.053689 | 6.32E-23 |
| HMMR | 4.066429 | 3.93E-11 |
| KIF4A | 4.090705 | 5.04E-09 |
| CEP55 | 4.093448 | 4.54E-10 |
| LSM4 | 4.095723 | 5.41E-34 |
| NUF2 | 4.135843 | 2.43E-10 |
| SPAG5 | 4.141797 | 8.60E-11 |
| UBE2M | 4.163831 | 4.97E-28 |
| BUB1B | 4.167253 | 1.94E-13 |
| OAS1 | 4.194211 | 3.19E-11 |
| UHRF1 | 4.195286 | 6.34E-17 |
| CCN4 | 4.21815 | 1.13E-08 |
| SLC7A5 | 4.228348 | 1.79E-08 |
| SAE1 | 4.291327 | 3.50E-33 |
| PACC1 | 4.298128 | 8.54E-22 |
| CDK1 | 4.364376 | 2.56E-17 |
| APOBEC3B | 4.380918 | 1.43E-09 |
| COMP | 4.387695 | 7.99E-06 |
| TK1 | 4.433131 | 4.11E-16 |
| M6PR | 4.442277 | 1.29E-35 |
| KIF11 | 4.475477 | 9.36E-16 |
| SQLE | 4.476874 | 3.97E-24 |
| MRPL42 | 4.486768 | 7.05E-35 |
| PSENEN | 4.490908 | 3.93E-38 |
| AURKA | 4.497595 | 9.24E-13 |
| MMP1 | 4.53536 | 0.00011 |
| DTL | 4.553367 | 1.55E-16 |
| TRIP13 | 4.573266 | 2.84E-12 |
| ECT2 | 4.618085 | 3.81E-26 |
| CKS2 | 4.633577 | 5.88E-22 |
| MELK | 4.658001 | 3.39E-14 |
| PBK | 4.688643 | 1.35E-11 |
| S100A8 | 4.708246 | 0.001895 |
| MAD2L1 | 4.722094 | 1.44E-16 |
| H2BS1 | 4.737291 | 8.48E-21 |
| PLAUR | 4.739326 | 1.26E-25 |
| CENPU | 4.76396 | 1.82E-23 |
| EZH2 | 4.776101 | 8.52E-21 |
| TTK | 4.781931 | 3.34E-09 |
| ANLN | 4.797738 | 5.42E-12 |
| UBE2S | 4.848337 | 7.85E-21 |
| GPRC5A | 4.927653 | 3.89E-10 |
| FAM83D | 4.935068 | 4.30E-10 |
| KIF2C | 4.93595 | 8.59E-12 |
| ZWINT | 4.982845 | 1.07E-24 |
| BIRC5 | 5.025918 | 9.13E-11 |
| CCNB2 | 5.031061 | 3.53E-15 |
| MMP11 | 5.053943 | 1.89E-09 |
| CDKN3 | 5.078218 | 2.22E-13 |
| CXCL9 | 5.093572 | 8.54E-06 |
| VMP1 | 5.217556 | 2.30E-17 |
| GJB2 | 5.240689 | 5.32E-13 |
| CENPF | 5.430944 | 7.80E-19 |
| PNP | 5.448329 | 2.79E-27 |
| CCNB1 | 5.461999 | 3.85E-21 |
| ASPM | 5.469538 | 1.11E-13 |
| TPX2 | 5.530104 | 4.95E-13 |
| CDC20 | 5.626936 | 7.98E-13 |
| RRM2 | 5.65524 | 3.25E-19 |
| ALYREF | 5.679342 | 2.90E-33 |
| UBE2C | 5.748188 | 6.75E-19 |
| UBE2T | 5.757736 | 1.92E-21 |
| NUSAP1 | 5.829473 | 9.15E-24 |
| PRC1 | 5.954548 | 4.33E-19 |
| EIF5A | 5.981033 | 2.68E-16 |
| CXCL10 | 6.426378 | 5.01E-10 |
| PCLAF | 6.507915 | 9.63E-29 |
| S100P | 6.591051 | 2.54E-09 |
| TOP2A | 6.719804 | 2.10E-21 |
| COL10A1 | 6.746242 | 1.78E-11 |
| COL11A1 | 7.010925 | 1.90E-10 |
