# Supplementary material for: Identification of hub genes and small molecule therapeutic drugs related to breast cancer with comprehensive bioinformatics analysis
Source: PeerJ. 2020 Sep 29;8:e9946. doi: 10.7717/peerj.9946 (PMC7556247; doi:10.7717/peerj.9946)
Supplement: Supplemental Information 15 [file peerj-08-9946-s015.docx]

| **Symbol** | \|**Log2FC**\| | **q-value** |
| --- | --- | --- |
| A2M | -1.12 | 1.94E-60 |
| A4GALT | -1.04 | 2.65E-53 |
| AADAC | -1.438 | 7.54E-145 |
| AAGAB | 1.198 | 1.42E-114 |
| AARD | 1.399 | 1.83E-42 |
| AASS | -2.558 | 1.69E-269 |
| ABAT | 1.115 | 1.68E-20 |
| ABC7-42404400C24.1 | -1.028 | 1.93E-88 |
| ABCA1 | -1.242 | 9.78E-90 |
| ABCA10 | -3.431 | 0.00E+00 |
| ABCA3 | 1.078 | 2.98E-52 |
| ABCA5 | -1.863 | 3.11E-195 |
| ABCA6 | -3.227 | 0.00E+00 |
| ABCA8 | -4.025 | 0.00E+00 |
| ABCA9 | -3.732 | 0.00E+00 |
| ABCB1 | -1.691 | 2.63E-148 |
| ABCB5 | -1.264 | 4.72E-210 |
| ABCC11 | 1.823 | 1.09E-20 |
| ABCC9 | -1.668 | 4.25E-152 |
| ABCD2 | -1.228 | 1.70E-174 |
| ABCD4 | -1.105 | 9.84E-146 |
| ABHD17C | 1.57 | 5.98E-122 |
| ABHD2 | 1.044 | 5.61E-32 |
| ABHD6 | -1.147 | 7.84E-106 |
| ABI3BP | -2.112 | 1.79E-97 |
| ABLIM1 | -1.394 | 3.02E-113 |
| ABLIM3 | -2.162 | 2.25E-127 |
| ABO | -1.207 | 1.61E-62 |
| ABRACL | 1.559 | 4.64E-145 |
| ABTB1 | -1.543 | 1.50E-143 |
| AC002116.8 | -1.232 | 1.03E-77 |
| AC002398.12 | -2.78 | 9.31E-174 |
| AC005104.3 | -1.067 | 3.29E-126 |
| AC005152.3 | -3.385 | 1.38E-49 |
| AC005154.6 | -1.176 | 3.45E-110 |
| AC005154.7 | -1.031 | 5.02E-187 |
| AC005255.3 | 1.9 | 3.10E-112 |
| AC005519.4 | -1.48 | 2.45E-227 |
| AC005682.5 | -1.855 | 5.29E-241 |
| AC005786.7 | 1.198 | 9.12E-17 |
| AC005943.2 | 1.722 | 1.01E-19 |
| AC005943.6 | 2.325 | 6.74E-16 |
| AC006128.2 | -1.719 | 1.98E-58 |
| AC007009.1 | 1.059 | 4.17E-51 |
| AC007566.10 | -1.316 | 3.36E-143 |
| AC007743.1 | -1.57 | 3.19E-142 |
| AC009005.2 | 1.364 | 2.65E-77 |
| AC009120.6 | -1.179 | 1.04E-108 |
| AC016735.2 | -1.528 | 2.93E-31 |
| AC016995.3 | -1.874 | 3.78E-74 |
| AC018766.4 | -1.141 | 2.13E-62 |
| AC024560.3 | -1.09 | 3.59E-74 |
| AC025165.8 | -1.115 | 6.40E-223 |
| AC055736.1 | -1.727 | 6.61E-11 |
| AC066694.1 | 2.866 | 5.61E-13 |
| AC073850.6 | -3.173 | 1.26E-274 |
| AC074289.1 | -1.845 | 3.53E-248 |
| AC083843.1 | -1.306 | 3.60E-160 |
| AC093110.3 | -2.206 | 0.00E+00 |
| AC093495.4 | -1.257 | 6.94E-122 |
| AC093850.2 | 3.036 | 5.17E-180 |
| AC097724.3 | -1.752 | 7.11E-179 |
| AC104667.3 | 1.113 | 5.81E-100 |
| AC113188.2 | 1.034 | 8.05E-44 |
| AC116035.1 | -1.091 | 8.37E-289 |
| AC116614.1 | -1.084 | 2.90E-119 |
| AC129492.1 | -1.091 | 3.18E-42 |
| AC129778.2 | -1.056 | 5.61E-203 |
| AC132217.4 | -2.313 | 1.54E-67 |
| AC135178.7 | -1.118 | 1.56E-233 |
| AC138035.2 | -1.274 | 4.47E-81 |
| AC144530.1 | 1.428 | 3.20E-54 |
| AC234582.1 | 1.66 | 3.80E-26 |
| AC239868.2 | 2.721 | 1.64E-116 |
| AC239868.3 | 2.713 | 2.35E-114 |
| ACACB | -4.028 | 2.71E-302 |
| ACAD11 | -1.582 | 1.18E-212 |
| ACADL | -2.508 | 1.22E-256 |
| ACADS | -1.579 | 5.37E-121 |
| ACADVL | -1.806 | 1.20E-163 |
| ACAP3 | -1.171 | 2.49E-42 |
| ACCS | -1.811 | 1.14E-169 |
| ACKR1 | -3.855 | 3.85E-164 |
| ACKR3 | -1.684 | 7.65E-84 |
| ACKR4 | -1.108 | 7.86E-58 |
| ACO1 | -1.085 | 2.41E-105 |
| ACOT4 | 1.055 | 1.25E-51 |
| ACOT7 | 1.288 | 1.23E-124 |
| ACP5 | 1.242 | 3.29E-65 |
| ACR | -1.029 | 3.84E-162 |
| ACSL1 | -2.099 | 1.24E-121 |
| ACSL3 | 1.131 | 8.87E-59 |
| ACSL4 | -1.068 | 2.50E-67 |
| ACSL5 | -1.178 | 8.11E-67 |
| ACSM5 | -2.23 | 1.45E-251 |
| ACSS2 | -1.393 | 4.16E-200 |
| ACTA1 | -1.123 | 5.00E-16 |
| ACTA2 | -1.693 | 1.06E-76 |
| ACTA2-AS1 | -3.699 | 1.10E-247 |
| ACTG2 | -3.719 | 4.64E-82 |
| ACTR2 | 1.139 | 7.25E-91 |
| ACTR3 | 1.043 | 7.49E-57 |
| ACVR1C | -1.895 | 1.42E-188 |
| ACVRL1 | -1.758 | 9.56E-197 |
| ADAM12 | 1.499 | 1.73E-44 |
| ADAM33 | -4.222 | 0.00E+00 |
| ADAM8 | 1.228 | 5.96E-68 |
| ADAMDEC1 | 1.539 | 2.49E-65 |
| ADAMTS1 | -2.952 | 2.57E-202 |
| ADAMTS10 | -2.272 | 6.87E-133 |
| ADAMTS4 | -1.063 | 5.19E-32 |
| ADAMTS5 | -2.48 | 9.68E-285 |
| ADAMTS9 | -1.921 | 1.55E-184 |
| ADAMTS9-AS1 | -2.032 | 9.33E-286 |
| ADAMTS9-AS2 | -1.809 | 0.00E+00 |
| ADAMTSL4 | -2.984 | 3.90E-248 |
| ADAT2 | -1.185 | 5.71E-123 |
| ADCK2 | 1.04 | 1.00E-154 |
| ADCY3 | -1.488 | 2.42E-131 |
| ADCY4 | -3.558 | 1.72E-298 |
| ADCY5 | -1.924 | 7.16E-67 |
| ADCYAP1R1 | -1.677 | 6.12E-260 |
| ADD3 | -1.652 | 4.03E-107 |
| ADGRA2 | -1.627 | 4.48E-116 |
| ADGRB2 | 1.369 | 8.06E-45 |
| ADGRD1 | -2.166 | 4.19E-218 |
| ADGRF5 | -2.086 | 2.36E-203 |
| ADGRG3 | -1.205 | 3.19E-190 |
| ADGRL2 | -1.366 | 8.08E-87 |
| ADGRL3 | -1.334 | 1.28E-136 |
| ADGRL4 | -1.883 | 7.11E-165 |
| ADH1A | -1.007 | 6.82E-228 |
| ADH1B | -6.669 | 3.50E-250 |
| ADH1C | -3.553 | 2.32e-310 |
| ADH4 | -1.122 | 4.73E-203 |
| ADHFE1 | -2.321 | 1.18E-212 |
| ADIPOQ | -5.615 | 5.77E-150 |
| ADIPOR1 | 1.113 | 6.89E-137 |
| ADIRF | -1.683 | 3.19E-58 |
| ADIRF-AS1 | -2.09 | 3.66E-208 |
| ADM | -3.568 | 8.62E-197 |
| ADM2 | 1.563 | 1.34E-139 |
| ADM5 | -1.331 | 6.61E-127 |
| ADRA1A | -1.41 | 1.53E-270 |
| ADRA2A | -1.889 | 8.18E-102 |
| ADRA2C | -1.204 | 1.13E-43 |
| ADRB2 | -2.477 | 0.00E+00 |
| AFAP1L1 | -1.748 | 2.05E-199 |
| AFAP1L2 | -1.016 | 1.88E-30 |
| AGAP11 | -2.762 | 0.00E+00 |
| AGAP4 | -1.041 | 1.62E-81 |
| AGAP6 | -1.304 | 1.48E-89 |
| AGAP9 | -1.311 | 5.26E-98 |
| AGER | -1.332 | 5.25E-87 |
| AGPAT2 | -1.491 | 2.88E-84 |
| AGPAT9 | -1.123 | 1.31E-85 |
| AGR2 | 4.086 | 1.32E-65 |
| AGR3 | 4.189 | 1.49E-44 |
| AGTR1 | -2.984 | 9.96E-57 |
| AHCY | 1.171 | 2.89E-141 |
| AHSA2 | -1.864 | 2.93E-114 |
| AIF1L | -1.433 | 8.57E-48 |
| AIFM2 | -1.406 | 4.27E-138 |
| AK5 | -2.457 | 3.01E-36 |
| AKAP12 | -2.727 | 6.00E-210 |
| AKAP17A | -1.041 | 7.10E-74 |
| AKAP2 | -1.706 | 3.28E-135 |
| AKR1C1 | -4.265 | 2.55E-199 |
| AKR1C2 | -4.632 | 3.80E-160 |
| AKR1C3 | -2.612 | 1.66E-152 |
| AKR7A3 | 2.054 | 9.62E-35 |
| AKT3 | -1.357 | 2.23E-67 |
| AL035610.1 | -2.396 | 9.02E-306 |
| AL158801.1 | 1.549 | 2.18E-103 |
| AL162151.3 | -1.508 | 5.34E-06 |
| AL442127.1 | -1.952 | 8.74E-62 |
| ALB | -1.455 | 9.85E-24 |
| ALCAM | 1.351 | 1.69E-39 |
| ALDH18A1 | 1.62 | 2.90E-213 |
| ALDH1A1 | -2.521 | 1.54E-162 |
| ALDH1A2 | -2.025 | 2.28E-226 |
| ALDH1A3 | -1.523 | 5.42E-56 |
| ALDH1L1 | -3.384 | 3.22E-283 |
| ALDH2 | -1.346 | 5.87E-75 |
| ALDH3A1 | -1.322 | 5.83E-78 |
| ALDH3B2 | 1.183 | 8.45E-24 |
| ALDOC | -1.827 | 1.05E-90 |
| ALG1 | 1.204 | 4.36E-177 |
| ALOX15B | -1.046 | 4.88E-05 |
| ALPL | -1.741 | 6.99E-79 |
| ALS2CL | -1.73 | 7.23E-171 |
| ALX4 | -1.329 | 6.96E-95 |
| AMACR | 1.244 | 2.99E-96 |
| AMOTL1 | -1.155 | 3.69E-80 |
| AMOTL2 | -1.499 | 7.57E-134 |
| AMT | -2.309 | 5.34E-177 |
| AMY2B | -2.594 | 1.57E-207 |
| ANGPT1 | -1.58 | 4.14E-131 |
| ANGPT4 | -1.594 | 2.80E-296 |
| ANGPTL1 | -3.164 | 2.47E-306 |
| ANGPTL2 | -1.468 | 2.02E-93 |
| ANGPTL4 | -3.45 | 1.91E-169 |
| ANGPTL7 | -1.951 | 4.76E-218 |
| ANK2 | -1.934 | 7.33E-139 |
| ANKDD1A | -1.331 | 1.66E-216 |
| ANKEF1 | 1.203 | 3.45E-80 |
| ANKRD10-IT1 | -1.097 | 7.69E-58 |
| ANKRD22 | 1.874 | 3.01E-125 |
| ANKRD29 | -2.474 | 1.58E-205 |
| ANKRD35 | -1.529 | 1.84E-130 |
| ANKRD36 | -1.011 | 3.72E-99 |
| ANKRD36B | -1.021 | 5.26E-90 |
| ANKRD36C | -1.073 | 2.61E-75 |
| ANKRD53 | -1.688 | 4.69e-315 |
| ANKRD65 | -1.179 | 4.87E-67 |
| ANKS6 | -1.007 | 2.27E-37 |
| ANLN | 2.79 | 6.38E-216 |
| ANO2 | -1.431 | 1.31E-98 |
| ANOS1 | 1.085 | 4.25E-36 |
| ANPEP | -2.376 | 2.50E-63 |
| ANTXR2 | -1.555 | 9.14E-122 |
| ANXA1 | -2.173 | 1.15E-166 |
| ANXA3 | -1.598 | 1.90E-62 |
| ANXA8 | -1.029 | 2.92E-05 |
| ANXA8L1 | -1.174 | 7.50E-14 |
| ANXA9 | 2.593 | 2.91E-77 |
| AOC2 | -1.124 | 1.85E-200 |
| AOC3 | -3.552 | 3.46E-239 |
| AOC4P | -1.594 | 1.66E-286 |
| AOX1 | -3.091 | 4.47E-204 |
| AP000275.65 | 1.734 | 1.75E-81 |
| AP000347.2 | -1.111 | 4.56E-140 |
| AP000349.2 | 4.561 | 7.37E-163 |
| AP000439.3 | 1.213 | 4.59E-40 |
| AP000473.5 | -1.061 | 1.54E-174 |
| AP000892.6 | -2.253 | 1.48E-194 |
| AP001189.4 | -1.319 | 4.63E-256 |
| AP003391.1 | 3.729 | 1.42E-33 |
| AP004372.1 | 1.057 | 6.55E-34 |
| AP1M2 | 2.201 | 3.19E-201 |
| AP1S1 | 1.304 | 5.50E-182 |
| AP1S3 | 1.196 | 2.08E-75 |
| APBB3 | -1.46 | 1.36E-101 |
| APCDD1 | -2.15 | 9.32E-154 |
| APCDD1L-AS1 | -1.025 | 2.22E-36 |
| APITD1 | 1.186 | 1.08E-155 |
| APOA1BP | 1.452 | 2.30E-229 |
| APOBEC3B | 2.344 | 1.03E-120 |
| APOBR | 1.109 | 4.72E-67 |
| APOC1 | 1.657 | 6.43E-66 |
| APOC2 | 2.046 | 5.31E-104 |
| APOC4-APOC2 | 2.148 | 6.24E-116 |
| APOD | -3.435 | 4.53E-102 |
| APOL3 | -1.393 | 1.98E-88 |
| APOL4 | -1.065 | 3.65E-55 |
| APOLD1 | -2.611 | 1.62E-185 |
| APOO | 1.332 | 2.62E-199 |
| AQP1 | -2.365 | 6.65E-160 |
| AQP5 | -1.492 | 1.59E-06 |
| AQP7 | -5.364 | 3.29E-278 |
| AQP7P1 | -2.58 | 1.95E-292 |
| AQP7P2 | -1.556 | 2.99E-219 |
| AR | 1.115 | 3.67E-08 |
| ARAP1-AS1 | 2.172 | 1.59E-20 |
| ARAP3 | -1.717 | 2.06E-224 |
| ARF1 | 1.015 | 1.35E-192 |
| ARF3 | 1.184 | 1.11E-114 |
| ARFGEF3 | 1.822 | 1.01E-70 |
| ARGLU1 | -1.777 | 1.59E-143 |
| ARHGAP10 | -1.694 | 1.81E-192 |
| ARHGAP11A | 1.803 | 1.52E-126 |
| ARHGAP20 | -1.861 | 0.00E+00 |
| ARHGAP23 | -1.168 | 2.32E-91 |
| ARHGAP24 | -1.164 | 4.91E-109 |
| ARHGAP26 | -1.204 | 3.36E-77 |
| ARHGAP39 | 1.004 | 8.76E-81 |
| ARHGAP40 | -2.329 | 3.99E-29 |
| ARHGAP6 | -1.696 | 7.08E-197 |
| ARHGEF1 | -1.004 | 2.77E-28 |
| ARHGEF10 | -1.134 | 8.14E-106 |
| ARHGEF15 | -2.409 | 1.44E-239 |
| ARHGEF17 | -1.401 | 3.54E-96 |
| ARHGEF25 | -1.328 | 1.67E-130 |
| ARHGEF28 | -1.043 | 7.32E-73 |
| ARHGEF4 | -1.458 | 3.51E-95 |
| ARHGEF40 | -1.267 | 2.06E-154 |
| ARHGEF7 | -1.154 | 4.16E-139 |
| ARID5A | -1.721 | 1.78E-102 |
| ARID5B | -1.347 | 3.11E-104 |
| ARL1 | 1.065 | 4.56E-94 |
| ARL4A | -1.232 | 6.35E-78 |
| ARL6IP1 | 1.421 | 3.62E-137 |
| ARMC9 | 1.176 | 1.02E-121 |
| ARMCX4 | -1.228 | 3.57E-105 |
| ARMT1 | 1.515 | 2.43E-46 |
| ARNT2 | 1.982 | 1.64E-82 |
| ARPC4-TTLL3 | -1.006 | 1.05E-78 |
| ARRDC2 | -1.317 | 8.00E-105 |
| ARRDC3 | -1.262 | 8.33E-54 |
| ARSG | 1.042 | 2.39E-23 |
| ARTN | 1.085 | 1.51E-42 |
| ARV1 | 1.188 | 5.81E-133 |
| ASF1B | 3.121 | 2.78E-306 |
| ASMTL-AS1 | -2.056 | 1.12E-95 |
| ASPA | -1.69 | 5.74E-259 |
| ASPM | 2.386 | 2.78E-203 |
| ASPN | 2.189 | 1.63E-58 |
| ASS1 | -1.011 | 4.04E-31 |
| ATAD2 | 1.979 | 5.21E-132 |
| ATAD3B | -1.352 | 6.31E-41 |
| ATF3 | -2.232 | 3.80E-72 |
| ATHL1 | -1.476 | 5.52E-30 |
| ATIC | 1.165 | 7.14E-195 |
| ATOH8 | -3.881 | 0.00E+00 |
| ATP1A2 | -3.5 | 4.79E-290 |
| ATP1B1 | 1.02 | 1.16E-46 |
| ATP1B2 | -1.192 | 1.34E-164 |
| ATP2C2 | 1.086 | 1.23E-44 |
| ATP6AP1 | 1.385 | 7.86E-180 |
| ATP6V0B | 1.214 | 5.16E-192 |
| ATP6V0E2 | 1.265 | 1.36E-89 |
| ATP6V1A | 1.065 | 2.71E-77 |
| ATP8B1 | 1.046 | 1.12E-39 |
| ATP8B4 | -1.003 | 4.30E-110 |
| AUNIP | 1.269 | 1.93E-141 |
| AURKA | 2.778 | 1.36E-225 |
| AURKB | 2.67 | 5.18E-193 |
| AVPI1 | -1.084 | 9.74E-102 |
| AVPR1A | -1.441 | 2.45E-154 |
| AVPR2 | -2.146 | 1.09E-289 |
| AZIN1 | 1.15 | 8.14E-59 |
| B3GALNT1 | 1.005 | 2.46E-70 |
| B4GALNT3 | 1.555 | 2.80E-64 |
| B4GALNT4 | 1.666 | 1.49E-81 |
| BAALC | -1.545 | 1.19E-72 |
| BAHCC1 | -1.07 | 3.28E-58 |
| BAK1 | 1.032 | 4.71E-143 |
| BAMBI | 1.741 | 4.98E-46 |
| BATF | 1.432 | 2.35E-55 |
| BBOX1 | -2.349 | 1.25E-34 |
| BCAS1 | 1.867 | 5.72E-37 |
| BCAS4 | 2.201 | 4.73E-142 |
| BCHE | -1.248 | 1.06E-47 |
| BCL2L2-PABPN1 | -1.009 | 4.23E-27 |
| BCL6 | -2.315 | 4.72E-213 |
| BCL9 | 1.351 | 2.82E-95 |
| BEGAIN | -1.005 | 2.92E-68 |
| BEND5 | -1.127 | 1.30E-71 |
| BEND7 | -1.455 | 1.63E-71 |
| BEST1 | -1.074 | 7.44E-143 |
| BGN | 2.153 | 1.09E-147 |
| BHMT2 | -2.613 | 3.03E-236 |
| BICC1 | -1.037 | 1.71E-57 |
| BIK | 1.832 | 8.45E-103 |
| BIN1 | -1.66 | 1.11E-128 |
| BIRC5 | 3.409 | 7.46E-244 |
| BLM | 1.342 | 5.79E-130 |
| BLNK | 1.401 | 1.60E-105 |
| BMP2 | -1.681 | 8.75E-199 |
| BMP6 | -2.097 | 2.57E-207 |
| BMPER | -1.444 | 2.23E-128 |
| BMPR1APS2 | 1.119 | 8.12E-16 |
| BMPR1B | 1.835 | 2.33E-45 |
| BMX | -1.768 | 2.03E-268 |
| BOC | -2.241 | 2.41E-136 |
| BOLA2 | 1.169 | 8.26E-74 |
| BOLA2B | 1.627 | 1.60E-201 |
| bP-21264C1.1 | -1.468 | 1.55E-237 |
| bP-2189O9.2 | -1.102 | 1.52E-256 |
| BPIFB1 | 1.814 | 1.80E-41 |
| BPNT1 | 1.341 | 8.90E-117 |
| BRCA1 | 1.113 | 1.42E-80 |
| BRCC3 | 1.127 | 9.24E-97 |
| BRI3BP | 1.693 | 2.85E-149 |
| BRICD5 | -1.076 | 3.26E-25 |
| BRINP1 | -1.016 | 1.67E-26 |
| BRIP1 | 1.132 | 4.64E-102 |
| BROX | 1.113 | 5.64E-89 |
| BSCL2 | 1.215 | 8.72E-104 |
| BSPRY | 1.14 | 1.44E-99 |
| BST2 | 1.048 | 1.93E-20 |
| BTAF1 | -1.243 | 5.59E-99 |
| BTBD19 | -1.159 | 3.75E-95 |
| BTNL9 | -5.207 | 0.00E+00 |
| BUB1 | 2.65 | 2.75E-218 |
| BUB1B | 2.239 | 2.20E-198 |
| BUB3 | 1.042 | 2.95E-105 |
| BZW1P2 | 1.299 | 5.74E-25 |
| BZW2 | 1.124 | 9.37E-118 |
| C10orf10 | -3.464 | 1.86E-234 |
| C10orf128 | -2.061 | 4.52E-180 |
| C10orf35 | 1.138 | 1.74E-112 |
| C10orf54 | -2.051 | 4.84E-194 |
| C10orf90 | -1.322 | 1.24E-106 |
| C11orf53 | -1.03 | 5.30E-141 |
| C11orf96 | -2.665 | 3.83E-150 |
| C14orf180 | -3.307 | 1.20E-231 |
| C15orf48 | 2.883 | 1.56E-117 |
| C15orf52 | -1.261 | 1.16E-41 |
| C16orf59 | 1.803 | 2.08E-187 |
| C16orf71 | 1.233 | 8.95E-85 |
| C16orf86 | -1.35 | 4.14E-152 |
| C16orf89 | -1.937 | 3.05E-24 |
| C19orf33 | 2.056 | 1.13E-27 |
| C19orf48 | 1.063 | 3.81E-113 |
| C19orf80 | -1.614 | 3.42E-158 |
| C1orf233 | 1.981 | 1.82E-164 |
| C1orf43 | 1.147 | 3.18E-169 |
| C1orf64 | 1.117 | 7.52E-13 |
| C1orf95 | -1.247 | 1.39E-39 |
| C1QTNF1 | -2.496 | 1.55E-171 |
| C1QTNF6 | 1.66 | 1.65E-117 |
| C1QTNF7 | -1.499 | 3.14E-137 |
| C1R | -1.333 | 8.98E-80 |
| C1S | -1.385 | 6.27E-65 |
| C20orf194 | -1.029 | 7.49E-131 |
| C2CD2 | -1.185 | 2.09E-115 |
| C2CD4B | -1.188 | 1.05E-75 |
| C2orf15 | 1.411 | 3.91E-120 |
| C2orf40 | -4.193 | 2.77E-193 |
| C2orf54 | 1.379 | 1.76E-43 |
| C2orf82 | -1.681 | 6.69E-30 |
| C2orf88 | -1.611 | 1.31E-138 |
| C3 | -1.042 | 1.33E-37 |
| C3orf14 | 1.561 | 1.05E-70 |
| C3orf52 | 1.436 | 3.39E-63 |
| C3orf67 | 1.098 | 1.35E-100 |
| C3orf80 | 1.232 | 8.96E-121 |
| C4A | 1.794 | 3.86E-28 |
| C4B | 1.719 | 2.84E-28 |
| C5AR2 | 1.215 | 1.16E-26 |
| C5orf30 | 1.006 | 6.31E-40 |
| C6 | -1.694 | 6.01E-117 |
| C6orf132 | 1.092 | 5.35E-103 |
| C6orf99 | 1.166 | 5.40E-134 |
| C7 | -3.359 | 3.84E-120 |
| C8orf46 | -1.042 | 2.58E-36 |
| C8orf88 | -1.791 | 1.16E-233 |
| C9orf116 | 2.041 | 1.55E-106 |
| C9orf152 | 1.425 | 6.34E-32 |
| CA12 | 2.227 | 1.15E-35 |
| CA3 | -1.88 | 9.06E-118 |
| CA4 | -5.024 | 0.00E+00 |
| CABP1 | -1.441 | 8.12E-264 |
| CABYR | 1.056 | 8.16E-52 |
| CACFD1 | 1.44 | 6.70E-134 |
| CACHD1 | -1.994 | 9.85E-192 |
| CACNG4 | 2.654 | 5.48E-65 |
| CACYBP | 1.111 | 7.30E-125 |
| CADM3 | -2.331 | 6.48E-170 |
| CADM3-AS1 | -1.568 | 5.33E-289 |
| CADM4 | 1.093 | 6.71E-74 |
| CAHM | -1.136 | 2.02E-203 |
| CALB2 | -2.643 | 2.35E-76 |
| CALCOCO1 | -1.41 | 6.64E-176 |
| CALR | 1.244 | 3.10E-193 |
| CAMK1 | -1.491 | 1.20E-165 |
| CANT1 | 1.823 | 5.70E-135 |
| CAPG | 1.418 | 1.03E-114 |
| CAPN11 | -2.02 | 1.67E-286 |
| CAPN13 | 1.97 | 1.20E-62 |
| CAPN3 | -2.291 | 5.44E-213 |
| CAPN6 | -1.883 | 1.25E-42 |
| CAPN9 | 1.431 | 3.38E-65 |
| CAPRIN1 | 1.032 | 3.71E-91 |
| CAPS | 1.088 | 2.30E-38 |
| CASC5 | 1.432 | 1.56E-158 |
| CASK | 1.039 | 5.91E-64 |
| CASP10 | -1.002 | 2.29E-72 |
| CASP6 | 1.005 | 3.65E-93 |
| CASQ2 | -1.976 | 1.23E-226 |
| CAT | -1.464 | 6.27E-161 |
| CAV1 | -3.01 | 7.51E-252 |
| CAV2 | -2.657 | 5.06E-234 |
| CBLC | 1.186 | 4.97E-70 |
| CBX3 | 1.002 | 4.54E-148 |
| CBX4 | 1.187 | 3.89E-108 |
| CBX7 | -1.788 | 1.47E-157 |
| CCDC117 | 1.018 | 1.97E-58 |
| CCDC125 | 1.216 | 6.78E-55 |
| CCDC130 | -1.174 | 3.40E-61 |
| CCDC136 | -1.05 | 2.84E-81 |
| CCDC152 | -1.184 | 2.80E-100 |
| CCDC160 | 1.257 | 9.45E-39 |
| CCDC167 | 1.795 | 1.26E-245 |
| CCDC178 | -2.201 | 1.34E-286 |
| CCDC3 | -2.123 | 5.30E-158 |
| CCDC34 | 1.056 | 4.11E-70 |
| CCDC39 | -1.177 | 3.61E-159 |
| CCDC47 | 1.024 | 3.29E-45 |
| CCDC64 | 2.274 | 2.24E-204 |
| CCDC68 | -1.261 | 2.59E-93 |
| CCDC69 | -2.638 | 1.08E-212 |
| CCDC78 | 1.375 | 1.96E-52 |
| CCDC8 | -1.394 | 3.76E-38 |
| CCDC80 | -1.043 | 2.93E-31 |
| CCDC82 | -1.85 | 2.53E-167 |
| CCDC84 | -1.51 | 4.09E-125 |
| CCDC85A | -1.322 | 4.84E-156 |
| CCDC85B | -1.254 | 8.22E-36 |
| CCDC85C | 1.19 | 3.86E-83 |
| CCDC96 | 1.034 | 4.62E-65 |
| CCL11 | 1.048 | 1.86E-101 |
| CCL14 | -4.182 | 1.19E-192 |
| CCL15-CCL14 | -1.134 | 7.29E-96 |
| CCL21 | -2.98 | 1.49E-75 |
| CCL23 | -1.447 | 7.19E-175 |
| CCL28 | -2.605 | 3.23E-48 |
| CCL3L3 | 1.141 | 1.29E-34 |
| CCL4L2 | 1.896 | 1.70E-60 |
| CCL5 | 1.405 | 2.97E-45 |
| CCM2L | -1.737 | 1.99E-218 |
| CCNA2 | 2.097 | 1.23E-165 |
| CCNB1 | 2.982 | 1.49E-287 |
| CCNB2 | 3.117 | 8.72E-248 |
| CCND1 | 1.465 | 3.67E-44 |
| CCNE1 | 1.06 | 2.33E-68 |
| CCNE2 | 1.761 | 3.66E-115 |
| CCNF | 1.763 | 9.79E-209 |
| CCNL1 | -1.476 | 1.94E-166 |
| CCNL2 | -1.936 | 1.94E-121 |
| CCNO | 1.131 | 1.47E-30 |
| CCR10 | -1.585 | 1.54E-159 |
| CCR5 | 1.348 | 5.41E-69 |
| CCT3 | 1.647 | 8.31E-260 |
| CD2 | 1.507 | 2.97E-49 |
| CD200 | -1.025 | 6.95E-55 |
| CD209 | -1.391 | 8.34E-108 |
| CD24 | 2.046 | 3.15E-83 |
| CD248 | -1.976 | 1.74E-132 |
| CD24P4 | 1.233 | 5.22E-31 |
| CD300LG | -5.376 | 0.00E+00 |
| CD302 | -1.379 | 1.26E-106 |
| CD34 | -1.985 | 1.50E-183 |
| CD36 | -4.669 | 2.42E-186 |
| CD3D | 1.128 | 8.97E-35 |
| CD3E | 1.11 | 5.04E-27 |
| CD3EAP | 1.156 | 2.31E-125 |
| CD48 | 1.463 | 4.41E-51 |
| CD52 | 1.168 | 9.72E-28 |
| CD86 | 1.106 | 9.58E-69 |
| CD9 | 1.131 | 4.64E-110 |
| CDC14B | -1.544 | 1.01E-183 |
| CDC20 | 3.375 | 1.79E-233 |
| CDC25A | 1.215 | 4.02E-110 |
| CDC25C | 1.965 | 3.58E-212 |
| CDC42EP3 | -1.198 | 3.72E-71 |
| CDC42EP5 | -1.131 | 3.58E-47 |
| CDC45 | 2.177 | 1.98E-176 |
| CDC6 | 2.478 | 7.94E-153 |
| CDCA2 | 1.624 | 1.43E-134 |
| CDCA3 | 2.401 | 1.51E-183 |
| CDCA4 | 1.12 | 2.67E-148 |
| CDCA5 | 2.686 | 4.04E-222 |
| CDCA8 | 2.452 | 1.38E-215 |
| CDCP1 | 1.423 | 5.80E-92 |
| CDH1 | 1.729 | 3.67E-67 |
| CDH11 | 1.545 | 4.36E-48 |
| CDH13 | -1.135 | 2.34E-69 |
| CDH23 | -1.466 | 3.74E-153 |
| CDH5 | -1.865 | 2.78E-175 |
| CDK1 | 2.842 | 1.21E-238 |
| CDK11A | -1.552 | 6.15E-91 |
| CDK3 | -1.323 | 9.82E-88 |
| CDK5 | 1.475 | 1.67E-238 |
| CDKN1C | -2.448 | 7.67E-228 |
| CDKN2A | 1.55 | 1.85E-76 |
| CDKN2C | -1.107 | 3.01E-85 |
| CDKN3 | 2.662 | 3.67E-225 |
| CDO1 | -3.221 | 4.22E-255 |
| CDRT4 | -1.343 | 1.08E-71 |
| CDS1 | 1.702 | 2.91E-141 |
| CDT1 | 2.051 | 1.48E-159 |
| CEACAM19 | -1.078 | 1.85E-53 |
| CEACAM5 | 1.576 | 5.38E-45 |
| CEACAM6 | 3.109 | 5.83E-67 |
| CEBPA | -1.759 | 3.10E-101 |
| CEBPD | -2.377 | 6.62E-128 |
| CECR7 | -1.242 | 2.98E-59 |
| CELF2 | -1.667 | 1.36E-131 |
| CELF6 | -1.501 | 8.59E-173 |
| CELSR1 | 2.055 | 6.88E-93 |
| CENPA | 2.164 | 5.91E-155 |
| CENPE | 1.46 | 1.20E-137 |
| CENPF | 3.872 | 3.86E-285 |
| CENPH | 1.163 | 3.18E-121 |
| CENPI | 1.322 | 8.76E-120 |
| CENPK | 1.407 | 6.44E-122 |
| CENPL | 1.225 | 6.47E-134 |
| CENPM | 2.53 | 1.34E-247 |
| CENPN | 1.491 | 2.26E-111 |
| CENPP | 1.087 | 5.29E-84 |
| CENPT | -1.738 | 6.17E-148 |
| CENPU | 2.369 | 1.41E-214 |
| CENPW | 1.025 | 2.26E-63 |
| CEP112 | -1.522 | 1.49E-195 |
| CEP55 | 2.787 | 6.23E-228 |
| CERS2 | 1.505 | 8.77E-123 |
| CERS4 | 1.078 | 6.92E-40 |
| CERS6 | 1.862 | 9.97E-85 |
| CERS6-AS1 | 1.692 | 7.60E-54 |
| CES1 | -2.902 | 3.71E-132 |
| CES4A | -1.031 | 3.71E-67 |
| CETN2 | 1.12 | 2.12E-149 |
| CFAP69 | -1.034 | 1.81E-58 |
| CFB | 2.27 | 1.22E-47 |
| CFD | -5.165 | 2.15E-231 |
| CFH | -1.522 | 4.34E-93 |
| CFI | -1.415 | 6.00E-84 |
| CFL1 | 1.046 | 1.72E-194 |
| CFL2 | -1.574 | 9.02E-165 |
| CFLAR | -1.317 | 1.94E-122 |
| CFLAR-AS1 | -1.033 | 1.01E-192 |
| CH17-13I23.3 | -1.329 | 7.81E-84 |
| CH17-472G23.4 | -1.266 | 3.01E-149 |
| CH507-42P11.8 | -1.614 | 3.61E-53 |
| CH507-513H4.5 | 1.698 | 8.36E-53 |
| CHAF1B | 1.165 | 5.79E-123 |
| CHEK1 | 1.556 | 2.10E-141 |
| CHI3L1 | -1.276 | 7.26E-11 |
| CHKB | -1.477 | 1.08E-132 |
| CHL1 | -2.525 | 1.50E-203 |
| CHL1-AS2 | -1.364 | 3.67E-304 |
| CHPF | 1.362 | 5.80E-144 |
| CHRD | -1.162 | 7.44E-15 |
| CHRDL1 | -4.297 | 9.99E-249 |
| CHST2 | -1.108 | 1.38E-64 |
| CHST3 | -1.538 | 6.12E-97 |
| CHST7 | -1.284 | 2.18E-216 |
| CHST9 | -1.082 | 7.31E-39 |
| CIB1 | 1.008 | 7.14E-93 |
| CICP14 | -1.16 | 4.44E-81 |
| CIDEA | -5.052 | 7.51E-199 |
| CIDEC | -6.453 | 1.73E-220 |
| CILP2 | 1.619 | 5.91E-118 |
| CISD2 | 1.046 | 1.38E-139 |
| CITED1 | -1.375 | 6.56E-23 |
| CKAP2 | 1.739 | 1.04E-157 |
| CKAP2L | 1.852 | 1.94E-166 |
| CKAP4 | 1.404 | 9.73E-164 |
| CKAP5 | 1.183 | 1.11E-79 |
| CKMT1A | 1.517 | 1.08E-44 |
| CKMT1B | 1.291 | 4.89E-38 |
| CKMT2 | -1.598 | 1.97E-177 |
| CKS2 | 3.053 | 4.09e-320 |
| CLASRP | -1.336 | 3.11E-74 |
| CLCN3 | 1.069 | 3.37E-32 |
| CLCN6 | -1.351 | 2.79E-193 |
| CLDN11 | -2.671 | 1.03E-155 |
| CLDN15 | -1.815 | 1.90E-158 |
| CLDN19 | -1.14 | 7.29E-94 |
| CLDN3 | 1.189 | 6.58E-70 |
| CLDN5 | -4.71 | 2.81E-299 |
| CLDN7 | 1.381 | 2.32E-114 |
| CLDN8 | -2.239 | 1.80E-36 |
| CLDND2 | -1.414 | 1.24E-180 |
| CLEC10A | -1.082 | 7.47E-26 |
| CLEC14A | -1.695 | 5.29E-162 |
| CLEC1A | -1.897 | 1.18E-265 |
| CLEC2B | -1.079 | 1.07E-93 |
| CLEC3B | -3.616 | 6.86E-261 |
| CLEC4G | -1.067 | 3.12E-179 |
| CLEC5A | 1.215 | 4.77E-87 |
| CLGN | 1.954 | 3.65E-62 |
| CLIC2 | -1.225 | 2.47E-96 |
| CLIC5 | -1.5 | 1.72E-147 |
| CLIP4 | -1.875 | 2.69E-134 |
| CLK1 | -1.551 | 9.82E-152 |
| CLK4 | -1.304 | 1.19E-146 |
| CLMP | -1.623 | 1.97E-129 |
| CLPSL1 | 1.368 | 1.07E-44 |
| CLPSL2 | 1.849 | 1.59E-78 |
| CLSPN | 1.5 | 3.10E-126 |
| CLTC | 1.173 | 5.06E-106 |
| CLTCL1 | -1.254 | 1.65E-111 |
| CMA1 | -1.659 | 7.04E-102 |
| CMAHP | -1.258 | 1.92E-53 |
| CMBL | 1.486 | 3.19E-32 |
| CMTM7 | -1.123 | 2.71E-30 |
| CMYA5 | -1.094 | 9.49E-26 |
| CNGA1 | -1.099 | 1.55E-58 |
| CNIH2 | 1.452 | 6.99E-110 |
| CNN1 | -3.08 | 8.57E-134 |
| CNR1 | -2.032 | 2.90E-182 |
| CNRIP1 | -1.938 | 6.42E-197 |
| CNTD2 | 1.247 | 2.09E-51 |
| CNTFR | -3.699 | 4.87E-206 |
| CNTN3 | -1.059 | 1.23E-63 |
| CNTNAP1 | -1.076 | 3.44E-91 |
| CNTNAP2 | 1.863 | 3.46E-69 |
| CNTNAP3 | -2.015 | 4.97E-206 |
| CNTNAP3B | -3.249 | 0.00E+00 |
| COA6 | 1.267 | 4.14E-194 |
| COL10A1 | 5.934 | 7.31E-269 |
| COL11A1 | 4.668 | 9.11E-185 |
| COL14A1 | -1.739 | 7.39E-50 |
| COL17A1 | -3.023 | 4.71E-41 |
| COL1A1 | 2.846 | 4.89E-113 |
| COL1A2 | 1.858 | 1.39E-55 |
| COL25A1 | -1.466 | 5.23E-275 |
| COL27A1 | -1.338 | 2.86E-46 |
| COL3A1 | 2.287 | 5.96E-72 |
| COL4A6 | -1.361 | 3.50E-49 |
| COL5A1 | 1.649 | 4.56E-59 |
| COL5A2 | 1.681 | 3.79E-58 |
| COL5A3 | -1.194 | 3.32E-36 |
| COL6A6 | -1.322 | 1.72E-129 |
| COL7A1 | -1.112 | 3.26E-20 |
| COL8A1 | 1.767 | 1.95E-57 |
| COLCA1 | -1.09 | 1.70E-65 |
| COLCA2 | -1.459 | 9.17E-109 |
| COMP | 3.396 | 9.04E-123 |
| COPA | 1.263 | 2.39E-102 |
| COPB2 | 1.194 | 6.61E-119 |
| COPZ1 | 1.176 | 8.07E-152 |
| CORO2A | 1.668 | 7.41E-111 |
| CORO2B | -1.93 | 1.21E-281 |
| CORO6 | -2.911 | 1.21E-69 |
| COX20 | 1.046 | 7.34E-135 |
| COX4I2 | -1.118 | 9.65E-86 |
| COX6C | 1.947 | 8.10E-109 |
| COX7A1 | -2.125 | 5.14E-123 |
| COX7CP1 | -1.696 | 1.29E-106 |
| CPA1 | -1.691 | 4.29E-265 |
| CPAMD8 | -1.906 | 6.10E-96 |
| CPEB1 | -1.219 | 7.47E-132 |
| CPED1 | -1.441 | 4.41E-105 |
| CPLX1 | -1.391 | 2.07E-42 |
| CPM | -1.886 | 1.60E-98 |
| CPNE8 | -1.622 | 5.26E-158 |
| CPSF2 | 1.031 | 9.32E-52 |
| CPXM2 | -1.16 | 1.37E-48 |
| CRABP2 | 2.741 | 7.18E-115 |
| CRB3 | 1.819 | 9.83E-213 |
| CREB3L1 | 1.797 | 1.40E-68 |
| CREB3L4 | 1.815 | 4.55E-109 |
| CREB5 | -2.027 | 3.55E-209 |
| CRHBP | -1.294 | 2.63E-178 |
| CRIM1 | -1.491 | 3.13E-93 |
| CRIP1 | 2.566 | 6.15E-73 |
| CRLF1 | -1.236 | 2.31E-30 |
| CRNKL1 | 1.068 | 5.37E-135 |
| CROCC | -1.098 | 4.27E-43 |
| CROCCP2 | -1.13 | 2.50E-68 |
| CROCCP3 | -1.003 | 4.71E-81 |
| CRTAC1 | -1.156 | 2.04E-45 |
| CRY2 | -1.096 | 1.24E-112 |
| CRYAB | -3.584 | 2.31E-147 |
| CRYBG3 | -1.668 | 4.37E-149 |
| CSAD | -1.907 | 4.94E-84 |
| CSE1L | 1.222 | 6.10E-134 |
| CSF1 | -1.175 | 2.20E-66 |
| CSNK2B-LY6G5B-1181 | -1.574 | 6.59E-105 |
| CSPG4 | -1.129 | 1.55E-48 |
| CSPG4P12 | -1.241 | 4.31E-234 |
| CSRNP1 | -1.736 | 2.29E-145 |
| CSRP1 | -1.228 | 4.73E-60 |
| CSRP2 | -1.256 | 4.19E-56 |
| CST1 | 4.044 | 3.34E-109 |
| CST2 | 1.88 | 2.36E-83 |
| CST6 | 1.131 | 5.87E-45 |
| CSTA | -1.026 | 1.91E-11 |
| CSTF2 | 1.094 | 3.63E-138 |
| CTA-212A2.1 | -1.084 | 1.16E-271 |
| CTA-228A9.3 | -1.021 | 3.58E-92 |
| CTA-384D8.35 | 1.358 | 4.23E-69 |
| CTB-13F3.1 | -1.534 | 7.99E-184 |
| CTB-191K22.5 | 1.513 | 7.09E-44 |
| CTB-31O20.4 | -1.348 | 6.66E-160 |
| CTB-50E14.5 | -1.004 | 1.28E-127 |
| CTB-50L17.14 | -1.017 | 2.49E-56 |
| CTB-55O6.8 | 1.069 | 4.13E-49 |
| CTB-79E8.3 | -2.272 | 1.20E-115 |
| CTC-425F1.4 | 1.091 | 9.39E-14 |
| CTC-510F12.2 | -1.077 | 6.65E-251 |
| CTC-510F12.6 | 1.745 | 1.96E-93 |
| CTC-524C5.2 | -1.069 | 1.19E-89 |
| CTC-529I10.1 | -1.119 | 3.80E-122 |
| CTC-529I10.2 | -1.119 | 2.63E-131 |
| CTD-2013N24.2 | -1.003 | 6.28E-151 |
| CTD-2015G9.2 | -1.324 | 6.11E-20 |
| CTD-2015H6.3 | 1.66 | 5.69E-132 |
| CTD-2026K11.6 | -1.028 | 1.88E-235 |
| CTD-2033D15.1 | 1.255 | 1.70E-33 |
| CTD-2054N24.2 | -1.094 | 9.85E-124 |
| CTD-2135D7.5 | -1.076 | 3.45E-288 |
| CTD-2224J9.4 | 2.568 | 1.38E-22 |
| CTD-2224J9.8 | -1.267 | 2.18E-245 |
| CTD-2228K2.7 | -1.392 | 2.56E-48 |
| CTD-2231E14.8 | 1.174 | 7.98E-237 |
| CTD-2263F21.1 | -1.143 | 3.97E-159 |
| CTD-2287O16.1 | -1.197 | 5.71E-35 |
| CTD-2303H24.2 | -1.606 | 4.98E-76 |
| CTD-2373N4.3 | -1.409 | 2.37E-137 |
| CTD-2396E7.11 | 1.815 | 1.41E-182 |
| CTD-2510F5.4 | 1.315 | 2.18E-89 |
| CTD-2537I9.12 | -1.778 | 1.28E-84 |
| CTD-2540B15.11 | -1.434 | 2.03E-50 |
| CTD-3035K23.7 | -1.07 | 3.87E-134 |
| CTD-3092A11.1 | -1.348 | 7.56E-202 |
| CTD-3092A11.2 | -1.893 | 6.14E-224 |
| CTD-3141N22.1 | 1.946 | 1.74E-15 |
| CTD-3193K9.11 | -1.59 | 1.23E-94 |
| CTD-3193O13.11 | 1.79 | 2.87E-72 |
| CTD-3252C9.4 | -1.438 | 4.83E-98 |
| CTF1 | -1.005 | 1.38E-38 |
| CTHRC1 | 2.261 | 4.23E-121 |
| CTIF | -1.091 | 5.19E-112 |
| CTSD | 1.235 | 9.07E-75 |
| CTSG | -1.919 | 4.17E-62 |
| CTTNBP2 | -1.481 | 1.29E-122 |
| CTXN1 | 2.495 | 4.52E-189 |
| CX3CL1 | -2.855 | 1.10E-121 |
| CXCL1 | -1.155 | 3.89E-24 |
| CXCL10 | 3.384 | 1.24E-148 |
| CXCL11 | 2.276 | 1.59E-99 |
| CXCL12 | -2.138 | 9.72E-120 |
| CXCL13 | 2.554 | 5.01E-42 |
| CXCL14 | -1.677 | 2.21E-23 |
| CXCL2 | -3.43 | 2.12E-230 |
| CXCL3 | -1.132 | 2.45E-109 |
| CXCL9 | 2.773 | 1.45E-87 |
| CXCR3 | 1.329 | 6.83E-69 |
| CXCR4 | 1.134 | 5.42E-61 |
| CXorf36 | -2.252 | 3.11E-244 |
| CXXC5 | 1.196 | 7.00E-45 |
| CYB561 | 1.335 | 1.24E-140 |
| CYB561D2 | 1.091 | 4.57E-95 |
| CYBB | 1.05 | 5.14E-27 |
| CYBRD1 | -1.149 | 4.92E-37 |
| CYGB | -2.071 | 1.85E-181 |
| CYP11A1 | -1 | 5.13E-102 |
| CYP1B1-AS1 | -1.193 | 2.90E-156 |
| CYP26B1 | -2.1 | 1.67E-170 |
| CYP2B7P | 2.883 | 8.65E-42 |
| CYP2D7 | -1.177 | 2.08E-125 |
| CYP39A1 | -1.043 | 5.59E-23 |
| CYP3A5 | -1.326 | 2.62E-145 |
| CYP4B1 | -1.466 | 1.34E-32 |
| CYP4F12 | -2.217 | 7.96E-139 |
| CYP4F29P | -1.806 | 4.36E-61 |
| CYP51A1 | 1.224 | 8.33E-87 |
| CYR61 | -2.014 | 4.71E-81 |
| CYTH3 | -1.048 | 9.08E-84 |
| CYTL1 | -1.321 | 1.53E-101 |
| CYYR1 | -1.979 | 1.31E-170 |
| D4S234E | -1.61 | 1.60E-61 |
| DAAM2 | -2.372 | 3.84E-223 |
| DAB2 | -1.07 | 3.48E-73 |
| DAB2IP | -1.178 | 3.82E-109 |
| DAP3 | 1.185 | 2.90E-212 |
| DARS2 | 1.37 | 6.23E-132 |
| DBF4 | 1.106 | 2.16E-102 |
| DBNDD1 | 2.047 | 1.39E-177 |
| DCAF13 | 1.307 | 3.54E-139 |
| DCLK1 | -1.534 | 7.01E-43 |
| DCLRE1A | 1.097 | 5.01E-95 |
| DCN | -2.089 | 2.01E-92 |
| DCTPP1 | 1.46 | 4.10E-188 |
| DDAH1 | 1.191 | 6.13E-67 |
| DDIAS | 1.246 | 1.15E-104 |
| DDIT4 | -1.128 | 5.98E-24 |
| DDR2 | -1.686 | 2.94E-125 |
| DDX26B | -1.215 | 1.35E-99 |
| DEFB1 | -2.399 | 3.67E-41 |
| DEFB124 | -1.043 | 2.97E-156 |
| DEGS2 | 2.766 | 4.26E-70 |
| DENND1C | 1.158 | 4.52E-110 |
| DENND2A | -2.054 | 6.66E-276 |
| DENND3 | -2.1 | 9.91E-168 |
| DENND4B | -1.067 | 1.04E-69 |
| DEPDC1 | 1.724 | 5.98E-141 |
| DEPDC1B | 1.654 | 1.01E-155 |
| DERL3 | 1.334 | 3.35E-48 |
| DES | -3.103 | 4.20E-167 |
| DGAT2 | -2.447 | 2.36E-67 |
| DHCR24 | 1.646 | 3.46E-71 |
| DHCR7 | 1.264 | 8.18E-70 |
| DHFRP1 | 1.073 | 3.16E-27 |
| DHRS2 | 1.11 | 2.28E-08 |
| DHRS3 | -1.564 | 5.22E-137 |
| DHTKD1 | 1.05 | 4.59E-90 |
| DIAPH3 | 1.44 | 8.59E-129 |
| DIO2 | 1.334 | 1.34E-32 |
| DIO3OS | -3.572 | 4.60E-275 |
| DIRAS3 | -1.118 | 1.14E-19 |
| DLC1 | -1.99 | 1.64E-150 |
| DLG3 | 1.429 | 8.23E-148 |
| DLGAP5 | 2.551 | 1.56E-216 |
| DLK1 | -1.622 | 9.06E-50 |
| DLK2 | -1.582 | 4.07E-86 |
| DLL1 | -1.864 | 1.29E-158 |
| DLL4 | -1.262 | 6.56E-125 |
| DMD | -2.414 | 1.38E-185 |
| DMGDH | -1.115 | 7.77E-188 |
| DMPK | -1.247 | 1.03E-50 |
| DMRT2 | -1.865 | 6.59E-228 |
| DMTF1 | -1.019 | 1.30E-78 |
| DMTN | -1.044 | 9.43E-60 |
| DNAAF3 | 1.479 | 1.07E-67 |
| DNAH1 | -1.51 | 2.34E-190 |
| DNAH14 | 1.263 | 8.83E-148 |
| DNAJA1 | 1.033 | 3.14E-104 |
| DNAJC1 | 1.122 | 2.43E-83 |
| DNAJC12 | 1.827 | 4.22E-39 |
| DNAJC19P9 | 1.215 | 4.76E-67 |
| DNAJC22 | 1.662 | 4.14E-67 |
| DNALI1 | 1.373 | 4.90E-10 |
| DNASE1L3 | -2.173 | 1.05E-201 |
| DNASE2 | 1.159 | 6.01E-187 |
| DNHD1 | -1.632 | 8.63E-147 |
| DNM1 | -1.245 | 3.55E-88 |
| DOC2B | -1.97 | 3.23E-135 |
| DOCK11 | -1.708 | 7.94E-152 |
| DOCK6 | -1.449 | 1.33E-163 |
| DOK7 | 1.21 | 2.55E-42 |
| DOPEY2 | 1.648 | 3.58E-115 |
| DPCD | 1.096 | 3.32E-123 |
| DPP3 | 1.433 | 2.33E-185 |
| DPP4 | -1.092 | 2.85E-52 |
| DPT | -3.41 | 1.11E-174 |
| DPY19L2 | -2.306 | 4.77E-218 |
| DPYD | -1.032 | 8.85E-67 |
| DPYSL2 | -1.238 | 4.91E-106 |
| DRAIC | 1.024 | 1.16E-25 |
| DRD2 | -1.133 | 2.53E-53 |
| DSC3 | -1.129 | 6.09E-09 |
| DSG3 | -1.244 | 2.10E-08 |
| DSN1 | 1.051 | 5.63E-121 |
| DSP | 1.834 | 5.30E-75 |
| DST | -2.04 | 3.84E-165 |
| DTL | 2.661 | 2.62E-215 |
| DTX1 | -2.136 | 1.99E-206 |
| DTX3L | 1.219 | 1.54E-92 |
| DUSP1 | -2.629 | 1.58E-150 |
| DUSP6 | -1.031 | 4.99E-45 |
| DYNLT1 | 1.032 | 3.05E-145 |
| DZIP1L | -1.342 | 2.32E-150 |
| E2F1 | 2.048 | 1.12E-171 |
| E2F2 | 1.229 | 1.92E-118 |
| E2F5 | 1.146 | 1.25E-89 |
| E2F8 | 1.347 | 2.37E-116 |
| EBF1 | -2.584 | 8.20E-288 |
| EBF2 | -1.942 | 9.28E-259 |
| EBF3 | -2.421 | 9.47E-295 |
| EBP | 1.101 | 7.13E-137 |
| ECE2 | 1.391 | 3.25E-158 |
| ECHDC2 | -1.7 | 1.48E-143 |
| ECHDC3 | -1.03 | 7.97E-91 |
| ECM2 | -1.68 | 2.09E-104 |
| ECT2 | 1.844 | 1.33E-167 |
| EDN1 | -1.762 | 3.18E-90 |
| EDN2 | 1.251 | 1.28E-36 |
| EDN3 | -1.451 | 3.01E-53 |
| EDNRB | -2.707 | 7.00E-278 |
| EEF1A1P12 | 1.698 | 4.27E-53 |
| EEF1A1P9 | 1.234 | 3.59E-45 |
| EEF1A2 | 3.071 | 6.15E-57 |
| EEPD1 | -1.551 | 3.22E-177 |
| EFCAB1 | -1.596 | 1.34E-82 |
| EFCAB11 | 1.149 | 1.38E-88 |
| EFCAB13 | -1.1 | 3.16E-115 |
| EFEMP1 | -2.009 | 2.13E-84 |
| EFHD1 | 1.054 | 3.61E-16 |
| EFNA3 | 1.52 | 7.09E-97 |
| EFNA4 | 1.308 | 5.81E-100 |
| EFNB1 | -1.316 | 1.86E-142 |
| EGFL7 | -2.531 | 3.71E-131 |
| EGFL8 | -1.778 | 1.80E-129 |
| EGFLAM | -2.285 | 2.49E-237 |
| EGFR | -2.695 | 3.19E-161 |
| EGLN3 | 1.078 | 7.79E-34 |
| EGR1 | -1.64 | 1.30E-45 |
| EGR3 | -1.08 | 2.17E-27 |
| EHBP1L1 | -1.033 | 3.40E-65 |
| EHD2 | -1.848 | 3.83E-158 |
| EIF1AXP1 | 1.495 | 5.97E-45 |
| EIF2AK1 | 1.377 | 3.23E-189 |
| EIF5AP4 | 2.915 | 2.40E-174 |
| ELANE | -1.274 | 2.33E-250 |
| ELF5 | -2.163 | 1.62E-15 |
| ELL3 | 1.301 | 1.64E-84 |
| ELMOD3 | -1.363 | 9.87E-186 |
| ELN | -1.441 | 6.10E-31 |
| ELOVL2 | 1.335 | 1.97E-18 |
| ELOVL6 | 1.138 | 1.86E-26 |
| EMCN | -1.94 | 4.30E-148 |
| EME1 | 1.03 | 5.97E-85 |
| EMILIN2 | -1.193 | 8.38E-86 |
| EML3 | -1.473 | 2.58E-145 |
| EMP1 | -1.406 | 5.90E-85 |
| EMX2 | -1.081 | 5.88E-112 |
| EMX2OS | -1.374 | 2.60E-203 |
| ENAH | 1.434 | 1.15E-60 |
| ENC1 | 1.302 | 6.07E-82 |
| ENG | -1.013 | 3.36E-89 |
| ENGASE | -1.779 | 1.10E-106 |
| ENPP2 | -2.924 | 1.16E-203 |
| ENPP5 | 1.428 | 7.72E-48 |
| ENSA | 1.082 | 2.85E-141 |
| TNMD | -3.323 | 7.75E-290 |
| FGR | -1.302 | 4.67E-83 |
| KLHL13 | -1.524 | 6.56E-59 |
| TFPI | -2.5 | 6.61E-173 |
| RBM5 | -1.254 | 1.06E-114 |
| FKBP4 | 1.78 | 7.88E-152 |
| RBM6 | -1.114 | 5.38E-119 |
| HSPB6 | -5.315 | 2.67E-305 |
| PDK4 | -4.513 | 1.80E-242 |
| ZMYND10 | 2.412 | 3.71E-84 |
| MEOX1 | -3.005 | 6.18E-202 |
| PRKAR2B | -1.188 | 2.65E-55 |
| SOX8 | -1.132 | 5.75E-23 |
| ITGAL | 1.022 | 5.91E-39 |
| TMEM132A | 2.211 | 1.43E-155 |
| TAC1 | -1.173 | 2.36E-68 |
| TNFRSF12A | 1.338 | 4.41E-64 |
| GGCT | 1.326 | 4.33E-127 |
| IFRD1 | -1.168 | 9.88E-90 |
| GTF2IRD1 | 1.096 | 6.05E-139 |
| PROM1 | -1.788 | 2.63E-03 |
| SLC13A2 | -1.365 | 1.56E-22 |
| SCN4A | -2.489 | 2.84E-297 |
| FMO3 | -1.298 | 9.30E-53 |
| PKD1 | -1.509 | 9.51E-114 |
| MAPK8IP2 | 1.606 | 5.37E-75 |
| REV3L | -1.227 | 3.46E-167 |
| MLXIPL | -3.86 | 1.45E-288 |
| ETV7 | 1.184 | 3.34E-73 |
| IFFO1 | -1.696 | 4.71E-129 |
| GIPR | -1.411 | 8.22E-73 |
| SEMA3G | -2.891 | 3.65E-222 |
| NISCH | -1.142 | 5.81E-121 |
| STAB1 | -1.724 | 4.65E-121 |
| FYN | -1.469 | 3.21E-114 |
| NME2 | 1.456 | 2.18E-88 |
| SYT7 | 1.127 | 3.20E-52 |
| PLAUR | 1.432 | 4.05E-88 |
| GPRC5A | 3.181 | 4.48E-131 |
| MAMLD1 | -1.199 | 7.36E-92 |
| TACC3 | 1.919 | 5.99E-192 |
| RGPD5 | -1.142 | 5.20E-97 |
| IGF1 | -2.741 | 8.67E-168 |
| VSIG2 | -1.802 | 8.32E-29 |
| PHLDB1 | -1.698 | 2.84E-191 |
| MARCO | -1.697 | 4.21E-31 |
| SYT13 | 1.828 | 5.61E-55 |
| SAMD4A | -1.558 | 3.51E-160 |
| FHL1 | -4.442 | 4.19E-281 |
| NLRP2 | 1.193 | 4.84E-11 |
| HSD17B6 | 1.823 | 4.68E-176 |
| NR1H3 | -1.208 | 1.68E-132 |
| VIM | -1.539 | 3.65E-126 |
| RTEL1-TNFRSF6B | -1.537 | 6.38E-82 |
| RNASET2 | 1.168 | 3.30E-120 |
| TNFRSF1B | -1.404 | 1.18E-93 |
| SLC39A9 | 1.284 | 1.91E-90 |
| IBSP | 1.036 | 2.88E-72 |
| HMGB3 | 2.47 | 1.11E-186 |
| FUT8 | 1.414 | 2.03E-59 |
| MYOC | -2.492 | 9.91E-247 |
| MYOM2 | -1.624 | 5.35E-99 |
| TUBG2 | -1.012 | 2.04E-86 |
| RPL26L1 | 1.181 | 1.96E-206 |
| FLT4 | -1.878 | 3.44E-220 |
| TLL1 | -1.064 | 1.59E-91 |
| VCAN | 1.501 | 1.50E-43 |
| MSR1 | 1.36 | 1.48E-56 |
| RAB27B | 1.561 | 1.83E-51 |
| FAM65C | -2.49 | 2.33E-119 |
| GPM6B | -1.687 | 1.25E-70 |
| PREX2 | -1.792 | 1.37E-231 |
| SCML1 | -1.551 | 9.98E-112 |
| LMO3 | -1.911 | 5.36E-90 |
| EPN3 | 2.103 | 2.47E-145 |
| FOXP3 | 1.312 | 1.96E-116 |
| SLC4A8 | 1.092 | 6.33E-32 |
| LAMC3 | -1.125 | 3.13E-46 |
| PTGER3 | -1.375 | 3.86E-49 |
| RAD51 | 2.231 | 1.06E-207 |
| THOC3 | 1.369 | 9.62E-151 |
| PRSS8 | 2.236 | 4.12E-155 |
| FOXN3 | -1.18 | 1.26E-99 |
| NNAT | -2.873 | 4.96E-201 |
| NRIP2 | -1.012 | 1.19E-123 |
| LAMA3 | -1.876 | 1.55E-63 |
| OPN3 | 1.317 | 9.29E-75 |
| FOXC1 | -1.593 | 2.01E-31 |
| MCOLN3 | -1.597 | 6.95E-87 |
| ITIH4 | -1.242 | 3.95E-76 |
| GYG2 | -2.568 | 6.57E-128 |
| F7 | 1.11 | 5.03E-42 |
| TMCC3 | -1.054 | 6.37E-83 |
| NDC1 | 1.159 | 1.82E-91 |
| SLC2A3 | -1.538 | 4.55E-98 |
| YBX3 | -1.255 | 1.25E-90 |
| HDAC7 | -1.054 | 6.96E-98 |
| MRPS35 | 1.093 | 5.85E-120 |
| SLC6A16 | -1.228 | 3.80E-151 |
| SPA17 | 1.298 | 9.59E-76 |
| WISP2 | -2.363 | 5.76E-73 |
| NGFR | -2.581 | 6.83E-117 |
| SNCAIP | -1.016 | 1.59E-57 |
| MCM10 | 1.313 | 1.26E-76 |
| ERBB3 | 1.601 | 1.19E-121 |
| ROPN1 | -1.633 | 3.14E-17 |
| MYLK | -2.036 | 7.87E-82 |
| TMEM206 | 1.237 | 3.53E-163 |
| GSTO2 | 1.034 | 1.72E-44 |
| TLE2 | -1.18 | 5.75E-74 |
| TIE1 | -1.895 | 8.92E-182 |
| MPPED2 | -1.069 | 1.18E-35 |
| KIF26A | -1.292 | 1.88E-160 |
| KLF6 | -1.437 | 1.04E-108 |
| PPAP2A | -1.874 | 1.21E-216 |
| PKM | 1.151 | 1.60E-141 |
| TRO | -1.419 | 3.39E-103 |
| NAV3 | -1.018 | 2.88E-27 |
| ROGDI | 1.353 | 5.02E-112 |
| PDZD4 | -1.33 | 1.60E-74 |
| FGFR3 | 1.261 | 3.15E-26 |
| PRR11 | 1.992 | 2.39E-129 |
| RASGRP2 | -2.429 | 1.40E-151 |
| PYGM | -1.797 | 3.40E-261 |
| PITX1 | 3.49 | 3.25E-173 |
| MAOB | -1.382 | 5.48E-28 |
| TGFBR3 | -3.053 | 7.71E-171 |
| NUCB2 | 1.304 | 1.53E-81 |
| PFN2 | 1.068 | 3.83E-37 |
| FGF10 | -2.627 | 2.76E-59 |
| FSTL3 | -1.369 | 1.24E-64 |
| ST6GALNAC1 | -1.101 | 5.06E-47 |
| ST6GALNAC2 | 1.006 | 5.74E-22 |
| PTPN21 | -1.197 | 3.45E-101 |
| RPS6KA2 | -1.227 | 9.45E-109 |
| TRIP13 | 2.073 | 1.82E-168 |
| LIMS2 | -2.682 | 5.29E-278 |
| SPEG | -1.271 | 1.92E-49 |
| SMC1A | 1.046 | 1.56E-36 |
| HMMR | 2.542 | 1.98E-220 |
| SIDT1 | 1.052 | 3.87E-21 |
| MRVI1 | -1.439 | 9.92E-137 |
| MCM2 | 1.964 | 2.69E-185 |
| TP63 | -1.971 | 4.83E-38 |
| LLGL2 | 1.281 | 1.93E-124 |
| PDE8A | -1.046 | 4.39E-112 |
| GSDMB | -1.454 | 2.27E-50 |
| FERMT2 | -1.681 | 5.22E-157 |
| PTGS2 | -1.024 | 4.66E-35 |
| IGF2BP2 | -1.773 | 4.45E-81 |
| FRY | -1.05 | 1.06E-69 |
| MRPS34 | 1.333 | 3.20E-172 |
| PPP2R2C | 1.843 | 1.03E-55 |
| MGLL | -1.836 | 6.03E-108 |
| SCARF1 | -1.859 | 7.00E-208 |
| HACD3 | 1.817 | 7.40E-114 |
| TUBE1 | -1.042 | 3.60E-128 |
| GTSE1 | 1.866 | 8.65E-162 |
| TTC38 | -1.027 | 3.43E-122 |
| PLD1 | -1.202 | 9.72E-69 |
| WDR62 | 1.362 | 2.96E-146 |
| SEC31B | -2.156 | 4.17E-182 |
| RBMS2 | -1.008 | 3.31E-113 |
| SPAG5 | 2.03 | 6.57E-158 |
| TPD52 | 2.702 | 3.28E-187 |
| MCAM | -2.196 | 1.46E-193 |
| UBE2T | 3.297 | 5.62E-302 |
| PPP1R12B | -1.652 | 7.90E-159 |
| PAK3 | -1.397 | 8.52E-167 |
| FAM76B | -1.081 | 1.74E-82 |
| GPR137B | 1.089 | 1.08E-57 |
| NAALAD2 | -1.626 | 0.00E+00 |
| JADE1 | -1.072 | 3.66E-122 |
| FGFR1 | -1.645 | 2.30E-77 |
| FBLN1 | -1.455 | 4.51E-62 |
| LAMP3 | 1.241 | 2.72E-51 |
| FAP | 1.315 | 5.55E-51 |
| HOXA9 | -2.124 | 2.77E-120 |
| ITM2A | -2.551 | 2.59E-159 |
| RUNX1T1 | -1.728 | 5.61E-231 |
| SLC1A3 | -1.04 | 2.75E-59 |
| TNS1 | -2.402 | 2.99E-214 |
| RAPGEF3 | -3.41 | 3.15E-261 |
| LIPE | -4.652 | 3.63E-211 |
| PAFAH1B3 | 2.649 | 0.00E+00 |
| KIF22 | 1.229 | 1.52E-171 |
| EPB41L2 | -1.644 | 4.97E-143 |
| HSP90AA1 | 1.016 | 3.02E-60 |
| NDC80 | 2.446 | 2.87E-208 |
| MEF2C | -1.201 | 3.44E-90 |
| PKP1 | -2.156 | 1.04E-50 |
| PCDHB4 | -1.079 | 3.66E-70 |
| PCDHGA2 | -1.009 | 3.89E-55 |
| SMARCD3 | -1.247 | 7.99E-75 |
| FAM135A | -1.058 | 6.95E-92 |
| EPB41L3 | -1.081 | 2.08E-41 |
| KCNK2 | -1.374 | 1.58E-21 |
| SERTAD4 | 1.325 | 1.93E-54 |
| GRHL2 | 1.516 | 6.95E-133 |
| ZMPSTE24 | 1.116 | 4.55E-99 |
| KIAA1467 | 1.381 | 3.48E-39 |
| NKAIN1 | 3.051 | 9.76E-90 |
| RAB10 | 1.133 | 1.73E-89 |
| MAPRE3 | -1.085 | 3.67E-87 |
| MECOM | -1.41 | 1.71E-119 |
| IGSF9 | 1.731 | 1.94E-134 |
| WNT11 | -2.639 | 2.51E-120 |
| TTC39A | 2.337 | 4.32E-92 |
| ORC1 | 1.148 | 8.17E-106 |
| RAD54L | 1.62 | 6.34E-148 |
| EPDR1 | -1.067 | 1.13E-65 |
| FAT2 | -1.13 | 3.02E-33 |
| TMED2 | 1.302 | 3.35E-126 |
| HSD17B2 | -1.585 | 1.95E-26 |
| PPEF1 | 1.006 | 5.21E-81 |
| PPP1R15A | -1.406 | 1.80E-123 |
| ERGIC2 | 1.104 | 1.90E-90 |
| SULT2B1 | 1.049 | 1.20E-29 |
| REM1 | -1.481 | 1.66E-174 |
| TPX2 | 3.526 | 7.52E-274 |
| TESC | -1.868 | 2.43E-129 |
| OAS1 | 1.335 | 1.07E-61 |
| FXYD3 | 1.837 | 5.78E-124 |
| LTBP4 | -2.027 | 2.36E-200 |
| IRAK3 | -1.953 | 4.43E-142 |
| LYZ | 1.898 | 1.27E-51 |
| P3H2 | -1.402 | 2.06E-71 |
| KIF4A | 2.754 | 9.29E-239 |
| NAT14 | 1.28 | 4.16E-123 |
| PITPNM2 | -1.256 | 2.54E-112 |
| ITGA6 | -1.173 | 3.34E-47 |
| TF | -2.943 | 1.94E-99 |
| NLRP1 | -2.111 | 4.57E-179 |
| ORC6 | 1.624 | 8.91E-121 |
| ESR1 | 2.636 | 4.11E-23 |
| SEMA6A | -1.846 | 4.39E-125 |
| WDR76 | 1.199 | 1.72E-121 |
| TBX15 | -2.175 | 1.16E-230 |
| EZR | 2.056 | 4.49E-209 |
| XXbac-B461K10.4 | -1.31 | 1.56E-124 |
| GABRP | -3.269 | 5.99E-18 |
| FMO2 | -3.845 | 1.98E-237 |
| SUCO | 1.25 | 2.28E-93 |
| MSH2 | 1.044 | 5.98E-83 |
| HOOK2 | -1.076 | 1.47E-80 |
| SH2D3C | -1.305 | 2.20E-121 |
| NANS | 1.192 | 1.34E-148 |
| SORBS1 | -2.689 | 2.05E-199 |
| TREM2 | 1.721 | 2.17E-108 |
| FKBP5 | -1.475 | 2.12E-54 |
| TMEM14A | 1.035 | 6.39E-124 |
| PCSK5 | -1.362 | 1.77E-135 |
| ERMP1 | 1.591 | 4.76E-108 |
| PALMD | -2.664 | 2.31E-229 |
| KCNK6 | 1.387 | 1.03E-73 |
| MISP | 3.4 | 6.42E-140 |
| IZUMO4 | -1.052 | 6.54E-63 |
| GADD45B | -1.116 | 1.12E-59 |
| PALM | -1.648 | 1.38E-99 |
| LZTR1 | -1.014 | 6.06E-71 |
| MMP11 | 5.567 | 7.49E-299 |
| SNRPD3 | 1.026 | 7.76E-147 |
| PPM1F | -1.07 | 2.12E-123 |
| MFNG | -1.199 | 3.22E-86 |
| LRP5L | -1.008 | 3.83E-70 |
| SOX10 | -3.799 | 1.32E-48 |
| TTC28 | -1.239 | 3.22E-124 |
| 3-Sep | 1.109 | 8.87E-47 |
| TPTEP1 | -2.218 | 4.40E-63 |
| KDELR3 | 1.518 | 4.52E-82 |
| XBP1 | 1.779 | 1.01E-52 |
| TIMP3 | -1.315 | 3.44E-54 |
| SUN2 | -1.107 | 1.59E-110 |
| TTLL12 | 1.011 | 1.30E-76 |
| FOXRED2 | 1.214 | 9.86E-102 |
| POLE2 | 1.178 | 3.90E-130 |
| GNPNAT1 | 1.332 | 2.07E-94 |
| PLEK2 | 1.709 | 7.37E-166 |
| SIX4 | 1.542 | 1.86E-108 |
| GALNT16 | -1.22 | 1.07E-43 |
| ERH | 1.046 | 5.63E-171 |
| SRSF5 | -1.203 | 2.56E-125 |
| GSKIP | 1.133 | 3.26E-89 |
| PAPLN | -1.395 | 2.27E-98 |
| PSME2 | 1.082 | 7.24E-128 |
| REC8 | -1.119 | 4.56E-24 |
| PLTP | -1.368 | 5.37E-93 |
| MMP9 | 3.199 | 6.75E-110 |
| PROCR | -1.159 | 3.39E-100 |
| GINS1 | 2.091 | 6.07E-205 |
| SGK2 | -2.095 | 9.51E-240 |
| MYBL2 | 3.535 | 3.16E-199 |
| SLMO2 | 1.114 | 3.42E-85 |
| SLCO4A1 | -3.048 | 4.25E-176 |
| PTK6 | 1.728 | 1.28E-96 |
| ISM1 | -2.131 | 3.04E-115 |
| TRIB3 | 1.628 | 3.10E-133 |
| SLC52A3 | 1.32 | 2.13E-79 |
| HM13 | 1.088 | 1.99E-204 |
| SEC23B | 1.343 | 2.58E-128 |
| MYL9 | -1.449 | 2.70E-103 |
| TLDC2 | -1.317 | 1.38E-166 |
| JAG1 | -1.043 | 7.98E-75 |
| PXMP4 | 1.161 | 1.88E-34 |
| PPP1R16B | -1.572 | 9.67E-107 |
| FAM83D | 2.382 | 2.97E-153 |
| SYNDIG1 | 1.454 | 2.02E-80 |
| PIGU | 1.057 | 6.30E-168 |
| TNNC2 | -1.212 | 5.85E-91 |
| MYOM1 | -1.752 | 5.03E-245 |
| RNF125 | -1.083 | 1.15E-112 |
| MXRA5 | 2.181 | 3.04E-105 |
| GPR143 | 1.243 | 8.60E-79 |
| MID1 | -1.655 | 3.06E-79 |
| PRPS2 | 1.266 | 8.43E-99 |
| SRPX | -3.215 | 1.33E-271 |
| SLC35A2 | 1.126 | 1.28E-202 |
| TAZ | -1.241 | 9.46E-77 |
| SMS | 1.398 | 1.11E-177 |
| VGLL1 | -1.531 | 3.94E-04 |
| TIMP1 | 1.043 | 8.34E-72 |
| GABRE | -4.253 | 1.73E-259 |
| MAGED2 | 1.665 | 2.29E-74 |
| KLF8 | -1.54 | 5.75E-134 |
| TNFSF13B | 1.029 | 1.12E-51 |
| SGCG | -1.105 | 8.85E-63 |
| SLC25A15 | 1.098 | 1.70E-76 |
| RGCC | -1.704 | 5.92E-158 |
| MEDAG | -2.87 | 1.31E-203 |
| OLFM4 | -1.595 | 1.41E-16 |
| HSF4 | -1.708 | 9.63E-75 |
| NFAT5 | -1.007 | 7.56E-63 |
| ZNF423 | -1.105 | 5.26E-79 |
| NDRG4 | -1.053 | 4.02E-55 |
| HAS3 | -2.263 | 5.29E-98 |
| SLC7A6 | -1.297 | 8.86E-107 |
| TAF1C | -1.918 | 5.65E-154 |
| HAGHL | 1.275 | 2.00E-61 |
| SLC7A5 | 1.742 | 3.13E-83 |
| METRN | 2.165 | 2.37E-82 |
| FBXO31 | -1.365 | 5.73E-212 |
| GSPT1 | 1.136 | 5.04E-103 |
| TOX3 | 1.448 | 7.19E-39 |
| QPRT | 1.229 | 3.36E-43 |
| PYCARD | 1.529 | 4.07E-78 |
| MAZ | 1.611 | 8.87E-270 |
| IL21R | 1.099 | 3.65E-93 |
| SYT17 | 1.101 | 2.49E-23 |
| TMC5 | 1.28 | 5.96E-17 |
| KNOP1 | 1.376 | 1.33E-183 |
| RASL12 | -1.43 | 1.02E-116 |
| OIP5 | 1.858 | 1.15E-199 |
| PDGFRL | -1.246 | 4.90E-52 |
| SFRP1 | -3.642 | 9.73E-77 |
| LAPTM4B | 1.285 | 1.23E-61 |
| ESRP1 | 2.138 | 4.73E-220 |
| WISP1 | 2.226 | 2.12E-144 |
| TRPS1 | 1.194 | 1.18E-47 |
| SPAG1 | 1.043 | 6.37E-83 |
| NCALD | -1.583 | 1.63E-52 |
| TSTA3 | 1.396 | 2.31E-156 |
| PYCRL | 1.324 | 1.51E-137 |
| SQLE | 2.287 | 5.47E-177 |
| GSR | 1.208 | 3.29E-68 |
| ERICH1 | -1.004 | 2.16E-101 |
| MCM4 | 2.031 | 2.35E-151 |
| SLC17A7 | -1.533 | 0.00E+00 |
| RNASEH2A | 1.678 | 9.62E-244 |
| IL4I1 | 1.824 | 3.15E-119 |
| NOVA2 | -1.313 | 1.50E-212 |
| TNNT1 | 3.311 | 3.63E-67 |
| SYDE1 | -1.25 | 5.14E-120 |
| TJP3 | 1.752 | 9.24E-122 |
| PTPRS | -1.156 | 3.24E-86 |
| LIG1 | 1.007 | 3.26E-112 |
| RAB3D | 1.732 | 2.23E-127 |
| FAM83E | 1.017 | 2.22E-21 |
| RASIP1 | -2.032 | 1.31E-198 |
| PLEKHA4 | -1.122 | 1.25E-38 |
| LILRB5 | -2.139 | 8.34E-184 |
| PIK3R2 | 1.557 | 5.31E-195 |
| HAMP | 1.153 | 4.93E-37 |
| LSR | 1.689 | 2.59E-188 |
| HPN | 2.161 | 1.48E-60 |
| SIPA1L3 | 1.451 | 2.02E-98 |
| RASA4 | -1.207 | 5.52E-98 |
| TFPI2 | -1.381 | 1.51E-17 |
| PON3 | -1.137 | 1.84E-10 |
| PTN | -2.094 | 6.73E-31 |
| MPP6 | -1.015 | 6.08E-43 |
| H2AFV | 1.294 | 4.59E-245 |
| MET | -1.355 | 4.65E-65 |
| WNT2 | 1.237 | 8.02E-46 |
| HOXA2 | -1.245 | 4.61E-221 |
| HOXA3 | -2.039 | 1.71E-230 |
| HOXA5 | -2.381 | 1.80E-228 |
| HOXA6 | -1.493 | 4.83E-180 |
| NOD1 | -1.322 | 5.54E-194 |
| EPHB6 | -1.627 | 1.82E-71 |
| NSUN5P2 | -1.058 | 1.04E-33 |
| PTPRZ1 | -1.653 | 2.04E-49 |
| RPA3 | 1.123 | 6.53E-158 |
| TMEM106B | 1.038 | 1.31E-59 |
| EZH2 | 1.581 | 8.85E-154 |
| MEST | -1.123 | 6.24E-46 |
| MEOX2 | -2.917 | 1.96E-270 |
| TSPAN13 | 1.707 | 1.85E-84 |
| RARRES2 | -1.882 | 1.56E-98 |
| MYL7 | -1.347 | 2.01E-39 |
| PRUNE2 | -1.217 | 3.45E-78 |
| TRIM14 | 1.257 | 1.51E-99 |
| OGN | -2.953 | 1.27E-100 |
| TLE4 | -1.359 | 2.21E-85 |
| LHX6 | -1.669 | 3.21E-159 |
| PLGRKT | 1.076 | 3.94E-87 |
| KANK1 | -1.526 | 6.85E-153 |
| MPDZ | -1.134 | 3.90E-105 |
| PIP5K1B | -1.333 | 6.75E-52 |
| PTGDS | -2.162 | 2.11E-69 |
| ZFAND5 | -1.036 | 3.93E-124 |
| PDLIM1 | -1.093 | 3.97E-57 |
| GATA3 | 2.399 | 8.08E-63 |
| RASSF4 | -1.13 | 4.03E-69 |
| UNC5B | 1.945 | 2.40E-153 |
| NEURL1 | 1.271 | 1.04E-50 |
| MAP3K8 | -1.434 | 1.31E-104 |
| UBE2S | 2.089 | 5.86E-208 |
| 4-Sep | -1.464 | 1.19E-129 |
| PIGL | -1.625 | 5.12E-160 |
| RASD1 | -3.048 | 2.09E-149 |
| ICAM2 | -1.784 | 4.37E-183 |
| SYNGR2 | 1.172 | 3.62E-138 |
| KAT2A | -1.203 | 1.13E-56 |
| EZH1 | -1.861 | 3.94E-223 |
| SGCA | -1.187 | 9.60E-127 |
| LRRC59 | 1.28 | 1.26E-130 |
| HLF | -2.422 | 7.02E-168 |
| SLC16A6 | 1.615 | 1.16E-50 |
| MMD | -1.453 | 7.78E-93 |
| WSB1 | -1.664 | 2.10E-139 |
| SLC9A3R1 | 2.24 | 2.48E-134 |
| MYH3 | -1.041 | 4.34E-190 |
| TMEM97 | 1.725 | 9.77E-125 |
| TMEM33 | 1.249 | 5.48E-89 |
| KIAA1211 | 1.099 | 3.00E-75 |
| MAPK10 | -1.245 | 2.42E-61 |
| TBC1D9 | 1.497 | 1.78E-19 |
| GALNT7 | 1.411 | 2.24E-56 |
| SOD3 | -3.59 | 5.06E-216 |
| NEIL3 | 1.222 | 4.87E-140 |
| SH3D19 | -1.681 | 1.84E-168 |
| FAM149A | -1.506 | 1.01E-162 |
| NCAPG | 2.394 | 3.24E-201 |
| UGDH | 1.3 | 6.18E-40 |
| PPARGC1A | -1.029 | 3.46E-74 |
| ZBTB16 | -3.955 | 5.20E-272 |
| MTCH2 | 1.068 | 1.73E-157 |
| FNBP4 | -1.138 | 8.83E-93 |
| HSPA8 | 1.223 | 2.14E-78 |
| FOLR1 | -2.168 | 2.23E-12 |
| MDK | 1.21 | 4.17E-68 |
| VWF | -2.358 | 1.16E-200 |
| SELPLG | 1.169 | 1.49E-55 |
| KRT18 | 2.199 | 1.34E-150 |
| TNS2 | -2.639 | 3.06E-250 |
| PPM1H | 1.308 | 4.08E-62 |
| MAGOHB | 1.156 | 5.45E-167 |
| FOXM1 | 2.902 | 2.19E-207 |
| PRR4 | -1.092 | 3.01E-31 |
| RAD51AP1 | 1.844 | 6.49E-148 |
| OAS3 | 1.467 | 1.91E-77 |
| OAS2 | 1.588 | 2.63E-72 |
| SLC38A1 | 1.63 | 2.43E-60 |
| RERGL | -3.338 | 5.93E-231 |
| TIMELESS | 1.297 | 5.80E-144 |
| GAPDH | 1.138 | 2.88E-132 |
| LDHB | -1.431 | 2.77E-50 |
| ST8SIA1 | -1.002 | 9.65E-15 |
| NEDD9 | -1.711 | 3.06E-117 |
| FAM184A | -1.217 | 4.19E-97 |
| NCOA7 | -1.003 | 3.61E-44 |
| SASH1 | -1.589 | 5.03E-122 |
| UST | -1.043 | 1.67E-62 |
| SOD2 | -1.146 | 4.94E-49 |
| FHL5 | -2.409 | 1.02E-282 |
| SOBP | -1.262 | 2.53E-179 |
| SLC39A7 | 1.023 | 3.00E-124 |
| PTK7 | 1.026 | 1.22E-60 |
| TTK | 2.047 | 1.68E-164 |
| LAMA4 | -1.329 | 1.04E-88 |
| LY86 | 1.388 | 9.19E-94 |
| TBX18 | -1.614 | 6.87E-167 |
| GHR | -2.312 | 1.15E-153 |
| KIF20A | 3.032 | 5.41E-245 |
| MRPS30 | 1.064 | 1.14E-53 |
| HBEGF | -1.416 | 2.65E-108 |
| GZMK | 1.188 | 1.95E-36 |
| SPARC | 1.062 | 3.21E-28 |
| PDE8B | -1.803 | 8.25E-92 |
| RASGRF2 | -1.216 | 8.68E-90 |
| LMNB1 | 2.847 | 6.98E-233 |
| SLC27A6 | -1.1 | 1.12E-35 |
| IRX4 | -1.367 | 6.63E-23 |
| PRLR | 2.067 | 1.56E-82 |
| LIFR | -2.718 | 4.32E-244 |
| H2AFY | 1.079 | 3.63E-180 |
| ERGIC1 | 1.019 | 2.02E-59 |
| PDGFRB | -1.04 | 1.14E-51 |
| SMC4 | 1.394 | 1.01E-114 |
| NPHP3 | -1.723 | 8.05E-207 |
| PCCB | 1.203 | 1.30E-127 |
| KAT2B | -1.128 | 6.56E-101 |
| HYAL1 | -1.941 | 1.42E-215 |
| GBE1 | -1.166 | 4.14E-102 |
| ROPN1B | -2.142 | 1.72E-23 |
| PODXL2 | 1.637 | 1.12E-88 |
| MRPL3 | 1.008 | 5.26E-155 |
| PLSCR4 | -2.177 | 7.49E-194 |
| VIPR1 | -1.401 | 4.61E-92 |
| NKTR | -1.682 | 1.96E-128 |
| TFCP2L1 | -1.094 | 5.03E-18 |
| FNDC4 | -1.716 | 1.03E-157 |
| PSMD14 | 1.021 | 9.63E-165 |
| PDE1A | -1.003 | 1.83E-88 |
| REEP6 | 1.273 | 4.86E-16 |
| SPTBN1 | -1.648 | 2.52E-218 |
| TACR1 | -2.044 | 1.45E-204 |
| FN1 | 2.999 | 2.16E-183 |
| STAT1 | 1.434 | 1.89E-79 |
| PDCL3 | 1.1 | 5.21E-198 |
| HSPE1 | 1.134 | 1.52E-141 |
| IL1R1 | -1.545 | 1.90E-91 |
| WNT6 | -1.295 | 2.19E-52 |
| IL1RL1 | -1.256 | 6.96E-120 |
| IL18R1 | -1.074 | 5.73E-114 |
| MLPH | 1.723 | 3.45E-15 |
| TPO | -2.259 | 3.95E-299 |
| SDC1 | 2.687 | 5.63E-176 |
| SLC1A4 | 1.784 | 8.12E-88 |
| RND3 | -1.347 | 3.36E-61 |
| EPAS1 | -2.281 | 4.57E-221 |
| MSH6 | 1.044 | 1.14E-77 |
| EPHA4 | -1.218 | 5.80E-86 |
| MARK1 | -1.186 | 2.04E-61 |
| RALGPS2 | 1.344 | 2.83E-48 |
| ERRFI1 | -1.677 | 6.10E-108 |
| KIAA1324 | 1.539 | 1.17E-62 |
| RHOU | -1.547 | 1.97E-98 |
| LAMTOR2 | 1.367 | 9.07E-183 |
| FBXO6 | 1.053 | 2.34E-123 |
| LEPR | -2.5 | 1.02E-208 |
| PRG4 | -1.779 | 2.14E-72 |
| PLA2G4A | -1.351 | 3.53E-72 |
| GADD45A | -1.229 | 2.66E-80 |
| RGS2 | -1.907 | 9.08E-74 |
| SRSF11 | -1.106 | 3.93E-81 |
| NR5A2 | -1.111 | 7.48E-131 |
| TMEM9 | 1.265 | 3.53E-168 |
| NID1 | -1.189 | 8.83E-63 |
| KMO | 1.443 | 3.31E-60 |
| MFAP2 | 1.678 | 2.63E-71 |
| KDM5B | 1.182 | 3.10E-97 |
| RGS4 | 1.536 | 7.64E-71 |
| SSX2IP | 1.869 | 2.14E-148 |
| RBBP5 | 1.101 | 1.60E-96 |
| KIF17 | -1.287 | 9.23E-216 |
| GALE | 1.27 | 1.49E-120 |
| ID3 | -1.227 | 1.48E-75 |
| SLC2A1 | 1.118 | 3.66E-67 |
| PTCH2 | -1.118 | 2.00E-126 |
| PRDX1 | 1.158 | 2.72E-149 |
| TSPAN1 | 2.725 | 1.67E-59 |
| F3 | -2.662 | 2.95E-161 |
| PTBP2 | -1.102 | 1.25E-95 |
| TNFSF4 | 1.215 | 1.48E-105 |
| HSD11B1 | -1.49 | 5.65E-90 |
| IRF6 | 1.058 | 5.49E-76 |
| RSRP1 | -1.317 | 1.83E-96 |
| STMN1 | 1.957 | 3.33E-169 |
| NEK2 | 3.743 | 5.38E-303 |
| PROX1 | -1.006 | 5.25E-106 |
| MUC5B | 1.103 | 9.43E-23 |
| KIF14 | 1.264 | 4.99E-121 |
| MREG | 1.419 | 1.78E-73 |
| LRMP | -1.203 | 1.96E-85 |
| PLAGL1 | -2.257 | 5.68E-184 |
| MYB | 1.801 | 2.50E-53 |
| SGK1 | -1.29 | 1.74E-83 |
| SLC16A7 | -1.448 | 3.22E-171 |
| VAMP8 | 1.376 | 4.21E-216 |
| RPN2 | 1.295 | 1.77E-179 |
| PKD2 | -1.363 | 1.96E-113 |
| SPP1 | 2.409 | 2.03E-79 |
| RARRES1 | -1.452 | 6.16E-17 |
| PPL | -1.577 | 1.11E-102 |
| KLF9 | -2.132 | 1.22E-209 |
| WDR34 | 1.295 | 5.66E-163 |
| SET | 1.24 | 2.10E-105 |
| PPP2R4 | 1.339 | 6.42E-170 |
| NR4A3 | -1.307 | 3.61E-70 |
| GPR68 | 1.149 | 6.10E-76 |
| RHOQ | -1.16 | 1.07E-124 |
| KLHL29 | -2.208 | 4.84e-316 |
| EPCAM | 2.871 | 1.23E-200 |
| GPAM | -3.186 | 8.12E-173 |
| PYROXD2 | -1.289 | 3.68E-107 |
| HELLS | 1.446 | 1.11E-139 |
| KCNIP2 | -4.446 | 9.53E-261 |
| HOXB3 | -1.275 | 7.83E-25 |
| TEK | -1.566 | 5.42E-139 |
| MOB3B | -1.469 | 1.07E-108 |
| PLEKHG1 | -1.243 | 4.76E-90 |
| MYCT1 | -1.836 | 3.29E-203 |
| MSANTD2 | -1.061 | 9.74E-137 |
| PDZD11 | 1.081 | 4.60E-175 |
| IQSEC3 | -1.248 | 1.00E-161 |
| SMAD9 | -1.314 | 9.81E-201 |
| HSPH1 | 1.086 | 1.18E-94 |
| PLS1 | 1.257 | 1.95E-56 |
| SOCS2 | -2.111 | 9.82E-113 |
| TNFRSF8 | -1.174 | 8.14E-180 |
| LYPLA1 | 1.249 | 9.29E-75 |
| TBX2 | -1.688 | 5.68E-107 |
| NCAPH | 2.389 | 3.93E-206 |
| MND1 | 1.933 | 4.93E-161 |
| KCNJ8 | -1.634 | 1.29E-132 |
| PDZRN3 | -1.264 | 5.55E-70 |
| KIF18A | 1.483 | 5.22E-144 |
| PILRB | -1.61 | 1.90E-64 |
| TMEM54 | 1.228 | 7.30E-141 |
| GPSM2 | 1.2 | 3.65E-90 |
| RASL11A | -1.625 | 3.82E-101 |
| XPNPEP2 | -1.305 | 1.18E-174 |
| LDB3 | -1.424 | 3.38E-191 |
| PTGFR | -1.928 | 3.72E-181 |
| FAM126A | -1.687 | 5.23E-143 |
| HOXA7 | -2.061 | 1.58E-147 |
| INHBA | 2.883 | 1.41E-192 |
| RAMP3 | -1.35 | 4.96E-42 |
| TWIST1 | -2.18 | 3.74E-136 |
| RECK | -1.433 | 4.87E-131 |
| PLAU | 1.124 | 5.86E-56 |
| ZWINT | 3.055 | 4.90E-281 |
| NECAB1 | -1.139 | 1.19E-108 |
| NLN | 1.261 | 1.47E-84 |
| ITIH5 | -3.379 | 4.11E-255 |
| MMP19 | -1.424 | 2.55E-100 |
| NR4A1 | -2.801 | 1.14E-116 |
| PDE1B | -1.994 | 3.87E-184 |
| HOXC13 | 2.337 | 7.87E-116 |
| LRP1 | -1.804 | 1.74E-133 |
| HOXC11 | 1.96 | 6.83E-83 |
| TUBA1B | 1.006 | 9.60E-109 |
| STIL | 1.421 | 1.21E-126 |
| HJURP | 2.311 | 3.13E-202 |
| PLP1 | -1.719 | 5.26E-91 |
| TNFAIP6 | 1.077 | 8.16E-29 |
| G0S2 | -4.49 | 2.01E-171 |
| SLC12A4 | -1.526 | 2.86E-185 |
| PI3 | -1.872 | 1.34E-19 |
| SNX21 | -1.134 | 1.26E-126 |
| SLPI | -1.439 | 6.41E-13 |
| PREX1 | 1.001 | 2.03E-18 |
| PARD6B | 1.707 | 3.20E-79 |
| PTGIS | -1.965 | 7.79E-115 |
| PPP4R1L | -1.454 | 3.30E-164 |
| KCNK15 | 1.327 | 3.89E-28 |
| PCK1 | -3.644 | 2.85E-230 |
| NEURL2 | -1.082 | 1.19E-137 |
| XG | -1.05 | 1.80E-43 |
| HIF3A | -3.864 | 0.00E+00 |
| LYPD3 | 1.204 | 7.68E-73 |
| F13A1 | -1.269 | 5.32E-37 |
| MOCS1 | -2.152 | 1.27E-254 |
| HIST1H2BJ | 1.257 | 1.06E-51 |
| SPDEF | 2.158 | 2.93E-50 |
| SOX4 | 1.333 | 9.06E-100 |
| GLO1 | 1.239 | 1.28E-133 |
| SSR1 | 1.166 | 2.73E-75 |
| NRN1 | -2.683 | 4.13E-213 |
| TMEM255A | -1.105 | 3.22E-87 |
| GTF3C4 | 1.008 | 1.29E-56 |
| PPP1R12C | -1.679 | 3.68E-110 |
| MBOAT7 | 1.507 | 3.40E-173 |
| SRMS | 1.169 | 7.10E-87 |
| IL1B | 1.163 | 1.49E-25 |
| TRIP10 | -1.929 | 1.31E-195 |
| FOSB | -1.648 | 2.03E-32 |
| OVOL2 | 1.026 | 2.95E-101 |
| LAMP5 | 2.698 | 2.28E-142 |
| TCF15 | -1.41 | 1.00E-134 |
| FAM110A | 1.257 | 1.80E-132 |
| ID1 | -1.868 | 6.15E-117 |
| F10 | -2.218 | 1.22E-249 |
| THRA | -1.115 | 7.56E-84 |
| NR1D1 | -1.518 | 2.84E-95 |
| RRAS | -1.187 | 7.43E-104 |
| STAT5A | -1.379 | 1.72E-122 |
| IFI6 | 2.562 | 1.96E-100 |
| SIX1 | 1.55 | 1.57E-30 |
| RHOJ | -1.629 | 2.83E-147 |
| L3HYPDH | -1.638 | 7.03E-185 |
| OMD | -1.029 | 1.50E-23 |
| FGD3 | 1.459 | 2.16E-54 |
| MASP1 | -1.295 | 5.50E-210 |
| TSPAN8 | -1.712 | 3.02E-31 |
| PTPRB | -1.829 | 3.38E-150 |
| PLA2G5 | -1.202 | 6.04E-111 |
| SIN3B | -1.38 | 1.02E-112 |
| KLF2 | -1.908 | 3.06E-135 |
| PKMYT1 | 3.415 | 5.75E-270 |
| FBXL16 | 1.863 | 6.46E-69 |
| IL17B | -1.414 | 2.36E-60 |
| GNG11 | -2.206 | 2.56E-242 |
| STYXL1 | 1.21 | 5.67E-163 |
| STEAP4 | -2.7 | 3.76E-89 |
| GNAI1 | -2.135 | 2.53E-182 |
| SGCE | -1.204 | 8.47E-49 |
| ZFP36 | -2.428 | 2.13E-142 |
| SRD5A3 | 1.361 | 7.73E-127 |
| RASL11B | 1.361 | 7.07E-82 |
| PAICS | 1.506 | 1.76E-146 |
| KDR | -1.196 | 5.44E-97 |
| PPAT | 1.202 | 9.22E-101 |
| TUBGCP6 | -1.266 | 2.01E-111 |
| POM121L9P | -1.203 | 1.34E-176 |
| MGAT3 | -1.465 | 7.17E-113 |
| RAC2 | 1.051 | 6.70E-54 |
| RIBC2 | 1.447 | 6.46E-133 |
| KRT17 | -3.196 | 3.75E-18 |
| RNF112 | -1.09 | 1.04E-189 |
| FOXP2 | -1.195 | 8.38E-170 |
| IFT22 | 1.141 | 4.02E-127 |
| FLNC | -1.27 | 1.70E-91 |
| HOXD3 | -1.364 | 1.81E-178 |
| HOXD9 | -1.264 | 2.03E-107 |
| KNSTRN | 1.18 | 1.16E-119 |
| LOXL1 | 2 | 1.03E-129 |
| SOX15 | -1.189 | 3.91E-39 |
| FAM64A | 2.166 | 5.23E-172 |
| TXNDC17 | 1.278 | 5.38E-205 |
| KLK10 | -1.905 | 1.40E-08 |
| KLK8 | -2.407 | 8.81E-21 |
| FOXA1 | 2.934 | 1.04E-58 |
| RNASE1 | -1.004 | 2.96E-42 |
| SEC14L1 | -1.01 | 1.65E-147 |
| MAP7D3 | -1.865 | 3.52E-195 |
| SGOL1 | 1.802 | 2.17E-163 |
| SHC2 | -1.502 | 1.06E-60 |
| GAMT | 1.061 | 2.27E-14 |
| PRRG3 | -1.239 | 4.10E-204 |
| STARD8 | -1.396 | 3.66E-182 |
| PVRL2 | 1.31 | 2.50E-123 |
| PLVAP | -1.128 | 3.61E-74 |
| USHBP1 | -2.343 | 3.31E-303 |
| KIAA1683 | -1.372 | 1.25E-122 |
| LSM4 | 1.24 | 6.79E-215 |
| HRC | -1.468 | 1.25E-189 |
| OLFM1 | -1.094 | 1.41E-37 |
| TNNT3 | -2.874 | 3.40E-196 |
| PNPLA7 | -1.713 | 3.48E-147 |
| PAK4 | 1.184 | 3.48E-113 |
| RBBP8NL | 1.169 | 7.47E-105 |
| LAMA5 | -1.471 | 1.61E-42 |
| SMPDL3B | 1.527 | 2.11E-114 |
| SLC7A10 | -2.933 | 5.50E-208 |
| HABP4 | -1.262 | 2.33E-177 |
| RGN | -1.792 | 1.48E-95 |
| SYNE1 | -1.363 | 1.87E-125 |
| GFAP | -1.701 | 3.29E-182 |
| GINS2 | 1.971 | 1.05E-173 |
| F12 | 2.07 | 3.07E-139 |
| IDO1 | 1.135 | 3.46E-29 |
| PPT1 | 1.333 | 7.57E-108 |
| RAB11FIP4 | 1.692 | 1.80E-111 |
| SH3BP5 | -1.097 | 7.59E-98 |
| GALNT15 | -3.786 | 3.20E-289 |
| NAPSB | 1.49 | 1.41E-63 |
| PDLIM4 | -1.515 | 6.49E-51 |
| GFPT2 | -1.119 | 1.26E-49 |
| TUBG1 | 1.067 | 7.81E-122 |
| RAMP2 | -1.415 | 1.43E-90 |
| NINJ1 | 1.028 | 3.82E-78 |
| MAP1B | -1.384 | 2.40E-83 |
| TNS4 | -1.878 | 4.67E-16 |
| TOP2A | 3.907 | 1.78E-275 |
| PPP1R1B | -1.506 | 1.02E-16 |
| RAI2 | -1.658 | 7.93E-71 |
| FAAP24 | 1.01 | 1.90E-146 |
| MATN3 | 1.578 | 2.84E-62 |
| PPARG | -3.343 | 3.48E-253 |
| NUP210 | 2.144 | 1.60E-213 |
| RAMP1 | 1.46 | 1.24E-48 |
| RAN | 1.115 | 8.50E-156 |
| PRKAA1 | -1.002 | 3.58E-59 |
| SERPINF1 | -1.549 | 2.76E-94 |
| PNISR | -1.493 | 1.49E-140 |
| MATN2 | -2.613 | 1.21E-138 |
| HSPA12B | -1.928 | 4.78E-189 |
| PCNA | 1.711 | 1.34E-209 |
| NES | -1.025 | 2.24E-46 |
| RAB25 | 2.201 | 4.63E-196 |
| MTL5 | 1.836 | 1.46E-121 |
| LPIN3 | -1.441 | 5.89E-109 |
| WASF3 | -2.009 | 1.80E-213 |
| LGR6 | -1.905 | 2.22E-34 |
| SPG20 | -1.092 | 2.37E-83 |
| EPSTI1 | 1.014 | 1.33E-38 |
| POSTN | 2.61 | 8.58E-99 |
| KL | -1.344 | 2.29E-228 |
| SLC39A11 | 1.564 | 2.18E-125 |
| LGALS12 | -3.803 | 6.60E-177 |
| RARRES3 | 1.47 | 5.14E-50 |
| HRASLS2 | 1.166 | 1.23E-31 |
| MYH11 | -3.557 | 1.85E-168 |
| PDZD2 | -1.379 | 5.86E-104 |
| GIMAP6 | -1.365 | 4.33E-93 |
| ZNF767P | -1.103 | 1.03E-92 |
| TMEM254 | 1.342 | 2.67E-118 |
| TMTC1 | -2.291 | 3.25E-140 |
| LRRCC1 | 1.073 | 1.48E-65 |
| LYVE1 | -4.43 | 0.00E+00 |
| MICAL2 | 1.548 | 6.42E-97 |
| TTC9 | 1.323 | 9.10E-86 |
| MEIS2 | -1.777 | 2.56E-155 |
| GSTM5 | -2.638 | 3.86E-153 |
| VAV3 | 1.883 | 1.30E-61 |
| PSRC1 | 1.228 | 3.63E-121 |
| HMGCS2 | -1.44 | 2.24E-03 |
| WNT2B | -1.028 | 3.62E-155 |
| NGF | -1.046 | 6.62E-113 |
| TMEM106C | 1.012 | 1.88E-86 |
| RSAD2 | 1.373 | 7.13E-52 |
| LPIN1 | -1.568 | 8.98E-134 |
| SAA2 | -3.643 | 2.83E-112 |
| TIMM17A | 1.09 | 1.30E-159 |
| GRP | 2.341 | 1.79E-36 |
| IL15RA | -1.228 | 7.02E-91 |
| TMEM241 | 1.275 | 2.46E-108 |
| SOX5 | -1.16 | 3.35E-107 |
| SPX | -1.712 | 7.70E-122 |
| PIWIL4 | -1.004 | 1.76E-108 |
| HOOK1 | 1.073 | 2.82E-74 |
| FHOD3 | -1.005 | 2.21E-35 |
| FADS2 | 1.671 | 2.93E-35 |
| TCN1 | -1.022 | 2.47E-02 |
| PDGFRA | -1.683 | 1.60E-94 |
| KLB | -1.437 | 1.72E-153 |
| NREP | 1.653 | 2.83E-134 |
| GOLM1 | 1.79 | 6.98E-96 |
| FAM189A2 | -2.653 | 7.62E-124 |
| SDS | 2.196 | 7.05E-146 |
| OASL | 1.613 | 2.36E-73 |
| RNFT2 | 1.276 | 4.94E-124 |
| NT5E | -1.044 | 1.31E-51 |
| LMO2 | -1.232 | 2.08E-131 |
| PRRG4 | 1.337 | 1.22E-92 |
| ITGA7 | -4.456 | 0.00E+00 |
| RDH5 | -3.612 | 1.54E-286 |
| PPP1R1A | -4.071 | 8.48E-172 |
| TROAP | 2.749 | 3.07E-208 |
| PAN2 | -1.657 | 8.39E-137 |
| ESPL1 | 1.546 | 1.31E-141 |
| SLC26A10 | -2.014 | 6.97E-191 |
| MAP7 | 1.437 | 1.75E-111 |
| HEY2 | -1.023 | 1.38E-30 |
| PKIB | 2.315 | 1.96E-90 |
| STX11 | -1.616 | 6.50E-159 |
| FBXL8 | -1.06 | 2.48E-57 |
| KCNK1 | 1.785 | 2.81E-45 |
| NTPCR | 1.046 | 4.77E-117 |
| KIAA1614 | -1.255 | 4.15E-164 |
| LAMC1 | -1.085 | 6.61E-65 |
| SLC19A3 | -4.077 | 2.15E-223 |
| THSD1 | -1.326 | 8.28E-187 |
| SPRY2 | -3.037 | 1.48E-306 |
| RCBTB2 | -1.01 | 1.75E-103 |
| LCP1 | 1.138 | 2.25E-39 |
| IL6 | -2.407 | 1.87E-95 |
| NACAD | -1.233 | 3.60E-154 |
| MRPL47 | 1.011 | 9.72E-179 |
| SCN7A | -1.331 | 6.22E-132 |
| EPRS | 1.303 | 1.96E-204 |
| HLX | -1.093 | 6.57E-91 |
| IL1RN | 1.286 | 1.32E-41 |
| UGGT1 | 1.051 | 1.06E-99 |
| GYPC | -2.082 | 1.23E-200 |
| KLF4 | -2.489 | 3.51E-177 |
| TMOD1 | -2.398 | 1.43E-225 |
| STXBP1 | -1.392 | 3.31E-130 |
| TLR4 | -1.281 | 3.52E-88 |
| LMX1B | 1.17 | 2.67E-47 |
| MYC | -1.036 | 1.54E-47 |
| NOV | -1.159 | 2.86E-47 |
| IL33 | -2.798 | 2.99E-136 |
| IL11RA | -1.936 | 6.84E-225 |
| PPIL1 | 1.203 | 3.55E-148 |
| PIM1 | -1.02 | 3.37E-54 |
| TMEM63B | 1.151 | 2.20E-131 |
| TUBB2B | -1.099 | 9.18E-17 |
| UQCC2 | 1.458 | 1.09E-194 |
| HMGA1 | 1.509 | 3.90E-135 |
| IER3 | 1.618 | 3.43E-74 |
| TPMT | 1.239 | 4.21E-140 |
| FGFBP2 | -2.237 | 1.06E-140 |
| FHDC1 | 1.13 | 1.31E-90 |
| LRRC32 | -1.278 | 5.87E-68 |
| PI15 | -2.648 | 1.76E-40 |
| GGH | 1.385 | 2.33E-60 |
| SULF1 | 1.908 | 4.87E-103 |
| TRPC6 | -1.268 | 2.92E-160 |
| TRIM29 | -2.001 | 7.83E-17 |
| FXYD6 | -1.29 | 5.81E-73 |
| MMP13 | 2.718 | 6.85E-112 |
| MAPKBP1 | -1.069 | 6.82E-113 |
| NUSAP1 | 3.209 | 2.88E-260 |
| KIF23 | 2.206 | 2.78E-191 |
| ITGA11 | 1.122 | 5.62E-33 |
| STRA6 | 1.117 | 2.74E-51 |
| SEMA6D | -1.595 | 3.74E-101 |
| GIPC2 | -1.537 | 4.22E-270 |
| EPT1 | 1.097 | 1.54E-90 |
| LOXL4 | -1.794 | 1.13E-114 |
| KIF11 | 2.594 | 7.27E-232 |
| RBP4 | -5.514 | 2.32E-199 |
| OLA1 | 1.395 | 3.85E-134 |
| SENP7 | -1.006 | 6.65E-74 |
| PARP9 | 1.157 | 2.92E-80 |
| FAM13A | -2.207 | 4.08E-240 |
| FGF2 | -2.469 | 9.18E-199 |
| MMRN1 | -3.147 | 4.42E-194 |
| PDE5A | -1.26 | 5.10E-57 |
| PRDM5 | -1.195 | 6.40E-158 |
| LEF1 | 1.462 | 1.31E-83 |
| MAPK8IP3 | -1.585 | 3.74E-60 |
| ERP27 | 1.881 | 1.16E-60 |
| GABARAPL1 | -1.899 | 4.01E-236 |
| FGD4 | -1.288 | 2.02E-139 |
| PIK3C2G | -1.829 | 1.34E-64 |
| LRIG3 | -1.794 | 2.55E-137 |
| TMEM19 | 1.195 | 3.88E-63 |
| LUM | 1.035 | 3.90E-22 |
| SDSL | 1.444 | 2.88E-126 |
| N4BP2L1 | -1.459 | 1.69E-141 |
| MAP3K12 | -1.145 | 3.48E-62 |
| GALNT6 | 2.247 | 2.23E-77 |
| LMBR1L | -1.42 | 3.97E-149 |
| TMBIM6 | 1.102 | 8.42E-69 |
| WDFY2 | -1.083 | 4.32E-161 |
| NOVA1 | -2.594 | 7.88E-67 |
| PELI2 | -1.715 | 3.68E-205 |
| FBLN5 | -2.282 | 6.22E-164 |
| SORD | 2.132 | 1.47E-102 |
| SLC27A2 | 1.402 | 3.05E-32 |
| FGF7 | -2.223 | 1.51E-134 |
| HDC | -1.029 | 4.96E-29 |
| NEIL1 | -1.373 | 3.64E-70 |
| MAN2C1 | -2.593 | 1.10E-187 |
| PCSK6 | 1.303 | 3.97E-30 |
| FANCI | 1.712 | 8.19E-162 |
| NTRK3 | -2.111 | 7.02E-106 |
| MFGE8 | -1.938 | 1.02E-103 |
| MCTP2 | -1.135 | 1.82E-49 |
| TGFB1I1 | -1.337 | 5.17E-107 |
| IGSF6 | 1.119 | 3.99E-58 |
| MARVELD3 | 1.201 | 1.83E-103 |
| KIFC3 | -1.197 | 2.04E-85 |
| NUDT7 | -1.111 | 2.37E-112 |
| KSR1 | -1.484 | 1.06E-107 |
| TOM1L1 | 1.34 | 2.09E-67 |
| SKAP1 | 1.525 | 9.42E-54 |
| LRRC46 | 1.274 | 1.19E-68 |
| SCRN2 | -1.374 | 1.82E-114 |
| GNAL | -1.289 | 2.05E-121 |
| SLC39A6 | 2.364 | 1.03E-60 |
| GALNT1 | 1.04 | 8.06E-59 |
| SLC16A3 | 1.05 | 9.83E-52 |
| PMAIP1 | 1.763 | 2.13E-93 |
| P3H4 | 1.47 | 1.39E-144 |
| ERBB2 | 1.211 | 5.52E-61 |
| STAC2 | -4.352 | 1.85E-60 |
| TPGS1 | -1.375 | 4.72E-22 |
| PPAP2C | 1.35 | 6.84E-95 |
| TMEM91 | -1.129 | 3.74E-67 |
| SIK1 | -1.756 | 1.71E-44 |
| TRPM2 | 1.448 | 1.86E-76 |
| EPHA2 | -1.5 | 2.46E-134 |
| PLK4 | 1.452 | 1.05E-133 |
| MAP3K6 | -1.702 | 2.31E-115 |
| FCN3 | -1.361 | 6.28E-125 |
| HSPG2 | -1.074 | 2.80E-68 |
| TINAGL1 | -2.855 | 3.46E-212 |
| KIF2C | 2.608 | 6.58E-205 |
| IGSF3 | 1.595 | 1.16E-98 |
| PSMA5 | 1.072 | 8.82E-193 |
| ITGA10 | -1.5 | 9.19E-104 |
| TIPRL | 1.026 | 8.22E-115 |
| POGK | 1.124 | 1.16E-113 |
| RXRG | -1.115 | 8.61E-182 |
| TMCO1 | 1.716 | 1.21E-189 |
| NUF2 | 2.777 | 4.60E-217 |
| SDHC | 1.094 | 1.66E-130 |
| MRPL24 | 1.122 | 2.59E-130 |
| PIGM | 1.292 | 4.99E-135 |
| ISG20L2 | 1.286 | 1.76E-115 |
| HDGF | 1.167 | 4.36E-173 |
| RGS16 | 1.287 | 8.49E-56 |
| RGL1 | -1.367 | 5.62E-117 |
| PRUNE | 1.09 | 2.49E-116 |
| TUFT1 | 1.487 | 4.32E-124 |
| SF3B4 | 1.123 | 3.91E-171 |
| OAZ3 | 1.166 | 4.40E-88 |
| GOLPH3L | 1.184 | 2.03E-78 |
| INTS7 | 1.569 | 9.91E-208 |
| SUSD4 | 1.505 | 1.49E-66 |
| S100A8 | -1.505 | 1.24E-04 |
| TPM3 | 1.374 | 1.30E-202 |
| ILF2 | 1.122 | 2.66E-153 |
| SCCPDH | 1.366 | 5.10E-60 |
| SRP9 | 1.511 | 8.33E-196 |
| NVL | 1.096 | 5.84E-131 |
| MBOAT2 | 1.122 | 2.39E-63 |
| PARP1 | 1.622 | 4.14E-208 |
| ETNK2 | 1.115 | 1.38E-25 |
| OSR1 | -3.157 | 4.51E-143 |
| MEIS1 | -1.275 | 9.58E-137 |
| GPR17 | -1.85 | 5.25E-272 |
| GULP1 | -1.532 | 1.18E-94 |
| KANSL1L | -1.3 | 3.90E-134 |
| HES6 | 1.451 | 1.12E-84 |
| FANCD2 | 1.059 | 2.03E-104 |
| RBMS3 | -2.144 | 1.39E-248 |
| STAC | -1.114 | 7.73E-39 |
| NFKBIZ | -1.861 | 4.63E-60 |
| PHLDB2 | -1.582 | 8.99E-102 |
| SRPRB | 1.162 | 6.69E-147 |
| TRPC1 | -1.329 | 5.15E-167 |
| NCEH1 | 1.088 | 3.28E-53 |
| ILDR1 | 1.668 | 3.55E-131 |
| SLIT2 | -1.011 | 3.71E-54 |
| OCIAD2 | 1.63 | 3.72E-204 |
| SLC10A6 | -1.016 | 3.20E-150 |
| SNCA | -1.273 | 4.85E-106 |
| USP53 | -1.431 | 5.96E-111 |
| GZMA | 1.023 | 1.34E-31 |
| PIK3R1 | -1.429 | 6.65E-70 |
| TSLP | -1.224 | 1.53E-264 |
| RNF145 | -1.023 | 4.20E-55 |
| ZNF300 | -1.009 | 2.07E-59 |
| N4BP3 | 1.137 | 1.24E-72 |
| KCNMB1 | -2.038 | 3.01E-114 |
| GFOD1 | -1.269 | 7.88E-125 |
| KLHL3 | -1.198 | 3.36E-170 |
| FAM193B | -1.626 | 1.48E-121 |
| NFKBIE | 1.051 | 1.07E-125 |
| PM20D2 | -1.465 | 1.51E-55 |
| RNF217 | -1.471 | 1.13E-130 |
| RSPO3 | -2.255 | 5.67E-225 |
| MTFR2 | 1.291 | 2.69E-124 |
| SLC22A3 | -1.981 | 3.23E-252 |
| IGFBP3 | -1.314 | 2.04E-85 |
| PSPH | 1.004 | 1.55E-81 |
| ZNF92 | 1.028 | 1.46E-41 |
| TMEM209 | 1.028 | 3.57E-79 |
| NCAPG2 | 1.224 | 2.01E-99 |
| SHROOM2 | 1.23 | 2.58E-101 |
| RAB19 | 1.053 | 8.82E-80 |
| TMEM47 | -1.505 | 1.79E-83 |
| OGT | -1.238 | 1.65E-97 |
| GPC3 | -3.302 | 3.29E-162 |
| GNRH1 | -1.26 | 6.74E-136 |
| SLC25A37 | -1.288 | 3.72E-85 |
| TACC1 | -2.252 | 5.87E-154 |
| GINS4 | 1.236 | 2.48E-85 |
| MTDH | 1.099 | 3.31E-37 |
| MAL2 | 2.771 | 6.40E-193 |
| SLC39A4 | 1.464 | 7.21E-77 |
| NFIB | -1.467 | 3.11E-71 |
| PLIN2 | -2.307 | 2.32E-160 |
| NTRK2 | -2.709 | 2.05E-112 |
| ZNF462 | -1.102 | 6.82E-49 |
| GSN | -2.361 | 2.01E-257 |
| HMCN2 | -1.361 | 1.47E-121 |
| FAM171A1 | -1.089 | 6.58E-32 |
| ZEB1 | -1.136 | 1.01E-62 |
| FAM13C | -1.992 | 1.40E-235 |
| TCF7L2 | -1.302 | 5.82E-117 |
| MKI67 | 3.089 | 8.71E-233 |
| RGS10 | 1.253 | 7.57E-138 |
| SAA4 | -1.585 | 2.26E-119 |
| SYT8 | -1.941 | 7.80E-23 |
| PAMR1 | -2.633 | 4.98E-173 |
| GLYAT | -2.519 | 1.06E-186 |
| SERPING1 | -1.589 | 4.40E-128 |
| KLHL35 | 1.154 | 9.03E-66 |
| NCAM1 | -1.35 | 1.18E-79 |
| P4HA3 | 1.641 | 8.51E-109 |
| ST14 | 1.427 | 6.88E-131 |
| PLCH2 | -1.834 | 2.66E-56 |
| FEZ1 | -1.38 | 1.22E-130 |
| ESAM | -1.747 | 2.99E-172 |
| SIDT2 | -1.071 | 1.07E-123 |
| TAGLN | -1.614 | 5.42E-92 |
| JPH2 | -1.937 | 4.32E-146 |
| PPP4C | 1.179 | 8.60E-198 |
| FCGR1A | 2.252 | 4.09E-175 |
| LATS2 | -1.013 | 3.71E-93 |
| LYPD6B | 1.182 | 5.31E-28 |
| FSIP1 | 1.874 | 4.82E-50 |
| FOXO1 | -1.789 | 8.34E-206 |
| TWF1 | 1.078 | 2.06E-57 |
| KCTD14 | -1.523 | 2.31E-32 |
| THRSP | -3.145 | 1.02E-80 |
| ME3 | -1.79 | 2.71E-132 |
| EPS8 | -1.4 | 6.76E-111 |
| NR3C2 | -1.645 | 6.25E-143 |
| INPP1 | -1.479 | 1.57E-175 |
| FLI1 | -1.155 | 1.24E-94 |
| ZNF385D | -1.427 | 7.07E-57 |
| TDO2 | 1.461 | 2.98E-101 |
| GFRA1 | 1.265 | 8.88E-04 |
| SCHIP1 | -1.208 | 5.16E-63 |
| HSPB8 | -1.177 | 3.27E-17 |
| GEMIN6 | 1.161 | 7.62E-207 |
| TMEM178A | -1.728 | 3.54E-161 |
| SPC25 | 2.327 | 3.26E-239 |
| PDE3B | -2.104 | 1.51E-136 |
| TCF7L1 | -1.58 | 1.64E-85 |
| UHMK1 | 1.149 | 7.97E-70 |
| NMT2 | -1.496 | 5.87E-161 |
| ZFP36L2 | -1.442 | 2.19E-109 |
| PLEKHH2 | -2.258 | 1.11E-217 |
| GRIA4 | -1.208 | 1.25E-84 |
| IGSF10 | -2.771 | 3.11E-244 |
| SPARCL1 | -2.123 | 9.09E-147 |
| PRDM8 | -1.316 | 7.89E-225 |
| GGPS1 | 1.238 | 2.54E-140 |
| ZNF117 | -1.027 | 9.56E-64 |
| RAB3C | -1.464 | 2.10E-109 |
| MARVELD2 | 1.95 | 1.70E-126 |
| MERTK | -1.263 | 2.00E-125 |
| PLA2R1 | -1.135 | 3.33E-84 |
| RBMS1 | -1.173 | 7.33E-97 |
| SLC25A27 | -3.228 | 7.02E-275 |
| FAM49B | 1.107 | 1.26E-102 |
| LINC00467 | 1.012 | 2.07E-98 |
| KCNJ16 | -1.212 | 7.68E-116 |
| PID1 | -2.745 | 6.75E-268 |
| LGI4 | -3.497 | 0.00E+00 |
| MSI2 | 1.24 | 4.42E-83 |
| SEMA3D | -1.082 | 6.72E-65 |
| THY1 | 1.371 | 3.10E-64 |
| ROBO4 | -2.265 | 1.94E-250 |
| ROBO3 | -2.609 | 4.97E-262 |
| PGM5 | -2.624 | 1.61E-217 |
| LONRF1 | -1.203 | 3.77E-115 |
| GBP5 | 1.295 | 1.10E-48 |
| PDLIM3 | -1.419 | 1.59E-42 |
| SORBS2 | -1.125 | 1.44E-34 |
| L3MBTL4 | -1.428 | 2.24E-84 |
| PDE1C | -1.726 | 2.72E-146 |
| JAM2 | -2.215 | 3.22E-198 |
| FGD5 | -1.371 | 1.51E-85 |
| PLCL2 | -1.006 | 2.09E-77 |
| SKA1 | 1.722 | 2.82E-161 |
| EPHB1 | -1.199 | 4.48E-57 |
| VOPP1 | 1.203 | 2.67E-117 |
| GRAMD3 | -1.347 | 1.54E-66 |
| PPM1J | 1.272 | 1.57E-62 |
| VSIG4 | -1.106 | 3.28E-41 |
| PDIA4 | 1.386 | 7.11E-173 |
| MICU3 | -1.93 | 1.91e-317 |
| WIF1 | -2.544 | 2.66E-84 |
| NDUFAF6 | 1.16 | 2.09E-121 |
| TSPAN7 | -2.846 | 1.76E-214 |
| RPGR | -1.303 | 4.51E-142 |
| SFXN2 | 1.321 | 1.06E-68 |
| SH3RF2 | -1.041 | 3.51E-53 |
| RAB11FIP1 | 1.534 | 1.02E-43 |
| MAPK13 | 1.251 | 1.78E-132 |
| NMNAT2 | -1.592 | 1.15E-107 |
| RBPMS | -1.505 | 1.01E-124 |
| TIMP4 | -4.84 | 1.75E-264 |
| SYN2 | -2.861 | 4.66E-306 |
| NRG1 | -1.252 | 9.67E-32 |
| SUSD3 | 2.351 | 7.16E-52 |
| IL34 | -1.65 | 1.29E-33 |
| KIT | -2.663 | 1.75E-78 |
| TSC22D3 | -1.218 | 1.01E-78 |
| ERG | -1.794 | 3.30E-183 |
| ETS2 | -1.594 | 1.10E-163 |
| SLC34A2 | -1.921 | 2.36E-09 |
| FMNL2 | -1.377 | 1.31E-96 |
| FAM213B | 1.017 | 3.23E-91 |
| TNFRSF14 | -1.347 | 2.46E-83 |
| RADIL | -1.077 | 1.41E-53 |
| MRPL17 | 1.293 | 1.64E-238 |
| MRAS | -1.849 | 2.40E-134 |
| SHROOM4 | -1.151 | 8.25E-134 |
| HIST1H2BD | 2.501 | 4.13E-117 |
| HIST1H4H | 2.267 | 1.63E-88 |
| KCNB1 | -1.272 | 1.76E-154 |
| TSPAN33 | 1.01 | 4.97E-65 |
| NRG2 | -2.057 | 4.58E-182 |
| GDPD5 | -1.669 | 1.13E-120 |
| PFKFB1 | -2.015 | 6.99E-174 |
| SLAMF8 | 1.275 | 6.39E-66 |
| F11R | 1.438 | 4.83E-120 |
| ZNF276 | -1.097 | 3.52E-83 |
| FAM160B2 | -1.311 | 6.50E-99 |
| MPZ | -1.755 | 2.80E-119 |
| MIS18A | 1.185 | 1.93E-160 |
| LAD1 | 1.752 | 9.55E-60 |
| RCAN1 | -1.205 | 5.53E-51 |
| IRX6 | -1.782 | 8.20E-169 |
| STARD9 | -2.322 | 0.00E+00 |
| NPR2 | -1.705 | 2.90E-193 |
| KALRN | -1.334 | 2.05E-99 |
| TFF3 | 2.733 | 4.63E-25 |
| TFF1 | 5.892 | 1.98E-53 |
| RSPH1 | 1.895 | 1.97E-88 |
| SLC37A1 | 1.094 | 6.17E-121 |
| PDE9A | -1.456 | 1.50E-75 |
| U2AF1 | -1.269 | 4.41E-22 |
| ITGB2 | 1.033 | 1.43E-42 |
| S100B | -3.96 | 8.86E-156 |
| ZNF208 | -1.142 | 7.14E-57 |
| FCN2 | -1.07 | 1.53E-54 |
| ST6GALNAC6 | -1.268 | 3.78E-167 |
| ZDHHC12 | 1.136 | 2.23E-157 |
| PKN3 | -1.282 | 3.90E-132 |
| PPAPDC3 | -1.322 | 5.43E-170 |
| TLCD1 | 2.168 | 2.98E-197 |
| RUSC1 | 1.049 | 7.21E-118 |
| PTH1R | -2.715 | 7.89E-251 |
| MYL3 | -1.533 | 1.09E-149 |
| NACC1 | 1.558 | 4.76E-225 |
| LY6K | -1.193 | 3.43E-05 |
| TONSL | 1.2 | 6.46E-108 |
| RECQL4 | 1.928 | 3.15E-148 |
| ZNF333 | -1.191 | 1.93E-146 |
| SCGB3A1 | -2.317 | 5.27E-26 |
| MPP3 | -1.028 | 1.03E-36 |
| LSM12 | 1.009 | 1.75E-137 |
| RACGAP1 | 2.179 | 3.17E-180 |
| GRASP | -2.907 | 3.70E-263 |
| SPC24 | 2.105 | 1.36E-200 |
| POLR3K | 1.622 | 7.97E-220 |
| SNRNP25 | 1.35 | 1.49E-179 |
| PAQR4 | 2.318 | 1.50E-216 |
| ZG16B | 2.532 | 1.41E-78 |
| TAL1 | -1.841 | 8.09E-294 |
| SLC1A7 | -1.237 | 2.01E-192 |
| PPAP2B | -2.176 | 8.95E-239 |
| KLHL21 | -1.095 | 4.19E-89 |
| RBP7 | -2.866 | 6.53E-183 |
| LAPTM5 | 1.01 | 8.46E-50 |
| RBBP4 | 1.141 | 2.30E-69 |
| MEGF6 | -1.295 | 4.07E-63 |
| NEXN | -1.489 | 5.86E-121 |
| ZNF281 | 1.485 | 4.48E-93 |
| KLHDC9 | 1.265 | 2.50E-47 |
| FLVCR1 | 1.137 | 2.34E-111 |
| SNED1 | -1.369 | 6.27E-95 |
| SPATA17 | 1.667 | 1.75E-129 |
| MT2P1 | -1.183 | 5.50E-101 |
| KIF26B | 1.731 | 7.60E-134 |
| TFB2M | 1.089 | 3.56E-124 |
| PKDCC | -2.774 | 1.25E-171 |
| HAAO | -1.14 | 2.62E-59 |
| PIGR | -3.104 | 1.51E-37 |
| H3F3A | 1.513 | 5.10E-231 |
| NOSTRIN | -1.118 | 5.22E-50 |
| INHBB | -1.528 | 7.95E-77 |
| MSX1 | -1.42 | 1.75E-129 |
| S100A11 | 1.249 | 9.55E-99 |
| S100A9 | -1.432 | 2.70E-04 |
| S100A12 | -1.051 | 7.62E-94 |
| TDRD10 | -2.384 | 2.09e-320 |
| PMVK | 1.086 | 1.66E-100 |
| PYGO2 | 1.162 | 1.19E-183 |
| EOGT | -1.208 | 6.95E-126 |
| FSTL1 | -1.076 | 9.29E-49 |
| LMOD1 | -3.052 | 4.43E-268 |
| TMEM79 | 1.166 | 2.64E-110 |
| SSR2 | 1.02 | 2.46E-163 |
| KIAA1524 | 1.179 | 9.24E-94 |
| TGFBR2 | -2.323 | 2.50E-198 |
| HDAC11 | 1.129 | 1.31E-64 |
| FBLN2 | -1.155 | 3.99E-40 |
| NFASC | -1.206 | 3.18E-24 |
| SGOL2 | 1.271 | 3.49E-132 |
| NUAK2 | 1.388 | 4.22E-147 |
| GMPS | 1.13 | 3.61E-106 |
| PTX3 | -2.76 | 1.40E-131 |
| SMIM14 | 1.516 | 6.77E-56 |
| RBM47 | 1.477 | 4.04E-88 |
| PRRT3 | 1.457 | 4.16E-74 |
| PCOLCE2 | -4.207 | 2.06E-170 |
| TM4SF18 | -1.16 | 3.73E-42 |
| KIF15 | 1.769 | 1.08E-161 |
| KLF15 | -3.119 | 0.00E+00 |
| LIPH | 1.027 | 5.08E-36 |
| RPN1 | 1.117 | 2.04E-199 |
| RPL39L | 1.313 | 7.29E-69 |
| PIGX | 1.201 | 9.05E-129 |
| S100P | 4.113 | 1.11E-103 |
| H2AFZ | 1.371 | 1.92E-192 |
| SPRY1 | -2.461 | 4.27E-192 |
| MST1R | 1.271 | 1.61E-58 |
| POC1A | 1.791 | 2.02E-247 |
| PRSS12 | -1.032 | 2.28E-24 |
| HAND2 | -1.454 | 4.47E-148 |
| MAD2L1 | 2.269 | 4.34E-195 |
| HPGD | -1.119 | 1.53E-16 |
| NPY5R | -1.093 | 2.12E-17 |
| F2RL2 | 1.748 | 1.03E-56 |
| PRRC1 | 1.032 | 5.03E-95 |
| ESM1 | 2.136 | 2.73E-176 |
| FABP7 | -1.307 | 4.10E-06 |
| SFXN1 | 1.18 | 3.59E-82 |
| PI16 | -3.366 | 7.90E-118 |
| KIAA0895 | 1.007 | 5.60E-70 |
| PTTG1 | 3.255 | 2.51E-277 |
| SLC29A4 | -1.71 | 1.41E-79 |
| HEY1 | -1.184 | 5.37E-88 |
| FABP5 | -1.798 | 2.73E-60 |
| FNDC1 | 2.338 | 4.72E-91 |
| SOX17 | -2.847 | 6.05E-292 |
| RAD21 | 1.004 | 8.64E-60 |
| PHKG1 | -1.22 | 3.58E-117 |
| GPR146 | -3.199 | 2.00e-319 |
| GPER1 | -1.418 | 1.05E-56 |
| NOS3 | -1.24 | 7.10E-85 |
| OSR2 | -1.352 | 2.18E-46 |
| YWHAZ | 1.256 | 1.02E-100 |
| FREM1 | -1.775 | 3.88E-135 |
| GEM | -1.301 | 3.86E-48 |
| NIPSNAP3B | -1.876 | 4.22E-294 |
| NFIL3 | -1.813 | 1.78E-133 |
| MAMDC2 | -2.729 | 1.19E-232 |
| RASEF | 1.3 | 3.70E-50 |
| SVEP1 | -2.948 | 1.26E-263 |
| FBP1 | 1.716 | 1.57E-39 |
| TMEM246 | -1.168 | 1.45E-194 |
| FIGF | -3.88 | 0.00E+00 |
| STRBP | 1.591 | 9.26E-193 |
| ZNF367 | 1.192 | 8.76E-118 |
| MELK | 2.783 | 3.16E-211 |
| ZCCHC24 | -1.608 | 2.49E-124 |
| FOLR2 | -1.123 | 1.12E-41 |
| GJB2 | 3.387 | 7.75E-168 |
| HEPACAM | -1.515 | 1.40E-207 |
| SKA3 | 1.956 | 1.94E-188 |
| TMEM63C | 1.745 | 2.93E-66 |
| VSTM4 | -1.15 | 1.16E-89 |
| ZNF503 | -1.032 | 6.91E-49 |
| HPRT1 | 1.204 | 2.92E-141 |
| RET | 2.252 | 1.92E-61 |
| NDRG2 | -2.666 | 7.51E-190 |
| ZNF219 | -1.509 | 3.99E-120 |
| VWA2 | 1.048 | 2.43E-21 |
| HSPA12A | -1.156 | 1.48E-103 |
| PDZRN4 | -1.006 | 2.47E-55 |
| HACD1 | -1.235 | 2.10E-51 |
| JAM3 | -1.265 | 4.65E-95 |
| SPINT1 | 1.07 | 1.52E-123 |
| LARP6 | -2.049 | 1.78E-172 |
| SGPL1 | 1.104 | 4.27E-92 |
| SCN3B | -1.141 | 8.92E-213 |
| PLEKHF1 | -1.209 | 6.15E-70 |
| TMEM100 | -3.437 | 6.70E-284 |
| KIAA0355 | -1.027 | 2.84E-118 |
| TUB | -1.277 | 4.82E-93 |
| RIC3 | -2.525 | 7.34E-126 |
| PLD4 | 1.051 | 3.83E-40 |
| ZMAT1 | -1.073 | 3.37E-65 |
| PKD1L2 | -2.387 | 1.13E-258 |
| LEO1 | 1.14 | 8.77E-94 |
| MFAP4 | -2.845 | 6.59E-127 |
| RIMKLB | -1.372 | 1.12E-155 |
| TMED3 | 1.623 | 1.25E-214 |
| SEC11C | 1.01 | 2.92E-92 |
| RRAD | -2.567 | 1.34E-164 |
| NNMT | -1.243 | 4.65E-66 |
| ZNF667-AS1 | -1.01 | 3.33E-37 |
| YPEL4 | -1.433 | 1.47E-235 |
| KIAA0101 | 3.936 | 0.00E+00 |
| LDHD | -1.275 | 2.66E-77 |
| PLIN1 | -6.025 | 2.57E-195 |
| MESP1 | -1.079 | 5.02E-19 |
| RBPMS2 | -1.614 | 6.22E-137 |
| NAV2 | -1.382 | 1.54E-50 |
| GLYATL1 | 1.126 | 8.36E-28 |
| PLK1 | 2.641 | 2.21E-200 |
| GREM1 | 1.336 | 8.03E-25 |
| EVA1C | -1.378 | 3.17E-96 |
| PDIA3 | 1.383 | 3.98E-197 |
| SNRPD1 | 1.196 | 7.81E-165 |
| FAM102A | 1.278 | 4.26E-113 |
| PRR15L | 1.806 | 5.32E-99 |
| GPRC5B | -1.402 | 1.08E-76 |
| FBXO22 | 1.087 | 8.83E-144 |
| TBC1D2B | -1.069 | 4.35E-105 |
| IGF2 | -1.401 | 1.77E-36 |
| ENTHD2 | -1.276 | 2.52E-58 |
| MYO5B | 1.663 | 3.71E-136 |
| RRM1 | 1.175 | 1.15E-103 |
| RAB8A | 1.218 | 1.19E-125 |
| TUBA1C | 1.799 | 8.82E-188 |
| GPD1 | -5.69 | 1.23E-207 |
| NFKBID | -1.008 | 7.24E-81 |
| TMC4 | 1.242 | 5.17E-75 |
| TTYH1 | -1.842 | 4.28E-21 |
| LENG8 | -2.364 | 1.67E-145 |
| PPP1R14A | -3.652 | 1.32E-282 |
| SPINT2 | 1.918 | 9.12E-180 |
| PLIN4 | -5.465 | 2.65E-203 |
| GPT | -1.781 | 1.39E-75 |
| RILP | -1.462 | 2.39E-164 |
| SERPINF2 | -1.649 | 2.05E-109 |
| KLK4 | 1.201 | 3.82E-30 |
| KLK5 | -2.882 | 1.60E-21 |
| KLK6 | -1.81 | 5.91E-09 |
| KLK11 | -1.649 | 1.10E-06 |
| IGFBP6 | -3.691 | 1.30E-294 |
| HID1 | 1.042 | 1.02E-28 |
| TMEM88 | -2.486 | 1.15E-255 |
| EVPL | 1.489 | 5.81E-147 |
| TK1 | 3.246 | 9.95E-287 |
| RAB26 | 1.765 | 1.38E-92 |
| VWCE | -1.5 | 3.25E-257 |
| RAB3IL1 | -1.107 | 3.26E-94 |
| HRASLS5 | -2.076 | 2.90E-70 |
| NAALADL1 | -1.566 | 1.70E-166 |
| SAC3D1 | 1.208 | 6.45E-148 |
| PBK | 2.854 | 1.77E-226 |
| SCARA5 | -4.23 | 0.00E+00 |
| VASN | -1.053 | 1.97E-49 |
| RNF187 | 1.22 | 1.61E-207 |
| GLYCTK | -1.144 | 1.53E-128 |
| FAM107A | -3.899 | 1.14E-267 |
| MLKL | -1.029 | 5.15E-90 |
| RHOH | 1.131 | 2.61E-44 |
| STIP1 | 1.016 | 1.12E-112 |
| SCNN1B | -1.291 | 1.36E-27 |
| RAB31 | 1.13 | 3.44E-44 |
| TNXB | -4.234 | 3.04E-289 |
| PHYHIP | -2.01 | 8.05E-295 |
| FEN1 | 1.83 | 5.32E-218 |
| SDPR | -4.047 | 0.00E+00 |
| SERINC2 | 1.523 | 1.74E-114 |
| FAM84B | 1.218 | 9.63E-91 |
| LDLRAD4 | 1.36 | 1.24E-44 |
| NPNT | 1.955 | 1.80E-49 |
| SHOX2 | -1.253 | 9.49E-108 |
| VAMP5 | -1.099 | 2.33E-84 |
| ZNF608 | -1.132 | 1.34E-58 |
| SLC35G2 | -1.927 | 9.34E-241 |
| STXBP6 | -1.624 | 5.73E-135 |
| JMJD7-PLA2G4B | -1.386 | 2.52E-140 |
| PXDC1 | -1.494 | 1.78E-119 |
| KLK7 | -1.973 | 1.91E-19 |
| PARM1 | -1.66 | 2.34E-88 |
| RP11-231C14.4 | -1.285 | 9.17E-75 |
| RSPO1 | -1.187 | 6.76E-56 |
| LMAN2 | 1.064 | 4.66E-134 |
| SLC50A1 | 1.827 | 2.57E-233 |
| GPRIN1 | 1.346 | 7.55E-147 |
| KCNAB1 | -1.516 | 4.96E-178 |
| SHE | -2.52 | 6.65E-298 |
| NPR1 | -3.557 | 0.00E+00 |
| MUC15 | -1.367 | 1.23E-14 |
| ZEB2 | -1.639 | 4.61E-136 |
| MT1E | -1.012 | 6.05E-32 |
| LDB2 | -2.369 | 6.10E-261 |
| HNRNPF | 1.022 | 4.98E-97 |
| REPS2 | 1.241 | 6.95E-62 |
| SYAP1 | 1.341 | 1.21E-131 |
| ZFPM2 | -1.059 | 2.57E-74 |
| IFFO2 | -1.086 | 9.49E-30 |
| SIK2 | -1.559 | 5.04E-189 |
| RNF150 | -1.353 | 6.30E-106 |
| HOXD4 | -2.016 | 9.28E-211 |
| MRAP | -4.334 | 1.04E-203 |
| FAXDC2 | -2.322 | 1.32E-117 |
| HSPB2 | -2.363 | 4.35E-251 |
| FABP4 | -6.623 | 8.32E-201 |
| FOS | -1.972 | 2.24E-53 |
| TMED10 | 1.09 | 1.08E-85 |
| OR2A20P | -1.12 | 6.64E-77 |
| LRRN2 | 1.296 | 1.56E-54 |
| KRT8 | 2.054 | 7.94E-159 |
| MZB1 | 1.043 | 9.10E-25 |
| HSD17B13 | -1.732 | 2.20E-291 |
| PFKFB3 | -1.611 | 6.09E-88 |
| IRX1 | -2.654 | 1.76E-81 |
| IRX2 | -1.012 | 1.93E-05 |
| HSPA4 | 1.278 | 6.30E-170 |
| ZNF296 | 1.015 | 2.73E-75 |
| KCNS3 | 1.013 | 4.66E-55 |
| SDR16C5 | 1.729 | 8.66E-34 |
| TPT1-AS1 | -1.631 | 1.07E-168 |
| PRKCDBP | -1.194 | 6.70E-64 |
| PDGFD | -1.55 | 2.96E-85 |
| S1PR1 | -2.736 | 3.38E-229 |
| LRRC8E | 1.669 | 5.20E-154 |
| SOX7 | -1.473 | 7.13E-156 |
| GIMAP8 | -1.735 | 6.99E-185 |
| PROL1 | -1.375 | 6.39E-35 |
| NETO2 | 1.127 | 5.54E-41 |
| TMEM37 | -1.596 | 3.57E-86 |
| LRG1 | 1.783 | 1.78E-27 |
| SHCBP1 | 1.639 | 2.53E-143 |
| SOSTDC1 | -2.019 | 3.61E-31 |
| ESCO2 | 1.654 | 2.04E-129 |
| KRT19 | 1.756 | 2.91E-104 |
| KRT15 | -3 | 7.32E-13 |
| KRT13 | -1.558 | 3.02E-33 |
| NAT1 | 3.542 | 1.21E-66 |
| PTGER4 | -1.193 | 7.13E-79 |
| PLEKHG5 | -1.156 | 1.21E-95 |
| LGALS4 | -1.016 | 1.13E-127 |
| LRRC34 | -1.012 | 9.49E-121 |
| SYCE1 | -1.036 | 4.66E-50 |
| RHNO1 | 1.381 | 1.45E-174 |
| RRM2 | 3.76 | 2.67E-251 |
| ZNF217 | 1.014 | 5.39E-43 |
| SYNPO | -1.322 | 1.26E-105 |
| KLF11 | -1.18 | 1.13E-119 |
| LRRC15 | 3.75 | 3.40E-173 |
| FRMD3 | -1.493 | 2.12E-102 |
| MRPL13 | 1.254 | 8.41E-146 |
| ID4 | -2.841 | 7.12E-130 |
| NEGR1 | -1.035 | 4.33E-41 |
| RCAN2 | -1.129 | 1.44E-98 |
| SYNPO2 | -2.424 | 2.42E-158 |
| PPP1CA | 1.153 | 1.65E-192 |
| MUCL1 | -3.29 | 1.97E-07 |
| SNTG2 | -1.418 | 2.97E-213 |
| RND1 | 1.957 | 1.79E-54 |
| SLFN11 | -1.013 | 6.23E-51 |
| OVOL1 | 1.721 | 3.10E-121 |
| LVRN | -2.457 | 4.82E-273 |
| MYEOV | -2.102 | 2.38E-59 |
| MRGPRF | -2.591 | 1.37E-246 |
| HPSE2 | -2.089 | 2.73E-246 |
| EVC2 | -1.082 | 1.73E-143 |
| RHOD | 1.103 | 1.14E-82 |
| VANGL1 | 1.524 | 2.24E-112 |
| SYT12 | 1.093 | 2.01E-26 |
| SNCG | -2.377 | 1.07E-82 |
| MMRN2 | -2.081 | 1.42E-207 |
| NDNF | -1.16 | 9.06E-53 |
| OLR1 | 2.228 | 6.46E-134 |
| SAA1 | -4.476 | 2.92E-147 |
| PPP1R14B | 1.133 | 8.94E-97 |
| PTPRM | -1.752 | 5.07E-167 |
| TNFRSF10D | -1.466 | 2.89E-158 |
| MST1 | -1.187 | 3.50E-45 |
| HSPB7 | -4.352 | 5.29E-276 |
| STAT5B | -1.198 | 3.01E-125 |
| GPR160 | 1.905 | 1.34E-64 |
| SPTBN2 | 1.085 | 4.02E-50 |
| FBXO45 | 1.296 | 2.52E-129 |
| FAM3C2 | 1.26 | 1.07E-32 |
| MSRB3 | -1.488 | 5.67E-98 |
| RP11-23P13.6 | -1.571 | 7.82E-76 |
| SELP | -1.932 | 5.28E-101 |
| PHLDA3 | -1.063 | 3.61E-73 |
| SLC16A11 | -1.389 | 4.69E-100 |
| PODN | -1.854 | 3.14E-87 |
| EXO1 | 2.364 | 1.06E-195 |
| ZWILCH | 1.071 | 9.21E-112 |
| MFSD4 | -1.003 | 3.66E-41 |
| GOLT1A | 1.529 | 1.27E-97 |
| LEP | -5.101 | 2.87E-213 |
| FZD4 | -2.155 | 1.55E-164 |
| PQLC2L | -2.864 | 4.28E-268 |
| SEZ6L2 | 1.965 | 1.07E-57 |
| UBE2C | 4.527 | 8.93E-301 |
| PDIK1L | 1.118 | 1.07E-88 |
| MARCKSL1 | 1.994 | 2.15E-223 |
| GOLGA8A | -2.985 | 9.32E-167 |
| PHYKPL | -1.428 | 4.68E-152 |
| GRAMD2 | -1.231 | 3.34E-12 |
| SCUBE2 | 1.1 | 5.79E-04 |
| LPL | -4.594 | 3.43E-210 |
| MCTP1 | -1.408 | 1.36E-106 |
| UCP2 | 1.109 | 9.24E-50 |
| SUGCT | 1.072 | 1.28E-58 |
| RMI2 | 2.638 | 4.29E-247 |
| KDF1 | 1.077 | 8.83E-135 |
| NR2F1 | -1.45 | 2.48E-55 |
| PRIMA1 | -1.407 | 2.14E-106 |
| RUVBL1 | 1.074 | 2.08E-150 |
| SFN | 1.253 | 2.02E-64 |
| GAPT | 1.194 | 1.28E-70 |
| HOXD8 | -1.314 | 2.39E-51 |
| PLEKHF2 | 1.542 | 2.68E-85 |
| TUBB6 | -1.228 | 1.61E-91 |
| TPRN | 1.619 | 6.51E-113 |
| RP11-574E24.3 | -1.669 | 2.86E-258 |
| GPX2 | -1.246 | 2.70E-14 |
| SYNE3 | -2.291 | 1.96E-281 |
| LPCAT4 | -1.146 | 3.49E-145 |
| SLCO3A1 | -1.018 | 2.97E-53 |
| PLA2G16 | -1.004 | 5.49E-47 |
| PRR15 | 2.833 | 1.78E-77 |
| LMNB2 | 1.027 | 1.25E-108 |
| TYMS | 2.223 | 1.58E-179 |
| FUT2 | 1.242 | 4.07E-55 |
| SCN4B | -2.22 | 1.57E-175 |
| LINC00982 | -1.171 | 1.96E-207 |
| LRRN4CL | -2.956 | 0.00E+00 |
| HIC1 | -1.377 | 1.66E-105 |
| GPR4 | -1.051 | 3.76E-89 |
| PTRF | -1.805 | 5.09E-164 |
| SAMD12 | 1.022 | 4.98E-61 |
| JUN | -1.747 | 1.54E-92 |
| GBA | 1.185 | 2.26E-154 |
| PNPLA2 | -2.117 | 1.68E-176 |
| PVRL3 | -1.046 | 1.14E-98 |
| SOX12 | 1.635 | 1.40E-166 |
| ZBTB41 | 1.176 | 1.45E-71 |
| UBE2N | 1.088 | 3.07E-110 |
| MLF1 | 1.221 | 1.05E-70 |
| STAP2 | 1.087 | 5.29E-104 |
| ZNF366 | -1.053 | 2.51E-154 |
| RNF212 | -1.505 | 3.97E-39 |
| ZNF354B | -1.272 | 8.76E-134 |
| MAF | -1.265 | 3.32E-127 |
| KCTD12 | -1.421 | 7.91E-109 |
| THBD | -1.12 | 1.18E-70 |
| RIIAD1 | 1.025 | 1.82E-53 |
| TRIM73 | -1.227 | 1.05E-103 |
| EXOSC4 | 1.166 | 1.35E-112 |
| LGALS7B | -1.215 | 2.60E-15 |
| ZNF552 | 1.629 | 5.28E-57 |
| RMI1 | 1.477 | 8.42E-124 |
| RCC2 | 1.286 | 1.19E-189 |
| PER1 | -3.85 | 3.22E-274 |
| SPTY2D1 | 1.055 | 2.91E-52 |
| GIMAP7 | -1.511 | 1.84E-96 |
| TMEM125 | 1.228 | 7.65E-120 |
| MAGED1 | 1.477 | 1.10E-143 |
| FAM156B | -1.36 | 1.15E-107 |
| ZBTB42 | 1.389 | 4.82E-85 |
| PDXDC1 | 1.011 | 5.14E-75 |
| SEPHS2 | 1.388 | 2.52E-148 |
| GPBAR1 | -1.847 | 2.94E-206 |
| ZNF48 | 1.101 | 1.35E-151 |
| LYNX1 | -1.477 | 1.52E-54 |
| FAHD1 | 1.122 | 2.44E-141 |
| RCC1 | 1.103 | 1.69E-115 |
| HERC2P3 | -2.207 | 6.47E-170 |
| ZNRF2 | 1.096 | 6.20E-83 |
| FZD2 | 1.063 | 2.61E-87 |
| MTURN | -1.527 | 2.99E-150 |
| GAS1 | -1.651 | 7.39E-95 |
| HIST1H2AC | 1.903 | 2.64E-89 |
| SRP9P1 | 2.168 | 4.85E-45 |
| HIST1H2BC | 1.936 | 2.65E-70 |
| MAB21L1 | -2.069 | 0.00E+00 |
| SHISA2 | 1.047 | 1.54E-18 |
| HOXC10 | 2.906 | 5.05E-66 |
| GREM2 | -1.062 | 4.74E-99 |
| OXTR | -3.243 | 6.87E-84 |
| FAM83H | 2.02 | 2.89E-223 |
| MRPL14 | 1.336 | 8.46E-207 |
| NQO1 | 1.024 | 6.20E-23 |
| F2R | 1.053 | 2.15E-44 |
| PENK | -1.531 | 4.57E-90 |
| HIST3H2A | 1.58 | 9.76E-41 |
| TMEM132C | -3.167 | 0.00E+00 |
| SYNE4 | 1.327 | 8.06E-117 |
| ZNF467 | 1.099 | 1.47E-48 |
| MRPS23 | 1.067 | 3.04E-125 |
| FDCSP | -1.568 | 1.08E-03 |
| YIPF6 | 1.115 | 7.57E-61 |
| SIAH2 | 1.605 | 9.88E-106 |
| SLC9A9 | -1.053 | 1.75E-102 |
| SLC2A4 | -2.632 | 0.00E+00 |
| SNRPE | 1.183 | 1.62E-180 |
| RTKN2 | 1.121 | 6.22E-127 |
| IDH2 | 1.696 | 2.12E-161 |
| TMEM259 | -1.354 | 1.66E-84 |
| TMEM30B | 1.15 | 1.92E-59 |
| RP11-69E11.4 | -1.694 | 4.66E-124 |
| FAM89A | -2.345 | 9.67E-178 |
| TDRKH | 1.345 | 1.28E-117 |
| RGMA | -2.517 | 4.70E-135 |
| SYNM | -3.554 | 1.66E-146 |
| FIGN | -1.15 | 2.27E-122 |
| TSHZ2 | -1.824 | 3.52E-126 |
| KPNA2 | 2.335 | 3.83E-222 |
| FES | -1.645 | 3.99E-144 |
| MXRA7 | -1.048 | 5.38E-81 |
| SATB1 | -1.267 | 1.15E-71 |
| SKA2 | 1.072 | 4.07E-103 |
| NDN | -1.856 | 3.96E-168 |
| NTM | 1.22 | 1.79E-42 |
| RGS6 | -1.619 | 1.59E-241 |
| HCAR2 | -1.786 | 1.22E-160 |
| TMEM198B | -1.315 | 2.45E-159 |
| PLCXD3 | -1.236 | 7.52E-202 |
| RRP7BP | -1.135 | 1.81E-71 |
| SLC25A18 | -1.841 | 6.36E-132 |
| TCEAL7 | -1.945 | 1.27E-170 |
| ZNF662 | -1.468 | 8.62E-118 |
| PYCR1 | 2.933 | 8.37E-303 |
| SPNS2 | -1.857 | 3.53E-119 |
| SLC25A10 | 1.137 | 3.16E-96 |
| LYSMD4 | -1.005 | 2.07E-127 |
| GAS6 | -1.151 | 6.26E-62 |
| RIPPLY3 | 1.007 | 3.39E-81 |
| RABIF | 1.622 | 5.30E-258 |
| FANCF | 1.274 | 1.39E-165 |
| RUVBL2 | 1.002 | 4.74E-142 |
| PRR36 | 1.719 | 4.75E-103 |
| OVCH2 | -1.076 | 8.98E-128 |
| NPIPA1 | -1.276 | 3.56E-101 |
| RP11-958N24.1 | -1.603 | 2.55E-87 |
| PI4KAP2 | -1.87 | 2.07E-145 |
| FAM46C | 1.365 | 3.64E-46 |
| PCBP3 | -1.17 | 6.68E-80 |
| TRMT12 | 1.005 | 9.76E-98 |
| UPP1 | -1.196 | 1.04E-49 |
| LHFP | -2.559 | 8.17E-237 |
| ZNF703 | 1.05 | 2.39E-19 |
| SLC35F3 | -1.108 | 2.63E-22 |
| OLFML1 | -1.081 | 4.00E-49 |
| FAM162B | -2.141 | 0.00E+00 |
| LIN9 | 1.018 | 9.63E-97 |
| GPR39 | 1.078 | 2.91E-39 |
| FAM3B | -1.114 | 6.23E-03 |
| KIRREL | -1.094 | 7.59E-63 |
| IQGAP3 | 2.542 | 1.64E-211 |
| NPIPA5 | -1.196 | 2.47E-82 |
| SMTN | -1.543 | 5.44E-125 |
| ST6GALNAC3 | -1.585 | 6.83E-218 |
| TMPRSS2 | -1.142 | 2.72E-07 |
| NIPSNAP1 | 1.3 | 3.77E-166 |
| FAM132A | -1.047 | 7.77E-48 |
| TSPYL2 | -1.987 | 5.34E-206 |
| GOLGA6L4 | -1.73 | 2.29E-236 |
| OAF | -1.236 | 5.49E-98 |
| POU6F1 | -1.651 | 8.51E-228 |
| PRKD1 | -1.39 | 6.91E-162 |
| SLIT3 | -1.918 | 9.69E-132 |
| PKP3 | 1.082 | 1.27E-126 |
| PLA2G6 | -1.958 | 2.67E-130 |
| MAML2 | -1.621 | 3.61E-113 |
| WDR27 | -1.605 | 1.26E-129 |
| TMEM255B | -1.769 | 6.34E-132 |
| PROS1 | -1.895 | 1.27E-151 |
| SOCS3 | -1.045 | 3.40E-40 |
| SLITRK6 | 1.149 | 8.52E-23 |
| NELL2 | 1.247 | 7.48E-39 |
| ZNF93 | 1.186 | 2.22E-101 |
| HIST2H2BE | 1.459 | 8.36E-46 |
| FAM110C | 1.067 | 8.40E-38 |
| SMIM10 | -1.52 | 1.74E-185 |
| TUSC5 | -4.899 | 4.27E-206 |
| TMED9 | 1.029 | 4.18E-167 |
| TCEAL2 | -1.895 | 7.68E-101 |
| F8 | -1.082 | 3.07E-91 |
| MAFF | -2.246 | 4.42E-189 |
| FLRT2 | -1.709 | 4.43E-101 |
| MANEAL | 1.935 | 3.77E-131 |
| FAM43A | -1.612 | 6.15E-176 |
| NRBP2 | -1.934 | 4.13E-133 |
| NUPR1L | 1.163 | 3.68E-35 |
| IL3RA | -1.137 | 1.68E-100 |
| TCN2 | -1.047 | 1.47E-78 |
| MAPK11 | -1.454 | 1.72E-121 |
| SMYD3 | 1.425 | 2.23E-130 |
| METTL7A | -1.656 | 2.31E-156 |
| FAM174B | 1.287 | 2.53E-64 |
| KRT6B | -1.059 | 1.58E-03 |
| PARPBP | 1.149 | 1.33E-113 |
| SDHAP1 | -1.027 | 2.14E-81 |
| RP11-504P24.3 | -1.027 | 9.04E-99 |
| MUC1 | 2.443 | 3.15E-81 |
| L3MBTL1 | -1.219 | 3.02E-92 |
| SV2B | -1.043 | 1.06E-132 |
| P4HB | 1.04 | 1.23E-142 |
| NTF3 | -1.282 | 1.79E-71 |
| EP400NL | -1.019 | 2.56E-71 |
| SLC52A2 | 1.249 | 9.49E-132 |
| NAT8L | -2.11 | 6.74E-109 |
| RPL12P4 | -1.162 | 8.71E-89 |
| NPIPB4 | -1.021 | 1.02E-48 |
| PTCH1 | -1.176 | 6.35E-109 |
| RNPC3 | -1.545 | 1.48E-171 |
| IRS2 | -1.931 | 6.04E-120 |
| RASA3 | -1.106 | 6.84E-96 |
| KRT5 | -3.766 | 7.26E-35 |
| LRRC70 | -1.905 | 1.67E-243 |
| KIF18B | 1.734 | 9.95E-151 |
| SAPCD2 | 1.926 | 6.17E-162 |
| 1-Mar | -1.907 | 3.61E-100 |
| GPAT2 | -1.738 | 2.39E-66 |
| TOR3A | 1.052 | 6.30E-175 |
| GOLGA8R | -1.249 | 2.54E-198 |
| GLDN | -1.822 | 3.87E-156 |
| GNG2 | -1.27 | 1.41E-98 |
| MIR22HG | -1.596 | 1.61E-147 |
| KIF24 | 1.015 | 8.31E-139 |
| PDE2A | -3.93 | 0.00E+00 |
| MST1L | -1.229 | 3.14E-90 |
| LILRB4 | 1.431 | 5.37E-79 |
| KRT16 | -1.217 | 2.34E-03 |
| KRT14 | -3.751 | 5.77E-34 |
| MAPT | 1.17 | 8.49E-08 |
| ERCC6L | 1.266 | 1.38E-150 |
| TNFRSF18 | 1.545 | 3.75E-75 |
| ZDHHC17 | -1.099 | 3.40E-101 |
| SERPINA11 | 1.236 | 8.49E-21 |
| ZACN | 3.104 | 5.64E-69 |
| KANK3 | -2.919 | 1.09E-286 |
| ESPN | 1.303 | 5.53E-39 |
| MITF | -1.246 | 9.14E-87 |
| SHTN1 | 1.359 | 2.31E-110 |
| MT1X | -2.441 | 2.10E-147 |
| WDR86 | -2.384 | 3.88E-202 |
| EPOR | -1.019 | 1.47E-70 |
| FPR3 | 1.672 | 2.09E-70 |
| KCNJ11 | 1.255 | 1.40E-36 |
| GJA4 | -1.63 | 5.36E-127 |
| ISG15 | 2.341 | 2.17E-99 |
| EXD3 | -1.037 | 1.10E-59 |
| THSD4 | 1.167 | 3.13E-20 |
| GABRD | 1.187 | 5.29E-121 |
| FANCA | 1.233 | 7.95E-93 |
| PEAR1 | -2.851 | 0.00E+00 |
| TMEM220 | -2.013 | 1.06E-260 |
| HIST1H1C | 1.475 | 5.75E-42 |
| LCN10 | -1.841 | 8.08E-210 |
| LDLRAD2 | -1.823 | 3.68E-143 |
| WNT7B | 1.712 | 1.04E-83 |
| MAPK12 | -1.007 | 2.87E-39 |
| TUBB4B | 1.229 | 1.37E-148 |
| PLA2G2A | -3.8 | 4.31E-86 |
| SBK1 | 2.238 | 5.02E-201 |
| PRR19 | 1.211 | 7.47E-200 |
| ZP3 | 1.27 | 3.43E-67 |
| SELL | 1.102 | 1.87E-40 |
| H2AFX | 1.582 | 1.43E-183 |
| HBA2 | -3.97 | 4.62E-183 |
| NPIPP1 | -1.297 | 6.70E-119 |
| GOLGA8M | -1.312 | 4.10E-301 |
| SMIM15 | 1.311 | 2.75E-90 |
| PRELP | -2.691 | 5.71E-152 |
| FAM26F | 1.109 | 1.95E-46 |
| USP32P1 | -2.085 | 1.85E-45 |
| RELN | -1.887 | 4.49E-186 |
| FAM111B | 2.65 | 4.85E-186 |
| PLAC9 | -3.301 | 5.32E-218 |
| UBE2Q2P1 | -1.972 | 3.18E-223 |
| HN1 | 2.853 | 2.04E-284 |
| PCDH18 | -1.42 | 2.43E-91 |
| MAOA | -3.425 | 3.38E-191 |
| PAX8-AS1 | -1.262 | 7.19E-13 |
| FAM150B | -3.156 | 1.35E-210 |
| S100A14 | 3.313 | 1.49E-135 |
| KIAA0408 | -1.69 | 7.55E-233 |
| MMP23B | -1.13 | 7.19E-46 |
| MYBPC1 | -2.712 | 8.82E-47 |
| KIAA0895L | -1.624 | 9.57E-85 |
| SERPINA3 | 1.069 | 1.80E-13 |
| S100A4 | -1.191 | 1.30E-55 |
| FAT4 | -1.206 | 2.79E-137 |
| ZNF681 | 1.009 | 9.48E-79 |
| HRCT1 | -2.662 | 1.99E-105 |
| GREB1 | 1.056 | 5.27E-09 |
| TUBB | 1.161 | 5.69E-147 |
| ZNF471 | -1.266 | 6.86E-115 |
| GIMAP5 | -1.926 | 8.58E-143 |
| EVL | 1.174 | 8.87E-32 |
| SPTSSB | 1.38 | 2.94E-30 |
| MAN2A2 | -1.413 | 5.05E-130 |
| MME | -2.651 | 1.81E-158 |
| LAMA2 | -2.254 | 3.19E-153 |
| MYO6 | 1.269 | 8.75E-63 |
| MMP1 | 2.158 | 1.90E-77 |
| TCF4 | -1.028 | 9.86E-60 |
| TTC30B | 1.009 | 1.21E-74 |
| TLR7 | 1.05 | 1.09E-47 |
| HSH2D | 1.855 | 1.31E-83 |
| PDXDC2P | -1.44 | 2.53E-97 |
| HLA-DQA1 | 1.232 | 8.28E-36 |
| HIST1H2AI | 1.516 | 5.96E-79 |
| ZNF239 | 1.061 | 5.52E-79 |
| HIST1H2AD | 1.642 | 1.62E-55 |
| LAMB3 | -1.243 | 3.10E-21 |
| ZNF252P | 1.04 | 3.93E-45 |
| LAGE3 | 1.816 | 1.63E-200 |
| ZNF300P1 | -1.451 | 1.49E-246 |
| HIST1H4J | 1.767 | 1.00E-93 |
| FAM110D | -2.197 | 3.10E-222 |
| SERPINA1 | 1.047 | 1.77E-16 |
| KANK2 | -1.289 | 1.81E-113 |
| RAMP2-AS1 | -1.681 | 1.08E-90 |
| GATA3-AS1 | 1.198 | 3.08E-17 |
| PELI1 | -1.265 | 2.24E-94 |
| HIST1H3D | 1.665 | 2.24E-58 |
| HNRNPAB | 1.068 | 1.15E-188 |
| PDGFA | -1.07 | 1.20E-62 |
| ZNF695 | 1.114 | 2.12E-87 |
| SLC2A10 | 1.204 | 3.19E-40 |
| SLC28A3 | -1.996 | 2.52E-39 |
| MIB2 | -1.542 | 1.61E-83 |
| HOXA4 | -2.291 | 7.34E-254 |
| NMB | -1.68 | 3.18E-117 |
| MAP1LC3C | -2.481 | 1.60e-311 |
| KLHDC1 | -1.124 | 5.64E-154 |
| OCLN | 1.631 | 1.60E-90 |
| FAM49A | -1.422 | 5.15E-140 |
| HIST1H2BK | 2.034 | 4.44E-116 |
| ZNF677 | -1.47 | 9.51E-93 |
| PLCG2 | -1.2 | 1.19E-75 |
| FCHSD1 | -1.075 | 3.55E-76 |
| SNHG12 | -1.067 | 4.67E-46 |
| MRPL42 | 1.054 | 1.76E-119 |
| ENTPD7 | 1.475 | 1.72E-122 |
| FCGR1B | 1.042 | 9.60E-79 |
| SIRPA | -1.271 | 1.58E-91 |
| RP11-347C12.1 | -1.025 | 2.26E-72 |
| SULT1C4 | -1.344 | 2.49E-85 |
| LPAR1 | -1.218 | 7.18E-89 |
| MB | 1.767 | 1.53E-83 |
| TMEM229B | 1.173 | 1.52E-61 |
| ZNF334 | -1.397 | 4.42E-57 |
| HSD17B11 | -1.558 | 8.47E-126 |
| SLC29A3 | 1.02 | 2.49E-90 |
| PEG3 | -1.068 | 8.20E-11 |
| HYLS1 | 1.194 | 4.08E-144 |
| TPM2 | -1.512 | 6.55E-78 |
| SH3BGRL2 | -1.918 | 2.49E-181 |
| MAFK | -1.555 | 6.91E-116 |
| PLN | -1.461 | 2.04E-91 |
| ZNF28 | 1.308 | 3.92E-118 |
| WDHD1 | 1.19 | 1.85E-135 |
| LRBA | 1.255 | 1.08E-76 |
| PPIAP22 | 2.235 | 8.26E-258 |
| FAM3D | -1.712 | 1.99E-40 |
| TAT | -2.754 | 1.59E-26 |
| MT-CYB | -1.35 | 4.35E-112 |
| SMOC1 | -1.666 | 4.79E-36 |
| MT-ND2 | -1.532 | 4.32E-116 |
| ZNF521 | -1.085 | 4.83E-43 |
| PAX9 | 1.392 | 2.62E-66 |
| RYR3 | -1.373 | 7.40E-272 |
| MT-ND3 | -1.313 | 9.27E-112 |
| RUSC2 | -1.005 | 1.49E-100 |
| GRK5 | -1.59 | 1.33E-186 |
| ITPRIPL1 | -1.336 | 3.55E-165 |
| MT-ND4 | -1.111 | 1.49E-83 |
| MT-ND1 | -1.339 | 1.71E-107 |
| MT-ATP6 | -1.224 | 3.05E-95 |
| PRC1 | 2.302 | 2.67E-183 |
| GPRASP1 | -2.1 | 5.07E-268 |
| TBKBP1 | -1.038 | 1.17E-82 |
| RNU4-1 | 2.954 | 1.93E-17 |
| FAM83H-AS1 | 2.274 | 2.06E-213 |
| SAMD5 | -1.255 | 4.81E-74 |
| FCGR3A | 1.633 | 2.59E-93 |
| PPAPDC1A | 2.921 | 7.18E-176 |
| HIST2H2AA3 | 1.77 | 4.88E-51 |
| SOX18 | -1.802 | 3.13E-140 |
| TCEAL5 | -1.398 | 5.04E-115 |
| GGTA1P | -1.378 | 5.60E-83 |
| TMEM57 | 1.044 | 2.71E-119 |
| TCEA3 | 1.257 | 7.59E-66 |
| HNRNPCP2 | 2.368 | 1.66E-251 |
| NOTCH4 | -2.241 | 4.15E-269 |
| FKBPL | 1.015 | 1.40E-168 |
| SLC44A4 | 3.09 | 1.52E-62 |
| NEU1 | 1.063 | 6.60E-133 |
| HSPA1B | 1.098 | 9.65E-62 |
| HSPA1A | 1.154 | 1.19E-40 |
| LST1 | 1.101 | 1.01E-55 |
| ZNF468 | 1.139 | 2.55E-68 |
| RNF39 | -1.678 | 1.59E-33 |
| GABBR1 | -1.508 | 1.90E-100 |
| ZNF204P | -1.532 | 6.22E-92 |
| IGFL2 | 1.097 | 3.78E-58 |
| PKHD1L1 | -1.506 | 7.65E-155 |
| SYCE1L | -1.252 | 2.71E-63 |
| PSENEN | 1.581 | 5.72E-254 |
| LGR4 | -1.005 | 4.83E-35 |
| VIT | -2.894 | 1.42E-265 |
| MT1A | -2.885 | 5.06E-101 |
| MT1M | -3.463 | 9.73E-246 |
| SAMD9 | 1.154 | 1.46E-49 |
| HMGN1 | 1.156 | 2.79E-166 |
| STAG3L1 | -1.142 | 7.65E-89 |
| ITSN1 | -1.169 | 1.07E-126 |
| RP11-1212A22.1 | -1.692 | 2.54E-77 |
| HN1L | 2.036 | 1.15E-217 |
| SERPINB5 | -1.268 | 9.27E-06 |
| HERC2P9 | -2.107 | 1.59E-200 |
| HBA1 | -3.792 | 1.80E-176 |
| H1FX-AS1 | -1.483 | 3.71E-116 |
| VGLL3 | -1.659 | 1.88E-137 |
| RNU1-106P | 2.42 | 1.85E-32 |
| SNORA70 | -1.257 | 1.33E-09 |
| GPX3 | -5.067 | 1.39E-286 |
| TRBV25-1 | 1.302 | 1.95E-33 |
| TRBC2 | 1.285 | 5.98E-28 |
| IGHA2 | -1.657 | 7.32E-05 |
| IGHG4 | 3.68 | 1.46E-96 |
| IGHG2 | 1.692 | 1.39E-37 |
| IGHG1 | 2.8 | 6.68E-52 |
| IGHG3 | 2.67 | 5.11E-53 |
| IGHV1-18 | 1.107 | 1.14E-14 |
| IGHV3-33 | 1.068 | 8.61E-09 |
| LINC01089 | -1.016 | 2.19E-60 |
| MT-ND4L | -1.11 | 5.47E-59 |
| TCTEX1D2 | 1.253 | 6.49E-131 |
| YWHAZP4 | 1.558 | 3.00E-101 |
| TRIM59 | 1.735 | 7.51E-197 |
| GIMAP1 | -1.393 | 6.26E-146 |
| YWHAZP5 | 1.601 | 8.77E-88 |
| GSTM2 | -2.395 | 2.56E-136 |
| LCAT | -2.789 | 5.13E-231 |
| VDAC1 | 1.023 | 9.18E-139 |
| ZBTB9 | 1.31 | 1.82E-152 |
| SLX1A-SULT1A3 | -1.063 | 8.43E-35 |
| SULT1A4 | -1.207 | 7.64E-65 |
| UBD | 2.284 | 1.01E-50 |
| LTB4R | -1.082 | 8.21E-67 |
| GALT | -1.105 | 1.24E-119 |
| TTLL3 | -1.855 | 1.28E-142 |
| FBXO16 | 1.04 | 3.86E-81 |
| TSPAN4 | -1.215 | 1.44E-98 |
| MYCBP | 1.278 | 1.15E-139 |
| PRCD | -2.419 | 0.00E+00 |
| PLEKHM1P | -1.401 | 2.65E-125 |
| HNRNPA1P10 | 1.141 | 1.14E-62 |
| RP11-252A24.2 | -1.145 | 3.08E-121 |
| FAM187A | 1.096 | 4.62E-68 |
| PLIN5 | -2.008 | 6.64E-70 |
| STARD10 | 2.481 | 4.55E-133 |
| MEG3 | -3.253 | 8.96E-215 |
| HMGN2P15 | -1.839 | 1.11E-85 |
| RP11-480I12.5 | 1.318 | 1.28E-91 |
| HLA-F-AS1 | -1.112 | 6.34E-147 |
| NEURL4 | -1.022 | 2.41E-80 |
| FAM166B | -1.048 | 2.61E-70 |
| GOLGA8B | -2.429 | 1.19E-145 |
| MIR99AHG | -1.61 | 1.64E-111 |
| NPEPL1 | -1.072 | 8.93E-38 |
| RP13-104F24.2 | -1.838 | 4.73E-155 |
| TNFRSF25 | -1.062 | 2.39E-48 |
| TSTD1 | 1.354 | 1.15E-114 |
| HIST1H1PS1 | 1.735 | 4.80E-62 |
| IFI30 | 1.97 | 1.29E-157 |
| RPS2P55 | 1.231 | 6.94E-112 |
| SYCE3 | 1.06 | 2.69E-55 |
| RP11-204C16.4 | 1.357 | 5.46E-75 |
| PP14571 | 1.341 | 2.25E-48 |
| RP11-40C6.2 | 5.642 | 6.70E-173 |
| MTMR9LP | -1.755 | 4.79E-139 |
| PLXNA4 | -1.779 | 1.06E-154 |
| HEPN1 | -2.395 | 1.68E-239 |
| SLC12A8 | 1.762 | 1.14E-188 |
| FADS3 | -2.125 | 3.55E-201 |
| RNU2-2P | -1.504 | 1.09E-24 |
| RP11-211G3.2 | -1.193 | 3.07E-186 |
| RP11-632K20.7 | -1.907 | 2.06E-224 |
| TINCR | 1.318 | 2.85E-45 |
| RP11-289I10.2 | 1.232 | 6.10E-84 |
| NSUN5P1 | -1.323 | 6.47E-63 |
| RP4-717I23.3 | -1.456 | 8.53E-122 |
| TSSC2 | -1.816 | 4.80E-271 |
| RPS20P14 | -1.408 | 2.01E-121 |
| RP11-428L9.2 | 1.043 | 7.30E-45 |
| EPB41L4A-AS1 | -1.1 | 6.70E-91 |
| UBE2SP2 | 2.789 | 6.10E-255 |
| RP11-561I11.3 | -1.057 | 2.21E-171 |
| SNRPGP15 | -1.052 | 4.39E-35 |
| SH3BP5-AS1 | -2.027 | 6.30E-196 |
| RP11-651P23.4 | 1.333 | 2.75E-83 |
| RPL29P11 | -1.178 | 2.71E-65 |
| PRRT4 | -1.853 | 1.57E-166 |
| PGM5-AS1 | -2.294 | 0.00E+00 |
| RP11-228B15.4 | -1.83 | 5.49E-209 |
| RP11-415J8.3 | -1.49 | 1.98E-137 |
| RPL35P5 | 1.156 | 2.13E-60 |
| MTND2P28 | -1.55 | 3.34E-42 |
| FAM229A | -1.638 | 8.59E-126 |
| RP11-384K6.2 | -1.162 | 6.52E-104 |
| NTF4 | -2.225 | 4.55E-92 |
| MTND1P23 | -1.167 | 1.10E-08 |
| RP4-706A16.3 | 1.154 | 2.79E-33 |
| RP11-536O18.1 | -1.644 | 2.61E-111 |
| RP11-419C5.2 | -1.526 | 9.15E-88 |
| TMEM191A | 1.092 | 6.42E-90 |
| FTH1P20 | 1.252 | 1.41E-73 |
| FTLP3 | -1.147 | 9.90E-96 |
| TEX41 | -1.074 | 3.02E-132 |
| LENG8-AS1 | -1.024 | 1.20E-84 |
| ERVMER34-1 | 1.005 | 4.72E-52 |
| LINC00511 | 1.056 | 1.85E-59 |
| WASH7P | -1.477 | 5.66E-127 |
| RP11-258C19.4 | -1.986 | 1.36E-18 |
| LINC01537 | -1.386 | 0.00E+00 |
| LTB | 1.533 | 1.00E-50 |
| RP1-28O10.1 | -3.269 | 1.42E-225 |
| RP11-864N7.2 | -1.244 | 8.15E-94 |
| HIGD1AP11 | -1.115 | 7.14E-108 |
| MT-ATP8 | -1.362 | 3.54E-72 |
| PCAT6 | 1.016 | 6.87E-78 |
| GUSBP11 | -1.935 | 3.23E-154 |
| RP11-251M1.1 | -1.082 | 7.17E-192 |
| LINC01125 | -1.316 | 5.33E-102 |
| RAB11FIP1P1 | -1.012 | 1.73E-82 |
| RP1-241P17.4 | 1.206 | 5.91E-33 |
| SPCS2P4 | 1.159 | 9.51E-96 |
| HOTAIR | 1.576 | 8.84E-56 |
| OR2A9P | -1.014 | 6.11E-85 |
| RP11-286B14.1 | -1.755 | 1.24E-90 |
| VDAC1P8 | -1.006 | 4.37E-117 |
| MEOX2-AS1 | -1.919 | 1.25E-276 |
| RP11-20J15.3 | -1.165 | 2.00E-206 |
| GS1-124K5.11 | -1.03 | 1.44E-190 |
| PGA4 | -1.857 | 1.45E-191 |
| HMGN1P37 | 3.076 | 9.94E-216 |
| RP11-274B21.12 | -1.198 | 2.20E-75 |
| MBNL1-AS1 | -1.181 | 4.83E-108 |
| RP11-305L7.6 | -1.311 | 2.05E-224 |
| PGA3 | -2.074 | 5.39E-202 |
| RP11-481H12.1 | -1.973 | 1.56E-216 |
| TMSB4XP6 | -4.422 | 3.21E-29 |
| TRPM2-AS | 1.493 | 9.51E-37 |
| HOXB-AS1 | -1.438 | 7.97E-96 |
| RP11-632C17__A.1 | -1.012 | 7.75E-53 |
| GOLGA6L5P | -2.671 | 3.31E-260 |
| TCEA1P2 | 1.393 | 1.56E-38 |
| RPS23P8 | -1.378 | 1.65E-41 |
| RP11-274B21.4 | -1.341 | 1.94E-104 |
| FOXD3-AS1 | 1.617 | 8.28E-61 |
| STMND1 | 1.787 | 9.67E-45 |
| RP3-525N10.2 | -1.372 | 3.37E-248 |
| MIR205HG | -1.735 | 1.63E-06 |
| RP4-631H13.6 | -1.755 | 1.28E-67 |
| KLF3-AS1 | -1.533 | 1.33E-195 |
| IGHV4-31 | 1.363 | 4.64E-14 |
| LINC-PINT | -1.679 | 2.59E-207 |
| TRAF3IP2-AS1 | -1.114 | 4.09E-177 |
| PGM5P4-AS1 | -1.275 | 3.44E-244 |
| FLJ27354 | -1.477 | 8.82E-222 |
| LSM12P1 | 1.246 | 1.28E-46 |
| RP11-14N7.2 | -1.163 | 2.30E-84 |
| HLA-DQB2 | 1.213 | 4.15E-41 |
| RP11-379F12.4 | 1.348 | 5.05E-42 |
| GOLGA8N | -1.775 | 3.35E-227 |
| LINC00665 | 1.029 | 1.72E-69 |
| SLCO4A1-AS1 | -1.187 | 2.44E-69 |
| LINC00342 | -1.311 | 2.35E-87 |
| RP11-5P18.5 | 1.72 | 6.97E-18 |
| LINC00702 | -1.212 | 4.52E-125 |
| USP32P2 | -1.052 | 1.35E-34 |
| HOTAIRM1 | -2.606 | 3.50E-213 |
| RP11-295G20.2 | 1.116 | 6.10E-75 |
| TMEM238 | 1.148 | 3.07E-71 |
| TWIST2 | -3.041 | 1.06E-246 |
| UBE2SP1 | 1.131 | 3.09E-89 |
| H2BFS | 1.664 | 2.19E-111 |
| ZNF37BP | -1.461 | 8.08E-141 |
| MAGI2-AS3 | -2.482 | 3.54E-274 |
| PINLYP | -1.45 | 3.88E-151 |
| HMGN2P5 | 1.509 | 2.92E-96 |
| RP11-166B2.1 | -1.082 | 2.77E-54 |
| SOX9-AS1 | -2.353 | 8.20E-29 |
| FABP5P7 | -1.781 | 3.11E-54 |
| SEMA3F-AS1 | -1.271 | 1.41E-167 |
| HMGN1P30 | 1.705 | 4.07E-51 |
| RP11-61I13.3 | -2.411 | 2.00E-306 |
| TECRP1 | 1.083 | 2.39E-77 |
| HGH1 | 1.124 | 1.88E-150 |
| RP11-122K13.12 | -1.004 | 1.72E-118 |
| FAM103A2P | 1.443 | 7.77E-62 |
| RP11-70C1.3 | 2.427 | 6.06E-11 |
| RP11-575L7.8 | -1.066 | 1.11E-51 |
| SNRPGP10 | -1.794 | 2.32E-79 |
| LINC00961 | -2.15 | 3.46E-213 |
| RP4-639F20.1 | -1.131 | 2.65E-86 |
| RP11-693N9.2 | -1.57 | 3.75E-151 |
| RPS2P7 | -1.286 | 1.69E-121 |
| H3F3AP4 | 1.788 | 2.26E-93 |
| LINC00894 | -1.444 | 4.70E-110 |
| HMGN1P36 | 2.488 | 6.10E-153 |
| RP11-215A21.2 | 1.181 | 1.19E-74 |
| HSPB1P1 | 3.342 | 7.40E-113 |
| TRHDE-AS1 | -2.78 | 4.18E-294 |
| RP11-175B9.3 | -2.122 | 2.09E-141 |
| LINC00106 | -1.09 | 1.05E-97 |
| RP4-669L17.10 | -1.213 | 2.49E-66 |
| HAND2-AS1 | -1.37 | 5.03E-241 |
| TTN-AS1 | -1.044 | 2.53E-81 |
| HOXD-AS2 | -1.303 | 2.04E-123 |
| HLA-DQA2 | 1.283 | 6.16E-33 |
| KIFC1 | 2.746 | 8.50E-213 |
| LINC00844 | -1.844 | 3.51E-187 |
| SNORD10 | -1.918 | 9.94E-07 |
| RPL23AP1 | -1.659 | 1.32E-186 |
| RP11-295P9.3 | -1.425 | 3.86E-136 |
| NME1 | 1.631 | 1.73E-167 |
| RP4-800G7.1 | 1.735 | 5.68E-14 |
| RN7SL608P | -1.221 | 7.84E-115 |
| IGKV1D-33 | 1.222 | 1.19E-14 |
| LY6G5B | -2.441 | 6.72E-197 |
| SMKR1 | 1.835 | 4.31E-120 |
| KRBOX1 | -1.367 | 2.72E-35 |
| MIF | 1.204 | 6.59E-87 |
| RP11-79D8.2 | -1.114 | 3.80E-55 |
| RPL37P6 | 1.251 | 2.53E-55 |
| IDS | -1.267 | 1.45E-60 |
| INMT | -1.987 | 2.81E-160 |
| LINC00893 | -1.888 | 1.17E-125 |
| TCEB1P19 | -1.244 | 2.15E-98 |
| LINC00969 | -1.168 | 1.09E-81 |
| STAG3L5P | -1.454 | 5.41E-64 |
| IGKV2D-28 | 1.706 | 2.19E-13 |
| RP11-274B21.14 | -1.262 | 6.17E-79 |
| MICAL3 | -1.159 | 1.69E-77 |
| STON1 | -1.379 | 1.12E-125 |
| RP11-274B21.2 | -1.269 | 2.42E-93 |
| RP11-379F12.3 | 1.739 | 2.38E-45 |
| RPL23AP49 | -1.078 | 1.78E-61 |
| TNFRSF6B | -1.196 | 4.15E-38 |
| RP11-274B21.3 | -1.735 | 3.46E-122 |
| PLA2G4B | -1.657 | 7.08E-114 |
| NPIPB5 | -1.634 | 9.26E-95 |
| RPLP0P2 | 1.427 | 1.34E-141 |
| WDR86-AS1 | -1.806 | 7.25E-178 |
| GSTA1 | -1.531 | 4.16E-21 |
| RN7SL417P | -1.39 | 8.42E-208 |
| TMEM141 | 1.287 | 4.58E-126 |
| PKD1P1 | -2.044 | 1.11E-89 |
| RP11-10G12.1 | 2.107 | 1.92E-100 |
| RP4-800G7.2 | -2.121 | 1.61E-117 |
| RP3-449O17.1 | -1.52 | 8.25E-153 |
| RP11-20O24.4 | -1.15 | 9.32E-109 |
| HBB | -3.865 | 8.05E-184 |
| NEAT1 | -2.259 | 9.69E-91 |
| RP11-175K6.1 | -2.803 | 3.94e-314 |
| RAD51-AS1 | -1.133 | 1.48E-89 |
| RP11-696N14.1 | -1.477 | 5.87E-285 |
| LINC00968 | -1.262 | 1.13E-175 |
| RP11-894P9.1 | -1.246 | 3.72E-141 |
| UBAP1L | -1.08 | 1.03E-170 |
| SOCS2-AS1 | -1.424 | 3.93E-58 |
| PGAM5 | 1.17 | 1.15E-147 |
| MTND4P12 | 4.005 | 2.10E-28 |
| NR2F2-AS1 | -1.251 | 2.24E-183 |
| LINC00926 | -1.115 | 3.74E-51 |
| NRAV | 1.208 | 2.73E-116 |
| FAM13A-AS1 | -1.557 | 3.17E-223 |
| RRN3P1 | -2.305 | 3.80E-289 |
| TRIM52-AS1 | -1.194 | 2.01E-159 |
| MEF2C-AS1 | -1.121 | 2.03E-212 |
| WHAMMP2 | -1.37 | 1.92E-304 |
| LINC00504 | 1.254 | 1.40E-31 |
| LINC01197 | -1.359 | 1.35E-225 |
| SEMA6A-AS1 | -1.27 | 6.65E-161 |
| MTATP6P1 | -1.295 | 3.13E-59 |
| ZNF436-AS1 | -1.566 | 2.40E-242 |
| LINC01088 | -1.32 | 4.08E-141 |
| MIR143HG | -2.936 | 0.00E+00 |
| RP11-423H2.3 | 1.085 | 5.28E-54 |
| ECSCR | -1.848 | 2.17E-170 |
| YJEFN3 | -1.196 | 5.15E-61 |
| FZD10-AS1 | -1.668 | 4.83E-104 |
| PKD1P6 | -1.962 | 2.64E-116 |
| RP11-356J5.12 | -1.099 | 1.51E-86 |
| KIAA1456 | -1.687 | 1.06E-128 |
| HOXC-AS1 | -1.205 | 1.11E-84 |
| RP4-669L17.8 | -1.068 | 2.91E-71 |
| RP11-834C11.7 | -1.127 | 1.50E-131 |
| SEPP1 | -1.043 | 7.03E-44 |
| ZBED3-AS1 | -1.003 | 1.97E-154 |
| HOXC-AS3 | 1.067 | 1.45E-54 |
| SHANK3 | -2.748 | 5.54E-263 |
| LINC01094 | 1.222 | 1.21E-104 |
| MALAT1 | -1.319 | 1.39E-72 |
| RNA5SP216 | -1.918 | 4.52E-32 |
| RN7SKP70 | -1.289 | 3.54E-110 |
| RP11-459E5.1 | 1.317 | 6.98E-50 |
| RP11-582J16.5 | -1.097 | 6.44E-165 |
| HOXA10 | -1.373 | 2.33E-67 |
| TMEM200B | -1.394 | 6.31E-72 |
| TRNP1 | -1.08 | 5.35E-43 |
| HOXA-AS2 | -1.657 | 3.36E-169 |
| IGHGP | 2.977 | 6.18E-75 |
| RBPMS-AS1 | -1.39 | 5.23E-205 |
| PCDHGB7 | -1.221 | 1.58E-104 |
| RP11-598P20.3 | 1.088 | 3.39E-40 |
| LINC01485 | -2.057 | 4.07E-161 |
| RHPN1-AS1 | 1.246 | 1.45E-148 |
| HSPB2-C11orf52 | -2.515 | 2.41E-256 |
| RP11-867G23.4 | 1.278 | 2.03E-29 |
| RP11-676M6.1 | 1.164 | 7.46E-50 |
| RP11-152H18.3 | -1.177 | 4.60E-17 |
| RP11-265D17.2 | 1.338 | 1.52E-58 |
| RP11-468E2.1 | 1.476 | 2.18E-57 |
| RP11-805J14.5 | 1.339 | 1.93E-126 |
| MEX3A | 1.864 | 3.42E-98 |
| NAV2-AS1 | -1.477 | 2.84E-80 |
| RP11-110I1.5 | -1.314 | 2.88E-67 |
| STX16-NPEPL1 | -1.546 | 5.22E-85 |
| RP11-326C3.2 | -1.338 | 3.74E-13 |
| SAA2-SAA4 | -1.863 | 3.19E-95 |
| PIGY | 1.38 | 5.40E-33 |
| MIR100HG | -1.043 | 7.80E-111 |
| RP11-350N15.4 | -2.013 | 1.00E-96 |
| RP11-166D19.1 | -1.775 | 1.85E-149 |
| RP11-624G17.3 | 1.019 | 7.31E-92 |
| RP11-411B6.6 | -1.04 | 1.03E-87 |
| HCAR3 | -1.018 | 3.16E-132 |
| RP11-736K20.5 | -1.834 | 4.84E-237 |
| MT1JP | -1.176 | 9.93E-151 |
| LINC01152 | -1.777 | 7.59E-86 |
| SMIM3 | -1.681 | 1.31E-156 |
| RP11-424C20.2 | 1.123 | 3.12E-112 |
| RP11-817J15.2 | -1.637 | 2.26E-21 |
| RP11-783K16.5 | 1.113 | 4.75E-59 |
| SNRPEP2 | 1.624 | 1.78E-177 |
| HP | -1.691 | 1.31E-60 |
| U47924.27 | 1.171 | 5.49E-85 |
| RP11-203J24.9 | -2.467 | 1.61E-161 |
| RP11-579D7.2 | 1.259 | 1.72E-20 |
| RP11-1100L3.7 | 1.175 | 1.29E-70 |
| RP3-416H24.1 | -1.186 | 4.64E-33 |
| RP11-1143G9.4 | 1.272 | 7.51E-19 |
| RP11-386G11.10 | 2.096 | 2.97E-115 |
| RP11-161H23.5 | 2.068 | 4.17E-134 |
| RP11-644F5.10 | -1.756 | 1.21E-295 |
| RP11-649E7.5 | 2.469 | 1.33E-59 |
| PPT2-EGFL8 | -1.762 | 6.69E-144 |
| LINC00641 | -1.458 | 7.97E-210 |
| RP11-164J13.1 | -1.73 | 1.82E-258 |
| RP11-203M5.7 | -1.069 | 1.13E-126 |
| RHOXF1-AS1 | -1.49 | 2.45E-158 |
| RP11-463D19.2 | 1.007 | 2.59E-24 |
| RP11-66N24.3 | -1.251 | 5.60E-88 |
| RNASE4 | -1.348 | 2.06E-82 |
| RP11-7F17.3 | -1.026 | 8.64E-267 |
| TUBB3 | 3.059 | 1.23E-139 |
| RP6-65G23.3 | 1.531 | 8.23E-141 |
| RP11-903H12.5 | -1.035 | 7.05E-48 |
| ITGB3 | -1.027 | 1.79E-59 |
| RP11-182J1.12 | -1.981 | 9.02E-220 |
| PAK6 | 1.616 | 1.70E-105 |
| RP11-798K3.2 | -1.007 | 6.58E-190 |
| RP11-109D20.2 | 1.05 | 5.19E-38 |
| RP11-316M1.12 | 2.952 | 9.59E-71 |
| TGIF2-C20orf24 | 1.597 | 1.78E-53 |
| RP11-158M2.3 | -1.027 | 5.23E-240 |
| RP11-321G12.1 | 1.334 | 4.80E-25 |
| SORD2P | 2.171 | 1.81E-123 |
| RP11-244F12.3 | -1.005 | 3.80E-133 |
| RP11-66B24.4 | -1.103 | 4.55E-124 |
| RP11-66B24.1 | -1.706 | 4.60E-104 |
| RP11-89K11.1 | -1.648 | 2.24E-243 |
| RP11-553L6.5 | -1.301 | 1.76E-116 |
| RP11-490M8.1 | -1.455 | 1.02E-130 |
| LA16c-390E6.4 | -1.042 | 5.40E-91 |
| RP5-1142A6.9 | -1.703 | 5.17E-153 |
| RP11-347C12.10 | 1.353 | 5.07E-104 |
| RP11-483P21.2 | 1.32 | 2.43E-45 |
| RP11-645C24.5 | -1.113 | 2.95E-269 |
| MRC1 | -1.615 | 7.19E-81 |
| RP11-63M22.2 | -1.539 | 1.38E-10 |
| MT1L | -2.03 | 2.42E-100 |
| RP13-516M14.1 | -1.206 | 1.32E-139 |
| RP11-23N2.4 | -1.046 | 3.85E-56 |
| RP11-72I8.1 | -1.204 | 4.52E-147 |
| RP11-311C24.1 | -1.028 | 1.37E-108 |
| RP11-161M6.2 | -1.874 | 9.98E-88 |
| RP11-96C23.11 | -1.976 | 1.55E-246 |
| RP11-417E7.2 | 1.554 | 1.08E-108 |
| WFDC21P | 1.09 | 9.56E-43 |
| RP11-6O2.4 | -1.341 | 4.54E-209 |
| RP11-303E16.2 | 1.199 | 4.82E-107 |
| RP11-1000B6.3 | -1.09 | 3.40E-117 |
| EPPK1 | 1.091 | 6.20E-61 |
| RP11-389C8.2 | -1.355 | 5.23E-161 |
| RP11-524D16__A.3 | -1.33 | 1.89E-95 |
| RP11-863P13.3 | 1.106 | 1.30E-93 |
| PECAM1 | -1.599 | 4.69E-115 |
| RP11-388M20.2 | 1.345 | 4.80E-31 |
| TEN1-CDK3 | -1.066 | 7.90E-67 |
| UBE2MP1 | 1.361 | 1.29E-128 |
| RP11-1024P17.1 | -1.566 | 0.00E+00 |
| RP11-448G15.3 | -1.154 | 8.74E-194 |
| SMG1P7 | -1.283 | 4.45E-150 |
| RP11-6O2.3 | -1.041 | 5.64E-121 |
| RP11-554A11.4 | -1.158 | 1.91E-276 |
| TTC39A-AS1 | 1.158 | 5.12E-35 |
| RP11-345J4.5 | 1.261 | 5.92E-151 |
| MIA | -2.322 | 2.70E-12 |
| RP1-302G2.5 | -1.585 | 1.80E-155 |
| RP11-473M20.9 | -1.915 | 1.31E-279 |
| RP11-667K14.4 | 3.233 | 2.44E-87 |
| RP11-498C9.2 | 1.078 | 8.32E-25 |
| RP11-1055B8.4 | -1.308 | 1.64E-62 |
| RP11-294J22.6 | -1.043 | 2.76E-02 |
| MYZAP | -2.325 | 1.20E-179 |
| RP1-59D14.5 | -1.041 | 5.36E-98 |
| TMEM220-AS1 | -1.228 | 4.91E-239 |
| IKBKE | 1.166 | 9.04E-115 |
| RP11-498C9.13 | 2.492 | 1.65E-47 |
| MSMB | 1.463 | 5.94E-23 |
| RP11-927P21.5 | -1.737 | 4.57E-131 |
| RP11-334E6.12 | 1.553 | 4.07E-52 |
| RP11-789C17.5 | -1.449 | 3.95E-75 |
| RP11-159D12.2 | -1.244 | 4.95E-55 |
| MAGOH2P | -1.338 | 6.06E-219 |
| SNRPGP2 | 1.557 | 2.63E-136 |
| RP11-186B7.4 | -1.353 | 2.69E-197 |
| RP13-104F24.3 | -1.718 | 1.78E-128 |
| RP11-138I1.4 | 1.538 | 4.10E-124 |
| RP11-524F11.1 | -1.52 | 4.17E-211 |
| MAFG-AS1 | 1.54 | 7.07E-161 |
| RP11-5A19.5 | -1.304 | 5.36E-110 |
| TXNIP | -2.437 | 4.95E-210 |
| RP11-452I5.2 | 1.492 | 1.79E-54 |
| RP5-890E16.4 | 1.989 | 2.10E-28 |
| GDF10 | -2.534 | 5.19E-273 |
| RP11-20B24.4 | 1.182 | 5.32E-97 |
| MYO15B | -2.77 | 6.22E-157 |
| RP13-104F24.1 | -1.635 | 1.79E-140 |
| RP11-19P22.8 | -1.13 | 6.50E-85 |
| FXYD1 | -4.597 | 2.54E-307 |
| PCAT19 | -1.82 | 5.27E-272 |
| RP11-677O4.6 | -1.753 | 4.51E-109 |
| RP11-78A19.3 | 1.368 | 6.15E-66 |
| SNRPGP4 | -1.316 | 2.89E-85 |
| LCN6 | -2.419 | 2.67E-229 |
| RP11-49K24.6 | 1.209 | 6.52E-21 |
| LINC01140 | -2.188 | 3.95E-177 |
| TBX2-AS1 | -1.865 | 4.95E-185 |
| RP11-793H13.10 | -1.671 | 1.58E-205 |
| SNHG22 | -1.226 | 3.63E-128 |
| RP11-242D8.3 | -1.007 | 4.57E-24 |
| LOC100421166 | -1.096 | 2.77E-267 |
| MIR497HG | -1.961 | 9.51E-203 |
| RP11-322E11.5 | -1.166 | 2.51E-112 |
| LINC01028 | -1.111 | 1.16E-219 |
| RP1-193H18.3 | -2.422 | 1.19E-227 |
| SMIM22 | 2.596 | 4.60E-126 |
| RP5-1057I20.4 | -1.245 | 1.25E-176 |
| SLC6A14 | -1.233 | 8.04E-14 |
| FAM156A | -1.349 | 1.80E-118 |
| TRABD2B | -1.337 | 4.08E-95 |
| FBXO17 | -1.84 | 5.45E-133 |
| PTOV1-AS2 | -1.6 | 1.80E-75 |
| RP11-442H21.2 | 2.014 | 4.54E-20 |
| RP11-932O9.9 | -1.375 | 2.08E-147 |
| RP11-394O4.5 | -3.922 | 1.14e-310 |
| RP5-940J5.9 | 4.191 | 1.14E-48 |
| RP11-540B6.6 | -1.016 | 4.06E-111 |
| MIR222HG | -1.757 | 2.82E-179 |
| MINOS1-NBL1 | 1.334 | 1.08E-118 |
| RP11-544M22.13 | 1.604 | 3.90E-39 |
| HIST2H4B | 1.56 | 1.11E-84 |
| LINC01235 | -1.451 | 3.50E-35 |
| IGHV3-30 | 1.055 | 4.83E-11 |
| PKD1P6 | -1.659 | 3.52E-149 |
| RPS10-NUDT3 | 1.28 | 1.27E-87 |
| HIST2H4A | 1.743 | 3.23E-73 |
| RP11-2E11.9 | -1.17 | 5.56E-175 |
| RP11-171I2.4 | -1.014 | 2.83E-118 |
| SRXN1 | 1.059 | 4.86E-101 |
| MMP28 | -1.741 | 2.53E-139 |
| RP3-368A4.6 | -1.055 | 1.38E-122 |
| LIX1L | -1.037 | 1.66E-100 |
| RP11-787I22.3 | -1.133 | 2.37E-159 |
| RP11-482H16.1 | -1.433 | 7.65E-149 |
| RP4-635E18.8 | -1.805 | 1.15E-181 |
| FGF14-AS2 | -1.57 | 4.60E-174 |
| HIST2H2AA4 | 1.861 | 7.96E-53 |
| RP5-855D21.1 | -1.088 | 5.85E-129 |
| XXbac-BPGBPG55C20.2 | -1.08 | 4.89E-198 |
| RP11-474O21.5 | -1.525 | 7.90E-220 |
| RP11-180N14.1 | -1.139 | 3.81E-107 |
| MUSTN1 | -3.103 | 7.75E-224 |
| GS1-114I9.1 | -1.2 | 6.56E-117 |
| STAG3L5P-PVRIG2P-PILRB | -1.259 | 6.22E-61 |
| RP11-286H15.1 | -2.36 | 5.16E-277 |
| RP1-170O19.23 | -1.183 | 5.37E-165 |
| RP5-855D21.3 | -1.089 | 5.22E-119 |
| RP11-513M16.7 | -1.068 | 2.05E-108 |
| KB-1572G7.2 | -1.153 | 3.61E-130 |
| RP3-508I15.20 | -1.239 | 5.68E-53 |
| RP11-20I20.4 | -1.278 | 2.69E-80 |
| SERPINA3 | 1.391 | 7.86E-20 |
| RP11-386I14.4 | -2.11 | 5.34E-110 |
| RP11-434H6.7 | -1.035 | 1.23E-102 |
| RP11-180C16.1 | -1.002 | 5.99E-44 |
| LL21NC02-21A1.1 | -1.022 | 1.69E-100 |
| HIST1H2BG | 1.58 | 1.03E-29 |
| Metazoa_SRP | 1.561 | 1.09E-63 |
| RP11-823E8.3 | -1.159 | 6.80E-244 |
| RP4-568C11.4 | 1.333 | 2.95E-64 |
| NATD1 | -1.009 | 7.97E-95 |
| SNORA73A | 1.496 | 7.97E-19 |
| RP11-44M6.7 | -1.128 | 3.42E-49 |
| PI4KAP1 | -1.923 | 1.37E-131 |
| RP11-234G16.4 | -1.593 | 2.80E-134 |
| RP11-334C17.6 | 1.692 | 5.91E-13 |
| DLEU2_6 | 2.478 | 4.49E-27 |
| HIST1H2BH | 1.675 | 9.57E-67 |
| RP13-516M14.10 | -1.764 | 2.00E-125 |
| UHRF1 | 3.008 | 8.39E-264 |
| WHAMMP3 | -1.554 | 8.12E-230 |
| RN7SL1 | 1.846 | 5.05E-105 |
| PVT1_1 | -1.037 | 6.27E-19 |
| HERC2P2 | -2.414 | 1.13E-187 |
| RP5-858L17.1 | -1.017 | 1.29E-118 |
| RP11-1000B6.7 | -1.018 | 7.92E-138 |
| HOTAIRM1_2 | -1.438 | 7.21E-100 |
| HIST1H2AE | 1.774 | 3.84E-53 |
| GPIHBP1 | -3.944 | 0.00E+00 |
| PGM5P3-AS1 | -1.522 | 7.08e-315 |
| RP11-295M3.4 | -1.512 | 6.07E-56 |
| RP11-427L15.2 | -1.119 | 1.67E-195 |
| HOTAIRM1_5 | -1.077 | 5.13E-18 |
| TRAC | 1.289 | 6.37E-34 |
| FP325317.1 | -2.527 | 2.30E-297 |
| RP11-681H18.2 | -1.38 | 8.14E-151 |
| uc_338 | -1.536 | 4.61E-35 |
| RDM1 | 1.046 | 1.56E-124 |
| RP11-70D24.2 | -1.022 | 4.86E-239 |
| HIST1H2AM | 1.345 | 6.45E-92 |
| HIST1H3H | 2.172 | 1.30E-102 |
| RP3-414A15.12 | -1.023 | 1.48E-181 |
| RP11-574K11.24 | -1.755 | 2.65E-187 |
| RP11-517I3.2 | -1.518 | 2.30E-219 |
| RP11-175K6.2 | -2.155 | 0.00E+00 |
| RP4-738P15.6 | 1.096 | 5.79E-09 |
| RP11-96D1.8 | -1.188 | 1.10E-190 |
| RP11-58O9.2 | -1.115 | 3.01E-37 |
| RP11-1221G12.2 | -1.618 | 3.78E-206 |
| RP11-87H9.4 | -1.109 | 5.82E-120 |
| RP11-736K20.4 | -2.353 | 0.00E+00 |
| FKSG62 | -1.519 | 7.48E-43 |
| IGHV1-69-2 | 1.15 | 6.05E-16 |
| PAGR1 | 1.021 | 3.31E-114 |
| U91328.1 | 3.058 | 3.57E-13 |
| NPTN-IT1 | -1.005 | 7.18E-173 |
| RBM5-AS1 | -1.048 | 1.15E-143 |
| GIMAP1-GIMAP5 | -1.246 | 1.85E-89 |
| TMEM265 | 1.351 | 1.70E-93 |
| TMBIM4 | -1.726 | 4.16E-13 |
| WASH5P | -1.021 | 1.72E-51 |
| C11orf71 | -1.57 | 2.92E-18 |
