# Supplementary material for: Identification of hub genes and small molecule therapeutic drugs related to breast cancer with comprehensive bioinformatics analysis
Source: PeerJ. 2020 Sep 29;8:e9946. doi: 10.7717/peerj.9946 (PMC7556247; doi:10.7717/peerj.9946)
Supplement: Supplemental Information 16 [file peerj-08-9946-s016.docx]

| **#node1** | **node2** | **coexpression** | **combined_score** |
| --- | --- | --- | --- |
| RACGAP1 | KIF20A | 0.878 | 0.999 |
| GINS1 | GINS2 | 0.932 | 0.999 |
| CDK1 | MAD2L1 | 0.991 | 0.999 |
| CDCA8 | CDC20 | 0.988 | 0.999 |
| CDC20 | CCNB1 | 0.972 | 0.999 |
| CCNB2 | CCNB1 | 0.955 | 0.999 |
| CDK1 | NDC80 | 0.982 | 0.999 |
| MAD2L1 | TRIP13 | 0.891 | 0.999 |
| BUB1B | CCNA2 | 0.934 | 0.999 |
| CDK1 | KIF11 | 0.988 | 0.999 |
| NEK2 | MAD2L1 | 0.8 | 0.999 |
| KIF20A | MAD2L1 | 0.821 | 0.999 |
| CDC20 | CCNB2 | 0.989 | 0.999 |
| CDC20 | UBE2S | 0.573 | 0.999 |
| PTTG1 | CDC20 | 0.931 | 0.999 |
| ZWINT | BUB1B | 0.806 | 0.999 |
| CDC20 | NDC80 | 0.975 | 0.999 |
| CDCA8 | BIRC5 | 0.948 | 0.999 |
| TPX2 | AURKA | 0.952 | 0.999 |
| NUF2 | NDC80 | 0.957 | 0.999 |
| BUB1B | NDC80 | 0.926 | 0.999 |
| DLGAP5 | AURKA | 0.946 | 0.999 |
| CDK1 | UBE2C | 0.978 | 0.999 |
| NEK2 | BUB1B | 0.902 | 0.999 |
| CDC20 | MAD2L1 | 0.978 | 0.999 |
| CDK1 | AURKA | 0.965 | 0.999 |
| MAD2L1 | BUB1B | 0.849 | 0.999 |
| RACGAP1 | ECT2 | 0.868 | 0.999 |
| ZWINT | NDC80 | 0.771 | 0.999 |
| CDC20 | UBE2C | 0.982 | 0.999 |
| RACGAP1 | KIF23 | 0.949 | 0.999 |
| CDK1 | NCAPG | 0.992 | 0.999 |
| CDK1 | CCNB1 | 0.969 | 0.999 |
| CDC20 | AURKA | 0.977 | 0.999 |
| CDK1 | CDC20 | 0.982 | 0.999 |
| MAD2L1 | NDC80 | 0.966 | 0.999 |
| CDK1 | CCNA2 | 0.986 | 0.999 |
| TACC3 | AURKA | 0.694 | 0.999 |
| CKS2 | CCNB1 | 0.878 | 0.999 |
| MAD2L1 | CCNA2 | 0.984 | 0.999 |
| CCNB2 | AURKA | 0.973 | 0.999 |
| CDK1 | BUB1B | 0.94 | 0.999 |
| CDC20 | NEK2 | 0.948 | 0.999 |
| CDC20 | CCNA2 | 0.984 | 0.999 |
| CDC20 | BUB1B | 0.953 | 0.999 |
| CDK1 | CCNB2 | 0.993 | 0.999 |
| CDK1 | CKS2 | 0.965 | 0.999 |
| CDK1 | CDCA8 | 0.971 | 0.998 |
| CCNA2 | CCNB1 | 0.971 | 0.998 |
| CDCA8 | NDC80 | 0.96 | 0.998 |
| BIRC5 | AURKA | 0.869 | 0.998 |
| CDK1 | BIRC5 | 0.967 | 0.998 |
| CDCA8 | CCNB1 | 0.968 | 0.998 |
| UBE2C | CCNA2 | 0.956 | 0.998 |
| ECT2 | KIF23 | 0.869 | 0.998 |
| ZWINT | NUF2 | 0.559 | 0.998 |
| CCNB2 | NDC80 | 0.961 | 0.998 |
| UBE2C | AURKA | 0.949 | 0.998 |
| MAD2L1 | CCNB2 | 0.926 | 0.998 |
| BUB1B | CCNB1 | 0.941 | 0.998 |
| CDCA8 | MAD2L1 | 0.968 | 0.998 |
| CDC20 | BIRC5 | 0.965 | 0.998 |
| MAD2L1 | CCNB1 | 0.955 | 0.998 |
| KIF11 | KIF23 | 0.981 | 0.998 |
| TTK | MAD2L1 | 0.963 | 0.998 |
| CDT1 | CCNA2 | 0.812 | 0.998 |
| UBE2C | CCNB1 | 0.924 | 0.998 |
| CDC20 | KIF2C | 0.958 | 0.998 |
| BIRC5 | MAD2L1 | 0.958 | 0.998 |
| PTTG1 | AURKA | 0.907 | 0.997 |
| PBK | CDK1 | 0.99 | 0.997 |
| CDC20 | TTK | 0.982 | 0.997 |
| HMMR | TPX2 | 0.837 | 0.997 |
| KIF20A | KIF11 | 0.966 | 0.997 |
| KIF2C | NDC80 | 0.917 | 0.997 |
| CDK1 | FOXM1 | 0.84 | 0.997 |
| CDCA8 | KIF2C | 0.931 | 0.997 |
| CDCA8 | CCNB2 | 0.938 | 0.997 |
| PTTG1 | UBE2C | 0.936 | 0.997 |
| NUSAP1 | CDK1 | 0.992 | 0.997 |
| CCNB2 | BUB1B | 0.93 | 0.997 |
| FOXM1 | CCNB1 | 0.811 | 0.997 |
| CDK1 | TPX2 | 0.925 | 0.997 |
| CKS2 | CCNB2 | 0.921 | 0.997 |
| CDCA8 | BUB1B | 0.927 | 0.997 |
| KIF2C | BUB1B | 0.923 | 0.997 |
| UBE2C | BUB1B | 0.903 | 0.997 |
| TOP2A | TPX2 | 0.993 | 0.997 |
| UBE2C | MAD2L1 | 0.919 | 0.997 |
| CDK1 | KIF2C | 0.901 | 0.997 |
| MAD2L1 | NUF2 | 0.93 | 0.997 |
| TYMS | TK1 | 0.787 | 0.997 |
| CDK1 | NUF2 | 0.969 | 0.997 |
| CDK1 | KIF23 | 0.982 | 0.996 |
| CDK1 | DLGAP5 | 0.991 | 0.996 |
| KIF4A | KIF11 | 0.949 | 0.996 |
| CENPF | BUB1B | 0.896 | 0.996 |
| KIF2C | CCNB1 | 0.929 | 0.996 |
| CDC20 | NUF2 | 0.922 | 0.996 |
| TTK | NDC80 | 0.983 | 0.996 |
| RACGAP1 | KIF4A | 0.909 | 0.996 |
| BUB1B | NUF2 | 0.798 | 0.996 |
| CENPF | NDC80 | 0.909 | 0.996 |
| FOXM1 | CCNA2 | 0.863 | 0.996 |
| KIF2C | CCNB2 | 0.914 | 0.996 |
| RACGAP1 | KIF11 | 0.933 | 0.996 |
| KIF20A | CDCA8 | 0.92 | 0.996 |
| NDC80 | CCNB1 | 0.912 | 0.996 |
| TOP2A | CDK1 | 0.986 | 0.996 |
| BIRC5 | CCNB1 | 0.888 | 0.996 |
| BIRC5 | BUB1B | 0.916 | 0.996 |
| DTL | CDT1 | 0.887 | 0.996 |
| BIRC5 | CCNB2 | 0.88 | 0.995 |
| CCNB2 | NCAPG | 0.937 | 0.995 |
| CENPF | NUF2 | 0.892 | 0.995 |
| TTK | BUB1B | 0.91 | 0.995 |
| KIF4A | KIF23 | 0.932 | 0.995 |
| CENPF | CCNB2 | 0.901 | 0.995 |
| ASPM | KIF11 | 0.988 | 0.995 |
| ISG15 | IFI6 | 0.876 | 0.995 |
| KIF2C | CENPF | 0.884 | 0.995 |
| CDC20 | KIF11 | 0.981 | 0.995 |
| CENPU | CENPM | 0.483 | 0.995 |
| KIF20A | KIF4A | 0.936 | 0.995 |
| NDC80 | KIF11 | 0.976 | 0.994 |
| KIF11 | AURKA | 0.94 | 0.994 |
| MAD2L1 | KIF11 | 0.971 | 0.994 |
| CDK1 | PTTG1 | 0.942 | 0.994 |
| KIF18B | KIF2C | 0.827 | 0.994 |
| NEK2 | NDC80 | 0.955 | 0.994 |
| BIRC5 | NDC80 | 0.926 | 0.994 |
| HMMR | AURKA | 0.827 | 0.994 |
| CDCA8 | NUF2 | 0.875 | 0.994 |
| KIF11 | DLGAP5 | 0.971 | 0.994 |
| CDK1 | ASPM | 0.99 | 0.994 |
| CDCA8 | CENPF | 0.89 | 0.994 |
| CENPK | CENPU | 0.369 | 0.994 |
| IGF1 | IGFBP6 | 0 | 0.994 |
| KIF20A | PRC1 | 0.892 | 0.994 |
| KIF2C | KIF11 | 0.921 | 0.994 |
| CDK1 | HMMR | 0.926 | 0.993 |
| CDCA8 | KIF23 | 0.875 | 0.993 |
| KIF20A | KIF2C | 0.915 | 0.993 |
| CDK1 | ECT2 | 0.957 | 0.993 |
| CENPU | NDC80 | 0.787 | 0.993 |
| NEK2 | TPX2 | 0.868 | 0.993 |
| GPIHBP1 | LPL | 0.147 | 0.993 |
| CDK1 | TTK | 0.989 | 0.993 |
| TTK | KIF11 | 0.98 | 0.993 |
| CDK1 | NEK2 | 0.922 | 0.993 |
| CENPF | CCNB1 | 0.849 | 0.993 |
| TENC1 | DLC1 | 0.063 | 0.993 |
| ASPM | NCAPG | 0.991 | 0.993 |
| TPX2 | KIF11 | 0.949 | 0.993 |
| UBE2C | CCNB2 | 0.976 | 0.993 |
| CCNE2 | CDK1 | 0.599 | 0.992 |
| TPX2 | DLGAP5 | 0.965 | 0.992 |
| CKS2 | CCNA2 | 0.838 | 0.992 |
| CDK1 | LMNB1 | 0.619 | 0.992 |
| TTK | NCAPG | 0.988 | 0.992 |
| PRC1 | KIF11 | 0.965 | 0.992 |
| KIF2C | BIRC5 | 0.872 | 0.992 |
| CDC20 | CENPF | 0.839 | 0.992 |
| KIF20A | BIRC5 | 0.875 | 0.992 |
| NUSAP1 | KIF11 | 0.986 | 0.992 |
| CDK1 | CENPF | 0.859 | 0.992 |
| CCNB1 | AURKA | 0.925 | 0.991 |
| CCNB2 | KIF11 | 0.964 | 0.991 |
| CKS2 | CDC20 | 0.909 | 0.991 |
| NUSAP1 | CDC20 | 0.981 | 0.991 |
| LEP | PPARG | 0.061 | 0.991 |
| BUB1B | KIF11 | 0.963 | 0.991 |
| ZWINT | MAD2L1 | 0.813 | 0.991 |
| UBE2T | FANCI | 0.538 | 0.991 |
| KIF11 | NCAPG | 0.985 | 0.991 |
| CDK1 | RRM2 | 0.972 | 0.991 |
| CENPF | FOXM1 | 0.82 | 0.991 |
| CDK1 | KIF20A | 0.963 | 0.991 |
| KIF2C | MAD2L1 | 0.794 | 0.991 |
| TOP2A | UBE2C | 0.982 | 0.991 |
| KIF4A | KIF2C | 0.889 | 0.991 |
| CCNB2 | NUF2 | 0.872 | 0.991 |
| FOXM1 | CCNB2 | 0.819 | 0.991 |
| PBK | TOP2A | 0.982 | 0.991 |
| CDC20 | TRIP13 | 0.898 | 0.991 |
| RACGAP1 | CDCA8 | 0.812 | 0.99 |
| STMN1 | CDK1 | 0.782 | 0.99 |
| CDC20 | DLGAP5 | 0.983 | 0.99 |
| NUSAP1 | DLGAP5 | 0.955 | 0.99 |
| CCNB1 | NCAPG | 0.867 | 0.99 |
| NEK2 | UBE2C | 0.825 | 0.99 |
| CENPF | BIRC5 | 0.809 | 0.99 |
| KIF2C | NUF2 | 0.749 | 0.99 |
| CDK1 | CDT1 | 0.687 | 0.99 |
| NEK2 | AURKA | 0.877 | 0.99 |
| KIF20A | KIF23 | 0.876 | 0.99 |
| BIRC5 | NUF2 | 0.85 | 0.989 |
| CCNA2 | KIF11 | 0.977 | 0.989 |
| NUSAP1 | NCAPG | 0.981 | 0.989 |
| CENPF | MAD2L1 | 0.802 | 0.989 |
| RACGAP1 | KIF2C | 0.794 | 0.989 |
| TTK | NUF2 | 0.927 | 0.989 |
| MAD2L1 | NCAPG | 0.982 | 0.989 |
| CDK1 | CDKN3 | 0.913 | 0.988 |
| TOP2A | NCAPG | 0.967 | 0.988 |
| CXCL11 | CXCL10 | 0.842 | 0.988 |
| CDK1 | ZWINT | 0.843 | 0.988 |
| CDK1 | PRC1 | 0.967 | 0.988 |
| ZWINT | CENPU | 0.795 | 0.988 |
| NUF2 | CCNB1 | 0.814 | 0.988 |
| BUB1B | CENPU | 0.783 | 0.988 |
| TTK | DLGAP5 | 0.979 | 0.988 |
| NUSAP1 | CCNA2 | 0.981 | 0.988 |
| PRC1 | KIF4A | 0.937 | 0.988 |
| NDC80 | AURKA | 0.909 | 0.988 |
| TTK | ASPM | 0.986 | 0.988 |
| ZWINT | CDC20 | 0.771 | 0.988 |
| ANLN | KIF23 | 0.917 | 0.988 |
| CDCA8 | TTK | 0.971 | 0.988 |
| NDC80 | NCAPG | 0.982 | 0.987 |
| HJURP | CENPM | 0.783 | 0.987 |
| ZWINT | CCNB2 | 0.799 | 0.987 |
| TOP2A | DLGAP5 | 0.979 | 0.987 |
| TTK | CCNA2 | 0.975 | 0.987 |
| ASPM | KIF23 | 0.976 | 0.987 |
| NUF2 | KIF11 | 0.947 | 0.987 |
| ZWINT | KIF2C | 0.767 | 0.987 |
| CCNB1 | DLGAP5 | 0.976 | 0.987 |
| CDKN1C | CCNA2 | 0.063 | 0.987 |
| CDC20 | FOXM1 | 0.96 | 0.987 |
| NCAPG | DLGAP5 | 0.975 | 0.986 |
| KIF11 | CCNB1 | 0.939 | 0.986 |
| TOP2A | CDC20 | 0.968 | 0.986 |
| CDK1 | TYMS | 0.981 | 0.986 |
| MAD2L1 | CENPU | 0.825 | 0.986 |
| CDK1 | CENPU | 0.821 | 0.986 |
| ZWINT | CCNB1 | 0.796 | 0.986 |
| TOP2A | CCNB1 | 0.964 | 0.986 |
| PBK | CCNB1 | 0.937 | 0.986 |
| CCNA2 | AURKA | 0.964 | 0.986 |
| ZWINT | BIRC5 | 0.78 | 0.986 |
| CCNB2 | DLGAP5 | 0.976 | 0.986 |
| TOP2A | CCNB2 | 0.965 | 0.986 |
| CDC20 | KIF23 | 0.971 | 0.986 |
| RACGAP1 | CDK1 | 0.955 | 0.986 |
| RACGAP1 | BIRC5 | 0.783 | 0.986 |
| PBK | MAD2L1 | 0.98 | 0.986 |
| CDC20 | CENPM | 0.806 | 0.986 |
| CDCA8 | AURKA | 0.933 | 0.986 |
| CDCA8 | CENPM | 0.809 | 0.985 |
| CCNB2 | CENPM | 0.797 | 0.985 |
| PRC1 | CDC20 | 0.967 | 0.985 |
| RACGAP1 | ANLN | 0.843 | 0.985 |
| KIF18B | KIF20A | 0.831 | 0.985 |
| TOP2A | KIF4A | 0.973 | 0.985 |
| PRC1 | TPX2 | 0.966 | 0.985 |
| ZWINT | CDCA8 | 0.792 | 0.985 |
| CENPK | CENPM | 0.178 | 0.985 |
| ASPM | DLGAP5 | 0.982 | 0.985 |
| TOP2A | KIF11 | 0.968 | 0.985 |
| NUSAP1 | NDC80 | 0.981 | 0.985 |
| ZWINT | CENPF | 0.678 | 0.984 |
| NUSAP1 | TOP2A | 0.966 | 0.984 |
| KIF2C | KIF23 | 0.795 | 0.984 |
| CDCA8 | CCNA2 | 0.974 | 0.984 |
| TOP2A | KIF20A | 0.972 | 0.984 |
| TOP2A | CCNA2 | 0.967 | 0.984 |
| HMMR | NEK2 | 0.796 | 0.984 |
| TOP2A | MKI67 | 0.966 | 0.984 |
| TOP2A | BUB1B | 0.963 | 0.983 |
| NDC80 | DLGAP5 | 0.971 | 0.983 |
| PTTG1 | CCNB1 | 0.929 | 0.983 |
| UBE2C | BIRC5 | 0.956 | 0.983 |
| PLIN1 | PPARG | 0.063 | 0.983 |
| IRS2 | IGF1 | 0 | 0.983 |
| ZWINT | CENPM | 0.682 | 0.983 |
| TOP2A | CENPF | 0.963 | 0.983 |
| UBE2C | UBE2S | 0.703 | 0.983 |
| CDC20 | ASPM | 0.972 | 0.983 |
| BIRC5 | KIF23 | 0.728 | 0.983 |
| CDCA8 | KIF11 | 0.966 | 0.983 |
| BIRC5 | CENPM | 0.797 | 0.983 |
| TTK | CCNB2 | 0.939 | 0.983 |
| TGFBR2 | TGFBR3 | 0.063 | 0.982 |
| CDK1 | MELK | 0.978 | 0.982 |
| CCNB2 | KIF23 | 0.944 | 0.982 |
| RACGAP1 | PRC1 | 0.954 | 0.982 |
| ASPM | CCNA2 | 0.976 | 0.981 |
| MAD2L1 | AURKA | 0.923 | 0.981 |
| PRC1 | NCAPG | 0.969 | 0.981 |
| KIF20A | CCNB1 | 0.956 | 0.981 |
| CAV1 | CAV2 | 0.819 | 0.981 |
| CDK1 | KIF4A | 0.935 | 0.981 |
| NUSAP1 | CDCA8 | 0.966 | 0.981 |
| CENPK | BUB1B | 0.439 | 0.981 |
| LPL | PPARG | 0.062 | 0.98 |
| KIF2C | AURKA | 0.868 | 0.98 |
| KIF20A | DLGAP5 | 0.963 | 0.98 |
| UBE2C | DLGAP5 | 0.969 | 0.98 |
| FN1 | VWF | 0.061 | 0.98 |
| CCNA2 | DLGAP5 | 0.967 | 0.98 |
| NUSAP1 | MKI67 | 0.965 | 0.98 |
| KIF4A | TPX2 | 0.96 | 0.98 |
| ASPM | NDC80 | 0.98 | 0.98 |
| ASPM | CENPF | 0.962 | 0.98 |
| KIF18B | KIF11 | 0.725 | 0.98 |
| CENPU | NUF2 | 0.722 | 0.98 |
| NUSAP1 | ASPM | 0.975 | 0.98 |
| KIF23 | CCNB1 | 0.941 | 0.979 |
| CENPF | CENPU | 0.683 | 0.979 |
| KIF2C | CENPM | 0.75 | 0.979 |
| KIF18B | KIF4A | 0.744 | 0.979 |
| CEP55 | KIF23 | 0.85 | 0.979 |
| CCNB2 | CENPU | 0.758 | 0.979 |
| KIF20A | CCNB2 | 0.952 | 0.979 |
| KIF20A | CDC20 | 0.958 | 0.979 |
| NEK2 | CCNB2 | 0.955 | 0.979 |
| CDC20 | MELK | 0.963 | 0.979 |
| ASPM | MAD2L1 | 0.96 | 0.978 |
| HJURP | CENPU | 0.614 | 0.978 |
| HMMR | DLGAP5 | 0.956 | 0.978 |
| CENPK | NUF2 | 0.645 | 0.978 |
| PRC1 | KIF23 | 0.882 | 0.978 |
| NUSAP1 | CCNB2 | 0.956 | 0.978 |
| CCNA2 | NDC80 | 0.97 | 0.978 |
| UBE2T | UBE2C | 0.733 | 0.978 |
| PBK | MELK | 0.951 | 0.978 |
| CKS2 | MAD2L1 | 0.957 | 0.978 |
| CDK1 | CENPM | 0.759 | 0.978 |
| CENPU | CCNB1 | 0.723 | 0.977 |
| BUB1B | DLGAP5 | 0.945 | 0.977 |
| KIF20A | TPX2 | 0.959 | 0.977 |
| SPP1 | FN1 | 0.102 | 0.977 |
| KIF2C | CENPU | 0.682 | 0.977 |
| CDCA3 | CDC20 | 0.942 | 0.977 |
| TOP2A | RRM2 | 0.939 | 0.977 |
| KIF2C | TPX2 | 0.942 | 0.977 |
| CCNB2 | CCNA2 | 0.97 | 0.977 |
| NUSAP1 | PRC1 | 0.962 | 0.977 |
| LAMA2 | DMD | 0.084 | 0.977 |
| PRC1 | CCNB1 | 0.941 | 0.977 |
| NUSAP1 | CEP55 | 0.957 | 0.976 |
| PBK | CDC20 | 0.964 | 0.976 |
| HOXA7 | HOXA5 | 0.356 | 0.976 |
| PRC1 | ASPM | 0.965 | 0.976 |
| CXCL12 | CXCL10 | 0 | 0.976 |
| NUSAP1 | MAD2L1 | 0.962 | 0.976 |
| CDCA8 | UBE2C | 0.961 | 0.976 |
| UBE2C | TPX2 | 0.95 | 0.976 |
| TOP2A | AURKA | 0.92 | 0.976 |
| CEP55 | KIF11 | 0.957 | 0.975 |
| PTTG1 | BUB1B | 0.821 | 0.975 |
| CENPK | NDC80 | 0.596 | 0.975 |
| PBK | DLGAP5 | 0.957 | 0.975 |
| CXCL12 | CXCL11 | 0 | 0.975 |
| TOP2A | TTK | 0.954 | 0.974 |
| KIF23 | AURKA | 0.919 | 0.974 |
| CENPK | CDK1 | 0.716 | 0.974 |
| KIF20A | AURKA | 0.94 | 0.974 |
| PTTG1 | CCNB2 | 0.936 | 0.974 |
| CENPF | CENPM | 0.575 | 0.974 |
| CCNB1 | CENPM | 0.715 | 0.974 |
| CDC20 | TACC3 | 0.964 | 0.974 |
| CENPK | CENPF | 0.545 | 0.974 |
| PBK | BIRC5 | 0.952 | 0.974 |
| TOP2A | ASPM | 0.967 | 0.973 |
| NUSAP1 | CENPF | 0.95 | 0.973 |
| ADH1C | ADH1B | 0.179 | 0.973 |
| ANK2 | SCN4B | 0.192 | 0.973 |
| UBE2C | TYMS | 0.796 | 0.972 |
| TPX2 | CCNB1 | 0.946 | 0.972 |
| BUB1B | CENPM | 0.626 | 0.972 |
| CENPK | MAD2L1 | 0.696 | 0.972 |
| NUF2 | NCAPG | 0.953 | 0.972 |
| MKI67 | KIF11 | 0.959 | 0.972 |
| NUF2 | DLGAP5 | 0.94 | 0.971 |
| CDCA3 | CDK1 | 0.942 | 0.971 |
| FAM83D | HMMR | 0.558 | 0.971 |
| CDCA8 | DLGAP5 | 0.95 | 0.971 |
| CDC20 | MKI67 | 0.94 | 0.971 |
| CDK1 | CEP55 | 0.933 | 0.971 |
| PBK | TTK | 0.947 | 0.971 |
| CDK1 | TACC3 | 0.958 | 0.971 |
| RRM2 | BUB1B | 0.948 | 0.971 |
| NDC80 | KIF23 | 0.962 | 0.971 |
| KIF20A | BUB1B | 0.935 | 0.971 |
| NUSAP1 | UBE2C | 0.935 | 0.97 |
| ASPM | CCNB1 | 0.95 | 0.97 |
| RRM2 | TYMS | 0.923 | 0.97 |
| NUSAP1 | KIF23 | 0.959 | 0.97 |
| MKI67 | DLGAP5 | 0.925 | 0.97 |
| KIF4A | ASPM | 0.945 | 0.97 |
| CDC20 | CDT1 | 0.923 | 0.97 |
| TOP2A | NDC80 | 0.965 | 0.97 |
| RRM2 | KIF11 | 0.934 | 0.97 |
| NDC80 | CENPM | 0.611 | 0.969 |
| CCNA2 | NCAPG | 0.958 | 0.969 |
| CDCA8 | ASPM | 0.966 | 0.969 |
| PBK | BUB1B | 0.942 | 0.969 |
| NUSAP1 | TPX2 | 0.913 | 0.968 |
| KIF20A | CCNA2 | 0.938 | 0.968 |
| CDC20 | RRM2 | 0.927 | 0.968 |
| MELK | CCNB2 | 0.919 | 0.968 |
| CCNA2 | KIF23 | 0.939 | 0.968 |
| ASPM | TPX2 | 0.966 | 0.968 |
| FN1 | IGF1 | 0.062 | 0.968 |
| ASPM | BUB1B | 0.923 | 0.968 |
| PBK | CCNB2 | 0.951 | 0.968 |
| KIF4A | BUB1B | 0.932 | 0.967 |
| CEP55 | CENPF | 0.934 | 0.967 |
| MAD2L1 | CENPM | 0.614 | 0.967 |
| KIF23 | DLGAP5 | 0.951 | 0.967 |
| CDK1 | TRIP13 | 0.959 | 0.967 |
| RMI2 | RAD51AP1 | 0.567 | 0.967 |
| BUB1B | AURKA | 0.844 | 0.967 |
| MELK | DLGAP5 | 0.943 | 0.966 |
| BIRC5 | KIF11 | 0.94 | 0.966 |
| CDC20 | CENPU | 0.551 | 0.966 |
| RACGAP1 | CEP55 | 0.858 | 0.966 |
| IRS2 | LEP | 0.061 | 0.966 |
| KIF20A | NCAPG | 0.948 | 0.966 |
| TOP2A | KIF23 | 0.94 | 0.966 |
| KIF18B | KIF23 | 0.594 | 0.966 |
| KIF20A | CEP55 | 0.915 | 0.966 |
| CDCA3 | CDCA8 | 0.924 | 0.966 |
| NUSAP1 | KIF20A | 0.94 | 0.965 |
| LMNB1 | CCNB1 | 0.47 | 0.965 |
| TPX2 | MELK | 0.943 | 0.965 |
| BIRC5 | CCNA2 | 0.921 | 0.965 |
| KIF20A | UBE2C | 0.937 | 0.965 |
| CENPF | KIF11 | 0.926 | 0.965 |
| CDC20 | NCAPG | 0.94 | 0.965 |
| RACGAP1 | CCNB1 | 0.937 | 0.965 |
| NEK2 | KIF11 | 0.921 | 0.965 |
| KIF4A | CCNB2 | 0.924 | 0.965 |
| CEP55 | DLGAP5 | 0.937 | 0.965 |
| NEK2 | CCNA2 | 0.919 | 0.965 |
| BIRC5 | CENPU | 0.609 | 0.964 |
| KIF4A | DLGAP5 | 0.939 | 0.964 |
| PBK | KIF11 | 0.939 | 0.964 |
| KIF2C | CCNA2 | 0.932 | 0.964 |
| MELK | CCNB1 | 0.92 | 0.964 |
| TACC3 | KIF11 | 0.913 | 0.964 |
| CDKN3 | CCNA2 | 0.887 | 0.964 |
| TOP2A | MELK | 0.935 | 0.964 |
| TTK | CCNB1 | 0.874 | 0.964 |
| NUSAP1 | PBK | 0.937 | 0.963 |
| NUSAP1 | AURKA | 0.94 | 0.963 |
| TOP2A | CEP55 | 0.937 | 0.963 |
| KIF4A | CDC20 | 0.937 | 0.963 |
| PBK | CCNA2 | 0.946 | 0.963 |
| TPX2 | NCAPG | 0.942 | 0.963 |
| NUSAP1 | TTK | 0.939 | 0.963 |
| CENPF | DLGAP5 | 0.932 | 0.963 |
| CDCA8 | CENPU | 0.573 | 0.963 |
| NEK2 | CENPF | 0.893 | 0.963 |
| TTK | CENPF | 0.932 | 0.962 |
| HJURP | CENPK | 0.285 | 0.962 |
| BIRC5 | TPX2 | 0.932 | 0.962 |
| CCNE2 | CCNA2 | 0.431 | 0.962 |
| FOXM1 | MELK | 0.816 | 0.962 |
| CEP55 | TTK | 0.942 | 0.962 |
| NUSAP1 | RRM2 | 0.924 | 0.962 |
| TOP2A | KIF2C | 0.94 | 0.962 |
| MKI67 | CENPF | 0.931 | 0.962 |
| KIF4A | AURKA | 0.921 | 0.962 |
| PRC1 | MAD2L1 | 0.893 | 0.962 |
| KIF18B | RACGAP1 | 0.426 | 0.962 |
| PRC1 | BUB1B | 0.928 | 0.962 |
| CDCA8 | TPX2 | 0.926 | 0.961 |
| PBK | KIF20A | 0.937 | 0.961 |
| CENPK | ZWINT | 0.28 | 0.961 |
| TPX2 | CCNB2 | 0.935 | 0.961 |
| NUSAP1 | BUB1B | 0.924 | 0.961 |
| MKI67 | FOXM1 | 0.937 | 0.961 |
| NUSAP1 | NUF2 | 0.94 | 0.961 |
| MMP9 | MMP1 | 0.518 | 0.961 |
| CDCA3 | CCNB2 | 0.926 | 0.961 |
| PTTG1 | CCNA2 | 0.896 | 0.961 |
| RRM2 | CCNA2 | 0.927 | 0.961 |
| RRM2 | MELK | 0.925 | 0.96 |
| CDCA3 | PBK | 0.938 | 0.96 |
| CCNE2 | CDKN1C | 0.063 | 0.96 |
| TOP2A | CDCA8 | 0.935 | 0.96 |
| PBK | NDC80 | 0.956 | 0.96 |
| KIF20A | CENPF | 0.927 | 0.96 |
| MELK | CCNA2 | 0.93 | 0.96 |
| PBK | UBE2C | 0.938 | 0.959 |
| UBE2C | KIF11 | 0.936 | 0.959 |
| CDCA3 | TOP2A | 0.943 | 0.959 |
| MELK | KIF23 | 0.943 | 0.959 |
| PTTG1 | DLGAP5 | 0.923 | 0.959 |
| PRC1 | CCNB2 | 0.9 | 0.959 |
| NEK2 | DLGAP5 | 0.934 | 0.959 |
| KIF20A | NDC80 | 0.934 | 0.959 |
| PBK | NCAPG | 0.937 | 0.959 |
| KIF20A | NEK2 | 0.923 | 0.959 |
| ECT2 | ASPM | 0.942 | 0.958 |
| PRC1 | TTK | 0.947 | 0.958 |
| RRM2 | CCNB2 | 0.909 | 0.958 |
| SHCBP1 | KIF23 | 0.717 | 0.958 |
| TYMS | NCAPG | 0.95 | 0.958 |
| ASPM | AURKA | 0.898 | 0.958 |
| RACGAP1 | CCNB2 | 0.934 | 0.958 |
| CEP55 | MKI67 | 0.916 | 0.958 |
| CDCA3 | UBE2C | 0.929 | 0.957 |
| CDCA8 | NEK2 | 0.915 | 0.957 |
| CENPK | BIRC5 | 0.521 | 0.957 |
| RRM2 | UBE2C | 0.92 | 0.957 |
| CDK1 | MKI67 | 0.841 | 0.957 |
| ECT2 | CDC20 | 0.938 | 0.957 |
| KIF4A | CCNA2 | 0.924 | 0.957 |
| CEP55 | UBE2C | 0.911 | 0.957 |
| MMP9 | CTSG | 0.089 | 0.957 |
| CDC20 | TPX2 | 0.922 | 0.957 |
| PRC1 | ECT2 | 0.916 | 0.957 |
| PBK | CEP55 | 0.924 | 0.956 |
| MAD2L1 | DLGAP5 | 0.931 | 0.956 |
| TPX2 | CCNA2 | 0.927 | 0.956 |
| CENPK | CDCA8 | 0.378 | 0.956 |
| TPX2 | NDC80 | 0.927 | 0.956 |
| CEP55 | RRM2 | 0.92 | 0.956 |
| KIF2C | DLGAP5 | 0.903 | 0.956 |
| NUSAP1 | TACC3 | 0.939 | 0.956 |
| RACGAP1 | BUB1B | 0.925 | 0.956 |
| GINS2 | NCAPG | 0.93 | 0.956 |
| CDK1 | EZH2 | 0.682 | 0.956 |
| FOXM1 | AURKA | 0.818 | 0.955 |
| NEK2 | CCNB1 | 0.899 | 0.955 |
| CENPF | NCAPG | 0.922 | 0.955 |
| PRC1 | AURKA | 0.927 | 0.955 |
| ANLN | KIF11 | 0.91 | 0.955 |
| KIF2C | UBE2C | 0.919 | 0.955 |
| TOP2A | NEK2 | 0.923 | 0.955 |
| HJURP | KIF11 | 0.945 | 0.955 |
| KIF20A | TTK | 0.922 | 0.955 |
| ASPM | TACC3 | 0.914 | 0.955 |
| MKI67 | CCNA2 | 0.922 | 0.955 |
| KIF2C | SPAG5 | 0.919 | 0.955 |
| STMN1 | CCNB1 | 0.331 | 0.954 |
| RRM2 | CCNB1 | 0.899 | 0.954 |
| NUSAP1 | BIRC5 | 0.91 | 0.954 |
| CEP55 | CCNA2 | 0.942 | 0.954 |
| KIF20A | ECT2 | 0.866 | 0.954 |
| PRC1 | CCNA2 | 0.928 | 0.954 |
| PBK | RRM2 | 0.936 | 0.954 |
| CD36 | PPARG | 0.063 | 0.954 |
| HMMR | LYVE1 | 0 | 0.954 |
| CEP55 | CCNB1 | 0.867 | 0.954 |
| CDKN3 | CCNB1 | 0.868 | 0.954 |
| CEP55 | BUB1B | 0.921 | 0.954 |
| HJURP | CCNA2 | 0.904 | 0.953 |
| MKI67 | RRM2 | 0.923 | 0.953 |
| MELK | NCAPG | 0.925 | 0.953 |
| CDCA8 | NCAPG | 0.932 | 0.953 |
| PTTG1 | CDCA8 | 0.905 | 0.953 |
| STIL | ASPM | 0.778 | 0.953 |
| SPAG5 | KIF11 | 0.92 | 0.952 |
| NUSAP1 | KIF2C | 0.905 | 0.952 |
| ECT2 | CCNA2 | 0.936 | 0.952 |
| CEP55 | ASPM | 0.932 | 0.952 |
| PBK | TPX2 | 0.927 | 0.952 |
| SPAG5 | DLGAP5 | 0.914 | 0.952 |
| SPAG5 | BUB1B | 0.923 | 0.952 |
| CIDEC | CIDEA | 0.171 | 0.952 |
| EZH2 | PPARG | 0 | 0.951 |
| KIF20A | ASPM | 0.914 | 0.951 |
| TTK | MKI67 | 0.937 | 0.951 |
| KIF2C | TTK | 0.911 | 0.951 |
| MKI67 | BUB1B | 0.92 | 0.951 |
| IGF1 | VWF | 0.1 | 0.951 |
| TTK | AURKA | 0.914 | 0.951 |
| PDGFD | PDGFRA | 0.084 | 0.951 |
| PBK | CENPF | 0.917 | 0.95 |
| TOP2A | BIRC5 | 0.883 | 0.95 |
| HMMR | ASPM | 0.931 | 0.95 |
| SPAG5 | CCNB2 | 0.928 | 0.95 |
| TTK | BIRC5 | 0.918 | 0.95 |
| CDC20 | CEP55 | 0.898 | 0.95 |
| KIF4A | MELK | 0.926 | 0.95 |
| PBK | NUF2 | 0.919 | 0.949 |
| TPX2 | BUB1B | 0.902 | 0.949 |
| RRM2 | DLGAP5 | 0.913 | 0.949 |
| ASPM | MELK | 0.923 | 0.949 |
| CENPF | CCNA2 | 0.908 | 0.949 |
| TPX2 | NUF2 | 0.899 | 0.949 |
| NCAPG | AURKA | 0.898 | 0.949 |
| BIRC5 | DLGAP5 | 0.913 | 0.949 |
| CDC20 | SPAG5 | 0.938 | 0.949 |
| ECT2 | KIF11 | 0.921 | 0.948 |
| KIF4A | BIRC5 | 0.907 | 0.948 |
| CENPF | RRM2 | 0.915 | 0.948 |
| KIF20A | PTTG1 | 0.917 | 0.948 |
| KIF20A | RRM2 | 0.927 | 0.948 |
| MAD2L1 | KIF23 | 0.894 | 0.948 |
| UBE2C | FOXM1 | 0.83 | 0.948 |
| GPD1 | GPAM | 0.063 | 0.948 |
| CDCA3 | ASPM | 0.93 | 0.948 |
| DEPDC1 | TOP2A | 0.94 | 0.947 |
| PBK | ASPM | 0.925 | 0.947 |
| CDCA8 | MELK | 0.91 | 0.947 |
| KIF2C | NEK2 | 0.896 | 0.947 |
| DEPDC1 | DLGAP5 | 0.923 | 0.947 |
| KIF20A | FOXM1 | 0.844 | 0.947 |
| MELK | BUB1B | 0.907 | 0.947 |
| NUF2 | CENPM | 0.349 | 0.947 |
| SPAG5 | AURKA | 0.818 | 0.947 |
| CENPK | CCNB1 | 0.482 | 0.946 |
| ECT2 | MELK | 0.924 | 0.946 |
| ASPM | CCNB2 | 0.909 | 0.946 |
| CDCA8 | SPAG5 | 0.915 | 0.946 |
| KIF2C | CEP55 | 0.905 | 0.946 |
| CDCA8 | CEP55 | 0.912 | 0.946 |
| PTTG1 | TPX2 | 0.913 | 0.946 |
| CENPK | CDC20 | 0.377 | 0.946 |
| PTTG1 | BIRC5 | 0.902 | 0.946 |
| CKS2 | TTK | 0.868 | 0.946 |
| CEP55 | NCAPG | 0.923 | 0.945 |
| CDKN3 | AURKA | 0.901 | 0.945 |
| CENPK | CCNB2 | 0.402 | 0.945 |
| MMP1 | IGF1 | 0 | 0.945 |
| DEPDC1 | KIF11 | 0.939 | 0.945 |
| RRM2 | NCAPG | 0.925 | 0.945 |
| KIF4A | NCAPG | 0.89 | 0.945 |
| TACC3 | NCAPG | 0.941 | 0.944 |
| KIF4A | CCNB1 | 0.888 | 0.944 |
| CENPF | TPX2 | 0.891 | 0.944 |
| DEPDC1 | CENPF | 0.919 | 0.944 |
| BIRC5 | NCAPG | 0.891 | 0.944 |
| TOP2A | FOXM1 | 0.844 | 0.944 |
| CAV1 | PLIN1 | 0 | 0.944 |
| E2F8 | CCNA2 | 0.82 | 0.944 |
| PRC1 | NDC80 | 0.934 | 0.944 |
| TTK | TPX2 | 0.908 | 0.944 |
| NUSAP1 | NEK2 | 0.922 | 0.944 |
| SPRY1 | SPRY2 | 0.088 | 0.943 |
| KIF20A | ANLN | 0.847 | 0.943 |
| TOP2A | TYMS | 0.83 | 0.943 |
| MAOA | AOC3 | 0.082 | 0.943 |
| TOP2A | MAD2L1 | 0.891 | 0.943 |
| CEP55 | MELK | 0.909 | 0.942 |
| KIF4A | NDC80 | 0.895 | 0.942 |
| KIF2C | ASPM | 0.915 | 0.942 |
| NUSAP1 | HJURP | 0.904 | 0.942 |
| CDCA3 | KIF20A | 0.896 | 0.942 |
| NEK2 | BIRC5 | 0.893 | 0.942 |
| TOP2A | PRC1 | 0.915 | 0.942 |
| CENPK | KIF2C | 0.321 | 0.942 |
| PBK | CDCA8 | 0.915 | 0.941 |
| KIF4A | UBE2C | 0.905 | 0.941 |
| TOP2A | SPAG5 | 0.929 | 0.941 |
| RRM2 | TPX2 | 0.923 | 0.941 |
| NUSAP1 | SPAG5 | 0.902 | 0.941 |
| MKI67 | ASPM | 0.927 | 0.941 |
| CKS2 | NCAPG | 0.918 | 0.941 |
| HJURP | NDC80 | 0.916 | 0.941 |
| BUB1B | KIF23 | 0.891 | 0.94 |
| CEP55 | FOXM1 | 0.826 | 0.94 |
| MELK | KIF11 | 0.907 | 0.94 |
| PBK | KIF2C | 0.913 | 0.94 |
| KIF4A | CDCA8 | 0.889 | 0.94 |
| CAV1 | PDGFRA | 0.095 | 0.939 |
| TACC3 | MAD2L1 | 0.932 | 0.939 |
| CDCA8 | MKI67 | 0.922 | 0.939 |
| DLC1 | TNS1 | 0.108 | 0.939 |
| TYMS | MAD2L1 | 0.922 | 0.939 |
| BIRC5 | MELK | 0.896 | 0.939 |
| TK1 | BIRC5 | 0.892 | 0.939 |
| PTTG1 | CENPF | 0.856 | 0.939 |
| CDCA8 | RRM2 | 0.918 | 0.939 |
| MELK | NDC80 | 0.937 | 0.939 |
| MAOA | ADH1B | 0.103 | 0.938 |
| RRM2 | BIRC5 | 0.884 | 0.938 |
| UBE2C | SPAG5 | 0.924 | 0.938 |
| TTK | RRM2 | 0.917 | 0.938 |
| CCNE2 | AURKA | 0.275 | 0.938 |
| CDCA3 | BIRC5 | 0.902 | 0.938 |
| FOXM1 | BUB1B | 0.816 | 0.938 |
| SPP1 | GPC3 | 0.069 | 0.938 |
| CEP55 | SPAG5 | 0.913 | 0.938 |
| KIF20A | SPAG5 | 0.92 | 0.937 |
| RACGAP1 | AURKA | 0.874 | 0.937 |
| TYMS | NDC80 | 0.921 | 0.937 |
| SHCBP1 | NCAPG | 0.936 | 0.937 |
| UBE2C | MELK | 0.89 | 0.937 |
| SPAG5 | NDC80 | 0.908 | 0.937 |
| HMMR | NCAPG | 0.929 | 0.936 |
| MMP1 | CTSG | 0 | 0.936 |
| CDK1 | GINS2 | 0.921 | 0.936 |
| RRM2 | MAD2L1 | 0.888 | 0.936 |
| PBK | HMMR | 0.911 | 0.936 |
| MELK | MAD2L1 | 0.901 | 0.936 |
| ASPM | CDKN3 | 0.897 | 0.936 |
| NUSAP1 | MELK | 0.873 | 0.936 |
| CDK1 | TK1 | 0.781 | 0.936 |
| PTTG1 | NCAPG | 0.902 | 0.936 |
| PTTG1 | MAD2L1 | 0.832 | 0.936 |
| TACC3 | CCNA2 | 0.925 | 0.936 |
| COL11A1 | COL10A1 | 0.201 | 0.935 |
| SPAG5 | CCNA2 | 0.922 | 0.935 |
| FOXM1 | TPX2 | 0.903 | 0.935 |
| PBK | PTTG1 | 0.908 | 0.935 |
| E2F8 | RRM2 | 0.904 | 0.934 |
| GPC3 | FN1 | 0.078 | 0.934 |
| SHCBP1 | CCNA2 | 0.929 | 0.934 |
| CDKN1C | CDK1 | 0.062 | 0.934 |
| TPX2 | KIF23 | 0.87 | 0.934 |
| DEPDC1 | BUB1B | 0.916 | 0.934 |
| SPAG5 | BIRC5 | 0.903 | 0.934 |
| ECT2 | ANLN | 0.643 | 0.934 |
| KIF2C | RRM2 | 0.915 | 0.934 |
| PROS1 | FN1 | 0.114 | 0.933 |
| TTK | KIF23 | 0.893 | 0.933 |
| CDCA8 | FOXM1 | 0.907 | 0.933 |
| CIDEC | PLIN1 | 0.229 | 0.933 |
| DTL | NCAPG | 0.93 | 0.933 |
| E2F8 | BUB1B | 0.914 | 0.933 |
| UBE2T | UBE2S | 0.27 | 0.933 |
| NUSAP1 | CCNB1 | 0.883 | 0.933 |
| TGFBR2 | CAV1 | 0.125 | 0.933 |
| E2F8 | CDCA8 | 0.869 | 0.932 |
| MKI67 | BIRC5 | 0.841 | 0.932 |
| KIF26B | KIF20A | 0 | 0.932 |
| NUSAP1 | KIF4A | 0.861 | 0.932 |
| RACGAP1 | DLGAP5 | 0.887 | 0.932 |
| KIF4A | NEK2 | 0.9 | 0.932 |
| CEP55 | NDC80 | 0.91 | 0.932 |
| ZBTB16 | UBE2S | 0 | 0.932 |
| HJURP | DLGAP5 | 0.894 | 0.932 |
| ASPM | SPAG5 | 0.916 | 0.932 |
| KIF2C | FOXM1 | 0.895 | 0.932 |
| PBK | KIF4A | 0.896 | 0.932 |
| CDK1 | ANLN | 0.864 | 0.931 |
| CCNE2 | CCNB2 | 0.354 | 0.931 |
| CKS2 | UBE2C | 0.867 | 0.931 |
| ECT2 | AURKA | 0.872 | 0.93 |
| CDCA8 | TACC3 | 0.909 | 0.93 |
| ANLN | DLGAP5 | 0.909 | 0.93 |
| TTK | MELK | 0.914 | 0.93 |
| CDCA3 | KIF2C | 0.9 | 0.93 |
| HJURP | KIF20A | 0.917 | 0.93 |
| KIF20A | MKI67 | 0.91 | 0.929 |
| GHR | IRS2 | 0 | 0.929 |
| KIF20A | MELK | 0.909 | 0.929 |
| RACGAP1 | MELK | 0.88 | 0.929 |
| CDCA3 | DLGAP5 | 0.892 | 0.929 |
| HJURP | NEK2 | 0.913 | 0.928 |
| CDKN3 | MAD2L1 | 0.847 | 0.928 |
| KIF2C | MKI67 | 0.904 | 0.928 |
| E2F8 | DLGAP5 | 0.915 | 0.928 |
| ASPM | UBE2C | 0.898 | 0.928 |
| HMMR | CEP55 | 0.907 | 0.928 |
| PTTG1 | CDKN3 | 0.907 | 0.928 |
| MELK | AURKA | 0.917 | 0.928 |
| HJURP | CDK1 | 0.836 | 0.928 |
| PTTG1 | KIF2C | 0.872 | 0.928 |
| PROS1 | VWF | 0.061 | 0.927 |
| E2F8 | TOP2A | 0.91 | 0.927 |
| NEK2 | SPAG5 | 0.903 | 0.927 |
| RACGAP1 | MAD2L1 | 0.902 | 0.927 |
| KIF4A | SPAG5 | 0.896 | 0.927 |
| NUSAP1 | DEPDC1 | 0.884 | 0.927 |
| CDCA3 | CCNA2 | 0.881 | 0.927 |
| NUSAP1 | CDCA3 | 0.884 | 0.926 |
| KIF23 | NCAPG | 0.891 | 0.926 |
| PBK | SPAG5 | 0.913 | 0.926 |
| CXCL12 | ADRB2 | 0 | 0.926 |
| DEPDC1 | ASPM | 0.9 | 0.926 |
| HMMR | CENPF | 0.88 | 0.926 |
| ZWINT | LMNB1 | 0.346 | 0.926 |
| TOP2A | PTTG1 | 0.854 | 0.925 |
| BUB1B | NCAPG | 0.899 | 0.925 |
| CFD | IGF1 | 0.064 | 0.925 |
| KIF26B | KIF23 | 0 | 0.925 |
| KIF20A | NUF2 | 0.825 | 0.925 |
| HJURP | MKI67 | 0.919 | 0.925 |
| TTK | SPAG5 | 0.913 | 0.925 |
| EBF1 | PPARG | 0 | 0.925 |
| CCNA2 | TRIP13 | 0.905 | 0.925 |
| DTL | RRM2 | 0.855 | 0.924 |
| CDCA8 | SHCBP1 | 0.855 | 0.924 |
| CDK1 | SPAG5 | 0.846 | 0.924 |
| CENPF | MELK | 0.874 | 0.924 |
| TOP2A | RACGAP1 | 0.887 | 0.924 |
| CDCA3 | KIF11 | 0.879 | 0.924 |
| CDT1 | CCNB1 | 0.746 | 0.924 |
| CENPF | SPAG5 | 0.886 | 0.923 |
| KIF2C | NCAPG | 0.884 | 0.923 |
| NUSAP1 | PTTG1 | 0.859 | 0.923 |
| PTTG1 | HMMR | 0.9 | 0.923 |
| CENPF | UBE2C | 0.85 | 0.923 |
| HJURP | TOP2A | 0.901 | 0.923 |
| NUSAP1 | HMMR | 0.834 | 0.923 |
| NEK2 | RRM2 | 0.882 | 0.923 |
| ASPM | NEK2 | 0.91 | 0.922 |
| HJURP | UBE2C | 0.89 | 0.922 |
| IGF1 | FGF2 | 0 | 0.922 |
| PBK | KIF23 | 0.902 | 0.922 |
| UHRF1 | TOP2A | 0.773 | 0.922 |
| CDCA3 | BUB1B | 0.877 | 0.922 |
| UHRF1 | CDC20 | 0.894 | 0.921 |
| PBK | CDKN3 | 0.904 | 0.92 |
| DEPDC1 | KIF20A | 0.854 | 0.92 |
| DEPDC1 | CEP55 | 0.86 | 0.92 |
| HJURP | CENPF | 0.893 | 0.92 |
| ECT2 | NDC80 | 0.902 | 0.92 |
| RACGAP1 | CDC20 | 0.882 | 0.92 |
| ASPM | DTL | 0.92 | 0.92 |
| UBE2C | NDC80 | 0.885 | 0.92 |
| CDT1 | CCNB2 | 0.867 | 0.92 |
| LEP | IGF1 | 0 | 0.92 |
| MKI67 | CCNB2 | 0.859 | 0.92 |
| ASPM | NUF2 | 0.897 | 0.92 |
| TPX2 | MAD2L1 | 0.841 | 0.92 |
| CDKN3 | DLGAP5 | 0.913 | 0.919 |
| CKS2 | ASPM | 0.917 | 0.919 |
| RRM2 | NDC80 | 0.904 | 0.919 |
| CDKN3 | CCNB2 | 0.857 | 0.919 |
| BUB1B | TRIP13 | 0.805 | 0.919 |
| UBE2C | ZBTB16 | 0 | 0.919 |
| RACGAP1 | KIF26B | 0.075 | 0.919 |
| CDCA3 | KIF4A | 0.881 | 0.919 |
| HJURP | FOXM1 | 0.897 | 0.919 |
| CEP55 | NEK2 | 0.87 | 0.919 |
| CDKN3 | MELK | 0.9 | 0.919 |
| PRC1 | UBE2C | 0.899 | 0.919 |
| EDNRB | GNG11 | 0.069 | 0.918 |
| NUSAP1 | ECT2 | 0.891 | 0.918 |
| NUSAP1 | FOXM1 | 0.888 | 0.918 |
| ADAMTS5 | ADAMTS1 | 0.157 | 0.918 |
| ASPM | RRM2 | 0.913 | 0.918 |
| TYMS | RNASEH2A | 0.877 | 0.918 |
| CCNA2 | NUF2 | 0.879 | 0.918 |
| TOP2A | NUF2 | 0.852 | 0.918 |
| TACC3 | DLGAP5 | 0.701 | 0.917 |
| RACGAP1 | SHCBP1 | 0.657 | 0.917 |
| KIF20A | SHCBP1 | 0.901 | 0.917 |
| CDCA3 | TTK | 0.888 | 0.917 |
| TOP2A | HMMR | 0.849 | 0.917 |
| E2F8 | KIF11 | 0.903 | 0.917 |
| TOP2A | ECT2 | 0.884 | 0.917 |
| KIF26B | KIF11 | 0 | 0.917 |
| PBK | AURKA | 0.877 | 0.917 |
| MAOA | INMT | 0.061 | 0.917 |
| HJURP | KIF2C | 0.888 | 0.917 |
| IGF1 | CTSG | 0 | 0.916 |
| HJURP | CCNB2 | 0.9 | 0.916 |
| PBK | RACGAP1 | 0.898 | 0.916 |
| ASPM | BIRC5 | 0.892 | 0.916 |
| TTK | TACC3 | 0.902 | 0.916 |
| KIF18B | CDC20 | 0.895 | 0.916 |
| CDC20 | TYMS | 0.872 | 0.915 |
| PROS1 | IGF1 | 0.062 | 0.915 |
| CDC20 | GINS2 | 0.869 | 0.915 |
| HJURP | CDC20 | 0.889 | 0.915 |
| CEP55 | ANLN | 0.832 | 0.915 |
| CFD | VWF | 0.077 | 0.915 |
| RRM2 | SPAG5 | 0.915 | 0.915 |
| EBF1 | LEP | 0 | 0.915 |
| RACGAP1 | CCNA2 | 0.878 | 0.914 |
| KIF4A | CEP55 | 0.825 | 0.914 |
| UHRF1 | CCNA2 | 0.877 | 0.914 |
| MELK | NUF2 | 0.868 | 0.914 |
| CEP55 | AURKA | 0.848 | 0.914 |
| FN1 | CFD | 0.061 | 0.914 |
| FAM83D | TPX2 | 0.773 | 0.914 |
| CEP55 | CCNB2 | 0.852 | 0.914 |
| MMP1 | MMP11 | 0 | 0.913 |
| KIF26B | KIF4A | 0 | 0.913 |
| FGF2 | PDGFRA | 0.084 | 0.913 |
| PBK | NEK2 | 0.886 | 0.913 |
| TACC3 | NDC80 | 0.889 | 0.913 |
| CDC20 | ZBTB16 | 0 | 0.913 |
| ECT2 | NCAPG | 0.883 | 0.913 |
| DEPDC1 | TTK | 0.854 | 0.913 |
| CENPF | AURKA | 0.846 | 0.913 |
| MKI67 | CCNB1 | 0.791 | 0.912 |
| CEP55 | NUF2 | 0.832 | 0.912 |
| MKI67 | UBE2C | 0.846 | 0.912 |
| HJURP | TTK | 0.893 | 0.912 |
| ADRB2 | GNG11 | 0 | 0.912 |
| KIF4A | ANLN | 0.797 | 0.912 |
| TYMS | KIF11 | 0.897 | 0.912 |
| KIF2C | MELK | 0.848 | 0.911 |
| FOXM1 | BIRC5 | 0.809 | 0.911 |
| HJURP | BUB1B | 0.887 | 0.911 |
| CKS2 | CDT1 | 0.299 | 0.911 |
| ECT2 | CCNB2 | 0.86 | 0.91 |
| CENPF | KIF23 | 0.835 | 0.91 |
| RACGAP1 | TPX2 | 0.867 | 0.91 |
| PRC1 | CDKN3 | 0.819 | 0.91 |
| IL4I1 | MAOA | 0 | 0.91 |
| ZNF423 | PPARG | 0 | 0.91 |
| CDK1 | DTL | 0.855 | 0.91 |
| CDCA3 | CCNB1 | 0.843 | 0.909 |
| PTTG1 | RRM2 | 0.833 | 0.909 |
| TOP2A | LMNB1 | 0.885 | 0.909 |
| PRC1 | TACC3 | 0.895 | 0.909 |
| PTTG1 | CKS2 | 0.85 | 0.909 |
| TPX2 | ANLN | 0.839 | 0.908 |
| CDC20 | CDKN3 | 0.825 | 0.908 |
| KIF26B | KIF2C | 0 | 0.908 |
| HJURP | MELK | 0.842 | 0.908 |
| PTTG1 | TTK | 0.833 | 0.908 |
| PTTG1 | SPAG5 | 0.875 | 0.908 |
| KIF18B | SPAG5 | 0.867 | 0.907 |
| PRC1 | ANLN | 0.854 | 0.907 |
| GPC3 | CHRDL1 | 0.061 | 0.907 |
| HJURP | BIRC5 | 0.887 | 0.907 |
| CDCA3 | AURKA | 0.87 | 0.907 |
| PTTG1 | CEP55 | 0.831 | 0.907 |
| TYMS | CCNA2 | 0.838 | 0.907 |
| SPRY2 | PTPRB | 0 | 0.907 |
| CDKN3 | NCAPG | 0.904 | 0.907 |
| CKS2 | TYMS | 0.863 | 0.907 |
| HJURP | ASPM | 0.893 | 0.906 |
| NRN1 | GPIHBP1 | 0.102 | 0.906 |
| SPRY1 | PTPRB | 0 | 0.906 |
| SPAG5 | TPX2 | 0.878 | 0.906 |
| HJURP | CDCA8 | 0.891 | 0.906 |
| NUSAP1 | CKS2 | 0.844 | 0.906 |
| KIF18B | KIF26B | 0 | 0.905 |
| CKS2 | BIRC5 | 0.838 | 0.905 |
| PBK | ANLN | 0.865 | 0.905 |
| PGM5 | DMD | 0.063 | 0.905 |
| CHRDL1 | FN1 | 0 | 0.905 |
| TTK | UBE2C | 0.871 | 0.905 |
| PROS1 | CFD | 0.061 | 0.905 |
| TYMS | AURKA | 0.86 | 0.904 |
| PTTG1 | NDC80 | 0.813 | 0.904 |
| PRC1 | CEP55 | 0.836 | 0.904 |
| RRM2 | AURKA | 0.825 | 0.904 |
| TOP2A | CKS2 | 0.826 | 0.904 |
| CDCA3 | SPAG5 | 0.867 | 0.904 |
| MAD2L1 | GINS2 | 0.889 | 0.904 |
| CD36 | PTPRB | 0.062 | 0.903 |
| ECT2 | CDCA8 | 0.852 | 0.903 |
| FOXM1 | KIF11 | 0.868 | 0.903 |
| CXCL12 | GNG11 | 0 | 0.903 |
| MKI67 | NDC80 | 0.883 | 0.903 |
| CDCA3 | MAD2L1 | 0.866 | 0.903 |
| CEP55 | BIRC5 | 0.834 | 0.903 |
| CXCL11 | GNG11 | 0 | 0.902 |
| KIF4A | CENPF | 0.837 | 0.902 |
| PRC1 | KIF2C | 0.839 | 0.902 |
| MKI67 | SPAG5 | 0.887 | 0.902 |
| KIF18B | BUB1B | 0.825 | 0.902 |
| DEPDC1 | NEK2 | 0.876 | 0.902 |
| CDCA3 | TPX2 | 0.877 | 0.902 |
| UBE2C | NCAPG | 0.864 | 0.902 |
| KIF4A | TTK | 0.866 | 0.902 |
| CDCA3 | RRM2 | 0.867 | 0.901 |
| FN1 | FGF2 | 0.088 | 0.901 |
| PRC1 | DLGAP5 | 0.899 | 0.901 |
| PTTG1 | NUF2 | 0.808 | 0.901 |
| DTL | CCNA2 | 0.826 | 0.9 |
| CXCL10 | GNG11 | 0 | 0.9 |
| SPP1 | CHRDL1 | 0 | 0.9 |
| SPAG5 | CCNB1 | 0.872 | 0.9 |
| MAML2 | DLGAP5 | 0 | 0.9 |
| KIF4A | NUF2 | 0.816 | 0.899 |
| TOP2A | CDKN3 | 0.874 | 0.899 |
| CKS2 | AURKA | 0.809 | 0.899 |
| FOXM1 | DLGAP5 | 0.864 | 0.899 |
| KIF11 | TRIP13 | 0.874 | 0.899 |
| CDCA3 | KIF23 | 0.851 | 0.899 |
| CEP55 | TPX2 | 0.859 | 0.899 |
| CKS2 | CDCA8 | 0.876 | 0.899 |
| NEK2 | KIF23 | 0.846 | 0.898 |
| PTTG1 | MELK | 0.858 | 0.898 |
| NUF2 | AURKA | 0.739 | 0.898 |
| HJURP | RRM2 | 0.88 | 0.898 |
| E2F8 | MKI67 | 0.87 | 0.898 |
| TTK | NEK2 | 0.87 | 0.898 |
| NEK2 | FOXM1 | 0.679 | 0.898 |
| CENPF | ANLN | 0.806 | 0.897 |
| PTTG1 | KIF11 | 0.801 | 0.897 |
| CDCA3 | NEK2 | 0.869 | 0.897 |
| PBK | MKI67 | 0.869 | 0.897 |
| KIF4A | MKI67 | 0.867 | 0.897 |
| CDCA3 | PTTG1 | 0.839 | 0.896 |
| MKI67 | NCAPG | 0.887 | 0.896 |
| TTK | CDKN3 | 0.873 | 0.896 |
| MKI67 | TPX2 | 0.852 | 0.896 |
| RACGAP1 | ASPM | 0.881 | 0.896 |
| CLDN5 | VWF | 0.687 | 0.895 |
| KIF20A | IQGAP3 | 0.885 | 0.895 |
| CEP55 | CDKN3 | 0.891 | 0.895 |
| HMMR | UBE2C | 0.83 | 0.894 |
| TYMS | GINS2 | 0.857 | 0.894 |
| ECT2 | CCNB1 | 0.802 | 0.893 |
| MKI67 | AURKA | 0.777 | 0.893 |
| CDK1 | SHCBP1 | 0.881 | 0.893 |
| E2F8 | ASPM | 0.863 | 0.893 |
| PRC1 | CDCA8 | 0.882 | 0.892 |
| UHRF1 | CDCA8 | 0.879 | 0.892 |
| ADH1B | ALDH1A1 | 0.086 | 0.892 |
| CENPU | NCAPG | 0.837 | 0.892 |
| ASPM | ANLN | 0.869 | 0.892 |
| NUSAP1 | RACGAP1 | 0.842 | 0.892 |
| KIF2C | ANLN | 0.786 | 0.892 |
| ECT2 | TTK | 0.853 | 0.891 |
| ANLN | CCNB1 | 0.823 | 0.891 |
| FN1 | AURKA | 0.049 | 0.891 |
| FAM83D | KIF4A | 0.66 | 0.891 |
| E2F8 | KIF2C | 0.872 | 0.891 |
| HMMR | CCNB1 | 0.825 | 0.891 |
| KIF18B | MKI67 | 0.89 | 0.89 |
| HMMR | ANLN | 0.843 | 0.89 |
| ZNF423 | EBF1 | 0.061 | 0.89 |
| CDT1 | GINS2 | 0.817 | 0.89 |
| CDCA3 | FOXM1 | 0.848 | 0.89 |
| PTTG1 | KIF4A | 0.79 | 0.889 |
| PTTG1 | ASPM | 0.844 | 0.889 |
| KIF4A | MAD2L1 | 0.8 | 0.889 |
| PRC1 | BIRC5 | 0.843 | 0.889 |
| ECT2 | BUB1B | 0.829 | 0.888 |
| NUSAP1 | ZWINT | 0.781 | 0.888 |
| PBK | ECT2 | 0.866 | 0.888 |
| RACGAP1 | NCAPG | 0.852 | 0.888 |
| CDCA3 | NDC80 | 0.881 | 0.887 |
| KIF20A | HMMR | 0.845 | 0.887 |
| NUSAP1 | TYMS | 0.847 | 0.887 |
| PRC1 | MELK | 0.858 | 0.887 |
| PTTG1 | NEK2 | 0.743 | 0.886 |
| NUF2 | KIF23 | 0.802 | 0.886 |
| PBK | PRC1 | 0.873 | 0.885 |
| HJURP | TPX2 | 0.86 | 0.885 |
| CCNA2 | GINS2 | 0.856 | 0.885 |
| HJURP | IQGAP3 | 0.875 | 0.885 |
| CCNB2 | TRIP13 | 0.835 | 0.885 |
| SHCBP1 | BUB1B | 0.885 | 0.885 |
| HMMR | CCNB2 | 0.83 | 0.884 |
| KIF18B | CDCA8 | 0.869 | 0.884 |
| RACGAP1 | HMMR | 0.804 | 0.884 |
| PRC1 | HMMR | 0.826 | 0.884 |
| UBE2C | KIF23 | 0.819 | 0.883 |
| HJURP | NCAPG | 0.835 | 0.883 |
| PRC1 | PTTG1 | 0.824 | 0.883 |
| TOP2A | ANLN | 0.834 | 0.883 |
| SHCBP1 | TPX2 | 0.858 | 0.883 |
| RRM2 | TK1 | 0.732 | 0.883 |
| CCNA2 | ANLN | 0.833 | 0.883 |
| HMMR | CDC20 | 0.824 | 0.882 |
| CEP55 | MAD2L1 | 0.841 | 0.882 |
| HMMR | MELK | 0.845 | 0.882 |
| TOP2A | SHCBP1 | 0.872 | 0.881 |
| CKS2 | KIF11 | 0.856 | 0.881 |
| HMMR | KIF11 | 0.838 | 0.881 |
| CDK1 | RAD51AP1 | 0.849 | 0.88 |
| HMMR | CCNA2 | 0.837 | 0.88 |
| ECT2 | CEP55 | 0.793 | 0.88 |
| TACC3 | CCNB1 | 0.812 | 0.88 |
| CCNB1 | TRIP13 | 0.831 | 0.88 |
| MKI67 | MELK | 0.813 | 0.88 |
| AURKA | TRIP13 | 0.859 | 0.88 |
| FANCI | NCAPG | 0.866 | 0.879 |
| NCAPG | RAD51AP1 | 0.823 | 0.879 |
| TOP2A | ATAD2 | 0.788 | 0.879 |
| MELK | ANLN | 0.831 | 0.879 |
| RACGAP1 | ZWINT | 0.814 | 0.879 |
| ASPM | FOXM1 | 0.871 | 0.879 |
| SHCBP1 | CCNB2 | 0.871 | 0.878 |
| HJURP | SPAG5 | 0.878 | 0.878 |
| CEP55 | DTL | 0.829 | 0.878 |
| DTL | CCNB1 | 0.819 | 0.878 |
| FOXM1 | NDC80 | 0.864 | 0.878 |
| PTTG1 | UBE2T | 0.84 | 0.877 |
| DEPDC1 | CCNA2 | 0.874 | 0.877 |
| FOXM1 | KIF23 | 0.84 | 0.876 |
| NUSAP1 | CENPM | 0.808 | 0.876 |
| DEPDC1 | CDK1 | 0.852 | 0.876 |
| GINS2 | TRIP13 | 0.858 | 0.876 |
| ASPM | FANCI | 0.845 | 0.875 |
| BIRC5 | GINS2 | 0.811 | 0.875 |
| TYMS | CCNB2 | 0.834 | 0.874 |
| PBK | FOXM1 | 0.808 | 0.874 |
| HMMR | BIRC5 | 0.807 | 0.874 |
| CDCA8 | TRIP13 | 0.832 | 0.874 |
| KIF20A | ZWINT | 0.785 | 0.874 |
| ZWINT | UBE2C | 0.803 | 0.874 |
| HMMR | KIF4A | 0.836 | 0.873 |
| RRM2 | FOXM1 | 0.815 | 0.873 |
| TYMS | NUF2 | 0.819 | 0.873 |
| MKI67 | NEK2 | 0.813 | 0.873 |
| CDKN3 | KIF11 | 0.822 | 0.873 |
| ADH1C | ALDH1A1 | 0.097 | 0.872 |
| ISG15 | CXCL10 | 0.393 | 0.872 |
| HMMR | TTK | 0.844 | 0.872 |
| RACGAP1 | RRM2 | 0.812 | 0.872 |
| TTK | FOXM1 | 0.822 | 0.872 |
| TTK | RAD51AP1 | 0.833 | 0.872 |
| ZWINT | TPX2 | 0.811 | 0.872 |
| CCNB2 | ANLN | 0.821 | 0.871 |
| MAD2L1 | ANLN | 0.824 | 0.871 |
| CDKN3 | BUB1B | 0.825 | 0.871 |
| PBK | DEPDC1 | 0.816 | 0.871 |
| PTTG1 | TYMS | 0.816 | 0.871 |
| CDCA3 | HJURP | 0.869 | 0.871 |
| ZWINT | KIF11 | 0.796 | 0.871 |
| TOP2A | RAD51AP1 | 0.824 | 0.871 |
| TACC3 | TPX2 | 0.67 | 0.871 |
| DTL | CENPF | 0.835 | 0.871 |
| CDK1 | GINS1 | 0.838 | 0.871 |
| CDK1 | FANCI | 0.84 | 0.871 |
| RACGAP1 | NDC80 | 0.83 | 0.87 |
| CKS2 | MELK | 0.821 | 0.87 |
| ECT2 | MAD2L1 | 0.823 | 0.87 |
| MELK | CENPU | 0.826 | 0.87 |
| UBE2C | NUF2 | 0.816 | 0.87 |
| NUSAP1 | FANCI | 0.834 | 0.869 |
| PTTG1 | ZWINT | 0.784 | 0.869 |
| HMMR | MAD2L1 | 0.848 | 0.869 |
| ZWINT | RAD51AP1 | 0.784 | 0.869 |
| DTL | TYMS | 0.809 | 0.869 |
| CDC20 | SHCBP1 | 0.867 | 0.869 |
| HJURP | KIF4A | 0.812 | 0.869 |
| ZWINT | DLGAP5 | 0.807 | 0.869 |
| CCNA2 | RAD51AP1 | 0.827 | 0.868 |
| FOXM1 | NCAPG | 0.838 | 0.868 |
| SPAG5 | NCAPG | 0.817 | 0.868 |
| TOP2A | ZWINT | 0.802 | 0.868 |
| UBE2T | MAD2L1 | 0.844 | 0.868 |
| PRC1 | CENPU | 0.776 | 0.867 |
| RRM2 | KIF23 | 0.842 | 0.867 |
| PRC1 | NEK2 | 0.803 | 0.867 |
| DEPDC1 | NDC80 | 0.848 | 0.867 |
| UBE2C | TRIP13 | 0.801 | 0.866 |
| TPX2 | GINS2 | 0.791 | 0.866 |
| HJURP | CEP55 | 0.839 | 0.866 |
| E2F8 | SPAG5 | 0.863 | 0.866 |
| CDCA8 | GINS2 | 0.837 | 0.866 |
| ZWINT | CEP55 | 0.813 | 0.866 |
| FANCI | KIF11 | 0.824 | 0.866 |
| CCNA2 | LMNB1 | 0.819 | 0.866 |
| KIF18B | HJURP | 0.849 | 0.865 |
| ZWINT | MELK | 0.827 | 0.865 |
| UBE2C | CDKN3 | 0.846 | 0.865 |
| DTL | CCNB2 | 0.82 | 0.865 |
| RACGAP1 | TTK | 0.815 | 0.865 |
| ZWINT | RRM2 | 0.822 | 0.865 |
| PRC1 | CENPF | 0.818 | 0.865 |
| RACGAP1 | UBE2C | 0.81 | 0.864 |
| RACGAP1 | PTTG1 | 0.798 | 0.864 |
| CCNB2 | GINS2 | 0.83 | 0.864 |
| RRM2 | CDKN3 | 0.838 | 0.864 |
| PRC1 | RRM2 | 0.839 | 0.863 |
| DTL | KIF11 | 0.846 | 0.863 |
| FOXM1 | SPAG5 | 0.863 | 0.863 |
| NUSAP1 | RAD51AP1 | 0.796 | 0.863 |
| ECT2 | KIF4A | 0.774 | 0.863 |
| NUSAP1 | GINS2 | 0.811 | 0.863 |
| NEK2 | MELK | 0.836 | 0.863 |
| TTK | ANLN | 0.833 | 0.862 |
| MELK | TRIP13 | 0.842 | 0.862 |
| CDKN3 | TPX2 | 0.843 | 0.862 |
| TPX2 | RAD51AP1 | 0.801 | 0.861 |
| UBE2T | NUF2 | 0.801 | 0.861 |
| FANCI | BUB1B | 0.822 | 0.861 |
| HJURP | AURKA | 0.835 | 0.861 |
| CDCA8 | RAD51AP1 | 0.806 | 0.861 |
| NCAPG | TRIP13 | 0.83 | 0.861 |
| NUSAP1 | ANLN | 0.811 | 0.861 |
| CDCA3 | TACC3 | 0.847 | 0.861 |
| KIF20A | CDKN3 | 0.832 | 0.861 |
| GINS2 | DLGAP5 | 0.819 | 0.861 |
| GINS2 | RAD51AP1 | 0.802 | 0.861 |
| PDGFD | IGF1 | 0.077 | 0.86 |
| CKS2 | DLGAP5 | 0.81 | 0.86 |
| CDC20 | ANLN | 0.792 | 0.86 |
| CEP55 | TRIP13 | 0.817 | 0.86 |
| ANLN | AURKA | 0.801 | 0.86 |
| HMMR | BUB1B | 0.816 | 0.86 |
| PRC1 | NUF2 | 0.835 | 0.86 |
| CKS2 | BUB1B | 0.77 | 0.859 |
| KIF18B | UBE2C | 0.817 | 0.859 |
| UBE2C | ANLN | 0.793 | 0.859 |
| CCNB2 | RAD51AP1 | 0.797 | 0.859 |
| HJURP | ZWINT | 0.778 | 0.859 |
| KIF20A | TRIP13 | 0.824 | 0.859 |
| CDKN3 | BIRC5 | 0.822 | 0.859 |
| CCNB1 | GINS2 | 0.817 | 0.859 |
| MAD2L1 | RAD51AP1 | 0.832 | 0.858 |
| CDCA8 | CDKN3 | 0.814 | 0.858 |
| NUSAP1 | DTL | 0.83 | 0.858 |
| HJURP | HMMR | 0.811 | 0.858 |
| NEK2 | NCAPG | 0.815 | 0.858 |
| HMMR | CENPU | 0.788 | 0.858 |
| NEK2 | NUF2 | 0.751 | 0.858 |
| KIF18B | TOP2A | 0.837 | 0.858 |
| PRC1 | SHCBP1 | 0.812 | 0.857 |
| TYMS | MELK | 0.821 | 0.857 |
| TOP2A | TK1 | 0.722 | 0.857 |
| RRM2 | CDT1 | 0.735 | 0.857 |
| NUSAP1 | SHCBP1 | 0.843 | 0.856 |
| CDCA3 | NUF2 | 0.745 | 0.856 |
| UBE2C | GINS2 | 0.822 | 0.856 |
| ECT2 | NEK2 | 0.8 | 0.856 |
| PBK | DTL | 0.831 | 0.856 |
| CENPF | CDKN3 | 0.822 | 0.856 |
| PTTG1 | FOXM1 | 0.687 | 0.856 |
| TOP2A | FANCI | 0.812 | 0.856 |
| KIF4A | SHCBP1 | 0.8 | 0.856 |
| HMMR | RRM2 | 0.811 | 0.856 |
| PBK | SHCBP1 | 0.811 | 0.856 |
| NUSAP1 | CENPU | 0.784 | 0.855 |
| TTK | TRIP13 | 0.826 | 0.855 |
| HMMR | NDC80 | 0.844 | 0.855 |
| GINS2 | AURKA | 0.821 | 0.855 |
| BUB1B | ANLN | 0.809 | 0.855 |
| MELK | RAD51AP1 | 0.824 | 0.855 |
| DTL | DLGAP5 | 0.848 | 0.855 |
| UHRF1 | ASPM | 0.83 | 0.854 |
| HMMR | FANCI | 0.801 | 0.854 |
| RACGAP1 | NEK2 | 0.767 | 0.854 |
| PBK | TRIP13 | 0.821 | 0.854 |
| PBK | TYMS | 0.822 | 0.853 |
| PBK | CKS2 | 0.819 | 0.853 |
| PRC1 | TYMS | 0.829 | 0.853 |
| RRM2 | CENPM | 0.784 | 0.853 |
| ZWINT | KIF23 | 0.782 | 0.853 |
| TOP2A | DTL | 0.826 | 0.853 |
| FOXM1 | EZH2 | 0.342 | 0.853 |
| KIF4A | RRM2 | 0.812 | 0.853 |
| PTTG1 | FGF2 | 0 | 0.853 |
| ANLN | NCAPG | 0.82 | 0.852 |
| HMMR | CDKN3 | 0.83 | 0.852 |
| ECT2 | TPX2 | 0.814 | 0.852 |
| PRC1 | FOXM1 | 0.813 | 0.852 |
| CDC20 | TK1 | 0.644 | 0.851 |
| KIF11 | GINS2 | 0.817 | 0.851 |
| E2F8 | CDK1 | 0.645 | 0.851 |
| DTL | FANCI | 0.826 | 0.851 |
| HJURP | PTTG1 | 0.814 | 0.851 |
| ZWINT | TTK | 0.781 | 0.851 |
| SHCBP1 | MELK | 0.832 | 0.85 |
| FOXM1 | CDKN3 | 0.782 | 0.85 |
| CXCL12 | FGF2 | 0.07 | 0.85 |
| KIF20A | CENPM | 0.79 | 0.85 |
| UHRF1 | SPAG5 | 0.842 | 0.85 |
| KIF4A | FOXM1 | 0.819 | 0.85 |
| FANCI | MELK | 0.819 | 0.85 |
| TACC3 | CCNB2 | 0.764 | 0.85 |
| TPX2 | TRIP13 | 0.824 | 0.85 |
| TTK | TYMS | 0.821 | 0.849 |
| KIF18B | DLGAP5 | 0.803 | 0.849 |
| GHR | IGF1 | 0.089 | 0.849 |
| TYMS | TPX2 | 0.83 | 0.849 |
| FANCI | CCNA2 | 0.814 | 0.849 |
| TTK | DTL | 0.847 | 0.848 |
| TOP2A | CENPM | 0.784 | 0.848 |
| ASPM | CENPU | 0.805 | 0.848 |
| PRC1 | CKS2 | 0.796 | 0.847 |
| UHRF1 | CCNB2 | 0.786 | 0.847 |
| GINS2 | CENPM | 0.816 | 0.846 |
| KIF2C | CDKN3 | 0.826 | 0.846 |
| TOP2A | TRIP13 | 0.804 | 0.846 |
| TYMS | BIRC5 | 0.754 | 0.846 |
| NUSAP1 | TRIP13 | 0.819 | 0.846 |
| ZWINT | CCNA2 | 0.79 | 0.845 |
| NDC80 | TRIP13 | 0.833 | 0.844 |
| EZH2 | MELK | 0.789 | 0.844 |
| KIF11 | RAD51AP1 | 0.816 | 0.844 |
| CEP55 | CENPU | 0.777 | 0.844 |
| SPAG5 | NUF2 | 0.743 | 0.844 |
| BUB1B | RAD51AP1 | 0.801 | 0.844 |
| ASPM | TYMS | 0.838 | 0.843 |
| RRM2 | ANLN | 0.795 | 0.843 |
| FANCI | RAD51AP1 | 0.786 | 0.843 |
| BUB1B | UBE2S | 0.201 | 0.843 |
| SPAG5 | MAD2L1 | 0.784 | 0.842 |
| NUF2 | ANLN | 0.748 | 0.842 |
| TTK | FANCI | 0.799 | 0.842 |
| HJURP | TRIP13 | 0.78 | 0.842 |
| DTL | BUB1B | 0.835 | 0.842 |
| ZWINT | MKI67 | 0.739 | 0.841 |
| RACGAP1 | RAD51AP1 | 0.792 | 0.841 |
| PBK | ZWINT | 0.813 | 0.841 |
| KIF20A | TYMS | 0.802 | 0.841 |
| NEK2 | SHCBP1 | 0.841 | 0.841 |
| FANCI | CCNB1 | 0.81 | 0.841 |
| DTL | MELK | 0.834 | 0.84 |
| PLIN4 | PLIN1 | 0.231 | 0.84 |
| CENPU | KIF11 | 0.79 | 0.84 |
| KIF20A | CKS2 | 0.806 | 0.84 |
| BIRC5 | CDT1 | 0.801 | 0.84 |
| ZWINT | SPAG5 | 0.748 | 0.84 |
| KIF18B | FOXM1 | 0.832 | 0.84 |
| KIF2C | CDT1 | 0.796 | 0.84 |
| DTL | GINS2 | 0.831 | 0.84 |
| PBK | HJURP | 0.828 | 0.84 |
| CEP55 | RAD51AP1 | 0.805 | 0.84 |
| CEP55 | CENPM | 0.748 | 0.84 |
| MMP9 | FGF2 | 0 | 0.839 |
| UBE2T | CENPF | 0.792 | 0.839 |
| HMMR | NUF2 | 0.808 | 0.838 |
| KIF4A | TRIP13 | 0.81 | 0.838 |
| KIF18B | ASPM | 0.771 | 0.838 |
| CDK1 | UBE2T | 0.801 | 0.838 |
| HMMR | KIF2C | 0.82 | 0.838 |
| MKI67 | NUF2 | 0.759 | 0.837 |
| RRM2 | TRIP13 | 0.819 | 0.837 |
| KIF20A | RAD51AP1 | 0.791 | 0.837 |
| TYMS | DLGAP5 | 0.819 | 0.837 |
| HMMR | MKI67 | 0.783 | 0.837 |
| KIF4A | CDKN3 | 0.809 | 0.837 |
| HJURP | CCNB1 | 0.806 | 0.837 |
| ATAD2 | KIF11 | 0.792 | 0.836 |
| ZWINT | GINS2 | 0.785 | 0.836 |
| DTL | TPX2 | 0.82 | 0.836 |
| KIF2C | GINS2 | 0.802 | 0.836 |
| ECT2 | BIRC5 | 0.795 | 0.836 |
| CDC20 | DTL | 0.765 | 0.836 |
| HMMR | CDCA8 | 0.81 | 0.836 |
| HMMR | FOXM1 | 0.785 | 0.835 |
| ASPM | SHCBP1 | 0.834 | 0.835 |
| MELK | CENPM | 0.796 | 0.835 |
| CKS2 | RRM2 | 0.758 | 0.835 |
| E2F8 | FOXM1 | 0.754 | 0.835 |
| ECT2 | SHCBP1 | 0.817 | 0.835 |
| CEP55 | TYMS | 0.785 | 0.835 |
| DEPDC1 | HMMR | 0.805 | 0.834 |
| TOP2A | GINS2 | 0.793 | 0.834 |
| HMMR | ECT2 | 0.799 | 0.834 |
| CXCL12 | MMP9 | 0 | 0.834 |
| PTTG1 | CENPM | 0.769 | 0.834 |
| TTK | CENPU | 0.789 | 0.834 |
| HMMR | CKS2 | 0.788 | 0.834 |
| MELK | GINS2 | 0.82 | 0.833 |
| RRM2 | LMNB1 | 0.825 | 0.833 |
| PTTG1 | GINS2 | 0.784 | 0.833 |
| PBK | RAD51AP1 | 0.824 | 0.833 |
| UBE2T | DTL | 0.781 | 0.833 |
| HMMR | ZWINT | 0.789 | 0.832 |
| HMMR | STIL | 0.743 | 0.832 |
| TOP2A | RNASEH2A | 0.563 | 0.832 |
| ANLN | NDC80 | 0.813 | 0.832 |
| FANCI | DLGAP5 | 0.81 | 0.832 |
| ZWINT | NCAPG | 0.795 | 0.832 |
| HJURP | RACGAP1 | 0.58 | 0.832 |
| DTL | CDKN3 | 0.785 | 0.832 |
| UBE2C | FANCI | 0.783 | 0.831 |
| CENPU | DLGAP5 | 0.787 | 0.831 |
| CDT1 | NCAPG | 0.807 | 0.831 |
| FANCI | CCNB2 | 0.793 | 0.831 |
| NUSAP1 | CDKN3 | 0.817 | 0.83 |
| DEPDC1 | KIF2C | 0.793 | 0.83 |
| CEP55 | SHCBP1 | 0.805 | 0.83 |
| TYMS | CCNB1 | 0.725 | 0.829 |
| FANCI | GINS2 | 0.786 | 0.829 |
| CEP55 | FANCI | 0.804 | 0.829 |
| HMMR | KIF23 | 0.802 | 0.829 |
| ECT2 | DLGAP5 | 0.805 | 0.829 |
| FANCI | AURKA | 0.794 | 0.828 |
| FOXM1 | TRIP13 | 0.799 | 0.828 |
| TK1 | CCNA2 | 0.698 | 0.828 |
| DLGAP5 | TRIP13 | 0.803 | 0.828 |
| PRC1 | FANCI | 0.811 | 0.828 |
| DLGAP5 | RAD51AP1 | 0.816 | 0.828 |
| DTL | NDC80 | 0.828 | 0.828 |
| TOP2A | IQGAP3 | 0.802 | 0.827 |
| NEK2 | RAD51AP1 | 0.76 | 0.827 |
| PRC1 | DTL | 0.812 | 0.827 |
| ASPM | RAD51AP1 | 0.827 | 0.827 |
| KIF2C | RAD51AP1 | 0.778 | 0.827 |
| HMMR | TRIP13 | 0.788 | 0.827 |
| ZWINT | TYMS | 0.772 | 0.827 |
| UHRF1 | BUB1B | 0.801 | 0.826 |
| ASPM | TRIP13 | 0.803 | 0.826 |
| RRM2 | RAD51AP1 | 0.792 | 0.826 |
| SHCBP1 | DLGAP5 | 0.784 | 0.826 |
| SHCBP1 | NDC80 | 0.826 | 0.826 |
| PBK | GINS2 | 0.802 | 0.826 |
| RRM2 | FANCI | 0.795 | 0.826 |
| KIF20A | FANCI | 0.795 | 0.826 |
| CDCA8 | ANLN | 0.777 | 0.826 |
| CKS2 | CDKN3 | 0.795 | 0.825 |
| HMMR | GINS2 | 0.784 | 0.824 |
| ATAD2 | CCNA2 | 0.783 | 0.824 |
| PRC1 | MKI67 | 0.819 | 0.824 |
| ECT2 | CENPF | 0.737 | 0.824 |
| CDKN3 | NDC80 | 0.807 | 0.823 |
| TTK | GINS2 | 0.8 | 0.823 |
| FANCI | CENPU | 0.785 | 0.823 |
| E2F8 | CDC20 | 0.734 | 0.823 |
| RRM2 | GINS2 | 0.815 | 0.823 |
| RACGAP1 | CENPF | 0.714 | 0.823 |
| CENPF | TRIP13 | 0.787 | 0.823 |
| UBE2C | TK1 | 0.739 | 0.823 |
| CENPF | FANCI | 0.79 | 0.822 |
| DEPDC1 | RRM2 | 0.773 | 0.822 |
| UHRF1 | RRM2 | 0.77 | 0.822 |
| CDCA8 | TYMS | 0.808 | 0.821 |
| MKI67 | IQGAP3 | 0.8 | 0.821 |
| ECT2 | NUF2 | 0.728 | 0.821 |
| CDK1 | ATAD2 | 0.785 | 0.821 |
| PTTG1 | KIF23 | 0.712 | 0.821 |
| ZWINT | NEK2 | 0.649 | 0.821 |
| DTL | FOXM1 | 0.795 | 0.821 |
| CDKN3 | KIF23 | 0.785 | 0.821 |
| CKS2 | NUF2 | 0.771 | 0.82 |
| SHCBP1 | BIRC5 | 0.779 | 0.82 |
| PTTG1 | TRIP13 | 0.781 | 0.819 |
| DEPDC1 | CDCA8 | 0.789 | 0.819 |
| DEPDC1 | NUF2 | 0.645 | 0.819 |
| E2F8 | KIF20A | 0.767 | 0.819 |
| FANCI | BIRC5 | 0.775 | 0.819 |
| KIF2C | TACC3 | 0.671 | 0.819 |
| ZWINT | AURKA | 0.743 | 0.819 |
| CDKN3 | TYMS | 0.772 | 0.818 |
| SPP1 | MMP9 | 0.109 | 0.818 |
| RRM2 | ATAD2 | 0.782 | 0.818 |
| PBK | CENPU | 0.796 | 0.818 |
| ASPM | UBE2T | 0.783 | 0.818 |
| KIF20A | GINS2 | 0.744 | 0.818 |
| PBK | FANCI | 0.791 | 0.818 |
| E2F8 | HJURP | 0.794 | 0.817 |
| CDCA8 | FANCI | 0.782 | 0.817 |
| STIL | KIF11 | 0.784 | 0.817 |
| ZWINT | DTL | 0.817 | 0.817 |
| DTL | MAD2L1 | 0.817 | 0.817 |
| CEP55 | GINS2 | 0.787 | 0.816 |
| CXCL12 | FN1 | 0.12 | 0.816 |
| UBE2C | CENPM | 0.766 | 0.816 |
| DEPDC1 | NCAPG | 0.797 | 0.816 |
| FANCI | NDC80 | 0.81 | 0.816 |
| HJURP | DEPDC1 | 0.816 | 0.816 |
| NEK2 | CDKN3 | 0.789 | 0.815 |
| STIL | TTK | 0.788 | 0.815 |
| CDCA3 | MKI67 | 0.728 | 0.815 |
| FOXM1 | CENPM | 0.792 | 0.815 |
| HMMR | RAD51AP1 | 0.807 | 0.815 |
| DTL | TRIP13 | 0.812 | 0.815 |
| UHRF1 | CDCA3 | 0.806 | 0.815 |
| DMD | ANK2 | 0.109 | 0.814 |
| SHCBP1 | CCNB1 | 0.775 | 0.814 |
| UHRF1 | NDC80 | 0.809 | 0.814 |
| KIF4A | ZWINT | 0.741 | 0.814 |
| RACGAP1 | CENPU | 0.681 | 0.814 |
| KIF4A | RAD51AP1 | 0.769 | 0.814 |
| RAD51AP1 | AURKA | 0.779 | 0.813 |
| KIF18B | CCNB1 | 0.727 | 0.813 |
| CDKN3 | ANLN | 0.809 | 0.813 |
| GINS2 | RNASEH2A | 0.799 | 0.813 |
| KIF20A | DTL | 0.808 | 0.813 |
| FANCI | TPX2 | 0.803 | 0.813 |
| SPAG5 | MELK | 0.813 | 0.813 |
| UBE2T | MELK | 0.73 | 0.812 |
| DTL | AURKA | 0.802 | 0.812 |
| CDCA8 | CDT1 | 0.796 | 0.812 |
| FAM83D | CDC20 | 0.76 | 0.812 |
| DLGAP5 | CENPM | 0.769 | 0.811 |
| CDKN3 | NUF2 | 0.793 | 0.811 |
| ASPM | ATAD2 | 0.782 | 0.811 |
| NEK2 | DTL | 0.788 | 0.811 |
| DTL | CENPU | 0.811 | 0.811 |
| RRM2 | CENPU | 0.785 | 0.811 |
| UHRF1 | NUSAP1 | 0.76 | 0.81 |
| NDC80 | GINS2 | 0.792 | 0.81 |
| DTL | UBE2C | 0.786 | 0.81 |
| ZWINT | CDKN3 | 0.806 | 0.81 |
| NUSAP1 | STIL | 0.773 | 0.81 |
| PTTG1 | DTL | 0.78 | 0.809 |
| MAD2L1 | ATAD2 | 0.774 | 0.809 |
| CCNA2 | CENPM | 0.796 | 0.809 |
| RACGAP1 | CDKN3 | 0.795 | 0.809 |
| CCNB1 | RAD51AP1 | 0.772 | 0.809 |
| E2F8 | TTK | 0.773 | 0.808 |
| KIF2C | TRIP13 | 0.797 | 0.808 |
| TPX2 | ATAD2 | 0.784 | 0.808 |
| PRC1 | TRIP13 | 0.808 | 0.808 |
| EZH2 | NCAPG | 0.808 | 0.808 |
| RRM2 | NUF2 | 0.74 | 0.808 |
| STIL | CCNA2 | 0.754 | 0.807 |
| NEK2 | ANLN | 0.74 | 0.807 |
| HMMR | TYMS | 0.785 | 0.807 |
| TOP2A | CDT1 | 0.675 | 0.807 |
| HJURP | GINS2 | 0.758 | 0.806 |
| CDKN3 | GINS2 | 0.785 | 0.806 |
| CDT1 | TPX2 | 0.767 | 0.805 |
| HMMR | DTL | 0.805 | 0.805 |
| SHCBP1 | KIF11 | 0.771 | 0.805 |
| SPAG5 | KIF23 | 0.738 | 0.805 |
| HJURP | KIF23 | 0.766 | 0.805 |
| KIF2C | FANCI | 0.79 | 0.804 |
| CDKN3 | RAD51AP1 | 0.784 | 0.804 |
| STIL | NCAPG | 0.796 | 0.804 |
| UBE2T | CCNB1 | 0.737 | 0.804 |
| NCAPG | CENPM | 0.804 | 0.804 |
| KIF4A | FANCI | 0.752 | 0.804 |
| KIF2C | TYMS | 0.777 | 0.804 |
| TPX2 | CENPM | 0.793 | 0.804 |
| CDC20 | RNASEH2A | 0.781 | 0.803 |
| FAM83D | BUB1B | 0.687 | 0.803 |
| CENPU | RAD51AP1 | 0.784 | 0.803 |
| PRC1 | SPAG5 | 0.802 | 0.803 |
| ZWINT | FANCI | 0.777 | 0.802 |
| ATAD2 | RAD51AP1 | 0.761 | 0.802 |
| RACGAP1 | DTL | 0.8 | 0.801 |
| PTTG1 | FANCI | 0.765 | 0.801 |
| GSN | FN1 | 0.07 | 0.801 |
| DEPDC1 | ECT2 | 0.74 | 0.801 |
| DEPDC1 | ANLN | 0.79 | 0.801 |
| ECT2 | RAD51AP1 | 0.776 | 0.8 |
| UHRF1 | CCNB1 | 0.725 | 0.8 |
| UHRF1 | GINS2 | 0.764 | 0.8 |
| TACC3 | NUF2 | 0.771 | 0.8 |
| DEPDC1 | MKI67 | 0.793 | 0.8 |
| ZWINT | FOXM1 | 0.777 | 0.8 |
| KIF11 | CENPM | 0.688 | 0.8 |
| DTL | NUF2 | 0.79 | 0.8 |
| PRC1 | RAD51AP1 | 0.8 | 0.8 |
| HMMR | CENPM | 0.728 | 0.799 |
| ZWINT | ASPM | 0.784 | 0.798 |
| CENPU | CCNA2 | 0.782 | 0.798 |
| FANCI | MAD2L1 | 0.784 | 0.798 |
| CDCA8 | TK1 | 0.724 | 0.798 |
| KIF2C | DTL | 0.79 | 0.797 |
| CENPF | TYMS | 0.728 | 0.797 |
| PRC1 | ZWINT | 0.78 | 0.797 |
| CDKN3 | SPAG5 | 0.796 | 0.796 |
| HJURP | CDT1 | 0.768 | 0.796 |
| BIRC5 | TRIP13 | 0.763 | 0.796 |
| HJURP | DTL | 0.783 | 0.796 |
| CKS2 | ZWINT | 0.684 | 0.796 |
| DTL | BIRC5 | 0.784 | 0.796 |
| NDC80 | RAD51AP1 | 0.79 | 0.795 |
| LEP | LIFR | 0 | 0.795 |
| EZH2 | BUB1B | 0.546 | 0.795 |
| AURKA | CENPM | 0.739 | 0.795 |
| CDCA8 | DTL | 0.79 | 0.795 |
| RACGAP1 | NUF2 | 0.719 | 0.794 |
| HMMR | SPAG5 | 0.794 | 0.794 |
| FOXM1 | GINS2 | 0.767 | 0.793 |
| ASPM | GINS2 | 0.792 | 0.792 |
| CIDEA | COX7A1 | 0.135 | 0.792 |
| SPAG5 | FANCI | 0.768 | 0.792 |
| ECT2 | MKI67 | 0.735 | 0.792 |
| HJURP | RAD51AP1 | 0.778 | 0.791 |
| CKS2 | TPX2 | 0.746 | 0.791 |
| TK1 | CCNB1 | 0.642 | 0.79 |
| CDKN3 | TRIP13 | 0.773 | 0.79 |
| DEPDC1 | KIF4A | 0.727 | 0.79 |
| HJURP | PRC1 | 0.789 | 0.789 |
| ATAD2 | NCAPG | 0.787 | 0.789 |
| TOP2A | CENPU | 0.756 | 0.789 |
| ANLN | TRIP13 | 0.789 | 0.789 |
| DTL | RAD51AP1 | 0.786 | 0.788 |
| KIF18B | RRM2 | 0.772 | 0.788 |
| HJURP | CDKN3 | 0.787 | 0.787 |
| UHRF1 | KIF20A | 0.755 | 0.787 |
| UHRF1 | KIF2C | 0.771 | 0.787 |
| BUB1B | GINS2 | 0.748 | 0.787 |
| ASPM | EZH2 | 0.777 | 0.787 |
| DEPDC1 | CCNB1 | 0.726 | 0.786 |
| DTL | SPAG5 | 0.786 | 0.786 |
| CDKN3 | CENPU | 0.786 | 0.786 |
| KIF20A | CENPU | 0.68 | 0.785 |
| TOP2A | EZH2 | 0.702 | 0.785 |
| RACGAP1 | CKS2 | 0.749 | 0.785 |
| CKS2 | NDC80 | 0.76 | 0.785 |
| CDKN3 | FANCI | 0.781 | 0.785 |
| ANLN | RAD51AP1 | 0.777 | 0.784 |
| DEPDC1 | MELK | 0.731 | 0.784 |
| UBE2T | AURKA | 0.742 | 0.784 |
| PRC1 | GINS2 | 0.783 | 0.783 |
| UHRF1 | TPX2 | 0.751 | 0.783 |
| KIF20A | TACC3 | 0.658 | 0.783 |
| CDT1 | CENPM | 0.728 | 0.783 |
| DEPDC1 | SPAG5 | 0.759 | 0.783 |
| HJURP | ANLN | 0.777 | 0.782 |
| DTL | ATAD2 | 0.782 | 0.782 |
| RRM2 | SHCBP1 | 0.743 | 0.782 |
| KIF4A | DTL | 0.776 | 0.782 |
| PBK | ATAD2 | 0.761 | 0.781 |
| STIL | MELK | 0.767 | 0.78 |
| TYMS | BUB1B | 0.711 | 0.78 |
| HJURP | FANCI | 0.749 | 0.78 |
| CKS2 | CEP55 | 0.74 | 0.78 |
| FAM83D | AURKA | 0.686 | 0.78 |
| FAM83D | CDCA8 | 0.674 | 0.779 |
| DTL | KIF23 | 0.774 | 0.778 |
| CKS2 | CENPF | 0.672 | 0.777 |
| E2F8 | CENPF | 0.724 | 0.777 |
| KIF4A | GINS2 | 0.725 | 0.777 |
| UHRF1 | MELK | 0.741 | 0.777 |
| CDCA8 | IQGAP3 | 0.758 | 0.777 |
| BIRC5 | ANLN | 0.692 | 0.777 |
| ECT2 | UBE2C | 0.708 | 0.777 |
| CDK1 | STIL | 0.687 | 0.776 |
| CIDEA | PLIN1 | 0.17 | 0.776 |
| FAM83D | TOP2A | 0.736 | 0.776 |
| RAD51AP1 | TRIP13 | 0.713 | 0.776 |
| FOXM1 | FANCI | 0.723 | 0.775 |
| UBE2C | RAD51AP1 | 0.693 | 0.774 |
| PTTG1 | ANLN | 0.68 | 0.774 |
| EZH2 | CCNB2 | 0.707 | 0.774 |
| MMP1 | FGF2 | 0 | 0.773 |
| GHR | LEP | 0.065 | 0.773 |
| EZH2 | CCNA2 | 0.606 | 0.773 |
| NEK2 | TRIP13 | 0.703 | 0.773 |
| CDC20 | FANCI | 0.726 | 0.772 |
| FOXM1 | RAD51AP1 | 0.736 | 0.772 |
| KIF18B | PRC1 | 0.562 | 0.772 |
| CCNB2 | RNASEH2A | 0.756 | 0.771 |
| CDT1 | KIF11 | 0.692 | 0.771 |
| TACC3 | KIF23 | 0.694 | 0.771 |
| CENPK | NCAPG | 0.649 | 0.77 |
| ECT2 | CKS2 | 0.68 | 0.77 |
| RACGAP1 | SPAG5 | 0.719 | 0.77 |
| CENPM | TRIP13 | 0.729 | 0.77 |
| UHRF1 | MAD2L1 | 0.707 | 0.77 |
| MELK | ATAD2 | 0.761 | 0.769 |
| GPC3 | FGF2 | 0.043 | 0.769 |
| MMP9 | CXCL10 | 0.14 | 0.769 |
| DEPDC1 | CDKN3 | 0.728 | 0.768 |
| ECM2 | OGN | 0.654 | 0.768 |
| PBK | UBE2T | 0.721 | 0.768 |
| NUSAP1 | ATAD2 | 0.768 | 0.768 |
| SPP1 | FGF2 | 0 | 0.768 |
| IQGAP3 | BUB1B | 0.754 | 0.767 |
| DEPDC1 | AURKA | 0.695 | 0.767 |
| UHRF1 | EZH2 | 0.309 | 0.766 |
| SHCBP1 | AURKA | 0.748 | 0.766 |
| SPAG5 | ANLN | 0.714 | 0.765 |
| IQGAP3 | KIF11 | 0.736 | 0.765 |
| CDC20 | UBE2T | 0.656 | 0.765 |
| UHRF1 | NEK2 | 0.73 | 0.765 |
| UHRF1 | PBK | 0.724 | 0.763 |
| DEPDC1 | KIF23 | 0.735 | 0.763 |
| FANCI | TRIP13 | 0.73 | 0.763 |
| ZWINT | TRIP13 | 0.684 | 0.763 |
| UBE2T | RRM2 | 0.688 | 0.763 |
| MKI67 | LMNB1 | 0.727 | 0.763 |
| UBE2C | SHCBP1 | 0.681 | 0.762 |
| LEP | LPL | 0.061 | 0.762 |
| FAM83D | CCNA2 | 0.739 | 0.762 |
| SHCBP1 | NUF2 | 0.762 | 0.762 |
| PBK | CENPM | 0.731 | 0.761 |
| ASPM | IQGAP3 | 0.707 | 0.76 |
| MKI67 | KIF23 | 0.731 | 0.76 |
| TOP2A | STIL | 0.758 | 0.76 |
| TTK | ATAD2 | 0.732 | 0.759 |
| CENPF | GINS2 | 0.721 | 0.759 |
| DEPDC1 | CCNB2 | 0.703 | 0.759 |
| CENPF | ATAD2 | 0.685 | 0.759 |
| CAV1 | DLC1 | 0.098 | 0.759 |
| DEPDC1 | UBE2C | 0.673 | 0.759 |
| CLMP | TFPI | 0.051 | 0.758 |
| RACGAP1 | EZH2 | 0.571 | 0.757 |
| BUB1B | LMNB1 | 0.728 | 0.757 |
| CDT1 | BUB1B | 0.596 | 0.757 |
| MTFR2 | TTK | 0.728 | 0.757 |
| UBE2C | CDT1 | 0.697 | 0.756 |
| PRC1 | CENPM | 0.734 | 0.756 |
| UBE2C | TACC3 | 0.703 | 0.756 |
| RRM2 | RNASEH2A | 0.733 | 0.755 |
| CDCA3 | MELK | 0.707 | 0.754 |
| MKI67 | SHCBP1 | 0.742 | 0.754 |
| CIDEA | PPARG | 0.061 | 0.754 |
| PTTG1 | RAD51AP1 | 0.677 | 0.754 |
| NUSAP1 | LMNB1 | 0.722 | 0.753 |
| MMP9 | TIMP4 | 0 | 0.753 |
| BIRC5 | RAD51AP1 | 0.685 | 0.752 |
| NEK2 | FANCI | 0.697 | 0.752 |
| DEPDC1 | TPX2 | 0.716 | 0.752 |
| UHRF1 | CDK1 | 0.656 | 0.752 |
| CDT1 | AURKA | 0.684 | 0.752 |
| TYMS | CENPU | 0.699 | 0.752 |
| KIF20A | CDT1 | 0.684 | 0.752 |
| DTL | CENPM | 0.751 | 0.751 |
| FAM83D | PBK | 0.734 | 0.75 |
| TYMS | CDT1 | 0.721 | 0.75 |
| KIF18B | BIRC5 | 0.706 | 0.75 |
| TMTC1 | GNAL | 0 | 0.75 |
| CENPU | GINS2 | 0.749 | 0.749 |
| CENPF | RAD51AP1 | 0.725 | 0.749 |
| GPX3 | GSTM5 | 0.061 | 0.749 |
| IGF1 | PDGFRA | 0 | 0.749 |
| EDNRB | CAV1 | 0 | 0.749 |
| MKI67 | CDKN3 | 0.708 | 0.748 |
| SPAG5 | GINS2 | 0.723 | 0.748 |
| FOXM1 | ANLN | 0.728 | 0.748 |
| ATAD2 | DLGAP5 | 0.692 | 0.747 |
| FAM83D | KIF23 | 0.736 | 0.747 |
| DTL | ANLN | 0.719 | 0.747 |
| TK1 | CCNB2 | 0.642 | 0.747 |
| KIF23 | RAD51AP1 | 0.703 | 0.746 |
| UHRF1 | KIF11 | 0.692 | 0.746 |
| NUSAP1 | CDT1 | 0.678 | 0.746 |
| FANCI | KIF23 | 0.73 | 0.746 |
| PTTG1 | RNASEH2A | 0.722 | 0.745 |
| GINS1 | NCAPG | 0.673 | 0.745 |
| UBE2T | CDKN3 | 0.74 | 0.745 |
| ASPM | CENPM | 0.74 | 0.744 |
| ATAD2 | NDC80 | 0.667 | 0.743 |
| CDKN3 | CENPM | 0.742 | 0.742 |
| CKS2 | KIF23 | 0.67 | 0.742 |
| HMMR | TACC3 | 0.594 | 0.742 |
| TTK | SHCBP1 | 0.742 | 0.742 |
| STIL | AURKA | 0.671 | 0.742 |
| LMNB1 | KIF11 | 0.618 | 0.741 |
| FOXM1 | CDT1 | 0.689 | 0.741 |
| UBE2T | BIRC5 | 0.607 | 0.74 |
| FAM83D | SPAG5 | 0.641 | 0.74 |
| DTL | EZH2 | 0.725 | 0.739 |
| UBE2C | CENPU | 0.709 | 0.739 |
| TACC3 | BIRC5 | 0.668 | 0.739 |
| TTK | CENPM | 0.704 | 0.738 |
| E2F8 | CCNB2 | 0.574 | 0.738 |
| DEPDC1 | FOXM1 | 0.592 | 0.738 |
| TYMS | CENPM | 0.738 | 0.738 |
| E2F8 | NEK2 | 0.677 | 0.738 |
| FAM83D | DLGAP5 | 0.564 | 0.738 |
| UBE2T | CCNA2 | 0.65 | 0.737 |
| STIL | TRIP13 | 0.682 | 0.737 |
| STMN1 | RACGAP1 | 0.694 | 0.736 |
| CCNE2 | CDT1 | 0.324 | 0.735 |
| KIF4A | CENPM | 0.674 | 0.735 |
| PBK | CDT1 | 0.723 | 0.734 |
| HJURP | MAD2L1 | 0.684 | 0.734 |
| KIF18B | TPX2 | 0.63 | 0.734 |
| TTK | EZH2 | 0.671 | 0.733 |
| UHRF1 | AURKA | 0.701 | 0.733 |
| CENPF | IQGAP3 | 0.703 | 0.733 |
| ECT2 | TACC3 | 0.696 | 0.733 |
| TYMS | RAD51AP1 | 0.67 | 0.733 |
| CCNB2 | LMNB1 | 0.685 | 0.733 |
| DEPDC1 | MAD2L1 | 0.705 | 0.732 |
| TYMS | TRIP13 | 0.696 | 0.732 |
| SPP1 | IGF1 | 0 | 0.732 |
| PBK | TK1 | 0.652 | 0.732 |
| FAM83D | CDCA3 | 0.622 | 0.731 |
| CEP55 | ATAD2 | 0.688 | 0.731 |
| UBE2T | CCNB2 | 0.603 | 0.731 |
| FAM83D | CENPF | 0.68 | 0.729 |
| UHRF1 | NCAPG | 0.668 | 0.728 |
| PTTG1 | CENPU | 0.68 | 0.728 |
| UBE2T | TYMS | 0.719 | 0.727 |
| RRM2 | EZH2 | 0.65 | 0.727 |
| FAM83D | KIF20A | 0.7 | 0.727 |
| NUF2 | GINS2 | 0.685 | 0.727 |
| ADH1C | MAOA | 0.102 | 0.727 |
| STIL | TPX2 | 0.663 | 0.727 |
| KIF18B | CCNA2 | 0.664 | 0.726 |
| MEOX1 | MEOX2 | 0.107 | 0.726 |
| KIF20A | TK1 | 0.6 | 0.725 |
| TK1 | AURKA | 0.618 | 0.725 |
| KIF2C | RNASEH2A | 0.725 | 0.725 |
| FAM83D | KIF2C | 0.585 | 0.724 |
| TMTC1 | EBF1 | 0.063 | 0.724 |
| ECT2 | RRM2 | 0.675 | 0.724 |
| UBE2T | TPX2 | 0.681 | 0.724 |
| CKS2 | NEK2 | 0.617 | 0.724 |
| KIF18B | CCNB2 | 0.591 | 0.723 |
| DEPDC1 | CDC20 | 0.649 | 0.723 |
| CDCA3 | PRC1 | 0.708 | 0.723 |
| TYMS | FANCI | 0.692 | 0.723 |
| CCNE2 | CKS2 | 0.198 | 0.723 |
| TK1 | KIF11 | 0.508 | 0.723 |
| CDT1 | DLGAP5 | 0.695 | 0.722 |
| STIL | CENPF | 0.577 | 0.722 |
| UBE2T | DLGAP5 | 0.705 | 0.722 |
| ECT2 | ZWINT | 0.673 | 0.722 |
| RACGAP1 | TRIP13 | 0.644 | 0.721 |
| CDCA3 | CENPM | 0.531 | 0.721 |
| KIF18B | PBK | 0.667 | 0.72 |
| TOP2A | TACC3 | 0.691 | 0.719 |
| TYMS | ANLN | 0.696 | 0.719 |
| FANCI | CENPM | 0.684 | 0.719 |
| MMP1 | TIMP4 | 0 | 0.718 |
| PTTG1 | ECT2 | 0.54 | 0.718 |
| FANCI | NUF2 | 0.649 | 0.718 |
| STIL | NDC80 | 0.702 | 0.717 |
| CD36 | LPL | 0.099 | 0.717 |
| MKI67 | EZH2 | 0.538 | 0.717 |
| CDT1 | KIF23 | 0.654 | 0.717 |
| CDCA8 | RNASEH2A | 0.716 | 0.716 |
| CAV1 | PPARG | 0.071 | 0.716 |
| HMMR | ATAD2 | 0.685 | 0.716 |
| CLDN5 | CAV1 | 0.062 | 0.716 |
| CIDEC | PLIN4 | 0.17 | 0.716 |
| CDCA3 | CENPF | 0.53 | 0.716 |
| CDCA3 | NCAPG | 0.652 | 0.716 |
| KIF18B | NDC80 | 0.527 | 0.715 |
| MKI67 | MAD2L1 | 0.626 | 0.715 |
| FOXM1 | MAD2L1 | 0.592 | 0.715 |
| CFD | LEP | 0.099 | 0.715 |
| UBE2T | GINS2 | 0.654 | 0.715 |
| CCNE2 | KIF11 | 0.576 | 0.715 |
| KIF18B | CDCA3 | 0.577 | 0.715 |
| FN1 | MMP1 | 0.139 | 0.715 |
| RBP4 | LEP | 0.06 | 0.715 |
| CCNE2 | RRM2 | 0.485 | 0.713 |
| CENPU | KIF23 | 0.634 | 0.713 |
| STIL | CEP55 | 0.69 | 0.713 |
| SHCBP1 | RAD51AP1 | 0.616 | 0.713 |
| TOP2A | UBE2T | 0.612 | 0.712 |
| CKS2 | KIF2C | 0.607 | 0.712 |
| CDC20 | IQGAP3 | 0.596 | 0.712 |
| KIF18B | TTK | 0.598 | 0.712 |
| CDCA3 | ECT2 | 0.674 | 0.711 |
| RACGAP1 | FANCI | 0.71 | 0.711 |
| PTTG1 | TK1 | 0.572 | 0.711 |
| SPAG5 | SHCBP1 | 0.711 | 0.711 |
| KIF2C | SHCBP1 | 0.671 | 0.711 |
| DEPDC1 | SHCBP1 | 0.607 | 0.71 |
| CENPK | ASPM | 0.592 | 0.71 |
| ECT2 | CDKN3 | 0.675 | 0.71 |
| DTL | RMI2 | 0.71 | 0.71 |
| KIF18B | CENPF | 0.639 | 0.71 |
| MMP9 | PPARG | 0 | 0.709 |
| FAM83D | CCNB2 | 0.654 | 0.709 |
| TACC3 | SPAG5 | 0.59 | 0.709 |
| KIF2C | STIL | 0.68 | 0.709 |
| TACC3 | BUB1B | 0.528 | 0.709 |
| KIF18B | NUSAP1 | 0.604 | 0.709 |
| UBE2T | TK1 | 0.696 | 0.709 |
| TK1 | TPX2 | 0.618 | 0.709 |
| TPX2 | RNASEH2A | 0.699 | 0.708 |
| KIF23 | TRIP13 | 0.692 | 0.708 |
| STIL | DLGAP5 | 0.698 | 0.708 |
| ATAD2 | KIF23 | 0.587 | 0.708 |
| KIF2C | IQGAP3 | 0.687 | 0.708 |
| NUSAP1 | TK1 | 0.571 | 0.707 |
| MMP9 | LEP | 0 | 0.707 |
| CENPF | CDT1 | 0.653 | 0.707 |
| CEP55 | TACC3 | 0.638 | 0.706 |
| CCNE2 | MAD2L1 | 0.41 | 0.706 |
| MKI67 | FANCI | 0.659 | 0.705 |
| PBK | STIL | 0.676 | 0.705 |
| KIF18B | MAD2L1 | 0.371 | 0.705 |
| NEK2 | ATAD2 | 0.561 | 0.704 |
| UHRF1 | SHCBP1 | 0.704 | 0.704 |
| CDCA8 | RMI2 | 0.704 | 0.704 |
| CCNE2 | CDC20 | 0.306 | 0.704 |
| GNAL | ADRB2 | 0 | 0.703 |
| CKS2 | CENPU | 0.623 | 0.703 |
| CCNE2 | ATAD2 | 0.627 | 0.703 |
| MKI67 | DTL | 0.703 | 0.703 |
| CDC20 | RAD51AP1 | 0.646 | 0.703 |
| NEK2 | LMNB1 | 0.695 | 0.702 |
| RACGAP1 | MKI67 | 0.614 | 0.702 |
| DTL | SHCBP1 | 0.654 | 0.702 |
| CDK1 | RNASEH2A | 0.675 | 0.702 |
| CDT1 | MELK | 0.701 | 0.701 |
| CENPK | TTK | 0.605 | 0.701 |
| CDT1 | NDC80 | 0.455 | 0.701 |
| UHRF1 | PRC1 | 0.656 | 0.701 |
| PTTG1 | MKI67 | 0.571 | 0.701 |
| HJURP | TYMS | 0.684 | 0.701 |
| FAM83D | NEK2 | 0.609 | 0.7 |
| KIF18B | CDK1 | 0.605 | 0.7 |
| SHCBP1 | MAD2L1 | 0.7 | 0.7 |
| RACGAP1 | FOXM1 | 0.653 | 0.7 |
| VWF | LYVE1 | 0.349 | 0.7 |
| TOP2A | RMI2 | 0.626 | 0.7 |
| CKS2 | SPAG5 | 0.605 | 0.7 |
| STIL | NEK2 | 0.559 | 0.7 |
| TK1 | MELK | 0.626 | 0.7 |
