# Supplementary material for: Identification of hub genes and small molecule therapeutic drugs related to breast cancer with comprehensive bioinformatics analysis
Source: PeerJ. 2020 Sep 29;8:e9946. doi: 10.7717/peerj.9946 (PMC7556247; doi:10.7717/peerj.9946)
Supplement: Supplemental Information 17 [file peerj-08-9946-s017.docx]

| **Group** | **Description** | **p.adjust** | **qvalue** | **geneID** |
| --- | --- | --- | --- | --- |
| BP | mitotic nuclear division | 7.67E-36 | 6.23E-36 | UBE2C/CENPF/NEK2/TPX2/BIRC5/CDC20/PTTG1/NUSAP1/MKI67/ZWINT/CCNB1/ANLN/AURKA/NUF2/KIF4A/KIF2C/KIF11/DLGAP5/CDCA8/NDC80/NCAPG/PRC1/MAD2L1/BUB1B/KIF23/RACGAP1/UBE2S/TRIP13/CDT1/TTK/SPAG5/TACC3/KIF18B/CENPK/PSRC1 |
| BP | nuclear division | 3.64E-35 | 2.96E-35 | UBE2C/TOP2A/CENPF/NEK2/TPX2/BIRC5/CDC20/PTTG1/NUSAP1/MKI67/ZWINT/CKS2/CCNB1/ANLN/AURKA/NUF2/KIF4A/KIF2C/KIF11/DLGAP5/CDCA8/NDC80/NCAPG/ASPM/PRC1/MAD2L1/BUB1B/KIF23/RACGAP1/UBE2S/TRIP13/CDT1/TTK/SPAG5/TACC3/CCNE2/KIF18B/CENPK/PSRC1 |
| BP | organelle fission | 4.24E-35 | 3.45E-35 | UBE2C/TOP2A/CENPF/NEK2/TPX2/BIRC5/CDC20/PTTG1/NUSAP1/MKI67/ZWINT/CKS2/CCNB1/ANLN/AURKA/NUF2/KIF4A/KIF2C/KIF11/DLGAP5/CDCA8/NDC80/NCAPG/ASPM/PRC1/MAD2L1/BUB1B/KIF23/RACGAP1/UBE2S/TRIP13/CDT1/TTK/SPAG5/TACC3/CCNE2/KIF18B/CENPK/MTFR2/PSRC1 |
| BP | chromosome segregation | 2.25E-33 | 1.83E-33 | TOP2A/CENPF/NEK2/BIRC5/CDC20/PTTG1/NUSAP1/MKI67/ZWINT/CCNB1/NUF2/KIF4A/RMI2/KIF2C/DLGAP5/CDCA8/NDC80/NCAPG/FAM83D/HJURP/PRC1/MAD2L1/BUB1B/KIF23/RACGAP1/TRIP13/CDT1/TTK/SPAG5/TACC3/ECT2/CCNE2/KIF18B/CENPK/PSRC1 |
| BP | nuclear chromosome segregation | 6.57E-32 | 5.34E-32 | TOP2A/CENPF/NEK2/CDC20/PTTG1/NUSAP1/ZWINT/CCNB1/NUF2/KIF4A/RMI2/KIF2C/DLGAP5/CDCA8/NDC80/NCAPG/FAM83D/PRC1/MAD2L1/BUB1B/KIF23/RACGAP1/TRIP13/CDT1/TTK/SPAG5/TACC3/ECT2/CCNE2/KIF18B/CENPK/PSRC1 |
| BP | sister chromatid segregation | 1.06E-31 | 8.63E-32 | TOP2A/CENPF/NEK2/CDC20/PTTG1/NUSAP1/ZWINT/CCNB1/NUF2/KIF4A/RMI2/KIF2C/DLGAP5/CDCA8/NDC80/NCAPG/PRC1/MAD2L1/BUB1B/KIF23/RACGAP1/TRIP13/CDT1/TTK/SPAG5/TACC3/KIF18B/CENPK/PSRC1 |
| BP | mitotic sister chromatid segregation | 2.65E-31 | 2.15E-31 | CENPF/NEK2/CDC20/PTTG1/NUSAP1/ZWINT/CCNB1/NUF2/KIF4A/KIF2C/DLGAP5/CDCA8/NDC80/NCAPG/PRC1/MAD2L1/BUB1B/KIF23/RACGAP1/TRIP13/CDT1/TTK/SPAG5/TACC3/KIF18B/CENPK/PSRC1 |
| BP | regulation of chromosome segregation | 9.30E-22 | 7.56E-22 | CENPF/NEK2/CDC20/PTTG1/MKI67/CCNB1/RMI2/KIF2C/DLGAP5/NDC80/MAD2L1/BUB1B/RACGAP1/TRIP13/CDT1/TTK/SPAG5/TACC3/ECT2 |
| BP | microtubule cytoskeleton organization involved in mitosis | 2.71E-21 | 2.20E-21 | NEK2/TPX2/BIRC5/CDC20/NUSAP1/CCNB1/AURKA/NUF2/KIF4A/KIF11/NDC80/PRC1/MAD2L1/KIF23/RACGAP1/TTK/STMN1/TACC3/STIL/PSRC1 |
| BP | mitotic spindle organization | 6.21E-20 | 5.05E-20 | NEK2/TPX2/BIRC5/CDC20/CCNB1/AURKA/NUF2/KIF4A/KIF11/NDC80/PRC1/KIF23/RACGAP1/TTK/STMN1/TACC3/STIL/PSRC1 |
| BP | spindle organization | 4.95E-19 | 4.02E-19 | NEK2/TPX2/BIRC5/CDC20/CCNB1/AURKA/NUF2/KIF4A/KIF11/NDC80/ASPM/PRC1/KIF23/RACGAP1/TTK/SPAG5/STMN1/TACC3/STIL/PSRC1 |
| BP | regulation of mitotic nuclear division | 6.50E-18 | 5.28E-18 | UBE2C/CENPF/NEK2/CDC20/PTTG1/NUSAP1/MKI67/CCNB1/ANLN/AURKA/KIF11/DLGAP5/NDC80/MAD2L1/BUB1B/TRIP13/CDT1/TTK/TACC3 |
| BP | regulation of nuclear division | 8.35E-17 | 6.78E-17 | UBE2C/CENPF/NEK2/CDC20/PTTG1/NUSAP1/MKI67/CCNB1/ANLN/AURKA/KIF11/DLGAP5/NDC80/MAD2L1/BUB1B/TRIP13/CDT1/TTK/TACC3 |
| BP | regulation of mitotic sister chromatid separation | 3.20E-14 | 2.60E-14 | CENPF/CDC20/PTTG1/CCNB1/DLGAP5/NDC80/MAD2L1/BUB1B/TRIP13/CDT1/TTK/TACC3 |
| BP | mitotic sister chromatid separation | 5.57E-14 | 4.52E-14 | CENPF/CDC20/PTTG1/CCNB1/DLGAP5/NDC80/MAD2L1/BUB1B/TRIP13/CDT1/TTK/TACC3 |
| BP | regulation of sister chromatid segregation | 5.57E-14 | 4.52E-14 | CENPF/CDC20/PTTG1/CCNB1/RMI2/DLGAP5/NDC80/MAD2L1/BUB1B/TRIP13/CDT1/TTK/TACC3 |
| BP | regulation of chromosome separation | 7.86E-14 | 6.39E-14 | CENPF/CDC20/PTTG1/CCNB1/DLGAP5/NDC80/MAD2L1/BUB1B/TRIP13/CDT1/TTK/TACC3 |
| BP | regulation of mitotic sister chromatid segregation | 2.34E-13 | 1.90E-13 | CENPF/CDC20/PTTG1/CCNB1/DLGAP5/NDC80/MAD2L1/BUB1B/TRIP13/CDT1/TTK/TACC3 |
| BP | chromosome separation | 2.34E-13 | 1.90E-13 | TOP2A/CENPF/CDC20/PTTG1/CCNB1/DLGAP5/NDC80/MAD2L1/BUB1B/TRIP13/CDT1/TTK/TACC3 |
| BP | metaphase/anaphase transition of mitotic cell cycle | 6.13E-13 | 4.98E-13 | CENPF/CDC20/CCNB1/DLGAP5/NDC80/MAD2L1/BUB1B/TRIP13/CDT1/TTK/TACC3 |
| BP | negative regulation of mitotic sister chromatid separation | 8.20E-13 | 6.67E-13 | CENPF/CDC20/PTTG1/CCNB1/NDC80/MAD2L1/BUB1B/TRIP13/CDT1/TTK |
| BP | metaphase/anaphase transition of cell cycle | 8.59E-13 | 6.98E-13 | CENPF/CDC20/CCNB1/DLGAP5/NDC80/MAD2L1/BUB1B/TRIP13/CDT1/TTK/TACC3 |
| BP | negative regulation of chromosome separation | 9.94E-13 | 8.08E-13 | CENPF/CDC20/PTTG1/CCNB1/NDC80/MAD2L1/BUB1B/TRIP13/CDT1/TTK |
| BP | negative regulation of mitotic sister chromatid segregation | 1.64E-12 | 1.33E-12 | CENPF/CDC20/PTTG1/CCNB1/NDC80/MAD2L1/BUB1B/TRIP13/CDT1/TTK |
| BP | negative regulation of sister chromatid segregation | 2.63E-12 | 2.14E-12 | CENPF/CDC20/PTTG1/CCNB1/NDC80/MAD2L1/BUB1B/TRIP13/CDT1/TTK |
| BP | negative regulation of chromosome segregation | 3.24E-12 | 2.63E-12 | CENPF/CDC20/PTTG1/CCNB1/NDC80/MAD2L1/BUB1B/TRIP13/CDT1/TTK |
| BP | attachment of spindle microtubules to kinetochore | 5.68E-12 | 4.62E-12 | NEK2/CCNB1/NUF2/KIF2C/NDC80/RACGAP1/CDT1/SPAG5/ECT2 |
| BP | mitotic spindle assembly checkpoint | 9.17E-12 | 7.45E-12 | CENPF/CDC20/CCNB1/NDC80/MAD2L1/BUB1B/TRIP13/CDT1/TTK |
| BP | spindle checkpoint | 9.17E-12 | 7.45E-12 | CENPF/CDC20/CCNB1/NDC80/MAD2L1/BUB1B/TRIP13/CDT1/TTK |
| BP | spindle assembly checkpoint | 9.17E-12 | 7.45E-12 | CENPF/CDC20/CCNB1/NDC80/MAD2L1/BUB1B/TRIP13/CDT1/TTK |
| BP | mitotic spindle checkpoint | 9.17E-12 | 7.45E-12 | CENPF/CDC20/CCNB1/NDC80/MAD2L1/BUB1B/TRIP13/CDT1/TTK |
| BP | regulation of cell cycle phase transition | 9.89E-12 | 8.03E-12 | UBE2C/CENPF/NEK2/TPX2/CDC20/CCNB1/CDK1/ANLN/AURKA/DTL/DLGAP5/HMMR/NDC80/FAM83D/MAD2L1/BUB1B/TRIP13/CDT1/TTK/EZH2/E2F8 |
| BP | regulation of mitotic metaphase/anaphase transition | 9.93E-12 | 8.07E-12 | CENPF/CDC20/CCNB1/DLGAP5/NDC80/MAD2L1/BUB1B/TRIP13/CDT1/TTK |
| BP | negative regulation of mitotic nuclear division | 1.19E-11 | 9.65E-12 | CENPF/CDC20/PTTG1/CCNB1/NDC80/MAD2L1/BUB1B/TRIP13/CDT1/TTK |
| BP | regulation of metaphase/anaphase transition of cell cycle | 1.40E-11 | 1.14E-11 | CENPF/CDC20/CCNB1/DLGAP5/NDC80/MAD2L1/BUB1B/TRIP13/CDT1/TTK |
| BP | negative regulation of mitotic metaphase/anaphase transition | 1.40E-11 | 1.14E-11 | CENPF/CDC20/CCNB1/NDC80/MAD2L1/BUB1B/TRIP13/CDT1/TTK |
| BP | establishment of chromosome localization | 1.54E-11 | 1.25E-11 | CENPF/CCNB1/NUF2/KIF2C/DLGAP5/CDCA8/NDC80/FAM83D/CDT1/SPAG5/PSRC1 |
| BP | chromosome localization | 1.70E-11 | 1.38E-11 | CENPF/CCNB1/NUF2/KIF2C/DLGAP5/CDCA8/NDC80/FAM83D/CDT1/SPAG5/PSRC1 |
| BP | negative regulation of metaphase/anaphase transition of cell cycle | 1.70E-11 | 1.38E-11 | CENPF/CDC20/CCNB1/NDC80/MAD2L1/BUB1B/TRIP13/CDT1/TTK |
| BP | mitotic cell cycle checkpoint | 1.70E-11 | 1.38E-11 | TOP2A/CENPF/CDC20/ZWINT/CCNB1/CDK1/AURKA/NDC80/MAD2L1/BUB1B/TRIP13/CDT1/TTK/E2F8 |
| BP | positive regulation of cell cycle | 1.78E-11 | 1.44E-11 | UBE2C/NUSAP1/CKS2/CCNB1/CDK1/AURKA/DTL/DLGAP5/NDC80/FAM83D/MAD2L1/KIF23/RACGAP1/CDT1/SPAG5/ECT2/EZH2/E2F8/PSRC1 |
| BP | regulation of mitotic cell cycle phase transition | 1.78E-11 | 1.44E-11 | UBE2C/CENPF/NEK2/TPX2/CDC20/CCNB1/CDK1/ANLN/AURKA/DTL/DLGAP5/HMMR/NDC80/MAD2L1/BUB1B/TRIP13/CDT1/TTK/EZH2/E2F8 |
| BP | metaphase plate congression | 2.51E-11 | 2.04E-11 | CENPF/CCNB1/NUF2/KIF2C/CDCA8/NDC80/FAM83D/CDT1/SPAG5/PSRC1 |
| BP | positive regulation of cell cycle process | 2.70E-11 | 2.19E-11 | UBE2C/NUSAP1/CCNB1/CDK1/AURKA/DTL/DLGAP5/NDC80/FAM83D/MAD2L1/KIF23/RACGAP1/CDT1/SPAG5/ECT2/EZH2/E2F8 |
| BP | cell cycle checkpoint | 3.92E-11 | 3.19E-11 | TOP2A/CENPF/CDC20/ZWINT/CCNB1/CDK1/AURKA/DTL/NDC80/MAD2L1/BUB1B/TRIP13/CDT1/TTK/E2F8 |
| BP | negative regulation of nuclear division | 4.03E-11 | 3.28E-11 | CENPF/CDC20/PTTG1/CCNB1/NDC80/MAD2L1/BUB1B/TRIP13/CDT1/TTK |
| BP | regulation of chromosome organization | 2.29E-10 | 1.86E-10 | TOP2A/CENPF/NEK2/CDC20/PTTG1/MKI67/CCNB1/RMI2/DLGAP5/NDC80/MAD2L1/BUB1B/TRIP13/CDT1/TTK/ATAD2/TACC3 |
| BP | mitotic cytokinesis | 3.00E-10 | 2.44E-10 | NUSAP1/KIF20A/ANLN/CEP55/KIF4A/KIF23/RACGAP1/CDT1/STMN1/ECT2 |
| BP | cytokinesis | 4.10E-10 | 3.33E-10 | NUSAP1/KIF20A/ANLN/CEP55/AURKA/KIF4A/PRC1/KIF23/RACGAP1/CDT1/STMN1/ECT2/E2F8 |
| BP | negative regulation of cell cycle process | 5.07E-10 | 4.12E-10 | CENPF/RRM2/NEK2/CDC20/PTTG1/CCNB1/CDK1/AURKA/DTL/NDC80/MAD2L1/BUB1B/TRIP13/CDT1/TTK/EZH2/E2F8 |
| BP | spindle assembly | 6.76E-10 | 5.49E-10 | NEK2/TPX2/BIRC5/CDC20/AURKA/KIF4A/KIF11/ASPM/KIF23/RACGAP1/SPAG5 |
| BP | negative regulation of chromosome organization | 9.98E-10 | 8.10E-10 | TOP2A/CENPF/CDC20/PTTG1/CCNB1/NDC80/MAD2L1/BUB1B/TRIP13/CDT1/TTK/ATAD2 |
| BP | negative regulation of mitotic cell cycle | 1.86E-09 | 1.51E-09 | TOP2A/CENPF/CDC20/PTTG1/ZWINT/CCNB1/CDK1/AURKA/NDC80/MAD2L1/BUB1B/TRIP13/CDT1/TTK/EZH2/E2F8 |
| BP | cytoskeleton-dependent cytokinesis | 6.57E-09 | 5.33E-09 | NUSAP1/KIF20A/ANLN/CEP55/KIF4A/KIF23/RACGAP1/CDT1/STMN1/ECT2 |
| BP | negative regulation of cell cycle phase transition | 8.20E-09 | 6.66E-09 | CENPF/CDC20/CCNB1/CDK1/AURKA/DTL/NDC80/MAD2L1/BUB1B/TRIP13/CDT1/TTK/EZH2/E2F8 |
| BP | anaphase-promoting complex-dependent catabolic process | 2.04E-08 | 1.65E-08 | UBE2C/CDC20/PTTG1/CCNB1/CDK1/AURKA/MAD2L1/BUB1B/UBE2S |
| BP | mitotic spindle assembly | 2.36E-08 | 1.92E-08 | NEK2/TPX2/BIRC5/CDC20/KIF4A/KIF11/KIF23/RACGAP1 |
| BP | cell cycle G1/S phase transition | 3.26E-08 | 2.65E-08 | RRM2/CCNB1/INHBA/CDK1/AURKA/CDKN3/IQGAP3/FAM83D/TYMS/CCNA2/CDT1/CCNE2/EZH2/E2F8 |
| BP | negative regulation of mitotic cell cycle phase transition | 3.49E-08 | 2.83E-08 | CENPF/CDC20/CCNB1/CDK1/AURKA/NDC80/MAD2L1/BUB1B/TRIP13/CDT1/TTK/EZH2/E2F8 |
| BP | meiotic nuclear division | 8.75E-08 | 7.11E-08 | TOP2A/CDC20/PTTG1/CKS2/AURKA/NUF2/ASPM/BUB1B/TRIP13/TTK/CCNE2 |
| BP | regulation of attachment of spindle microtubules to kinetochore | 1.03E-07 | 8.35E-08 | NEK2/CCNB1/RACGAP1/SPAG5/ECT2 |
| BP | mitotic metaphase plate congression | 1.19E-07 | 9.65E-08 | CCNB1/NUF2/KIF2C/CDCA8/NDC80/CDT1/PSRC1 |
| BP | meiotic cell cycle process | 2.12E-07 | 1.72E-07 | TOP2A/CDC20/PTTG1/CKS2/AURKA/NUF2/ASPM/BUB1B/TRIP13/TTK/CCNE2 |
| BP | G2/M transition of mitotic cell cycle | 3.40E-07 | 2.76E-07 | CENPF/NEK2/TPX2/CCNB2/CCNB1/FOXM1/CDK1/MELK/AURKA/DTL/HMMR/CCNA2 |
| BP | meiotic cell cycle | 3.67E-07 | 2.98E-07 | TOP2A/NEK2/CDC20/PTTG1/CKS2/AURKA/NUF2/ASPM/BUB1B/TRIP13/TTK/CCNE2 |
| BP | positive regulation of mitotic cell cycle | 6.40E-07 | 5.20E-07 | UBE2C/NUSAP1/CCNB1/CDK1/AURKA/DTL/DLGAP5/NDC80/MAD2L1/CDT1 |
| BP | cell cycle G2/M phase transition | 7.39E-07 | 6.01E-07 | CENPF/NEK2/TPX2/CCNB2/CCNB1/FOXM1/CDK1/MELK/AURKA/DTL/HMMR/CCNA2 |
| BP | negative regulation of organelle organization | 9.98E-07 | 8.11E-07 | TOP2A/CENPF/NEK2/CDC20/PTTG1/CCNB1/NDC80/MAD2L1/BUB1B/TRIP13/CDT1/TTK/ATAD2/STMN1 |
| BP | G1/S transition of mitotic cell cycle | 1.21E-06 | 9.86E-07 | RRM2/CCNB1/INHBA/CDK1/AURKA/CDKN3/IQGAP3/TYMS/CDT1/CCNE2/EZH2/E2F8 |
| BP | cytokinetic process | 1.74E-06 | 1.41E-06 | KIF20A/ANLN/CEP55/KIF23/RACGAP1/ECT2 |
| BP | kinetochore organization | 2.18E-06 | 1.77E-06 | CENPF/NUF2/NDC80/CDT1/CENPK |
| BP | positive regulation of cell cycle phase transition | 3.16E-06 | 2.57E-06 | UBE2C/CCNB1/CDK1/DTL/DLGAP5/FAM83D/CDT1/EZH2 |
| BP | spindle elongation | 4.27E-06 | 3.47E-06 | KIF4A/PRC1/KIF23/RACGAP1 |
| BP | DNA conformation change | 6.32E-06 | 5.14E-06 | TOP2A/NUSAP1/CCNB1/CDK1/CENPM/HMGB3/NCAPG/CENPU/HJURP/GINS1/GINS2/CENPK |
| BP | DNA replication | 8.31E-06 | 6.75E-06 | RRM2/CDK1/DTL/RMI2/CCNA2/GINS1/CDT1/GINS2/CCNE2/RNASEH2A/E2F8 |
| BP | regulation of cell division | 9.11E-06 | 7.40E-06 | KIF20A/AURKA/ASPM/PRC1/KIF23/RACGAP1/ECT2/KIF18B/E2F8 |
| BP | protein localization to cytoskeleton | 1.31E-05 | 1.06E-05 | ANLN/AURKA/FAM83D/TTK/SPAG5/STIL |
| BP | attachment of mitotic spindle microtubules to kinetochore | 1.34E-05 | 1.09E-05 | NUF2/KIF2C/NDC80/CDT1 |
| BP | regulation of cytokinesis | 1.37E-05 | 1.11E-05 | KIF20A/AURKA/PRC1/KIF23/RACGAP1/ECT2/E2F8 |
| BP | meiotic chromosome segregation | 1.46E-05 | 1.18E-05 | TOP2A/PTTG1/NUF2/BUB1B/TRIP13/TTK/CCNE2 |
| BP | regulation of spindle checkpoint | 1.76E-05 | 1.43E-05 | CCNB1/NDC80/MAD2L1/CDT1 |
| BP | regulation of mitotic cell cycle spindle assembly checkpoint | 1.76E-05 | 1.43E-05 | CCNB1/NDC80/MAD2L1/CDT1 |
| BP | regulation of mitotic spindle checkpoint | 1.76E-05 | 1.43E-05 | CCNB1/NDC80/MAD2L1/CDT1 |
| BP | regulation of microtubule cytoskeleton organization | 1.94E-05 | 1.57E-05 | NEK2/TPX2/AURKA/KIF11/SPAG5/STMN1/TACC3/STIL/PSRC1 |
| BP | regulation of cyclin-dependent protein serine/threonine kinase activity | 3.21E-05 | 2.61E-05 | CCNB2/CKS2/CCNB1/CDKN3/CCNA2/CCNE2/PSRC1 |
| BP | protein localization to kinetochore | 3.99E-05 | 3.24E-05 | CDK1/NDC80/CDT1/TTK |
| BP | regulation of cyclin-dependent protein kinase activity | 4.06E-05 | 3.30E-05 | CCNB2/CKS2/CCNB1/CDKN3/CCNA2/CCNE2/PSRC1 |
| BP | DNA packaging | 5.05E-05 | 4.10E-05 | TOP2A/NUSAP1/CCNB1/CDK1/CENPM/NCAPG/CENPU/HJURP/CENPK |
| BP | regulation of microtubule-based process | 6.78E-05 | 5.51E-05 | NEK2/TPX2/AURKA/KIF11/SPAG5/STMN1/TACC3/STIL/PSRC1 |
| BP | protein localization to chromosome, centromeric region | 9.43E-05 | 7.66E-05 | CDK1/NDC80/CDT1/TTK |
| BP | regulation of ubiquitin protein ligase activity | 0.000113097 | 9.19E-05 | UBE2C/CDC20/MAD2L1/UBE2S |
| BP | chromosome condensation | 0.000113097 | 9.19E-05 | TOP2A/NUSAP1/CCNB1/CDK1/NCAPG |
| BP | protein localization to microtubule cytoskeleton | 0.000152503 | 0.000123889 | AURKA/FAM83D/TTK/SPAG5/STIL |
| BP | female gamete generation | 0.000196371 | 0.000159526 | TOP2A/CCNB1/INHBA/AURKA/ASPM/TRIP13/TTK |
| BP | positive regulation of mitotic nuclear division | 0.000199545 | 0.000162105 | UBE2C/NUSAP1/AURKA/DLGAP5/CDT1 |
| BP | positive regulation of mitotic cell cycle phase transition | 0.000205167 | 0.000166672 | UBE2C/CCNB1/CDK1/DTL/DLGAP5/CDT1 |
| BP | regulation of G2/M transition of mitotic cell cycle | 0.000240918 | 0.000195715 | CENPF/NEK2/TPX2/CCNB1/CDK1/AURKA/DTL/HMMR |
| BP | centromere complex assembly | 0.000254097 | 0.000206421 | CENPF/CENPM/CENPU/HJURP/CENPK |
| BP | spindle midzone assembly | 0.000331965 | 0.000269679 | KIF4A/KIF23/RACGAP1 |
| BP | regulation of cell cycle G2/M phase transition | 0.000423037 | 0.000343663 | CENPF/NEK2/TPX2/CCNB1/CDK1/AURKA/DTL/HMMR |
| BP | female meiotic nuclear division | 0.000423037 | 0.000343663 | TOP2A/AURKA/TRIP13/TTK |
| BP | DNA integrity checkpoint | 0.000459175 | 0.00037302 | TOP2A/CCNB1/CDK1/AURKA/DTL/CDT1/E2F8 |
| BP | regulation of cell cycle checkpoint | 0.000472059 | 0.000383487 | CCNB1/NDC80/MAD2L1/CDT1 |
| BP | positive regulation of nuclear division | 0.000536032 | 0.000435456 | UBE2C/NUSAP1/AURKA/DLGAP5/CDT1 |
| BP | mitotic nuclear envelope disassembly | 0.000563816 | 0.000458028 | CCNB2/CCNB1/CDK1 |
| BP | positive regulation of ubiquitin protein ligase activity | 0.000563816 | 0.000458028 | UBE2C/CDC20/UBE2S |
| BP | meiosis I | 0.000664252 | 0.000539619 | TOP2A/PTTG1/CKS2/AURKA/TRIP13/CCNE2 |
| BP | meiosis I cell cycle process | 0.000818463 | 0.000664895 | TOP2A/PTTG1/CKS2/AURKA/TRIP13/CCNE2 |
| BP | animal organ regeneration | 0.000818463 | 0.000664895 | CDK1/AURKA/TYMS/CCNA2/EZH2 |
| BP | signal transduction involved in DNA integrity checkpoint | 0.000818463 | 0.000664895 | CCNB1/CDK1/AURKA/DTL/E2F8 |
| BP | signal transduction involved in DNA damage checkpoint | 0.000818463 | 0.000664895 | CCNB1/CDK1/AURKA/DTL/E2F8 |
| BP | signal transduction involved in cell cycle checkpoint | 0.000860386 | 0.000698952 | CCNB1/CDK1/AURKA/DTL/E2F8 |
| BP | histone phosphorylation | 0.000860386 | 0.000698952 | CCNB1/CDK1/AURKA/CCNA2 |
| BP | mitotic centrosome separation | 0.000860386 | 0.000698952 | NEK2/AURKA/KIF11 |
| BP | centrosome separation | 0.001061815 | 0.000862587 | NEK2/AURKA/KIF11 |
| BP | regulation of spindle organization | 0.001135855 | 0.000922735 | TPX2/SPAG5/TACC3/PSRC1 |
| BP | membrane disassembly | 0.00126847 | 0.001030468 | CCNB2/CCNB1/CDK1 |
| BP | nuclear envelope disassembly | 0.00126847 | 0.001030468 | CCNB2/CCNB1/CDK1 |
| BP | protein localization to chromosome | 0.001326654 | 0.001077735 | CDK1/NDC80/CDT1/TTK/EZH2 |
| BP | cortical actin cytoskeleton organization | 0.001326654 | 0.001077735 | ANLN/KIF23/RACGAP1/ECT2 |
| BP | signal transduction in response to DNA damage | 0.001386881 | 0.001126661 | CCNB1/FOXM1/CDK1/AURKA/DTL/E2F8 |
| BP | CENP-A containing nucleosome assembly | 0.001417658 | 0.001151663 | CENPM/CENPU/HJURP/CENPK |
| BP | CENP-A containing chromatin organization | 0.001417658 | 0.001151663 | CENPM/CENPU/HJURP/CENPK |
| BP | endodermal cell differentiation | 0.00153684 | 0.001248483 | COL11A1/MMP9/FN1/INHBA |
| BP | proteasome-mediated ubiquitin-dependent protein catabolic process | 0.001559307 | 0.001266735 | UBE2C/CDC20/PTTG1/CCNB1/PBK/CDK1/AURKA/MAD2L1/BUB1B/UBE2S |
| BP | retrograde vesicle-mediated transport, Golgi to ER | 0.001578807 | 0.001282576 | KIF4A/KIF2C/KIF11/KIF23/RACGAP1 |
| BP | regeneration | 0.001598598 | 0.001298654 | CCNB1/CDK1/AURKA/SPP1/TYMS/CCNA2/EZH2 |
| BP | cortical cytoskeleton organization | 0.001890036 | 0.00153541 | ANLN/KIF23/RACGAP1/ECT2 |
| BP | chromatin remodeling at centromere | 0.001890036 | 0.00153541 | CENPM/CENPU/HJURP/CENPK |
| BP | spindle localization | 0.001890036 | 0.00153541 | NUSAP1/NDC80/ASPM/MAD2L1 |
| BP | regulation of meiotic cell cycle | 0.002033629 | 0.001652061 | CDC20/ASPM/TRIP13/TTK |
| BP | regulation of reproductive process | 0.002120577 | 0.001722695 | CDC20/INHBA/AURKA/ASPM/TRIP13/TTK |
| BP | endoderm formation | 0.002167954 | 0.001761182 | COL11A1/MMP9/FN1/INHBA |
| BP | DNA strand elongation involved in DNA replication | 0.002237351 | 0.001817558 | GINS1/GINS2/RNASEH2A |
| BP | microtubule-based movement | 0.002298888 | 0.001867549 | KIF20A/KIF4A/KIF2C/KIF11/DLGAP5/KIF23/RACGAP1/KIF18B |
| BP | regulation of ubiquitin-protein transferase activity | 0.002471103 | 0.002007451 | UBE2C/CDC20/MAD2L1/UBE2S |
| BP | DNA-dependent DNA replication | 0.002635276 | 0.002140821 | GINS1/CDT1/GINS2/CCNE2/RNASEH2A/E2F8 |
| BP | antigen processing and presentation of exogenous peptide antigen via MHC class II | 0.002666492 | 0.00216618 | KIF4A/KIF2C/KIF11/KIF23/RACGAP1 |
| BP | DNA replication-independent nucleosome assembly | 0.002800355 | 0.002274926 | CENPM/CENPU/HJURP/CENPK |
| BP | DNA replication-independent nucleosome organization | 0.002985658 | 0.002425461 | CENPM/CENPU/HJURP/CENPK |
| BP | antigen processing and presentation of peptide antigen via MHC class II | 0.003004071 | 0.002440419 | KIF4A/KIF2C/KIF11/KIF23/RACGAP1 |
| BP | antigen processing and presentation of peptide or polysaccharide antigen via MHC class II | 0.003122927 | 0.002536974 | KIF4A/KIF2C/KIF11/KIF23/RACGAP1 |
| BP | DNA damage response, signal transduction by p53 class mediator resulting in cell cycle arrest | 0.003134475 | 0.002546355 | CCNB1/CDK1/AURKA/E2F8 |
| BP | signal transduction involved in mitotic G1 DNA damage checkpoint | 0.003310397 | 0.002689269 | CCNB1/CDK1/AURKA/E2F8 |
| BP | intracellular signal transduction involved in G1 DNA damage checkpoint | 0.003310397 | 0.002689269 | CCNB1/CDK1/AURKA/E2F8 |
| BP | regulation of centrosome cycle | 0.003516236 | 0.002856487 | NEK2/AURKA/KIF11/STIL |
| BP | mitotic DNA integrity checkpoint | 0.003606512 | 0.002929824 | TOP2A/CCNB1/CDK1/AURKA/E2F8 |
| BP | signal transduction involved in mitotic cell cycle checkpoint | 0.003642822 | 0.002959322 | CCNB1/CDK1/AURKA/E2F8 |
| BP | signal transduction involved in mitotic DNA damage checkpoint | 0.003642822 | 0.002959322 | CCNB1/CDK1/AURKA/E2F8 |
| BP | signal transduction involved in mitotic DNA integrity checkpoint | 0.003642822 | 0.002959322 | CCNB1/CDK1/AURKA/E2F8 |
| BP | proteasomal protein catabolic process | 0.003642822 | 0.002959322 | UBE2C/CDC20/PTTG1/CCNB1/PBK/CDK1/AURKA/MAD2L1/BUB1B/UBE2S |
| BP | DNA damage response, signal transduction by p53 class mediator | 0.003642822 | 0.002959322 | CCNB1/FOXM1/CDK1/AURKA/E2F8 |
| BP | regulation of cell cycle arrest | 0.003778072 | 0.003069194 | CCNB1/FOXM1/CDK1/AURKA/E2F8 |
| BP | histone exchange | 0.003798924 | 0.003086134 | CENPM/CENPU/HJURP/CENPK |
| BP | cell cycle arrest | 0.003934237 | 0.003196059 | CCNB1/FOXM1/INHBA/CDK1/AURKA/CDKN3/E2F8 |
| BP | oocyte maturation | 0.004277944 | 0.003475276 | CCNB1/AURKA/TRIP13 |
| BP | mitotic G1 DNA damage checkpoint | 0.004465606 | 0.003627727 | CCNB1/CDK1/AURKA/E2F8 |
| BP | mitotic G1/S transition checkpoint | 0.004465606 | 0.003627727 | CCNB1/CDK1/AURKA/E2F8 |
| BP | positive regulation of G2/M transition of mitotic cell cycle | 0.004654687 | 0.003781331 | CCNB1/CDK1/DTL |
| BP | DNA strand elongation | 0.004654687 | 0.003781331 | GINS1/GINS2/RNASEH2A |
| BP | G1 DNA damage checkpoint | 0.004654687 | 0.003781331 | CCNB1/CDK1/AURKA/E2F8 |
| BP | response to gonadotropin | 0.005085971 | 0.004131693 | GJB2/INHBA/CCNA2 |
| BP | regulation of B cell differentiation | 0.005085971 | 0.004131693 | INHBA/HMGB3/SLAMF8 |
| BP | positive regulation of chromosome segregation | 0.005085971 | 0.004131693 | CCNB1/DLGAP5/CDT1 |
| BP | regulation of transcription involved in G1/S transition of mitotic cell cycle | 0.005581856 | 0.004534536 | RRM2/TYMS/CDT1 |
| BP | centrosome localization | 0.005581856 | 0.004534536 | AURKA/ASPM/MAD2L1 |
| BP | microtubule organizing center localization | 0.006031704 | 0.004899979 | AURKA/ASPM/MAD2L1 |
| BP | protein K11-linked ubiquitination | 0.006031704 | 0.004899979 | UBE2C/UBE2T/UBE2S |
| BP | liver regeneration | 0.006031704 | 0.004899979 | AURKA/TYMS/EZH2 |
| BP | positive regulation of cell cycle G2/M phase transition | 0.006031704 | 0.004899979 | CCNB1/CDK1/DTL |
| BP | centrosome cycle | 0.006365081 | 0.005170805 | NEK2/CDK1/AURKA/KIF11/STIL |
| BP | negative regulation of G1/S transition of mitotic cell cycle | 0.00642429 | 0.005218904 | CCNB1/CDK1/AURKA/EZH2/E2F8 |
| BP | exit from mitosis | 0.00642429 | 0.005218904 | UBE2C/ANLN/UBE2S |
| BP | regulation of meiotic nuclear division | 0.00642429 | 0.005218904 | CDC20/TRIP13/TTK |
| BP | positive regulation of cyclin-dependent protein serine/threonine kinase activity | 0.00642429 | 0.005218904 | CKS2/CCNB1/PSRC1 |
| BP | protein localization to centrosome | 0.00642429 | 0.005218904 | AURKA/SPAG5/STIL |
| BP | signal transduction by p53 class mediator | 0.006980964 | 0.00567113 | TPX2/CCNB1/FOXM1/CDK1/AURKA/RMI2/E2F8 |
| BP | protein localization to microtubule organizing center | 0.006980964 | 0.00567113 | AURKA/SPAG5/STIL |
| BP | DNA biosynthetic process | 0.007519419 | 0.006108555 | CENPF/NEK2/TK1/DTL/ISG15/TYMS |
| BP | establishment of mitotic spindle localization | 0.007521345 | 0.00611012 | NUSAP1/NDC80/MAD2L1 |
| BP | positive regulation of ubiquitin-protein transferase activity | 0.007521345 | 0.00611012 | UBE2C/CDC20/UBE2S |
| BP | negative regulation of cell cycle G1/S phase transition | 0.007666096 | 0.006227711 | CCNB1/CDK1/AURKA/EZH2/E2F8 |
| BP | endoderm development | 0.007857357 | 0.006383086 | COL11A1/MMP9/FN1/INHBA |
| BP | microtubule organizing center organization | 0.008117754 | 0.006594625 | NEK2/CDK1/AURKA/KIF11/STIL |
| BP | regulation of cell cycle G1/S phase transition | 0.008507837 | 0.006911517 | CCNB1/CDK1/AURKA/FAM83D/EZH2/E2F8 |
| BP | positive regulation of cyclin-dependent protein kinase activity | 0.00870943 | 0.007075285 | CKS2/CCNB1/PSRC1 |
| BP | regulation of protein ubiquitination | 0.008856671 | 0.007194899 | UBE2C/CDC20/ISG15/MAD2L1/UBE2S/FANCI |
| BP | positive regulation of DNA replication | 0.00936226 | 0.007605625 | CDK1/CDT1/E2F8 |
| BP | regulation of mitotic spindle organization | 0.01005744 | 0.008170369 | TPX2/TACC3/PSRC1 |
| BP | positive regulation of cell cycle arrest | 0.01005744 | 0.008170369 | CCNB1/CDK1/AURKA/E2F8 |
| BP | DNA damage checkpoint | 0.011501591 | 0.009343555 | CCNB1/CDK1/AURKA/DTL/E2F8 |
| BP | deoxyribonucleoside monophosphate biosynthetic process | 0.011618437 | 0.009438477 | TK1/TYMS |
| BP | positive regulation of metaphase/anaphase transition of cell cycle | 0.011618437 | 0.009438477 | DLGAP5/CDT1 |
| BP | positive regulation of oxidative phosphorylation | 0.011618437 | 0.009438477 | CCNB1/CDK1 |
| BP | oogenesis | 0.012209184 | 0.009918382 | CCNB1/AURKA/ASPM/TRIP13 |
| BP | positive regulation of cytokinesis | 0.012227412 | 0.009933191 | KIF23/RACGAP1/ECT2 |
| BP | ATP-dependent chromatin remodeling | 0.012607103 | 0.01024164 | CENPM/CENPU/HJURP/CENPK |
| BP | cellular response to oxidative stress | 0.012772407 | 0.010375928 | GJB2/MMP9/CDK1/MELK/CCNA2/ECT2/EZH2 |
| BP | histone-serine phosphorylation | 0.013588412 | 0.011038827 | CCNB1/AURKA |
| BP | negative regulation of meiotic nuclear division | 0.013588412 | 0.011038827 | TRIP13/TTK |
| BP | ventricular cardiac muscle cell development | 0.013588412 | 0.011038827 | CCNB1/CDK1 |
| BP | positive regulation of chromosome separation | 0.013588412 | 0.011038827 | DLGAP5/CDT1 |
| BP | establishment of spindle localization | 0.01456926 | 0.011835638 | NUSAP1/NDC80/MAD2L1 |
| BP | regulation of protein modification by small protein conjugation or removal | 0.0153084 | 0.012436094 | UBE2C/CDC20/ISG15/MAD2L1/UBE2S/FANCI |
| BP | microtubule depolymerization | 0.015420525 | 0.012527181 | KIF2C/STMN1/KIF18B |
| BP | positive regulation of DNA-dependent DNA replication | 0.015931448 | 0.01294224 | CDT1/E2F8 |
| BP | mitotic DNA damage checkpoint | 0.017159759 | 0.013940084 | CCNB1/CDK1/AURKA/E2F8 |
| BP | oocyte development | 0.017280047 | 0.014037802 | CCNB1/AURKA/TRIP13 |
| BP | collagen catabolic process | 0.018296124 | 0.014863234 | MMP11/MMP9/MMP1 |
| BP | meiotic spindle organization | 0.018315234 | 0.014878758 | AURKA/ASPM |
| BP | deoxyribonucleoside monophosphate metabolic process | 0.018315234 | 0.014878758 | TK1/TYMS |
| BP | cellular response to reactive oxygen species | 0.019756498 | 0.016049599 | MMP9/CDK1/CCNA2/ECT2/EZH2 |
| BP | protein-DNA complex assembly | 0.020909451 | 0.016986223 | CENPF/CENPM/CENPU/HJURP/CDT1/CENPK |
| BP | regulation of DNA metabolic process | 0.021530096 | 0.017490417 | NEK2/FOXM1/CDK1/RMI2/CCNA2/CDT1/RAD51AP1/E2F8 |
| BP | oocyte differentiation | 0.02237339 | 0.018175485 | CCNB1/AURKA/TRIP13 |
| BP | positive regulation of fibroblast proliferation | 0.02237339 | 0.018175485 | FN1/CCNB1/CCNA2 |
| BP | antigen processing and presentation of exogenous peptide antigen | 0.023015686 | 0.018697267 | KIF4A/KIF2C/KIF11/KIF23/RACGAP1 |
| BP | nuclear envelope organization | 0.023430978 | 0.019034638 | CCNB2/CCNB1/CDK1 |
| BP | deoxyribonucleotide biosynthetic process | 0.02358257 | 0.019157787 | RRM2/TYMS |
| BP | regulation of DNA replication | 0.023836382 | 0.019363976 | CDK1/CCNA2/CDT1/E2F8 |
| BP | interstrand cross-link repair | 0.024401417 | 0.019822994 | UBE2T/RAD51AP1/FANCI |
| BP | kinetochore assembly | 0.026137516 | 0.02123335 | CENPF/CENPK |
| BP | negative regulation of meiotic cell cycle | 0.026137516 | 0.02123335 | TRIP13/TTK |
| BP | ventricular cardiac muscle cell differentiation | 0.026137516 | 0.02123335 | CCNB1/CDK1 |
| BP | chromatin remodeling | 0.026137516 | 0.02123335 | CENPM/HMGB3/CENPU/HJURP/CENPK |
| BP | antigen processing and presentation of exogenous antigen | 0.026137516 | 0.02123335 | KIF4A/KIF2C/KIF11/KIF23/RACGAP1 |
| BP | microtubule polymerization or depolymerization | 0.026323777 | 0.021384663 | KIF2C/STMN1/KIF18B/PSRC1 |
| BP | regulation of G1/S transition of mitotic cell cycle | 0.027142533 | 0.022049796 | CCNB1/CDK1/AURKA/EZH2/E2F8 |
| BP | cellular process involved in reproduction in multicellular organism | 0.028112282 | 0.022837592 | TOP2A/CCNB1/INHBA/AURKA/ASPM/TRIP13/TTK |
| BP | homologous chromosome segregation | 0.028502123 | 0.023154287 | PTTG1/TRIP13/CCNE2 |
| BP | mitotic chromosome condensation | 0.028502123 | 0.023154287 | NUSAP1/NCAPG |
| BP | midbody abscission | 0.028502123 | 0.023154287 | KIF20A/CEP55 |
| BP | positive regulation of mitotic sister chromatid segregation | 0.028502123 | 0.023154287 | DLGAP5/CDT1 |
| BP | antigen processing and presentation of peptide antigen | 0.02963255 | 0.024072613 | KIF4A/KIF2C/KIF11/KIF23/RACGAP1 |
| BP | positive regulation of protein ubiquitination | 0.029717763 | 0.024141837 | UBE2C/CDC20/UBE2S/FANCI |
| BP | DNA geometric change | 0.031277631 | 0.025409028 | TOP2A/HMGB3/GINS1/GINS2 |
| BP | negative regulation of cell division | 0.031277631 | 0.025409028 | ASPM/E2F8 |
| BP | cellular response to gonadotropin stimulus | 0.031277631 | 0.025409028 | INHBA/CCNA2 |
| BP | extracellular matrix organization | 0.031760851 | 0.025801582 | COL10A1/MMP11/COL11A1/MMP9/FN1/SPP1/MMP1 |
| BP | formation of primary germ layer | 0.032785812 | 0.02663423 | COL11A1/MMP9/FN1/INHBA |
| BP | regulation of binding | 0.033875117 | 0.02751915 | NEK2/MMP9/AURKA/HJURP/CTHRC1/CDT1/STMN1 |
| BP | regulation of exit from mitosis | 0.034255155 | 0.027827882 | UBE2C/ANLN |
| BP | regulation of DNA binding | 0.035304519 | 0.028680354 | NEK2/MMP9/HJURP/CDT1 |
| BP | actomyosin structure organization | 0.035304519 | 0.028680354 | ANLN/KIF23/RACGAP1/STMN1/ECT2 |
| BP | protein-DNA complex subunit organization | 0.037652998 | 0.030588189 | CENPF/CENPM/CENPU/HJURP/CDT1/CENPK |
| BP | chromosome organization involved in meiotic cell cycle | 0.038953354 | 0.03164456 | BUB1B/TRIP13/CCNE2 |
| BP | DNA recombination | 0.039886694 | 0.032402778 | TOP2A/RMI2/HMGB3/TRIP13/GINS2/RAD51AP1 |
| BP | protein monoubiquitination | 0.040317338 | 0.032752621 | UBE2T/UHRF1/DTL |
| BP | rhythmic process | 0.041535509 | 0.033742227 | TOP2A/INHBA/CDK1/DTL/TYMS/EZH2 |
| BP | liver development | 0.046031414 | 0.037394568 | AURKA/TYMS/EZH2/E2F8 |
| BP | positive regulation of protein modification by small protein conjugation or removal | 0.046031414 | 0.037394568 | UBE2C/CDC20/UBE2S/FANCI |
| BP | cell cycle DNA replication | 0.048575657 | 0.039461436 | GINS1/CDT1/E2F8 |
| BP | hepaticobiliary system development | 0.049286685 | 0.040039055 | AURKA/TYMS/EZH2/E2F8 |
| CC | spindle | 2.76E-23 | 2.01E-23 | CENPF/NEK2/TPX2/BIRC5/CDC20/NUSAP1/KIF20A/CCNB1/CDK1/AURKA/KIF4A/KIF11/DLGAP5/CDCA8/ASPM/FAM83D/PRC1/MAD2L1/BUB1B/KIF23/RACGAP1/TTK/SPAG5/TACC3/ECT2/KIF18B/SHCBP1/PSRC1 |
| CC | condensed chromosome, centromeric region | 1.00E-19 | 7.33E-20 | CENPF/NEK2/BIRC5/ZWINT/CCNB1/AURKA/NUF2/KIF2C/CENPM/NDC80/NCAPG/CENPU/HJURP/MAD2L1/BUB1B/CDT1/SPAG5/CENPK |
| CC | chromosome, centromeric region | 9.35E-19 | 6.82E-19 | CENPF/NEK2/BIRC5/ZWINT/CCNB1/AURKA/NUF2/KIF2C/CENPM/CDCA8/NDC80/NCAPG/CENPU/HJURP/MAD2L1/BUB1B/CDT1/TTK/SPAG5/CENPK |
| CC | condensed chromosome kinetochore | 1.03E-17 | 7.49E-18 | CENPF/NEK2/BIRC5/ZWINT/CCNB1/NUF2/KIF2C/CENPM/NDC80/CENPU/HJURP/MAD2L1/BUB1B/CDT1/SPAG5/CENPK |
| CC | condensed chromosome | 1.03E-17 | 7.49E-18 | TOP2A/CENPF/NEK2/BIRC5/MKI67/ZWINT/CCNB1/AURKA/NUF2/KIF2C/CENPM/NDC80/NCAPG/CENPU/HJURP/MAD2L1/BUB1B/CDT1/SPAG5/CENPK |
| CC | kinetochore | 1.43E-17 | 1.04E-17 | CENPF/NEK2/BIRC5/ZWINT/CCNB1/NUF2/KIF2C/CENPM/NDC80/CENPU/HJURP/MAD2L1/BUB1B/CDT1/TTK/SPAG5/CENPK |
| CC | midbody | 3.50E-17 | 2.55E-17 | CENPF/NEK2/BIRC5/KIF20A/CDK1/ANLN/CEP55/AURKA/KIF4A/CDCA8/ASPM/PRC1/KIF23/RACGAP1/SPAG5/ECT2/SHCBP1/PSRC1 |
| CC | chromosomal region | 1.81E-16 | 1.32E-16 | CENPF/NEK2/BIRC5/ZWINT/CCNB1/CDK1/AURKA/NUF2/KIF2C/CENPM/CDCA8/NDC80/NCAPG/CENPU/HJURP/MAD2L1/BUB1B/CDT1/TTK/SPAG5/EZH2/CENPK |
| CC | mitotic spindle | 1.08E-14 | 7.89E-15 | TPX2/NUSAP1/CDK1/AURKA/KIF11/ASPM/FAM83D/MAD2L1/KIF23/RACGAP1/SPAG5/TACC3/ECT2/KIF18B |
| CC | spindle pole | 1.47E-13 | 1.07E-13 | CENPF/NEK2/TPX2/CDC20/CCNB1/AURKA/KIF11/DLGAP5/ASPM/FAM83D/PRC1/MAD2L1/SPAG5/TACC3/PSRC1 |
| CC | microtubule | 9.32E-12 | 6.79E-12 | NEK2/TPX2/BIRC5/NUSAP1/KIF20A/CDK1/AURKA/KIF4A/KIF2C/KIF11/ASPM/PRC1/KIF23/RACGAP1/SPAG5/STMN1/KIF18B/KIF26B/PSRC1 |
| CC | spindle microtubule | 3.12E-10 | 2.28E-10 | BIRC5/NUSAP1/CDK1/AURKA/KIF4A/KIF11/PRC1/KIF18B/PSRC1 |
| CC | condensed nuclear chromosome, centromeric region | 4.91E-08 | 3.58E-08 | CCNB1/AURKA/NUF2/NDC80/BUB1B/CENPK |
| CC | microtubule associated complex | 9.28E-08 | 6.76E-08 | BIRC5/KIF20A/AURKA/KIF4A/KIF2C/KIF11/CDCA8/KIF23/KIF18B/KIF26B |
| CC | condensed nuclear chromosome kinetochore | 1.14E-07 | 8.33E-08 | CCNB1/NUF2/NDC80/BUB1B/CENPK |
| CC | kinesin complex | 1.56E-07 | 1.14E-07 | KIF20A/KIF4A/KIF2C/KIF11/KIF23/KIF18B/KIF26B |
| CC | cyclin-dependent protein kinase holoenzyme complex | 7.98E-07 | 5.82E-07 | CCNB2/CKS2/CCNB1/CDK1/CCNA2/CCNE2 |
| CC | intercellular bridge | 5.87E-06 | 4.28E-06 | TPX2/KIF20A/CEP55/KIF4A/CDCA8/KIF23 |
| CC | condensed chromosome outer kinetochore | 5.87E-06 | 4.28E-06 | CENPF/CCNB1/NDC80/BUB1B |
| CC | condensed nuclear chromosome | 7.22E-06 | 5.26E-06 | NEK2/CCNB1/AURKA/NUF2/NDC80/BUB1B/CENPK |
| CC | pronucleus | 7.22E-06 | 5.26E-06 | CENPF/AURKA/CCNA2/EZH2 |
| CC | anaphase-promoting complex | 2.95E-05 | 2.15E-05 | UBE2C/CDC20/BUB1B/UBE2S |
| CC | serine/threonine protein kinase complex | 5.00E-05 | 3.65E-05 | CCNB2/CKS2/CCNB1/CDK1/CCNA2/CCNE2 |
| CC | mitotic spindle pole | 7.73E-05 | 5.64E-05 | AURKA/ASPM/FAM83D/SPAG5 |
| CC | microtubule end | 9.96E-05 | 7.26E-05 | KIF2C/ASPM/SPAG5/KIF18B |
| CC | cell division site part | 0.00012593 | 9.18E-05 | KIF20A/ANLN/CEP55/RACGAP1/ECT2 |
| CC | protein kinase complex | 0.000145383 | 0.000106024 | CCNB2/CKS2/CCNB1/CDK1/CCNA2/CCNE2 |
| CC | cell division site | 0.000164288 | 0.000119811 | KIF20A/ANLN/CEP55/RACGAP1/ECT2 |
| CC | spindle midzone | 0.000164288 | 0.000119811 | AURKA/CDCA8/RACGAP1/KIF18B |
| CC | nuclear ubiquitin ligase complex | 0.00040734 | 0.000297063 | UBE2C/CDC20/BUB1B/UBE2S |
| CC | microtubule plus-end | 0.000773007 | 0.000563734 | KIF2C/SPAG5/KIF18B |
| CC | cullin-RING ubiquitin ligase complex | 0.000963792 | 0.000702869 | UBE2C/CDC20/CKS2/DTL/BUB1B/UBE2S |
| CC | cleavage furrow | 0.000974453 | 0.000710643 | KIF20A/CEP55/RACGAP1/ECT2 |
| CC | Flemming body | 0.001963624 | 0.00143202 | CEP55/KIF23/RACGAP1 |
| CC | contractile ring | 0.005373951 | 0.003919084 | ANLN/PRC1 |
| CC | meiotic spindle | 0.006363543 | 0.004640768 | AURKA/ASPM |
| CC | transferase complex, transferring phosphorus-containing groups | 0.010915848 | 0.007960646 | CCNB2/CKS2/CCNB1/CDK1/CCNA2/CCNE2 |
| CC | nuclear matrix | 0.011059308 | 0.008065268 | CENPF/UHRF1/LMNB1/KIF4A |
| CC | spindle pole centrosome | 0.011059308 | 0.008065268 | AURKA/DLGAP5 |
| CC | microtubule organizing center part | 0.011939971 | 0.008707513 | TOP2A/CEP55/AURKA/SPAG5/STIL |
| CC | ubiquitin ligase complex | 0.014956286 | 0.010907233 | UBE2C/CDC20/CKS2/DTL/BUB1B/UBE2S |
| CC | germ cell nucleus | 0.016493937 | 0.012028602 | AURKA/TRIP13 |
| CC | nuclear periphery | 0.018874676 | 0.013764814 | CENPF/UHRF1/LMNB1/KIF4A |
| CC | centriole | 0.022700141 | 0.016554626 | TOP2A/CEP55/AURKA/STIL |
| CC | collagen trimer | 0.038270684 | 0.027909821 | COL10A1/COL11A1/CTHRC1 |
| MF | microtubule binding | 2.86E-08 | 2.47E-08 | BIRC5/NUSAP1/KIF20A/KIF4A/KIF2C/KIF11/FAM83D/PRC1/KIF23/RACGAP1/SPAG5/KIF18B/KIF26B/PSRC1 |
| MF | tubulin binding | 8.85E-08 | 7.63E-08 | BIRC5/NUSAP1/KIF20A/KIF4A/KIF2C/KIF11/FAM83D/PRC1/KIF23/RACGAP1/SPAG5/STMN1/KIF18B/KIF26B/PSRC1 |
| MF | microtubule motor activity | 3.69E-05 | 3.19E-05 | KIF20A/KIF4A/KIF2C/KIF11/KIF23/KIF18B/KIF26B |
| MF | cyclin-dependent protein serine/threonine kinase regulator activity | 0.000460469 | 0.000397221 | CCNB2/CKS2/CCNB1/CCNA2/CCNE2 |
| MF | motor activity | 0.000559259 | 0.000482442 | KIF20A/KIF4A/KIF2C/KIF11/KIF23/KIF18B/KIF26B |
| MF | histone kinase activity | 0.004072671 | 0.003513267 | CCNB1/CDK1/AURKA |
| MF | protein kinase regulator activity | 0.017917209 | 0.01545618 | CXCL10/CCNB2/CKS2/CCNB1/CCNA2/CCNE2 |
| MF | protein C-terminus binding | 0.019144658 | 0.016515032 | TOP2A/CENPF/CDC20/MKI67/FN1/MAD2L1 |
| MF | kinase regulator activity | 0.026543863 | 0.022897915 | CXCL10/CCNB2/CKS2/CCNB1/CCNA2/CCNE2 |
| MF | ubiquitin conjugating enzyme activity | 0.026543863 | 0.022897915 | UBE2C/UBE2T/UBE2S |
| MF | ubiquitin-like protein conjugating enzyme activity | 0.026543863 | 0.022897915 | UBE2C/UBE2T/UBE2S |
| MF | ATPase activity | 0.026543863 | 0.022897915 | TOP2A/KIF20A/KIF4A/KIF2C/KIF11/KIF23/ATAD2/KIF18B |
| MF | CXCR chemokine receptor binding | 0.026543863 | 0.022897915 | CXCL10/CXCL11 |
| MF | kinesin binding | 0.026543863 | 0.022897915 | FAM83D/PRC1/KIF18B |
| MF | metalloendopeptidase activity | 0.040619998 | 0.035040615 | MMP11/MMP9/MMP1/ECE2 |
