# Supplementary material for: Identification of hub genes and small molecule therapeutic drugs related to breast cancer with comprehensive bioinformatics analysis
Source: PeerJ. 2020 Sep 29;8:e9946. doi: 10.7717/peerj.9946 (PMC7556247; doi:10.7717/peerj.9946)
Supplement: Supplemental Information 18 [file peerj-08-9946-s018.docx]

| **Group** | **Description** | **p.adjust** | **qvalue** | **geneID** |
| --- | --- | --- | --- | --- |
| BP | regulation of cellular response to growth factor stimulus | 0.000889032 | 0.000703258 | ZNF423/MMRN2/TGFBR2/CDKN1C/SPRY1/FGF2/CAV2/CAV1/SPRY2/TGFBR3/GPC3/TNMD/CHRDL1/CIDEA |
| BP | negative regulation of cellular response to growth factor stimulus | 0.001925756 | 0.001523345 | MMRN2/TGFBR2/SPRY1/CAV2/CAV1/SPRY2/TGFBR3/TNMD/CHRDL1/CIDEA |
| BP | temperature homeostasis | 0.001925756 | 0.001523345 | ZNF423/ADRB2/ADAMTS5/ALDH1A1/EDNRB/CAV1/G0S2/CD36/CIDEA/LEP |
| BP | retinoid metabolic process | 0.001925756 | 0.001523345 | ALDH1A1/AKR1C3/GPC3/ADH1C/GPIHBP1/LPL/RBP4/ADH1B |
| BP | cold-induced thermogenesis | 0.001925756 | 0.001523345 | ZNF423/ADRB2/ADAMTS5/ALDH1A1/CAV1/G0S2/CD36/CIDEA/LEP |
| BP | regulation of cold-induced thermogenesis | 0.001925756 | 0.001523345 | ZNF423/ADRB2/ADAMTS5/ALDH1A1/CAV1/G0S2/CD36/CIDEA/LEP |
| BP | regulation of vasculature development | 0.001925756 | 0.001523345 | PDGFD/SASH1/MMRN2/ISM1/TGFBR2/AQP1/HOXA5/FGF2/MEOX2/ENPP2/SPRY2/TNMD/PPARG/TMEM100/LEP |
| BP | diterpenoid metabolic process | 0.001925756 | 0.001523345 | ALDH1A1/AKR1C3/GPC3/ADH1C/GPIHBP1/LPL/RBP4/ADH1B |
| BP | multicellular organismal homeostasis | 0.001925756 | 0.001523345 | ZNF423/NDN/AQP1/LDB2/ADRB2/ADAMTS5/ALDH1A1/TNS2/EDNRB/CAV1/G0S2/PDK4/CD36/CIDEA/LEP/RBP4 |
| BP | adaptive thermogenesis | 0.002414176 | 0.001909703 | ZNF423/ADRB2/ADAMTS5/ALDH1A1/CAV1/G0S2/CD36/CIDEA/LEP |
| BP | primary alcohol metabolic process | 0.002414176 | 0.001909703 | ALDH1A1/AKR1C3/IGF1/ADH1C/RBP4/GPD1/ADH1B |
| BP | terpenoid metabolic process | 0.002414176 | 0.001909703 | ALDH1A1/AKR1C3/GPC3/ADH1C/GPIHBP1/LPL/RBP4/ADH1B |
| BP | response to acid chemical | 0.002414176 | 0.001909703 | PDGFD/PDGFRA/GSN/AQP1/AKR1C3/TGFBR3/CDO1/PPARG/PDK4/LPL/CD36/LEP/RBP4 |
| BP | retinol metabolic process | 0.005044195 | 0.003990147 | ALDH1A1/AKR1C3/ADH1C/RBP4/ADH1B |
| BP | lipid storage | 0.005044195 | 0.003990147 | CAV1/PPARG/LPL/CD36/CIDEA/LEP |
| BP | regulation of epithelial cell proliferation | 0.005044195 | 0.003990147 | CXCL12/HOXA5/CDKN1C/FGF2/CAV2/EDNRB/IGF1/CAV1/TGFBR3/GPC3/TNMD/PPARG/LEP |
| BP | connective tissue development | 0.005044195 | 0.003990147 | MAF/PDGFD/EFEMP1/TGFBR2/HOXA5/FGF2/MATN2/ID4/TNMD/ZBTB16/LEP |
| BP | isoprenoid metabolic process | 0.005044195 | 0.003990147 | ALDH1A1/AKR1C3/GPC3/ADH1C/GPIHBP1/LPL/RBP4/ADH1B |
| BP | regulation of angiogenesis | 0.005177014 | 0.004095212 | SASH1/MMRN2/ISM1/TGFBR2/AQP1/HOXA5/FGF2/MEOX2/ENPP2/SPRY2/TNMD/PPARG/LEP |
| BP | urogenital system development | 0.005177014 | 0.004095212 | PDGFD/PDGFRA/CDKN1C/SPRY1/FGF2/TNS2/MME/ID4/ADAMTS1/GPC3/ZBTB16/RBP4 |
| BP | hormone metabolic process | 0.005445617 | 0.004307687 | PDGFRA/CTSG/GHR/ALDH1A1/AKR1C3/MME/ADH1C/LEP/RBP4/ADH1B |
| BP | regulation of lipid storage | 0.00638854 | 0.005053574 | PPARG/LPL/CD36/CIDEA/LEP |
| BP | muscle cell proliferation | 0.00638854 | 0.005053574 | PDGFD/TGFBR2/FGF2/NDRG2/IGF1/ADAMTS1/OGN/TGFBR3/PPARG/RBP4 |
| BP | renal system development | 0.006465992 | 0.005114841 | PDGFD/PDGFRA/CDKN1C/SPRY1/FGF2/TNS2/MME/ADAMTS1/GPC3/ZBTB16/RBP4 |
| BP | ameboidal-type cell migration | 0.006465992 | 0.005114841 | AMOTL2/SASH1/SEMA6D/MMRN2/TGFBR2/TNS1/FGF2/EDNRB/AKAP12/SEMA3G/MEOX2/ENPP2/GPC3/PPARG |
| BP | regulation of developmental growth | 0.006465992 | 0.005114841 | DPYSL2/SEMA6D/CXCL12/GHR/TGFBR2/FGF2/ADRB2/IGF1/SEMA3G/TGFBR3/LEP/RBP4 |
| BP | extracellular structure organization | 0.009895849 | 0.007827986 | ECM2/PDGFRA/ABCA5/CTSG/JAM2/LAMA2/VWF/FGF2/ADAMTS5/DPT/GPIHBP1/LPL/CD36 |
| BP | response to fatty acid | 0.011273853 | 0.008918039 | AKR1C3/TGFBR3/PPARG/PDK4/LPL/CD36 |
| BP | epithelial cell proliferation | 0.011290938 | 0.008931554 | CXCL12/HOXA5/CDKN1C/FGF2/CAV2/EDNRB/IGF1/CAV1/TGFBR3/GPC3/TNMD/PPARG/LEP |
| BP | regulation of smooth muscle cell proliferation | 0.011290938 | 0.008931554 | PDGFD/TGFBR2/FGF2/NDRG2/IGF1/ADAMTS1/OGN/PPARG |
| BP | response to peptide hormone | 0.011290938 | 0.008931554 | IRS2/CXCL12/GHR/CAV2/EDNRB/CAV1/TGFBR3/CDO1/PPARG/PDK4/LPL/TIMP4/LEP |
| BP | regulation of macrophage derived foam cell differentiation | 0.011290938 | 0.008931554 | ABCA5/PPARG/LPL/CD36 |
| BP | smooth muscle cell proliferation | 0.011290938 | 0.008931554 | PDGFD/TGFBR2/FGF2/NDRG2/IGF1/ADAMTS1/OGN/PPARG |
| BP | ethanol oxidation | 0.011290938 | 0.008931554 | ALDH1A1/ADH1C/ADH1B |
| BP | regulation of sequestering of triglyceride | 0.011290938 | 0.008931554 | PPARG/LPL/CIDEA |
| BP | lung development | 0.011303527 | 0.008941512 | PDGFRA/TGFBR2/HOXA5/SPRY1/MME/SPRY2/GPC3/RBP4 |
| BP | respiratory tube development | 0.012780168 | 0.01010959 | PDGFRA/TGFBR2/HOXA5/SPRY1/MME/SPRY2/GPC3/RBP4 |
| BP | cardiac epithelial to mesenchymal transition | 0.012780168 | 0.01010959 | TGFBR2/SPRY1/TGFBR3/TMEM100 |
| BP | heart trabecula formation | 0.012780168 | 0.01010959 | ADAMTS1/TGFBR3/RBP4 |
| BP | kidney development | 0.012780168 | 0.01010959 | PDGFD/PDGFRA/CDKN1C/SPRY1/FGF2/TNS2/MME/ADAMTS1/GPC3/ZBTB16 |
| BP | lipid localization | 0.015538318 | 0.01229139 | GULP1/ABCA5/IRS2/CAV1/ABCA6/PPARG/ABCA9/LPL/CD36/CIDEA/LEP/RBP4 |
| BP | transmembrane receptor protein serine/threonine kinase signaling pathway | 0.017055606 | 0.013491621 | ZNF423/TGFBR2/CDKN1C/CAV2/CAV1/TGFBR3/GPC3/TMEM100/CHRDL1/CLDN5/CIDEA |
| BP | macrophage derived foam cell differentiation | 0.017055606 | 0.013491621 | ABCA5/PPARG/LPL/CD36 |
| BP | foam cell differentiation | 0.017055606 | 0.013491621 | ABCA5/PPARG/LPL/CD36 |
| BP | muscle organ development | 0.017055606 | 0.013491621 | LAMA2/TGFBR2/DMD/HLF/FGF2/CAV2/IGF1/MEOX2/CAV1/TGFBR3/FHL1/RBP4 |
| BP | regulation of lipid metabolic process | 0.017055606 | 0.013491621 | PDGFRA/IRS2/FGF2/AKR1C3/CAV1/GPAM/PPARG/G0S2/PDK4/CD36/CIDEA/LEP |
| BP | sequestering of triglyceride | 0.017055606 | 0.013491621 | PPARG/LPL/CIDEA |
| BP | regulation of transmembrane receptor protein serine/threonine kinase signaling pathway | 0.017070101 | 0.013503088 | ZNF423/TGFBR2/CDKN1C/CAV2/CAV1/TGFBR3/GPC3/CHRDL1/CIDEA |
| BP | regulation of wound healing | 0.01960499 | 0.01550828 | PDGFRA/PROS1/TGFBR2/FGF2/TFPI/CAV1/CD36 |
| BP | regulation of cholesterol storage | 0.01960499 | 0.01550828 | PPARG/LPL/CD36 |
| BP | respiratory system development | 0.02076038 | 0.016422236 | PDGFRA/TGFBR2/HOXA5/SPRY1/MME/SPRY2/GPC3/RBP4 |
| BP | organ growth | 0.024840652 | 0.019649884 | TGFBR2/FGF2/MATN2/IGF1/SPRY2/TGFBR3/LEP/RBP4 |
| BP | cholesterol storage | 0.026604572 | 0.02104521 | PPARG/LPL/CD36 |
| BP | cartilage development | 0.027567111 | 0.021806615 | MAF/EFEMP1/TGFBR2/HOXA5/FGF2/MATN2/ZBTB16/LEP |
| BP | cellular response to acid chemical | 0.027567111 | 0.021806615 | PDGFD/PDGFRA/AQP1/AKR1C3/PPARG/PDK4/LPL/LEP |
| BP | cellular response to peptide | 0.031057876 | 0.02456794 | IRS2/GHR/ADRB2/CAV2/IGF1/CAV1/PPARG/PDK4/LPL/CD36/LEP |
| BP | regulation of cardiac muscle tissue growth | 0.031057876 | 0.02456794 | TGFBR2/FGF2/IGF1/TGFBR3/RBP4 |
| BP | negative regulation of epithelial cell proliferation | 0.031057876 | 0.02456794 | CDKN1C/CAV2/CAV1/TGFBR3/GPC3/TNMD/PPARG |
| BP | regulation of blood coagulation | 0.031057876 | 0.02456794 | PDGFRA/PROS1/TFPI/CAV1/CD36 |
| BP | regulation of transforming growth factor beta receptor signaling pathway | 0.031057876 | 0.02456794 | TGFBR2/CDKN1C/CAV2/CAV1/TGFBR3/CIDEA |
| BP | striated muscle tissue development | 0.031057876 | 0.02456794 | PDGFRA/TGFBR2/HLF/FGF2/PGM5/CAV2/IGF1/MEOX2/CAV1/TGFBR3/RBP4 |
| BP | maintenance of location | 0.031057876 | 0.02456794 | ANK2/GSN/DMD/FGF2/CAV1/PPARG/LPL/CD36/CIDEA/LEP |
| BP | regulation of hemostasis | 0.031057876 | 0.02456794 | PDGFRA/PROS1/TFPI/CAV1/CD36 |
| BP | regulation of cellular response to transforming growth factor beta stimulus | 0.031057876 | 0.02456794 | TGFBR2/CDKN1C/CAV2/CAV1/TGFBR3/CIDEA |
| BP | negative regulation of transforming growth factor beta receptor signaling pathway | 0.031057876 | 0.02456794 | TGFBR2/CAV2/CAV1/TGFBR3/CIDEA |
| BP | positive regulation of ion transport | 0.031057876 | 0.02456794 | ANK2/CXCL12/SCN4B/DMD/ADRB2/CAV1/FHL1/FXYD1/LEP |
| BP | positive regulation of smooth muscle cell migration | 0.031057876 | 0.02456794 | PDGFD/SEMA6D/IGF1/ADAMTS1 |
| BP | negative regulation of cold-induced thermogenesis | 0.031057876 | 0.02456794 | ZNF423/ADAMTS5/ALDH1A1/CIDEA |
| BP | axon guidance | 0.031057876 | 0.02456794 | DPYSL2/SEMA6D/IRS2/CXCL12/LAMA2/ROBO4/CHL1/MATN2/SEMA3G |
| BP | neuron projection guidance | 0.031057876 | 0.02456794 | DPYSL2/SEMA6D/IRS2/CXCL12/LAMA2/ROBO4/CHL1/MATN2/SEMA3G |
| BP | ethanol metabolic process | 0.031057876 | 0.02456794 | ALDH1A1/ADH1C/ADH1B |
| BP | mesenchyme development | 0.031057876 | 0.02456794 | SEMA6D/TGFBR2/HOXA5/SPRY1/EDNRB/SEMA3G/MEOX1/TGFBR3/TMEM100 |
| BP | regulation of fibroblast proliferation | 0.031057876 | 0.02456794 | PDGFD/PDGFRA/AQP1/IGF1/PPARG |
| BP | negative regulation of cellular response to transforming growth factor beta stimulus | 0.031057876 | 0.02456794 | TGFBR2/CAV2/CAV1/TGFBR3/CIDEA |
| BP | muscle system process | 0.031057876 | 0.02456794 | ANK2/SCN4B/GSN/DMD/ADRB2/EDNRB/IGF1/CAV1/LMOD1/SYNM/FXYD1/LEP |
| BP | negative regulation of angiogenesis | 0.031057876 | 0.02456794 | MMRN2/ISM1/HOXA5/MEOX2/SPRY2/TNMD/PPARG |
| BP | negative regulation of transmembrane receptor protein serine/threonine kinase signaling pathway | 0.031057876 | 0.02456794 | TGFBR2/CAV2/CAV1/TGFBR3/CHRDL1/CIDEA |
| BP | fibroblast proliferation | 0.031057876 | 0.02456794 | PDGFD/PDGFRA/AQP1/IGF1/PPARG |
| BP | regulation of coagulation | 0.031057876 | 0.02456794 | PDGFRA/PROS1/TFPI/CAV1/CD36 |
| BP | regulation of heart growth | 0.031057876 | 0.02456794 | TGFBR2/FGF2/IGF1/TGFBR3/RBP4 |
| BP | positive regulation of lipid storage | 0.031486233 | 0.024906786 | LPL/CD36/CIDEA |
| BP | cellular response to low-density lipoprotein particle stimulus | 0.031486233 | 0.024906786 | PPARG/LPL/CD36 |
| BP | negative regulation of blood vessel morphogenesis | 0.031486233 | 0.024906786 | MMRN2/ISM1/HOXA5/MEOX2/SPRY2/TNMD/PPARG |
| BP | regulation of endothelial cell proliferation | 0.032164993 | 0.025443711 | CXCL12/FGF2/CAV2/CAV1/TNMD/PPARG/LEP |
| BP | lung morphogenesis | 0.032335831 | 0.02557885 | TGFBR2/HOXA5/SPRY1/SPRY2 |
| BP | cellular hormone metabolic process | 0.032335831 | 0.02557885 | PDGFRA/ALDH1A1/AKR1C3/ADH1C/RBP4/ADH1B |
| BP | muscle tissue development | 0.032335831 | 0.02557885 | PDGFRA/TGFBR2/HLF/FGF2/PGM5/CAV2/IGF1/MEOX2/CAV1/TGFBR3/RBP4 |
| BP | positive regulation of phosphatidylinositol 3-kinase signaling | 0.033088205 | 0.026174007 | PDGFD/PDGFRA/IRS2/IGF1/LEP |
| BP | positive regulation of fibroblast proliferation | 0.033129055 | 0.026206321 | PDGFD/PDGFRA/AQP1/IGF1 |
| BP | regulation of response to wounding | 0.033129055 | 0.026206321 | PDGFRA/PROS1/TGFBR2/FGF2/TFPI/CAV1/CD36 |
| BP | response to alcohol | 0.033790599 | 0.026729627 | TGFBR2/GSN/AKR1C3/TGFBR3/CDO1/PPARG/LEP/RBP4 |
| BP | regulation of fat cell differentiation | 0.034319894 | 0.027148318 | RUNX1T1/ID4/PPARG/ZBTB16/LPL/LEP |
| BP | roof of mouth development | 0.03455188 | 0.027331828 | PDGFRA/TGFBR2/MEOX2/TGFBR3/CLDN5 |
| BP | developmental growth involved in morphogenesis | 0.03455188 | 0.027331828 | DPYSL2/SEMA6D/NDN/CXCL12/TGFBR2/SPRY1/SEMA3G/SPRY2 |
| BP | morphogenesis of a branching epithelium | 0.034564958 | 0.027342174 | RSPO3/TGFBR2/HOXA5/SPRY1/FGF2/SPRY2/GPC3 |
| BP | membrane raft organization | 0.034688713 | 0.027440068 | GSN/CAV2/CAV1 |
| BP | trabecula formation | 0.034688713 | 0.027440068 | ADAMTS1/TGFBR3/RBP4 |
| BP | positive regulation of developmental growth | 0.035694194 | 0.028235441 | CXCL12/GHR/TGFBR2/FGF2/IGF1/TGFBR3/LEP |
| BP | muscle cell development | 0.037608195 | 0.029749488 | PDGFRA/ANK2/DMD/PGM5/CAV2/IGF1/LMOD1 |
| BP | negative regulation of vasculature development | 0.038399759 | 0.030375644 | MMRN2/ISM1/HOXA5/MEOX2/SPRY2/TNMD/PPARG |
| BP | alcohol metabolic process | 0.041019861 | 0.032448243 | FGF2/ALDH1A1/AKR1C3/IGF1/GPAM/ADH1C/LEP/RBP4/GPD1/ADH1B |
| BP | lipid transport | 0.041451929 | 0.032790024 | GULP1/ABCA5/IRS2/CAV1/ABCA6/PPARG/ABCA9/CD36/LEP/RBP4 |
| BP | endothelial cell proliferation | 0.042096819 | 0.033300157 | CXCL12/FGF2/CAV2/CAV1/TNMD/PPARG/LEP |
| BP | regulation of cardiac muscle cell proliferation | 0.043005724 | 0.034019134 | TGFBR2/FGF2/TGFBR3/RBP4 |
| BP | cellular response to fatty acid | 0.043005724 | 0.034019134 | AKR1C3/PPARG/PDK4/LPL |
| BP | hexose metabolic process | 0.043402841 | 0.034333269 | IRS2/ALDH1A1/PGM5/IGF1/PDK4/LEP/RBP4/GPD1 |
| BP | negative regulation of growth | 0.043402841 | 0.034333269 | SEMA6D/TGFBR2/ADRB2/SEMA3G/GPC3/PPARG/FHL1/RBP4 |
| BP | cellular response to transforming growth factor beta stimulus | 0.043402841 | 0.034333269 | PDGFD/TGFBR2/CDKN1C/CAV2/CAV1/TGFBR3/CLDN5/CIDEA |
| BP | positive regulation of cold-induced thermogenesis | 0.04343488 | 0.034358613 | ADRB2/CAV1/G0S2/CD36/LEP |
| BP | regulation of lipase activity | 0.045037201 | 0.035626109 | PDGFRA/ARHGAP6/FGF2/GPIHBP1/LPL |
| BP | morphogenesis of a branching structure | 0.045264692 | 0.035806063 | RSPO3/TGFBR2/HOXA5/SPRY1/FGF2/SPRY2/GPC3 |
| BP | multicellular organism growth | 0.045264692 | 0.035806063 | GHR/HOXA5/CDKN1C/ADRB2/TNS2/IGF1 |
| BP | digestive system development | 0.045264692 | 0.035806063 | CLMP/PDGFRA/TGFBR2/HOXA5/CDKN1C/EDNRB |
| BP | protein-lipid complex remodeling | 0.045264692 | 0.035806063 | ABCA5/GPIHBP1/LPL |
| BP | plasma lipoprotein particle remodeling | 0.045264692 | 0.035806063 | ABCA5/GPIHBP1/LPL |
| BP | regeneration | 0.045264692 | 0.035806063 | CXCL12/TGFBR2/GSN/MATN2/IGF1/TGFBR3/PPARG |
| BP | cholesterol transport | 0.045264692 | 0.035806063 | ABCA5/CAV1/PPARG/CD36/LEP |
| BP | regulation of cardiac muscle tissue development | 0.045264692 | 0.035806063 | TGFBR2/FGF2/IGF1/TGFBR3/RBP4 |
| BP | response to transforming growth factor beta | 0.045264692 | 0.035806063 | PDGFD/TGFBR2/CDKN1C/CAV2/CAV1/TGFBR3/CLDN5/CIDEA |
| BP | regulation of glucose import | 0.045264692 | 0.035806063 | IRS2/IGF1/GPC3/LEP |
| BP | transforming growth factor beta receptor signaling pathway | 0.045264692 | 0.035806063 | TGFBR2/CDKN1C/CAV2/CAV1/TGFBR3/CLDN5/CIDEA |
| BP | positive regulation of smooth muscle cell proliferation | 0.046350741 | 0.036665168 | PDGFD/TGFBR2/FGF2/IGF1/ADAMTS1 |
| BP | ERK1 and ERK2 cascade | 0.046350741 | 0.036665168 | PDGFD/PDGFRA/SPRY1/FGF2/NDRG2/AKAP12/IGF1/SPRY2/CD36 |
| BP | glycosaminoglycan catabolic process | 0.046685835 | 0.03693024 | FGF2/OGN/GPC3/LYVE1 |
| BP | protein-containing complex remodeling | 0.046685835 | 0.03693024 | ABCA5/GPIHBP1/LPL |
| BP | epithelial tube branching involved in lung morphogenesis | 0.046685835 | 0.03693024 | HOXA5/SPRY1/SPRY2 |
| BP | heart morphogenesis | 0.047204448 | 0.037340482 | DLC1/TGFBR2/SPRY1/ADAMTS1/TGFBR3/TMEM100/CLDN5/RBP4 |
| BP | branching morphogenesis of an epithelial tube | 0.04735012 | 0.037455714 | TGFBR2/HOXA5/SPRY1/FGF2/SPRY2/GPC3 |
| BP | negative regulation of cellular component movement | 0.047555174 | 0.037617919 | SEMA6D/DLC1/HOXA7/MMRN2/CXCL12/FGF2/SEMA3G/MEOX2/TGFBR3/PPARG |
| BP | positive regulation of transmembrane transport | 0.048305783 | 0.038211679 | IRS2/ANK2/DMD/ADRB2/IGF1/GPC3/FXYD1 |
| BP | regulation of peptidase activity | 0.048305783 | 0.038211679 | SOX7/PROS1/DLC1/GSN/AQP1/TFPI/CAV1/GPC3/PPARG/ITIH5/TIMP4 |
| BP | sensory perception of pain | 0.048410114 | 0.038294209 | NDN/CXCL12/MME/EDNRB/TMEM100 |
| BP | organic hydroxy compound transport | 0.048410114 | 0.038294209 | ABCA5/CXCL12/AQP1/CAV1/PPARG/CD36/LEP/RBP4 |
| BP | regulation of fatty acid oxidation | 0.048464013 | 0.038336845 | IRS2/PPARG/PDK4 |
| BP | plasma membrane organization | 0.049316653 | 0.039011315 | ANK2/PLSCR4/GSN/CAV2/CAV1 |
| BP | cardiac muscle tissue growth | 0.049316653 | 0.039011315 | TGFBR2/FGF2/IGF1/TGFBR3/RBP4 |
| CC | collagen-containing extracellular matrix | 0.000100076 | 8.23E-05 | ECM2/FREM1/CTSG/EFEMP1/MMRN2/CXCL12/LAMA2/VWF/ADAMTS5/MATN2/ADAMTS1/OGN/SRPX/GPC3/ITIH5/DPT |
| CC | sarcolemma | 0.00028777 | 0.000236745 | ANK2/LAMA2/AQP1/DMD/PGM5/CAV2/CAV1/SYNM/FXYD1 |
| CC | costamere | 0.001283219 | 0.001055689 | ANK2/DMD/PGM5/SYNM |
| CC | caveola | 0.002854978 | 0.002348755 | DLC1/TGFBR2/TFPI/CAV2/CAV1/FXYD1 |
| CC | lipid droplet | 0.002854978 | 0.002348755 | CAV1/G0S2/CIDEA/PLIN4/PLIN1/CIDEC |
| CC | membrane raft | 0.002854978 | 0.002348755 | ANK2/DLC1/TGFBR2/DMD/TFPI/MME/CAV2/EDNRB/CAV1/FXYD1/CD36 |
| CC | membrane microdomain | 0.002854978 | 0.002348755 | ANK2/DLC1/TGFBR2/DMD/TFPI/MME/CAV2/EDNRB/CAV1/FXYD1/CD36 |
| CC | membrane region | 0.003467347 | 0.002852544 | ANK2/DLC1/TGFBR2/DMD/TFPI/MME/CAV2/EDNRB/CAV1/FXYD1/CD36 |
| CC | plasma membrane raft | 0.008375175 | 0.006890154 | DLC1/TGFBR2/TFPI/CAV2/CAV1/FXYD1 |
| CC | intercalated disc | 0.017259841 | 0.01419946 | ANK2/SCN4B/PGM5/FXYD1 |
| CC | cell-substrate junction | 0.017259841 | 0.01419946 | DLC1/GSN/TNS1/DMD/PGM5/TNS2/MME/CAV2/AKAP12/CAV1/FHL1 |
| CC | platelet alpha granule | 0.019727977 | 0.016229966 | PROS1/VWF/IGF1/CD36/CFD |
| CC | basement membrane | 0.02207149 | 0.018157945 | FREM1/MMRN2/LAMA2/MATN2/ADAMTS1 |
| CC | external side of plasma membrane | 0.032787565 | 0.026973929 | PDGFRA/CXCL12/GHR/TGFBR2/LIFR/TGFBR3/GPIHBP1/CD36/CA4/BTNL9 |
| CC | platelet alpha granule lumen | 0.035342159 | 0.029075563 | PROS1/VWF/IGF1/CFD |
| CC | focal adhesion | 0.035342159 | 0.029075563 | DLC1/GSN/TNS1/PGM5/TNS2/MME/CAV2/AKAP12/CAV1/FHL1 |
| CC | cell-substrate adherens junction | 0.035342159 | 0.029075563 | DLC1/GSN/TNS1/PGM5/TNS2/MME/CAV2/AKAP12/CAV1/FHL1 |
| CC | cell-cell contact zone | 0.037859568 | 0.031146604 | ANK2/SCN4B/PGM5/FXYD1 |
| MF | glycosaminoglycan binding | 0.002774008 | 0.002532195 | CFH/ECM2/CTSG/RSPO3/TGFBR2/FGF2/ADAMTS5/ADAMTS1/TGFBR3/LYVE1/LPL |
| MF | heparin binding | 0.004281875 | 0.003908619 | CFH/ECM2/CTSG/RSPO3/FGF2/ADAMTS5/ADAMTS1/TGFBR3/LPL |
| MF | extracellular matrix structural constituent | 0.014578395 | 0.013307581 | EFEMP1/MMRN2/LAMA2/VWF/MATN2/OGN/SRPX/DPT |
| MF | growth factor binding | 0.02302277 | 0.021015851 | PDGFRA/GHR/TGFBR2/LIFR/TGFBR3/IGFBP6/CD36 |
| MF | sulfur compound binding | 0.033695081 | 0.030757845 | CFH/ECM2/CTSG/RSPO3/FGF2/ADAMTS5/ADAMTS1/TGFBR3/LPL |
| MF | retinol dehydrogenase activity | 0.046407692 | 0.042362285 | AKR1C3/ADH1C/ADH1B |
