# Supplementary material for: Identification of hub genes and small molecule therapeutic drugs related to breast cancer with comprehensive bioinformatics analysis
Source: PeerJ. 2020 Sep 29;8:e9946. doi: 10.7717/peerj.9946 (PMC7556247; doi:10.7717/peerj.9946)
Supplement: Supplemental Information 19 [file peerj-08-9946-s019.docx]

| **ID** | **Description** | **p.adjust** | **qvalue** | **geneID** |
| --- | --- | --- | --- | --- |
| hsa04110 | Cell cycle | 0.000592602 | 0.000537325 | CDC20/PTTG1/CCNB2/CCNB1/CDK1/MAD2L1/BUB1B/CCNA2/TTK/CCNE2/CDKN1C |
| hsa04114 | Oocyte meiosis | 0.012178689 | 0.011042689 | CDC20/PTTG1/CCNB2/CCNB1/CDK1/AURKA/MAD2L1/CCNE2/IGF1 |
| hsa00350 | Tyrosine metabolism | 0.012178689 | 0.011042689 | IL4I1/MAOA/AOC3/ADH1C/ADH1B |
| hsa04512 | ECM-receptor interaction | 0.020034735 | 0.01816594 | FN1/HMMR/SPP1/FREM1/LAMA2/VWF/CD36 |
| hsa04914 | Progesterone-mediated oocyte maturation | 0.029484196 | 0.026733976 | CCNB2/CCNB1/CDK1/AURKA/MAD2L1/CCNA2/IGF1 |
| hsa04115 | p53 signaling pathway | 0.029484196 | 0.026733976 | RRM2/CCNB2/CCNB1/CDK1/CCNE2/IGF1 |
| hsa03320 | PPAR signaling pathway | 0.033479778 | 0.03035686 | MMP1/PPARG/LPL/CD36/PLIN4/PLIN1 |
| hsa00360 | Phenylalanine metabolism | 0.043797316 | 0.039712001 | IL4I1/MAOA/AOC3 |
