# Supplementary material for: Identification of hub genes and small molecule therapeutic drugs related to breast cancer with comprehensive bioinformatics analysis
Source: PeerJ. 2020 Sep 29;8:e9946. doi: 10.7717/peerj.9946 (PMC7556247; doi:10.7717/peerj.9946)
Supplement: Supplemental Information 20 [file peerj-08-9946-s020.docx]

| **miRNA_ID** | **Log2FC** | **adj.P.Val** |
| --- | --- | --- |
| hsa-miR-21-5p | 3.1963564 | 5.76E-14 |
| hsa-miR-182-5p | 2.8486358 | 5.76E-14 |
| hsa-miR-96-5p | 2.7882002 | 8.75E-13 |
| hsa-miR-425-5p | 2.7723758 | 5.76E-14 |
| hsa-miR-429 | 2.2539269 | 2.18E-09 |
| hsa-miR-22-3p | 2.0155372 | 7.07E-09 |
| hsa-miR-183-5p | 1.8577856 | 8.41E-05 |
| hsa-miR-21-3p | 1.8566015 | 2.59E-10 |
| hsa-miR-301a-3p | 1.833941 | 6.16E-03 |
| hsa-miR-142-3p | 1.7765397 | 1.65E-05 |
| hsa-miR-491-3p | 1.7559535 | 2.91E-12 |
| hsa-miR-1298-5p | 1.6801959 | 3.49E-11 |
| hsa-miR-200a-3p | 1.6757768 | 1.44E-05 |
| hsa-miR-3916 | 1.5924044 | 7.03E-10 |
| hsa-miR-141-3p | 1.5503659 | 2.34E-07 |
| hsa-miR-155-5p | 1.5135281 | 2.74E-06 |
| hsa-miR-4633-5p | 1.5029573 | 3.49E-11 |
| hsa-miR-18b-5p | 1.4965699 | 2.80E-03 |
| hsa-miR-22-5p | 1.4948682 | 1.56E-04 |
| hsa-miR-5195-5p | 1.4654575 | 1.41E-11 |
| hsa-miR-193b-3p | 1.4604255 | 3.33E-06 |
| hsa-miR-15b-5p | 1.445268 | 8.09E-07 |
| hsa-miR-106b-5p | 1.4444365 | 1.23E-08 |
| hsa-miR-3907 | 1.4273784 | 9.45E-11 |
| hsa-miR-142-5p | 1.412174 | 8.82E-04 |
| hsa-miR-185-5p | 1.4035179 | 5.76E-14 |
| hsa-miR-4284 | 1.3898454 | 1.39E-08 |
| hsa-miR-107 | 1.3847113 | 1.23E-08 |
| hsa-miR-103a-3p | 1.3690702 | 5.49E-09 |
| hsa-miR-191-5p | 1.3681976 | 7.43E-06 |
| hsa-miR-4435 | 1.3580963 | 1.74E-11 |
| hsa-miR-1289 | 1.3580731 | 2.73E-07 |
| hsa-miR-342-3p | 1.3413583 | 4.25E-04 |
| hsa-miR-605-5p | 1.302097 | 1.29E-12 |
| hsa-miR-15a-5p | 1.2977948 | 6.13E-05 |
| hsa-miR-422a | 1.2815052 | 2.51E-11 |
| hsa-miR-93-5p | 1.2511806 | 6.46E-08 |
| hsa-miR-27a-3p | 1.2495107 | 7.09E-05 |
| hsa-miR-4291 | 1.2375607 | 3.93E-08 |
| hsa-miR-29b-3p | 1.2315698 | 1.03E-03 |
| hsa-miR-340-5p | 1.2282425 | 1.48E-02 |
| hsa-miR-196a-5p | 1.2221055 | 4.44E-02 |
| hsa-miR-331-3p | 1.2086504 | 6.46E-08 |
| hsa-miR-200b-3p | 1.1764002 | 6.57E-05 |
| hsa-miR-365b-3p | 1.1402192 | 1.23E-08 |
| hsa-miR-365a-3p | 1.1402192 | 1.23E-08 |
| hsa-miR-27b-3p | 1.0830577 | 4.35E-04 |
| hsa-miR-1973 | 1.0713962 | 2.18E-09 |
| hsa-miR-18a-5p | 1.0628703 | 4.59E-02 |
| hsa-miR-339-5p | 1.0568744 | 6.33E-08 |
| hsa-miR-6765-3p | 1.0350083 | 5.55E-06 |
| hsa-miR-138-2-3p | 1.0320055 | 1.15E-07 |
| hsa-miR-29c-3p | 1.0240639 | 1.23E-02 |
| hsa-miR-212-3p | 1.0171166 | 1.74E-11 |
| hsa-miR-4317 | 1.0141854 | 1.66E-05 |
| hsa-miR-200c-3p | 1.0128167 | 1.26E-06 |
| hsa-miR-4289 | 1.0042399 | 8.03E-08 |
| hsa-miR-193a-3p | 1.0032551 | 3.34E-04 |
| hsa-miR-3160-5p | -1.0030595 | 5.30E-04 |
| hsa-miR-132-3p | -1.0390487 | 8.92E-04 |
| hsa-miR-410-5p | -1.0527721 | 1.95E-02 |
| hsa-miR-518c-3p | -1.072895 | 6.57E-03 |
| hsa-miR-8058 | -1.0855002 | 3.86E-02 |
| hsa-miR-4525 | -1.1021134 | 5.10E-06 |
| hsa-miR-5087 | -1.1084853 | 9.05E-03 |
| hsa-miR-329-5p | -1.1248353 | 1.91E-02 |
| hsa-miR-3194-3p | -1.1261004 | 2.92E-03 |
| hsa-miR-101-5p | -1.1265115 | 3.84E-03 |
| hsa-miR-4480 | -1.1303287 | 3.51E-07 |
| hsa-miR-1295a | -1.1351446 | 1.75E-05 |
| hsa-miR-4328 | -1.1602997 | 3.04E-05 |
| hsa-miR-497-5p | -1.180138 | 4.20E-05 |
| hsa-miR-145-3p | -1.194078 | 2.14E-02 |
| hsa-miR-6866-5p | -1.2191064 | 1.95E-02 |
| hsa-miR-4718 | -1.2489806 | 1.86E-04 |
| hsa-miR-195-5p | -1.258666 | 2.86E-04 |
| hsa-miR-106a-3p | -1.2748191 | 2.14E-02 |
| hsa-miR-5704 | -1.3163228 | 1.65E-02 |
| hsa-miR-204-5p | -1.3373266 | 2.06E-08 |
| hsa-miR-548d-5p | -1.3624453 | 1.47E-02 |
| hsa-miR-130a-3p | -1.380714 | 5.10E-05 |
| hsa-miR-221-5p | -1.4705959 | 2.57E-02 |
| hsa-miR-4263 | -1.4845039 | 5.77E-05 |
| hsa-miR-4771 | -1.4898481 | 9.05E-05 |
| hsa-miR-145-5p | -1.5514101 | 6.68E-06 |
| hsa-miR-132-5p | -1.5703162 | 2.46E-02 |
| hsa-miR-100-5p | -1.5903243 | 5.82E-05 |
| hsa-let-7a-3p | -1.5951308 | 2.95E-02 |
| hsa-miR-647 | -1.6329451 | 3.21E-02 |
| hsa-miR-6733-5p | -1.6866793 | 4.51E-02 |
| hsa-miR-3168 | -1.752769 | 1.41E-02 |
| hsa-miR-580-5p | -1.7546744 | 4.12E-02 |
| hsa-miR-6512-5p | -1.8124969 | 3.10E-02 |
| hsa-miR-125b-5p | -1.8140948 | 3.50E-07 |
| hsa-miR-99a-5p | -1.8210494 | 1.02E-05 |
| hsa-miR-4324 | -1.8918832 | 1.46E-07 |
| hsa-miR-205-5p | -2.2883081 | 3.01E-04 |
| hsa-miR-4772-5p | -2.4507465 | 2.40E-02 |
| hsa-miR-136-3p | -2.4991519 | 2.09E-02 |
| hsa-miR-4650-5p | -3.060994 | 1.02E-02 |
| hsa-miR-4645-3p | -3.7748159 | 4.11E-03 |
| hsa-miR-208b-5p | -4.5429333 | 1.56E-02 |
| hsa-miR-219a-5p | -4.6738056 | 1.58E-02 |
