# Supplementary material for: Identification of hub genes and small molecule therapeutic drugs related to breast cancer with comprehensive bioinformatics analysis
Source: PeerJ. 2020 Sep 29;8:e9946. doi: 10.7717/peerj.9946 (PMC7556247; doi:10.7717/peerj.9946)
Supplement: Supplemental Information 21 [file peerj-08-9946-s021.docx]

| **Compound name** | **Dose** | **Time** | **Genes with opposite expression based on overlapping DEGs** | |
| --- | --- | --- | --- | --- |
|  |  |  | **Up** | **Down** |
| 1β-hydroxyalantolactone | 10μM | 12 h | ZNF423/GULP1/CRYBG3/MEIS2/PLAGL1/VWF/HLF/AKR1C3/AKAP12/ID4/ENPP2/PPARG/ITIH5 | CENPF/RRM2/NEK2/PTTG1/NUSAP1/CCNB2/KIF20A/PBK/PAFAH1B3/SQLE/MAD2L1/TYMS/CCNA2/TRIP13/STMN1/CCNE2/KIF26B/PSRC1 |
| Andrographolide | 10μM | 12 h | AKR1C3/MATN2/LDB2 | - |
| Berberine hydrochloride | 10μM | 12 h | CA4/HOXA5/AMOTL2/CFH/LDB2/PCNX1/THRB/PLPP3/TGFBR2/SPRY1 | CENPM/TACC3/CDCA8/TK1/FANCI/JPT1/E2F8/FOXM1/LMNB1/NDC80/KIF20A/GINS1/CDC20/KIF11/HMMR/CCNE2/GINS2/KIF4A/ZWINT/CENPF/PRC1/PBK/CDKN3/MELK/CCNB2/DTL/BIRC5/ASPM/RRM2 |
| Britanin | 10μM | 12 h | ZNF423/AMOTL2/CFH/PLAGL1/TNS1/AKR1C3/ADAMTS1/PPARG | TOP2A/CENPF/RRM2/PTTG1/CCNB2/MKI67/ZWINT/KIF20A/INHBA/PBK/HMGB3/CDCA3/BUB1B/TYMS/CCNA2/GINS1/TRIP13/SPAG5/STMN1/RAD51AP1/FANCI/E2F8/ZNF423/AMOTL2/CFH/PLAGL1/TNS1/AKR1C3/ADAMTS1/PPARG |
| Hyodeoxycholic acid | 10μM | 12 h | PLAGL1/TGFBR2 | LMNB1 |
| Japonicone A | 10μM | 12 h | ZNF423/AMOTL2/GULP1/ABCA5/FAM13A/TGFBR2/GSN/LDB2/CDKN1C/TFPI/AKR1C3/MATN2/ID4/PPARG/SYNM/IGFBP6/ZBTB16/GPX3 | UBE2C/TOP2A/CENPF/RRM2/NEK2/TPX2/BIRC5/CDC20/PTTG1/NUSAP1/CCNB2/MKI67/ZWINT/CKS2/KIF20A/CCNB1/FOXM1/INHBA/PBK/LMNB1/CDK1/CEP55/MELK/AURKA/KIF4A/CDKN3/DTL/KIF2C/KIF11/DLGAP5/HMMR/CENPM/HMGB3/CDCA8/NDC80/CDCA3/NCAPG/ASPM/CENPU/HJURP/PRC1/MAD2L1/BUB1B/TYMS/KIF23/RACGAP1/CCNA2/GINS1/UBE2S/TRIP13/CDT1/TTK/SPAG5/ATAD2/GINS2/STMN1/TACC3/ECT2/RAD51AP1/CCNE2/KIF18B/DEPDC1/FANCI/RNASEH2A/SHCBP1/EZH2/STIL/E2F8/PSRC1 |
| Nitidine chloride | 10μM | 12 h | HLF/HOXA5/IGFBP6/SYNM/NDRG2/CFH/PLAGL1/LDB2/TGFBR2/SPRY1 | RACGAP1/STIL/UBE2S/MKI67/ECT2/CDCA3/LMNB1/TTK/NDC80/CCNA2/MAD2L1/DLGAP5/KIF11/HMMR/INHBA/KIF4A/CENPF/PTTG1/CCNB1/EZH2/CDKN3/MELK/BUB1B/TPX2/CCNB2/DTL/ASPM |
| Tanshinone IIA | 10μM | 12 h | VWF | CENPM/SPAG5/E2F8/KIF20A/GINS1/DLGAP5/CDC20/HMMR/GINS2/KIF4A/CDK1/PTTG1/CDKN3/AURKA/DTL/BIRC5/ASPM/RRM2 |
