# Supplementary material for: Identification of hub genes and small molecule therapeutic drugs related to breast cancer with comprehensive bioinformatics analysis
Source: PeerJ. 2020 Sep 29;8:e9946. doi: 10.7717/peerj.9946 (PMC7556247; doi:10.7717/peerj.9946)
Supplement: Supplemental Information 2 — (A) A PPI network was constructed via STRING. (B) six hub genes were identified by MCC, MNC and Degree methods. [file peerj-08-9946-s002.pdf]

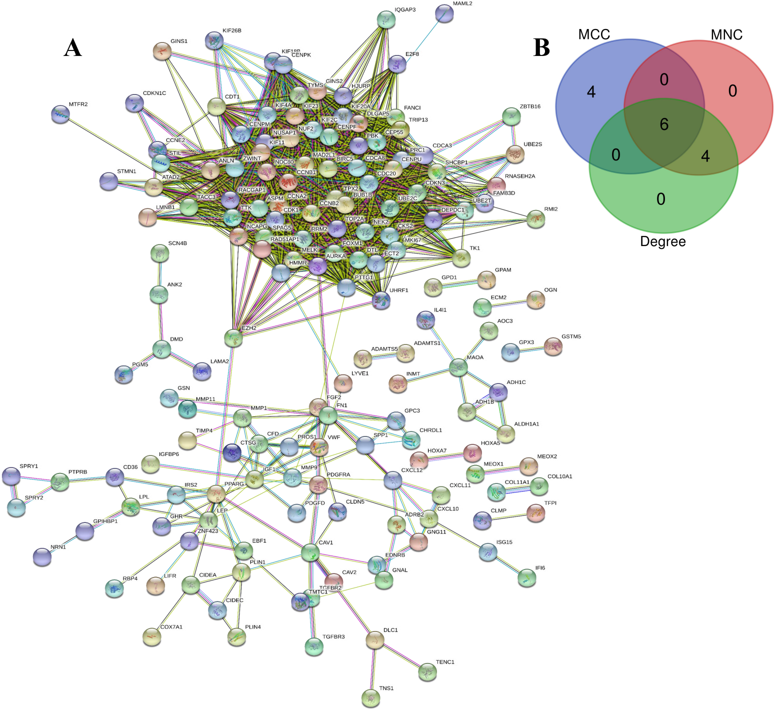
A
IQGAP3
MAML2
KIF26B
GINS1
KIF18B
CENPK
E2F8
CDKN1C
CDT1
TYMS
GINS2
HJURP
MTFR2
KIF4A
KIF23
KIF20A
DLGAP5
FANCI
ZBTB16
CENPM
KIF2C
TRIP13
NUSAP1
NUF2
CENPF
CCNE2
PBK
CEP55
UBE2S
KIF11
STIL
PRC1
CDCA3
MAD2L1
ZWINT
ANLN
BIRC5
CDCA8
CENPU
SHCBP1
STMN1
NDC80
CCNB1
CDC20
ATAD2
RNASEH2A
RACGAP1
TPX2
CDKN3
FAM83D
BUB1B
TACC3
ASPM
CCNA2
UBE2C
UBE2T
LMNB1
TTK
CDK1
CCNB2
DEPDC1
RMI2
TOP2A
NCAPG
NEK2
SPAG5
RRM2
CKS2
RAD51AP1
MKI67
FOXM1
MELK
TK1
DTL
ECT2
HMMR
AURKA
PTTG1
SCN4B
UHRF1
GPD1
GPAM
ANK2
ECM2
OGN
IL4I1
EZH2
AOC3
DMD
GPX3
GSTM5
ADAMTS5
ADAMTS1
MAOA
PGM5
LAMA2
LYVE1
INMT
ADH1C
GSN
MMP11
FGF2
ADH1B
MMP1
FN1
GPC3
ALDH1A1
TIMP4
CFD
CHRDL1
SPP1
PROS1
VWF
CTSG
HOXA5
HOXA7
IGFBP6
MEOX2
CD36
MMP9
CXCL12
SPRY1
PTPRB
IGF1
PDGFRA
MEOX1
COL10A1
COL11A1
IRS2
PPARG
CXCL11
SPRY2
LPL
PDGFD
TFPI
LEP
CLDN5
CXCL10
CLMP
GHR
GPIHBP1
ADRB2
ZNF423
GNG11
NRN1
EBF1
CAV1
ISG15
EDNRB
PLIN1
GNAL
IFI6
CAV2
LIFR
RBP4
CIDEA
TGFBR2
TMTC1
CIDEC
COX7A1
PLIN4
DLC1
TGFBR3
TENC1
TNS1
B
MCC
MNC
Degree
4
0
0
6
0
4
0
